# Supplementary material for: Structure-Activity Relationship Study of Majusculamide D: Overcoming Metabolic Instability and Severe Toxicity with a Fluoro Analogue
Source: Mar Drugs. 2024 Nov 29;22(12):537. doi: 10.3390/md22120537 (PMC11679793; doi:10.3390/md22120537)
Supplement: Supplementary file 1 [file marinedrugs-22-00537-s001.zip › marinedrugs-3287802-supplementary.pdf]

Structure-Activity Relationship Study of Majusculamide D: Overcoming Metabolic Instability and Severe Toxicity with a Fluoro Analogue

Xiuhe Zhao [1], Xiaonan Xi [1,*], Mingxiao Zhang [1], Mengxue Lv [1], Xiang Zhang [2], Yaxin Lu [2], Liang Wang [2,*] and Yue Chen [2,3,*]

[1] The State Key Laboratory of Medicinal Chemical Biology, College of Pharmacy, Nankai University, Tianjin, 300350, People's Republic of China; 15522215338@163.com (X.Zhao.); x18716062989@163.com (M.Z.); mengxuelv@qq.com (M.L.)

[2] College of Chemistry, Nankai University, Tianjin, 300071, People's Republic of China; 2120231187@mail.nankai.edu.cn (X.Zhang.); yaxinlu@nankai.edu.cn (Y.L.)

[3] Haihe Laboratory of Sustainable Chemical Transformations, Tianjin, 300192, People's Republic of China

* Correspondence: xi_xiaonan@foxmail.com (X.X.); lwang@nankai.edu.cn (L.W.); yuechen@nankai.edu.cn (Y.C.)

Content of Supporting Information

Part 1. Synthetic procedures and characterization data (page 5 to 59)
Figure S1-S2. NMR spectrum of compound **17**
Figure S3-S4. NMR spectrum of compound **8b**
Figure S5-S6. NMR spectrum of compound **3b**
Figure S7-S8. NMR spectrum of compound **13b**
Figure S9-S10. NMR spectrum of compound **1b**
Figure S11-S12. NMR spectrum of compound **14a**
Figure S13-S15. NMR spectrum of compound **6c**
Figure S16-S18. NMR spectrum of compound **8c**
Figure S19-S21. NMR spectrum of compound **3c**
Figure S22-S24. NMR spectrum of compound **13c**
Figure S25-S27. NMR spectrum of compound **1c**
Figure S28-S29. NMR spectrum of compound **6d**
Figure S30-S31. NMR spectrum of compound **8d**
Figure S32-S33. NMR spectrum of compound **3d**
Figure S34-S35. NMR spectrum of compound **13d**
Figure S36-S37. NMR spectrum of compound **1d**
Figure S38-S39. NMR spectrum of compound **19**
Figure S40-S41. NMR spectrum of compound **20**
Figure S42-S43. NMR spectrum of compound **21**
Figure S44-S45. NMR spectrum of compound **22**
Figure S46-S47. NMR spectrum of compound **6e**
Figure S48-S49. NMR spectrum of compound **8e**
Figure S50-S51. NMR spectrum of compound **3e**
Figure S52-S53. NMR spectrum of compound **13e**
Figure S54-S55. NMR spectrum of compound **1e**
Figure S56-S57. NMR spectrum of compound **23**
Figure S58-S59. NMR spectrum of compound **24**
Figure S60-S61. NMR spectrum of compound **6f**
Figure S62-S63. NMR spectrum of compound **8f**
Figure S64-S65. NMR spectrum of compound **3f**
Figure S66-S67. NMR spectrum of compound **13f**
Figure S68-S69. NMR spectrum of compound **1f**
Figure S70-S71. NMR spectrum of compound **14b**
Figure S72-S73. NMR spectrum of compound **26**
Figure S74-S75. NMR spectrum of compound **27**
Figure S76-S77. NMR spectrum of compound **7b**
Figure S78-S79. NMR spectrum of compound **29**
Figure S80-S81. NMR spectrum of compound **30**
Figure S82-S83. NMR spectrum of compound **31**
Figure S84-S85. NMR spectrum of compound **3g**

Figure S86-S87. NMR spectrum of compound **13g**
Figure S88-S89. NMR spectrum of compound **1g**
Figure S90-S91. NMR spectrum of compound **14c**
Figure S92-S93. NMR spectrum of compound **7c**
Figure S94-S95. NMR spectrum of compound **8h**
Figure S96-S97. NMR spectrum of compound **3h**
Figure S98-S99. NMR spectrum of compound **13h**
Figure S100-S101. NMR spectrum of compound **1h**
Figure S102-S104. NMR spectrum of compound **7d**
Figure S105-S107. NMR spectrum of compound **8i**
Figure S108-S110. NMR spectrum of compound **3i**
Figure S111-S113. NMR spectrum of compound **13i**
Figure S114-S116. NMR spectrum of compound **1i**
Figure S117-S118. NMR spectrum of compound **7e**
Figure S119-S120. NMR spectrum of compound **8j**
Figure S121-S122. NMR spectrum of compound **3j**
Figure S123-S124. NMR spectrum of compound **13j**
Figure S125-S126. NMR spectrum of compound **1j**
Figure S127-S129. NMR spectrum of compound **10b**
Figure S130-S132. NMR spectrum of compound **12b**
Figure S133-S135. NMR spectrum of compound **4b**
Figure S136-S138. NMR spectrum of compound **13k**
Figure S139-S141. NMR spectrum of compound **1k**
Figure S142-S143. NMR spectrum of compound **12c**
Figure S144-S145. NMR spectrum of compound **4c**
Figure S146-S147. NMR spectrum of compound **13l**
Figure S148-S149. NMR spectrum of compound **1l**
Figure S150-S151. NMR spectrum of compound **12d**
Figure S152-S153. NMR spectrum of compound **4d**
Figure S154-S155. NMR spectrum of compound **13m**
Figure S156-S157. NMR spectrum of compound **1m**
Figure S158-S159. NMR spectrum of compound **15a**
Figure S160-S161. NMR spectrum of compound **16a**
Figure S162-S163. NMR spectrum of compound **15b**
Figure S164-S165. NMR spectrum of compound **16b**
Figure S166-S167. NMR spectrum of compound **15c**
Figure S168-S169. NMR spectrum of compound **16c**
Figure S170-S171. NMR spectrum of compound **15d**
Figure S172-S173. NMR spectrum of compound **16d**
Figure S174-S175. NMR spectrum of compound **15e**
Figure S176-S177. NMR spectrum of compound **16e**
Figure S178-S179. NMR spectrum of compound **15f**
Figure S180-S181. NMR spectrum of compound **16f**
Figure S182-S183. NMR spectrum of compound **15g**

Figure S184-S185. NMR spectrum of compound **16g**
Figure S186-S187. NMR spectrum of compound **15h**
Figure S188-S189. NMR spectrum of compound **16h**
Figure S190-S191. NMR spectrum of compound **15i**
Figure S192-S193. NMR spectrum of compound **16i**
Figure S194-S195. NMR spectrum of compound **15j**
Figure S196-S197. NMR spectrum of compound **16j**
Figure S198-S199. NMR spectrum of compound **15k**
Figure S200-S201. NMR spectrum of compound **16k**
Figure S202-S203. NMR spectrum of compound **15l**
Figure S204-S205. NMR spectrum of compound **16l**
Figure S206-S207. NMR spectrum of compound **15m**
Figure S208-S209. NMR spectrum of compound **16m**
Figure S210-S212. NMR spectrum of compound **8n**
Figure S213-S215. NMR spectrum of compound **3n**
Figure S216-S218. NMR spectrum of compound **13n**
Figure S219-S221. NMR spectrum of compound **1n**
Figure S222. HPLC analysis of compound **1a**
Figure S223. HPLC analysis of compound **1c**
Figure S224. HPLC analysis of compound **1n**
Figure S225. Compound **1a** inhibited the migration, invasion and proliferation of pancreatic cancer cells

1. Synthetic Procedures and Characterization Data

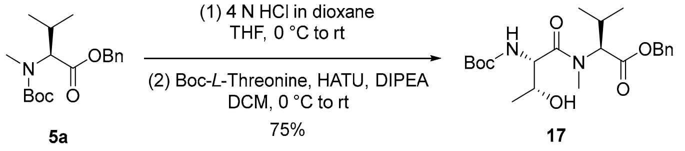

Synthesis of (17). To the solution of **5a** (5.53 g, 17.2 mmol) in THF (20 mL) was added 4 N HCl in dioxane (21.5 mL, 86.0 mmol) at 0 °C. The mixture was allowed to reach room temperature, stirred for 1 h, and concentrated under reduced pressure to provide as a white solid. To this salt was added a solution of Boc-L-Threonine (3.96 g, 18.07 mmol) and HATU (9.81 g, 25.8 mmol) in DCM (50 mL), then DIPEA (8.5 mL, 51.6 mmol) was added dropwise at 0 °C. The reaction was allowed to reach room temperature and stirred overnight. The mixture was concentrated, diluted with EtOAc (100 mL) and washed with aqueous 5% $NaHSO_4$ (3 × 100 mL) and brine (100 mL), dried (Na_2SO_4), filtered, concentrated in vacuo and purified by column chromatography (25/1 to 5/1 petroleum ether/EtOAc) to provide compound **17** (5.45 g, 75%) as a white solid. $[\alpha]_D^{22}$ = −114.8 (*c* = 0.5, $CHCl_3$). **^{1}H NMR** (400 MHz, $CDCl_3$) *δ* 7.31 (s, 5H), 5.46 (d, *J* = 9.3 Hz, 1H), 5.18 (d, *J* = 12.2 Hz, 1H), 5.07 (d, *J* = 12.2 Hz, 1H), 4.88 (d, *J* = 10.5 Hz, 1H), 4.43 (dd, *J* = 20.6, 9.8 Hz, 1H), 3.95 (d, *J* = 6.9 Hz, 1H), 3.74 (s, 1H), 3.00 (s, 3H), 2.21 (m, 1H), 1.40 (s, 9H), 1.11 (d, *J* = 6.2 Hz, 3H), 0.97 (d, *J* = 6.7 Hz, 3H), 0.81 (d, *J* = 6.8 Hz, 3H). **^{13}C NMR** (100 MHz, $CDCl_3$) *δ* 173.6, 170.3, 156.2, 135.5, 128.7, 128.6, 128.5, 128.5, 128.5, 128.4, 128.4, 128.0, 80.0, 67.3, 66.7, 61.7, 53.7, 31.6, 28.3, 28.2, 28.2, 27.3, 19.8, 18.7, 18.6. **HRMS (ESI)** *m/z*: calcd for $C_{22}H_{34}N_2O_6Na^+$ $[M+Na]^+$: 445.2309, found: 445.2304.

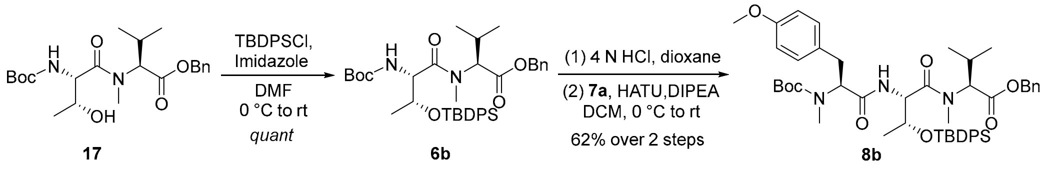

Synthesis of (8b). To the solution of **17** (5.00 g, 11.83 mmol) in DMF (50 mL) was added TBDPS-Cl (3.7 mL, 14.20 mmol) and imidazole (2.00 g, 29.58 mmol) at 0 °C. The reaction was allowed to reach room temperature, and stirred overnight. The mixture was extracted with EtOAc (100 mL) first, then washed with brine (5 × 100 mL), dried (Na_2SO_4), filtered, concentrated in vacuo.

To the solution of **6b** crude in THF (20 mL) was added 4 N HCl in dioxane (29.8 mL, 119.2 mmol) at 0 °C. The mixture was allowed to reach room temperature, stirred for 1 h, and concentrated under reduced pressure to provide as a white solid. To this salt was added a solution of **7a** (4.06 g, 13.11 mmol) and HATU (6.80 g, 17.88 mmol) in DCM (50 mL), then DIPEA (5.9 mL, 35.77 mmol) was added dropwise at 0 °C. The reaction was allowed to reach room

temperature and stirred overnight. The mixture was concentrated, diluted with EtOAc (100 mL) and washed with aqueous 5% $NaHSO_4$ (3 × 100 mL) and brine (100 mL), dried (Na_2SO_4), filtered, concentrated in vacuo and purified by column chromatography (15/1 to 2/1 petroleum ether/EtOAc) to provide compound **8b** (6.34 g, 62%) as a pale yellow oil. $[\alpha]_D^{25}$ = −54.8 (*c* = 1.0, $CHCl_3$). **^{1}H NMR** (400 MHz, $CDCl_3$) *δ* 7.71 – 7.64 (m, 4H), 7.45 – 7.35 (m, 6H), 7.29 (s, 5H), 7.10 (m, 2H), 6.80 (d, *J* = 8.1 Hz, 3H), 5.15 (d, *J* = 8.9 Hz, 1H), 5.01 (d, *J* = 12.2 Hz, 2H), 4.94 (dd, *J* = 8.3, 3.5 Hz, 1H), 4.18 (s, 1H), 3.75 (s, 3H), 3.20 (dd, *J* = 14.6, 5.1 Hz, 1H), 3.09 (s, 3H), 2.97 – 2.84 (m, 2H), 2.78 – 2.66 (m, 3H), 2.22 (s, 1H), 1.41 – 1.21 (m, 9H), 1.01 (s, 9H), 0.96 (d, *J* = 6.2 Hz, 3H), 0.90 (d, *J* = 6.2 Hz, 3H), 0.85 (d, *J* = 5.8 Hz, 3H). **^{13}C NMR** (100 MHz, $CDCl_3$) *δ* 170.9, 170.7, 169.6, 158.3, 156.0, 155.2, 135.9, 135.8, 134.0, 132.8, 130.1, 130.0, 129.8, 128.6, 128.5, 128.4, 128.4, 127.9, 127.7, 114.0, 113.8, 80.5, 80.1, 70.3, 67.1, 66.6, 61.7, 61.5, 60.1, 55.3, 54.9, 52.2, 33.3, 31.6, 29.0, 28.2, 28.2, 27.8, 26.9, 21.6, 20.5, 20.3, 19.7, 19.6, 19.3, 19.3, 19.1. **HRMS (ESI)** *m*/*z*: calcd for $C_{49}H_{66}N_3O_8Si^+$ [M+Na]$^+$: 852.4614, found: 852.4614.

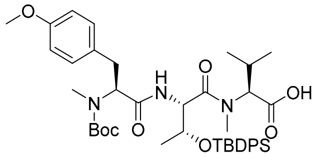

3b

Synthesis of (3b). To the solution of **8b** (6.22 g, 7.30 mmol) in EtOH (50 mL) was added Pd/C 10% Wt (620 mg) at 0 °C. The reaction vessel was evacuated/backfilled with argon three times first, then the reaction vessel was evacuated/backfilled with H_2 three times and stirred under this atmosphere overnight. The mixture was filtered through a pad of celite, washed with MeOH, and concentrated in vacuo and purified by column chromatography (100/1 to 15/1 DCM/MeOH) to provide compound **3b** (4.30 g, 77%) as a white foamed solid. $[\alpha]_D^{25}$ = −41.8 (*c* = 1.0, $CHCl_3$). **^{1}H NMR** (400 MHz, $CDCl_3$) *δ* 7.65 (dd, *J* = 15.3, 6.3 Hz, 4H), 7.37 (d, *J* = 7.0 Hz, 6H), 7.09 (t, *J* = 9.4 Hz, 2H), 6.80 (d, *J* = 7.9 Hz, 3H), 5.05 – 4.68 (m, 3H), 4.17 (s, 1H), 3.76 (s, 3H), 3.19 (d, *J* = 9.7 Hz, 1H), 3.07 (s, 3H), 2.86 (d, *J* = 25.2 Hz, 1H), 2.72 (d, *J* = 25.9 Hz, 3H), 2.25 (m, 1H), 1.29 (d, *J* = 25.0 Hz, 9H), 1.02 (d, *J* = 10.3 Hz, 12H), 0.97 (d, *J* = 6.3 Hz, 3H), 0.86 (s, 3H). **^{13}C NMR** (100 MHz, $CDCl_3$) *δ* 174.6, 170.9, 158.3, 156.0, 155.3, 136.2, 135.9, 135.7, 133.9, 132.6, 130.0, 130.0, 129.7, 129.5, 128.4, 127.8, 127.7, 127.7, 127.6, 127.5, 127.0, 114.0, 113.8, 80.7, 80.3, 70.1, 62.2, 61.6, 60.1, 55.2, 54.9, 33.3, 32.2, 31.5, 30.7, 29.7, 29.1, 28.3, 28.2, 27.4, 26.9, 21.8, 20.6, 20.4, 19.9, 19.9, 19.7, 19.3, 19.2, 19.1. **HRMS (ESI)** *m*/*z*: calcd for $C_{42}H_{60}N_3O_8Si^+$ [M+Na]$^+$: 762.4144, found: 762.4142.

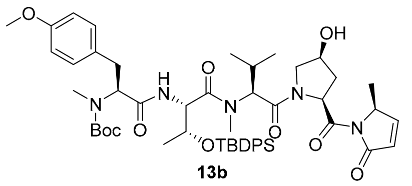

13b

Synthesis of (13b). To the solution of **4a** (568 mg, 1.34 mmol) in DCM (5 mL) was added 4 N HCl in dioxane (6.7 mL, 26.77 mmol) at 0 °C. The mixture was allowed to reach room temperature, stirred for 30 min, and concentrated under reduced pressure to provide as a white solid. To this salt was added a solution of **3b** (1.02 g, 1.34 mmol) and HATU (763 mg, 2.01 mmol) in DCM (10 mL), then DIPEA (663 μL, 4.02 mmol) was added dropwise at 0 °C. The reaction was allowed to reach room temperature and stirred overnight. The mixture was concentrated, diluted with EtOAc (30 mL) and washed with aqueous 5% $NaHSO_4$ (3 × 30 mL) and brine (50 mL), dried (Na_2SO_4), filtered, concentrated in vacuo and purified by column chromatography (15/1 to 2/1 petroleum ether/EtOAc) to provide compound **13b** (857 mg, 67%) as a white foamed solid. $[\alpha]_D^{25}$ = −60.3 (*c* = 1.0, $CHCl_3$). **^{1}H NMR** (400 MHz, $CDCl_3$) *δ* 7.62 (d, *J* = 7.0 Hz, 2H), 7.56 (d, *J* = 6.7 Hz, 2H), 7.41 (dd, *J* = 10.5, 7.1 Hz, 6H), 7.28 (d, *J* = 6.8 Hz, 1H), 7.08 (d, *J* = 10.3 Hz, 1H), 6.80 (d, *J* = 8.2 Hz, 3H), 6.10 (d, *J* = 6.1 Hz, 1H), 5.65 (d, *J* = 9.9 Hz, 1H), 5.13 (s, 1H), 4.99 – 4.71 (m, 3H), 4.24 – 4.08 (m, 2H), 3.88 (d, *J* = 11.7 Hz, 1H), 3.76 (d, *J* = 1.9 Hz, 3H), 3.65 – 3.48 (m, 2H), 3.23 (dd, *J* = 14.7, 4.9 Hz, 1H), 3.00 (s, 3H), 2.95 – 2.83 (m, 1H), 2.72 (d, *J* = 23.4 Hz, 3H), 2.25 (s, 1H), 2.18 – 2.08 (m, 1H), 1.92 (d, *J* = 14.6 Hz, 1H), 1.47 (d, *J* = 6.7 Hz, 3H), 1.28 (d, *J* = 33.1 Hz, 9H), 1.04 (d, *J* = 16.4 Hz, 15H), 0.81 (s, 3H). **^{13}C NMR** (100 MHz, $CDCl_3$) *δ* 174.2, 170.4, 170.2, 169.7, 169.2, 158.2, 154.3, 135.8, 135.7, 135.6, 135.5, 133.3, 133.1, 129.9, 129.8, 128.4, 127.9, 127.7, 127.7, 127.6, 125.1, 113.9, 113.7, 80.4, 80.0, 77.5, 77.4, 77.2, 76.8, 71.5, 69.6, 60.1, 59.0, 58.3, 58.1, 56.8, 55.1, 54.3, 54.1, 36.4, 33.0, 31.4, 30.3, 28.2, 28.1, 28.0, 27.3, 26.9, 20.9, 20.6, 19.6, 19.2, 19.1, 18.7, 18.4, 18.2, 17.0, 16.9. **HRMS (ESI)** *m/z*: calcd for $C_{52}H_{71}N_5O_{10}SiNa^+$ $[M+Na]^+$: 976.4862, found: 976.4865.

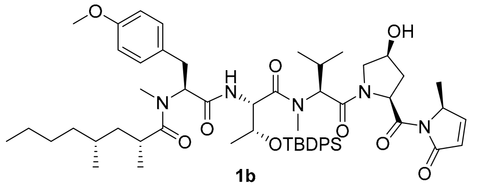

1b

Synthesis of (1b). To the solution of **13b** (857 mg, 0.90 mmol) in DCM (5 mL) was added 4 N HCl in dioxane (9.0 mL, 35.90 mmol) at 0 °C. The mixture was allowed to reach room temperature, stirred for 2 h, and concentrated under reduced pressure to provide as a white solid. To this salt was added a solution of **2a** (464 mg, 2.69 mmol) and HATU (1.02 g, 2.69 mmol) in DCM (10 mL), then DIPEA (890 μL, 5.39 mmol) was added dropwise at 0 °C. The reaction was allowed to reach room temperature and stirred overnight. The mixture was concentrated,

diluted with EtOAc (30 mL) and washed with aqueous 5% $NaHSO_4$ (3 × 30 mL) and brine (30 mL), dried (Na_2SO_4), filtered, concentrated in vacuo and purified by column chromatography (100/1 to 20/1 DCM/MeOH) to provide compound **1b** (304 mg, 34%) as a white foamed solid.$[\alpha]_D^{25}$ = −71.2 (*c* = 0.5, $CHCl_3$). **^{1}H NMR** (400 MHz, $CDCl_3$) *δ* 7.62 (d, *J* = 7.0 Hz, 2H), 7.56 (d, *J* = 7.1 Hz, 2H), 7.38 (m, 6H), 7.27 (d, *J* = 6.1 Hz, 1H), 7.08 (d, *J* = 8.1 Hz, 2H), 7.00 (d, *J* = 8.4 Hz, 1H), 6.85 – 6.72 (m, 2H), 6.08 (d, *J* = 6.1 Hz, 1H), 5.62 (dd, *J* = 18.5, 7.5 Hz, 2H), 5.13 (d, *J* = 11.0 Hz, 1H), 4.90 (dd, *J* = 8.4, 2.9 Hz, 1H), 4.79 (q, *J* = 6.9 Hz, 1H), 4.21 – 4.05 (m, 2H), 3.86 (d, *J* = 11.7 Hz, 1H), 3.73 (s, 3H), 3.61 (dd, *J* = 11.8, 4.5 Hz, 1H), 3.56 (d, *J* = 11.1 Hz, 1H), 3.14 (dd, *J* = 15.0, 5.6 Hz, 1H), 2.99 (s, 3H), 2.93 (d, *J* = 14.6 Hz, 1H), 2.83 (d, *J* = 4.9 Hz, 3H), 2.55 (dq, *J* = 10.9, 6.4 Hz, 1H), 2.24 (dq, *J* = 11.7, 6.3 Hz, 1H), 2.14 (ddd, *J* = 14.4, 10.2, 4.6 Hz, 1H), 1.92 (d, *J* = 14.5 Hz, 1H), 1.46 (d, *J* = 6.7 Hz, 3H), 1.33 – 1.17 (m, 5H), 1.10 (d, *J* = 24.3 Hz, 4H), 1.02 (s, 8H), 0.95 (q, *J* = 6.9 Hz, 8H), 0.86 (t, *J* = 6.9 Hz, 4H), 0.80 (d, *J* = 6.6 Hz, 4H), 0.49 (d, *J* = 5.2 Hz, 3H). **^{13}C NMR** (100 MHz, $CDCl_3$) *δ* 178.2, 177.5, 177.4, 173.9, 171.2, 170.4, 170.3, 170.3, 170.1, 170.0, 169.7, 169.1, 167.6, 158.1, 154.2, 154.0, 135.8, 135.7, 135.7, 135.6, 135.5, 135.4, 133.8, 133.3, 133.0, 132.7, 129.9, 129.8, 129.8, 129.7, 129.7, 129.6, 129.6, 129.5, 129.4, 129.3, 128.8, 128.7, 128.7, 128.6, 127.7, 127.7, 127.6, 127.6, 127.4, 125.2, 125.0, 113.6, 71.3, 70.2, 70.1, 69.6, 60.0, 59.0, 58.9, 58.3, 58.0, 56.7, 56.1, 55.2, 55.0, 54.9, 54.8, 54.6, 54.0, 41.4, 36.9, 36.4, 33.2, 32.4, 30.7, 30.3, 30.3, 30.0, 29.9, 28.8, 28.7, 27.2, 26.9, 26.8, 26.7, 22.9, 22.8, 22.8, 22.8, 22.5, 20.3, 20.1, 19.8, 19.3, 19.2, 19.1, 19.0, 18.7, 18.6, 18.4, 18.3, 18.1, 17.9, 17.9, 17.1, 16.9, 14.0, 14.0, 13.9. **HRMS (ESI)** *m/z*: calcd for $C_{57}H_{81}N_5O_9SiNa^+$ $[M+Na]^+$: 1030.5696, found: 1030.5690.

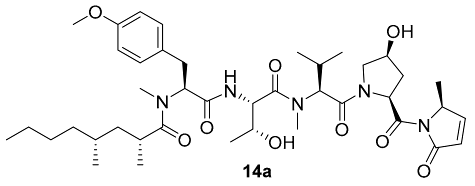

14a

Synthesis of (14a). To the solution of **1b** (100 mg, 99 μmol) in THF (0.5 mL) was added $3HF\cdot Et_3N$ (162 μL, 0.99 mmol) at 0 °C. The mixture was allowed to reach room temperature and stirred overnight. The mixture was quenched with saturated aqueous $NaHCO_3$ (20 mL). The aqueous phase was extracted with EtOAc (3 × 10 mL) and the combined organics were washed with brine (30 mL), dried (Na_2SO_4), filtered, concentrated in vacuo and purified by column chromatography (60/1 to 20/1 DCM/MeOH) to provide compound **14a** (17 mg, 22%) as a white foamed solid, recovery **1b** (76 mg, 76%). $[\alpha]_D^{25}$ = −79.2 (*c* = 0.5, $CHCl_3$). **^{1}H NMR** (400 MHz, $CDCl_3$) *δ* 7.27 (d, *J* = 9.0 Hz, 1H), 7.10 (d, *J* = 6.5 Hz, 2H), 6.92 (d, *J* = 9.5 Hz, 1H), 6.76 (d, *J* = 6.4 Hz, 2H), 6.08 (d, *J* = 6.1 Hz, 1H), 5.64 (d, *J* = 10.0 Hz, 1H), 5.48 (t, *J* = 8.4 Hz, 1H), 4.97 (d, *J* = 11.1 Hz, 1H), 4.81 (q, *J* = 7.3 Hz, 1H), 4.74 (d, *J* = 9.5 Hz, 1H), 4.39 (t, *J* = 6.5 Hz, 1H), 4.08 (q, *J* = 6.9 Hz, 1H), 3.99 (s, 1H), 3.93 (d, *J* = 11.4 Hz, 1H), 3.81 (d, *J* = 11.5 Hz, 1H), 3.75 (s, 3H), 3.62 (d, *J* =

11.2 Hz, 1H), 3.23 (dd, *J* = 15.1, 6.9 Hz, 1H), 3.16 – 3.05 (m, 1H), 2.95 (d, *J* = 2.1 Hz, 6H), 2.67 (q, *J* = 7.0, 6.5 Hz, 1H), 2.49 – 2.37 (m, 1H), 2.21 (dp, *J* = 12.9, 6.5 Hz, 1H), 2.02 (d, *J* = 14.4 Hz, 1H), 1.66 (t, *J* = 9.8 Hz, 1H), 1.46 (d, *J* = 6.7 Hz, 3H), 1.20 (d, *J* = 24.5 Hz, 5H), 1.05 (t, *J* = 6.7 Hz, 5H), 0.98 (d, *J* = 6.5 Hz, 4H), 0.91 – 0.84 (m, 6H), 0.73 (d, *J* = 6.7 Hz, 3H), 0.57 (d, *J* = 3.5 Hz, 3H). **^{13}C NMR** (100 MHz, $CDCl_3$) δ 178.3, 174.6, 172.7, 170.6, 170.0, 169.1, 158.5, 154.4, 130.0, 128.8, 125.4, 114.0, 72.0, 67.3, 59.5, 58.6, 58.3, 57.2, 56.9, 55.3, 52.3, 41.7, 37.3, 36.6, 33.7, 32.9, 31.0, 30.7, 30.5, 29.8, 29.1, 27.5, 23.2, 19.6, 19.1, 18.9, 18.5, 18.3, 17.0, 14.3. **HRMS (ESI)** *m/z*: calcd for $C_{41}H_{63}N_5O_9Na^+$ [M+Na]$^+$: 792.4518, found: 792.4517.

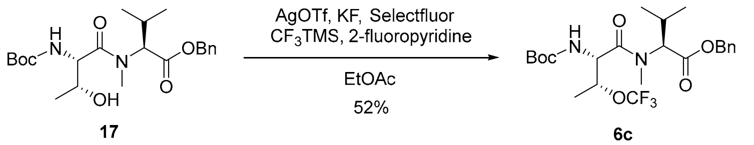

17 **6c**

Synthesis of (6c). To a reaction tube that was equipped with a stirring bar, AgOTf (9.89 g, 38.5 mmol), Selectfluor (6.82 g, 19.2 mmol), KF (2.98 g, 51.3 mmol), **17** (5.42 g, 12.8 mmol) were added successively in a nitrogen-filled glovebox. Then EtOAc (60 mL), 2-fluoropyridine (3.3 mL, 38.5 mmol) and CF_3TMS (5.7 mL, 38.5 mmol) were added successively under Ar atmosphere. The reaction mixture was stirred at room temperature and stirred overnight. The mixture was filtered through a pad of celite, washed with EtOAc, and concentrated in vacuo and purified by column chromatography (30/1 to 5/1 petroleum ether/EtOAc) to provide compound **6c** (3.26 g, 52%) as a colorless oil. $[\alpha]_D^{22}$ = −75.5 (*c* = 0.2, $CHCl_3$). **^{1}H NMR** (400 MHz, $CDCl_3$) δ 7.36 – 7.29 (m, 5H), 5.28 (d, *J* = 8.5 Hz, 1H), 5.17 (d, *J* = 12.1 Hz, 1H), 5.08 (d, *J* = 12.1 Hz, 1H), 4.92 (d, *J* = 10.5 Hz, 1H), 4.76 – 4.69 (m, 1H), 4.46 (m, 1H), 3.03 (s, 3H), 2.25 (m, 1H), 1.42 (s, 9H), 1.20 (d, *J* = 6.4 Hz, 3H), 1.01 (d, *J* = 6.6 Hz, 3H), 0.83 (d, *J* = 6.8 Hz, 3H). **^{13}C NMR** (100 MHz, $CDCl_3$) δ 170.3, 170.1, 155.5, 135.4, 128.7, 128.6, 128.3, 122.9, 120.4, 80.5, 75.6, 67.0, 62.1, 53.7, 31.8, 28.3, 28.1, 27.3, 19.9, 18.7, 17.2. **^{19}F NMR** (376 MHz, $CDCl_3$) δ -58.2, -58.5 (rotamer). **HRMS (ESI)** *m/z*: calcd for $C_{23}H_{33}N_2O_6F_3Na^+$ [M+Na]$^+$: 513.2183, found: 513.2183.

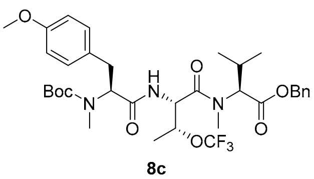

8c

Synthesis of (8c). The titled compound **8c** was obtained following the general procedure described for **8b**. The reaction was purified by column chromatography (20/1 to 3/1 petroleum ether/EtOAc) to provide compound **8c** (910 mg, 57%) as a pale yellow oil. $[\alpha]_D^{22}$ = −92.0 (*c* = 0.5, $CHCl_3$). **^{1}H NMR** (400 MHz, $CDCl_3$) δ 7.31 (d, *J* = 3.3 Hz, 5H), 7.07 (m, 2H), 6.84 – 6.57 (m, 3H), 5.16 (d, *J* = 12.1 Hz, 1H), 5.07 (d, *J* = 12.1 Hz, 1H), 5.01 (d, *J* = 8.7 Hz, 1H), 4.88 (d, *J* = 10.8 Hz, 1H), 4.78 (d, *J* = 7.9 Hz, 1H), 4.52 (t, *J* = 6.6 Hz, 1H), 3.75 (s, 3H), 3.25 (dd, *J* = 14.4, 6.4 Hz, 1H),

3.06 – 2.80 (m, 4H), 2.79 – 2.62 (m, 3H), 2.19 (s, 1H), 1.35 (d, *J* = 22.6 Hz, 9H), 1.19 (d, *J* = 6.3 Hz, 3H), 1.02 – 0.95 (m, 3H), 0.84 – 0.67 (m, 3H). ^{13}**C NMR** (100 MHz, $CDCl_3$) δ 170.5, 170.2, 169.3, 158.4, 156.2, 135.4, 130.2, 130.0, 129.5, 128.7, 128.7, 128.6, 128.4, 122.9, 120.4, 114.1, 113.9, 81.0, 80.6, 75.2, 66.9, 61.9, 55.3, 52.2, 52.1, 33.2, 32.0, 31.6, 29.8, 29.8, 29.5, 28.3, 27.3, 22.8, 19.9, 19.8, 18.7, 17.3, 14.2. ^{19}**F NMR** (376 MHz, $CDCl_3$) δ -57.8, -58.2, -58.4 (rotamer). **HRMS (ESI)** *m/z*: calcd for $C_{34}H_{46}N_3O_8F_3Na^+$ [M+Na]$^+$: 704.3129, found: 704.3129.

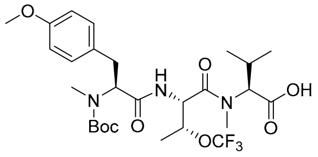

3c

Synthesis of (3c). The titled compound **3c** was obtained following the general procedure described for **3b**. The reaction was purified by column chromatography (100/1 to 20/1 DCM/MeOH) to provide compound **3c** (630 mg, 97%) as a white foamed solid. $[\alpha]_D^{22}$ = –94.2 (*c* = 0.5, $CHCl_3$). ^{1}**H NMR** (400 MHz, $CDCl_3$) δ 9.70 (s, 1H), 7.09 (dd, *J* = 15.8, 7.8 Hz, 3H), 6.78 (d, *J* = 8.0 Hz, 2H), 5.12 (d, *J* = 8.3 Hz, 1H), 4.92 – 4.69 (m, 2H), 4.59 (m, 1H), 3.76 (s, 3H), 3.25 (dd, *J* = 14.3, 6.7 Hz, 1H), 3.14 – 2.85 (m, 4H), 2.81 – 2.64 (m, 3H), 2.21 (s, 1H), 1.35 (dd, *J* = 17.8, 6.8 Hz, 12H), 1.05 (q, *J* = 7.1 Hz, 3H), 0.87 – 0.71 (m, 3H). ^{13}**C NMR** (100 MHz, $CDCl_3$) δ 173.3, 173.3, 170.9, 170.6, 169.8, 169.6, 158.3, 156.6, 156.2, 155.3, 130.2, 130.0, 129.9, 129.4, 129.2, 125.4, 122.9, 120.3, 117.8, 114.1, 113.9, 81.0, 80.8, 75.2, 75.0, 62.1, 61.2, 59.8, 55.2, 53.5, 52.4, 52.3, 33.4, 32.0, 32.0, 31.5, 31.0, 30.3, 30.2, 29.7, 29.7, 29.5, 29.4, 28.2, 28.1, 28.0, 27.5, 27.2, 22.7, 19.9, 19.7, 18.8, 18.1, 17.5, 14.2.^{19}**F NMR** (376 MHz, $CDCl_3$) δ -57.9, -58.2 (rotamer). **HRMS (ESI)** *m/z*: calcd for $C_{27}H_{40}N_3O_8F_3Na^+$ [M+Na]$^+$: 614.2660, found: 614.2665.

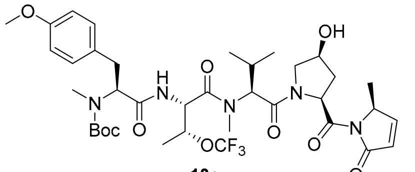

13c

Synthesis of (13c). The titled compound **13c** was obtained following the general procedure described for **13b**. The reaction was purified by column chromatography (100/1 to 20/1 DCM/MeOH) to provide compound **13c** (376 mg, 57%) as a white foamed solid. $[\alpha]_D^{22}$ = –111.4 (*c* = 0.5, $CHCl_3$). ^{1}**H NMR** (400 MHz, $CDCl_3$) δ 7.28 (d, *J* = 2.0 Hz, 1H), 7.10 (t, *J* = 9.3 Hz, 2H), 6.79 (d, *J* = 8.6 Hz, 3H), 6.08 (dd, *J* = 6.0, 1.6 Hz, 1H), 5.60 (dd, *J* = 10.1, 2.0 Hz, 1H), 5.12 – 5.00 (m, 2H), 4.85 – 4.70 (m, 2H), 4.57 (m, 1H), 4.37 (d, *J* = 10.7 Hz, 1H), 3.93 (d, *J* = 11.5 Hz, 1H), 3.82 (dd, *J* = 11.7, 4.3 Hz, 1H), 3.76 (s, 3H), 3.54 (d, *J* = 11.1 Hz, 1H), 3.26 (dd, *J* = 14.4, 6.3 Hz, 1H), 3.03 (d, *J* = 35.5 Hz, 3H), 2.90 (t, *J* = 10.3 Hz, 1H), 2.74 (d, *J* = 33.9 Hz, 3H), 2.39 (m, 1H), 2.25 (s, 1H), 2.02 (d, *J* = 14.6 Hz, 1H), 1.45 (d, *J* = 6.6 Hz, 3H), 1.37 (d, *J* = 20.8 Hz, 9H), 1.34 – 1.31 (m, 3H), 0.99

(d, *J* = 6.5 Hz, 3H), 0.81 – 0.69 (m, 3H). **^{13}C NMR** (100 MHz, $CDCl_3$) 174.5, 174.4, 170.6, 170.0, 169.0, 168.9, 158.4, 156.2, 154.3, 130.1, 129.9, 129.5, 125.4, 122.8, 120.3, 114.1, 113.9, 81.1, 80.7, 74.9, 71.8, 71.8, 61.3, 60.4, 59.4, 58.6, 58.2, 57.1, 55.3, 52.4, 52.4, 52.2, 36.7, 33.2, 32.0, 31.6, 30.6, 29.8, 29.7, 29.4, 28.3, 28.2, 27.3, 22.8, 18.9, 18.4, 17.8, 17.0, 14.2. **^{19}F NMR** (376 MHz, $CDCl_3$) *δ* -58.3, -58.4 (rotamer). **HRMS (ESI)** *m*/*z*: calcd for $C_{37}H_{52}N_5O_{10}F_3Na^+$ [M+Na]$^+$: 806.3558, found: 806.3560.

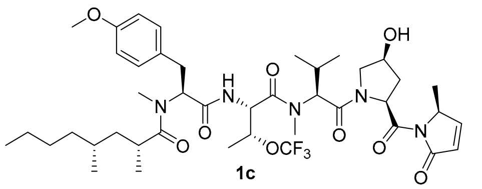

1c

Synthesis of (1c). The titled compound **1c** was obtained following the general procedure described for **1b**. The reaction was purified by column chromatography (100/1 to 20/1 DCM/MeOH) to provide compound **1c** (228 mg, 71%) as a white foamed solid. $[\alpha]_D^{22}$ = −125.6 (*c* = 0.5, $CHCl_3$). **^{1}H NMR** (400 MHz, $CDCl_3$) *δ* 7.28 (d, *J* = 2.1 Hz, 1H), 7.09 (d, *J* = 8.5 Hz, 2H), 6.96 (d, *J* = 8.9 Hz, 1H), 6.76 (d, *J* = 8.5 Hz, 2H), 6.08 (dd, *J* = 6.1, 1.7 Hz, 1H), 5.59 (dd, *J* = 10.1, 2.0 Hz, 1H), 5.48 (dd, *J* = 10.5, 6.1 Hz, 1H), 5.05 (dd, *J* = 9.8, 5.4 Hz, 2H), 4.80 (m, 1H), 4.54 (m, 1H), 4.37 (m, 1H), 3.96 – 3.81 (m, 2H), 3.75 (s, 3H), 3.53 (d, *J* = 11.1 Hz, 1H), 3.17 (dd, *J* = 14.9, 6.0 Hz, 1H), 3.01 (d, *J* = 7.9 Hz, 3H), 2.97 – 2.92 (m, 1H), 2.88 (d, *J* = 7.7 Hz, 3H), 2.63 (m, 1H), 2.39 (m, 1H), 2.29 – 2.19 (m, 1H), 2.02 (d, *J* = 14.6 Hz, 1H), 1.56 (t, *J* = 9.1 Hz, 1H), 1.45 (d, *J* = 6.7 Hz, 3H), 1.30 (d, *J* = 6.2 Hz, 3H), 1.25 (s, 4H), 1.18 – 1.11 (m, 3H), 1.03 (d, *J* = 6.6 Hz, 3H), 1.00 (d, *J* = 6.7 Hz, 3H), 0.87 (t, *J* = 7.0 Hz, 4H), 0.78 (d, *J* = 6.7 Hz, 3H), 0.54 (d, *J* = 5.1 Hz, 3H). **^{13}C NMR** (100 MHz, $CDCl_3$) *δ* 178.3, 174.4, 170.6, 170.0, 169.0, 168.8, 158.4, 154.3, 130.0, 129.9, 128.8, 125.4, 122.8, 120.3, 113.9, 113.9, 77.5, 77.4, 77.2, 76.8, 74.8, 74.8, 71.8, 59.4, 58.6, 58.2, 57.1, 56.9, 55.2, 52.2, 41.7, 37.0, 36.7, 33.6, 32.7, 30.9, 30.6, 30.3, 29.8, 29.1, 29.1, 27.3, 23.1, 23.1, 19.6, 18.9, 18.5, 18.5, 17.9, 17.7, 17.0, 14.2, 14.2. **^{19}F NMR** (376 MHz, $CDCl_3$) *δ* -58.3. **HRMS (ESI)** *m*/*z*: calcd for $C_{42}H_{62}N_5O_9F_3Na^+$ [M+Na]$^+$: 860.4392, found: 860.4389.

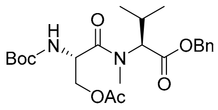

6d

Synthesis of (6d). The titled compound **6d** was obtained following the general procedure described for **17**. The reaction was purified by column chromatography (20/1 to 3/1 petroleum ether/EtOAc) to provide compound **6d** (1.73 g, 62%) as a pale yellow oil. $[\alpha]_D^{22}$ = −62.8 (*c* = 0.5, $CHCl_3$). **^{1}H NMR** (400 MHz, $CDCl_3$) *δ* 7.35 – 7.29 (m, 4H), 5.38 (d, *J* = 8.9 Hz, 1H), 5.14 (m, 2H), 4.97 – 4.84 (m, 2H), 4.18 (dd, *J* = 11.3, 4.8 Hz, 1H), 3.94 (dd, *J* = 11.2, 7.6 Hz, 1H), 3.07 (s, 3H), 2.24 (m, 1H), 1.98 (s, 3H), 1.43 (s, 9H), 1.03 – 0.94 (m, 3H), 0.84 (t, *J* = 6.7 Hz, 3H). **^{13}C NMR** (100 MHz, $CDCl_3$) *δ* 170.8, 170.4, 170.2, 155.4, 135.6, 128.7, 128.5, 128.4, 80.1, 66.8, 64.1, 62.2, 49.9, 31.5, 29.8,

28.4, 28.3, 28.3, 27.3, 20.7, 19.9, 19.0. **HRMS (ESI)** *m/z*: calcd for $C_{23}H_{34}N_2O_7Na^+$ [M+Na]+: 473.2258, found: 473.2252.

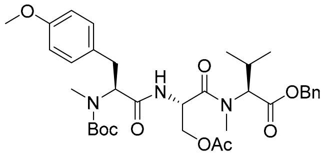

8d

Synthesis of (8d). The titled compound **8d** was obtained following the general procedure described for **8b**. The reaction was purified by column chromatography (20/1 to 3/1 petroleum ether/EtOAc) to provide compound **8d** (1.41 g, 63%) as a pale yellow oil. $[\alpha]_D^{22}$ = −98.0 (*c* = 0.5, $CHCl_3$). **^{1}H NMR** (400 MHz, $CDCl_3$) *δ* 7.36 – 7.28 (m, 5H), 7.14 – 7.00 (m, 2H), 6.78 (d, *J* = 8.5 Hz, 3H), 5.24 – 5.09 (m, 3H), 4.85 (d, *J* = 10.3 Hz, 1H), 4.73 – 4.41 (m, 1H), 4.24 (m, 1H), 3.96 – 3.88 (m, 1H), 3.75 (d, *J* = 3.0 Hz, 3H), 3.24 (dd, *J* = 13.8, 6.5 Hz, 1H), 3.01 (d, *J* = 39.3 Hz, 3H), 2.87 – 2.82 (m, 1H), 2.78 – 2.67 (m, 3H), 2.30 – 2.15 (m, 1H), 1.97 (s, 3H), 1.36 (d, *J* = 17.0 Hz, 9H), 1.02 – 0.94 (m, 3H), 0.87 – 0.75 (m, 3H). **^{13}C NMR** (100 MHz, $CDCl_3$) *δ* 170.4, 170.1, 170.0, 169.4, 169.2, 158.2, 156.2, 155.0, 135.4, 129.9, 128.5, 128.3, 128.2, 113.9, 113.7, 80.6, 80.4, 66.8, 66.6, 66.3, 65.0, 63.4, 61.9, 61.3, 61.0, 59.7, 55.1, 48.8, 48.4, 33.1, 31.9, 31.2, 30.7, 29.6, 28.9, 28.1, 27.5, 27.5, 27.4, 27.1, 21.8, 20.6, 20.5, 19.6, 19.5, 19.4, 18.9, 18.8, 18.6. **HRMS (ESI)** *m/z*: calcd for $C_{34}H_{48}N_3O_9Na^+$ [M+Na]+: 642.3385, found: 642.3381.

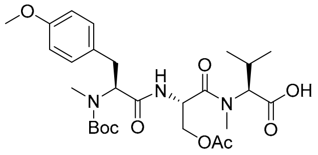

3d

Synthesis of (3d). The titled compound **3d** was obtained following the general procedure described for **3b**. The reaction was purified by column chromatography (100/1 to 20/1 DCM/MeOH) to provide compound **3d** (1.03 g, 93%) as a white foamed solid. $[\alpha]_D^{22}$ = −95.6 (*c* = 0.5, $CHCl_3$). **^{1}H NMR** (400 MHz, $CDCl_3$) *δ* 7.18 – 6.92 (m, 3H), 6.80 (s, 2H), 5.25 (s, 1H), 4.95 – 4.61 (m, 2H), 4.40 (m, 1H), 4.16 – 4.02 (m, 1H), 3.76 (s, 3H), 3.24 (dd, *J* = 14.3, 6.1 Hz, 1H), 3.15 – 2.84 (m, 4H), 2.73 (t, *J* = 14.5 Hz, 3H), 2.36 – 2.18 (m, 1H), 2.02 (s, 3H), 1.41 – 1.30 (m, 9H), 1.07 (m, 3H), 0.94 – 0.79 (m, 3H). **^{13}C NMR** (100 MHz, $CDCl_3$) *δ* 172.3, 171.3, 170.5, 170.4, 169.6, 158.1, 158.0, 158.0, 156.0, 155.1, 129.7, 129.6, 129.2, 128.9, 113.7, 113.5, 80.7, 80.4, 64.6, 63.8, 63.1, 63.0, 62.0, 61.3, 59.5, 54.9, 48.6, 48.3, 33.1, 31.5, 31.0, 30.8, 30.6, 29.4, 29.1, 27.9, 27.9, 27.3, 27.0, 26.8, 20.4, 20.3, 19.7, 19.4, 18.7, 18.6. **HRMS (ESI)** *m/z*: calcd for $C_{27}H_{42}N_3O_9^+$ [M+H]+: 552.2916, found: 552.2910.

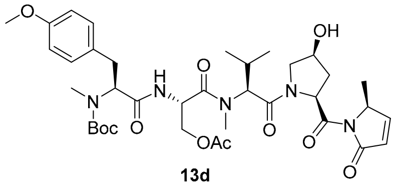

13d

Synthesis of (13d). The titled compound **13d** was obtained following the general procedure described for **13b**. The reaction was purified by column chromatography (100/1 to 20/1 DCM/MeOH) to provide compound **13d** (223 mg, 42%) as a white foamed solid. $[\boldsymbol{\alpha}]_{\boldsymbol{D}}^{\mathbf{22}}$ = −107.8 (*c* = 0.5, $CHCl_3$). ^{1}**H NMR** (400 MHz, $CDCl_3$) *δ* 7.28 (dd, *J* = 6.3, 2.2 Hz, 1H), 7.08 (d, *J* = 11.8 Hz, 2H), 6.81 (d, *J* = 7.5 Hz, 3H), 6.09 (dd, *J* = 6.1, 1.6 Hz, 1H), 5.67 (d, *J* = 10.0 Hz, 1H), 5.17 (dt, *J* = 8.3, 6.0 Hz, 1H), 4.99 (d, *J* = 11.0 Hz, 1H), 4.88 – 4.80 (m, 1H), 4.37 (td, *J* = 11.6, 4.3 Hz, 2H), 4.16 – 4.03 (m, 1H), 3.92 (d, *J* = 11.6 Hz, 1H), 3.81 (d, *J* = 4.3 Hz, 1H), 3.76 (s, 3H), 3.59 (d, *J* = 11.3 Hz, 1H), 3.26 (dd, *J* = 14.5, 6.2 Hz, 1H), 3.06 (d, *J* = 41.4 Hz, 3H), 2.88 (d, *J* = 12.3 Hz, 1H), 2.75 (d, *J* = 9.2 Hz, 3H), 2.45 (m, 1H), 2.25 (s, 1H), 2.05 (d, *J* = 2.9 Hz, 1H), 2.01 (s, 3H), 1.66 (s, 1H), 1.46 (d, *J* = 6.8 Hz, 3H), 1.37 (d, *J* = 16.9 Hz, 9H), 0.99 (d, *J* = 6.4 Hz, 3H), 0.85 – 0.74 (m, 3H). ^{13}**C NMR** (100 MHz, $CDCl_3$) *δ* 174.3, 170.4, 170.2, 169.9, 169.5, 168.9, 158.3, 156.3, 155.1, 154.3, 154.3, 130.0, 130.0, 129.9, 129.7, 129.3, 125.4, 125.3, 114.0, 113.8, 80.8, 80.6, 71.7, 63.4, 63.1, 59.9, 59.4, 58.6, 58.5, 58.2, 56.9, 55.2, 48.8, 48.4, 36.6, 33.2, 31.9, 31.0, 30.3, 29.7, 29.7, 29.6, 29.3, 28.2, 27.2, 22.7, 20.7, 18.9, 18.5, 16.9, 14.1. **HRMS (ESI)** *m/z*: calcd for $C_{37}H_{53}N_5O_{11}Na^+$ $[M+Na]^+$: 766.3634, found: 766.3632.

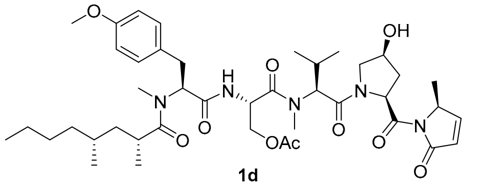

1d

Synthesis of (1d). The titled compound **1d** was obtained following the general procedure described for **1b**. The reaction was purified by column chromatography (100/1 to 20/1 DCM/MeOH) to provide compound **1d** (85 mg, 66%) as a white foamed solid. $[\boldsymbol{\alpha}]_{\boldsymbol{D}}^{\mathbf{22}}$ = −84.4 (*c* = 0.5, $CHCl_3$). ^{1}**H NMR** (400 MHz, $CDCl_3$) *δ* 7.28 (d, *J* = 2.0 Hz, 1H), 7.10 (dd, *J* = 8.8, 3.0 Hz, 2H), 6.93 (d, *J* = 8.5 Hz, 1H), 6.77 (d, *J* = 8.6 Hz, 2H), 6.09 (dd, *J* = 6.1, 1.6 Hz, 1H), 5.66 (dd, *J* = 10.1, 1.8 Hz, 1H), 5.48 (dd, *J* = 10.5, 6.2 Hz, 1H), 5.14 (m, 1H), 5.00 (d, *J* = 11.1 Hz, 1H), 4.86 – 4.76 (m, 1H), 4.42 – 4.30 (m, 2H), 4.00 (dd, *J* = 11.2, 6.6 Hz, 1H), 3.92 (d, *J* = 11.6 Hz, 1H), 3.82 (dd, *J* = 11.7, 4.3 Hz, 1H), 3.75 (s, 3H), 3.56 (d, *J* = 11.2 Hz, 1H), 3.23 – 3.12 (m, 1H), 3.04 (d, *J* = 6.4 Hz, 3H), 2.95 (d, *J* = 10.1 Hz, 1H), 2.91 (d, *J* = 5.6 Hz, 3H), 2.69 – 2.61 (m, 1H), 2.44 (m, 1H), 2.29 – 2.21 (m, 1H), 2.06 (d, *J* = 6.4 Hz, 1H), 2.00 (s, 3H), 1.63 (d, *J* = 11.0 Hz, 2H), 1.46 (d, *J* = 6.8 Hz, 3H), 1.25 (s, 5H), 1.17 (t, *J* = 6.5 Hz, 2H), 1.06 (d, *J* = 6.7 Hz, 3H), 0.99 (d, *J* = 6.4 Hz, 3H), 0.88 (t, *J* = 6.9 Hz, 4H), 0.80 (d, *J* = 6.7 Hz, 3H), 0.55 (d, *J* = 5.0 Hz, 3H). ^{13}**C NMR** (100 MHz, $CDCl_3$) *δ* 178.1, 174.3,

170.3, 170.2, 169.9, 169.3, 169.0, 158.3, 154.3, 129.9, 129.9, 129.8, 128.7, 125.3, 113.9, 71.7, 71.7, 63.4, 59.5, 58.6, 58.2, 57.0, 56.6, 55.1, 48.6, 41.5, 37.1, 36.6, 33.5, 32.6, 30.7, 30.3, 29.7, 29.0, 27.2, 23.1, 20.7, 19.5, 18.9, 18.6, 17.9, 17.0, 14.2, 14.2. **HRMS (ESI)** *m/z*: calcd for $C_{42}H_{63}N_5O_{10}Na^+$ [M+Na]$^+$: 820.4467, found: 820.4464.

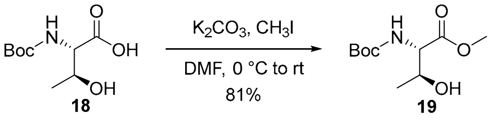

Synthesis of (19). To the solution of **18** (2.0 g, 9.12 mmol) in DMF (90 mL), then K_2CO_3 (1.89 g, 13.68 mmol) was added at 0 °C. After the mixture had been stirred for 15 min at 0 °C, CH_3I (1.7 mL, 27.4 mmol) was added dropwise at 0 °C. The reaction was allowed to reach room temperature and stirred overnight., and then the reaction mixture was extracted with EtOAc (100 mL) first, then washed with brine (5 × 50 mL), dried (Na_2SO_4), filtered, concentrated in vacuo and purified by column chromatography (20/1 to 2/1 petroleum ether/EtOAc) to provide compound **19** (1.72 g, 81%) as a colorless oil. $[\alpha]_D^{25}$ = 30.3 (*c* = 1.0, $CHCl_3$). ^{1}**H NMR** (400 MHz, $CDCl_3$) δ 5.48 (d, *J* = 7.4 Hz, 1H), 4.44 – 4.31 (m, 1H), 4.20 – 4.05 (m, 1H), 3.76 (s, 3H), 2.91 (s, 1H), 1.43 (s, 9H), 1.17 (d, *J* = 6.5 Hz, 3H). ^{13}**C NMR** (100 MHz, $CDCl_3$) δ 171.1, 156.2, 80.5, 69.0, 59.1, 52.5, 28.3, 18.8. **HRMS (ESI)** *m/z*: calcd for $C_{10}H_{19}NO_5Na^+$ [M+Na]$^+$: 256.1155, found: 256.1153.

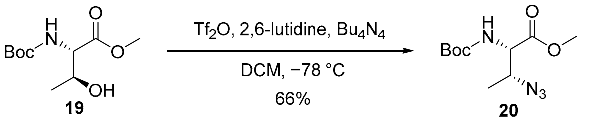

Synthesis of (20). To the solution of compound **19** (920 mg, 3.94 mmol) in DCM (40 mL) was added Tf_2O (835 μL, 4.73 mmol) and 2,6-lutidine (551 μL,4.73 mmol) dropwise at −78 °C under argon. After the mixture had been stirred for 1 h at −78 °C, Bu_4N_4 (2.81g, 9.86 mmol) was added dropwise at −78 °C. After the mixture had been stirred for 3 h at −78 °C, the reaction was quenched with saturated aqueous $NaHCO_3$ (40 mL). The aqueous phase was extracted with DCM (3 × 30 mL) and the combined organics were washed with brine (30 mL), dried (Na_2SO_4), filtered, concentrated in vacuo and purified by column chromatography (50/1 to 4/1 petroleum ether/EtOAc) to provide compound **20** (610 mg, 66%) as a colorless oil. $[\alpha]_D^{25}$ = 7.7 (*c* = 1.0, $CHCl_3$). ^{1}**H NMR** (400 MHz, $CDCl_3$) δ 5.15 (d, *J* = 9.5 Hz, 1H), 4.34 (dd, *J* = 2.7 Hz, 9.5 Hz, 1H), 4.13 (qd, *J* = 6.6, 2.5 Hz, 1H), 3.76 (s, 3H), 1.43 (s, 9H), 1.32 (d, *J* = 6.8 Hz, 3H). ^{13}**C NMR** (100 MHz, $CDCl_3$) δ 170.6, 155.8, 80.3, 77.4, 77.1, 76.7, 58.7, 57.1, 52.7, 28.2, 16.0. **HRMS (ESI)** *m/z*: calcd for $C_{10}H_{18}N_4O_4Na^+$ [M+Na]$^+$: 281.1220, found: 281.1218.

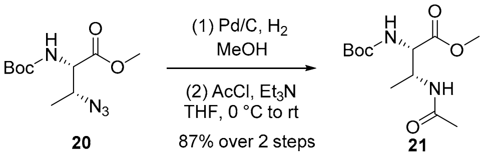

Synthesis of (21). To the solution of **20** (585 mg, 2.27 mmol) in MeOH (25 mL) was added Pd/C 10% Wt (60 mg) at 0 °C. The reaction vessel was evacuated/backfilled with argon three times first, then the reaction vessel was evacuated/backfilled with H_2 three times and stirred under this atmosphere overnight. The mixture was filtered through a pad of celite, washed with MeOH, and concentrated in vacuo.

To the solution of crude in THF (20 mL) was added Et_3N (944 μL, 6.79 mmol) and AcCl (240 μL, 3.40 mmol) dropwise at 0 °C under argon. The reaction was allowed to reach room temperature and stirred 4 h. The mixture was quenched with saturated aqueous NH_4Cl (20 mL). The aqueous phase was extracted with EtOAc (3 × 15 mL) and the combined organics were washed with brine (25 mL), dried (Na_2SO_4), filtered, concentrated in vacuo and purified by column chromatography (50/1 to 5/1 petroleum ether/EtOAc) to provide compound **21** (539 mg, 87%) as a colorless oil. $[\alpha]_D^{25}$ = 72.2 (c = 1.0, $CHCl_3$). **^{1}H NMR** (400 MHz, Acetone-*d*6) *δ* 7.17 (d, *J* = 8.4 Hz, 1H, major rotamer), 6.20 (d, *J* = 8.4 Hz, 1H, minor rotamer),4.57 – 4.30 (m, 1H), 4.21 (q, *J* = 4.4, 3.6 Hz, 1H), 3.65 (s, 3H), 2.96 (s, 1H), 1.84 (s, 3H), 1.40 (s, 9H), 1.17 (d, *J* = 6.5 Hz, 3H). **^{13}C NMR** (100MHz, Acetone-*d*6) *δ* 172.0, 170.1, 170.0, 156.5, 79.6, 59.0, 58.9, 52.3, 47.1, 47.0, 28.5, 22.9, 22.8, 18.0, 17.9. **HRMS (ESI)** m/z: calcd for $C_{12}H_{22}N_2O_5Na^+$ $[M+Na]^+$: 297.1421, found: 297.1418.

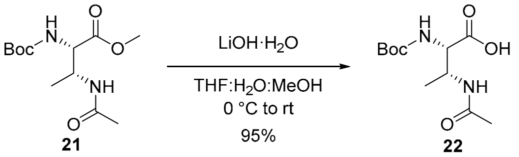

Synthesis of (22). To the solution of **21** (330 mg, 1.20 mmol) in THF (9 mL), MeOH (3 mL) and H_2O (3 mL) was added LiOH (101 mg, 2.41 mmol) at dropwise 0 °C. After addition, the reaction was allowed to reach room temperature and stirred 5 h. The mixture was concentrated, diluted with EtOAc (10 mL), the aqueous phase was made acidic with 1 N HCl (pH = 2) and extracted with EtOAc (3 × 10 mL) and the combined organics were washed with brine (25 mL), dried (Na_2SO_4), filtered, concentrated in vacuo and purified by column chromatography (10/1 to 1/1 petroleum ether/EtOAc) to provide compound **22** (297 mg, 95%) as a white solid. $[\alpha]_D^{25}$ = 80.6 (*c* = 1.0, $CHCl_3$). **^{1}H NMR** (400 MHz, Acetone-*d*6) *δ* 4.54 – 4.41 (m, 1H), 4.49 (dt, *J* = 3.7 Hz, 7.0 Hz, 1H), 4.23 (d, *J* = 4.6 Hz, 1H), 1.87 (s, 3H), 1.41 (s, 9H), 1.19 (d, *J* = 6.2 Hz, 3H). **^{13}C NMR** (100 MHz, Acetone-*d*6) *δ* 172.4, 170.5, 156.6, 79.5, 58.7, 47.1, 28.5, 22.8, 18.1. **HRMS (ESI)** *m*/*z*: calcd for $C_{11}H_{20}N_2O_5Na^+$ $[M+Na]^+$: 283.1264, found: 283.1263.

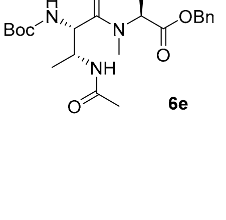

Synthesis of (6e). The titled compound **6e** was obtained following the general procedure described for **17**. The reaction was purified by column chromatography (50/1 to 2/1 petroleum ether/EtOAc) to provide compound **6e** (575 mg, 87%) as a white solid. $[\alpha]_D^{25}$ = – 60.2 (*c* = 1.0, $CHCl_3$). **^{1}H NMR** (400 MHz, Acetone-*d6*) *δ* 7.42 – 7.32 (m, 5H), 7.03 (d, *J* = 8.8 Hz, 1H), 6.05 (d, *J* = 8.2 Hz, 1H), 5.22 – 5.13 (m, 2H), 4.92 (d, *J* = 10.7 Hz,1H), 4.51 – 4.43 (m, 1H), 4.33 – 4.24 (m, 1H), 3.09 (s, 3H), 2.29 – 2.16 (m, 1H), 1.82 (s, 3H), 1.37 (s, 9H), 1.01 (d, *J* = 6.7 Hz, 3H), 0.97 (d, *J* = 6.5 Hz, 3H), 0.80 (d, *J* = 6.7 Hz, 3H). **^{13}C NMR** (100 MHz, Acetone-*d6*) *δ* 171.6, 170.1, 169.6, 155.7, 136.2, 128.5, 128.4, 128.1, 78.5, 66.2, 61.5, 55.6, 55.5, 46.2, 46.2, 30.7, 27.6, 27.0, 22.1, 22.1, 19.3, 18.0, 16.7. **HRMS (ESI)** *m*/*z*: calcd for $C_{24}H_{38}N_3O_6^+$ $[M+H]^+$: 464.2755, found: 464.2754.

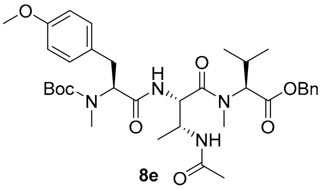

8e

Synthesis of (8e). The titled compound **8e** was obtained following the general procedure described for **8b**. The reaction was purified by column chromatography (100/1 to 20/1 DCM/MeOH) to provide compound **8e** (423 mg, 56%) as a white solid. $[\alpha]_D^{25}$ = – 105.5 (*c* = 1.0, $CHCl_3$). **^{1}H NMR** (400 MHz, Acetone-*d6*) *δ* 7.48 (dd, *J* = 6.2 Hz, 39.1 Hz, 1H), 7.42 – 7.30 (m, 5H), 7.13 (d, *J* = 8.4 Hz, 2H), 7.03 (dd, *J* = 8.6 Hz, 17.3 Hz, 1H), 6.81 (t, *J* = 8.7 Hz, 2H), 5.21 (d, *J* = 11.8 Hz, 1H), 5.12 (d, *J* = 12.2 Hz, 1H), 5.01 – 4.93 (m, 1H), 4.89 (d, *J* = 12.8 Hz, 1H), 4.67 (dd, *J* = 5.4 Hz, 10.8 Hz, 1H), 4.64 – 4.40 (m, 1H), 4.31 (d, *J* = 9.5 Hz, 1H), 3.74 (s, 3H), 3.12 (s, 3H, rotamer), 2.86 – 2.77 (m, 1H), 2.69 (s, 3H), 2.30 – 2.15 (m, 1H), 1.87 (d, *J* = 6.9 Hz, 3H), 1.35 (s, 9H, minor rotamer), 1.30 (s, 9H, major rotamer), 0.99 (d, *J* = 6.8 Hz, 3H), 0.97 (s, 3H), 0.79 (t, *J* = 7.5 Hz, 3H). **^{13}C NMR** (100 MHz, Acetone-*d6*) *δ* 171.9, 171.8, 171.2, 171.0, 170.9, 159.1, 156.3, 155.2, 136.8, 130.7, 130.6, 129.3, 129.1, 128.9, 114.3, 114.2, 79.8, 66.9, 62.1, 59.8, 56.4, 55.2, 47.2, 47.1, 34.2, 34.0, 31.5, 30.8, 30.7, 28.3, 28.2, 27.7, 22.9, 22.8, 20.0, 18.9, 17.1, 16.9. **HRMS (ESI)** *m*/*z*: calcd for $C_{35}H_{51}N_4O_8^+$ $[M+H]^+$: 655.3701, found: 655.3703.

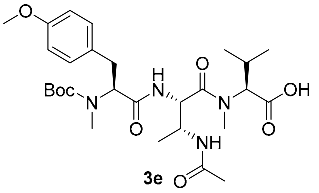

3e

Synthesis of (3e). The titled compound **3e** was obtained following the general procedure described for **3b**. The reaction was purified by column chromatography (100/1 to 20/1 DCM/MeOH) to provide compound **3e** (262 mg, 89%) as a white foamed solid. $[\alpha]_D^{25}$ = – 49.3 (*c* = 1.0, $CHCl_3$). **^{1}H NMR** (400 MHz, $CDCl_3$) *δ* 7.14 (s, 1H), 7.08 (d, *J* = 8.3 Hz, 2H), 6.78 (d, *J* = 8.2 Hz, 2H), 6.44 (s, 1H), 4.95 (dd, *J* = 3.8 Hz, 8.5 Hz, 1H), 4.72 (d, *J* = 10.1 Hz, 1H), 4.62 (s, 1H), 4.51

– 4.37 (m, 1H), 3.75 (s, 3H), 3.19 (dd, *J* = 6.3 Hz, 14.5 Hz, 1H), 3.06 (s, 1H), 3.02 (s, 3H), 2.76 (s, 3H), 2.30 – 2.10 (m, 1H), 1.91 (s, 3H), 1.40 (s, 9H, major rotamer), 1.34 (s, 9H, minor rotamer), 1.15 (d, *J* = 6.7 Hz, 3H), 1.03 (d, *J* = 6.5 Hz, 3H), 0.77 (d, *J* = 6.7 Hz, 3H). **^{13}C NMR** (100 MHz, $CDCl_3$) *δ* 171.4, 171.2, 170.9, 170.3, 158.3, 156.5, 129.8, 129.0, 113.9, 80.5, 62.4, 61.0, 55.2, 52.1, 47.0, 33.4, 29.7, 29.6, 29.3, 28.3, 28.2, 27.0, 22.9, 22.7, 20.1, 18.7, 14.1. **HRMS (ESI)** *m/z*: calcd for $C_{28}H_{45}N_4O_8^+$ [M+H]$^+$: 565.3235, found: 565.3232.

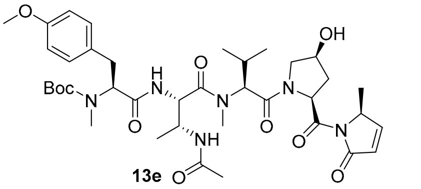

13e

Synthesis of (13e). The titled compound **13e** was obtained following the general procedure described for **13b**. The reaction was purified by column chromatography (100/1 to 20/1 DCM/MeOH) to provide compound **13e** (181 mg, 51%) as a white foamed solid. $[\alpha]_D^{25}$ = – 89.0 (*c* = 1.0, $CHCl_3$). **^{1}H NMR** (400 MHz, $CDCl_3$) *δ* 7.09 (d, *J* = 8.3 Hz, 2H), 6.87 (d, *J* = 8.9 Hz, 1H), 6.79 (d, *J* = 8.2 Hz, 2H), 6.08 (d, *J* = 6.1 Hz, 1H), 6.04 (d, *J* = 11.2 Hz, 1H), 5.64 (dd, *J* = 2.8 Hz, 9.9 Hz, 1H), 4.95 (d, *J* = 10.8 Hz, 1H), 4.85 (d, *J* = 8.9 Hz, 1H), 4.80 (d, *J* = 6.9 Hz, 1H), 4.62 (d, *J* = 46.3 Hz, 1H), 4.35 (s, 2H), 3.76 (s, 3H), 3.73 (s, 1H), 3.58 (s, 1H), 3.21 (dd, *J* = 6.8 Hz, 14.2 Hz, 1H), 3.15 – 3.04 (m, 1H), 3.00 (s, 3H), 2.78 (s, 3H), 2.54 (d, *J* = 11.4 Hz, 1H), 2.22 (d, *J* = 13.4 Hz, 1H), 2.04 (s, 1H), 1.96 (d, *J* = 12.9 Hz, 1H), 1.90 (s, 3H), 1.46 (s, 9H minor rotamor), 1.44 (s, 9H minor rotamor), 1.43 (s, 9H major rotamor), 1.34 (d, *J* = 4.2 Hz, 3H), 1.12 (d, *J* = 6.8 Hz, 3H), 0.95 (d, *J* = 6.3 Hz, 3H), 0.70 (d, *J* = 6.6 Hz, 3H). **^{13}C NMR** (100 MHz, $CDCl_3$) *δ* 174.1, 174.0, 170.6, 170.0, 169.8, 169.5, 168.4, 158.3, 156.4, 154.1, 129.8, 128.9, 125.3, 113.9, 80.6, 71.5, 60.8, 59.4, 58.5, 58.1, 56.2, 55.1, 52.3, 46.9, 36.3, 33.4, 31.8, 31.6, 30.3, 28.3, 27.0, 23.2, 19.1, 19.0, 18.2, 16.9, 14.1. **HRMS (ESI)** *m/z*: calcd for $C_{38}H_{56}N_6O_{10}Na^+$ [M+Na]$^+$: 779.3950, found: 779.3947.

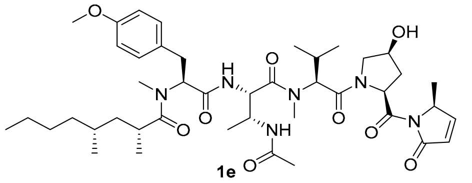

1e

Synthesis of (1e). The titled compound **1e** was obtained following the general procedure described for **1b**. The reaction was purified by column chromatography (100/1 to 20/1 DCM/MeOH) to provide compound **1e** (61 mg, 43%) as a white foamed solid. $[\alpha]_D^{25}$ = – 108.7 (*c* = 1.0, $CHCl_3$). **^{1}H NMR** (400 MHz, $CDCl_3$) *δ* 7.24 (d, *J* = 2.0 Hz, 1H), 7.09 (d, *J* = 8.7 Hz, 2H), 7.02 (s, 1H), 6.77 (d, *J* = 8.6 Hz, 2H), 6.08 (d, *J* = 1.6 Hz, 1H), 6.07 (d, *J* = 1.6 Hz, 1H), 5.65 (dd, *J* = 2.9 Hz, 9.7 Hz, 1H), 5.23 (dd, *J* = 6.3 Hz, 10.3 Hz, 1H), 4.97 (d, *J* = 10.9 Hz, 1H), 4.87 (dd, *J* = 3.2 Hz, 9.0 Hz, 1H), 4.82 – 4.76 (m, 1H), 4.36 (d, *J* = 2.9 Hz, 1H), 4.32 (d, *J* = 2.9 Hz, 1H), 3.75 (s, 3H),

3.74 – 3.73 (m, 1H), 3.15 (dd, *J* = 6.2 Hz, 14.7 Hz, 1H), 3.04 (s, 1H), 3.02 (s, 3H), 2.97 (s, 3H), 2.74 – 2.64 (m, 1H), 2.62 – 2.51 (m, 1H), 2.29 – 2.22 (m, 1H), 2.06 (d, *J* = 13.9 Hz, 1H), 1.97 (d, *J* = 3.0 Hz, 1H), 1.92 (s, 3H), 1.64 (t, *J* = 9.0 Hz, 1H), 1.45 (d, *J* = 6.8 Hz, 3H), 1.23 – 1.16 (m, 3H), 1.16 – 1.11 (m, 2H), 1.07 (s, 2H), 1.04 (d, *J* = 5.0 Hz, 3H), 1.02 (s, 2H), 0.97 (s, 3H), 0.95 (s, 3H), 0.89 (d, *J* = 6.7 Hz, 2H), 0.76 (d, *J* = 6.6 Hz, 3H), 0.56 (d, *J* = 5.6 Hz, 3H). **^{13}C NMR** (100 MHz, $CDCl_3$) *δ* 178.7, 174.1, 170.0, 169.8, 169.7, 169.5, 168.3, 158.3, 154.0, 129.5, 128.4, 125.3, 113.9, 71.5, 59.4, 58.6, 58.0, 57.8, 56.2, 55.0, 52.2, 46.7, 41.7, 37.0, 36.3, 33.7, 32.7, 31.1, 30.3, 30.3, 29.6, 28.9, 26.9, 23.2, 22.9, 19.4, 19.2, 19.2, 19.0, 18.3, 18.0, 16.9, 14.1. **HRMS (ESI)** *m*/*z*: calcd for $C_{43}H_{66}N_6O_9Na^+$ $[M+Na]^+$: 833.4783, found: 833.4781.

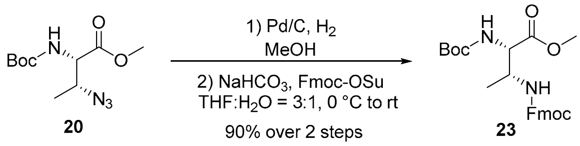

Synthesis of (23). To the solution of **20** (1.00 g, 3.87 mmol) in MeOH (30 mL) was added Pd/C 10% Wt (100 mg) at 0 °C. The reaction vessel was evacuated/backfilled with argon three times first, then the reaction vessel was evacuated/backfilled with H_2 three times and stirred under this atmosphere overnight. The mixture was filtered through a pad of celite, washed with MeOH, and concentrated in vacuo.

To the solution of crude in THF (27 mL) and H_2O (9 mL) was added $NaHCO_3$ (650 mg, 7.74 mmol) and Fmoc-OSu (1.70 g, 5.03 mmol) dropwise at 0 °C. The reaction was allowed to reach room temperature and stirred 5 h. The mixture was quenched with saturated aqueous NH_4Cl (50 mL). The aqueous phase was extracted with EtOAc (3 × 25 mL) and the combined organics were washed with brine (50 mL), dried (Na_2SO_4), filtered, concentrated in vacuo and purified by column chromatography (50/1 to 2/1 petroleum ether/EtOAc) to provide compound **23** (1.60 g, 90%) as a white solid. $[\alpha]_D^{25}$ = 37.9 (*c* = 1.0, $CHCl_3$). **^{1}H NMR** (400 MHz, Acetone-*d*6) *δ* 7.86 (d, *J* = 7.6 Hz, 2H), 7.67 (t, *J* = 7.2 Hz, 2H), 7.41 (t, *J* = 7.4 Hz, 2H), 7.32 (td, *J* = 7.6, 3.0 Hz, 2H), 6.59 (d, *J* = 8.9 Hz, 1H), 6.26 – 6.15 (m, 1H), 4.36 (dd, *J* = 6.2 Hz, 9.6 Hz, 1H), 4.34 – 4.29 (m, 1H), 4.27 (s, 1H), 4.24 (d, *J* = 10.1 Hz, 2H), 3.66 (s, 3H), 1.41 (s, 9H), 1.24 (d, *J* = 6.7 Hz, 3H). **^{13}C NMR** (100 MHz, Acetone-*d*6) *δ* 172.1, 156.7, 145.2, 145.0, 142.1, 128.6, 128.0, 128.0, 126.2, 126.1, 120.9, 79.7, 67.1, 59.0, 52.5, 49.4, 48.0, 28.5, 18.3. **HRMS (ESI)** *m*/*z*: calcd for $C_{25}H_{30}N_2O_6Na^+$ $[M+Na]^+$: 477.1996, found: 477.1997.

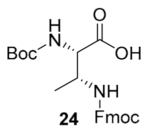

24

Synthesis of (24). The titled compound **24** was obtained following the general procedure described for **22**. The reaction was purified by column chromatography (20/1 to 1/1 petroleum

ether/EtOAc) to provide compound **24** (756 mg, 78%) as a white solid. $[\alpha]_D^{25}$ = 6.9 (*c* = 1.0, $CHCl_3$). **^{1}H NMR** (400 MHz, DMSO-*d6*) *δ* 7.88 (d, *J* = 7.5 Hz, 2H), 7.66 (t, *J* = 6.8 Hz, 2H), 7.41 (t, *J* = 7.4 Hz, 2H), 7.33 (td, *J* = 7.4, 2.7 Hz, 2H), 7.21 (d, *J* = 9.3 Hz, 1H), 6.81 (d, *J* = 9.0 Hz, 1H), 4.27 (d, *J* = 6.9 Hz, 2H), 4.20 (t, *J* = 6.9 Hz, 1H), 4.18 – 4.09 (m, 1H), 4.03 (dd, *J* = 3.9 Hz, 9.2 Hz, 1H), 1.39 (s, 9H), 1.04 (d, *J* = 6.7 Hz, 3H). **^{13}C NMR** (100 MHz, DMSO-*d6*) *δ* 172.1, 155.7, 155.3, 144.0, 143.7, 140.7, 127.6, 127.1, 127.1, 125.2, 125.1, 120.1, 78.3, 65.5, 57.6, 47.5, 46.7, 28.2, 18.0. **HRMS (ESI)** *m*/*z*: calcd for $C_{24}H_{29}N_2O_6^+$ [M+H]$^+$: 441.2020, found: 441.2013.

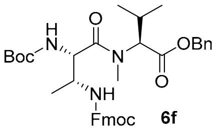
6f

Synthesis of (6f). The titled compound **6f** was obtained following the general procedure described for **17**. The reaction was purified by column chromatography (30/1 to 1/1 petroleum ether/EtOAc) to provide compound **6f** (22.2 g, 76%) as a white solid. $[\alpha]_D^{25}$ = −61.7 (*c* = 1.0, $CHCl_3$). **^{1}H NMR** (400 MHz, Acetone-*d6*) *δ* 7.85 (d, *J* = 7.5 Hz, 2H), 7.67 (dd, *J* = 7.4 Hz, 10.2 Hz, 2H), 7.41 (d, *J* = 7.4 Hz, 2H), 7.39 (s, 1H), 7.36 (s, 2H), 7.33 (d, *J* = 7.5 Hz, 2H), 7.32 – 7.28 (m, 2H), 6.48 (d, *J* = 9.0 Hz, 1H, minor rotamer), 6.03 (d, *J* = 8.7 Hz, 1H, major rotamer), 5.15 (s, 2H), 4.94 (d, *J* = 10.6 Hz, 1H), 4.60 (dd, *J* = 2.8 Hz, 8.3 Hz, 1H), 4.39 – 4.29 (m, 1H), 4.24 (d, *J* = 8.3 Hz, 2H), 4.04 (p, *J* = 7.0 Hz, 1H), 3.10 (s, 3H), 2.89 (s, 1H), 2.32 – 2.17 (m, H), 1.33 (s, 9H), 1.09 (d, *J* = 6.8 Hz, 3H), 0.97 (d, *J* = 6.5 Hz, 3H), 0.81 (d, *J* = 6.7 Hz, 3H). **^{13}C NMR** (100 MHz, Acetone-*d6*) *δ* 172.6, 171.0, 157.2, 156.6, 156.6, 145.3, 144.9, 142.1, 142.1, 137.0, 129.3, 129.3, 129.0, 128.9, 128.5, 128.5, 127.9, 127.9, 126.2, 126.1, 120.8, 79.5, 67.1, 67.1, 62.4, 55.9, 55.9, 49.3, 48.0, 48.0, 31.7, 28.5, 27.9, 20.2, 18.9, 17.8, 17.8. **HRMS (ESI)** *m*/*z*: calcd for $C_{37}H_{46}N_3O_7^+$ [M+H]$^+$: 644.3330, found: 644.3329.

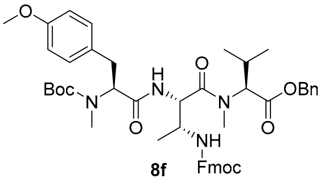
8f

Synthesis of (8f). The titled compound **8f** was obtained following the general procedure described for **8b**. The reaction was purified by column chromatography (20/1 to 1/1 petroleum ether/EtOAc) to provide compound **8f** (21.3 g, 82%) as a white solid. $[\alpha]_D^{25}$ = −80.5 (*c* = 1.0, $CHCl_3$). **^{1}H NMR** (400 MHz, Acetone-*d6*) *δ* 7.86 (d, *J* = 7.6 Hz, 2H), 7.71 (dd, *J* = 7.2 Hz, 17.9 Hz, 2H), 7.42 (d, *J* = 7.5 Hz, 2H), 7.39 (s, 2H), 7.36 (s, 2H), 7.34 (s, 2H), 7.32 (s, 1H), 7.31 (s, 1H), 7.09 (d, *J* = 13.3 Hz, 2H), 6.79 (dt, *J* = 7.9 Hz, 15.9 Hz, 2H), 6.62 – 6.22 (m, 1H), 5.20 (dd, *J* = 5.0 Hz, 16.7 Hz, 1H), 5.13 (d, *J* = 12.2 Hz, 1H), 5.04 – 4.93 (m, 1H), 4.86 (d, *J* = 41.0 Hz, 1H), 4.71 (d, *J* = 9.5 Hz, 1H), 4.54 – 4.39 (m, 1H), 4.32 – 4.22 (m, 1H), 4.12 – 4.00 (m, 1H), 3.73 (s, 3H), 3.12 (s, 1H, major rotamer), 3.08 (s, 1H, minor rotamer), 2.99 – 2.87 (m, 1H), 2.84 – 2.75 (m, 1H), 2.67 (s, 1H,

major rotamer), 2.56 (s, 1H, minor rotamer), 2.30 – 2.14 (m, 1H), 1.29 (s, 1H, major rotamer), 1.25 (s, 1H, minor rotamer), 1.07 (d, *J* = 6.5 Hz, 3H), 0.99 (d, *J* = 6.4 Hz, 3H), 0.81 (d, *J* = 6.0 Hz, 3H). **^{13}C NMR** (100 MHz, Acetone-*d6*) *δ* 172.1, 171.9, 171.3, 171.2, 171.0, 159.2, 157.9, 157.4, 156.5, 155.4, 145.4, 145.1, 144.9, 142.1, 136.9, 130.9, 130.7, 130.6, 129.4, 129.3, 129.3, 129.1, 128.9, 128.5, 127.9, 126.1, 126.0, 120.8, 114.5, 114.4, 80.0, 67.4, 67.1, 62.3, 61.9, 60.1, 56.3, 55.4, 55.1, 49.5, 47.9, 34.1, 34.0, 31.7, 30.9, 28.4, 27.9, 20.2, 19.0, 17.7, 17.3. **HRMS (ESI)** *m/z*: calcd for $C_{48}H_{59}N_4O_9^+$ [M+H]$^+$: 835.4277, found: 835.4276.

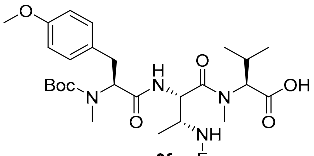

3f

Synthesis of (3f). The titled compound **3f** was obtained following the general procedure described for **3b**. The reaction was purified by column chromatography (100/1 to 25/1 DCM/MeOH) to provide compound **3f** (407 mg, 87%) as a white solid. $[\alpha]_D^{25}$ = −67.4 (*c* = 1.0, $CHCl_3$). **^{1}H NMR** (400 MHz, $CDCl_3$) *δ* 7.79 – 7.70 (m, 2H), 7.58 (d, *J* = 9.2 Hz, 2H), 7.38 (t, *J* = 5.2 Hz, 2H), 7.33 – 7.27 (m, 2H), 7.14 – 7.06 (m, 2H), 7.02 (d, *J* = 29.2 Hz, 1H), 6.84 – 6.75 (m, 2H), 6.73 (s, 1H, minor rotamer), 5.29 (d, *J* = 81.6 Hz, 1H, major rotamer), 4.92 (d, *J* = 60.8 Hz, 1H), 4.75 (d, *J* = 9.8 Hz, 1H), 4.56 (s, 1H), 4.39 (s, 1H), 4.30 (s, 1H), 4.18 (s, 2H), 3.76 (s, 3H), 3.24 (d, *J* = 15.6 Hz, 1H), 3.02 (s, 3H), 2.70 (s, 3H), 2.30 – 2.12 (m, 1H), 2.04 (d, *J* = 3.5 Hz, 1H), 1.41 (s, 9H, major rotamer), 1.34 (s, 9H, minor rotamer), 1.29 (s, 9H, minor rotamer), 1.20 (d, *J* = 18.0 Hz, 3H), 1.05 (d, *J* = 9.4 Hz, 3H), 0.78 (s, 3H). **^{13}C NMR** (100 MHz, $CDCl_3$) *δ* 176.3, 171.9, 171.4, 158.9, 157.0, 144.5, 144.1, 141.9, 141.8, 130.5, 129.9, 128.3, 127.7, 125.7, 120.6, 114.5, 81.3, 68.1, 63.4, 62.3, 55.8, 52.9, 49.9, 47.6, 33.9, 33.4, 32.3, 28.9, 27.6, 21.2, 20.7, 19.2. **HRMS (ESI)** *m/z*: calcd for $C_{41}H_{51}N_4O_9^-$ [M−H]$^-$: 743.3662, found: 743.3660.

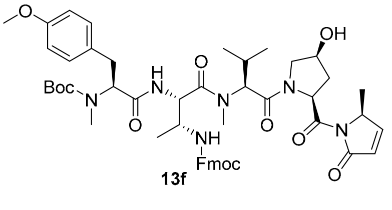

13f

Synthesis of (13f). The titled compound **13f** was obtained following the general procedure described for **13b**. The reaction was purified by column chromatography (100/1 to 20/1 DCM/MeOH) to provide compound **13f** (127 mg, 47%) as a white solid. $[\alpha]_D^{25}$ = −86.1 (*c* = 1.0, $CHCl_3$). **^{1}H NMR** (400 MHz, Acetone-*d6*) *δ* 7.86 (d, *J* = 7.5 Hz, 2H), 7.72 (s, 2H), 7.59 – 7.48 (m, 1H), 7.41 (t, *J* = 7.5 Hz, 2H), 7.34 (d, *J* = 7.5 Hz, 2H), 7.31 (s, 1H), 7.11 (d, *J* = 8.1 Hz, 2H), 6.82 (d, *J* = 10.5 Hz, 2H), 6.11 (dd, *J* = 1.6 Hz, 6.1 Hz, 1H), 5.43 (t, *J* = 7.6 Hz, 1H), 5.05 (d, *J* = 10.9 Hz, 1H), 4.88 (s, 1H), 4.84 – 4.80 (m, 1H), 4.80 – 4.74 (m, 1H), 4.50 – 4.39 (m, 1H), 4.33 (d, *J* = 6.6 Hz, 2H),

4.23 (s, 1H), 4.12 (s, 2H), 3.74 (s, 3H), 3.50 (t, *J* = 6.5 Hz, 1H), 3.32 (d, *J* = 15.5 Hz, 1H), 3.14 (s, 3H, major rotamer), 3.09 (s, 3H, minor rotamer),, 2.85 (s, 3H), 2.80 (dd, *J* = 10.5 Hz, 14.4 Hz, 1H), 2.69 (s, 1H), 2.59 (d, *J* = 18.5 Hz, 2H), 2.27 – 2.15 (m, 1H), 1.79 – 1.70 (m, 1H), 1.41 (d, *J* = 6.7 Hz, 3H), 1.29 (s, 9H), 1.19 (d, *J* = 6.8 Hz, 3H), 0.97 (d, *J* = 6.4 Hz, 3H), 0.77 (s, 3H). **^{13}C NMR** (100 MHz, Acetone-*d*6) *δ* 173.0, 172.1, 171.8, 171.2, 170.7, 169.0, 159.2, 156.0, 155.5, 145.0, 142.1, 130.8, 128.5, 127.9, 126.2, 125.6, 120.8, 114.5, 80.1, 70.6, 67.3, 61.9, 60.4, 59.8, 59.0, 59.0, 55.8, 55.4, 54.8, 49.4, 47.9, 37.3, 34.2, 31.2, 30.8, 28.4, 28.0, 19.4, 18.7, 18.2, 17.8, 17.5. **HRMS (ESI)** *m*/*z*: calcd for $C_{41}H_{63}N_5O_8Na^+$ [M+Na]$^+$: 959.4525, found: 959.4521.

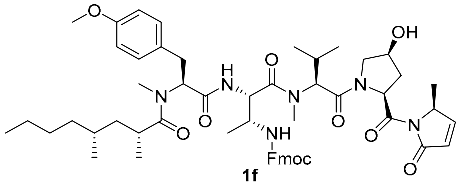

1f

Synthesis of (1f). The titled compound **1f** was obtained following the general procedure described for **1b**. The reaction was purified by column chromatography (100/1 to 20/1 DCM/MeOH) to provide compound **1f** (127 mg, 40%) as a white solid. $[\alpha]_D^{25}$ = −122.3 (*c* = 1.0, $CHCl_3$). **^{1}H NMR** (400 MHz, Acetone-*d*6) *δ* 7.85 (d, *J* = 7.5 Hz, 2H), 7.71 (t, *J* = 7.1 Hz, 2H), 7.49 (dd, *J* = 2.0 Hz, 6.0 Hz, 1H), 7.41 (tt, *J* = 1.5 Hz, 7.5 Hz, 2H), 7.33 (tdd, *J* = 1.2 Hz, 3.8 Hz, 7.4 Hz, 2H), 7.21 (d, *J* = 8.6 Hz, 1H), 7.10 (d, *J* = 8.6 Hz, 2H), 6.77 (d, *J* = 8.6 Hz, 2H), 6.39 (d, *J* = 9.2 Hz, 1H), 6.10 (dd, *J* = 1.6 Hz, 6.0 Hz, 1H), 5.52 (dd, *J* = 5.2 Hz, 11.2 Hz, 1H), 5.47 (dd, *J* = 6.2 Hz, 8.8 Hz, 1H), 5.07 (d, *J* = 10.9 Hz, 1H), 4.96 (t, *J* = 8.0 Hz, 1H), 4.78 (qt, *J* = 1.8 Hz, 6.8 Hz, 1H), 4.44 (dd, *J* = 6.7 Hz, 10.1 Hz, 1H), 4.41 – 4.31 (m, 2H), 4.27 (dd, *J* = 7.4 Hz, 10.1 Hz, 1H), 4.23 (d, *J* = 6.8 Hz, 1H), 4.18 – 4.13 (m, 1H), 4.14 – 4.03 (m, 1H), 3.73 (s, 3H, major rotamer), 3.72 (s, 3H, minor rotamer), 3.57 (dd, *J* = 4.9 Hz, 10.6 Hz, 1H), 3.19 (dd, *J* = 5.4 Hz, 14.4 Hz, 1H), 3.13 (s, 3H), 2.87 (s, 3H), 2.83 (t, *J* = 6.3 Hz, 1H), 2.65 (ddd, *J* = 4.9 Hz, 6.8 Hz, 9.2 Hz, 1H), 2.58 (td, *J* = 4.2 Hz, 8.4 Hz, 1H), 2.35 – 2.16 (m, 1H), 1.79 (dt, *J* = 5.9 Hz, 12.4 Hz, 1H), 1.59 – 1.44 (m, 1H), 1.41 (d, *J* = 6.7 Hz, 3H), 1.23 (dd, *J* = 2.3 Hz, 6.7 Hz, 2H), 1.20 – 1.16 (m, 3H), 1.16 – 1.04 (m, 3H), 1.04 – 0.99 (m, 3H), 0.98 (s, 3H), 0.93 – 0.86 (m, 3H), 0.86 – 0.82 (m, 2H), 0.81 – 0.79 (m, 2H), 0.79 – 0.71 (m, 2H), 0.52 (d, *J* = 5.9 Hz, 3H). **^{13}C NMR** (100 MHz, Acetone-*d*6) *δ* 178.0, 172.8, 171.6, 171.2, 170.5, 168.8, 159.0, 156.9, 155.8, 144.9, 144.8, 141.9, 141.9, 130.5, 130.4, 130.1, 128.4, 128.4, 127.8, 126.1, 125.9, 125.4, 120.7, 120.6, 114.3, 114.2, 70.6, 67.1, 59.7, 59.0, 58.8, 57.5, 55.8, 55.2, 54.1, 49.6, 47.8, 42.3, 37.7, 37.2, 33.8, 33.6, 31.2, 30.7, 30.7, 29.5, 27.9, 23.5, 19.9, 19.3, 18.7, 18.3, 18.3, 17.4, 14.4. **HRMS (ESI)** *m*/*z*: calcd for $C_{56}H_{74}N_6O_{10}Na^+$ [M+Na]$^+$: 1013.5359, found: 1013.5354.

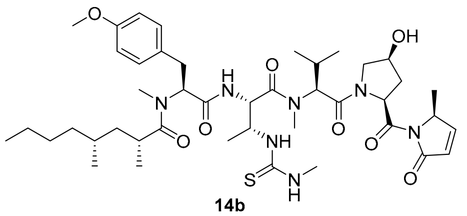

14b

Synthesis of (14b). To the solution of **1f** (260 mg, 0.262 mmol) in DCM (2.6 mL) was added Et_2NH (1.3 mL) at 0 °C. The reaction was allowed to reach room temperature and stirred 4 h. The mixture was quenched with saturated aqueous NH_4Cl (5 mL). The aqueous phase was extracted with EtOAc (3 × 5 mL) and the combined organics were washed with brine (10 mL), dried (Na_2SO_4), filtered, concentrated in vacuo.

To the solution of crude in $CHCl_3$ (2 mL) was added Methyl isothiocyanate (19 mg, 260 μmol) at 0 °C. The reaction was allowed to reach room temperature and stirred overnight. The mixture was concentrated in vacuo and purified by column chromatography (50/1 to 10/1 DCM/MeOH) to provide compound **14b** (109 mg, 51%) as a white solid. $[\boldsymbol{\alpha}]_{\boldsymbol{D}}^{\mathbf{25}}$ = −86.5 (*c* = 1.0, $CHCl_3$). **^{1}H NMR** (400 MHz, $CDCl_3$) *δ* 7.33 (dd, *J* = 7.6 Hz, 30.4 Hz, 1H), 7.23 (s, 1H), 7.07 (d, *J* = 8.2 Hz, 2H), 6.75 (d, *J* = 8.1 Hz, 2H), 6.40 (s, 1H), 6.07 (d, *J* = 6.0 Hz, 1H), 5.65 (dd, *J* = 9.7 Hz, 44.4 Hz, 1H), 5.25 (dd, *J* = 6.2 Hz, 10.6 Hz, 1H), 5.00 (d, *J* = 10.9 Hz, 1H), 4.97 – 4.88 (m, 1H), 4.88 – 4.81 (m, 1H), 4.81 – 4.72 (m, 1H), 4.35 (s, 1H), 3.85 (dd, *J* = 4.6 Hz, 11.5 Hz, 1H), 3.77 (s, 1H), 3.73 (s, 3H), 3.70 – 3.30 (m, 1H), 3.19 (d, *J* = 6.3 Hz, 1H), 3.17 – 3.08 (m, 1H), 3.04 (s, 3H, major rotamer), 3.03 (s, 3H, minor rotamer), 3.00 (s, 3H), 2.96 (s, 3H, major rotamer), 2.94 (s, 3H, minor rotamer), 2.91 – 2.76 (m, 1H), 2.74 – 2.63 (m, 1H), 2.63 – 2.54 (m, 1H), 2.30 – 2.17 (m, 1H), 1.93 (t, *J* = 13.4 Hz, 1H), 1.53 (t, *J* = 9.4 Hz, 1H), 1.45 – 1.36 (m, 1H), 1.33 – 1.18 (m, 3H), 1.18 – 1.13 (m, 3H), 1.13 (s, 2H), 0.99 (s, 2H), 0.98 (s, 3H), 0.93 (d, *J* = 8.7 Hz, 2H), 0.87 (d, *J* = 6.8 Hz, 3H), 0.84 – 0.77 (m, 1H), 0.75 (d, *J* = 6.5 Hz, 3H), 0.55 (d, *J* = 4.6 Hz, 3H). **^{13}C NMR** (100 MHz, $CDCl_3$) *δ* 179.0, 174.5, 173.3, 170.1, 169.8, 169.7, 169.5, 168.5, 168.3, 158.4, 154.3, 154.0, 129.6, 128.3, 125.4, 125.3, 114.0, 71.8, 71.5, 59.6, 58.6, 58.6, 58.1, 57.9, 56.7, 56.5, 55.1, 53.1, 52.0, 41.7, 37.1, 36.9, 36.5, 33.8, 32.8, 31.3, 30.7, 30.4, 30.3, 29.0, 27.0, 26.9, 23.0, 19.5, 19.2, 19.0, 18.8, 18.3, 18.3, 17.9, 17.6, 16.9, 14.1. **HRMS (ESI)** *m*/*z*: calcd for $C_{43}H_{68}N_7O_8S^+$ $[M+H]^+$: 842.4845, found: 842.4847.

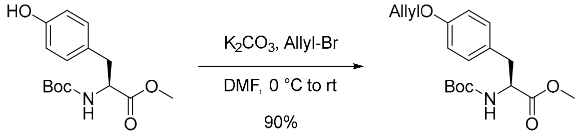

Synthesis of (26). To the solution of **25** (10.0 g, 33.9 mmol) in DMF (100 mL), then K_2CO_3 (12.7 g, 74.5 mmol) was added at 0 °C. After the mixture had been stirred for 30 min at 0 °C, Allyl-Br (5.9 mL, 67.7 mmol) was added dropwise at 0 °C. The reaction was allowed to reach room temperature and stirred overnight., and then the reaction mixture was extracted with

EtOAc (200 mL) first, then washed with brine (5 × 100 mL), dried (Na_2SO_4), filtered, concentrated in vacuo and purified by column chromatography (15/1 to 3/1 petroleum ether/EtOAc) to provide compound **26** (11.8 g, 90%) as a pale yellow oil. $[\alpha]_D^{22}$ = 44.8 (*c* = 0.5, $CHCl_3$). **^{1}H NMR** (400 MHz, $CDCl_3$) δ 7.02 (d, *J* = 8.1 Hz, 2H), 6.83 (d, *J* = 8.5 Hz, 2H), 6.04 (m, 1H), 5.39 (dd, *J* = 17.3, 1.7 Hz, 1H), 5.27 (dd, *J* = 10.5, 1.8 Hz, 1H), 4.97 (d, *J* = 8.3 Hz, 1H), 4.58 – 4.52 (m, 1H), 4.50 (d, *J* = 5.4 Hz, 2H), 3.70 (s, 3H), 3.01 (m, 2H), 1.41 (s, 9H). **^{13}C NMR** (100 MHz, $CDCl_3$) δ 172.5, 157.8, 155.2, 133.4, 130.4, 128.2, 117.7, 114.9, 79.9, 68.9, 54.6, 52.3, 37.6, 29.8, 28.4. **HRMS (ESI)** *m/z*: calcd for $C_{18}H_{25}NO_5Na^+$ $[M+Na]^+$: 358.1625, found: 358.1620.

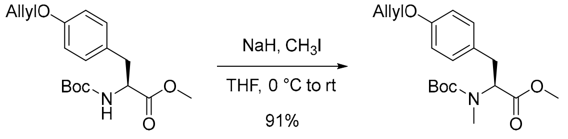

26 27

Synthesis of (27). To the solution of **26** (11.2 g, 33.4 mmol) in THF (100 mL), then NaH (2.00 g, 50.1 mmol) was added at 0 °C. After the mixture had been stirred for 15 min at 0 °C, CH_3I (6.2 mL, 100.2 mmol) was added dropwise at 0 °C. The reaction was allowed to reach room temperature and stirred overnight. The mixture was quenched with H_2O (50 mL) and concentrated, then extracted with EtOAc (200 mL) first, washed with brine (300 mL), dried (Na_2SO_4), filtered, concentrated in vacuo and purified by column chromatography (15/1 to 3/1 petroleum ether/EtOAc) to provide compound **27** (10.6 g, 91%) as a pale yellow oil. $[\alpha]_D^{22}$ = −5.6 (*c* = 0.5, $CHCl_3$). **^{1}H NMR** (400 MHz, $CDCl_3$) δ 7.08 (dd, *J* = 14.9, 8.1 Hz, 2H), 6.82 (d, *J* = 8.1 Hz, 2H), 6.03 (m, 1H), 5.38 (d, *J* = 17.2 Hz, 1H), 5.26 (d, *J* = 10.7 Hz, 1H), 4.88 (s, 1H rotamer), 4.49 (d, *J* = 5.4 Hz, 2H), 4.44 (s, 1H rotamer), 3.72 (d, *J* = 6.3 Hz, 3H), 3.21 (dt, *J* = 14.6, 7.0 Hz, 1H), 2.99 – 2.89 (m, 1H), 2.71 (s, 3H), 1.35 (d, *J* = 13.1 Hz, 9H). **^{13}C NMR** (100 MHz, $CDCl_3$) δ 172.1, 171.7, 157.5, 155.9, 155.1, 133.4, 130.1, 130.0, 129.7, 117.7, 114.9, 114.7, 80.3, 80.0, 68.9, 61.9, 59.7, 53.5, 52.2, 34.7, 34.3, 32.7, 32.0, 31.9, 29.8, 29.8, 29.5, 28.4, 28.3, 22.8, 14.2. **HRMS (ESI)** *m/z*: calcd for $C_{19}H_{27}NO_5Na^+$ $[M+Na]^+$: 372.1781, found: 372.1776.

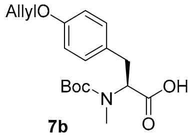

7b

Synthesis of (7b). The titled compound **7b** was obtained following the general procedure described for **22**.The reaction was purified by column chromatography (10/1 to 1/1 petroleum ether/EtOAc) to provide compound **7b** (6.8 g, 66%) as a colorless oil. $[\alpha]_D^{22}$ = −6.0 (*c* = 0.5, $CHCl_3$). **^{1}H NMR** (400 MHz, $CDCl_3$) δ 11.12 (s, 1H), 7.09 (dd, *J* = 8.8, 4.5 Hz, 2H), 6.85 (dd, *J* = 7.5, 4.4 Hz, 2H), 6.04 (m, 1H), 5.40 (d, *J* = 17.3 Hz, 1H), 5.27 (d, *J* = 10.9 Hz, 1H), 4.82 (m, 1H rotamer), 4.61 – 4.55 (m, 1H rotamer), 4.53 (d, *J* = 14.7 Hz, 2H), 3.25 (m, 1H), 3.01 (m, 1H), 2.72

(d, *J* = 23.0 Hz, 3H), 1.38 (dd, *J* = 18.1, 4.4 Hz, 9H). **^{13}C NMR** (100 MHz, $CDCl_3$) *δ* 176.7, 176.5, 157.6, 157.5, 156.4, 155.2, 133.4, 130.0, 130.0, 129.7, 129.4, 117.7, 115.0, 114.9, 80.8, 80.7, 68.9, 61.7, 60.7, 34.5, 34.0, 32.9, 32.8, 29.8, 28.4, 28.3. **HRMS (ESI)** *m/z*: calcd for $C_{18}H_{25}NO_5Na^+$ [M+Na]$^+$: 358.1625, found: 358.1622.

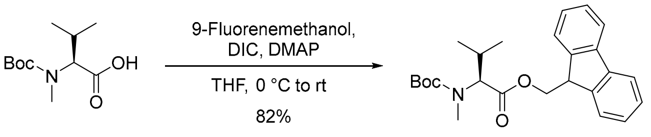

28 **29**

Synthesis of (29). To the solution of **28** (10.0 g, 43.2 mmol) in THF (150 mL) was added 9-Fluorenemethanol (10.2 g, 51.9 mmol), DIC (8.0 mL, 51.9 mmol) and DAMP (1.06 g, 8.65 mmol) at 0 °C. The reaction was allowed to reach room temperature and stirred overnight. The mixture was concentrated, diluted with EtOAc (200 mL) and washed with aqueous 5% $NaHSO_4$ (150 mL), saturated aqueous $NaHCO_3$ (150 mL) and brine (150 mL), dried (Na_2SO_4), filtered, concentrated in vacuo and purified by column chromatography (50/1 to 15/1 petroleum ether/EtOAc) to provide compound **29** (14.6 g, 82%) as a pale yellow oil. $[\alpha]_D^{22}$ = −44.2 (*c* = 0.5, $CHCl_3$). **^{1}H NMR** (400 MHz, $CDCl_3$) *δ* 7.76 (d, *J* = 7.6 Hz, 2H), 7.60 (dd, *J* = 13.1, 7.4 Hz, 2H), 7.40 (t, *J* = 7.5 Hz, 2H), 7.36 – 7.27 (m, 2H), 4.63 – 4.43 (m, 2H), 4.40 – 4.17 (m, 2H), 2.75 (d, *J* = 9.3 Hz, 3H), 2.14 (m, 1H), 1.48 (d, *J* = 16.8 Hz, 9H), 0.99 – 0.87 (m, 6H). **^{13}C NMR** (100 MHz, $CDCl_3$) *δ* 171.5, 171.0, 156.3, 155.6, 143.9, 143.7, 141.4, 128.0, 127.9, 127.3, 125.3, 125.1, 120.1, 80.4, 80.1, 66.7, 66.5, 65.2, 63.4, 62.5, 46.9, 30.4, 28.5, 28.5, 27.7, 27.6, 21.2, 20.1, 19.9, 19.0, 18.8. **HRMS (ESI)** *m/z*: calcd for $C_{25}H_{31}NO_4Na^+$ [M+Na]$^+$: 423.2145, found: 423.2139.

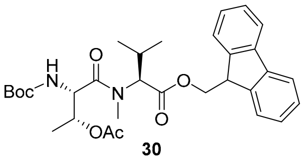

30

Synthesis of (30). The titled compound **30** was obtained following the general procedure described for **17**. The reaction was purified by column chromatography (25/1 to 5/1 petroleum ether/EtOAc) to provide compound **30** (7.5 g, 67%) as a colorless oil. $[\alpha]_D^{22}$ = −47.0 (*c* = 0.5, $CHCl_3$). **^{1}H NMR** (400 MHz, $CDCl_3$) *δ* 7.75 (d, *J* = 7.5 Hz, 2H), 7.61 (dd, *J* = 7.5, 3.1 Hz, 2H), 7.39 (t, *J* = 7.6 Hz, 2H), 7.31 (t, *J* = 7.4 Hz, 2H), 5.39 (d, *J* = 9.4 Hz, 1H), 5.25 – 5.15 (m, 1H), 4.91 (d, *J* = 10.5 Hz, 1H), 4.64 (dd, *J* = 9.5, 5.1 Hz, 1H), 4.60 – 4.55 (m, 1H), 4.46 (dd, *J* = 10.8, 6.6 Hz, 1H), 4.21 (t, *J* = 6.4 Hz, 1H), 2.92 (s, 3H), 2.13 – 2.04 (m, 1H), 1.92 (s, 3H), 1.42 (s, 9H), 1.18 (d, *J* = 6.5 Hz, 3H), 0.90 (d, *J* = 6.6 Hz, 3H), 0.80 (d, *J* = 6.7 Hz, 3H). **^{13}C NMR** (100 MHz, $CDCl_3$) *δ* 170.7, 170.4, 170.3, 170.1, 155.8, 155.5, 143.7, 143.5, 143.5, 141.4, 141.3, 127.8, 127.3, 127.2, 127.2, 125.1, 125.0, 125.0, 120.1, 120.0, 80.0, 70.2, 69.2, 66.6, 66.3, 62.1, 61.8, 54.0, 53.7, 46.8, 46.7, 31.2, 31.2, 29.7, 28.3,

28.3, 27.3, 27.3, 21.0, 20.9, 19.6, 19.6, 18.7, 18.7, 16.9, 16.7. **HRMS (ESI)** *m*/*z*: calcd for $C_{31}H_{40}N_2O_7Na^+$ [M+Na]+: 575.2728, found: 575.2725.

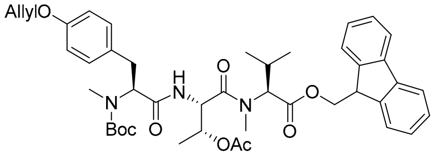

31

Synthesis of (31). The titled compound **31** was obtained following the general procedure described for **8b**. The reaction was purified by column chromatography (20/1 to 3/1 petroleum ether/EtOAc) to provide compound **31** (7.22 g, 65%) as a colorless oil.$[\alpha]_D^{22}$ = 11.6 (*c* = 0.5, $CHCl_3$). **^{1}H NMR** (400 MHz, $CDCl_3$) *δ* 7.75 (d, *J* = 7.6 Hz, 2H), 7.57 (dd, *J* = 25.1, 7.3 Hz, 2H), 7.40 (t, *J* = 7.9 Hz, 2H), 7.31 (t, *J* = 7.5 Hz, 2H), 7.13 – 7.01 (m, 2H), 6.80 (m, 3H), 6.04 (m, 1H), 5.37 (dd, *J* = 17.5, 10.2 Hz, 1H), 5.25 (t, *J* = 10.2 Hz, 2H), 5.11 – 4.93 (m, 1H), 4.92 – 4.82 (m, 1H), 4.76 (s, 1H), 4.56 (dd, *J* = 11.0, 6.2 Hz, 1H), 4.49 (d, *J* = 4.4 Hz, 2H), 4.46 (s, 1H), 4.26 – 4.16 (m, 1H), 3.35 – 3.15 (m, 1H), 3.02 – 2.91 (m, 3H), 2.78 – 2.62 (m, 4H), 2.06 (m, 1H), 2.03 – 1.83 (m, 3H), 1.37 (dd, *J* = 18.7, 8.7 Hz, 9H), 1.20 – 0.96 (m, 3H), 0.89 (d, *J* = 6.5 Hz, 3H), 0.82 – 0.71 (m, 3H). **^{13}C NMR** (100 MHz, $CDCl_3$) *δ* 170.3, 157.4, 143.7, 143.6, 143.5, 141.3, 133.4, 129.8, 127.9, 127.9, 127.8, 127.3, 127.2, 125.1, 125.1, 125.1, 125.0, 124.9, 124.6, 120.1, 120.0, 120.0, 120.0, 117.6, 114.9, 114.7, 80.4, 69.6, 68.8, 66.6, 66.3, 62.3, 61.8, 52.4, 46.8, 46.7, 33.2, 31.9, 31.4, 31.3, 31.2, 30.2, 29.7, 29.4, 28.2, 27.4, 27.3, 22.7, 20.9, 20.8, 19.6, 19.5, 18.8, 16.9, 16.8, 14.1. **HRMS (ESI)** *m*/*z*: calcd for $C_{44}H_{55}N_3O_9Na^+$ [M+Na]+: 792.3831, found: 792.3827.

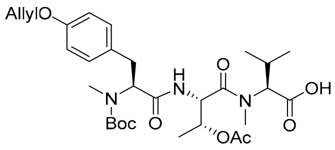

3g

Synthesis of (3g). To the solution of **31** (7.22 g, 9.40 mmol) in DCM (47 mL) was added Et_2NH (47 mL) at 0 °C. The reaction was allowed to reach room temperature and stirred 5 h. The mixture was concentrated in vacuo and purified by column chromatography (100/1 to 20/1 DCM/MeOH) to provide compound **3g** (4.36 g, 79%) as a white foamed solid. $[\alpha]_D^{22}$ = −15.0 (*c* = 0.5, $CHCl_3$). **^{1}H NMR** (400 MHz, $CDCl_3$) *δ* 7.14 – 6.97 (m, 3H), 6.81 (d, *J* = 7.5 Hz, 2H), 6.03 (m, 1H), 5.38 (d, *J* = 17.2 Hz, 1H), 5.27 (t, *J* = 9.4 Hz, 2H), 5.08 – 4.94 (m, 1H), 4.74 (t, *J* = 11.9 Hz, 1H), 4.49 (d, *J* = 5.2 Hz, 2H), 3.34 – 3.17 (m, 1H), 3.14 – 2.87 (m, 4H), 2.81 – 2.65 (m, 3H), 2.21 (s, 1H), 1.96 (d, *J* = 17.9 Hz, 3H), 1.37 (d, *J* = 5.4 Hz, 9H), 1.20 (d, *J* = 7.8 Hz, 3H), 1.03 (d, *J* = 6.5 Hz, 3H), 0.81 (d, *J* = 6.8 Hz, 3H). **^{13}C NMR** (100 MHz, $CDCl_3$) *δ* 173.4, 171.2, 170.7, 170.0, 169.8, 157.4, 157.3, 156.3, 156.1, 155.1, 133.3, 133.3, 130.0, 129.8, 129.6, 117.6, 114.8, 114.7, 80.9, 80.5, 69.3, 68.8,

59.7, 52.7, 38.6, 33.2, 31.8, 29.7, 28.3, 28.2, 28.2, 28.1, 26.9, 20.9, 20.9, 20.9, 19.9, 19.7, 18.8, 18.7, 16.8. **HRMS (ESI)** *m*/*z*: calcd for $C_{30}H_{45}N_3O_9Na^+$ [M+Na]$^+$: 614.3048, found: 614.3045.

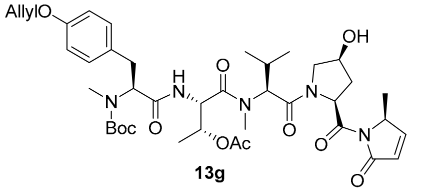

13g

Synthesis of (13g). The titled compound **13g** was obtained following the general procedure described for **13b**. The reaction was purified by column chromatography (100/1 to 20/1 DCM/MeOH) to provide compound **13g** (1.17 g, 63%) as a white foamed solid. $[\boldsymbol{\alpha}]_{\boldsymbol{D}}^{\mathbf{22}}$ = −59.2 (*c* = 0.5, $CHCl_3$). **^{1}H NMR** (400 MHz, $CDCl_3$) δ 7.24 (s, 1H), 7.07 (m, 2H), 6.87 – 6.66 (m, 3H), 6.05 (d, *J* = 6.1 Hz, 1H), 5.98 (m, 1H), 5.62 (d, *J* = 9.7 Hz, 1H), 5.36 (d, *J* = 17.2 Hz, 1H), 5.23 (d, *J* = 10.5 Hz, 2H), 4.96 (m, 2H), 4.77 (m, 1H), 4.46 (d, *J* = 5.3 Hz, 2H), 4.33 (s, 1H), 3.79 (m, 2H), 3.57 (d, *J* = 10.7 Hz, 1H), 3.23 (m, 1H), 3.11 – 2.96 (m, 3H), 2.90 – 2.79 (m, 1H), 2.71 (dd, *J* = 34.0, 14.9 Hz, 3H), 2.45 (m, 1H), 2.24 (m, 1H), 1.99 (s, 1H), 1.93 (d, *J* = 19.4 Hz, 3H), 1.43 (d, *J* = 6.8 Hz, 3H), 1.35 (d, *J* = 15.5 Hz, 9H), 1.15 (dd, *J* = 14.9, 7.3 Hz, 3H), 0.95 (t, *J* = 5.2 Hz, 3H), 0.79 – 0.70 (m, 3H). **^{13}C NMR** (100 MHz, $CDCl_3$) δ 174.4, 170.6, 170.0, 169.9, 169.8, 168.8, 157.3, 156.4, 156.2, 155.1, 154.3, 133.4, 130.0, 129.9, 129.7, 125.4, 117.6, 114.9, 114.7, 80.7, 80.5, 71.8, 71.7, 69.0, 68.8, 68.7, 61.3, 60.1, 59.7, 59.3, 58.6, 58.2, 56.9, 56.8, 52.3, 52.3, 51.9, 36.7, 36.6, 33.3, 33.2, 32.0, 30.9, 30.5, 30.4, 30.3, 29.7, 29.4, 28.3, 28.2, 27.2, 27.1, 22.7, 21.1, 21.0, 19.0, 18.9, 18.4, 18.4, 17.6, 17.4, 17.1, 17.0, 14.2. **HRMS (ESI)** *m*/*z*: calcd for $C_{40}H_{57}N_5O_{11}Na^+$ [M+Na]$^+$: 806.3947, found: 806.3943.

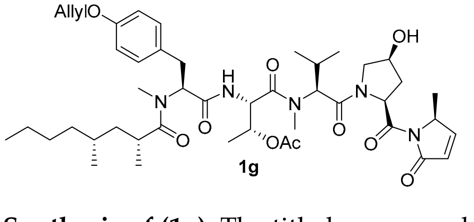

1g

Synthesis of (1g). The titled compound **1g** was obtained following the general procedure described for **1b**. The reaction was purified by column chromatography (100/1 to 20/1 DCM/MeOH) to provide compound **1g** (1.19 g, 43%) as a white foamed solid. $[\boldsymbol{\alpha}]_{\boldsymbol{D}}^{\mathbf{22}}$ = −27.0 (*c* = 0.3, $CHCl_3$). **^{1}H NMR** (400 MHz, $CDCl_3$) δ 7.25 (d, *J* = 4.6 Hz, 1H), 7.07 (d, *J* = 8.3 Hz, 2H), 7.03 (d, *J* = 9.2 Hz, 1H), 6.77 (d, *J* = 8.2 Hz, 2H), 6.06 (d, *J* = 6.3 Hz, 1H), 5.99 (dd, *J* = 10.7, 5.4 Hz, 0H), 5.64 (d, *J* = 9.7 Hz, 1H), 5.54 (m, 1H), 5.37 (d, *J* = 17.1 Hz, 1H), 5.24 (d, *J* = 10.4 Hz, 2H), 4.97 (dd, *J* = 15.0, 10.4 Hz, 2H), 4.79 (m, 1H), 4.45 (d, *J* = 5.4 Hz, 2H), 4.35 (s, 1H), 3.82 (d, *J* = 4.3 Hz, 2H), 3.54 (s, 1H), 3.14 (dd, *J* = 15.4, 5.9 Hz, 1H), 3.06 (s, 3H), 2.99 – 2.94 (m, 1H), 2.91 (s, 3H), 2.63 (m, 1H), 2.46 (m, 1H), 2.24 (m, 1H), 2.01 (s, 1H), 1.96 (s, 3H), 1.62 (m, 1H), 1.44 (d, *J* = 6.8 Hz, 3H), 1.24 (s, 5H), 1.15 (d, *J* = 6.4 Hz, 4H), 1.04 (d, *J* = 6.7 Hz, 3H), 0.97 (d, *J* = 6.5 Hz, 3H), 0.86 (t, *J* = 7.0 Hz, 5H), 0.79 (d, *J* = 6.6 Hz, 3H), 0.50 (d, *J* = 5.1 Hz, 3H). **^{13}C NMR** (100 MHz, $CDCl_3$) δ 178.0,

174.7, 171.0, 169.9, 169.8, 168.7, 157.3, 154.1, 133.4, 129.9, 129.8, 129.7, 129.2, 125.4, 117.5, 114.7, 114.7, 71.9, 71.9, 68.8, 68.8, 68.4, 59.2, 58.6, 58.6, 58.1, 56.9, 56.8, 52.2, 41.0, 37.2, 36.8, 36.6, 36.6, 33.4, 33.0, 31.9, 31.4, 31.0, 30.4, 30.3, 30.3, 30.2, 29.7, 29.6, 29.6, 29.5, 29.5, 29.3, 29.3, 29.3, 29.2, 29.1, 29.0, 29.0, 27.2, 27.0, 24.8, 23.1, 23.0, 22.7, 21.0, 20.9, 20.2, 18.9, 18.8, 18.5, 18.4, 18.1, 17.6, 17.4, 16.9, 14.1, 14.1. **HRMS (ESI)** *m*/*z*: calcd for $C_{45}H_{67}N_5O_{10}Na^+$ $[M+Na]^+$: 860.4780, found: 860.4777.

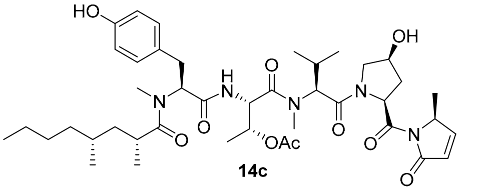

14c

Synthesis of (14c). To the solution of **1g** (100 mg, 0.12 mmol) in THF (4 mL), then $(Ph_3)_4Pd$ (27.6 mg, 24 μmol) and $PhSiH_3$ (29 μL, 0.24 mmol) was added, and then stirred overnight. The mixture was washed with brine (10 mL), dried (Na_2SO_4), filtered, concentrated in vacuo and purified by column chromatography (80/1 to 15/1 DCM/MeOH) to provide compound **14c** (58 mg, 61%) as a white foamed solid. $[\boldsymbol{\alpha}]_{\boldsymbol{D}}^{22}$ = −83.4 (*c* = 0.5, $CHCl_3$). **¹H NMR** (400 MHz, $CDCl_3$) *δ* 7.23 (dd, *J* = 6.0, 2.3 Hz, 1H), 7.03 (dd, *J* = 8.8, 3.3 Hz, 1H), 6.97 (t, *J* = 8.8 Hz, 2H), 6.66 (d, *J* = 8.4 Hz, 2H), 6.04 (m, 1H), 5.61 (dd, *J* = 9.9, 2.5 Hz, 1H), 5.57 – 5.43 (m, 1H), 5.21 (m, 1H), 4.93 (m, 2H), 4.75 (m, 1H), 4.34 (s, 1H), 3.82 – 3.76 (m, 2H), 3.73 – 3.64 (m, 2H), 3.12 – 3.06 (m, 1H), 3.03 (s, 3H), 2.91 (s, 3H), 2.78 (dd, *J* = 10.7, 2.9 Hz, 1H), 2.65 (m, 1H), 2.45 (m, 1H), 2.24 – 2.17 (m, 1H), 1.98 (s, 1H), 1.93 (s, 3H), 1.58 (m, 1H), 1.40 (d, *J* = 6.7 Hz, 3H), 1.21 (s, 5H), 1.13 (d, *J* = 6.6 Hz, 4H), 1.03 (dd, *J* = 6.7 Hz, 3H), 0.98 – 0.93 (m, 4H), 0.83 (m, 4H), 0.75 (t, *J* = 6.5 Hz, 3H), 0.55 (d, *J* = 5.7 Hz, 3H). **¹³C NMR** (100 MHz, $CDCl_3$) *δ* 178.8, 178.4, 175.7, 174.6, 174.5, 171.0, 170.8, 170.0, 170.0, 169.9, 169.9, 169.8, 169.0, 168.9, 155.5, 155.4, 154.3, 130.0, 129.9, 129.9, 129.8, 127.9, 127.8, 125.4, 115.6, 115.6, 115.5, 71.9, 71.8, 68.5, 68.3, 59.4, 59.3, 58.8, 58.7, 58.2, 57.1, 57.0, 56.9, 53.6, 52.4, 52.1, 41.6, 41.0, 37.1, 36.8, 36.7, 36.7, 33.6, 33.5, 33.0, 32.7, 32.0, 31.3, 31.1, 31.0, 30.5, 30.4, 30.4, 30.4, 29.0, 27.3, 27.1, 25.3, 23.2, 23.1, 23.1, 21.1, 21.0, 20.3, 19.6, 19.2, 19.2, 19.0, 18.9, 18.6, 18.5, 18.1, 17.6, 17.6, 17.0, 17.0, 14.3, 14.2. **HRMS (ESI)** *m*/*z*: calcd for $C_{45}H_{67}N_5O_{10}Na^+$ $[M+Na]^+$: 820.4467, found: 820.4463.

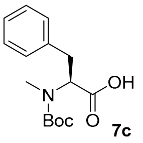

7c

Synthesis of (7c). The titled compound **7c** was obtained following the general procedure described for **27**. The reaction was purified by column chromatography (20/1 to 3/1 petroleum ether/EtOAc) to provide compound **7c** (5.61 g, quant) as a pale yellow oil. $[\boldsymbol{\alpha}]_{\boldsymbol{D}}^{22}$ = −79.6 (*c* = 0.5,

CHCl3). **^{1}H NMR** (400 MHz, $CDCl_3$) δ 10.93 (s, 1H), 7.29 (m, 2H), 7.25 – 7.17 (m, 3H), 4.76 (m, 1H), 3.33 (m, 1H), 3.06 (m, 1H), 2.73 (d, J = 25.2 Hz, 3H), 1.36 (d, J = 20.8 Hz, 9H). **^{13}C NMR** (100 MHz, $CDCl_3$) δ 176.7, 176.6, 156.4, 155.2, 137.6, 137.2, 129.1, 129.0, 128.7, 128.6, 126.9, 126.8, 80.9, 80.7, 61.6, 60.5, 35.4, 34.8, 32.9, 32.7, 28.4, 28.3. **HRMS (ESI)** *m*/*z*: calcd for $C_{15}H_{21}NO_4Na^+$ $[M+Na]^+$: 302.1363, found: 302.1359.

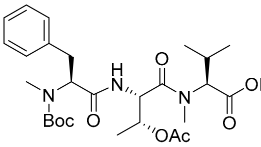

8h

Synthesis of (8h). The titled compound **8h** was obtained following the general procedure described for **8b**. The reaction was purified by column chromatography (25/1 to 5/1 petroleum ether/EtOAc) to provide compound **8h** (4.8 g, 87%) as a colorless oil. $[\alpha]_D^{22}$ = −83.4 (c = 0.5, $CHCl_3$). **^{1}H NMR** (400 MHz, $CDCl_3$) δ 7.33 (s, 5H), 7.18 (m, 5H), 6.81 (dd, J = 49.3, 8.7 Hz, 1H), 5.25 (m, 1H), 5.14 (s, 2H), 4.98 (m, 1H), 4.92 – 4.73 (m, 2H), 3.28 (m, 1H), 3.02 (d, J = 40.0 Hz, 3H), 2.88 (d, J = 34.7 Hz, 1H), 2.74 (d, J = 31.2 Hz, 3H), 2.19 (d, J = 6.4 Hz, 1H), 1.93 (s, 3H), 1.36 (d, J = 22.3 Hz, 9H), 1.14 (d, J = 6.4 Hz, 3H), 0.97 (m, 3H), 0.78 (dd, J = 16.5, 6.7 Hz, 3H). **^{13}C NMR** (100 MHz, $CDCl_3$) δ 170.5, 170.3, 169.8, 137.6, 135.6, 129.2, 129.0, 128.7, 128.5, 126.7, 81.0, 80.6, 69.6, 69.3, 67.2, 66.9, 61.9, 61.0, 60.1, 53.0, 52.4, 34.2, 31.6, 31.4, 30.3, 29.8, 28.4, 28.2, 27.3, 21.0, 19.8, 18.8, 17.0, 16.8. **HRMS (ESI)** *m*/*z*: calcd for $C_{34}H_{47}N_3O_8Na^+$ $[M+Na]^+$: 648.3255, found: 648.3251.

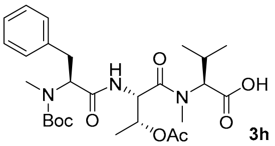

3h

Synthesis of (3h). The titled compound **3h** was obtained following the general procedure described for **3b**. The reaction was purified by column chromatography (25/1 petroleum ether/EtOAc to EtOAc) to provide compound **3h** (3.60 g, 88%) as a white foamed solid. $[\alpha]_D^{22}$ = −73.6 (c = 0.5, $CHCl_3$). **^{1}H NMR** (400 MHz, $CDCl_3$) δ 10.00 (s, 1H), 7.19 (m, 5H), 7.01 (dd, J = 57.1, 9.0 Hz, 1H), 5.28 (s, 1H), 5.04 (s, 1H), 4.80 (m, 2H), 3.26 (m, 1H), 3.04 (d, J = 37.0 Hz, 3H), 2.95 – 2.82 (m, 1H), 2.74 (d, J = 31.1 Hz, 3H), 2.28 – 2.10 (m, 1H), 1.96 (s, 3H), 1.34 (d, J = 22.4 Hz, 9H), 1.22 (d, J = 6.8 Hz, 3H), 1.00 (s, 3H), 0.77 (dd, J = 15.7, 6.7 Hz, 3H). **^{13}C NMR** (100 MHz, $CDCl_3$) δ 173.3, 173.1, 170.8, 170.7, 170.0, 169.8, 156.1, 155.1, 137.5, 137.4, 131.0, 129.0, 129.0, 128.9, 128.6, 128.4, 126.7, 126.5, 81.0, 80.8, 80.5, 69.6, 69.4, 65.6, 62.3, 60.9, 60.5, 60.0, 52.8, 52.4, 34.1, 31.9, 31.8, 31.6, 30.6, 30.3, 29.7, 29.7, 28.3, 28.2, 28.1, 26.9, 21.1, 21.0, 19.9, 19.8, 19.2, 19.1, 18.8, 17.4, 17.0, 16.8, 14.2, 13.8. **HRMS (ESI)** *m*/*z*: calcd for $C_{27}H_{41}N_3O_8Na^+$ $[M+Na]^+$: 558.2786, found: 558.2780.

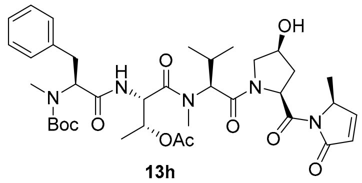

13h

Synthesis of (13h). The titled compound **13h** was obtained following the general procedure described for **13b**.The reaction was purified by column chromatography (100/1 to 20/1 DCM/MeOH) to provide compound **13h** (483 mg, 56%) as a white foamed solid. $[\alpha]_D^{22}$ = −94.6 (*c* = 0.5, $CHCl_3$). **^{1}H NMR** (400 MHz, $CDCl_3$) *δ* 7.28 (d, *J* = 2.1 Hz, 1H), 7.26 – 7.14 (m, 5H), 6.79 (dd, *J* = 57.7, 8.8 Hz, 1H), 6.09 (dd, *J* = 6.2, 1.6 Hz, 1H), 5.67 (d, *J* = 10.0 Hz, 1H), 5.27 (s, 1H), 4.99 (s, 2H), 4.90 – 4.76 (m, 2H), 4.37 (s, 1H), 3.88 (d, *J* = 11.6 Hz, 1H), 3.77 (dd, *J* = 11.6, 4.4 Hz, 1H), 3.54 (d, *J* = 11.1 Hz, 1H), 3.31 (dd, *J* = 14.5, 5.8 Hz, 1H), 3.07 (d, *J* = 38.9 Hz, 3H), 2.99 – 2.89 (m, 1H), 2.77 (d, *J* = 36.1 Hz, 3H), 2.52 – 2.41 (m, 1H), 2.32 – 2.18 (m, 1H), 2.05 (s, 1H), 1.99 (s, 3H), 1.46 (d, *J* = 6.8 Hz, 3H), 1.37 (d, *J* = 26.9 Hz, 9H), 1.22 (m, 3H), 0.98 (s, 3H), 0.77 (dd, *J* = 19.5, 6.9 Hz, 3H). **^{13}C NMR** (100 MHz, $CDCl_3$) *δ* 174.4, 170.4, 169.9, 169.9, 169.8, 169.8, 168.8, 168.7, 156.1, 155.1, 154.3, 137.6, 137.4, 129.0, 128.9, 128.6, 128.4, 126.6, 126.5, 125.3, 80.9, 80.5, 71.7, 68.9, 68.7, 61.1, 59.9, 59.2, 58.6, 58.1, 56.8, 52.4, 51.9, 36.6, 34.1, 34.0, 31.5, 30.5, 29.7, 28.3, 28.2, 27.2, 21.0, 18.9, 18.4, 17.6, 17.3, 17.0. **HRMS (ESI)** *m/z*: calcd for $C_{37}H_{53}N_5O_{10}Na^+$ $[M+Na]^+$: 750.3685, found: 750.3679.

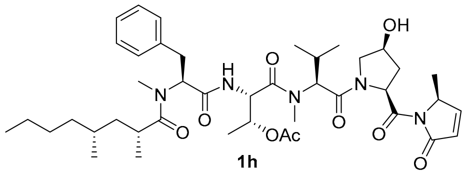

1h

Synthesis of (1h). The titled compound **1h** was obtained following the general procedure described for **1b**. The reaction was purified by column chromatography (100/1 to 20/1 DCM/MeOH) to provide compound **1h** (197 mg, 61%) as a white foamed solid. $[\alpha]_D^{22}$ = −122.8 (*c* = 0.5, $CHCl_3$). **^{1}H NMR** (400 MHz, $CDCl_3$) *δ* 7.27 (d, *J* = 1.9 Hz, 1H), 7.24 – 7.13 (m, 5H), 7.04 (d, *J* = 8.9 Hz, 1H), 6.08 (dd, *J* = 6.1, 1.6 Hz, 1H), 5.66 (dd, *J* = 10.1, 2.1 Hz, 1H), 5.65 – 5.60 (m, 1H), 5.25 (dd, *J* = 6.6, 2.7 Hz, 1H), 5.00 (d, *J* = 11.2 Hz, 1H), 4.96 (dd, *J* = 9.2, 3.0 Hz, 1H), 4.85 – 4.76 (m, 1H), 4.37 (d, *J* = 10.6 Hz, 1H), 3.87 (d, *J* = 11.4 Hz, 1H), 3.81 (dd, *J* = 11.6, 4.4 Hz, 1H), 3.50 (d, *J* = 11.1 Hz, 1H), 3.22 (dd, *J* = 15.0, 5.7 Hz, 1H), 3.06 (s, 3H), 3.04 – 2.96 (m, 1H), 2.92 (s, 3H), 2.66 – 2.59 (m, 1H), 2.46 (m, 1H), 2.26 (m, 1H), 2.02 (d, *J* = 15.1 Hz, 1H), 1.98 (s, 3H), 1.67 – 1.60 (m, 1H), 1.46 (d, *J* = 6.7 Hz, 3H), 1.27 – 1.19 (m, 4H), 1.16 (d, *J* = 6.5 Hz, 5H), 1.06 (d, *J* = 6.7 Hz, 3H), 0.99 (d, *J* = 6.5 Hz, 3H), 0.88 (t, *J* = 7.1 Hz, 4H), 0.81 (d, *J* = 6.7 Hz, 4H), 0.48 (d, *J* = 5.7 Hz, 3H). **^{13}C NMR** (100 MHz, $CDCl_3$); *δ* 178.0, 174.6, 170.7, 169.9, 169.8, 169.6, 169.0, 154.2, 136.9, 128.8, 128.5, 128.5, 126.7, 125.4, 71.8, 68.5, 59.2, 58.7, 58.2, 57.0, 56.3, 52.0, 52.0, 41.6, 37.2, 36.7,

33.5, 33.3, 30.7, 30.4, 30.4, 30.3, 29.7, 29.0, 27.2, 23.1, 21.1, 19.4, 18.9, 18.6, 18.5, 18.2, 17.5, 17.0, 14.2, 14.2. **HRMS (ESI)** *m/z*: calcd for $C_{42}H_{63}N_5O_9Na^+$ $[M+Na]^+$: 804.4518, found: 804.4519.

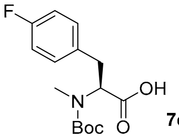

7d

Synthesis of (7d). The titled compound **7d** was obtained following the general procedure described for **27**. The reaction was purified by column chromatography (100/1 to 30/1 DCM/MeOH) to provide compound **7d** (6.07 g, quant) as a pale yellow oil. $[\alpha]_D^{22}$ = −64.8 (*c* = 0.5, $CHCl_3$). **^{1}H NMR** (400 MHz, $CDCl_3$) δ 11.32 (s, 1H), 7.16 (m, 2H), 7.04 – 6.93 (m, 2H), 4.84 (dd, *J* = 11.0, 5.2 Hz, 1H rotamer), 4.56 (dd, *J* = 10.8, 4.5 Hz, 1H rotamer), 3.29 (m, 1H), 3.04 (m, 1H), 2.73 (d, *J* = 22.5 Hz, 3H), 1.37 (d, *J* = 17.0 Hz, 9H). **^{13}C NMR** (100 MHz, $CDCl_3$) δ 176.5, 176.4, 160.7, 156.4, 155.1, 133.3, 132.9, 130.6, 130.6, 130.5, 115.7, 115.5, 115.5, 115.3, 81.0, 80.9, 61.7, 60.5, 34.6, 34.1, 32.8, 28.5, 28.4, 28.3, 20.9. **^{19}F NMR** (376 MHz, $CDCl_3$) δ -116.1, -116.3 (rotamer). **HRMS (ESI)** *m/z*: calcd for $C_{15}H_{20}NO_4FNa^+$ $[M+Na]^+$: 320.1269, found: 320.1266.

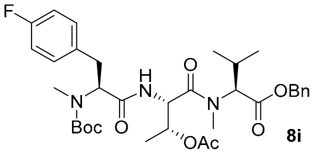

8i

Synthesis of (8i). The titled compound **8i** was obtained following the general procedure described for **8b**. The reaction was purified by column chromatography (25/1 to 5/1 petroleum ether/EtOAc) to provide compound **8i** (7.50 g, 67%) as a colorless oil. $[\alpha]_D^{22}$ = −83.4 (*c* = 0.5, $CHCl_3$). **^{1}H NMR** (400 MHz, $CDCl_3$) δ 7.34 (d, *J* = 4.1 Hz, 5H), 7.13 (m, 2H), 6.95 (m, 2H), 6.79 (dd, *J* = 49.1, 8.7 Hz, 1H), 5.29 – 5.19 (m, 1H), 5.15 (s, 2H), 4.99 (m, 1H), 4.87 (d, *J* = 9.4 Hz, 1H), 4.84 – 4.67 (m, 1H), 3.26 (m, 1H), 3.04 (d, *J* = 28.9 Hz, 3H), 2.96 – 2.83 (m, 1H), 2.75 (d, *J* = 30.2 Hz, 3H), 2.20 (s, 1H), 1.93 (s, 3H), 1.38 (d, *J* = 14.2 Hz, 9H), 1.14 (d, *J* = 6.4 Hz, 3H), 0.97 (s, 3H), 0.83 – 0.73 (m, 3H). **^{13}C NMR** (100 MHz, $CDCl_3$) δ 170.3, 170.0, 169.7, 163.0, 160.5, 156.1, 155.0, 135.6, 133.4, 133.2, 130.6, 130.5, 128.7, 128.6, 128.6, 128.5, 128.5, 128.3, 115.5, 115.3, 115.2, 81.1, 80.7, 69.5, 69.2, 67.1, 66.8, 61.9, 60.9, 60.0, 53.0, 52.4, 33.3, 31.4, 31.4, 30.2, 29.8, 29.3, 28.3, 28.2, 27.2, 21.0, 19.8, 19.7, 18.7, 16.9, 16.8. **^{19}F NMR** (376 MHz, $CDCl_3$) δ -116.4, -116.7 (rotamer). **HRMS (ESI)** *m/z*: calcd for $C_{34}H_{46}N_3O_8FNa^+$ $[M+Na]^+$: 666.3161, found: 666.3165.

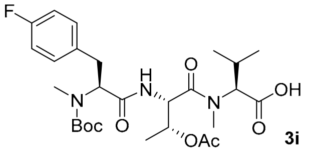

3i

Synthesis of (3i). The titled compound **3i** was obtained following the general procedure described for **3b**. The reaction was purified by column chromatography (25/1 petroleum

ether/EtOAc to EtOAc) to provide compound **3i** (3.53 g, 98%) as a white foamed solid. $[\alpha]_D^{22}$ = −80.4 (*c* = 0.5, $CHCl_3$). **^{1}H NMR** (400 MHz, $CDCl_3$) δ 7.14 (s, 2H), 7.03 – 6.78 (m, 3H), 5.30 (s, 1H), 5.12 – 5.00 (m, 1H), 4.88 – 4.63 (m, 2H), 3.26 (dd, *J* = 14.1, 7.6 Hz, 1H), 3.08 (d, *J* = 24.7 Hz, 3H), 2.95 (m, 1H), 2.76 (d, *J* = 30.9 Hz, 3H), 2.23 (s, 1H), 1.99 (s, 3H), 1.39 (d, *J* = 13.0 Hz, 9H), 1.25 (s, 3H), 1.04 (d, *J* = 6.3 Hz, 3H), 0.80 (d, *J* = 6.9 Hz, 3H). **^{13}C NMR** (100 MHz, $CDCl_3$) δ 173.2, 173.0, 170.7, 170.6, 170.1, 170.0, 162.9, 160.5, 156.1, 155.1, 133.3, 133.1, 130.6, 130.5, 130.5, 115.5, 115.3, 115.1, 81.2, 80.7, 69.6, 69.3, 62.4, 60.9, 60.0, 52.9, 52.4, 33.4, 31.9, 31.6, 30.3, 29.4, 28.3, 28.2, 26.9, 21.0, 19.9, 18.8, 17.0, 16.8. **^{19}F NMR** (376 MHz, $CDCl_3$) δ -116.4, -116.6 (rotamer). **HRMS (ESI)** *m/z*: calcd for $C_{27}H_{40}N_3O_8FNa^+$ $[M+Na]^+$: 576.2692, found: 576.2694.

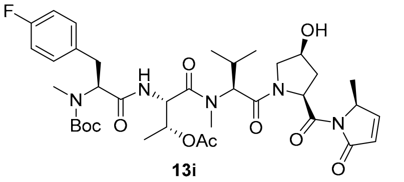

13i

Synthesis of (13i). The titled compound **13i** was obtained following the general procedure described for **13b**. The reaction was purified by column chromatography (100/1 to 20/1 DCM/MeOH) to provide compound **13i** (739 mg, 48%) as a white foamed solid. $[\alpha]_D^{22}$ = −99.2 (*c* = 0.5, $CHCl_3$). **^{1}H NMR** (400 MHz, $CDCl_3$) δ 7.28 (d, *J* = 2.1 Hz, 1H), 7.18 – 7.10 (m, 2H), 6.95 (d, *J* = 7.8 Hz, 2H), 6.77 (dd, *J* = 63.0, 8.8 Hz, 1H), 6.08 (dd, *J* = 6.1, 1.6 Hz, 1H), 5.66 (d, *J* = 9.9 Hz, 1H), 5.26 (s, 1H), 4.98 (d, *J* = 8.6 Hz, 2H), 4.87 – 4.70 (m, 2H), 4.37 (d, *J* = 10.9 Hz, 1H), 3.86 (d, *J* = 11.5 Hz, 1H), 3.77 (dd, *J* = 11.7, 4.1 Hz, 1H), 3.57 – 3.45 (m, 1H), 3.27 (dd, *J* = 14.5, 6.1 Hz, 1H), 3.08 (d, *J* = 25.0 Hz, 3H), 2.91 (dd, *J* = 26.1, 12.3 Hz, 1H), 2.76 (d, *J* = 33.8 Hz, 3H), 2.46 (m, 1H), 2.24 (s, 1H), 2.03 (d, *J* = 4.2 Hz, 1H), 1.98 (s, 3H), 1.46 (d, *J* = 6.7 Hz, 3H), 1.39 (d, *J* = 16.7 Hz, 9H), 1.20 (d, *J* = 6.9 Hz, 3H), 0.98 (d, *J* = 6.3 Hz, 3H), 0.80 – 0.70 (m, 3H). **^{13}C NMR** (100 MHz, $CDCl_3$) δ 175.4, 173.7, 170.1, 169.8, 169.8, 169.7, 169.6, 169.5, 168.6, 168.5, 162.7, 160.2, 155.9, 154.8, 154.2, 133.2, 133.0, 130.4, 130.3, 130.2, 125.1, 115.4, 115.3, 115.1, 114.9, 80.8, 80.4, 71.2, 68.7, 68.6, 60.9, 59.6, 59.0, 58.9, 58.5, 58.0, 56.3, 56.2, 53.4, 53.3, 52.2, 51.8, 36.5, 33.1, 33.0, 31.7, 31.1, 30.3, 28.1, 28.0, 27.0, 25.1, 20.8, 19.0, 18.7, 18.2, 17.4, 17.1, 16.8. **^{19}F NMR** (376 MHz, $CDCl_3$) δ -116.4, -116.6 (rotamer). **HRMS (ESI)** *m/z*: calcd for $C_{37}H_{52}N_5O_{10}FNa^+$ $[M+Na]^+$: 768.3590, found: 768.3582.

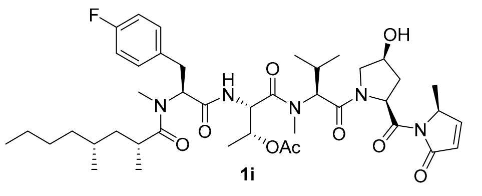

1i

Synthesis of (1i). The titled compound **1i** was obtained following the general procedure described for **1b**. The reaction was purified by column chromatography (100/1 to 20/1 DCM/MeOH) to provide compound **1i** (21 mg, 65%) as a white foamed solid. $[\alpha]_D^{22}$ = −119.7 (*c*

= 0.3, $CHCl_3$). **^{1}H NMR** (400 MHz, $CDCl_3$) δ 7.27 (d, *J* = 2.5 Hz, 1H), 7.14 (dd, *J* = 8.4, 5.3 Hz, 2H), 7.01 (d, *J* = 8.9 Hz, 1H), 6.91 (t, *J* = 8.5 Hz, 2H), 6.07 (dd, *J* = 6.1, 1.6 Hz, 1H), 5.65 (dd, *J* = 10.0, 2.1 Hz, 1H), 5.59 (dd, *J* = 11.1, 5.6 Hz, 1H), 5.30 – 5.20 (m, 1H), 4.99 (d, *J* = 11.1 Hz, 1H), 4.95 (dd, *J* = 9.3, 2.5 Hz, 1H), 4.85 – 4.75 (m, 1H), 4.37 (d, *J* = 9.3 Hz, 1H), 3.89 – 3.77 (m, 2H), 3.53 (d, *J* = 10.9 Hz, 1H), 3.17 (dd, *J* = 15.0, 5.6 Hz, 1H), 3.07 (s, 3H), 2.99 (dd, *J* = 14.9, 10.8 Hz, 1H), 2.91 (s, 3H), 2.68 – 2.59 (m, 1H), 2.46 (m, 1H), 2.26 (m, 1H), 2.02 (d, *J* = 14.8 Hz, 1H), 1.97 (d, *J* = 2.8 Hz, 3H), 1.63 (m, 1H), 1.45 (d, *J* = 6.7 Hz, 3H), 1.26 – 1.20 (m, 4H), 1.15 (d, *J* = 6.5 Hz, 5H), 1.05 (d, *J* = 6.6 Hz, 3H), 0.98 (d, *J* = 6.4 Hz, 3H), 0.87 (t, *J* = 7.0 Hz, 5H), 0.80 (d, *J* = 6.7 Hz, 3H), 0.50 (d, *J* = 6.2 Hz, 3H). **^{13}C NMR** (100 MHz, $CDCl_3$) δ 178.1, 178.1, 174.5, 174.3, 170.5, 170.4, 169.9, 169.9, 169.8, 169.7, 169.7, 169.6, 168.9, 168.9, 162.9, 160.5, 154.3, 132.6, 132.6, 130.4, 130.3, 130.2, 125.4, 115.4, 115.2, 71.8, 68.5, 59.3, 58.7, 58.2, 56.9, 56.4, 52.0, 41.6, 37.1, 36.7, 33.5, 32.5, 31.9, 30.7, 30.4, 30.4, 29.7, 29.7, 29.4, 29.0, 27.2, 23.0, 22.7, 21.1, 19.4, 18.8, 18.5, 18.5, 18.2, 17.4, 17.0, 14.2. **^{19}F NMR** (376 MHz, $CDCl_3$) δ -116.4. **HRMS (ESI)** *m*/*z*: calcd for $C_{42}H_{62}N_5O_9FNa^+$ $[M+Na]^+$: 822.4424, found: 822.4416.

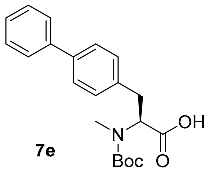

7e

Synthesis of (7e). The titled compound **7e** was obtained following the general procedure described for **27**. The reaction was purified by column chromatography (70/1 to 50/1 DCM/MeOH) to provide compound **7e** (3.10 g, 99%) as a pale yellow oil. $[\alpha]_D^{22}$ = −71.0 (*c* = 0.5, $CHCl_3$). **^{1}H NMR** (400 MHz, $CDCl_3$) δ 10.95 (s, 1H), 7.61 – 7.27 (m, 9H), 4.86 (m, 1H), 3.40 (m, 1H), 3.13 (m, 1H), 2.80 (d, *J* = 28.0 Hz, 3H), 1.39 (d, *J* = 19.9 Hz, 9H). **^{13}C NMR** (100 MHz, $CDCl_3$) δ 176.5, 176.4, 156.4, 155.3, 140.9, 139.8, 139.6, 136.6, 136.3, 129.5, 129.4, 128.9, 127.4, 127.3, 127.3, 127.2, 127.1, 127.1, 81.0, 80.7, 61.5, 60.1, 35.0, 34.5, 32.6, 28.3, 28.2. **HRMS (ESI)** *m*/*z*: calcd for $C_{21}H_{25}NO_4Na^+$ $[M+Na]^+$: 378.1676, found: 378.1678.

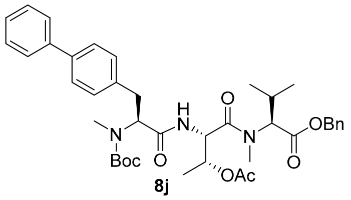

8j

Synthesis of (8j). The titled compound **8j** was obtained following the general procedure described for **8b**. The reaction was purified by column chromatography (30/1 to 5/1 petroleum ether/EtOAc) to provide compound **8j** (1.62 g, 54%) as a colorless oil. $[\alpha]_D^{22}$ = −68.0 (*c* = 0.5, $CHCl_3$). **^{1}H NMR** (400 MHz, $CDCl_3$) δ 7.60 – 7.31 (m, 14H), 6.90 (dd, *J* = 52.7, 8.8 Hz, 1H), 5.30 (dq, *J* = 12.5, 5.9, 5.2 Hz, 1H), 5.18 (s, 2H), 5.04 (p, *J* = 5.7, 5.2 Hz, 1H), 4.92 (p, *J* = 10.6, 9.5 Hz,

2H), 3.37 (ddd, *J* = 19.7, 14.3, 4.8 Hz, 1H), 3.08 (d, *J* = 35.3 Hz, 3H), 2.97 (t, *J* = 6.0 Hz, 0H), 2.82 (d, *J* = 32.0 Hz, 3H), 2.22 (s, 1H), 1.97 (s, 2H), 1.40 (d, *J* = 22.1 Hz, 8H), 1.19 (d, *J* = 6.4 Hz, 3H), 0.99 (dd, *J* = 13.4, 6.5 Hz, 3H), 0.81 (dd, *J* = 20.8, 6.6 Hz, 3H). **^{13}C NMR** (100 MHz, $CDCl_3$) δ 170.4, 170.2, 170.0, 169.9, 169.7, 169.7, 156.1, 155.1, 140.9, 139.7, 139.4, 136.8, 136.6, 135.6, 129.5, 129.4, 128.8, 128.6, 128.6, 128.5, 128.5, 128.4, 127.3, 127.2, 127.1, 127.0, 80.9, 80.6, 69.5, 69.2, 67.0, 66.8, 66.7, 61.9, 60.9, 59.9, 52.9, 52.4, 33.7, 31.3, 29.7, 28.3, 28.2, 27.2, 20.9, 19.8, 19.7, 18.7, 18.7, 16.9, 16.8. **HRMS (ESI)** *m*/*z*: calcd for $C_{40}H_{52}N_3O_8^+$ [M+Na]$^+$: 702.3749, found: 702.3754.

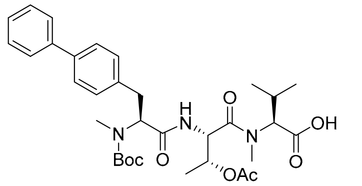

3j

Synthesis of (3j). The titled compound **3j** was obtained following the general procedure described for **3b**. The reaction was purified by column chromatography (3/1 petroleum ether/EtOAc to EtOAc) to provide compound **3j** (1.29 g, 95%) as a white foamed solid. $[\alpha]_D^{22}$ = −51.0 (*c* = 0.5, $CHCl_3$). **^{1}H NMR** (400 MHz, $CDCl_3$) δ 7.58 – 7.29 (m, 9H), 7.07 (d, *J* = 8.8 Hz, 1H, rotamer), 6.91 (d, *J* = 8.5 Hz, 1H, rotamer), 5.32 (s, 1H), 5.07 (s, 1H), 4.98 – 4.84 (m, 1H), 4.74 (dd, *J* = 28.8, 10.2 Hz, 1H), 3.32 (d, *J* = 14.4 Hz, 1H), 3.08 (d, *J* = 30.2 Hz, 3H), 2.91 – 2.66 (m, 4H), 2.21 (s, 1H), 2.00 (s, 3H), 1.37 (d, *J* = 20.8 Hz, 9H), 1.25 (s, 3H), 1.02 (t, *J* = 8.1 Hz, 3H), 0.80 (dd, *J* = 17.5, 6.4 Hz, 3H). **^{13}C NMR** (100 MHz, $CDCl_3$) δ 173.3, 173.1, 170.8, 170.7, 170.0, 169.8, 156.1, 155.1, 137.5, 137.4, 131.0, 129.0, 129.0, 128.9, 128.6, 128.4, 126.7, 126.5, 81.0, 80.8, 80.5, 69.6, 69.4, 65.6, 62.3, 60.9, 60.5, 60.0, 52.8, 52.4, 34.1, 31.9, 31.8, 31.6, 30.6, 30.3, 29.7, 29.7, 28.3, 28.2, 28.1, 26.9, 21.1, 21.0, 19.9, 19.8, 19.2, 19.1, 18.8, 17.4, 17.0, 16.8, 14.2, 13.8. **HRMS (ESI)** *m*/*z*: calcd for $C_{33}H_{45}N_3O_8Na^+$ [M+Na]$^+$: 634.3099, found: 634.3101.

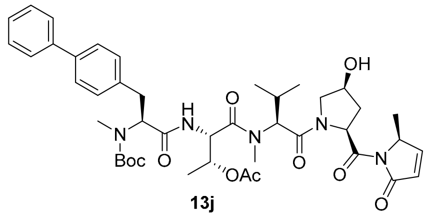

13j

Synthesis of (13j). The titled compound **13j** was obtained following the general procedure described for **13b**. The reaction was purified by column chromatography (100/1 to 20/1 DCM/MeOH) to provide compound **13j** (73 mg, 38%) as a white foamed solid. $[\alpha]_D^{22}$ = −61.8 (*c* = 0.5, $CHCl_3$). **^{1}H NMR** (400 MHz, $CDCl_3$) δ 7.54 (d, *J* = 7.7 Hz, 2H), 7.49 (d, *J* = 7.9 Hz, 2H), 7.41 (t, *J* = 7.6 Hz, 2H), 7.33 (d, *J* = 7.3 Hz, 2H), 7.25 (d, *J* = 2.0 Hz, 2H), 6.91 (d, *J* = 9.0 Hz, 1H, rotamer), 6.75 (d, *J* = 8.6 Hz, 1H, rotamer), 6.08 (dd, *J* = 6.1, 1.6 Hz, 1H), 5.66 (d, *J* = 9.8 Hz, 1H), 5.28 (s, 1H), 5.00 (t, *J* = 5.4 Hz, 2H), 4.80 (qt, *J* = 6.8, 1.8 Hz, 1H), 4.40 – 4.33 (m, 1H), 3.86 (d, *J* = 11.5 Hz,

1H), 3.78 (dd, *J* = 11.5, 4.3 Hz, 1H), 3.55 (d, *J* = 10.9 Hz, 1H), 3.35 (dd, *J* = 14.5, 5.7 Hz, 1H), 3.09 (d, *J* = 26.6 Hz, 3H), 3.01 – 2.92 (m, 1H), 2.80 (d, *J* = 36.2 Hz, 3H), 2.47 (td, *J* = 14.8, 14.3, 4.5 Hz, 1H), 2.31 – 2.19 (m, 1H), 2.03 (s, 1H), 1.99 (s, 3H), 1.45 (d, *J* = 6.7 Hz, 3H), 1.37 (d, *J* = 25.1 Hz, 9H), 1.24 – 1.18 (m, 3H), 0.97 (d, *J* = 8.5 Hz, 3H), 0.76 (dd, *J* = 20.5, 6.5 Hz, 3H). **^{13}C NMR** (100 MHz, $CDCl_3$) δ 174.6, 170.5, 169.9, 169.8, 168.8, 156.2, 155.1, 154.2, 140.9, 139.7, 139.4, 136.8, 136.5, 129.4, 128.7, 127.3, 127.1, 127.0, 125.3, 80.9, 80.5, 71.9, 71.8, 68.9, 68.8, 61.1, 59.8, 59.2, 58.6, 58.2, 58.1, 56.9, 52.4, 52.0, 36.6, 33.7, 31.9, 31.4, 30.5, 29.7, 29.7, 29.4, 28.3, 28.2, 27.2, 22.7, 21.0, 18.8, 18.4, 17.6, 17.4, 17.3, 16.9, 14.1. **HRMS (ESI)** *m/z*: calcd for $C_{43}H_{57}N_5O_{10}Na^+$ $[M+Na]^+$: 826.3998, found: 826.4002.

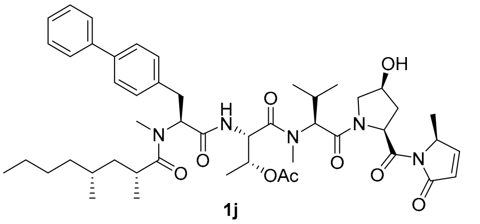

1j

Synthesis of (1j). The titled compound **1j** was obtained following the general procedure described for **1b**. The reaction was purified by column chromatography (100/1 to 20/1 DCM/MeOH) to provide compound **1j** (21 mg, 62%) as a white foamed solid. $[\boldsymbol{\alpha}]_{\boldsymbol{D}}^{\mathbf{22}}$ = −102.0 (*c* = 0.2, $CHCl_3$). **^{1}H NMR** (400 MHz, $CDCl_3$) δ 7.55 (d, *J* = 7.8 Hz, 3H), 7.48 (dd, *J* = 8.1, 1.8 Hz, 2H), 7.41 (t, *J* = 7.5 Hz, 2H), 7.34 – 7.27 (m, 3H), 7.08 (d, *J* = 8.9 Hz, 1H), 6.08 (d, *J* = 6.0 Hz, 1H), 5.68 (t, *J* = 11.1 Hz, 2H), 5.27 (q, *J* = 7.5, 7.1 Hz, 1H), 5.05 – 4.94 (m, 2H), 4.84 – 4.77 (m, 1H), 4.44 – 4.35 (m, 1H), 3.91 – 3.78 (m, 2H), 3.52 (d, *J* = 11.0 Hz, 1H), 3.26 (dd, *J* = 15.2, 5.5 Hz, 1H), 3.14 – 3.04 (m, 4H), 2.96 (d, *J* = 1.8 Hz, 3H), 2.70 – 2.61 (m, 1H), 2.47 (ddd, *J* = 14.8, 10.1, 4.7 Hz, 1H), 2.25 (dp, *J* = 12.8, 6.5 Hz, 1H), 2.03 (d, *J* = 14.6 Hz, 1H), 1.99 (d, *J* = 1.7 Hz, 3H), 1.80 – 1.62 (m, 3H), 1.47 (d, *J* = 6.7 Hz, 3H), 1.18 (d, *J* = 6.5 Hz, 3H), 1.07 (d, *J* = 6.4 Hz, 5H), 0.99 (d, *J* = 6.5 Hz, 3H), 0.87 (q, *J* = 7.3, 5.3 Hz, 3H), 0.83 – 0.75 (m, 6H), 0.43 (d, *J* = 4.4 Hz, 3H). **^{13}C NMR** (100 MHz, $CDCl_3$) δ 178.1, 174.9, 170.8, 170.0, 169.9, 169.7, 169.1, 154.3, 140.8, 139.6, 136.1, 131.1, 129.3, 128.9, 127.2, 127.0, 125.5, 72.1, 68.5, 65.7, 59.3, 58.8, 58.2, 57.2, 56.3, 52.1, 41.8, 37.4, 36.8, 33.6, 33.0, 30.8, 30.5, 29.2, 27.3, 23.1, 21.2, 19.4, 19.0, 18.7, 18.3, 17.6, 17.1, 14.2. **HRMS (ESI)** *m/z*: calcd for $C_{48}H_{67}N_5O_9Na^+$ $[M+Na]^+$: 880.4831, found: 880.4833.

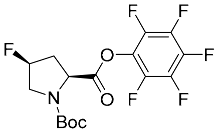

10b

Synthesis of (10b). To a solution of **9b** (5.0 g, 21.44 mmol) and pentafluorophenol (4.34 g, 23.58 mmol) in EtOAc (80 mL) was added DCC (4.87 g, 23.58 mmol). The reaction was stirred at room temperature for 4 h. The mixture was filtered through a pad of celite, and concentrated,

diluted with EtOAc (200 mL), washed with K_2CO_3 (15% aqueous, 3 × 100 mL) and brine (100 mL), dried (Na_2SO_4), filtered, concentrated in vacuo and purified by column chromatography (40/1 to 20/1 petroleum ether/EtOAc) to provide compound **10b** (8.39 g, 98%) as a white semi-solid. $[\alpha]_D^{25}$ = −31.0 (*c* = 0.5, $CHCl_3$). **^{1}H NMR** (400 MHz, $CDCl_3$) δ 5.40 – 5.33 (m, 1H, rotamer), 5.23 (t, *J* = 3.9 Hz, 1H, rotamer), 4.87 (dd, *J* = 9.8, 1.4 Hz, 1H, minor rotamer), 4.78 (dd, *J* = 9.7, 1.4 Hz, 1H, major rotamer), 3.91 (dtd, *J* = 27.3, 14.1, 13.7, 1.7 Hz, 1H), 3.81 – 3.62 (m, 1H), 2.78 – 2.64 (m, 1H), 2.64 – 2.42 (m, 1H), 1.48 (d, *J* = 8.8 Hz, 9H). **^{13}C NMR** (100 MHz, $CDCl_3$) δ 168.1, 167.7, 153.9, 153.4, 142.5, 141.1, 140.3, 139.2, 136.8, 92.9, 91.9, 91.2, 90.1, 81.4, 81.2, 77.5, 77.4, 77.2, 76.8, 57.4, 57.2, 53.5, 53.3, 53.0, 38.0, 37.8, 37.0, 36.8, 28.4, 28.2. **^{19}F NMR** (376 MHz, $CDCl_3$) δ -151.8, -151.8, -151.9, -151.9, -151.9, -151.9, -152.6, -152.6, -152.7, -152.7, -152.7, -152.7, -152.7, -152.7, -157.5, -157.5, -157.6, -157.9, -158.0, -158.0, -162.0, -162.0, -162.0, -162.0, -162.0, -162.0, -162.1, -162.1, -162.1, -162.1, -162.3, -162.4, -162.4, -162.4, -162.5, -162.5, -172.7, -172.8, -172.8, -172.8. **HRMS (ESI)** *m/z*: calcd for $C_{16}H_{15}F_6NO_4Na^+$ [M+Na]$^+$: 422.0797, measured: 422.0796.

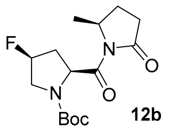
12b

Synthesis of (12b). To the solution of **11a** (2.0 g, 20.2 mmol) in THF (20 mL) was added *n*-BuLi (2.4 M, 8.8 mL, 21.2 mmol) dropwise at −78 °C under argon. After the mixture had been stirred for 1 h at −78 °C, compound **10b** (7.39 g, 20.2 mmol) in THF (20 mL) was added dropwise at −78 °C. After the mixture had been stirred for 4 h at −78 °C, the reaction was quenched with saturated aqueous NH_4Cl (50 mL). The aqueous phase was extracted with EtOAc (3 × 50 mL) and the combined organics were washed with brine (150 mL), dried (Na_2SO_4), filtered, concentrated in vacuo and purified by column chromatography (30/1 to 10/1 petroleum ether/EtOAc) to provide compound **12b** (5.20 g, 82%) as a white semi-solid. $[\alpha]_D^{25}$ = −36.8 (*c* = 1.0, $CHCl_3$). **^{1}H NMR** (400 MHz, $CDCl_3$) δ 5.34 – 5.08 (m, 2H), 4.57 – 4.41 (m, 1H), 3.91 (dd, *J* = 27.6, 13.3 Hz, 1H), 3.74 (dddd, *J* = 33.2, 13.1, 9.6, 4.7 Hz, 1H), 2.84 – 2.63 (m, 1H), 2.64 – 2.41 (m, 2H), 2.26 (tdd, *J* = 21.1, 12.3, 4.7 Hz, 2H), 1.76 (dtd, *J* = 12.7, 9.1, 1.8 Hz, 1H), 1.44 (d, *J* = 27.2 Hz, 9H), 1.35 (d, *J* = 6.6 Hz, 3H). **^{13}C NMR** (100 MHz, $CDCl_3$) δ 175.4, 175.4, 171.5, 171.5, 153.8, 153.6, 93.1, 92.1, 91.4, 90.4, 80.0, 79.9, 60.1, 60.0, 53.9, 53.7, 53.6, 53.5, 53.4, 38.0, 37.8, 37.1, 36.9, 31.7, 28.3, 28.2, 28.1, 25.4, 25.3, 19.3, 19.1. **^{19}F NMR** (376 MHz, $CDCl_3$) δ -169.5, -169.6, -169.6, -169.6, -169.6, -169.7, -169.7, -169.7, -169.7, -170.0, -170.0, -170.0, -170.0, -170.1, -170.1, -170.1, -170.1, -170.1, -170.1, -170.1, -170.2, -170.2, -170.2, -170.2, -170.2, -170.2, -170.3, -170.3, -170.3, -170.3, -170.3, -170.4, -170.4, -170.4, -170.4, -170.4, -170.5. **HRMS (ESI)** *m/z*: calcd for $C_{15}H_{23}FN_2O_4Na^+$ [M+Na]$^+$: 337.1534, found: 337.1533.

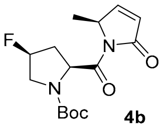

4b

Synthesis of (4b). To the solution of **12b** (5.0 g,15.9 mmol) in THF (30 mL) was added LiHMDS (1.0 M, 23.9 mL, 23.9 mmol) dropwise at −78 °C under argon. After the mixture had been stirred for 1 h at −78 °C, HMPA (11.1 mL, 63.6 mmol) was added at −78 °C. After the mixture had been stirred for 30 min at −78 °C, PhSeBr (5.63 g, 23.9 mmol) in THF (20 mL) was added dropwise at −78 °C. After the mixture had been stirred for 3 h at −78 °C, the reaction was warmed to 0 °C. After 30 min stirred at 0 °C, 30% H_2O_2 (9.8 M, 8.1 mL, 79.5 mmol) was added. After stirred 30 min at 0 °C, quenched with saturated aqueous $Na_2O_3S_2$ (50 mL). The aqueous phase was extracted with EtOAc (3 × 50 mL) and the combined organics were washed with brine (100 mL), dried (Na_2SO_4), filtered, concentrated in vacuo and purified by column chromatography (15/1 to 3/1 petroleum ether/EtOAc) to provide compound **4b** (2.03 g, 41%) as a pale yellow solid. $[\boldsymbol{\alpha}]_D^{25}$ = −15.1 (*c* = 1.0, $CHCl_3$). **^{1}H NMR** (400 MHz, $CDCl_3$) *δ* 7.28 (dd, *J* = 6.1, 2.0 Hz, 1H), 6.07 (ddd, *J* = 14.5, 6.0, 1.6 Hz, 1H), 5.39 – 5.12 (m, 2H), 4.87 – 4.74 (m, 1H), 4.02 – 3.69 (m, 2H), 2.71 – 2.51 (m, 1H), 2.40 – 2.18 (m, 1H), 1.50 (d, *J* = 6.7 Hz, 3H), 1.43 (d, *J* = 29.5 Hz, 9H). **^{13}C NMR** (100 MHz, $CDCl_3$) *δ* 170.9, 170.1, 154.6, 154.5, 154.0, 153.7, 125.4, 125.3, 93.3, 92.3, 91.5, 90.5, 80.2, 80.1, 59.6, 59.5, 58.4, 58.3, 54.1, 53.9, 53.9, 53.9, 53.7, 37.9, 37.6, 36.9, 36.7, 28.4, 28.3, 28.3, 28.2, 17.9, 17.4. **^{19}F NMR** (376 MHz, $CDCl_3$) *δ* -169.9, -170.0, -170.0, -170.0, -170.0, -170.0, -170.0, -170.1, -170.1, -170.1, -170.1, -170.1, -170.1, -170.1, -170.2, -170.2, -170.2, -170.2, -170.2, -170.2, -170.3, -170.3, -170.3, -170.3, -170.3, -170.3, -170.3, -170.3, -170.4, -170.4. **HRMS (ESI)** *m*/*z*: calcd for $C_{15}H_{21}N_2O_4FNa^+$ $[M+Na]^+$: 335.1378, measured: 335.1377.

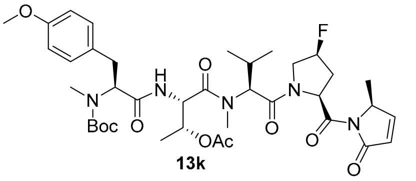

13k

Synthesis of (13k). The titled compound **13k** was obtained following the general procedure described for **13b**. The reaction was purified by column chromatography (100/1 to 20/1 DCM/MeOH) to provide compound **13k** (705 mg, 58%) as a white foamed solid. $[\boldsymbol{\alpha}]_D^{25}$ = −76.3 (*c* = 1.0, $CHCl_3$). **^{1}H NMR** (400 MHz, $CDCl_3$) *δ* 7.25 (d, *J* = 2.5 Hz, 1H), 7.10 (s, 2H), 6.90 – 6.68 (m, 3H), 6.06 (d, *J* = 4.6 Hz, 1H), 5.56 (d, *J* = 9.9 Hz, 1H), 5.22 (d, *J* = 48.7 Hz, 2H), 4.97 (t, *J* = 13.6 Hz, 2H), 4.82 – 4.72 (m, 2H), 4.18 – 3.92 (m, 2H), 3.77 (s, 3H), 3.25 (d, *J* = 14.3 Hz, 1H), 3.10 (d, *J* = 25.8 Hz, 3H), 2.92 (d, *J* = 20.1 Hz, 1H), 2.76 (d, *J* = 32.6 Hz, 3H), 2.68 – 2.54 (m, 1H), 2.38 – 2.24 (m, 2H), 1.98 (s, 3H), 1.48 (d, *J* = 6.8 Hz, 3H), 1.39 (d, *J* = 18.9 Hz, 9H), 1.24 – 1.20 (m, 3H), 1.06 (d, *J* = 6.4 Hz, 3H), 0.83 – 0.73 (m, 3H). **^{13}C NMR** (100 MHz, $CDCl_3$) *δ* 170.2, 170.1, 169.6,

169.6, 169.6, 169.4, 169.4, 169.2, 169.1, 168.9, 168.2, 168.1, 158.0, 157.9, 155.8, 154.7, 154.3, 129.9, 129.6, 129.6, 129.4, 129.3, 129.1, 129.0, 124.8, 113.6, 113.4, 113.3, 92.5, 90.8, 80.3, 79.9, 68.2, 59.7, 59.6, 59.1, 58.8, 57.9, 57.8, 54.8, 54.0, 53.8, 51.9, 51.6, 35.5, 35.3, 32.8, 32.7, 31.5, 31.0, 30.3, 30.1, 30.0, 29.2, 29.2, 28.9, 27.9, 27.8, 27.8, 27.7, 26.8, 22.3, 20.6, 20.6, 19.6, 18.5, 18.1, 17.6, 17.4, 17.3, 17.0, 17.0, 16.8, 16.8, 16.7, 16.6, 16.5, 13.8. **^{19}F NMR** (376 MHz, $CDCl_3$) δ -170.4, -170.5, -170.6, -170.7, -170.8, -170.8. **HRMS (ESI)** *m/z*: calcd for $C_{38}H_{54}N_5O_{10}FNa^+$ $[M+Na]^+$: 782.3747, found: 782.3744.

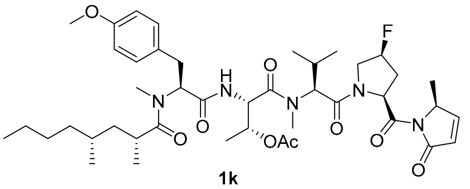

1k

Synthesis of (1k). The titled compound **1k** was obtained following the general procedure described for **1b**. The reaction was purified by column chromatography (100/1 to 20/1 DCM/MeOH) to provide compound **1k** (34 mg, 40%) as a white foamed solid. $[\alpha]_D^{25}$ = −88.0 (*c* = 1.0, $CHCl_3$) **^{1}H NMR** (400 MHz, $CDCl_3$) δ 7.24 (d, *J* = 2.0 Hz, 1H), 7.09 (d, *J* = 8.6 Hz, 2H), 7.03 (d, *J* = 8.9 Hz, 1H), 6.76 (d, *J* = 8.6 Hz, 2H), 6.05 (dd, *J* = 6.0, 1.6 Hz, 1H), 5.57 (ddd, *J* = 10.2, 7.1, 4.0 Hz, 2H), 5.33 – 5.12 (m, 2H), 5.01 – 4.90 (m, 2H), 4.77 (qt, *J* = 6.8, 1.8 Hz, 1H), 4.14 (ddd, *J* = 31.7, 12.6, 4.9 Hz, 1H), 3.99 (dd, *J* = 26.1, 12.6 Hz, 1H), 3.75 (s, 3H), 3.20 – 3.10 (m, 1H), 3.10 (s, 3H), 3.02 – 2.96 (m, 1H), 2.92 (s, 3H), 2.77 – 2.53 (m, 2H), 2.38 – 2.20 (m, 2H), 1.97 (s, 3H), 1.65 (t, *J* = 9.2 Hz, 1H), 1.48 (d, *J* = 6.7 Hz, 3H), 1.24 (s, 4H), 1.16 (d, *J* = 6.5 Hz, 5H), 1.07 (d, *J* = 3.6 Hz, 3H), 1.05 (d, *J* = 3.9 Hz, 3H), 0.86 (d, *J* = 5.4 Hz, 4H), 0.82 (d, *J* = 6.6 Hz, 4H), 0.50 (d, *J* = 5.8 Hz, 3H). **^{13}C NMR** (100 MHz, $CDCl_3$) δ 177.9, 170.7, 169.9, 169.7, 169.6, 169.5, 168.6, 158.2, 154.4, 129.6, 129.6, 129.5, 128.6, 125.2, 113.8, 92.9, 91.1, 68.2, 59.4, 59.1, 58.2, 56.4, 55.0, 55.0, 54.4, 54.2, 51.8, 41.5, 37.2, 35.9, 35.7, 33.4, 32.3, 30.5, 30.3, 30.2, 29.6, 29.2, 28.9, 28.9, 27.1, 23.0, 20.9, 19.3, 18.8, 18.5, 18.1, 17.5, 17.3, 14.1. **^{19}F NMR** (376 MHz, $CDCl_3$) δ -170.2, -170.2, -170.3, -170.3, -170.3, -170.3, -170.3, -170.3, -170.4, -170.4, -170.4, -170.4, -170.4, -170.4, -170.4, -170.4, -170.5, -170.5, -170.5, -170.5, -170.5, -170.6, -170.6, -170.6, -170.6. **HRMS (ESI)** *m/z*: calcd for $C_{48}H_{64}N_5O_9FNa^+$ $[M+Na]^+$: 836.4580, found: 836.4578.

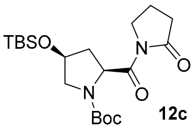

12c

Synthesis of (12c). The titled compound **12c** was obtained following the general procedure described for **12b**. The reaction was purified by column chromatography (30/1 to 10/1 petroleum ether/EtOAc) to provide compound **12c** (9.83 g, 72%) as a pale yellow semi-solid. $[\alpha]_D^{22}$ = −65.2 (*c* = 0.5, $CHCl_3$). **^{1}H NMR** (400 MHz, $CDCl_3$) δ 5.21 (m, 1H), 4.32 (m, 1H), 3.93 –

3.64 (m, 3H), 3.29 (m, 1H), 2.57 (m, 3H), 2.14 – 1.95 (m, 2H), 1.85 – 1.74 (m, 1H), 1.40 (d, *J* = 35.3 Hz, 9H), 0.83 (d, *J* = 2.0 Hz, 9H), 0.04 – -0.02 (m, 6H). **^{13}C NMR** (100 MHz, $CDCl_3$) *δ* 175.4, 175.4, 172.9, 172.3, 154.4, 153.8, 80.0, 79.8, 70.4, 69.6, 59.3, 59.2, 54.8, 54.1, 49.3, 45.8, 45.7, 39.6, 39.0, 34.0, 33.7, 33.6, 28.6, 28.4, 25.7, 25.0, 18.0, 18.0, 17.8, 17.7, -4.8, -4.8, -4.9. **HRMS (ESI)** *m*/*z*: calcd for $C_{20}H_{36}N_2O_5SiNa^+$ [M+Na]$^+$: 435.2286, found: 435.2281.

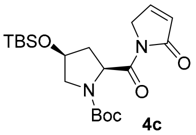

4c

Synthesis of (4c). The titled compound **4c** was obtained following the general procedure described for **4b**. The reaction was purified by column chromatography (15/1 to 3/1 petroleum ether/EtOAc) to provide compound **4c** (1.40 g, 35%) as a pale yellow solid. $[\alpha]_D^{22}$ = −62.0 (*c* = 0.5, $CHCl_3$). **^{1}H NMR** (400 MHz, $CDCl_3$) *δ* 7.31 (dd, *J* = 20.3, 6.3 Hz, 1H), 6.20 – 6.05 (m, 1H), 5.31 – 5.13 (m, 1H), 4.61 – 4.22 (m, 3H), 3.72 (m, 1H), 3.29 (m, 1H), 2.59 (m, 1H), 1.83 (m, 1H), 1.43 (s, 9H, rotamer), 1.32 (s, 9H, rotamer), 0.80 (d, *J* = 1.6 Hz, 9H), -0.00 (d, *J* = 13.1 Hz, 6H). **^{13}C NMR** (100 MHz, $CDCl_3$) *δ* 171.9, 171.3, 169.8, 169.8, 154.3, 153.7, 148.2, 147.3, 147.3, 128.4, 127.6, 127.5, 82.4, 82.1, 82.0, 80.1, 79.9, 79.7, 70.3, 69.6, 58.9, 58.7, 54.8, 54.0, 54.0, 50.9, 50.9, 39.4, 38.8, 28.5, 28.5, 28.3, 28.3, 25.7, 25.7, 25.6, 17.9, 17.9, -4.8, -4.8, -4.9, -4.9. **HRMS (ESI)** *m*/*z*: calcd for $C_{20}H_{34}N_2O_5SiNa^+$ [M+Na]$^+$: 433.2129, measured: 433.2123.

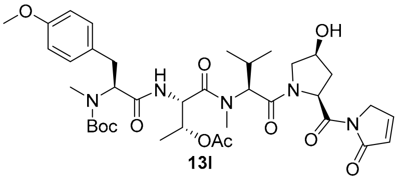

13l

Synthesis of (13l). The titled compound **13l** was obtained following the general procedure described for **13b**. The reaction was purified by column chromatography (100/1 to 20/1 DCM/MeOH) to provide compound **13l** (520 mg, 34%) as a white foamed solid. $[\alpha]_D^{22}$ = −101.8 (*c* = 0.5, $CHCl_3$). **^{1}H NMR** (400 MHz, $CDCl_3$) *δ* 7.35 (d, *J* = 6.2 Hz, 1H), 7.08 (d, *J* = 11.3 Hz, 2H), 6.89 – 6.66 (m, 3H), 6.18 (d, *J* = 6.2 Hz, 1H), 5.73 (d, *J* = 9.9 Hz, 1H), 5.28 (d, *J* = 10.3 Hz, 1H), 4.99 (s, 2H), 4.77 (d, *J* = 24.2 Hz, 1H), 4.54 – 4.33 (m, 3H), 3.84 (s, 2H), 3.76 (s, 3H), 3.28 (d, *J* = 10.6 Hz, 1H), 3.22 (d, *J* = 5.6 Hz, 1H), 3.09 (d, *J* = 29.8 Hz, 3H), 2.91 (m, 1H), 2.76 (d, *J* = 32.6 Hz, 3H), 2.50 (m, 1H), 2.24 (s, 1H), 2.07 – 2.01 (m, 1H), 1.98 (s, 3H), 1.39 (d, *J* = 18.5 Hz, 9H), 1.23 – 1.17 (m, 3H), 1.01 (d, *J* = 6.3 Hz, 3H), 0.78 (m, 3H). **^{13}C NMR** (100 MHz, $CDCl_3$) *δ* 173.2, 170.5, 170.0, 169.8, 169.8, 169.7, 169.7, 169.0, 168.9, 158.2, 156.1, 155.1, 147.7, 129.9, 129.8, 129.5, 129.3, 127.3, 113.9, 113.8, 80.8, 80.7, 80.4, 71.2, 68.8, 68.7, 61.2, 59.9, 59.1, 58.3, 56.4, 55.2, 52.3, 51.9, 50.7, 36.8, 33.2, 33.0, 31.3, 30.4, 29.7, 29.6, 29.6, 28.2, 28.1, 27.2, 25.5, 22.6, 21.0, 18.9, 18.5, 17.5, 17.2, 14.1. **HRMS (ESI)** *m*/*z*: calcd for $C_{37}H_{53}N_5O_{11}Na^+$ [M+Na]$^+$: 766.3634, found: 766.3628.

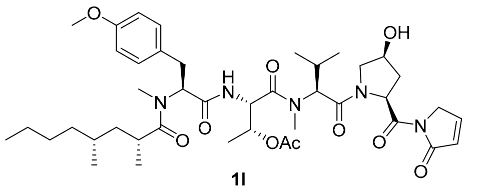

1l

Synthesis of (1l). The titled compound **1l** was obtained following the general procedure described for **1b**. The reaction was purified by column chromatography (100/1 to 20/1 DCM/MeOH) to provide compound **1l** (34 mg, 40%) as a white foamed solid. $[\alpha]_D^{22}$ = −108.8 (*c* = 0.5, $CHCl_3$). **^{1}H NMR** (400 MHz, $CDCl_3$) δ 7.35 (d, *J* = 5.9 Hz, 1H), 7.10 (d, *J* = 8.6 Hz, 2H), 7.05 (d, *J* = 9.0 Hz, 1H), 6.77 (d, *J* = 8.0 Hz, 2H), 6.18 (d, *J* = 5.9 Hz, 1H), 5.73 (dd, *J* = 10.0, 2.5 Hz, 1H), 5.58 (dd, *J* = 11.1, 5.6 Hz, 1H), 5.26 (dd, *J* = 6.6, 2.8 Hz, 1H), 5.04 – 4.93 (m, 2H), 4.56 – 4.36 (m, 3H), 3.90 (dd, *J* = 11.5, 4.6 Hz, 1H), 3.82 (d, *J* = 11.5 Hz, 1H), 3.76 (s, 3H), 3.36 (d, *J* = 10.1 Hz, 1H), 3.20 – 3.10 (m, 1H), 3.09 (s, 3H), 3.03 – 2.93 (m, 1H), 2.93 (s, 3H), 2.65 (m, 1H), 2.52 (m, 1H), 2.30 – 2.20 (m, 1H), 2.04 (s, 1H), 1.98 (s, 3H), 1.65 (t, *J* = 9.3 Hz, 1H), 1.26 (s, 6H), 1.17 (d, *J* = 6.5 Hz, 5H), 1.06 (d, *J* = 6.6 Hz, 3H), 1.03 (d, *J* = 6.5 Hz, 3H), 0.85 – 0.80 (m, 6H), 0.51 (d, *J* = 5.6 Hz, 3H). **^{13}C NMR** (100 MHz, $CDCl_3$) δ 178.0, 173.9, 170.8, 169.8, 169.8, 169.2, 158.4, 147.7, 129.8, 129.8, 128.8, 127.4, 113.9, 113.9, 71.7, 68.5, 59.2, 58.4, 56.9, 56.5, 55.2, 52.0, 50.8, 41.7, 37.3, 36.9, 33.5, 32.4, 32.0, 30.7, 30.5, 30.4, 30.1, 29.8, 29.8, 29.7, 29.1, 27.3, 23.1, 22.8, 21.1, 19.5, 19.0, 18.7, 18.2, 17.5, 14.2, 14.2. **HRMS (ESI)** *m*/*z*: calcd for $C_{42}H_{63}N_5O_{10}Na^+$ $[M+Na]^+$: 820.4467, found: 820.4468.

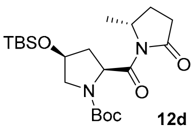

12d

Synthesis of (12d). The titled compound **12d** was obtained following the general procedure described for **12b**. The reaction was purified by column chromatography (30/1 to 15/1 petroleum ether/EtOAc) to provide compound **12d** (9.44 g, 85%) as a pale yellow oil. $[\alpha]_D^{25}$ = −94.2 (*c* = 1.0, $CHCl_3$). **^{1}H NMR** (400 MHz, $CDCl_3$) δ 5.18 (ddd, *J* = 12.3, 8.8, 5.9 Hz, 1H), 4.48 (dddd, *J* = 10.6, 8.3, 6.3, 1.9 Hz, 1H), 4.31 (p, *J* = 5.8 Hz, 1H), 3.72 (ddd, *J* = 35.4, 10.9, 6.3 Hz, 1H), 3.27 (dt, *J* = 10.6, 5.0 Hz, 1H), 2.74 – 2.53 (m, 2H), 2.46 (dddd, *J* = 18.1, 15.7, 9.4, 2.6 Hz, 1H), 2.28 – 2.09 (m, 1H), 1.69 (qt, *J* = 12.5, 8.0 Hz, 2H), 1.38 (d, *J* = 37.6 Hz, 9H), 1.30 – 1.21 (m, 3H), 0.82 (d, *J* = 2.3 Hz, 9H), 0.03 – -0.02 (m, 6H). **^{13}C NMR** (100 MHz, $CDCl_3$) δ 175.4, 175.3, 172.6, 171.8, 154.3, 153.7, 79.9, 79.7, 70.4, 69.7, 59.2, 59.0, 54.9, 54.1, 53.4, 53.2, 39.2, 38.8, 31.8, 31.8, 29.8, 28.6, 28.3, 25.9, 25.6, 25.3, 19.8, 19.7, 18.1, -4.7, -4.9. **HRMS (ESI)** *m*/*z*: calcd for $C_{21}H_{38}N_2O_5SiNa^+$ $[M+Na]^+$: 449.2442, measured: 449.2437.

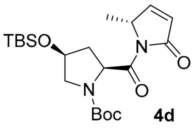

4d

Synthesis of (4d). The titled compound **4d** was obtained following the general procedure described for **4b**. The reaction was purified by column chromatography (10/1 to 5/1 petroleum ether/EtOAc) to provide compound **4d** (2.60 g, 29%) as a white solid. $[\alpha]_D^{25}$ = −25.1 (*c* = 1.0, $CHCl_3$). **¹H NMR** (400 MHz, $CDCl_3$) δ 7.26 – 7.19 (m, 1H), 6.05 (ddd, *J* = 15.8, 6.0, 1.6 Hz, 1H), 5.35 – 5.26 (m, 1H), 4.89 – 4.76 (m, 1H), 4.43 – 4.30 (m, 1H), 3.76 (ddd, *J* = 35.6, 11.0, 6.1 Hz, 1H), 3.32 (dt, *J* = 10.8, 5.2 Hz, 1H), 2.63 (ddt, *J* = 13.4, 8.9, 6.5 Hz, 1H), 1.83 (ddt, *J* = 13.2, 7.4, 5.4 Hz, 1H), 1.42 (t, *J* = 6.8 Hz, 3H), 1.40 (d, *J* = 49.3 Hz, 9H), 0.84 (d, *J* = 1.8 Hz, 9H), 0.11 – 0.00 (m, 6H). **¹³C NMR** (100 MHz, $CDCl_3$) δ 171.5, 170.8, 169.8, 169.8, 154.3, 154.2, 154.2, 153.8, 125.5, 79.9, 79.7, 70.6, 69.8, 59.4, 59.1, 58.1, 58.0, 55.0, 54.2, 39.6, 39.1, 28.6, 28.4, 25.9, 18.2, 17.9, 17.8, -4.7, -4.7, -4.8. **HRMS (ESI)** *m/z*: calcd for $C_{21}H_{36}N_2O_5SiNa^+$ $[M+Na]^+$: 447.2286, measured: 447.2282.

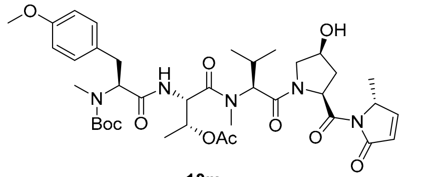

13m

Synthesis of (13m). The titled compound **13m** was obtained following the general procedure described for **13b**. The reaction was purified by column chromatography (3/1 to 1/1 DCM/EtOAc) to provide compound **13m** (228 mg, 71%) as a white foamed solid. $[\alpha]_D^{25}$ = −106.5 (*c* = 1.0, $CHCl_3$). **¹H NMR** (400 MHz, $CDCl_3$) δ 7.25 (d, *J* = 7.7 Hz, 1H), 7.11 – 6.97 (m, 2H), 6.90 – 6.62 (m, 3H), 6.06 (d, *J* = 5.9 Hz, 1H), 5.73 (d, *J* = 9.7 Hz, 1H), 5.24 (s, 1H), 4.96 (d, *J* = 9.0 Hz, 2H), 4.85 – 4.70 (m, 2H), 4.36 (s, 1H), 3.89 – 3.77 (m, 2H), 3.73 (d, *J* = 2.4 Hz, 3H), 3.31 (d, *J* = 9.6 Hz, 1H), 3.25 – 3.17 (m, 1H), 3.05 (d, *J* = 30.1 Hz, 3H), 2.90 (dt, *J* = 14.9, 7.0 Hz, 1H), 2.73 (d, *J* = 28.7 Hz, 3H), 2.47 (ddd, *J* = 14.6, 10.1, 4.7 Hz, 1H), 2.20 (s, 1H), 1.94 (d, *J* = 10.7 Hz, 4H), 1.36 (d, *J* = 19.8 Hz, 9H), 1.20 (d, *J* = 16.6 Hz, 3H), 0.99 (d, *J* = 6.3 Hz, 3H), 0.88 – 0.69 (m, 6H). **¹³C NMR** (100 MHz, $CDCl_3$) δ 173.3, 170.6, 169.9, 169.9, 169.8, 169.7, 169.0, 158.3, 156.2, 155.2, 154.6, 130.0, 129.9, 129.7, 129.6, 129.4, 125.3, 114.0, 113.9, 80.9, 80.5, 71.5, 68.9, 68.8, 61.3, 60.4, 60.1, 59.3, 58.7, 58.1, 58.0, 56.6, 55.3, 52.3, 51.9, 37.2, 37.1, 33.2, 32.0, 31.4, 30.5, 30.2, 30.1, 29.8, 29.7, 29.7, 29.4, 28.3, 28.3, 27.3, 27.1, 22.7, 21.2, 21.1, 19.1, 18.5, 18.3, 18.0, 17.7, 17.3, 17.0, 14.2. **HRMS (ESI)** *m/z*: calcd for $C_{38}H_{55}N_5O_{11}Na^+$ $[M+Na]^+$: 780.3790, measured: 780.3793.

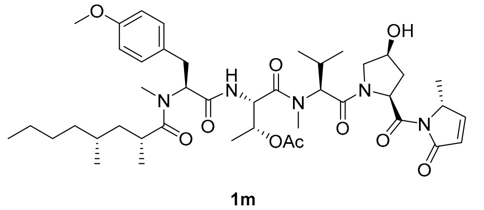

1m

Synthesis of (1m). The titled compound **1m** was obtained following the general procedure described for **1b**. The reaction was purified by column chromatography (2/1 to 1/1 DCM/EtOAc)

to provide compound **1m** (120 mg, 37%) as a white foamed solid. $[\alpha]_D^{25}$ = −85.2 (c = 1.0, $CHCl_3$). ^{1}**H NMR** (400 MHz, $CDCl_3$) δ 7.26 (d, J = 8.2 Hz, 1H), 7.08 (d, J = 8.7 Hz, 2H), 7.02 (d, J = 8.8 Hz, 1H), 6.75 (d, J = 8.6 Hz, 2H), 6.07 (d, J = 6.1 Hz, 1H), 5.76 (dd, J = 10.1, 2.3 Hz, 1H), 5.56 (dd, J = 11.2, 5.6 Hz, 1H), 5.24 (qd, J = 6.5, 2.5 Hz, 1H), 5.02 – 4.91 (m, 2H), 4.88 – 4.76 (m, 1H), 4.43 – 4.34 (m, 1H), 3.92 – 3.76 (m, 2H), 3.74 (s, 3H), 3.28 (d, J = 9.9 Hz, 1H), 3.13 (dd, J = 14.2, 4.7 Hz, 1H), 3.07 (s, 3H), 2.97 (d, J = 12.0 Hz, 1H), 2.91 (s, 3H), 2.67 – 2.58 (m, 1H), 2.48 (ddd, J = 14.5, 10.1, 4.8 Hz, 1H), 2.28 – 2.18 (m, 1H), 1.98 (d, J = 8.6 Hz, 4H), 1.63 (t, J = 9.4 Hz, 1H), 1.42 (d, J = 6.7 Hz, 3H), 1.25 – 1.19 (m, 4H), 1.15 (d, J = 6.6 Hz, 5H), 1.04 (d, J = 6.7 Hz, 3H), 1.01 (d, J = 6.5 Hz, 3H), 0.86 (t, J = 7.1 Hz, 4H), 0.80 (d, J = 6.6 Hz, 4H), 0.48 (d, J = 5.8 Hz, 3H). ^{13}**C NMR** (100 MHz, $CDCl_3$) δ 178.0, 173.5, 170.8, 169.9, 169.8, 169.7, 169.2, 158.4, 154.6, 129.8, 129.8, 128.8, 125.4, 113.9, 71.7, 68.6, 66.3, 65.0, 59.3, 58.7, 58.1, 56.8, 56.5, 55.2, 52.0, 43.4, 41.7, 37.3, 37.2, 33.5, 32.5, 30.7, 30.6, 30.5, 30.4, 29.8, 29.1, 27.3, 23.1, 21.1, 19.5, 19.1, 19.1, 18.7, 18.2, 17.8, 17.5, 14.2, 14.2, 13.7. **HRMS (ESI)** m/z: calcd for $C_{43}H_{65}N_5O_{10}Na^+$ $[M+Na]^+$: 834.4624, measured: 834.4621.

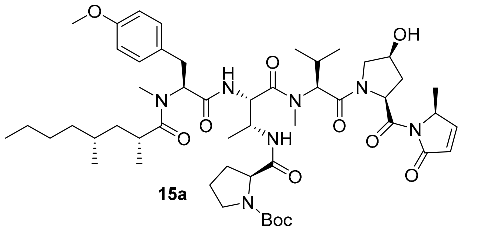

15a

Synthesis of (15a). To the solution of **1f** (260 mg, 0.262 mmol) in DCM (2.6 mL) was added Et_2NH (1.3 mL) at 0 °C. The reaction was allowed to reach room temperature and stirred 4 h. The mixture was quenched with saturated aqueous NH_4Cl (5 mL). The aqueous phase was extracted with EtOAc (3 × 5 mL) and the combined organics were washed with brine (10 mL), dried (Na_2SO_4), filtered, concentrated in vacuo.

To the solution of crude in CH_2Cl_2 (2 mL) was added *N*-Boc Acid (56 mg, 260 μmol) and HATU (148 mg, 0.390 mmol), then DIPEA (34 μL, 0.195 mmol) was added dropwise at 0 °C. The reaction was allowed to reach room temperature and stirred overnight. The mixture was concentrated, diluted with EtOAc (10 mL) and washed with aqueous 5% $NaHSO_4$ (3 × 5 mL) and brine (10 mL), dried (Na_2SO_4), filtered, concentrated in vacuo and purified by column chromatography (100/1 to 20/1 DCM/MeOH) to provide compound **15a** (107 mg, 43%) as a white foamed solid. $[\alpha]_D^{25}$ = −142.6 (c = 1.0, $CHCl_3$). ^{1}**H NMR** (400 MHz, $CDCl_3$) δ 7.25 (d, J = 2.0 Hz, 1H), 7.08 (d, J = 8.3 Hz, 2H), 7.00 (s, 1H), 6.76 (d, J = 8.6 Hz, 2H), 6.27 (s, 1H), 6.08 (d, J = 6.0 Hz, 1H), 5.69 (ddd, J = 3.9 Hz, 9.5 Hz, 41.1 Hz, 1H), 5.28 (d, J = 10.7 Hz, 1H), 4.97 (d, J = 10.9 Hz, 1H), 4.87 (dd, J = 2.8 Hz, 9.1 Hz, 1H), 4.85 – 4.76 (m, 1H), 4.40 (s, 2H), 4.13 (s, 1H), 3.94 (t, J = 11.5 Hz, 1H), 3.75 (s, 3H), 3.71 (s, 1H), 3.62 (s, 1H), 3.50 (s, 1H), 3.13 (s, 1H), 3.05 (s, 3H), 2.96 (s, 3H), 2.71 – 2.54 (m, 2H), 2.28 – 2.19 (m, 1H), 2.16 – 2.09 (m, 1H), 2.08 – 1.98 (m, 2H), 1.92 (d, J = 6.4

Hz, 1H), 1.88 (s, 2H), 1.86 – 1.77 (m, 1H)1.76 – 1.58 (m, 1H), 1.46 (s, 2H), 1.43 (s, 9H), 1.30 – 1.23 (m, 3H), 1.23 – 1.16 (m, 3H), 1.11 – 1.04 (m, 3H), 1.04 – 1.00 (m, 3H), 0.99 – 0.95 (m, 3H), 0.90 (s, 2H), 0.88 (s, 2H), 0.87 – 0.77 (m, 2H), 0.74 (d, *J* = 6.7 Hz, 3H), 0.55 (d, *J* = 3.2 Hz, 3H). **^{13}C NMR** (100 MHz, $CDCl_3$) *δ* 178.6, 174.3, 172.9, 172.5, 170.1, 169.8, 169.6, 168.7, 168.6, 158.4, 154.3, 154.0, 129.6, 128.5, 125.4, 125.4, 113.9, 80.4, 71.5, 71.2, 61.5, 59.3, 58.5, 58.1, 58.0, 57.3, 57.3, 56.3, 55.9, 55.1, 51.7, 47.2, 45.6, 41.6, 37.1, 36.7, 36.3, 33.8, 32.7, 30.8, 30.5, 30.4, 30.3, 29.7, 29.0, 28.4, 27.3, 23.0, 19.6, 19.2, 19.0, 18.4, 18.2, 17.7, 17.0, 14.2. **HRMS (ESI)** *m*/*z*:calcd for $C_{51}H_{79}N_7O_{11}Na^+$ $[M+Na]^+$: 988.5730, found: 988.5735.

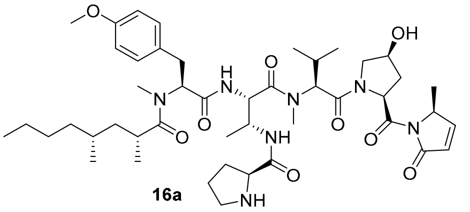

16a

Synthesis of (16a). To the solution of **15a** (96 mg, 99.4 μmol) in DCM (1 mL) was added CF_3COOH (250 μL) at 0 °C. The reaction was allowed to reach room temperature and stirred 2 h. The mixture was quenched with saturated aqueous NH_4Cl (2 mL). The aqueous phase was extracted with DCM (3 × 2 mL) and the combined organics were washed with brine (3 mL), dried (Na_2SO_4), filtered, concentrated in vacuo and purified by column chromatography (100/1 to 20/1 DCM/MeOH) to provide compound **16a** (65 mg, 76%) as a white foamed solid. $[\alpha]_D^{25}$ = −122.9 (*c* = 1.0, $CHCl_3$). **^{1}H NMR** (400 MHz, $CDCl_3$) *δ* 8.01 – 7.60 (m, 1H), 7.33 (s, 1H), 7.23 (d, *J* = 1.7 Hz, 1H), 7.09 (d, *J* = 8.1 Hz, 2H), 6.75 (d, *J* = 8.2 Hz, 2H), 6.06 (d, *J* = 6.1 Hz, 1H), 5.57 (ddd, *J* = 6.5 Hz, 8.6 Hz, 46.8 Hz, 1H), 5.47 – 5.37 (m, 1H), 4.97 – 4.92 (m, 1H), 4.88 (d, *J* = 8.0 Hz, 1H), 4.85 – 4.75 (m, 1H),, 4.45 (t, *J* = 6.9 Hz, 1H), 4.37 – 4.33 (m, 1H), 4.33 – 4.27 (m, 1H), 3.83 (s, 1H), 3.73 (s, 3H), 3.70 (s, 1H), 3.50 (ddd, *J* = 5.9 Hz, 10.6 Hz, 22.4 Hz, 1H), 3.27 – 3.19 (m, 1H), 3.18 – 3.15 (m, 1H), 3.15 – 3.10 (m, 1H), 3.05 (s, 3H, major rotamer), 3.04 (s, 3H, minor rotamer), 3.01 (s, 1H), 2.93 (s, 3H), 2.89 (d, *J* = 2.5 Hz, 1H), 2.88 – 2.80 (m, 1H), 2.69 – 2.58 (m, 2H), 2.26 – 2.15 (m, 2H), 1.87 – 1.82 (m, 1H), 1.82 – 1.76 (m, 2H), 1.76 – 1.68 (m, 1H), 1.50 – 1.42 (m, 2H), 1.41 – 1.31 (m, 2H), 1.22 – 1.18 (m, 2H), 1.18 – 1.13 (m, 3H), 1.29 – 1.08 (m, 3H), 1.03 (d, *J* = 6.6 Hz, 3H), 0.98 (d, *J* = 6.5 Hz, 2H), 0.96 – 0.91 (m, 3H), 0.88 (d, *J* = 6.8 Hz, 3H), 0.76 – 0.71 (m, 3H), 0.54 (d, *J* = 5.4 Hz, 3H). **^{13}C NMR** (100 MHz, $CDCl_3$) *δ* 178.2, 175.4, 173.0, 172.8, 171.6, 170.7, 169.8, 169.6, 168.1, 168.0, 167.9, 158.3, 154.2, 154.0, 129.7, 128.6, 125.4, 125.4, 113.8, 70.2, 69.9, 60.4, 59.9, 58.2, 58.1, 57.9, 57.2, 55.1, 52.5, 46.9, 46.0, 41.6, 36.8, 36.3, 36.0, 33.6, 33.0, 31.1, 30.8, 30.5, 30.5, 30.2, 29.0, 27.1, 27.1, 25.7, 23.0, 19.6, 19.3, 19.1, 18.8, 18.8, 18.2, 18.2, 17.8, 17.6, 17.0, 14.2. **HRMS (ESI)** *m*/*z*: calcd for $C_{46}H_{72}N_7O_9^+$ $[M+H]^+$: 866.5386, found: 866.5391.

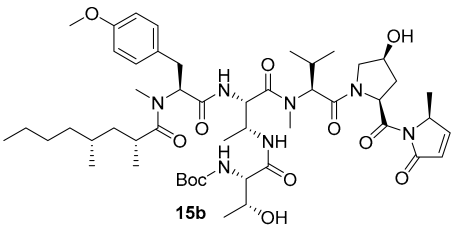

15b

Synthesis of (15b). The titled compound **15b** was obtained following the general procedure described for **15a**. The reaction was purified by column chromatography (100/1 to 20/1 DCM/MeOH) to provide compound **15b** (90 mg, 40%) as a white foamed solid. $[\boldsymbol{\alpha}]_{\boldsymbol{D}}^{\mathbf{25}}$ = −95.6 (c = 1.0, $CHCl_3$). **^{1}H NMR** (400 MHz, $CDCl_3$) δ 7.33 (d, J = 9.3 Hz, 1H), 7.25 – 7.15 (m, 1H), 7.07 (d, J = 8.3 Hz, 2H), 6.76 (d, J = 8.6 Hz, 2H), 6.50 (d, J = 9.2 Hz, 1H), 6.08 (dt, J = 2.1 Hz, 6.1 Hz, 1H), 6.00 – 5.79 (m, 1H), 5.69 (ddd, J = 3.6 Hz, 9.6 Hz, 44.7 Hz, 1H), 5.18 (dd, J = 5.9 Hz, 10.7 Hz, 1H), 4.98 (d, J = 11.0 Hz, 1H), 4.88 – 4.83 (m, 1H), 4.83 – 4.73 (m, 1H), 4.50 – 4.44 (m, 1H), 4.44 – 4.40 (m, 1H), 4.30 (s, 1H), 4.10 (d, J = 8.8 Hz, 1H), 4.05 – 3.96 (m, 1H), 3.75 (s, 3H), 3.74 – 3.71 (m, 1H), 4.70 – 3.60 (m, 1H), 3.29 – 3.14 (m, 1H), 3.12 (s, 3H, major rotamer), 3.10 (s, 3H, minor rotamer), 3.09 – 3.02 (m, 1H), 2.98 (s, 3H), 2.96 – 2.89 (m, 1H), 2.74 – 2.65 (m, 1H), 2.62 – 2.53 (m, 1H), 2.29 – 2.20 (m, 1H), 2.88 – 2.01 (m, 1H), 1.78 – 1.65 (m, 1H), 1.45 (d, J = 4.0 Hz, 9H), 1.44 – 1.40 (m, 3H), 1.26 – 1.23 (m, 2H), 1.22 – 1.19 (m, 3H), 1.19 – 1.16 (m, 2H), 1.15 – 1.10 (m, 2H), 1.09 – 1.06 (m, 2H), 1.06 – 1.03 (m, 3H), 1.01 – 0.95 (m, 3H), 0.89 (d, J = 6.9 Hz, 3H), 0.87 – 0.78 (m, 3H), 0.74 (d, J = 6.7 Hz, 3H), 0.52 (d, J = 5.5 Hz, 3H). **^{13}C NMR** (100 MHz, $CDCl_3$) δ 179.3, 174.1, 172.8, 171.4, 171.1, 171.1, 170.2, 169.8, 169.6, 168.5, 168.5, 158.4, 156.5, 154.3, 154.1, 129.6, 128.3, 125.4, 113.9, 113.9, 80.3, 71.6, 71.3, 67.5, 60.8, 59.3, 58.5, 58.1, 57.9, 57.7, 56.3, 56.0, 55.1, 52.0, 51.9, 45.8, 42.2, 37.1, 36.8, 36.4, 33.9, 32.8, 31.2, 30.7, 30.7, 30.4, 29.0, 28.4, 27.4, 23.1, 19.3, 19.1, 19.1, 18.9, 18.4, 18.3, 18.2, 18.0, 17.6, 17.0, 14.2. **HRMS (ESI)** m/z: calcd for $C_{50}H_{79}N_7O_{12}Na^+$ $[M+Na]^+$: 992.5679, found: 992.5679.

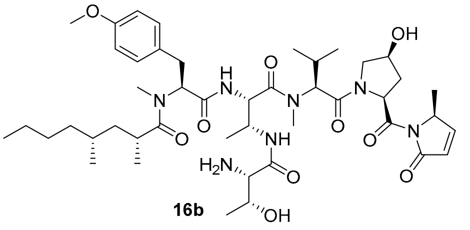

16b

Synthesis of (16b). The titled compound **16b** was obtained following the general procedure described for **16a**. The reaction was purified by column chromatography (100/1 to 20/1 DCM/MeOH) to provide compound **16b** (60 mg, 70%) as a white foamed solid. $[\boldsymbol{\alpha}]_{\boldsymbol{D}}^{\mathbf{25}}$ = −124.5 (c = 1.0, $CHCl_3$). **^{1}H NMR** (400 MHz, $CDCl_3$) δ 7.69 (d, J = 9.1 Hz, 1H), 7.40 (d, J = 8.7 Hz, 1H), 7.24 (d, J = 7.2 Hz, 1H), 7.08 (d, J = 8.1 Hz, 2H), 6.75 (d, J = 8.0 Hz, 2H), 6.07 (d, J = 6.0 Hz, 1H), 5.74 – 5.54 (m, 1H), 5.38 (dd, J = 5.8 Hz, 11.0 Hz, 1H), 4.95 (d, J = 10.9 Hz, 1H), 4.92 – 4.87

(m, 1H), 4.84 – 4.75 (m, 1H), 4.51 – 4.45 (m, 1H), 4.43 – 4.38 (m, 1H), 4.26 – 4.20 (m, 1H), 4.01 – 3.93 (m, 1H), 3.74 (s, 3H), 3.69 – 3.62 (m, 1H), 3.59 (dd, *J* = 4.2 Hz, 10.8 Hz, 1H), 3.30 (s, 1H), 3.25 (d, *J* = 13.1 Hz, 1H), 3.12 (s, 3H), 3.08 (d, *J* = 4.5 Hz, 1H), 2.99 (s, 3H), 2.94 (s, 1H), 2.93 – 2.86 (m, 1H), 2.70 – 2.58 (m, 2H), 2.27 – 2.18 (m, 1H), 1.93 – 1.82 (m, 1H), 1.61 – 1.48 (m, 1H), 1.42 (dd, *J* = 6.6 Hz, 14.7 Hz, 3H), 1.23 (s, 3H), 1.20 – 1.16 (m, 2H), 1.13 (s, 3H), 1.12 (s, 3H), 1.04 (d, *J* = 6.6 Hz, 3H), 1.00 (d, *J* = 6.4 Hz, 2H), 0.96 (d, *J* = 6.4 Hz, 2H), 0.92 – 0.87 (m, 3H), 0.86 (s, 2H), 0.74 (d, *J* = 6.7 Hz, 3H), 0.51 (d, *J* = 4.8 Hz, 3H). **^{13}C NMR** (100 MHz, $CDCl_3$) δ 178.5, 173.5, 172.2, 171.0, 170.9, 169.8, 169.6, 168.4, 168.3, 158.3, 154.3, 154.1, 129.6, 128.4, 125.4, 113.8, 71.0, 70.7, 67.9, 60.4, 60.4, 59.5, 58.5, 58.4, 58.1, 57.9, 56.9, 55.1, 52.2, 52.1, 45.7, 41.7, 37.0, 36.6, 36.3, 33.7, 33.1, 31.0, 30.7, 30.6, 30.3, 29.0, 27.4, 27.4, 23.0, 19.4, 19.1, 18.9, 18.6, 18.5, 18.3, 18.3, 17.9, 17.6, 17.0, 14.2. **HRMS (ESI)** *m/z*: calcd for $C_{45}H_{72}N_7O_{10}^+$ [M+H]$^+$: 870.5335, found: 870.5341.

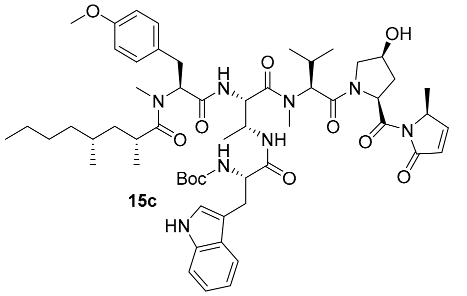

15c

Synthesis of (15c). The titled compound **15c** was obtained following the general procedure described for **15a**. The reaction was purified by column chromatography (100/1 to 20/1 DCM/MeOH) to provide compound **15c** (95 mg, 46%) as a white foamed solid. $[\boldsymbol{\alpha}]_{\boldsymbol{D}}^{25}$ = −111.8 (*c* = 1.0, $CHCl_3$). **^{1}H NMR** (400 MHz, $CDCl_3$) δ 9.11 – 8.91 (m, 1H), 7.65 (d, *J* = 7.8 Hz, 1H), 7.33 (d, *J* = 7.5 Hz, 1H), 7.24 – 7.17 (m, 1H), 7.16 – 7.12 (m, 1H), 7.10 (d, *J* = 7.5 Hz, 1H), 7.08 – 7.06 (m, 1H), 7.04 (d, *J* = 8.6 Hz, 2H), 7.03 – 6.97 (m, 1H), 6.74 (d, *J* = 8.7 Hz, 2H), 6.15 (s, 1H), 6.06 – 5.99 (m, 1H), 5.72 – 5.57 (m, 1H), 5.46 (s, 1H), 5.38 – 5.30 (m, 1H), 4.95 (d, *J* = 10.8 Hz, 1H), 4.79 – 4.73 (m, 1H), 4.73 – 4.64 (m, 1H), 4.37 (s, 1H), 4.31 (s, 1H), 4.22 (s, 1H), 3.94 – 3.80 (m, 1H), 3.73 (s, 3H), 3.72 – 3.64 (m, 1H), 3.64 – 3.42 (m, 1H), 3.24 (dd, *J* = 6.0 Hz, 14.6 Hz, 1H), 3.19 – 3.14 (m, 1H), 3.12 (dd, *J* = 4.3 Hz, 10.5 Hz, 1H), 3.00 (s, 1H), 2.99 (s, 1H), 2.93 – 2.89 (m, 1H), 2.86 (s, 3H, major rotamer), 2.84 (s, 3H, minor rotamer), 2.78 (s, 3H, major rotamer), 2.71 (s, 3H, minor rotamer), 2.65 – 2.58 (m, 1H), 2.52 (d, *J* = 7.1 Hz, 1H), 2.26 – 2.16 (m, 1H), 1.89 (t, *J* = 14.6 Hz, 1H), 1.55 – 1.39 (m, 3H), 1.37 (s, 9H), 1.27 – 1.18 (m, 3H), 1.17 – 1.08 (m, 3H), 1.01 (s, 2H), 0.99 – 0.96 (m, 3H), 0.96 – 0.91 (m, 2H), 0.89 – 0.85 (m, 3H), 0.85 – 0.83 (m, 2H), 0.83 – 0.79 (m, 2H), 0.74 (dd, *J* = 3.0 Hz, 6.6 Hz, 3H), 0.53 (d, *J* = 4.8 Hz, 3H). **^{13}C NMR** (100 MHz, $CDCl_3$) δ 178.5, 173.9, 172.6, 171.6, 171.6, 170.7, 170.2, 170.2, 169.9, 169.6, 168.9, 168.7, 158.3, 155.4, 154.4, 154.2, 136.3, 129.6, 128.5, 127.5, 125.2, 125.1, 123.4, 122.0, 119.5, 118.7, 113.8, 111.4, 111.2, 110.4, 80.0, 70.9, 59.2, 58.5, 58.1, 57.9, 57.2, 56.1, 55.3, 55.0, 52.8, 46.7, 41.6, 38.6, 36.9, 33.5, 33.2, 31.1, 30.4, 30.4, 30.2, 28.9,

28.8, 28.2, 27.3, 23.0, 19.4, 19.0, 18.8, 18.5, 18.4, 17.9, 17.5, 16.9, 14.1. **HRMS (ESI)** *m/z*: calcd for $C_{57}H_{82}N_8O_{11}Na^+$ $[M+Na]^+$: 1077.5995, found: 1077.5994.

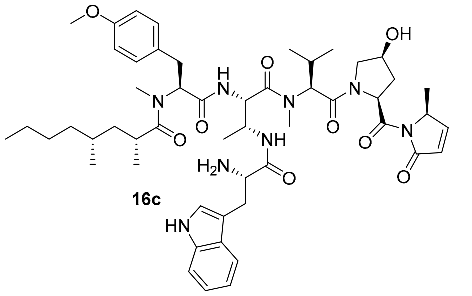

16c

Synthesis of (16c). The titled compound **16c** was obtained following the general procedure described for **16a**. The reaction was purified by column chromatography (100/1 to 20/1 DCM/MeOH) to provide compound **16c** (55 mg, 73%) as a white foamed solid. $[\alpha]_D^{25}$ = – 114.4 (*c* = 1.0, $CHCl_3$); **^{1}H NMR** (400 MHz, $CDCl_3$) *δ* 8.94 (s, 1H), 7.64 (d, *J* = 7.8 Hz, 1H), 7.36 (d, *J* = 8.0 Hz, 1H), 7.25 – 7.21 (m, 1H), 7.20 (d, *J* = 1.8 Hz, 1H), 7.17 (d, *J* = 7.8 Hz, 1H), 7.13 (s, 1H), 7.11 (s, 1H), 7.06 (d, *J* = 8.2 Hz, 2H), 6.75 (d, *J* = 8.2 Hz, 2H), 6.08 – 6.00 (m, 1H), 5.68 – 5.50 (m, 1H), 5.49 – 5.43 (m, 1H)), 4.94 (dd, *J* = 2.3 Hz, 10.9 Hz, 1H), 4.91 – 4.86 (m, 1H), 4.81 – 4.74 (m, 1H), 4.35 – 4.31 (m, 1H), 4.31 – 4.26 (m, 1H), 3.81 – 3.74 (m, 1H), 3.74 (s, 3H), 3.72 – 3.69 (m, 1H), 3.69 – 3.65 (m, 1H), 3.57 – 3.48 (m, 1H), 3.25 (d, *J* = 5.4 Hz, 1H), 3.22 (d, *J* = 5.2 Hz, 1H), 3.16 (d, *J* = 5.8 Hz, 1H), 3.13 – 3.09 (m, 1H), 3.03 (s, 3H), 2.95 (s, 3H), 2.92 – 2.88 (m, 1H), 2.88 – 2.80 (m, 1H), 2.67 – 2.56 (m, 2H), 2.28 – 2.21 (m, 1H), 1.85 – 1.74 (m, 1H), 1.56 – 1.47 (m, 1H), 1.44 (d, *J* = 6.7 Hz, 2H), 1.39 – 1.29 (m, 2H), 1.24 – 1.16 (m, 3H), 1.16 – 1.08 (m, 3H), 1.04 (d, *J* = 6.5 Hz, 3H), 0.99 (d, *J* = 6.5 Hz, 3H), 0.94 (d, *J* = 6.4 Hz, 2H), 0.87 (d, *J* = 6.8 Hz, 3H), 0.84 – 0.78 (m, 2H), 0.78 – 0.74 (m, 3H), 0.51 (d, *J* = 5.1 Hz, 3H); **^{13}C NMR** (100 MHz, $CDCl_3$) *δ* 178.5, 175.4, 174.1, 173.7, 173.2, 171.8, 170.7, 170.3, 169.8, 169.6, 168.3, 168.2, 168.1, 158.3, 154.3, 154.0, 136.4, 129.6, 128.4, 127.3, 125.4, 125.3, 123.6, 122.1, 119.6, 118.7, 113.9, 111.5, 110.7, 70.1, 69.8, 59.6, 58.4, 58.3, 58.1, 57.9, 57.0, 55.4, 55.3, 55.1, 53.4, 52.6, 52.5, 46.4, 41.6, 36.9, 36.3, 35.9, 33.6, 33.2, 31.4, 31.0, 30.5, 30.2, 29.0, 27.1, 25.2, 23.0, 19.5, 19.2, 19.2, 19.1, 18.4, 18.3, 18.2, 17.9, 17.6, 17.0, 14.2. **HRMS (ESI)** *m/z*: calcd for $C_{52}H_{75}N_8O_9^+$ $[M+H]^+$: 955.5652, found: 955.5656.

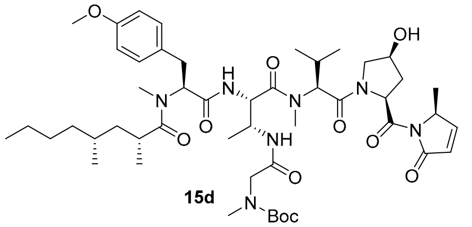

15d

Synthesis of (15d). The titled compound **15d** was obtained following the general procedure described for **15a**. The reaction was purified by column chromatography (100/1 to 20/1 DCM/MeOH) to provide compound **15d** (89 mg, 46%) as a white foamed solid. $[\alpha]_D^{25}$ =

−114.7 (c = 1.0, $CHCl_3$). **¹H NMR** (400 MHz, $CDCl_3$) δ 7.22 (d, J = 6.0 Hz, 1H), 7.06 (d, J = 8.1 Hz, 2H), 7.00 (d, J = 9.0 Hz, 1H), 6.74 (d, J = 8.1 Hz, 2H), 6.48 – 6.14 (m, 1H), 6.04 (d, J = 6.1 Hz, 1H), 5.68 – 5.51 (m, 1H), 5.27 (d, J = 2.5 Hz, 1H), 4.94 (d, J = 10.9 Hz, 1H), 4.88 – 4.82 (m, 1H), 4.82 – 4.74 (m, 1H), 4.51 – 4.40 (m, 1H), 4.40 – 4.29 (m, 1H), 3.90 (s, 1H), 3.83 (s, 2H), 3.73 (s, 3H), 3.70 (s, 1H), 3.63 – 3.46 (m, 1H), 3.13 (d, J = 18.7 Hz, 1H), 3.02 (s, 3H), 2.96 (s, 3H), 2.91 (s, 3H), 2.85 – 2.76 (m, 1H), 2.73 – 2.63 (m, 1H), 2.63 – 2.53 (m, 1H), 2.25 – 2.16 (m, 1H), 1.94 – 1.76 (m, 1H), 1.66 – 1.46 (m, H), 1.44 (s, 9H), 1.41 – 1.33 (m, 3H), 1.21 – 1.17 (m, 2H), 1.14 (s, 3H), 1.06 (d, J = 5.9 Hz, 3H), 1.01 (s, 2H), 0.99 – 0.96 (m, 3H), 0.95 – 0.90 (m, 3H), 0.90 – 0.86 (m, 2H), 0.72 (d, J = 6.8 Hz, 3H), 0.54 (s, 3H). **¹³C NMR** (100 MHz, $CDCl_3$) δ 178.7, 175.3, 174.5, 173.6, 172.2, 169.9, 169.8, 169.5, 168.6, 168.3, 168.3, 158.4, 156.4, 156.4, 154.2, 154.0, 129.6, 128.4, 125.4, 125.3, 113.9, 80.7, 70.7, 59.2, 58.4, 58.1, 57.9, 57.6, 55.8, 55.1, 53.4, 52.3, 52.0, 46.7, 41.7, 37.0, 36.5, 36.3, 36.1, 35.5, 33.7, 32.8, 31.0, 30.4, 30.4, 30.3, 29.0, 28.3, 27.3, 25.2, 23.0, 19.5, 19.2, 19.1, 18.9, 18.3, 18.1, 17.6, 17.0, 14.1. **HRMS (ESI)** m/z: calcd for $C_{49}H_{77}N_7O_{11}Na^+$ $[M+Na]^+$: 962.5573, found: 962.5567.

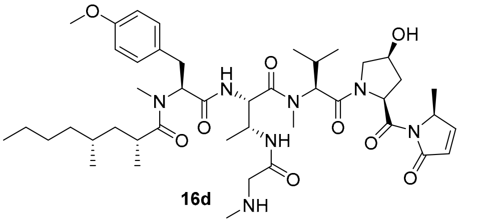

16d

Synthesis of (16d). The titled compound **16d** was obtained following the general procedure described for **16a**. The reaction was purified by column chromatography (100/1 to 20/1 DCM/MeOH) to provide compound **16d** (61 mg, 75%) as a white foamed solid. $[\boldsymbol{\alpha}]_{\boldsymbol{D}}^{\mathbf{25}}$ = −114.4 (c = 1.0, $CHCl_3$). **¹H NMR** (400 MHz, $CDCl_3$) δ 7.24 – 7.21 (m, 1H), 7.21 – 7.15 (m, 1H), 7.07 (d, J = 8.3 Hz, 2H), 6.74 (d, J = 8.2 Hz, 2H), 6.05 (d, J = 5.9 Hz, 1H), 5.72 – 5.48 (m, 1H), 5.39 (dd, J = 5.8 Hz, 11.0 Hz, 1H), 5.00 – 4.92 (m, 1H), 4.92 – 4.88 (m, 1H), 4.85 – 4.72 (m, 1H), 4.44 – 4.31 (m, 2H), 3.72 (s, 3H), 3.71 – 3.64 (m, 1H), 3.61 – 3.54 (m, 1H), 3.52 (dd, J = 4.9 Hz, 10.6 Hz, 1H), 3.26 (d, J = 16.1 Hz, 1H), 3.20 (d, J = 2.4 Hz, 1H), 3.16 (t, J = 5.3 Hz, 1H), 3.13 – 3.05 (m, 1H), 3.04 (s, 3H, minor rotamer), 3.03 (s, 3H, major rotamer), 2.93 (s, 3H, major rotamer), 2.91 (s, 3H, minor rotamer), 2.88 – 2.73 (m, 1H), 2.69 – 2.61 (m, 1H), 2.60 – 2.50 (m, 1H), 2.44 (s, 3H), 2.29 – 2.18 (m, 1H), 1.87 – 1.74 (m, 1H), 1.59 – 1.45 (m, 1H), 1.43 (d, J = 6.7 Hz, 1H), 1.41 – 1.32 (m, 2H), 1.25 – 1.17 (m, 3H), 1.16 – 1.11 (m, 2H), 1.09 (d, J = 6.8 Hz, 3H), 1.06 – 0.99 (m, 3H), 0.97 (d, J = 6.4 Hz, 2H), 0.94 – 0.88 (m, 3H), 0.88 – 0.85 (m, 3H), 0.84 – 0.79 (m, 2H), 0.74 (d, J = 6.6 Hz, 3H), 0.52 (d, J = 5.0 Hz, 3H). **¹³C NMR** (100 MHz, $CDCl_3$) δ 178.5, 173.4, 172.1, 170.5, 170.5, 170.1, 170.1, 170.1, 169.8, 169.5, 168.2, 168.1, 158.3, 154.2, 154.0, 129.6, 128.5, 125.4, 125.3, 113.9, 70.5, 70.2, 59.6, 58.4, 58.1, 57.9, 57.1, 55.5, 55.2, 55.1, 53.8, 52.5, 52.4, 46.1, 46.1, 41.6, 36.9, 36.4, 36.0,

33.6, 32.8, 30.9, 30.4, 30.4, 30.3, 29.0, 27.0, 27.0, 23.0, 19.5, 19.2, 19.1, 18.6, 18.2, 18.2, 17.9, 17.6, 17.0, 14.1. **HRMS (ESI)** *m*/*z*: calcd for $C_{44}H_{70}N_7O_9^+$ [M+H]+: 840.5230, found: 840.5234.

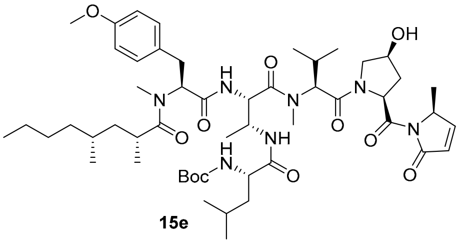

15e

Synthesis of (15e). The titled compound **15e** was obtained following the general procedure described for **15a**. The reaction was purified by column chromatography (100/1 to 20/1 DCM/MeOH) to provide compound **15e** (94 mg, 44%) as a white foamed solid. $[\alpha]_D^{25}$ = −122.6 (*c* = 1.0, $CHCl_3$). **^{1}H NMR** (400 MHz, $CDCl_3$) *δ* 7.24 (s, 1H), 7.07 (d, *J* = 8.3 Hz, 2H), 7.05 – 6.94 (m, 1H), 6.76 (d, *J* = 8.6 Hz, 2H), 6.57 (d, *J* = 7.8 Hz, 1H), 6.14 – 6.03 (m, 1H), 5.76 – 5.58 (m, 1H), 5.49 (s, 1H), 5.21 (dd, *J* = 5.6 Hz 11.7 Hz, 1H), 4.99 (d, *J* = 10.9 Hz, 1H), 4.88 – 4.82 (m, 1H), 4.82 – 4.75 (m, 1H), 4.46 – 4.38 (m, 1H), 4.37 – 4.28 (m, 1H), 4.13 (s, 1H), 3.96 – 3.83 (m, 1H), 3.75 (s, 3H, minor rotamer), 3.74 (s, 3H, major rotamer), 3.73 – 3.65 (m, 1H), 3.59 – 3.25 (m, 1H), 3.24 – 3.14 (m, 1H), 3.12 (d, *J* = 5.7 Hz, 1H), 3.05 (s, 3H, major rotamer), 3.02 (s, 3H, minor rotamer), 2.99 (s, 3H, minor rotamer), 2.98 (s, 3H, minor rotamer), 2.95 (s, 3H, major rotamer), 2.91 – 2.80 (m, 1H), 2.70 – 2.61 (m, H), 2.61 – 2.48 (m, 1H), 2.30 – 2.19 (m, H), 1.92 (t, *J* = 16.5 Hz, 1H), 1.68 – 1.63 (m, 1H), 1.62 – 1.54 (m, 2H), 1.45 (s, 9H, minor rotamer), 1.43 (s, 9H, major rotamer), 1.42 – 1.35 (m, 3H), 1.24 – 1.16 (m, 3H), 1.16 – 1.11 (m, 2H), 1.08 (d, *J* = 6.6 Hz, 3H), 1.03 (d, *J* = 6.7 Hz, 2H), 1.02 – 0.96 (m, 3H), 0.94 – 0.92 (m, 3H), 0.96 – 0.94 (m, 3H), 0.92 – 0.90 (m, 2H), 0.89 – 0.86 (m, 2H), 0.86 – 0.79 (m, 2H), 0.76 (d, *J* = 6.6 Hz, 3H), 0.53 (d, *J* = 4.8 Hz, 3H). **^{13}C NMR** (100 MHz, $CDCl_3$) *δ* 178.7, 174.3, 172.7, 170.7, 170.2, 169.8, 169.6, 168.7, 168.7, 158.4, 155.8, 154.4, 154.1, 129.5, 128.5, 125.4, 125.4, 113.9, 79.9, 71.6, 71.3, 59.3, 58.5, 58.1, 58.0, 56.5, 56.1, 55.1, 53.3, 52.6, 46.7, 41.7, 37.0, 36.7, 36.3, 33.7, 33.1, 31.4, 30.5, 30.4, 30.4, 29.0, 28.4, 27.2, 24.8, 23.2, 23.0, 21.9, 19.5, 19.1, 19.0, 18.8, 18.4, 18.0, 17.6, 17.0, 14.2. **HRMS (ESI)** *m*/*z*: calcd for $C_{52}H_{83}N_7O_{11}Na^+$ [M+Na]+: 1004.6043, found: 1004.6043.

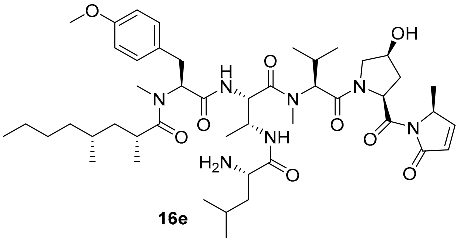

16e

Synthesis of (16e). The titled compound **16e** was obtained following the general procedure described for **16a**. The reaction was purified by column chromatography (100/1 to 20/1 DCM/MeOH) to provide compound **16e** (58 mg, 77%) as a white foamed solid. $[\alpha]_D^{25}$ = −125.1

(c = 1.0, $CHCl_3$). **^{1}H NMR** (400 MHz, $CDCl_3$) δ 7.25 – 7.22 (m, 1H), 7.09 (d, J = 8.3 Hz, 2H), 6.76 (d, J = 8.6 Hz, 2H), 6.07 (dd, J = 1.6 Hz, 6.1 Hz, 1H), 5.70 – 5.50 (m, 1H), 5.46 – 5.32 (m, 1H), 4.99 – 4.94 (m, 1H), 4.93 – 4.89 (m, 1H), 4.87 – 4.75 (m, 1H), 4.48 – 4.37 (m, 1H), 4.37 – 4.29 (m, 1H), 3.83 – 3.74 (m, 1H), 3.74 (s, 3H), 3.58 – 3.47 (m, 1H), 3.46 – 3.30 (m, 1H), 3.18 (dd, J = 5.8 Hz, 14.6 Hz, 1H), 3.14 – 3.07 (m, 1H), 3.05 (s, 3H, minor rotamer), 3.04 (s, 3H, major rotamer), 2.97 (s, 3H, major rotamer), 2.96 (s, 3H, minor rotamer), 2.95 – 2.92 (m, 1H), 2.92 – 2.82 (m, 1H), 2.70 – 2.58 (m, 2H), 2.31 – 2.18 (m, 1H), 1.88 – 1.76 (m, 1H), 1.74 – 1.65 (m, 1H), 1.61 – 1.53 (m, 1H), 1.45 (d, J = 6.7 Hz, 2H), 1.42 – 1.37 (m, 2H), 1.29 – 1.20 (m, 3H), 1.18 – 1.13 (m, 3H), 1.13 – 1.06 (m, 3H), 1.05 – 1.00 (m, 3H), 0.99 – 0.96 (m, 3H), 0.95 (d, J = 1.4 Hz, 2H), 0.94 – 0.92 (m, 3H), 0.91 (s, 2H), 0.88 (d, J = 6.8 Hz, 3H), 0.74 (d, J = 6.6 Hz, 3H), 0.54 (d, J = 5.4 Hz, 3H). **^{13}C NMR** (100 MHz, $CDCl_3$) δ 178.6, 173.3, 172.0, 170.5, 170.4, 169.8, 169.6, 168.2, 168.1, 158.3, 154.3, 154.0, 129.7, 128.5, 125.4, 125.4, 113.9, 70.4, 70.0, 59.7, 58.3, 58.1, 57.9, 57.3, 55.1, 53.2, 52.1, 46.1, 44.1, 41.6, 36.8, 36.2, 35.9, 33.7, 33.0, 31.0, 30.6, 30.5, 30.3, 29.0, 27.1, 27.1, 24.7, 23.4, 23.1, 21.4, 19.6, 19.3, 19.1, 18.8, 18.2, 17.9, 17.6, 17.0, 14.2. **HRMS (ESI)** *m/z*: calcd for $C_{47}H_{75}N_7O_9Na^+$ $[M+Na]^+$: 904.5518, found: 904.5522.

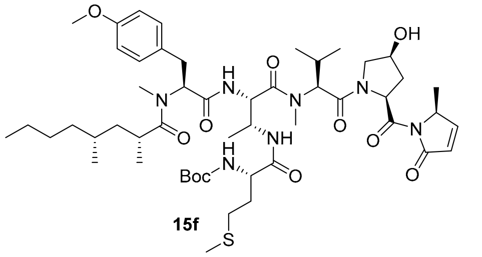

15f

Synthesis of (15f). The titled compound **15f** was obtained following the general procedure described for **15a**. The reaction was purified by column chromatography (100/1 to 20/1 DCM/MeOH) to provide compound **15f** (91 mg, 47%) as a white foamed solid. $[\alpha]_D^{25}$ = −120.1 (c = 1.0, $CHCl_3$). **^{1}H NMR** (400 MHz, $CDCl_3$) δ 7.24 (s, 1H), 7.07 (d, J = 8.5 Hz, 2H), 7.04 (s, 1H), 6.76 (d, J = 8.1 Hz, 2H), 6.63 (d, J = 8.8 Hz, 1H), 6.07 (d, J = 6.0 Hz, 1H), 5.74 (t, J = 10.9 Hz, 1H), 5.62 (d, J = 9.5 Hz, 1H), 5.23 – 5.10 (m, 1H), 4.99 (d, J = 10.9 Hz, 1H), 4.86 – 4.81 (m, 1H), 4.81 – 4.74 (m, 1H), 4.40 (s, 1H), 4.39 – 4.32 (m, 1H), 4.26 (s, 1H), 3.93 – 3.83 (m, 1H), 3.78 (s, 1H), 3.75 (s, 3H), 3.72 (s, 1H), 3.14 (dd, J = 5.5 Hz, 15.0 Hz, 1H), 3.05 (s, 3H), 2.94 (s, 3H), 2.89 (s, 1H), 2.69 – 2.63 (m, 1H), 2.59 – 2.55 (m, 1H), 2.55 – 2.48 (m, 2H), 2.28 – 2.19 (m, 1H), 1.17 – 1.11 (m, 1H), 2.09 (s, 3H), 1.98 – 1.88 (m, 2H), 1.64 – 1.51 (m, 1H), 1.51 – 1.45 (m, 2H), 1.43 (s, 9H), 1.41 – 1.33 (m, 3H), 1.18 – 1.12 (m, 3H), 1.09 (d, J = 6.4 Hz, 3H), 1.04 (d, J = 6.5 Hz, 3H), 0.98 – 0.95 (m, 3H), 0.95 – 0.91 (m, 2H), 0.90 – 0.87 (m, 2H), 0.84 – 0.78 (m, 2H), 0.76 (d, J = 6.7 Hz, 3H), 0.55 (d, J = 4.9 Hz, 3H). **^{13}C NMR** (100 MHz, $CDCl_3$) δ 178.8, 174.2, 171.6, 170.7, 170.1, 169.8, 169.6, 168.7, 158.4, 155.8, 154.4, 154.1, 129.5, 128.5, 125.4, 113.9, 80.1, 71.5, 71.2, 59.3, 58.5, 58.2, 58.1, 58.0, 56.5,

55.1, 53.7, 52.8, 46.9, 41.7, 36.9, 36.4, 33.8, 33.2, 32.2, 31.6, 30.5, 30.3, 30.3, 29.7, 29.7, 29.4, 29.0, 28.4, 27.2, 23.0, 19.5, 18.9, 18.8, 18.4, 17.9, 17.6, 17.0, 15.5, 14.2. **HRMS (ESI)** *m*/*z*: calcd for $C_{51}H_{81}N_7O_{11}SNa^+$ $[M+Na]^+$: 1022.5607, found: 1022.5605.

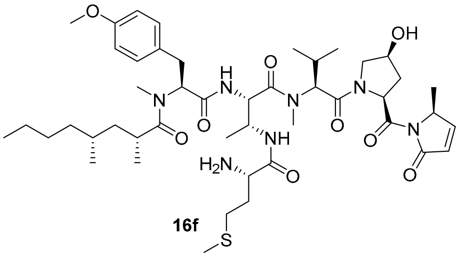

16f

Synthesis of (16f). The titled compound **16f** was obtained following the general procedure described for **16a**. The reaction was purified by column chromatography (100/1 to 20/1 DCM/MeOH) to provide compound **16f** (62 mg, 79%) as a white foamed solid. $[\alpha]_D^{25}$ = −116.1 (*c* = 1.0, $CHCl_3$). **^{1}H NMR** (400 MHz, $CDCl_3$) *δ* 7.48 – 7.28 (m, 1H), 7.25 (d, *J* = 3.0 Hz, 1H), 7.24 – 7.15 (m, 1H), 7.08 (d, *J* = 8.2 Hz, 2H), 6.75 (d, *J* = 8.3 Hz, 2H), 6.06 (dt, *J* = 1.4 Hz, 6.1 Hz, 1H), 5.61 (ddd, *J* = 5.4 Hz, 9.1 Hz, 48.0 Hz, 1H), 5.37 (dd, *J* = 6.1 Hz, 10.7 Hz, 1H), 5.05 – 4.93 (m, 1H), 4.93 – 4.90 (m, 1H), 4.86 – 4.74 (m, 1H), 4.41 (q, *J* = 5.3 Hz, 1H), 4.36 – 4.29 (m, 1H), 3.87 – 3.77 (m, 1H), 3.74 (s, 3H), 3.62 – 3.54 (m, 1H), 3.54 – 3.49 (m, 1H), 3.24 – 3.15 (m, 1H), 3.15 – 3.09 (m, 1H), 3.05 (s, 3H, major rotamer), 3.04 (s, 3H, minor rotamer), 2.99 (d, *J* = 10.3 Hz, 1H), 2.96 (s, 3H), 2.93 – 2.87 (m, 1H), 2.69 – 2.64 (m, 1H), 2.62 (d, *J* = 7.0 Hz, 1H), 2.60 – 2.54 (m, 2H), 2.28 – 2.20 (m, 1H), 2.09 (s, 3H), 2.08 – 2.02 (m, 1H), 1.89 – 1.72 (m, 2H), 1.53 – 1.45 (m, 1H), 1.44 (d, *J* = 6.7 Hz, 1H), 1.40 (d, *J* = 6.7 Hz, 2H), 1.27 – 1.19 (m, 3H), 1.18 – 1.14 (m, 2H), 1.12 (d, *J* = 7.0 Hz, 3H), 1.07 – 1.01 (m, 3H), 1.01 – 0.96 (m, 2H), 0.96 – 0.90 (m, 3H), 0.88 (d, *J* = 6.8 Hz, 3H), 0.86 – 0.82 (m, 2H), 0.75 (d, *J* = 6.6 Hz, 3H), 0.53 (d, *J* = 5.2 Hz, 3H). **^{13}C NMR** (100 MHz, $CDCl_3$) *δ* 178.5, 173.5, 172.1, 170.5, 170.5, 170.3, 169.8, 169.6, 168.4, 168.3, 158.3, 154.3, 154.1, 129.7, 128.5, 125.4, 125.3, 113.9, 70.6, 70.3, 59.6, 58.3, 58.1, 57.9, 57.3, 55.6, 55.3, 55.1, 53.9, 52.1, 52.0, 46.1, 41.6, 36.9, 36.3, 36.0, 34.0, 33.7, 32.9, 31.0, 30.6, 30.5, 30.4, 30.3, 29.0, 27.2, 27.1, 23.0, 19.5, 19.2, 19.0, 18.8, 18.8, 18.3, 18.3, 17.9, 17.6, 17.0, 15.4, 14.2. **HRMS (ESI)** *m*/*z*: calcd for $C_{46}H_{74}N_7O_9^+$ $[M+H]^+$: 900.5263, found: 900.5269.

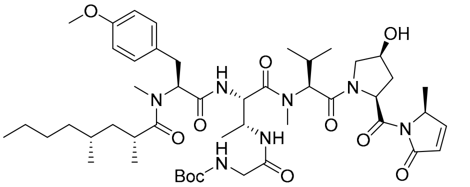

15g

Synthesis of (15g). The titled compound **15g** was obtained following the general procedure described for **15a**. The reaction was purified by column chromatography (100/1 to 20/1 DCM/MeOH) to provide compound **15g** (91 mg, 48%) as a white foamed solid. $[\alpha]_D^{25}$ =

−106.7 (c = 1.0, $CHCl_3$). **^{1}H NMR** (400 MHz, $CDCl_3$) δ 7.24 (dd, J = 2.1 Hz, 6.0 Hz, 1H), 7.06 (d, J = 8.3 Hz, 2H), 7.04 – 6.97 (m, 1H), 6.75 (d, J = 8.6 Hz, 2H), 6.42 (d, J = 9.0 Hz, 1H), 6.13 – 6.01 (m, 1H), 5.69 (dd, J = 4.2 Hz, 9.6 Hz, 1H), 5.62 – 5.46 (m, 1H), 5.20 (dd, J = 5.8 Hz, 10.9 Hz, 1H), 5.02 – 4.93 (m, 1H), 4.86 (dd, J = 5.0 Hz, 8.9 Hz, 1H), 4.83 – 4.73 (m, 1H), 4.47 – 4.39 (m, 1H), 4.39 – 4.31 (m, 1H), 3.92 – 3.83 (m, 1H), 3.82 – 3.75 (m, 2H), 3.73 (s, 3H), 3.72 – 3.66 (m, 1H), 3.23 – 3.12 (m, 1H), 3.10 (s, 3H, minor rotamer), 3.05 (s, 3H, major rotamer), 3.03 – 2.97 (m, 1H), 2.94 (s, 3H, minor rotamer), 2.93 (s, 3H, major rotamer), 2.89 – 2.80 (m, 1H), 2.72 – 2.62 (m, 1H), 2.61 – 2.52 (m, 1H), 2.28 – 2.19 (m, 1H), 1.89 (tt, J = 4.0 Hz, 14.2 Hz, 1H), 1.62 – 1.50 (m, 1H), 1.43 (s, 9H), 1.42 – 1.37 (m, 3H), 1.25 – 1.19(m, 2H), 1.20 – 1.15 (m, 2H), 1.14 – 1.10 (m, 2H), 1.10 – 1.05 (m, 3H), 1.04 – 0.98 (m, 3H), 0.99 – 0.93 (m, 3H), 0.93 – 0.88 (m, 2H), 0.87 (d, J = 6.8 Hz, 3H), 0.76 (d, J = 5.0 Hz, 3H), 0.53 (d, J = 4.9 Hz, 3H). **^{13}C NMR** (100 MHz, $CDCl_3$) δ 178.8, 178.8, 173.8, 172.5, 170.5, 170.1, 170.0, 169.8, 169.5, 168.6, 168.5, 158.3, 156.1, 154.3, 154.1, 129.5, 128.4, 125.3, 125.3, 113.9, 80.1, 71.2, 70.8, 59.3, 58.5, 58.1, 57.9, 57.9, 56.3, 56.0, 55.1, 52.5, 47.0, 44.2, 41.7, 37.0, 36.7, 36.3, 33.7, 32.9, 31.3, 31.3, 30.4, 30.4, 30.3, 29.0, 28.3, 27.2, 27.2, 23.0, 19.4, 19.1, 18.9, 18.7, 18.6, 18.4, 18.3, 18.0, 17.6, 17.0, 14.1. **HRMS (ESI)** m/z: calcd for $C_{48}H_{75}N_7O_{11}Na^+$ $[M+Na]^+$: 948.5417, found: 948.5421.

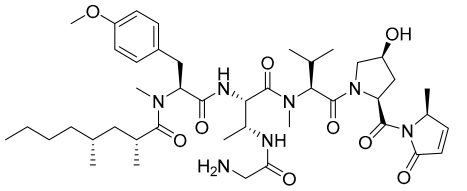

16g

Synthesis of (16g). The titled compound **16g** was obtained following the general procedure described for **16a**. The reaction was purified by column chromatography (100/1 to 20/1 DCM/MeOH) to provide compound **16g** (63 mg, 80%) as a white foamed solid. $[\boldsymbol{\alpha}]_{\boldsymbol{D}}^{25}$ = −127.0 (c = 1.0, $CHCl_3$). **^{1}H NMR** (400 MHz, $CDCl_3$) δ 7.59 – 7.34 (m, 1H), 7.33 – 7.27 (m, 1H), 7.25 – 7.15 (m, 1H), 7.08 (d, J = 8.2 Hz, 2H), 6.76 (d, J = 7.9 Hz, 2H), 6.13 – 6.01 (m, 1H), 5.61 (ddd, J = 5.4 Hz, 9.0 Hz, 48.0 Hz, 1H), 5.41 (dd, J = 5.7 Hz, 11.1 Hz, 1H), 5.03 – 4.95 (m, 1H), 4.95 – 4.89 (m, 1H), 4.86 – 4.73 (m, 1H), 4.42 (d, J = 6.1 Hz, 1H), 4.39 (d, J = 5.7 Hz, 1H), 3.79 (dd, J = 5.6 Hz, 10.9 Hz, 1H), 3.74 (s, 3H), 3.73 – 3.68 (m, 1H), 3.66 – 3.58 (m, 1H), 3.58 – 3.49 (m, 1H), 3.39 (s, 2H), 3.33 (d, J = 22.3 Hz, 1H), 3.22 – 3.17 (m, 1H), 3.17 – 3.10 (m, 1H), 3.06 (s, 3H, major rotamer), 3.05 (s, 3H, minor rotamer), 2.95 (s, 3H), 2.92 – 2.86 (m, 1H), 2.69 – 2.62 (m, 1H), 2.62 – 2.55 (m, 1H), 2.29 – 2.20 (m, 1H), 1.90 – 1.78 (m, 1H), 1.42 (dd, J = 6.7 Hz, 15.6 Hz, 3H), 1.25 – 1.17 (m, 3H), 1.16 – 1.11 (m, 3H), 1.11 – 1.09 (m, 2H), 1.04 – 1.00 (m, 3H), 1.00 – 0.96 (m, 2H), 0.96 – 0.91 (m, 2H), 0.90 – 0.86(m, 3H), 0.85 – 0.82 (m, 2H), 0.76 (d, J = 3.8 Hz, 3H), 0.52 (d, J = 5.2 Hz, 3H). **^{13}C NMR** (100 MHz, $CDCl_3$) δ 178.7, 173.5, 172.2, 171.1, 170.6, 170.4, 169.8, 169.6, 168.4, 168.3,

158.3, 154.3, 154.1, 129.7, 128.5, 125.4, 125.4, 113.9, 70.6, 70.3, 59.6, 58.4, 58.1, 58.0, 57.2, 55.6, 55.4, 55.1, 52.6, 52.5, 46.1, 44.0, 41.6, 36.8, 36.5, 36.1, 33.6, 33.0, 31.0, 30.5, 30.5, 30.2, 29.0, 27.2, 27.1, 23.0, 19.5, 19.2, 19.1, 18.6, 18.5, 18.4, 18.3, 17.8, 17.6, 17.0, 14.2. **HRMS (ESI)** *m*/*z*: calcd for $C_{43}H_{68}N_7O_9^+$ [M+H]+: 826.5073, found: 826.5078.

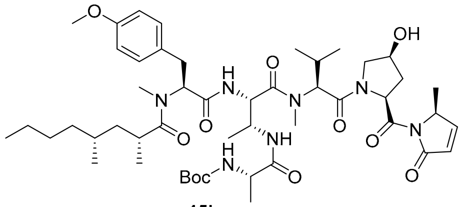

15h

Synthesis of (15h). The titled compound **15h** was obtained following the general procedure described for **15a**. The reaction was purified by column chromatography (100/1 to 20/1 DCM/MeOH) to provide compound **15h** (91 mg, 44%) as a white foamed solid. $[\alpha]_D^{25}$ = −120.4 (*c* = 1.0, CHCl3). **¹H NMR** (400 MHz, CDCl3) *δ* 7.26 – 7.20 (m, 1H), 7.06 (d, *J* = 8.5 Hz, 2H), 7.05 – 6.95 (m, 1H), 6.76 (d, *J* = 8.6 Hz, 2H), 6.64 – 6.41 (m, 1H), 6.16 – 6.00 (m, 1H), 5.78 – 5.67 (m, 1H), 5.65 – 5.50(m, 1H), 5.15 (dd, *J* = 5.7 Hz, 11.2 Hz, 1H), 4.99 (d, *J* = 11.0 Hz, 1H), 4.88 – 4.82 (m, 1H), 4.82 – 4.73 (m, 1H), 4.48 – 4.38 (m, 1H), 4.38 – 4.32 (m, 1H), 4.17 (s, 1H), 3.95 – 3.82 (m, 1H), 3.74 (s, 3H), 3.73 – 3.69 (m, 1H), 3.26 – 3.13 (m, 1H), 3.12 (d, *J* = 5.5 Hz, 1H), 3.06 (s, 3H, major rotamer), 3.04 (s, 3H, minor rotamer), 2.94 (s, 3H, minor rotamer), 2.93 (s, 3H, major rotamer), 2.90 – 2.82 (m, 1H), 2.69 – 2.60 (m, 1H), 2.60 – 2.50 (m, 1H), 2.29 – 2.19 (m, 1H), 1.98 – 1.85 (m, 1H), 1.66 – 1.51 (m, 1H), 1.45 (s, 3H, minor rotamer), 1.43 (s, 3H, major rotamer), 1.42 – 1.38 (m, 3H), 1.38 – 1.34 (m, 3H), 1.34 – 1.24 (m, 2H), 1.21 – 1.16 (m, 2H), 1.16 – 1.11 (m, 2H), 1.08 (d, *J* = 6.9 Hz, 3H), 1.05 – 1.00 (m, 3H), 1.00 – 0.94 (m, 3H), 0.94 – 0.89 (m, 2H), 0.88 – 0.84 (m, 3H), 0.76 (d, *J* = 6.6 Hz, 3H), 0.55 (d, *J* = 5.4 Hz, 3H). **¹³C NMR** (100 MHz, CDCl3) *δ* 178.7, 174.2, 172.8, 170.6, 170.2, 169.8, 169.6, 168.8, 168.7, 158.4, 155.6, 154.4, 154.1, 129.5, 128.5, 125.4, 125.3, 113.9, 79.9, 71.5, 71.2, 59.3, 58.5, 58.4, 58.1, 58.0, 56.5, 56.2, 55.1, 52.7, 50.2, 46.8, 41.7, 36.9, 36.4, 33.7, 33.1, 31.6, 30.5, 30.5, 30.3, 29.0, 28.4, 27.3, 27.2, 23.0, 19.5, 19.1, 18.9, 18.7, 18.4, 18.3, 17.9, 17.6, 17.0, 14.2. **HRMS (ESI)** *m*/*z*: calcd for $C_{49}H_{77}N_7O_{11}Na^+$ [M+Na]+: 962.5573, found: 962.5576.

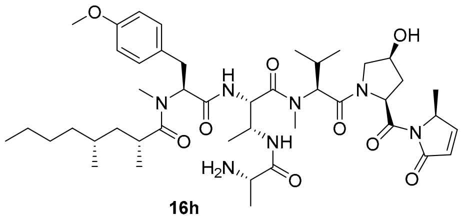

16h

Synthesis of (16h). The titled compound **16h** was obtained following the general procedure described for **16a**. The reaction was purified by column chromatography (100/1 to 20/1 DCM/MeOH) to provide compound **16h** (59 mg, 74%) as a white foamed solid. $[\alpha]_D^{25}$ =

−130.0 (c = 1.0, $CHCl_3$). **^{1}H NMR** (400 MHz, $CDCl_3$) δ 7.52 – 7.33 (m, 1H), 7.28 – 7.26 (m, 1H), 7.24 (d, J = 7.2 Hz, 1H), 7.09 (d, J = 8.2 Hz, 2H), 6.75 (d, J = 8.7 Hz, 2H), 6.06 (d, J = 6.0 Hz, 1H), 5.70 – 5.49 (m, 1H), 5.41 (dd, J = 6.0 Hz, 10.8 Hz, 1H), 4.97 – 4.93 (m, 1H), 4.93 – 4.87 (m, 1H), 4.85 – 4.75 (m, 1H), 4.44 – 4.37 (m, 1H), 4.36 – 4.29(m, 1H), 3.87 – 3.75 (m, 1H), 3.74 (s, 3H), 3.72 – 3.68 (m, 1H), 3.67 – 3.60 (m, 1H), 3.59 – 3.54 (m, 1H), 3.54 – 3.47 (m, 1H), 3.46 – 3.29 (m, 1H), 3.19 (dd, J = 6.5 Hz, 14.1 Hz, 1H), 3.06 (s, 3H, major rotamer), 3.05 (s, 3H, minor rotamer), 2.96 (s, 3H), 2.95 – 2.92 (m, 1H), 2.92 – 2.85 (m, 1H), 2.71 – 2.64 (m, 1H), 2.63 – 2.56 (m, 1H), 2.29 – 2.20 (m, 1H), 1.81 (tt, J = 5.9 Hz, 13.1 Hz, 1H), 1.46 – 1.39 (m, 3H), 1.37 – 1.32 (m, 3H), 1.24 – 1.20 (m, 2H), 1.17 – 1.13 (m, 3H), 1.13 – 1.10 (m, 2H), 1.06 – 1.00 (m, 3H), 1.00 – 0.96 (m, 2H), 0.95 – 0.92 (m, 2H), 0.90 – 0.86 (m, 3H), 0.86 – 0.79 (m, 3H), 0.75 (d, J = 7.1 Hz, 3H), 0.54 (d, J = 5.5 Hz, 3H). **^{13}C NMR** (100 MHz, $CDCl_3$) δ 178.7, 173.8, 173.3, 171.9, 170.7, 170.4, 169.8, 169.6, 168.3, 168.1, 158.3, 154.3, 154.0, 129.7, 128.5, 125.4, 125.4, 113.9, 70.3, 70.0, 59.7, 58.3, 58.1, 57.9, 57.4, 55.3, 55.2, 55.1, 52.4, 50.3, 46.1, 41.5, 36.7, 36.3, 35.9, 33.7, 33.1, 31.1, 30.6, 30.5, 30.2, 29.0, 27.1, 27.1, 23.0, 21.5, 19.6, 19.3, 19.1, 18.7, 18.3, 18.2, 17.8, 17.6, 17.0, 14.2. **HRMS (ESI)** m/z: calcd for $C_{44}H_{70}N_7O_9^+$ [M+H]$^+$: 840.5230, found: 840.5233.

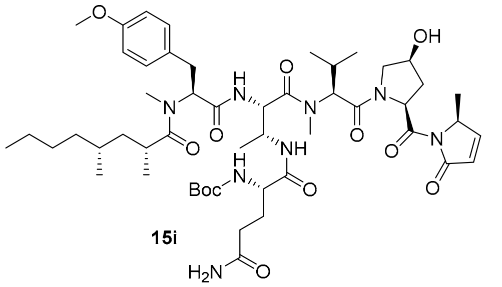

15i

Synthesis of (15i). The titled compound **15i** was obtained following the general procedure described for **15a**. The reaction was purified by column chromatography (100/1 to 20/1 DCM/MeOH) to provide compound **15i** (91 mg, 44%) as a white foamed solid. $[\boldsymbol{\alpha}]_{\boldsymbol{D}}^{\mathbf{25}}$ = −96.5 (c = 1.0, $CHCl_3$). **^{1}H NMR** (400 MHz, $CDCl_3$) δ 7.39 – 7.29 (m, 1H), 7.25 – 7.21 (m, 1H), 7.06 (d, J = 8.3 Hz, 2H), 7.03 – 6.97 (m, 1H), 6.83 – 6.77 (m, 1H), 6.75 (d, J = 8.6 Hz, 2H), 6.16 – 6.08 (m, 1H), 6.07 (d, J = 1.5 Hz, 1H), 6.05 – 5.93 (m, 1H), 5.71 – 5.55 (m, 1H), 5.41 – 5.32 (m, 1H), 5.02 – 4.93 (m, 1H), 4.90 – 4.84 (m, 1H), 4.83 – 4.74 (m, 1H), 4.47 – 4.39 (m, 1H), 4.39 – 4.31 (m, 1H), 4.11 (s, 1H), 3.98 – 3.86 (m, 1H), 3.73 (s, 3H), 3.72 – 3.66 (m, 1H), 3.66 – 3.49 (m, 1H), 3.20 – 3.12 (m, 1H), 3.08 (d, J = 3.1 Hz, 3H), 2.95 (s, 3H, minor rotamer), 2.94 (s, 3H, major rotamer), 2.90 – 2.79 (m, 1H), 2.70 – 2.51 (m, 2H), 2.41 – 2.30 (m, 2H), 2.28 – 2.20 (m, 1H), 2.19 – 2.09 (m, 1H), 2.91 – 2.00 (m, 1H), 1.91 – 1.80 (m, 1H), 1.56 – 1.46 (m, 1H), 1.45 – 1.42 (m, 2H), 1.41 (s, 9H), 1.39 – 1.27 (m, 1H), 1.26 – 1.17 (m, 3H), 1.16 – 1.11 (m, 3H), 1.17 – 1.07 (m, 2H), 1.05 – 1.01 (m, 3H), 1.01 – 0.92 (m, 3H), 0.92 – 0.87 (m, 2H), 0.87 – 0.83 (m, 3H), 0.76 (dd, J = 2.3 Hz, 6.7 Hz, 3H), 0.52 (d, J = 4.9 Hz, 3H). **^{13}C NMR** (100 MHz, $CDCl_3$) δ 178.7, 178.7, 175.8, 173.7, 172.3, 171.6, 170.9, 170.8, 170.6,

170.6, 169.8, 169.6, 168.7, 168.6, 158.3, 155.9, 154.3, 154.1, 129.6, 128.5, 125.4, 125.3, 113.9, 80.2, 71.0, 70.6, 59.3, 58.5, 58.4, 58.1, 58.0, 57.3, 56.0, 55.7, 55.1, 52.8, 52.7, 46.3, 41.6, 36.9, 36.8, 36.3, 33.6, 33.3, 31.7, 31.2, 30.6, 30.6, 30.2, 28.9, 28.3, 27.4, 27.3, 23.0, 19.5, 19.1, 18.9, 18.5, 18.4, 18.3, 17.9, 17.6, 17.0, 14.2. **HRMS (ESI)** *m*/*z*: calcd for $C_{51}H_{80}N_8O_{12}Na^+$ [M+Na]$^+$: 1019.5788, found: 1019.5786.

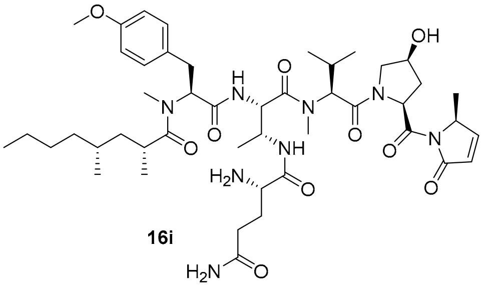

16i

Synthesis of (16i). The titled compound **16i** was obtained following the general procedure described for **16a**. The reaction was purified by column chromatography (100/1 to 20/1 DCM/MeOH) to provide compound **16i** (58 mg, 78%) as a white foamed solid. $[\alpha]_D^{25}$ = −89.5 (*c* = 1.0, $CHCl_3$). ^{1}**H NMR** (400 MHz, $CDCl_3$) *δ* 7.56 (d, *J* = 29.5 Hz, 1H), 7.25 – 7.22 (m, 1H), 7.07 (d, *J* = 8.3 Hz, 2H), 6.75 (d, *J* = 8.6 Hz, 2H), 6.06 (dd, *J* = 1.6 Hz, 6.0 Hz, 1H), 6.04 – 5.58 (m, 1H), 5.55 – 5.46 (m, 1H), 5.00 – 4.93 (m, 1H), 4.92 – 4.87 (m, 1H), 4.87 – 4.73 (m, 1H), 4.47 – 4.39 (m, 1H), 4.34 (s, 1H), 3.95 – 3.83 (m, 1H), 3.74 (s, 3H, major rotamer), 3.71 (s, 3H, minor rotamer), 3.60 – 3.51 (m, 1H), 3.51 – 3.46 (m, 1H), 3.18 – 3.12 (m, 1H), 3.10 (s, 3H, minor rotamer), 3.09 (s, 3H, major rotamer), 2.97 (s, 3H, major rotamer), 2.96 (s, 3H, minor rotamer), 2.95 – 2.90 (m, 1H), 2.67 – 2.55 (m, 2H), 2.46 – 2.33 (m, 2H), 2.29 – 2.20 (m, 1H), 2.10 – 2.01 (m, 1H), 2.01 – 1.91 (m, 1H), 1.87 – 1.74 (m, 1H), 1.42 (dd, *J* = 6.8 Hz, 18.2 Hz, 3H), 1.26 – 1.17 (m, 3H), 1.17 – 1.13 (m, 3H), 1.13 – 1.08 (m, 2H), 1.08 – 1.02 (m, 2H), 1.02 – 0.98 (m, 3H), 0.98 – 0.90 (m, 2H), 0.90 – 0.85 (m, 3H), 0.85 – 0.82 (m, 2H), 0.79 – 0.73 (m, 3H), 0.50 (d, *J* = 5.2 Hz, 3H). ^{13}**C NMR** (100 MHz, $CDCl_3$) *δ* 178.5, 173.5, 172.1, 170.5, 170.5, 170.3, 169.8, 169.6, 168.4, 168.3, 158.3, 154.3, 154.1, 129.7, 128.5, 125.4, 125.3, 113.9, 70.6, 70.3, 59.6, 58.3, 58.1, 57.9, 57.3, 55.6, 55.3, 55.1, 53.9, 52.1, 52.0, 46.1, 41.6, 36.9, 36.3, 36.0, 34.0, 33.7, 32.9, 31.0, 30.6, 30.5, 30.4, 30.3, 29.0, 27.2, 27.1, 23.0, 19.5, 19.2, 19.0, 18.8, 18.8, 18.3, 18.3, 17.9, 17.6, 17.0, 15.4, 14.2. **HRMS (ESI)** *m*/*z*: calcd for $C_{46}H_{73}N_8O_{10}^+$ [M+H]$^+$: 897.5444, found: 897.5445.

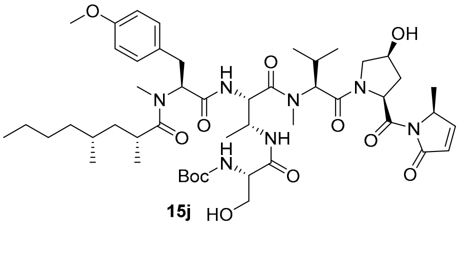

15j

Synthesis of (15j). The titled compound **15j** was obtained following the general procedure described for **15a**. The reaction was purified by column chromatography (100/1 to 20/1 DCM/MeOH) to provide compound **15j** (88 mg, 42%) as a white foamed solid. $[\boldsymbol{\alpha}]_{\boldsymbol{D}}^{\mathbf{25}}$ = −99.8 (*c* = 1.0, $CHCl_3$). **^{1}H NMR** (400 MHz, $CDCl_3$) *δ* 7.37 (d, *J* = 8.6 Hz, 1H), 7.25 – 7.22 (m, 1H), 7.06 (d, *J* = 8.1 Hz, 2H), 6.75 (d, *J* = 8.1 Hz, 2H), 6.54 (d, *J* = 8.1 Hz, 1H), 6.14 – 6.07 (m, 1H), 6.07 – 5.95 (m, 1H), 5.75 – 5.58 (m, 1H), 5.28 – 5.13 (m, 1H), 5.02 – 4.95 (m, 1H), 4.89 – 4.83 (m, 1H), 4.83 – 4.72 (m, 1H), 4.54 – 4.44(m, 1H), 4.44 – 4.37 (m, 1H), 4.20 (s, 1H), 4.10 – 4.01 (m, 1H), 4.01 – 3.94 (m, 1H), 3.74 (s, 3H), 3.73 – 3.70 (m, 1H), 3.70 – 3.50 (m, 2H), 3.13 (s, 3H, major rotamer), 3.12 (s, 3H, minor rotamer), 3.08 (d, *J* = 5.1 Hz, 1H), 2.96 (s, 3H), 2.94 – 2.87 (m, 1H), 2.73 – 2.63 (m, 1H), 2.63 – 2.52 (m, 1H), 2.31 – 2.17 (m, 1H), 1.93 (tt, *J* = 3.6 Hz, 13.6 Hz, 1H), 1.78 – 1.51 (m, 1H), 1.43 (s, 9H), 1.42 – 1.35 (m, 3H), 1.27 – 1.18 (m, 3H), 1.18 – 1.11 (m, 3H), 1.11 – 1.05 (m, 3H), 1.05 – 1.02 (m, 2H), 1.02 – 0.99 (m, 2H), 0.98 – 0.92 (m, 2H), 0.89 – 0.86 (m, 3H), 0.86 – 0.82 (m, 2H), 0.76 (d, *J* = 6.6 Hz, 3H), 0.50 (d, *J* = 5.3 Hz, 3H). **^{13}C NMR** (100 MHz, $CDCl_3$) *δ* 179.3, 174.0, 172.7, 171.3, 171.2, 170.4, 169.8, 169.6, 168.6, 168.5, 158.3, 155.8, 154.4, 154.1, 129.5, 128.2, 125.4, 125.3, 113.9, 80.4, 71.4, 71.1, 63.6, 59.3, 58.5, 58.1, 57.9, 57.6, 57.2, 56.3, 56.0, 55.1, 52.3, 46.1, 42.1, 37.0, 36.8, 36.4, 33.8, 32.9, 31.2, 30.7, 30.7, 30.3, 29.0, 28.4, 27.4, 23.0, 19.3, 19.0, 18.8, 18.4, 18.3, 18.3, 18.0, 17.6, 17.0, 14.2. **HRMS (ESI)** *m*/*z*: calcd for $C_{49}H_{77}N_7O_{12}Na^+$ $[M+Na]^+$: 978.5522, found: 978.5519.

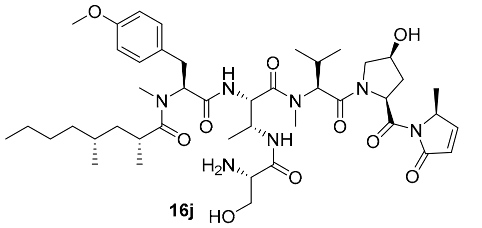

16j

Synthesis of (16j). The titled compound **16j** was obtained following the general procedure described for **16a**. The reaction was purified by column chromatography (100/1 to 20/1 DCM/MeOH) to provide compound **16j** (63 mg, 85%) as a white foamed solid. $[\boldsymbol{\alpha}]_{\boldsymbol{D}}^{\mathbf{25}}$ = −123.7 (*c* = 1.0, $CHCl_3$). **^{1}H NMR** (400 MHz, $CDCl_3$) *δ* 7.69 (s, 1H), 7.50 – 7.34 (m, 1H), 7.26 – 7.21 (m, 1H), 7.08 (d, *J* = 8.3 Hz, 2H), 6.75 (d, *J* = 8.6 Hz, 2H), 6.07 (dt, *J* = 2.0 Hz, 6.1 Hz, 1H), 5.73 – 5.55 (m, 1H), 5.39 (dd, *J* = 5.7 Hz, 11.1 Hz, 1H), 4.97 (d, *J* = 11.0 Hz, 1H), 4.92 – 4.86 (m, 1H), 4.85 – 4.74 (m, 1H), 4.49 – 4.42 (m, 1H), 4.42 – 4.35 (m, 1H), 4.00 – 3.95 (m, 1H), 3.95 – 3.87 (m, 1H), 3.74 (s, 3H), 3.73 – 3.71 (m, 1H), 3.70 – 3.67 (m, 1H), 3.67 – 3.64 (m, 1H), 3.95 – 3.58 (m, 1H), 3.55 – 3.49 (m, 1H), 3.49 – 3.44 (m, 1H), 3.44 – 3.33 (m, 1H), 3.23 – 3.14 (m, 1H), 3.13 (s, 3H, minor rotamer), 3.12 (s, 3H, major rotamer), 2.97 (s, 3H, major rotamer), 2.96 (s, 3H, minor rotamer), 2.95 – 2.91 (m, 1H), 2.68 – 2.55 (m, 2H), 2.29 – 2.19 (m, 1H), 1.95 – 1.83 (m, 1H), 1.50 – 1.43(m, 2H), 1.42 – 1.37 (m, 2H), 1.24 – 1.17 (m, 3H), 1.15 – 1.12 (m, 3H), 1.12 – 1.06 (m, 2H), 1.03 – 0.99

(m, 3H), 0.99 – 0.93 (m, 2H), 0.91 – 0.87 (m, 3H), 0.87 – 0.83 (m, 3H), 0.76 (d, *J* = 6.7 Hz, 3H), 0.51 (d, *J* = 5.1 Hz, 3H). **^{13}C NMR** (100 MHz, $CDCl_3$) *δ* 178.7, 173.6, 172.8, 172.4, 171.1, 171.0, 170.8, 170.8, 169.9, 169.6, 168.6, 168.5, 158.3, 154.4, 154.1, 129.6, 128.4, 125.4, 125.3, 113.9, 71.0, 70.8, 64.8, 59.5, 58.5, 58.5, 58.1, 58.0, 57.0, 56.3, 55.8, 55.6, 55.1, 52.7, 52.6, 45.9, 41.6, 36.9, 36.6, 36.2, 33.6, 33.2, 31.0, 30.7, 30.7, 30.2, 29.0, 27.4, 27.4, 23.1, 19.4, 19.1, 18.9, 18.4, 18.4, 17.8, 17.6, 17.0, 14.2. **HRMS (ESI)** *m/z*: calcd for $C_{44}H_{70}N_7O_{10}^+$ $[M+H]^+$: 856.5179, found: 856.5184.

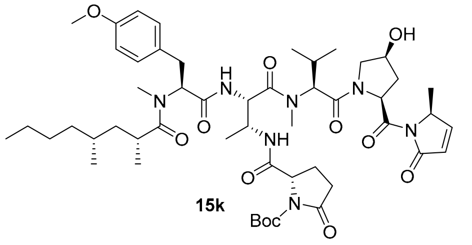

15k

Synthesis of (15k). The titled compound **15k** was obtained following the general procedure described for **15a**. The reaction was purified by column chromatography (100/1 to 20/1 DCM/MeOH) to provide compound **15k** (85 mg, 41%) as a white foamed solid. $[\boldsymbol{\alpha}]_{\boldsymbol{D}}^{25}$ = −81.7 (*c* = 1.0, $CHCl_3$). **^{1}H NMR** (400 MHz, $CDCl_3$) *δ* 7.22 (dd, *J* = 1.9 Hz, 6.0 Hz, 1H), 7.14 (d, *J* = 8.6 Hz, 1H), 7.05 (d, *J* = 8.6 Hz, 2H), 7.01 – 6.83 (m, 1H), 6.76 (d, *J* = 8.7 Hz, 2H), 6.05 (d, *J* = 5.9 Hz, 1H), 5.73 – 5.53 (m, 1H), 5.30 – 5.15 (m, 1H), 4.97 (d, *J* = 11.0 Hz, 1H), 4.88 – 4.83 (m, 1H), 4.83 – 4.74 (m, 1H), 4.62 – 4.53 (m, 1H), 4.45 – 4.39 (m, 1H), 4.38 – 4.29 (m, 1H), 3.96 – 3.85 (m, 1H), 3.75 (s, 3H, major rotamer), 3.74 (s, 3H, minor rotamer), 3.72 (s, 1H), 3.60 – 3.49 (m, 1H), 3.16 – 3.09 (m, 1H), 3.06 (s, 3H, minor rotamer), 3.05 (s, 3H, major rotamer), 3.01 (s, 3H, minor rotamer), 3.00 (s, 3H, major rotamer), 2.99 – 2.96 (m, 1H), 2.96 – 2.86 (m, 1H), 2.83 – 2.74 (m, 1H), 2.69 – 2.55 (m, 2H), 2.48 – 2.39 (m, 1H), 2.26 – 2.15 (m, 1H), 2.04 (s, 1H), 1.89 – 1.77 (m, 1H), 1.54 (s, 9H, minor rotamer), 1.53 (s, 9H, major rotamer), 1.50 – 1.42 (m, 3H), 1.41 – 1.35 (m, 2H), 1.24 – 1.18 (m, 2H), 1.18 – 1.14 (m, 3H), 1.13 (s, 3H), 1.00 (d, *J* = 7.0 Hz, 3H), 0.98 (s, 2H), 0.95 (s, 2H), 0.87 (d, *J* = 6.8 Hz, 3H), 0.76 (d, *J* = 4.1 Hz, 3H), 0.56 (d, *J* = 3.3 Hz, 3H). **^{13}C NMR** (100 MHz, $CDCl_3$) *δ* 178.6, 175.3, 173.9, 172.5, 170.8, 170.8, 169.9, 169.7, 169.5, 168.8, 168.7, 158.4, 154.2, 153.9, 149.6, 129.5, 128.4, 128.4, 125.4, 113.9, 83.9, 83.9, 70.9, 70.5, 60.3, 59.1, 59.1, 58.4, 58.1, 57.9, 56.0, 55.6, 55.1, 52.1, 52.0, 46.6, 41.6, 41.6, 36.9, 36.6, 36.2, 33.7, 33.7, 33.2, 31.7, 31.1, 30.3, 30.3, 29.0, 28.0, 27.9, 27.4, 27.3, 22.9, 22.6, 19.7, 19.4, 19.3, 19.0, 18.9, 18.5, 18.5, 18.0, 17.6, 17.1, 14.2. **HRMS (ESI)** *m/z*: calcd for $C_{51}H_{77}N_7O_{12}Na^+$ $[M+Na]^+$: 1002.5522, found: 1002.5525.

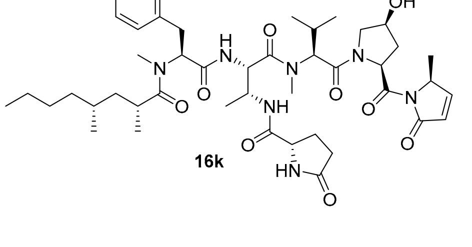

16k

Synthesis of (16k). The titled compound **16k** was obtained following the general procedure described for **16a**. The reaction was purified by column chromatography (100/1 to 20/1 DCM/MeOH) to provide compound **16k** (54 mg, 75%) as a white foamed solid. $[\alpha]_D^{25}$ = −87.9 (*c* = 1.0, $CHCl_3$). **^{1}H NMR** (400 MHz, $CDCl_3$) *δ* 7.25 – 7.21 (m, 1H), 7.08 (d, *J* = 8.4 Hz, 2H), 6.86 (d, *J* = 17.6 Hz, 1H), 6.77 (d, *J* = 8.6 Hz, 2H), 6.76 – 6.67 (m, 1H), 6.06 (dd, *J* = 1.5 Hz, 6.0 Hz, 1H), 5.71 – 5.52 (m, 1H), 5.23 – 5.14 (m, 1H), 4.99 – 4.93 (m, 1H), 4.92 – 4.88 (m, 1H), 4.86 – 4.75 (m, 1H), 4.53 – 4.46 (m, 1H), 4.39 – 4.30 (m, 1H), 4.16 – 4.10 (m, 1H), 3.94 – 3.84 (m, 1H), 3.75 (s, 3H, major rotamer), 3.74 (s, 3H, minor rotamer), 3.61 – 3.50 (m, 1H), 3.23 (s, 1H), 3.16 – 3.11 (m, 1H), 3.09 (s, 3H, major rotamer), 3.08 (s, 3H, minor rotamer), 3.02 (s, 3H, major rotamer), 3.02 (s, 3H, minor rotamer), 2.99 – 2.92 (m, 1H), 2.74 – 2.67 (m, 1H), 2.67 – 2.59 (m, 1H), 2.55 – 2.46 (m, 1H), 2.46 – 2.30 (m, 2H), 2.30 – 2.18 (m, 2H), 2.17 – 2.08 (m, 1H), 1.89 – 1.78 (m, 1H), 1.56 (t, *J* = 10.1 Hz, 1H), 1.43 (dd, *J* = 6.7 Hz, 18.2 Hz, 3H), 1.26 – 1.18 (m, 3H), 1.18 – 1.14 (m, 2H), 1.10 (d, *J* = 6.9 Hz, 3H), 1.03 (s, 2H), 1.01 (d, *J* = 6.5 Hz, 3H), 0.98 (s, 2H), 0.97 – 0.92 (m, 2H), 0.89 (d, *J* = 6.8 Hz, 3H), 0.75 (d, *J* = 6.7 Hz, 3H), 0.59 (d, *J* = 5.7 Hz, 3H). **^{13}C NMR** (100 MHz, $CDCl_3$) *δ* 179.7, 179.7, 179.1, 173.1, 171.9, 171.8, 171.8, 170.3, 170.3, 170.0, 169.8, 169.6, 168.4, 168.3, 158.5, 154.2, 154.0, 129.6, 128.3, 125.4, 125.4, 114.0, 70.4, 70.0, 59.3, 59.3, 58.5, 58.5, 58.2, 58.1, 57.9, 56.9, 55.4, 55.2, 52.0, 51.9, 46.7, 41.9, 36.9, 36.7, 36.3, 33.8, 33.1, 31.4, 30.5, 30.4, 29.4, 29.0, 27.5, 27.5, 25.5, 23.0, 19.6, 19.0, 18.5, 18.4, 18.0, 17.6, 17.1, 14.2. **HRMS (ESI)** *m/z*: calcd for $C_{46}H_{69}N_7O_{10}Na^+$ $[M+Na]^+$: 902.4998, found: 902.5001.

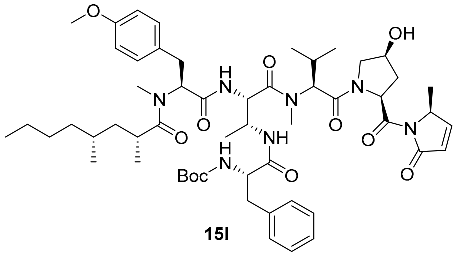

15l

Synthesis of (15l). The titled compound **15l** was obtained following the general procedure described for **15a**. The reaction was purified by column chromatography (100/1 to 20/1 DCM/MeOH) to provide compound **15l** (83 mg, 44%) as a white foamed solid. $[\alpha]_D^{25}$ = −115.9 (*c* = 1.0, $CHCl_3$). **^{1}H NMR** (400 MHz, $CDCl_3$) *δ* 7.46 – 7.33 (m, 1H), 7.32 – 7.21 (m, 1H), 7.25 – 7.22 (m, 2H), 7.22 – 7.17 (m, 2H), 7.07 (d, *J* = 8.2 Hz, 2H), 7.00 (d, *J* = 8.1 Hz, 1H), 6.76 (d, *J* = 8.1 Hz, 2H), 6.27 (d, *J* = 8.7 Hz, 1H), 6.07 (d, *J* = 6.1 Hz, 1H), 5.78 – 5.62 (m, 1H), 5.61 – 5.50 (m, 1H), 5.22 (dd, *J* = 5.7 Hz, 11.0 Hz, 1H), 4.99 (d, *J* = 10.9 Hz, 1H), 4.86 – 4.79 (m, 1H), 4.78 – 4.72 (m, 1H), 4.39 (s, 1H), 4.34 – 4.29 (m, 1H), 3.97 – 3.84 (m, 1H), 3.84 – 3.76 (m, 1H), 3.74 (s, 3H, major rotamer), 3.72 (s, 3H, minor rotamer), 3.7 (s, 3H, minor rotamer), 3.16 (d, *J* = 5.8 Hz, 1H), 3.12 (d, *J* = 6.0 Hz, 1H), 3.03 (s, 3H, major rotamer), 3.01 (s, 3H, minor rotamer), 2.98 (s, 1H), 2.95 (s,

3H, minor rotamer), 2.91 (s, 3H, major rotamer), 2.90 – 2.86 (m, 1H), 2.79 (s, 2H), 2.68 – 2.61 (m, 1H), 2.60 – 2.52 (m, 1H), 2.27 – 2.18 (m, 1H), 1.93 (t, *J* = 15.1 Hz, 1H), 1.59 (s, 1H), 1.42 (dd, *J* = 6.7 Hz, 13.9 Hz, 3H), 1.34 (s, 9H), 1.25 – 1.19 (m, 3H), 1.16 (d, *J* = 7.2 Hz, 2H), 1.14 – 1.09 (m, 2H), 1.01 (t, *J* = 5.9 Hz, 3H), 0.97 (s, 2H), 0.96 – 0.93 (m, 3H), 0.93 – 0.89 (m, 2H), 0.86 (t, *J* = 6.9 Hz, 3H), 0.76 (d, *J* = 6.5 Hz, 3H), 0.54 (d, *J* = 4.8 Hz, 3H). **^{13}C NMR** (100 MHz, $CDCl_3$) δ 178.7, 174.2, 172.9, 171.4, 170.7, 170.1, 169.8, 169.6, 168.8, 168.7, 158.4, 155.5, 154.4, 154.1, 137.1, 129.8, 129.5, 129.4, 128.5, 126.7, 125.3, 125.3, 113.9, 79.9, 71.5, 71.2, 59.2, 58.1, 57.9, 57.9, 56.6, 56.1, 55.1, 52.7, 46.9, 41.8, 38.6, 37.0, 36.4, 33.7, 33.1, 31.4, 30.4, 30.3, 29.0, 28.3, 27.2, 23.0, 19.4, 19.1, 18.9, 18.4, 18.4, 18.0, 17.6, 17.0, 14.1. **HRMS (ESI)** *m/z*: calcd for $C_{55}H_{81}N_7O_{11}Na^+$ [M+Na]$^+$: 1038.5886, found: 1038.5885.

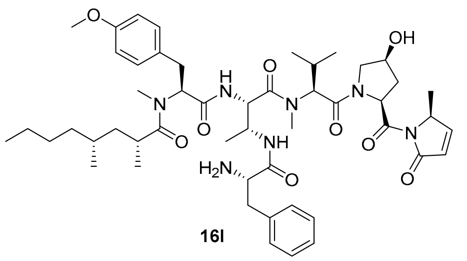

16l

Synthesis of (16l). The titled compound **16l** was obtained following the general procedure described for **16a**. The reaction was purified by column chromatography (100/1 to 20/1 DCM/MeOH) to provide compound **16l** (56 mg, 78%) as a white foamed solid. $[\alpha]_D^{25}$ =–140.3 (*c* = 1.0, $CHCl_3$). **^{1}H NMR** (400 MHz, $CDCl_3$) δ 7.31 (s, 1H), 7.29 (s, 1H), 7.28 (s, 1H), 7.24 (s, 2H), 7.22 (s, 2H), 7.08 (d, *J* = 8.1 Hz, 2H), 6.75 (d, *J* = 8.0 Hz, 2H), 6.07 (dd, *J* = 1.6 Hz, 6.0 Hz, 1H), 5.69 – 5.51 (m, 1H), 5.45 – 5.33 (m, 1H), 5.00 – 4.92 (m, 1H), 4.92 – 4.88 (m, 1H), 4.86 – 4.75 (m, 1H), 4.46 – 4.37 (m, 1H), 4.36 – 4.26 (m, 1H), 3.88 – 3.75 (m, 1H), 3.74 (s, 3H), 3.71 (s, 1H), 3.66 – 3.55 (m, 1H), 3.54 – 3.46 (m, 1H), 3.31 (s, 1H), 3.24 – 3.17 (m, 1H), 3.17 – 3.14 (m, 1H), 3.14 – 3.09 (m, 1H), 3.05 (s, 3H, major rotamer), 3.03 (s, 3H, minor rotamer), 2.98 (s, 3H, minor rotamer), 2.95 (s, 3H, major rotamer), 2.95 – 2.93 (m, 1H), 2.93 – 2.88 (m, 1H), 2.87 (s, 1H), 2.74 – 2.67 (m, 1H), 2.67 – 2.59 (m, 2H), 2.29 – 2.20 (m, 1H), 1.88 – 1.74 (m, 1H), 1.45 (d, *J* = 6.7 Hz, 2H), 1.40 (d, *J* = 6.6 Hz, 2H), 1.27 – 1.19 (m, 3H), 1.18 – 1.09 (m, 3H), 1.07 – 1.02 (m, 3H), 1.00 (d, *J* = 3.1 Hz, 2H), 0.98 (d, *J* = 2.9 Hz, 2H), 0.94 (t, *J* = 8.2 Hz, 3H), 0.88 (d, *J* = 6.9 Hz, 3H), 0.76 (d, *J* = 6.0 Hz, 3H), 0.53 (d, *J* = 5.4 Hz, 3H). **^{13}C NMR** (100 MHz, $CDCl_3$) δ 178.6, 173.3, 172.7, 172.0, 170.6, 170.4, 169.8, 169.6, 168.2, 168.1, 158.4, 154.3, 154.0, 137.3, 129.7, 129.4, 128.8, 126.9, 125.4, 125.4, 113.9, 70.3, 70.0, 59.7, 58.3, 58.1, 57.9, 57.3, 56.4, 55.4, 55.1, 52.3, 46.2, 41.6, 36.9, 36.3, 35.9, 33.7, 33.0, 31.1, 30.5, 30.5, 30.3, 29.0, 27.1, 27.1, 23.1, 19.5, 19.3, 19.1, 18.7, 18.3, 18.3, 17.9, 17.6, 17.0, 14.2. **HRMS (ESI)** *m/z*: calcd for $C_{50}H_{74}N_7O_9^+$ [M+H]$^+$: 916.5543, found: 916.5548.

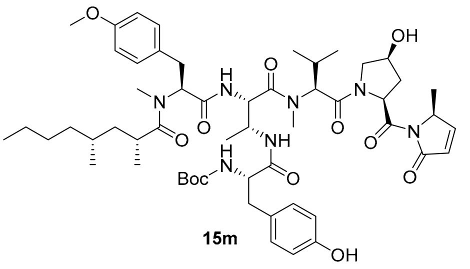
15m

Synthesis of (15m). The titled compound **15m** was obtained following the general procedure described for **15a**. The reaction was purified by column chromatography (100/1 to 20/1 DCM/MeOH) to provide compound **15m** (79 mg, 45%) as a white foamed solid. $[\boldsymbol{\alpha}]_{\boldsymbol{D}}^{\mathbf{25}}$ = −89.2 (*c* = 1.0, $CHCl_3$). **^{1}H NMR** (400 MHz, $CDCl_3$) δ 7.28 (s, 1H), 7.26 – 7.25 (m, 1H), 7.25 – 7.17 (m, 1H), 7.07 (d, *J* = 8.3 Hz, 2H), 7.01 (d, *J* = 8.0 Hz, 2H), 6.99 – 6.91 (m, 1H), 6.77 (d, *J* = 3.8 Hz, 2H), 6.75 (d, *J* = 4.3 Hz, 2H), 6.22 (d, *J* = 8.5 Hz, 1H), 6.06 (t, *J* = 5.2 Hz, 1H), 5.59 – 5.76 (m, 1H), 5.51 (d, *J* = 8.1 Hz, 1H), 5.34 (d, *J* = 11.3 Hz, 1H), 4.98 (d, *J* = 11.0 Hz, 1H), 4.88 – 4.79 (m, 1H), 4.78 – 4.70 (m, 1H), 4.42 – 4.32 (m, 1H), 4.32 – 4.24 (m, 1H), 4.21 (s, 1H), 3.96 – 3.82 (m, 1H), 3.77 (s, 3H, minor rotamer), 3.74 (s, 3H, major rotamer), 3.73 – 3.67 (m, 1H), 3.13 (dd, *J* = 6.0 Hz, 15.1 Hz, 1H), 3.03 (s, 3H, minor rotamer), 3.02 (s, 3H, major rotamer), 2.94 (s, 3H, major rotamer), 2.93 (s, 3H, minor rotamer), 2.92 (s, 1H), 2.91 – 2.87 (m, 1H), 2.87 – 2.76 (m, 1H), 2.68 – 2.50 (m, 2H), 2.29 – 2.17 (m, 1H), 1.92 (t, *J* = 15.4 Hz, 1H), 1.61 – 1.45 (m, 1H), 1.45 – 1.40 (m, 2H), 1.39 (s, 3H, minor rotamer), 1.38 (s, 3H, major rotamer), 1.28 – 1.18 (m, 3H), 1.18 – 1.09 (m, 3H), 1.08 – 1.02 (m, 2H), 1.01 (d, *J* = 7.2 Hz, 3H), 0.97 (d, *J* = 6.1 Hz, 3H), 0.94 – 0.88 (m, 3H), 0.88 – 0.85 (m, 2H), 0.85 – 0.79 (m, 2H), 0.76 (d, *J* = 6.6 Hz, 3H), 0.52 (d, *J* = 4.6 Hz, 3H). **^{13}C NMR** (100 MHz, $CDCl_3$) δ 178.8, 174.0, 172.7, 171.3, 170.3, 169.9, 169.7, 168.8, 168.7, 158.4, 155.5, 154.5, 154.2, 130.4, 129.6, 128.3, 128.0, 125.3, 125.3, 115.8, 113.9, 80.1, 71.4, 71.0, 59.4, 58.5, 58.2, 58.0, 57.4, 56.3, 55.1, 52.7, 46.6, 41.6, 38.3, 36.9, 36.4, 33.6, 33.4, 31.3, 30.5, 30.4, 30.3, 29.0, 28.3, 27.2, 23.0, 19.5, 19.1, 18.9, 18.4, 18.4, 18.3, 17.9, 17.6, 16.9, 14.2. **HRMS (ESI)** *m/z*: calcd for $C_{55}H_{81}N_7O_{12}Na^+$ [M+Na]$^+$: 1054.5835, found: 1054.5834.

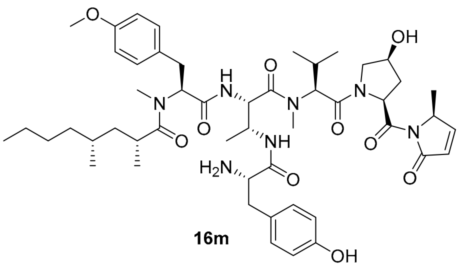
16m

Synthesis of (16m). The titled compound **16m** was obtained following the general procedure described for **16a**. The reaction was purified by column chromatography (100/1 to 20/1 DCM/MeOH) to provide compound **16m** (52 mg, 84%) as a white foamed solid. $[\boldsymbol{\alpha}]_{\boldsymbol{D}}^{\mathbf{25}}$ = −106.7 (*c* = 1.0, $CHCl_3$). **^{1}H NMR** (400 MHz, $CDCl_3$) δ 7.23 (s, 1H), 7.22 (s, 1H), 7.12 (d, *J* = 8.2 Hz,

2H), 6.99 (d, *J* = 7.8 Hz, 2H), 6.86 (d, *J* = 7.9 Hz, 2H), 6.78 (d, *J* = 8.1 Hz, 2H), 6.73 (s, 1H), 6.06 (d, *J* = 6.0 Hz, 1H), 5.63 – 5.48 (m, 1H), 5.48 – 5.40 (m, 1H), 4.95 (s, 1H), 4.91 (s, 1H), 4.84 – 4.73 (m, 1H), 4.36 (s, 1H), 4.33 (s, 1H), 3.75 (s, 3H), 3.73 (s, 1H), 3.68 (s, 1H), 3.46 – 3.38 (m, 1H), 3.17 (s, 1H), 3.13 (d, *J* = 5.9 Hz, 1H), 3.10 (d, *J* = 5.7 Hz, 1H), 3.04 (s, 3H), 3.03 (s, 3H), 3.00 (s, 1H), 2.95 (s, 1H), 2.92 (s, 1H), 2.89 (s, 1H), 2.68 (s, 2H), 2.53 (s, 1H), 2.30 – 2.21 (m, 1H), 1.78 – 1.65 (m, 1H), 1.55 (s, 1H), 1.41 (dd, *J* = 6.7 Hz, 19.9 Hz, 3H), 1.27 – 1.21 (m, 1H), 1.17 (s, 3H), 1.08 (d, *J* = 6.6 Hz, 3H), 1.05 – 1.00 (m, 3H), 1.00 – 0.97 (m, 2H), 0.95 (d, *J* = 6.2 Hz, 2H), 0.90 (d, *J* = 7.0 Hz, 3H), 0.88 – 0.85 (m, 2H), 0.78 – 0.72 (m, 3H), 0.54 (d, *J* = 5.0 Hz, 3H). **^{13}C NMR** (100 MHz, $CDCl_3$) δ 179.0, 172.3, 170.9, 170.4, 169.8, 169.6, 167.7, 167.6, 158.5, 155.8, 154.2, 154.0, 130.7, 129.7, 128.1, 125.4, 125.4, 116.1, 114.0, 69.0, 68.7, 60.0, 58.4, 58.3, 58.1, 57.9, 57.0, 56.5, 55.1, 54.3, 54.1, 52.2, 46.5, 41.6, 37.0, 36.2, 35.8, 33.7, 33.3, 31.0, 30.5, 30.5, 30.3, 29.0, 27.0, 23.1, 19.6, 19.3, 19.1, 18.6, 18.2, 18.1, 18.0, 17.6, 17.1, 14.2. **HRMS (ESI)** *m/z*: calcd for $C_{50}H_{74}N_7O_{10}^+$ [M+H]$^+$: 932.5492, found: 932.5496.

2. ¹H and ¹³C NMR Spectra

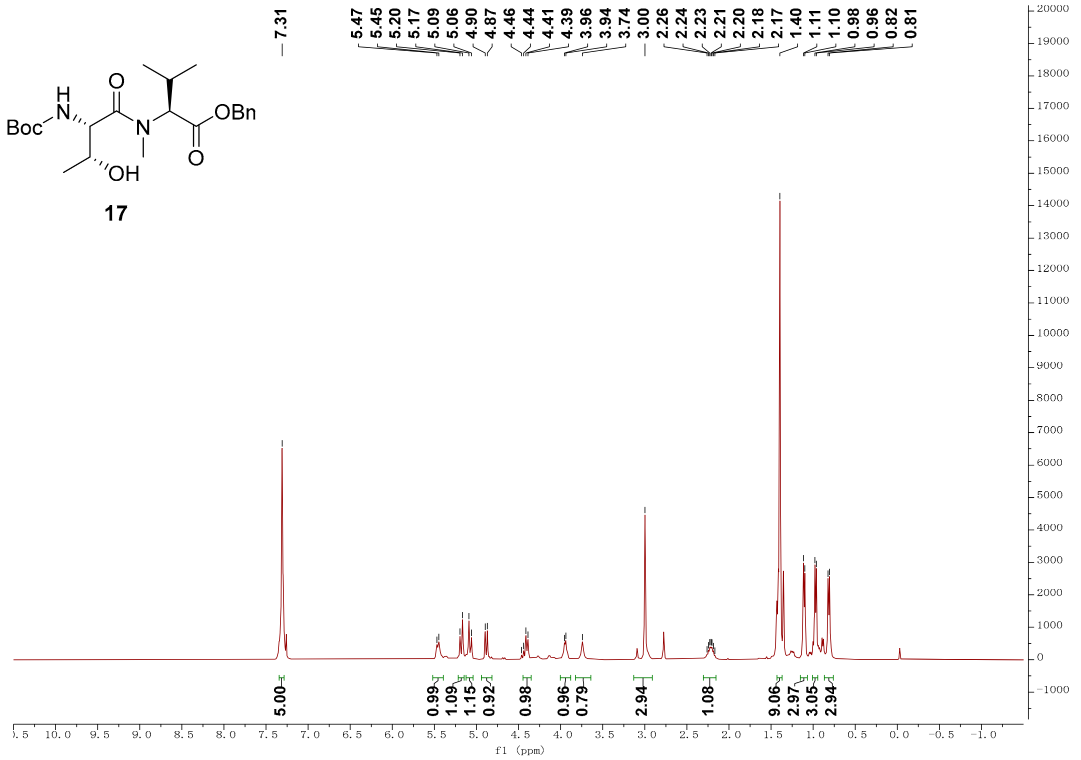

Figure S1. ¹H NMR spectrum of compound **17** in $CDCl_3$ (400 MHz)

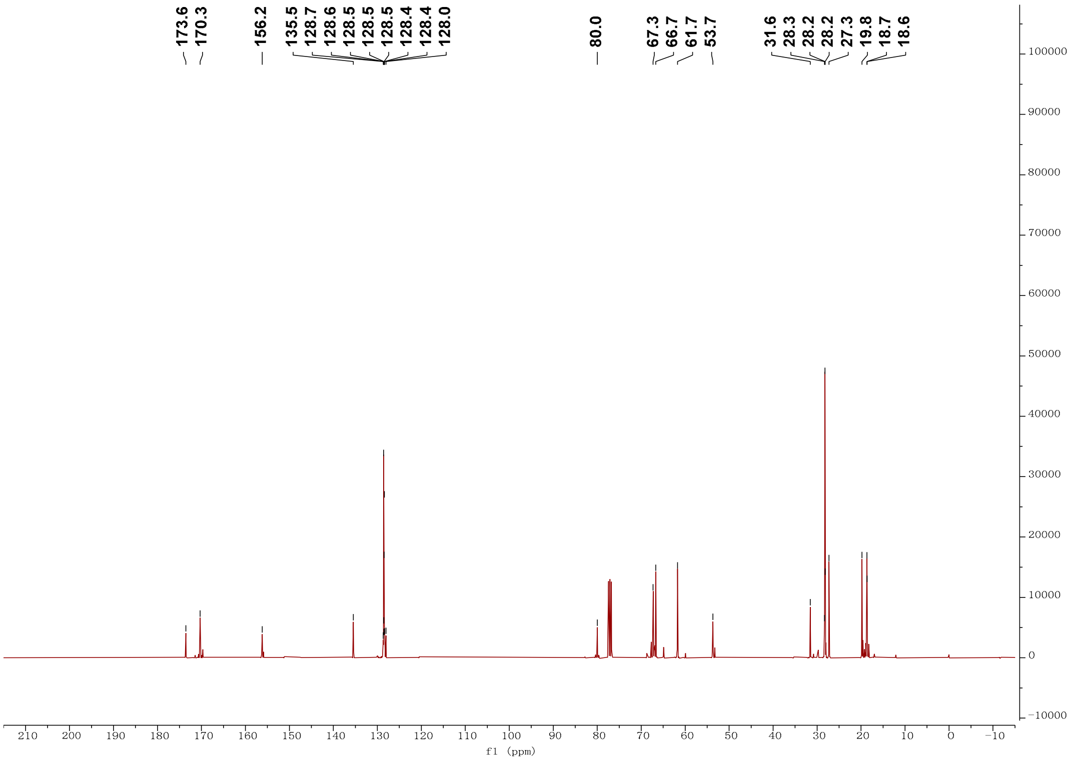

Figure S2. ¹³C NMR spectrum of compound **17** in $CDCl_3$ (100 MHz)

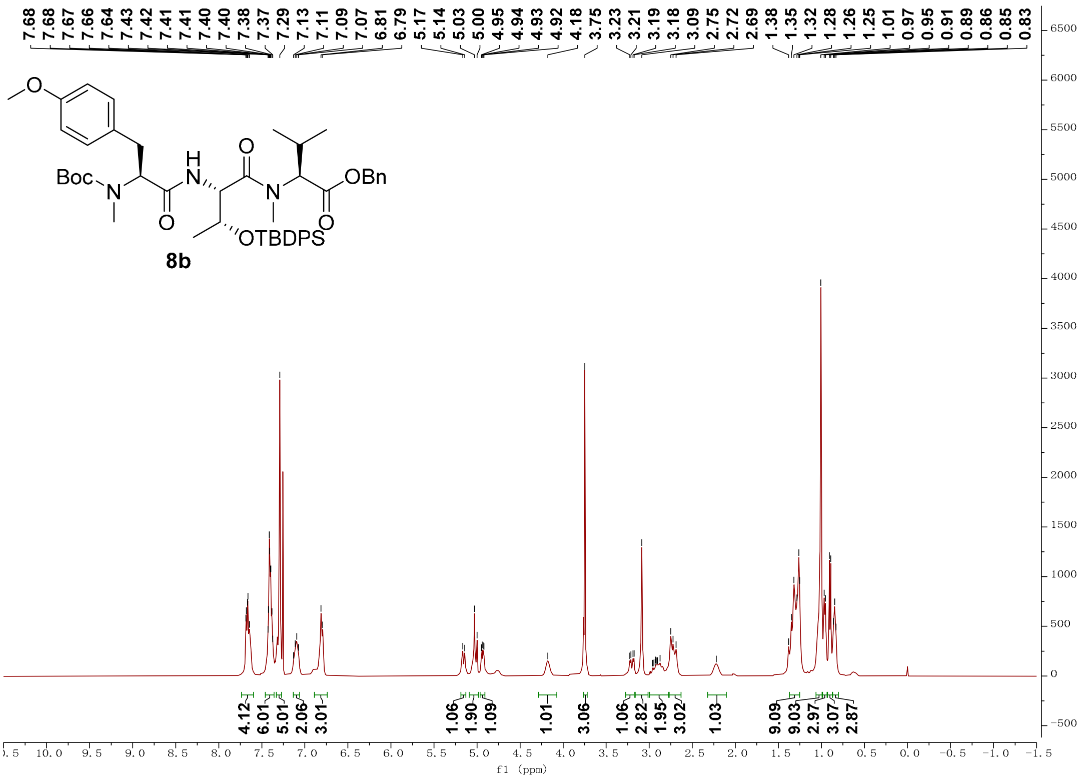

Figure S3. ^{1}H NMR spectrum of compound **8b** in $CDCl_3$ (400 MHz)

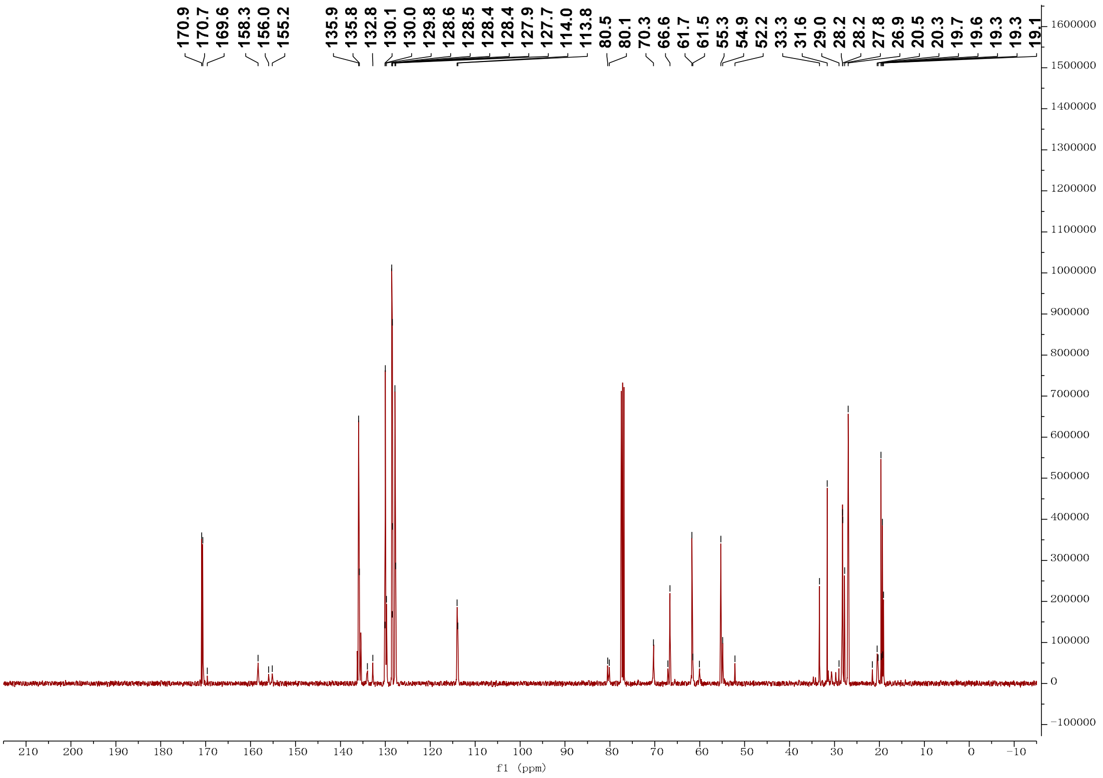

Figure S4. ^{13}C NMR spectrum of compound **8b** in $CDCl_3$ (100 MHz)

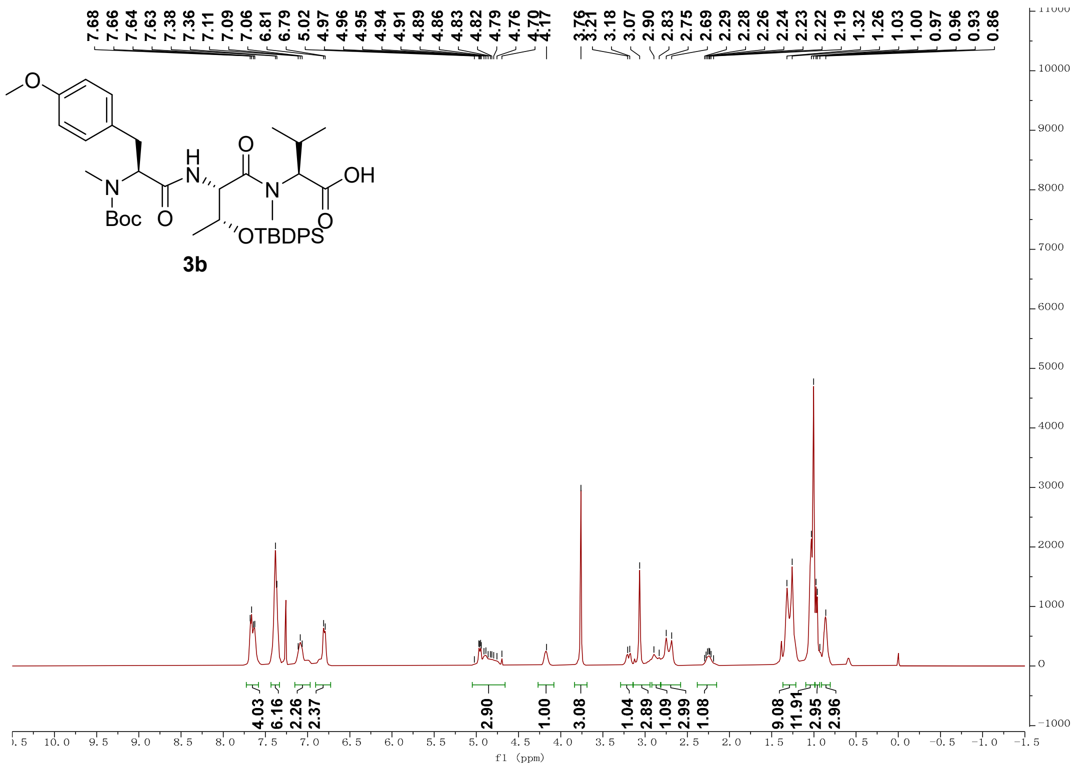

Figure S5. ^{1}H NMR spectrum of compound **3b** in $CDCl_3$ (400 MHz)

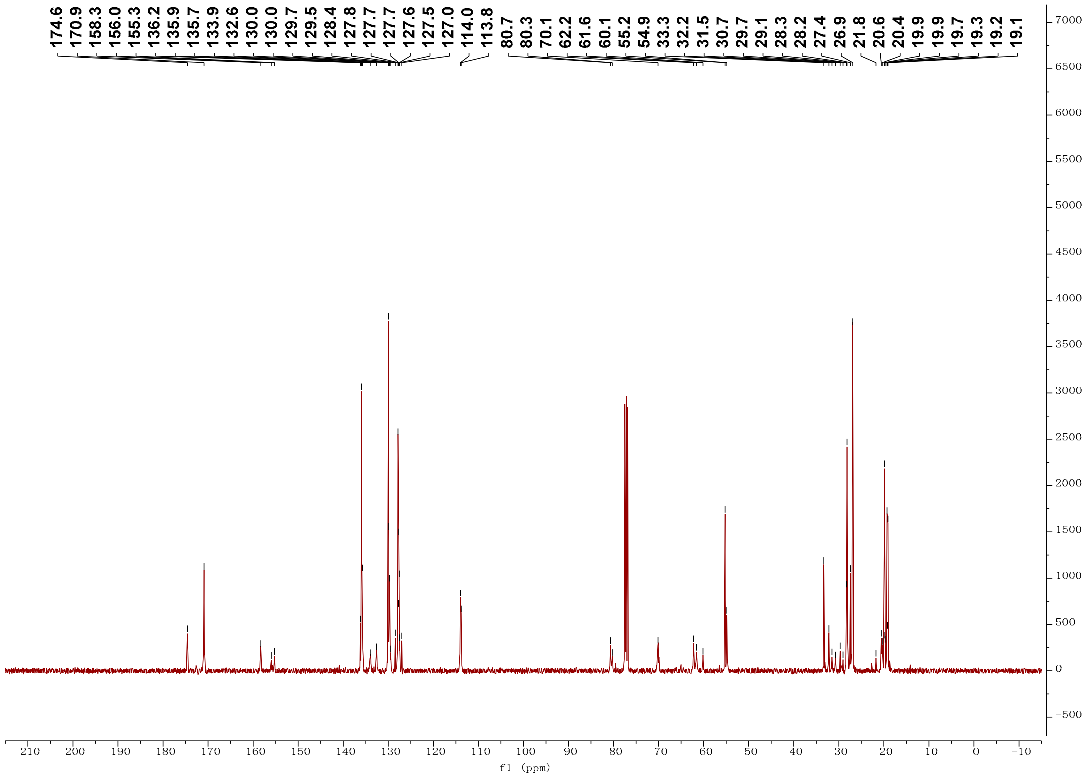

Figure S6. ^{13}C NMR spectrum of compound **3b** in $CDCl_3$ (100 MHz)

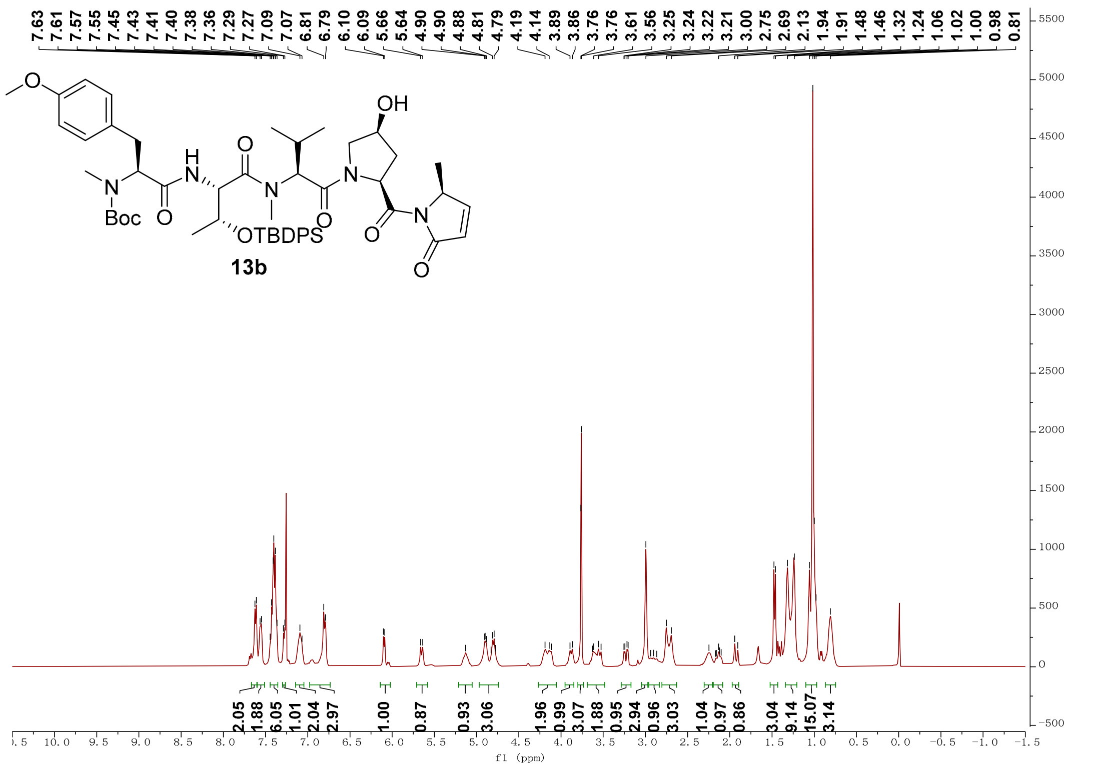

Figure S7. ^{1}H NMR spectrum of compound **13b** in $CDCl_3$ (400 MHz)

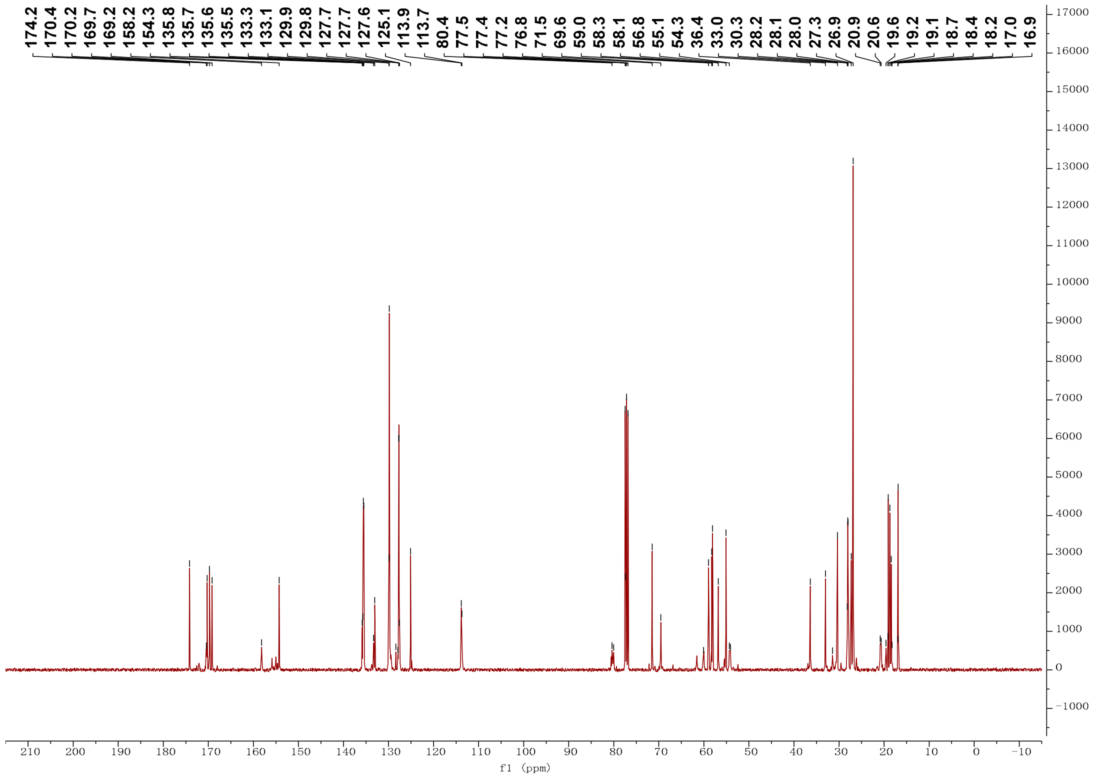

Figure S8. ^{13}C NMR spectrum of compound **13b** in $CDCl_3$ (100 MHz)

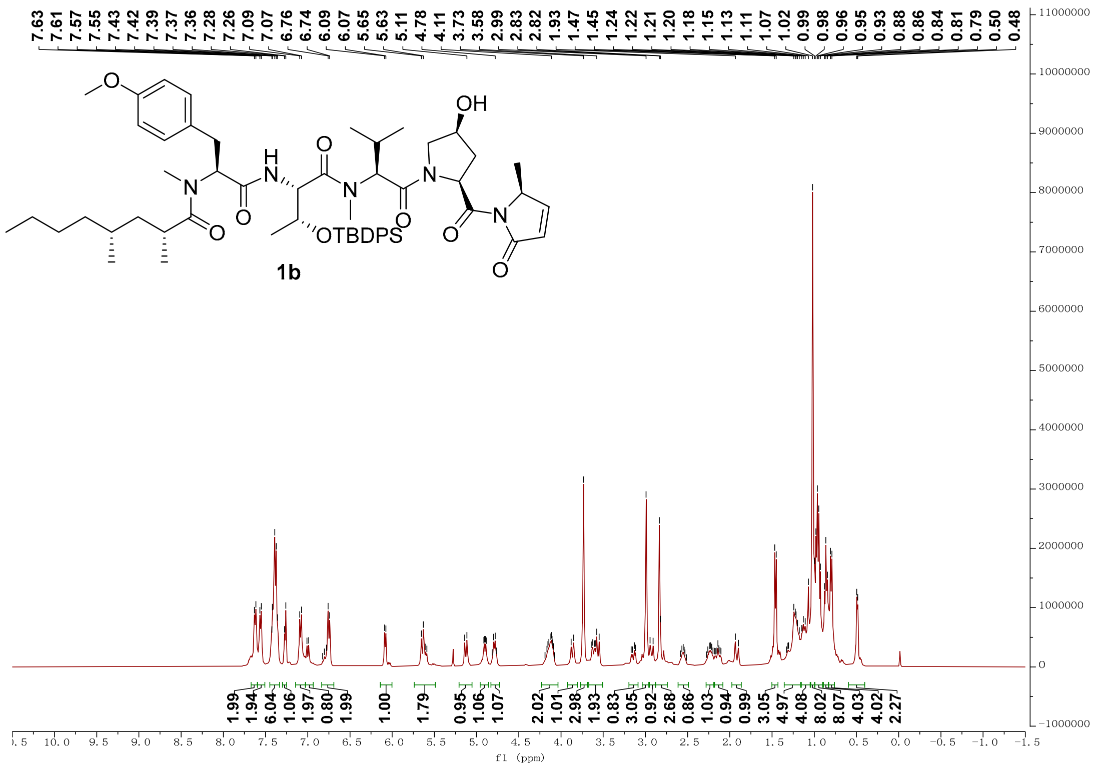

Figure S9. ^{1}H NMR spectrum of compound **1b** in $CDCl_3$ (400 MHz)

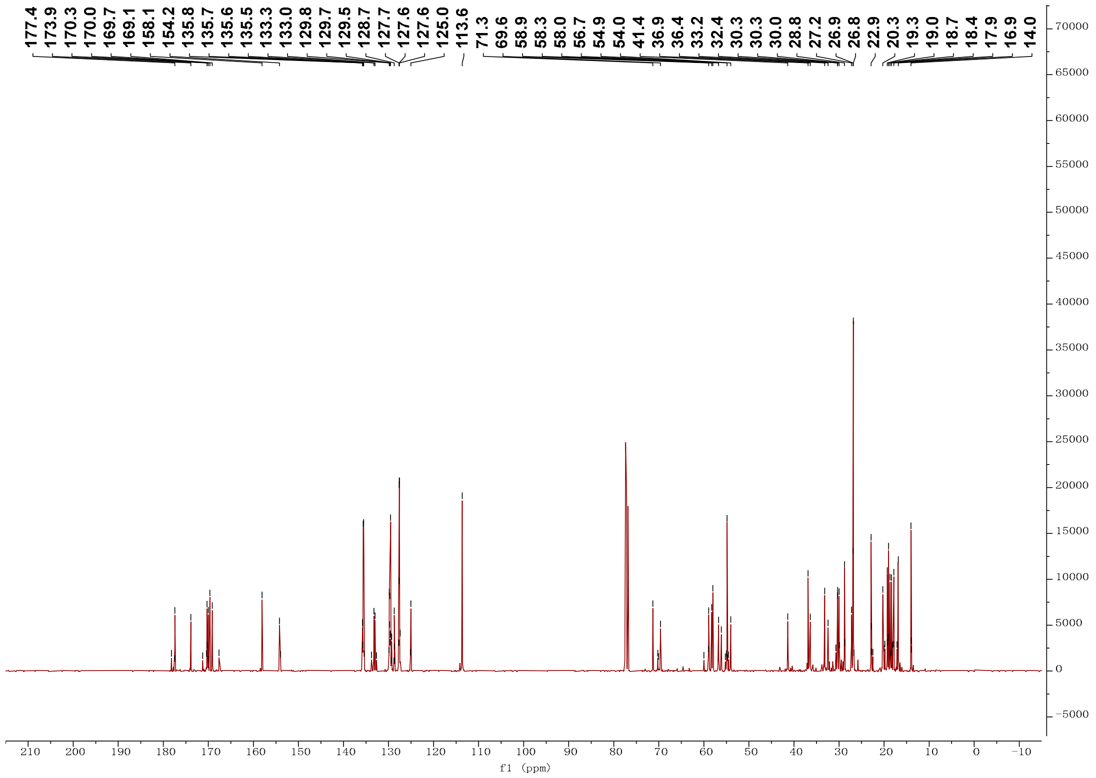

Figure S10. ^{13}C NMR spectrum of compound **1b** in $CDCl_3$ (100 MHz)

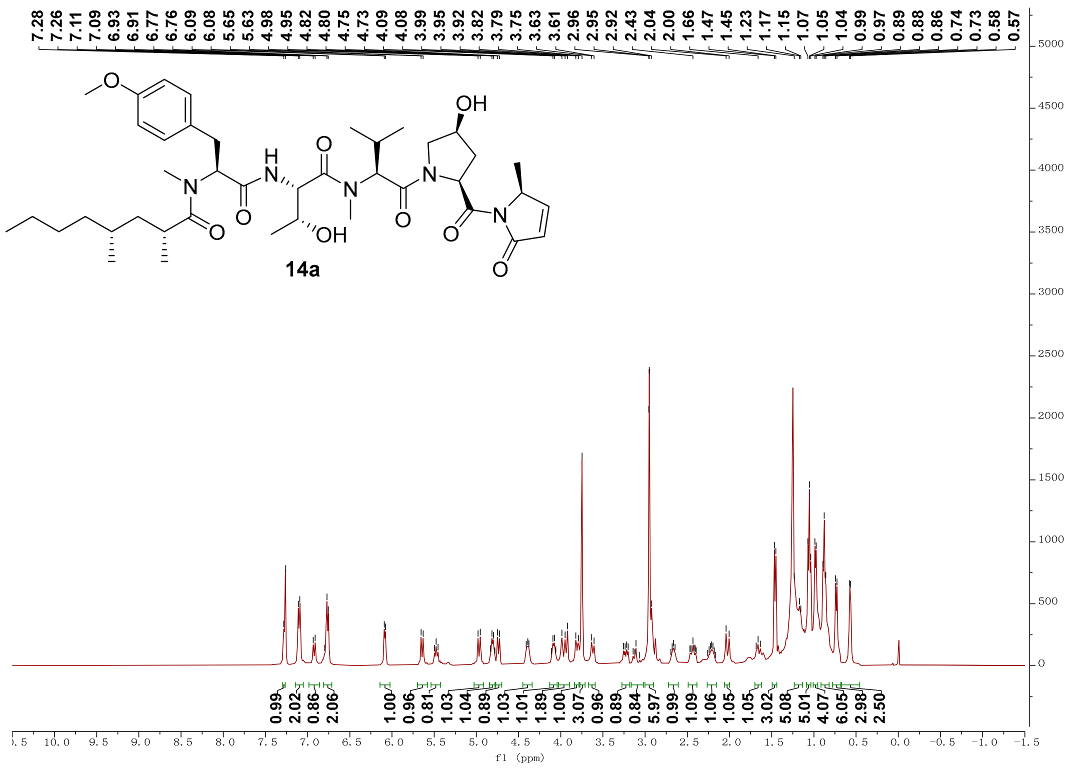

Figure S11. ^{1}H NMR spectrum of compound **14a** in $CDCl_3$ (400 MHz)

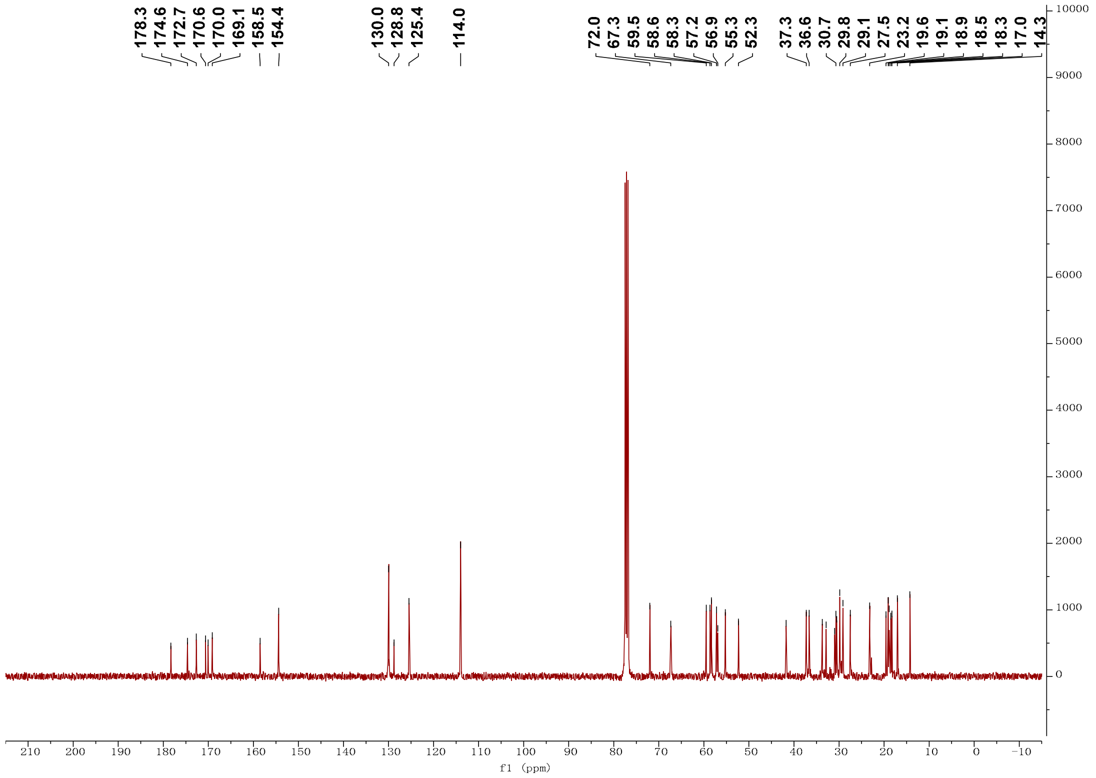

Figure S12. ^{13}C NMR spectrum of compound **14a** in $CDCl_3$ (100 MHz)

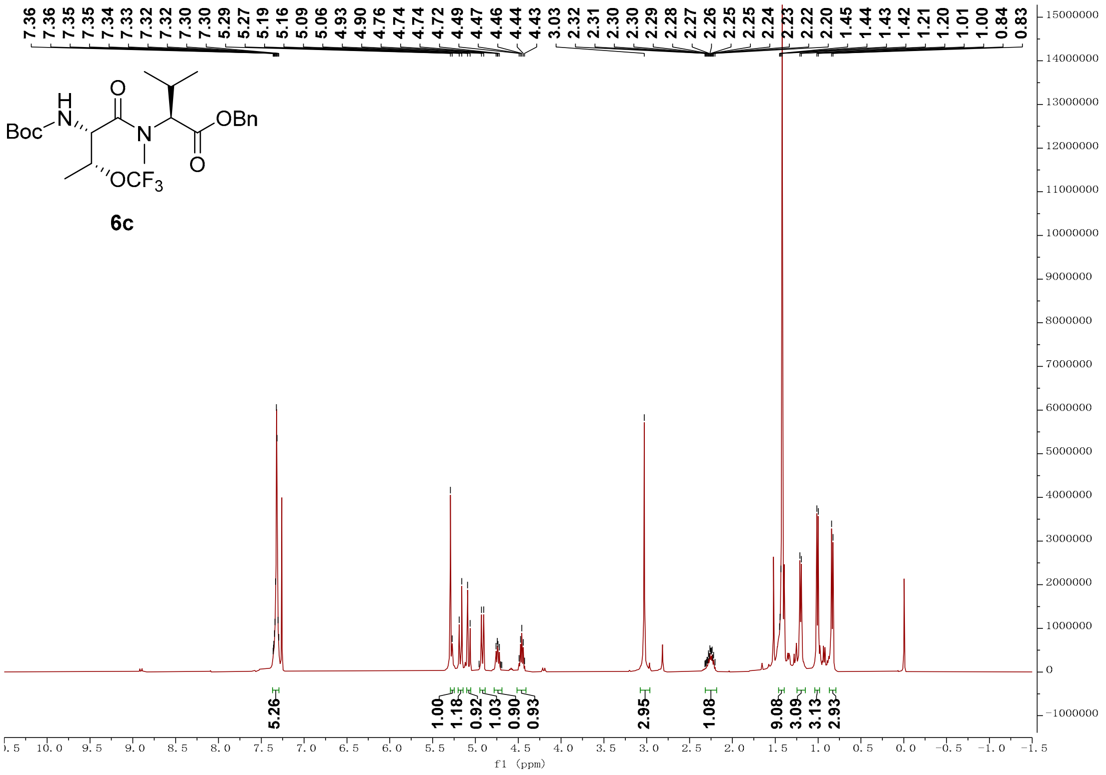

Figure S13. ^{1}H NMR spectrum of compound **6c** in $CDCl_3$ (400 MHz)

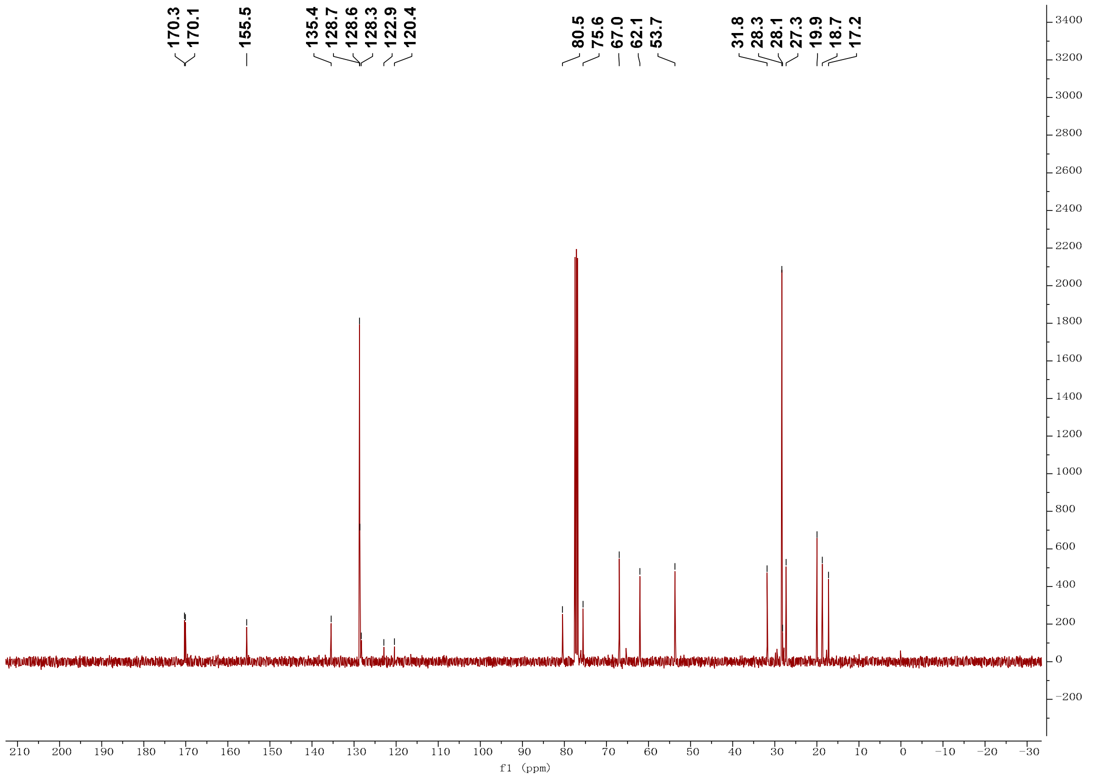

Figure S14. ^{13}C NMR spectrum of compound **6c** in $CDCl_3$ (100 MHz)

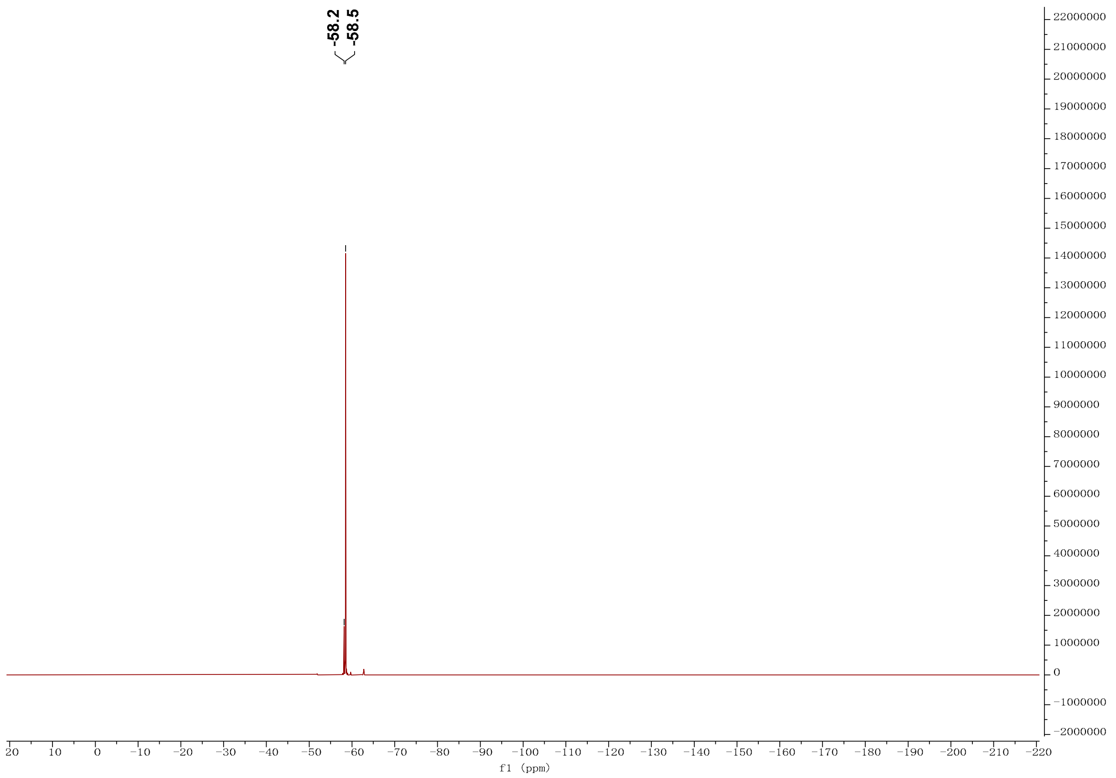

Figure S15. ^{19}F NMR spectrum of compound **6c** in $CDCl_3$ (376 MHz)

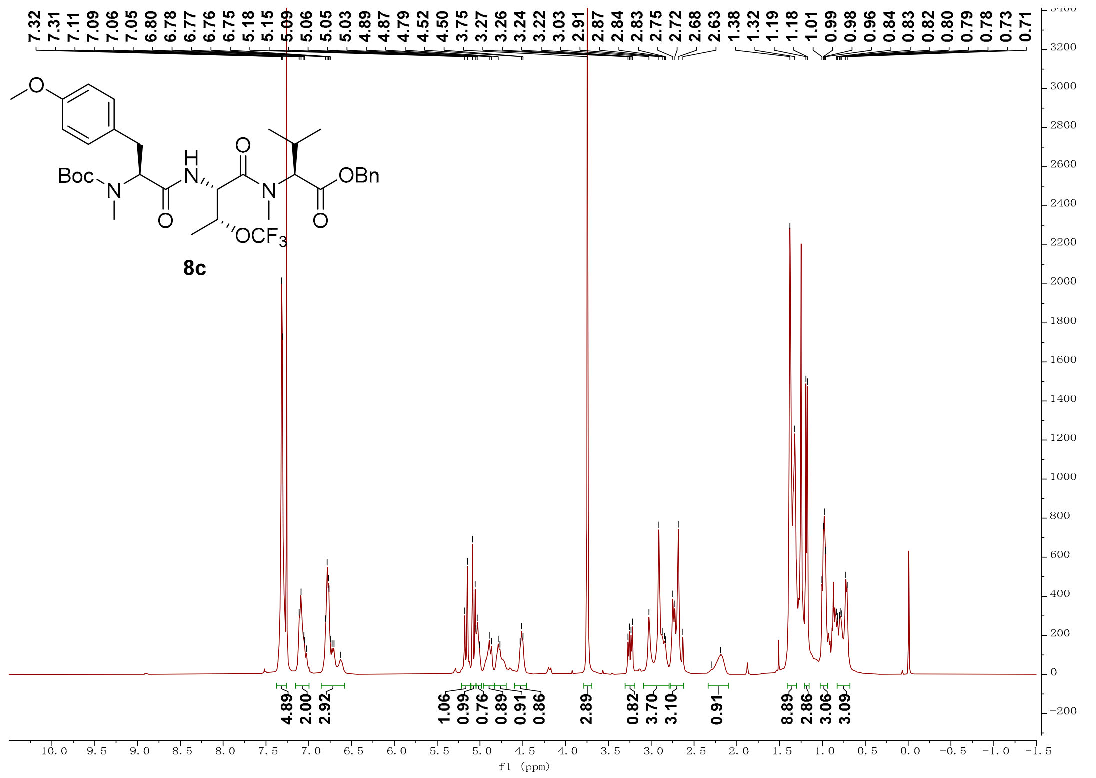

Figure S16. ^{1}H NMR spectrum of compound **8c** in $CDCl_3$ (400 MHz)

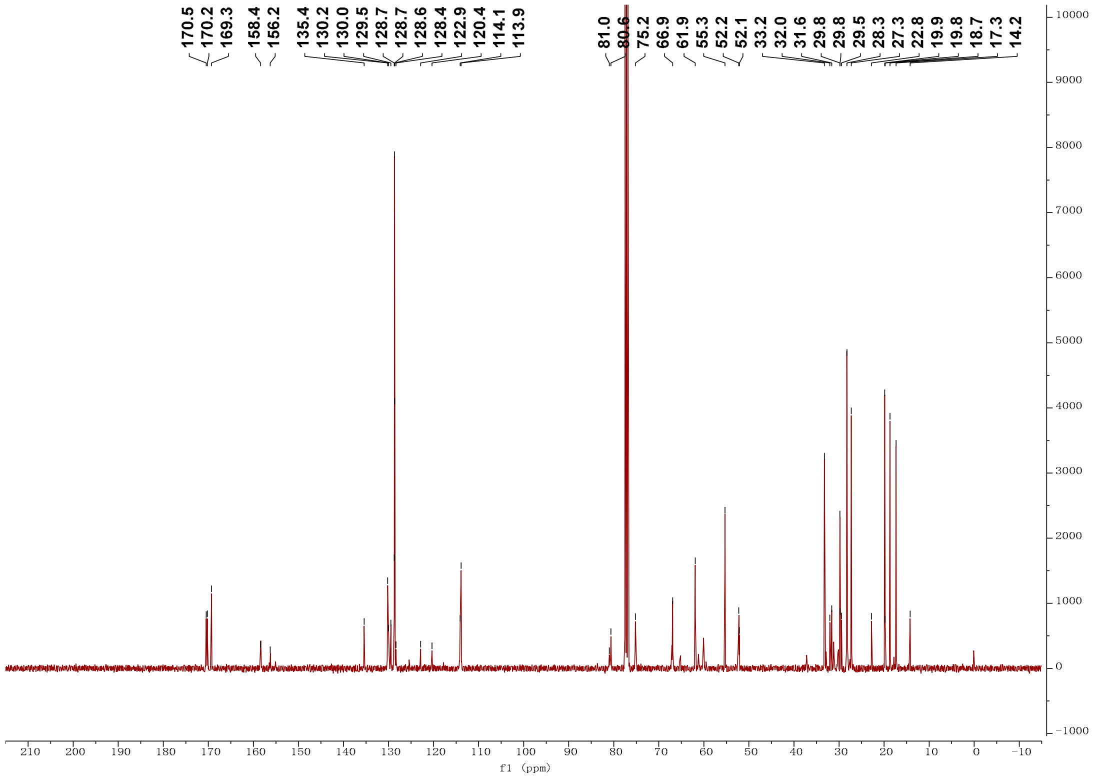

Figure S17. ^{13}C NMR spectrum of compound **8c** in $CDCl_3$ (100 MHz)

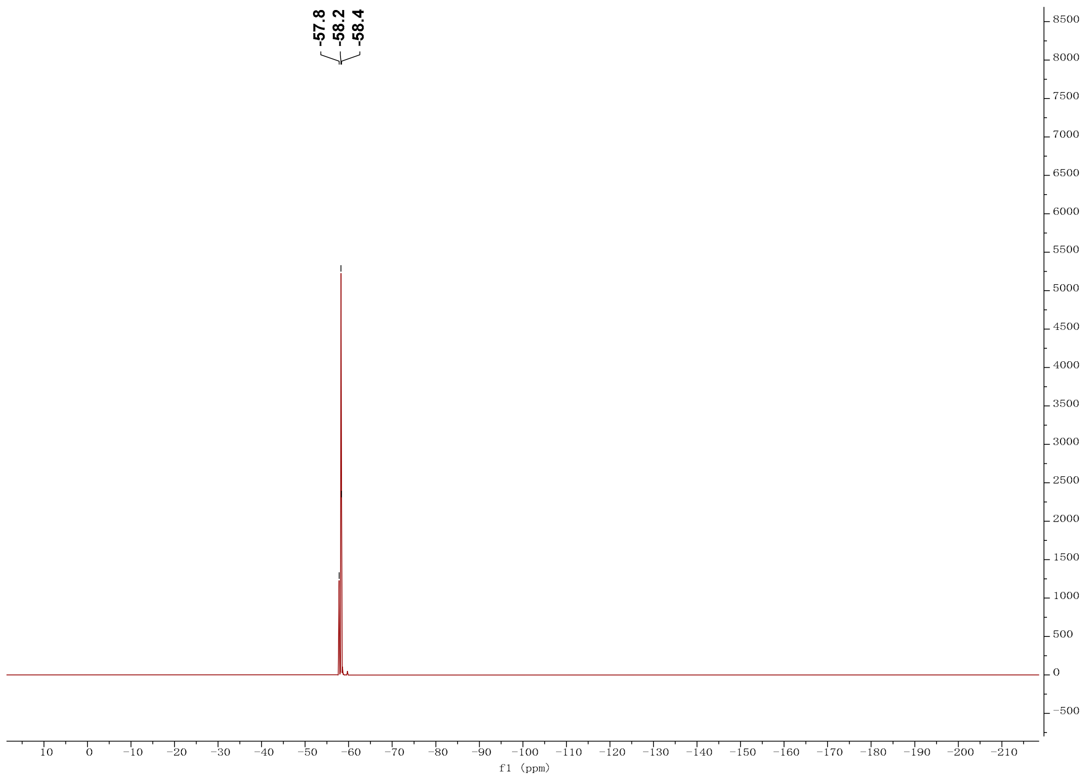

Figure S18. ^{19}F NMR spectrum of compound **8c** in $CDCl_3$ (376 MHz)

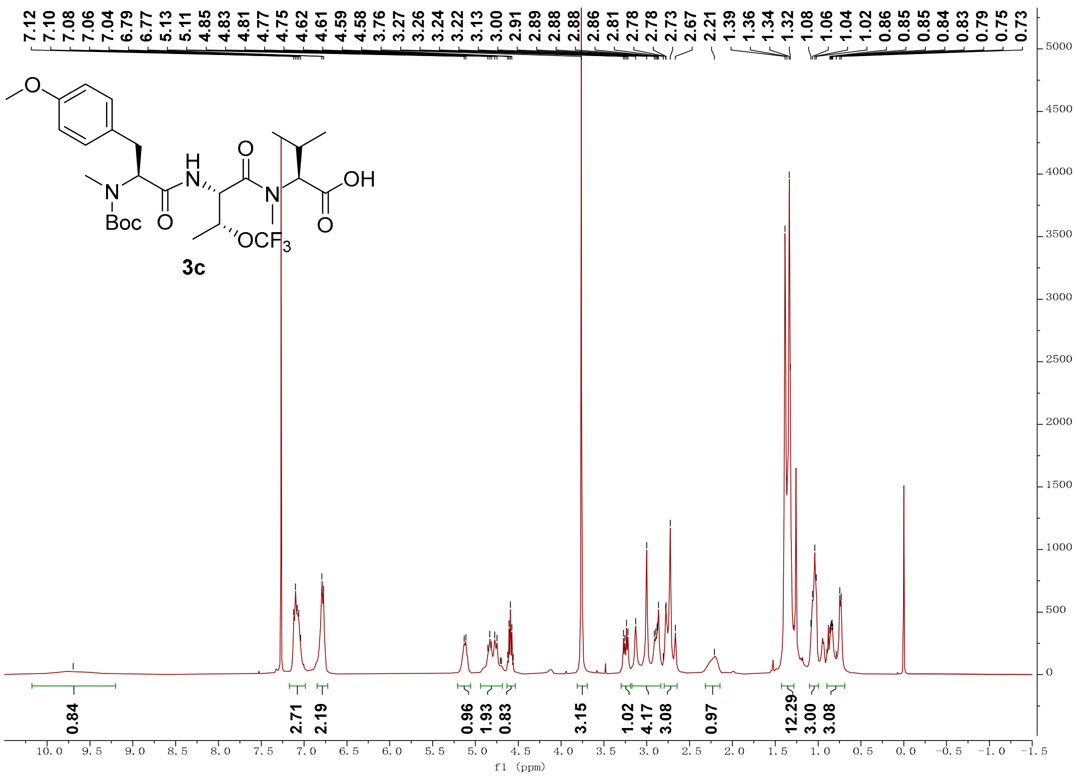

Figure S19. ^{1}H NMR spectrum of compound **3c** in $CDCl_3$ (400 MHz)

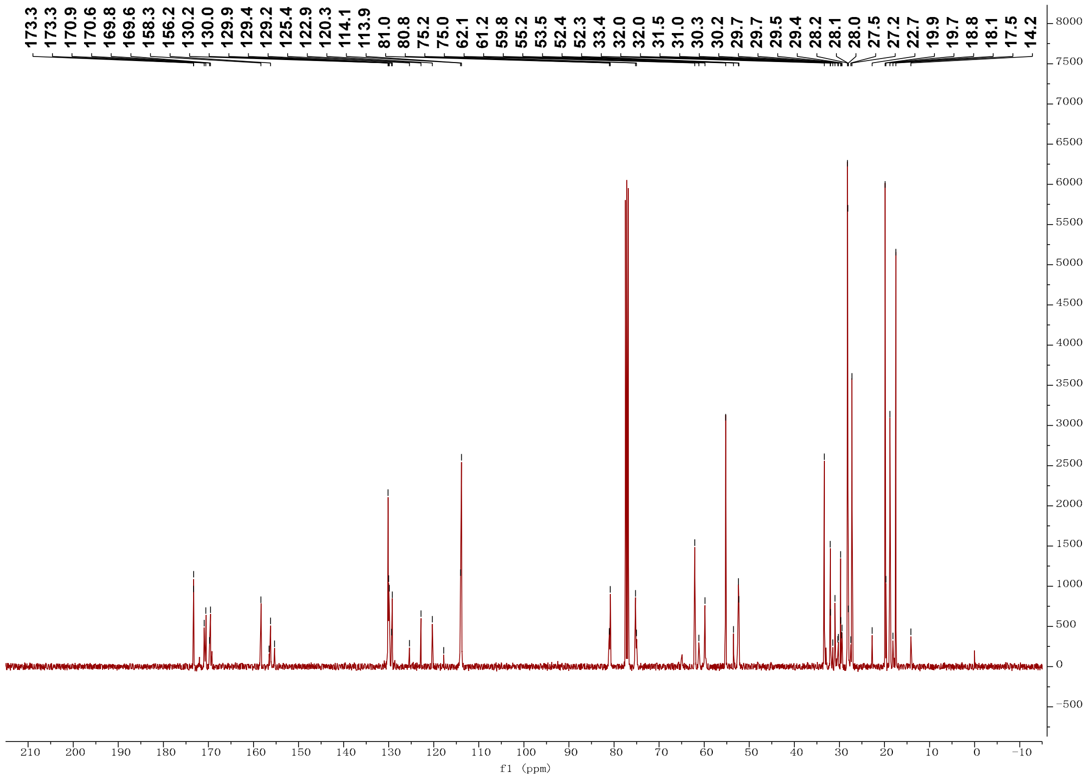

Figure S20. ^{13}C NMR spectrum of compound **3c** in $CDCl_3$ (100 MHz)

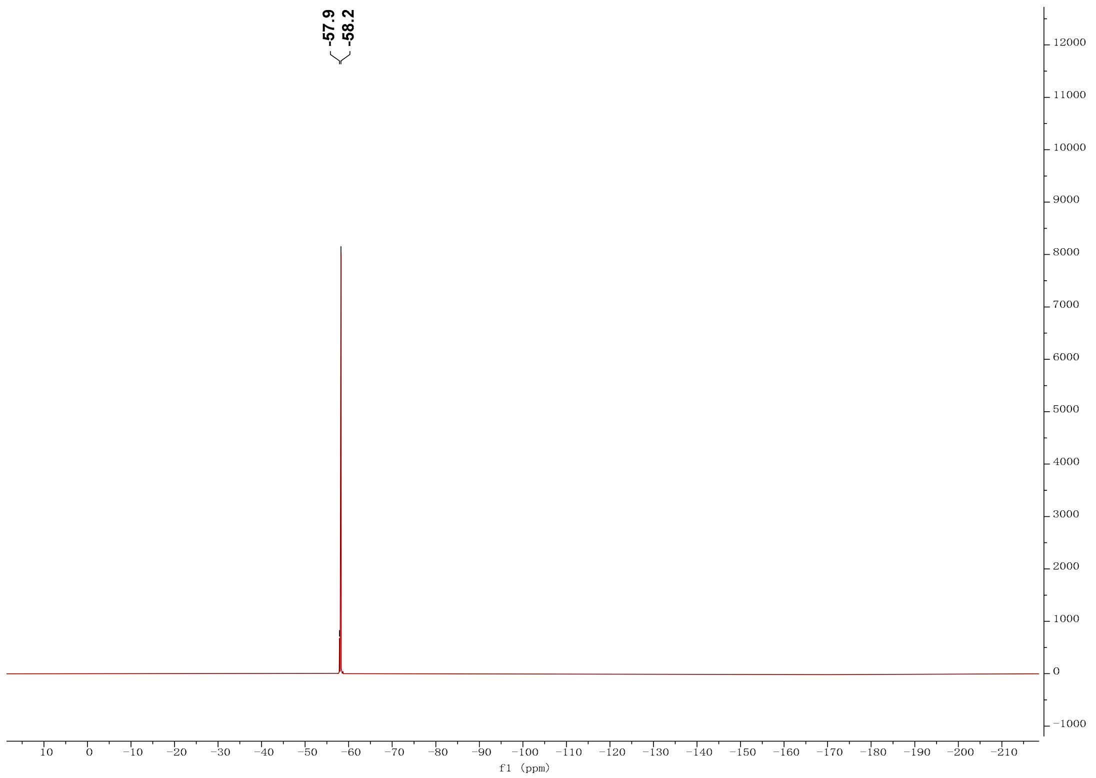

Figure S21. ^{19}F NMR spectrum of compound **3c** in $CDCl_3$ (376 MHz)

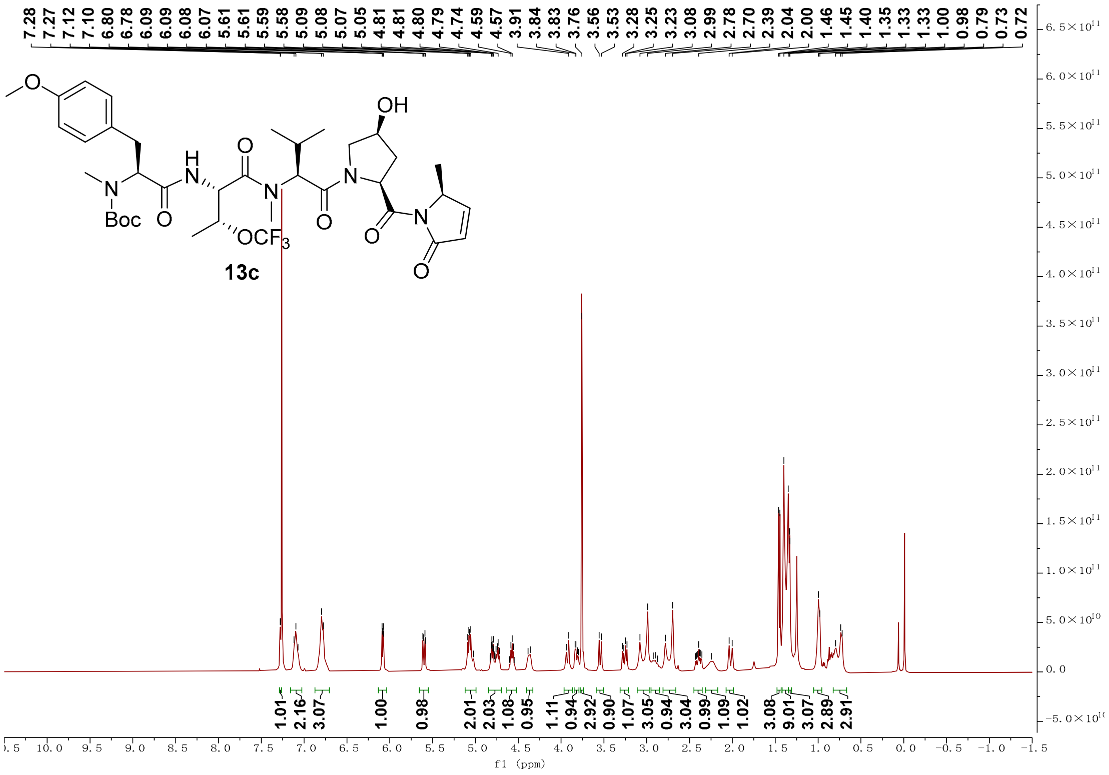

Figure S22. ^{1}H NMR spectrum of compound **13c** in $CDCl_3$ (400 MHz)

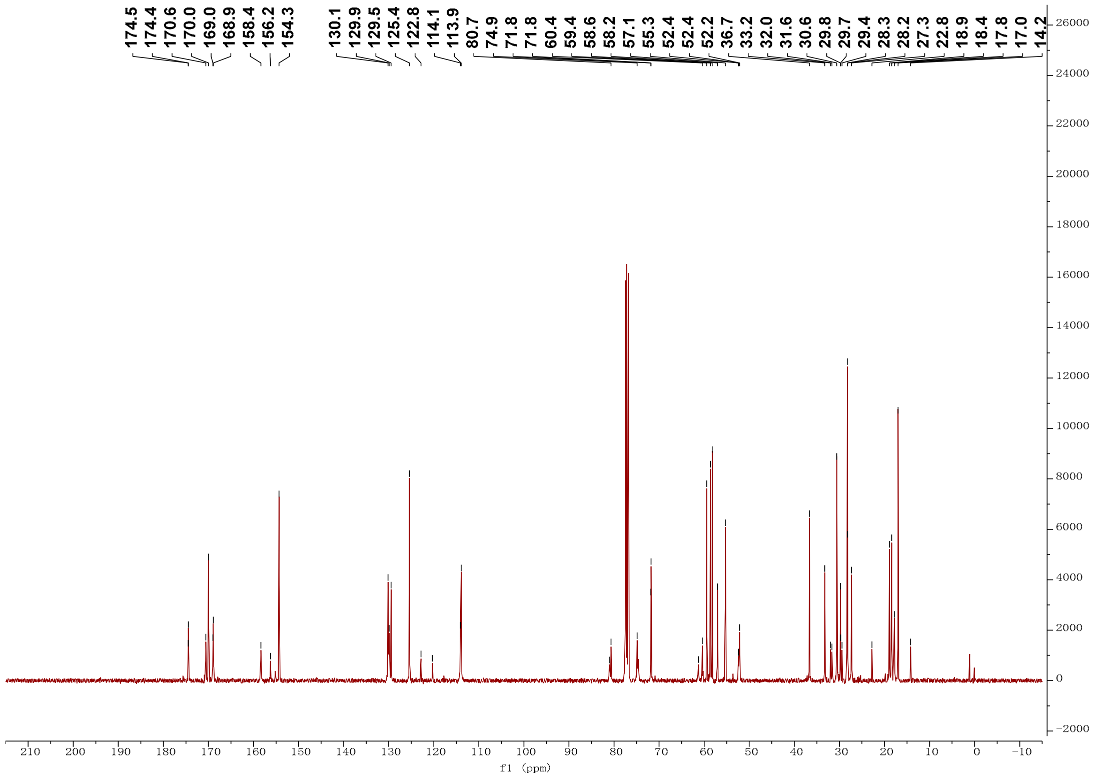

Figure S23. ^{13}C NMR spectrum of compound **13c** in $CDCl_3$ (100 MHz)

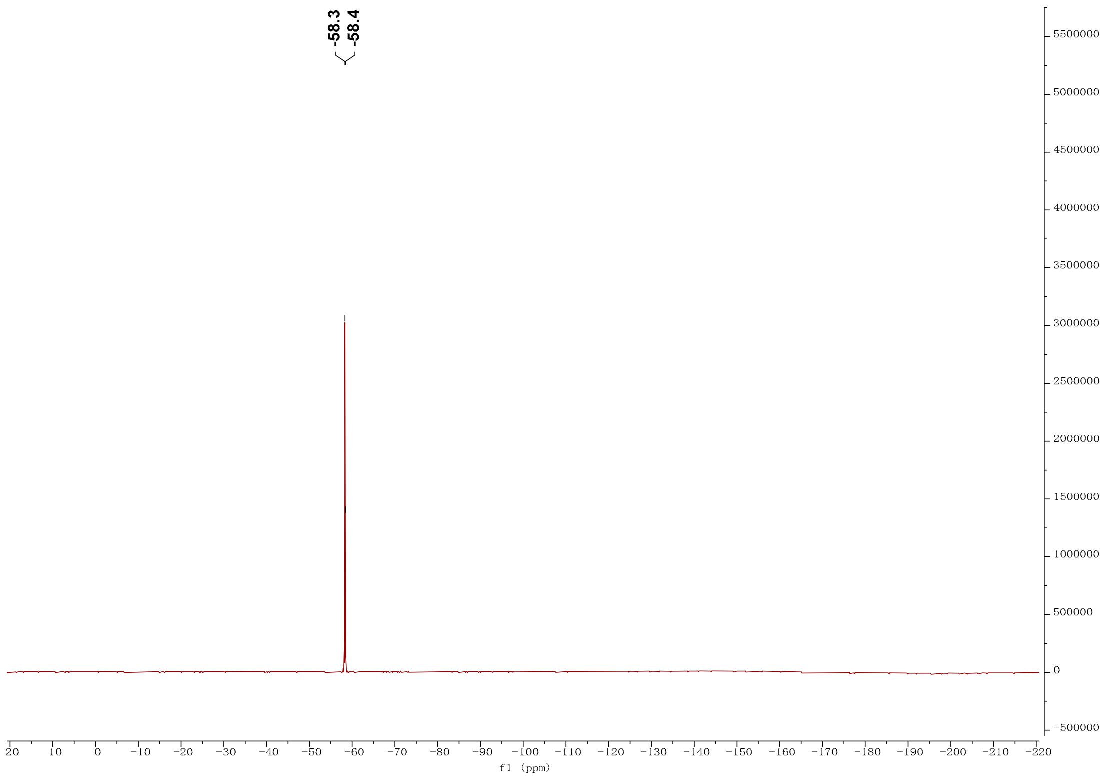

Figure S24. ^{19}F NMR spectrum of compound **13c** in $CDCl_3$ (376 MHz)

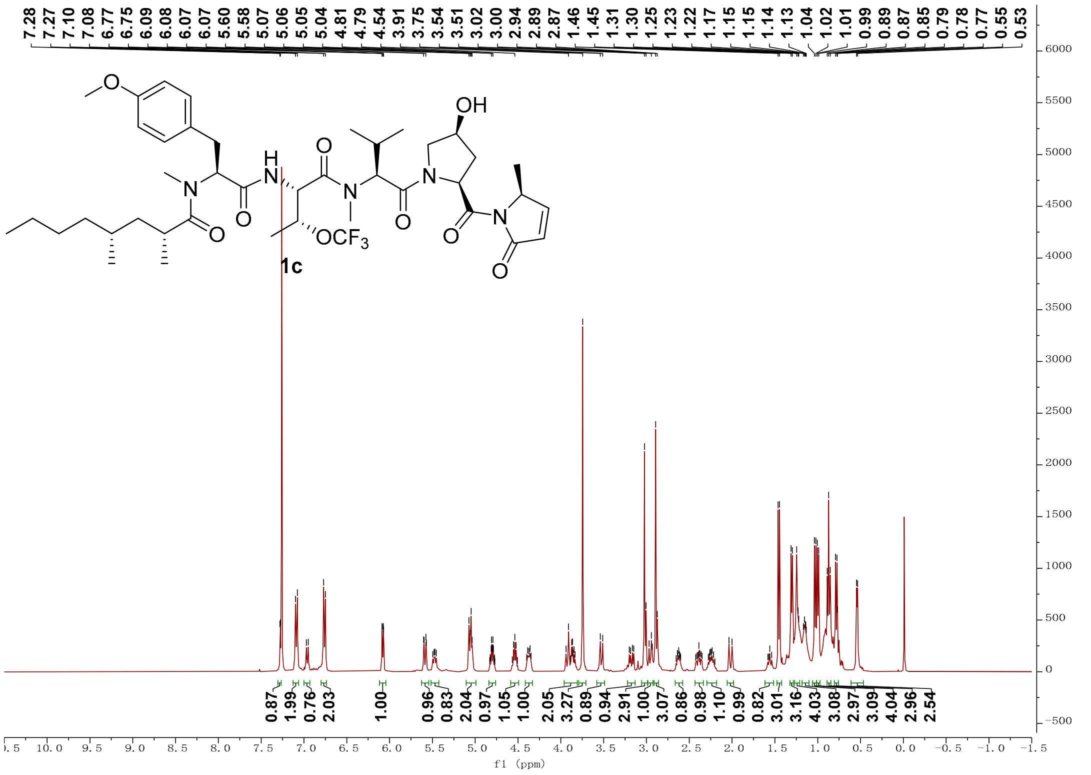

Figure S25. ^{1}H NMR spectrum of compound **1c** in $CDCl_3$ (400 MHz)

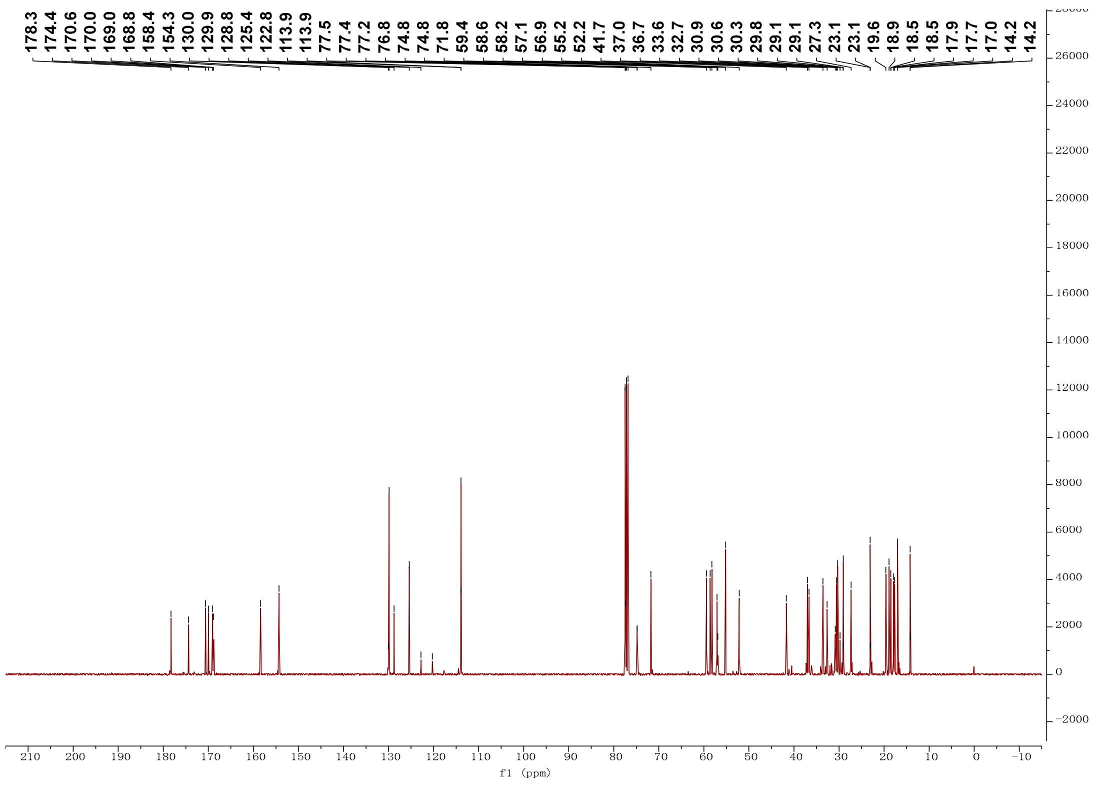

Figure S26. ^{13}C NMR spectrum of compound **1c** in $CDCl_3$ (100 MHz)

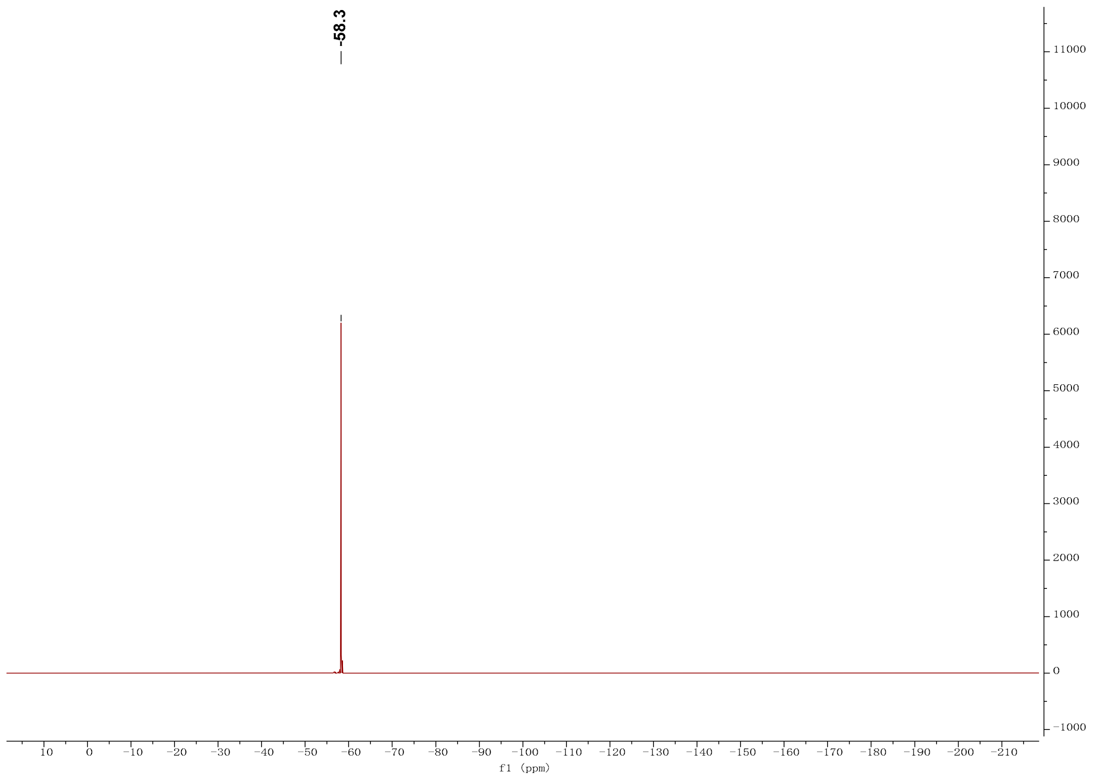

Figure S27. ^{19}F NMR spectrum of compound **1c** in $CDCl_3$ (376 MHz)

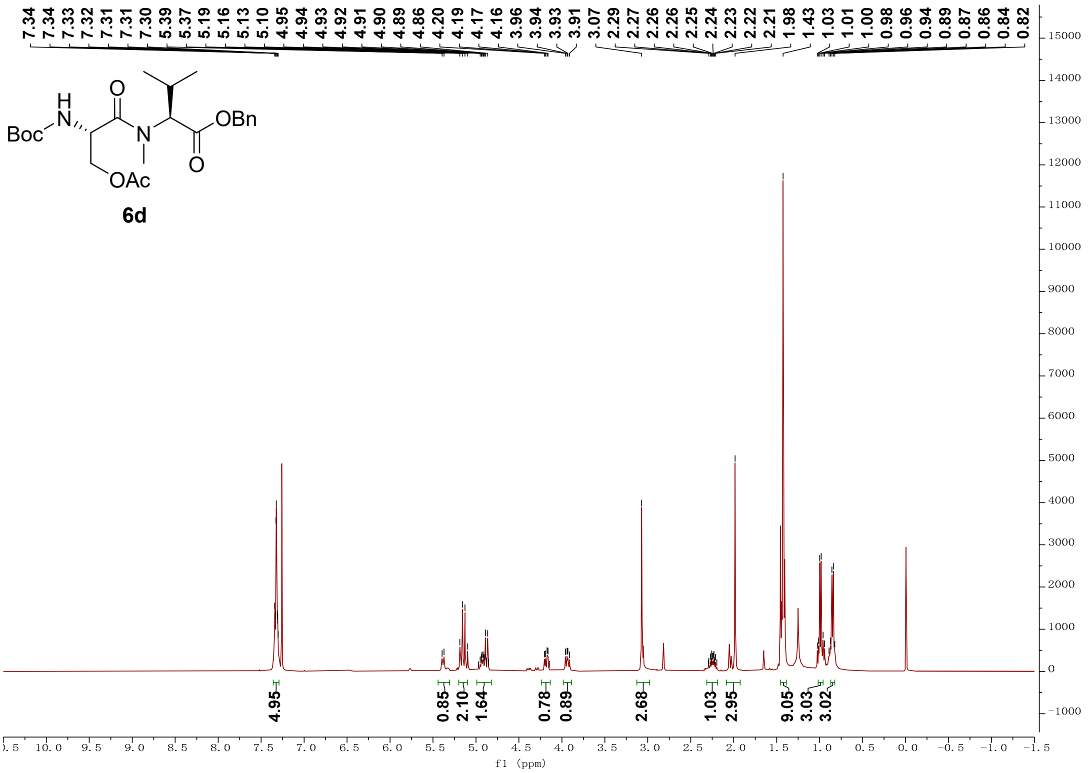

Figure S28. ^{1}H NMR spectrum of compound **6d** in $CDCl_3$ (400 MHz)

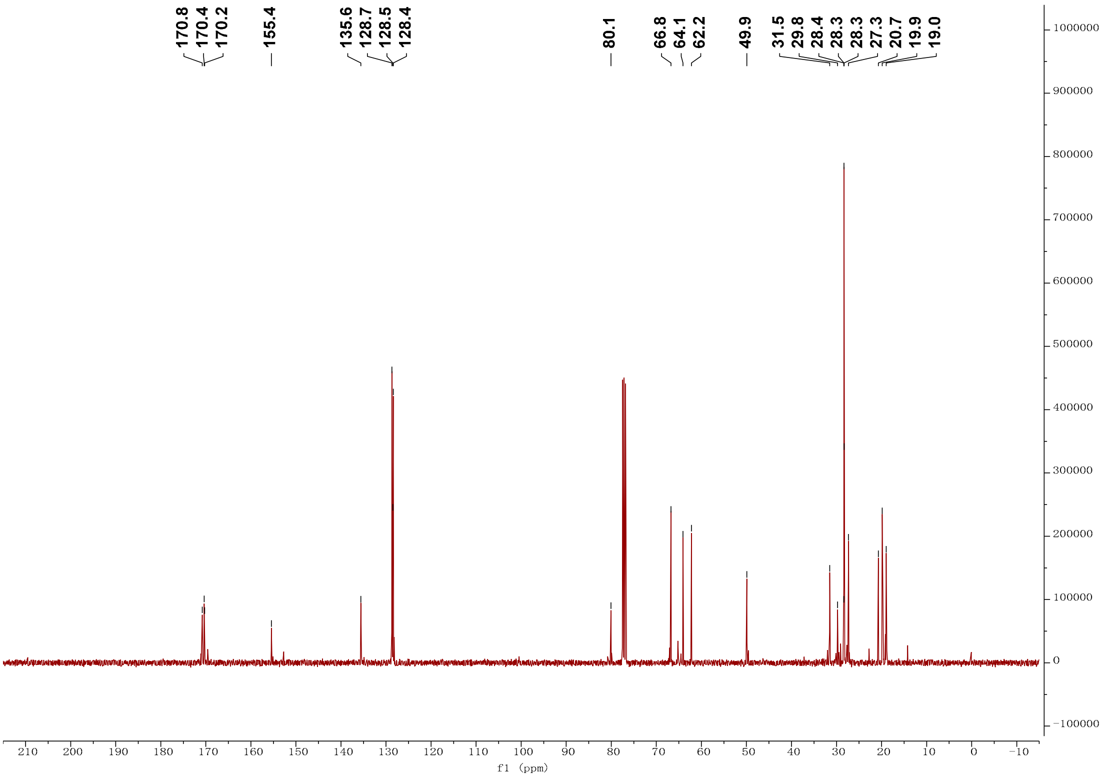

Figure S29. ^{13}C NMR spectrum of compound **6d** in $CDCl_3$ (100 MHz)

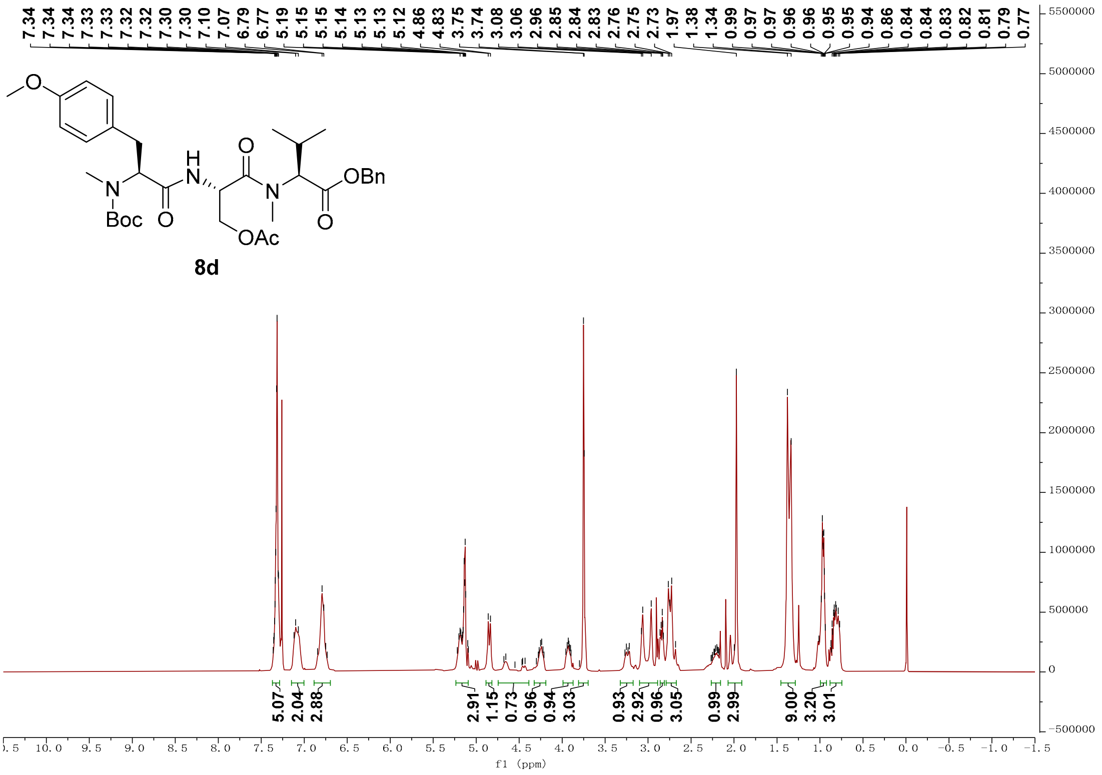

Figure S30. ^{1}H NMR spectrum of compound **8d** in $CDCl_3$ (400 MHz)

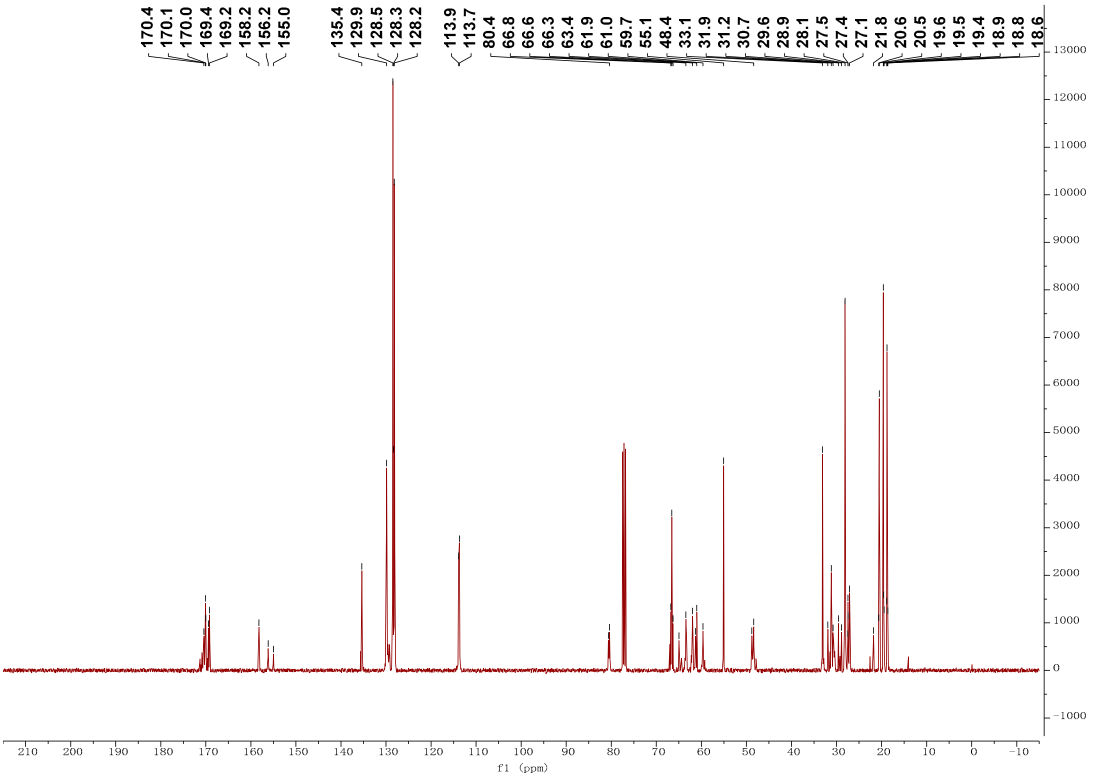

Figure S31. ^{13}C NMR spectrum of compound **8d** in $CDCl_3$ (100 MHz)

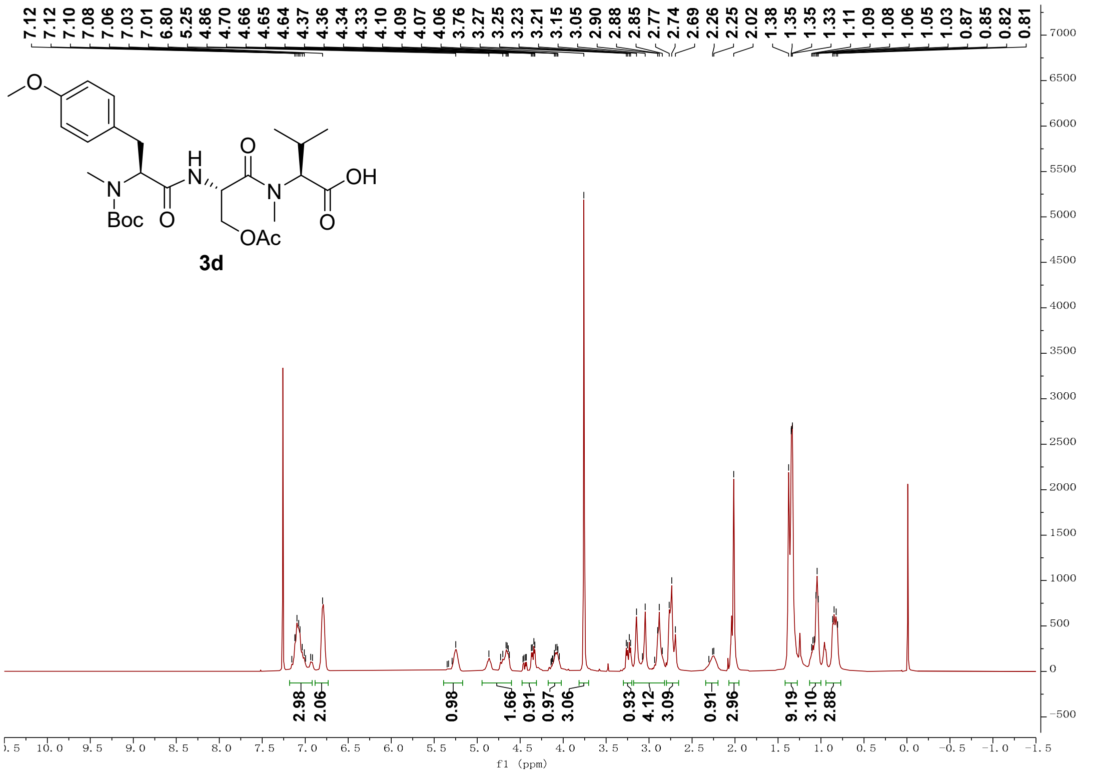

Figure S32. ^{1}H NMR spectrum of compound **3d** in $CDCl_3$ (400 MHz)

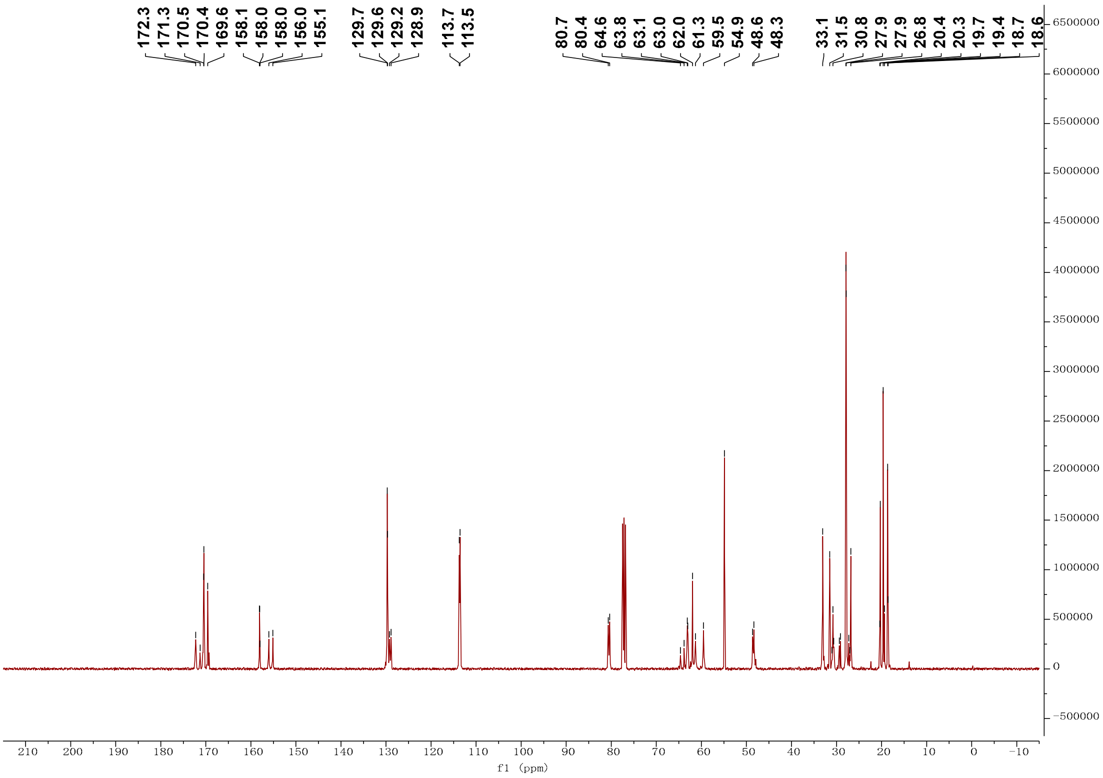

Figure S33. ^{13}C NMR spectrum of compound **3d** in $CDCl_3$ (100 MHz)

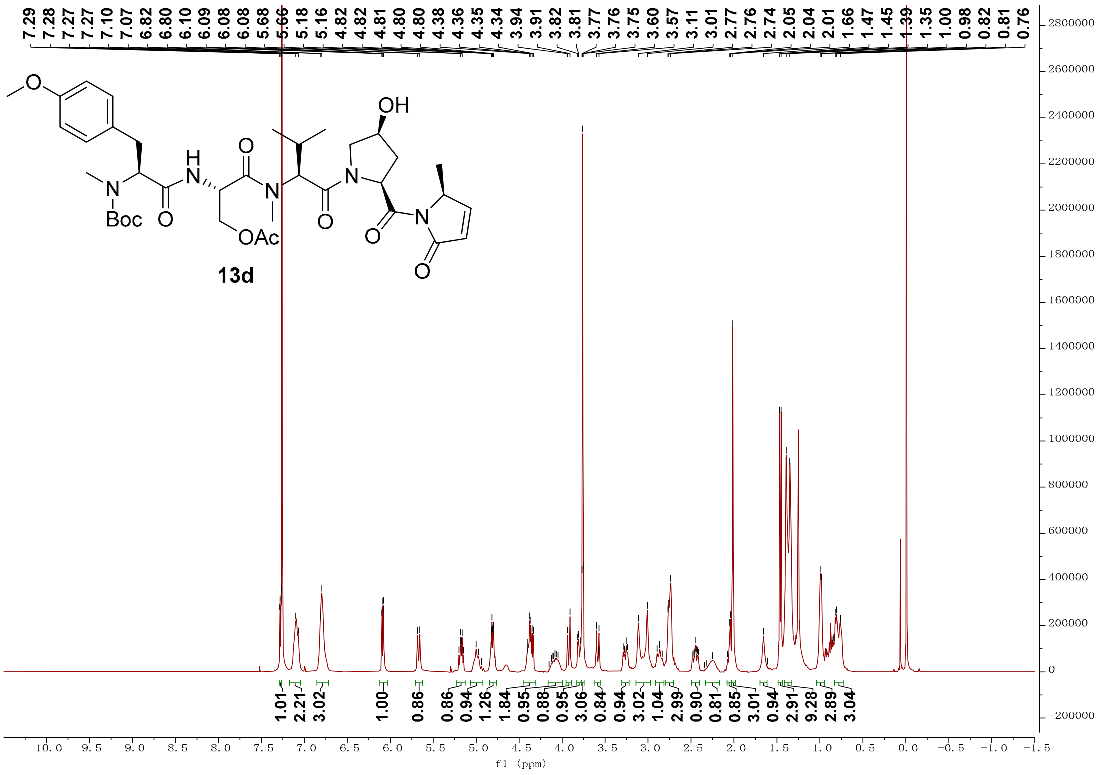

Figure S34. ^{1}H NMR spectrum of compound **13d** in $CDCl_3$ (400 MHz)

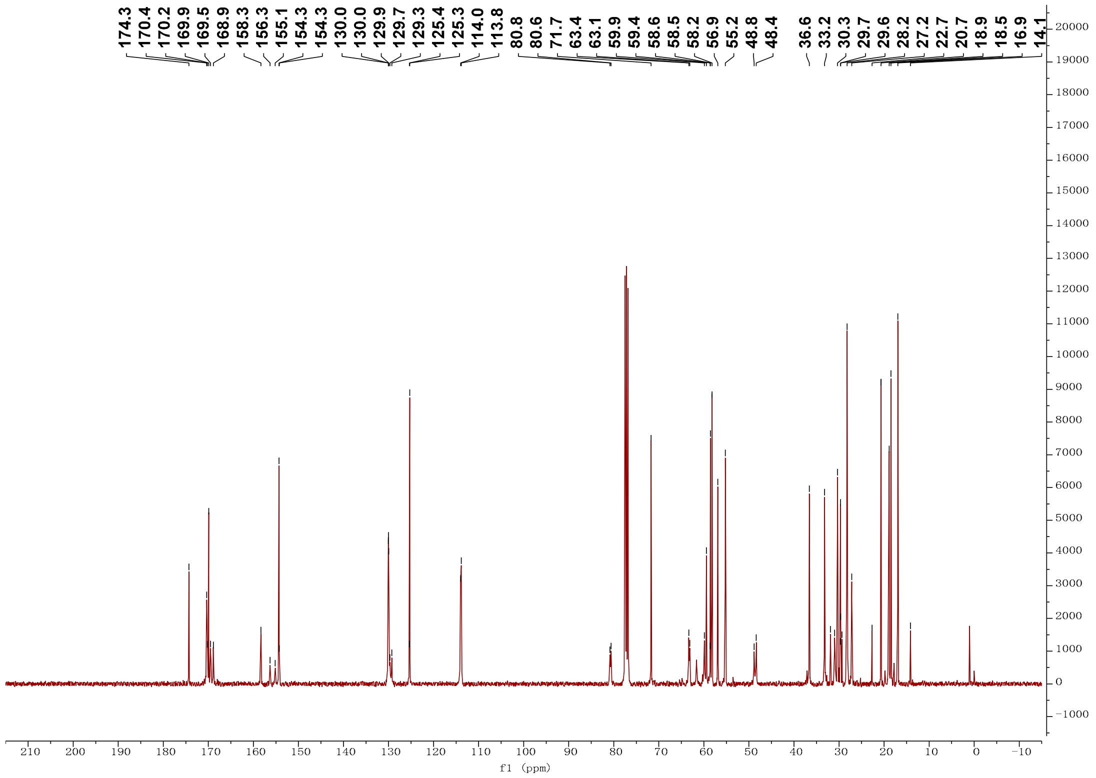

Figure S35. ^{13}C NMR spectrum of compound **13d** in $CDCl_3$ (100 MHz)

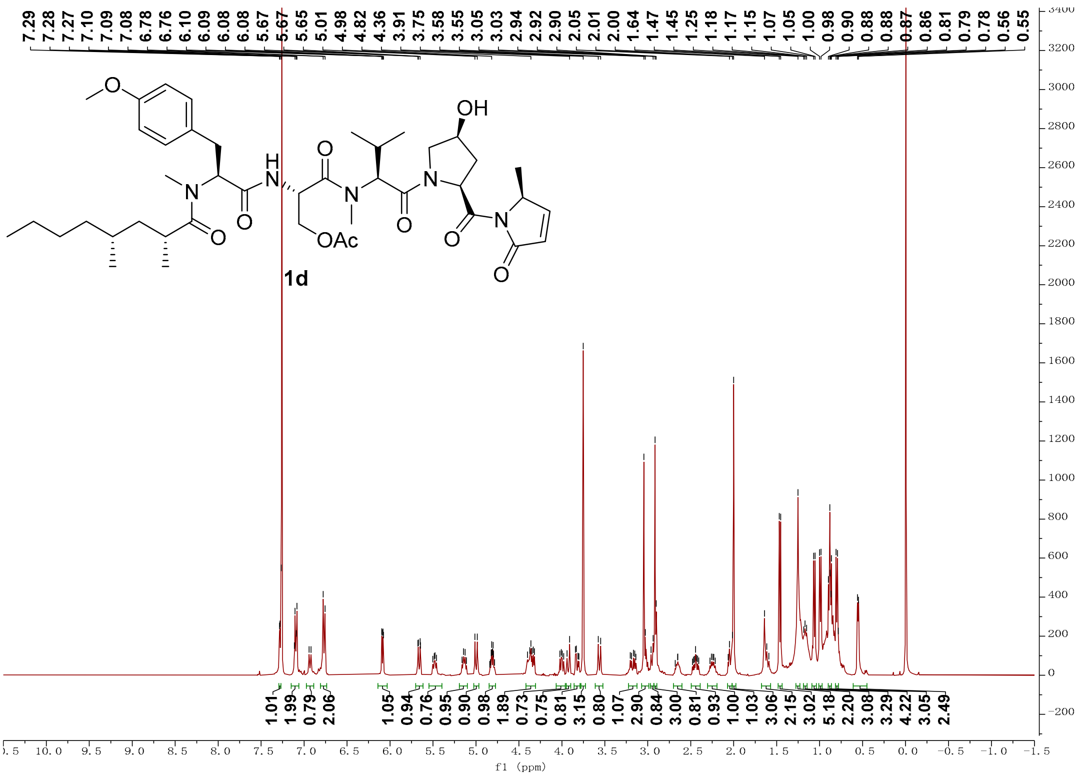

Figure S36. ^1H NMR spectrum of compound **1d** in $CDCl_3$ (400 MHz)

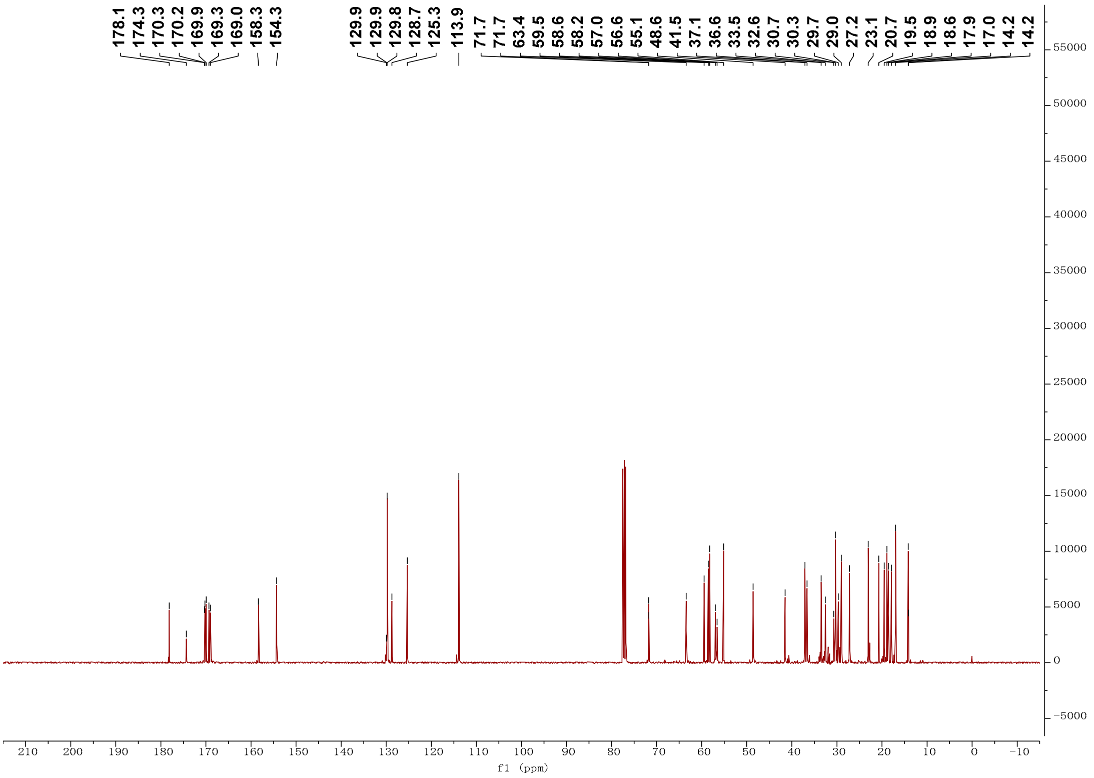

Figure S37. ^{13}C NMR spectrum of compound **1d** in $CDCl_3$ (100 MHz)

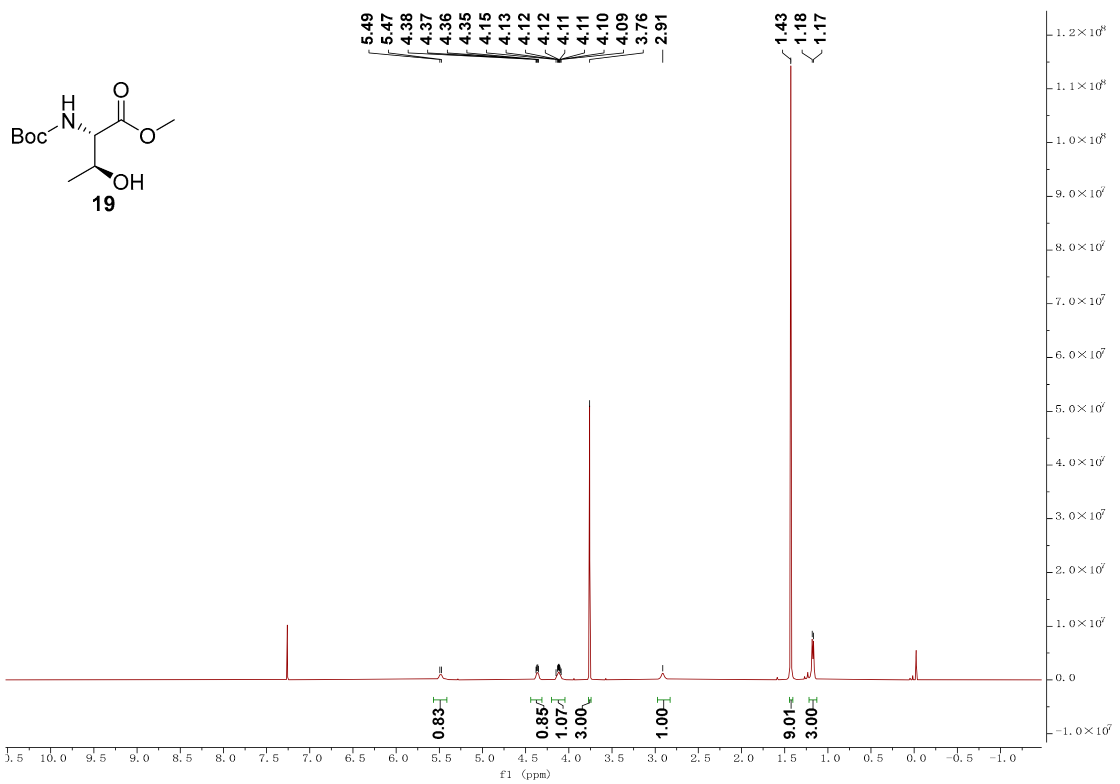

Figure S38. ^{1}H NMR spectrum of compound **19** in $CDCl_3$ (400 MHz)

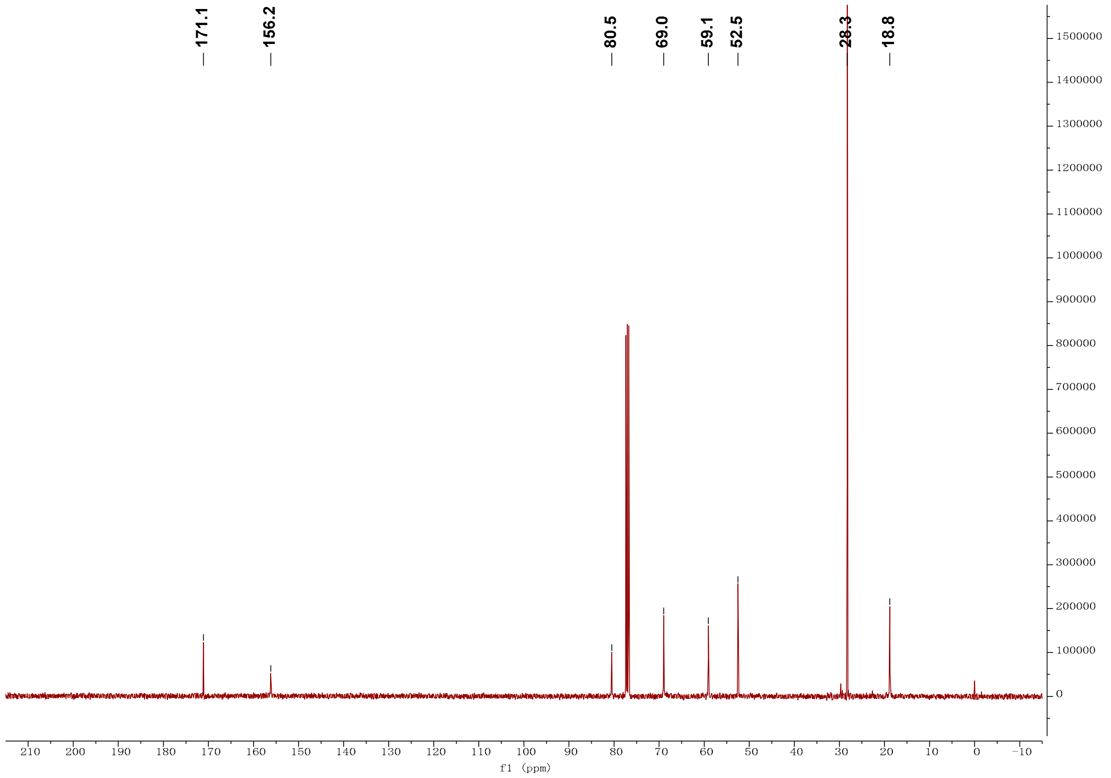

Figure S39. ^{13}C NMR spectrum of compound **19** in $CDCl_3$ (100 MHz)

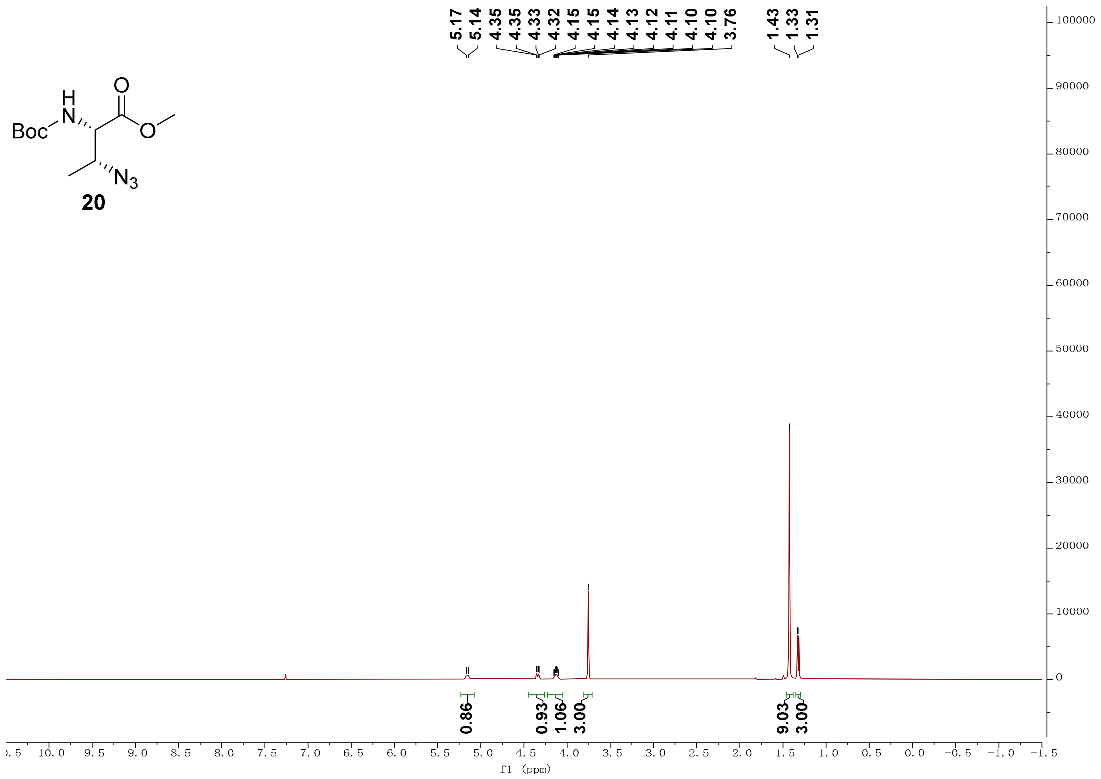

Figure S40. ^{1}H NMR spectrum of compound **20** in $CDCl_3$ (400 MHz)

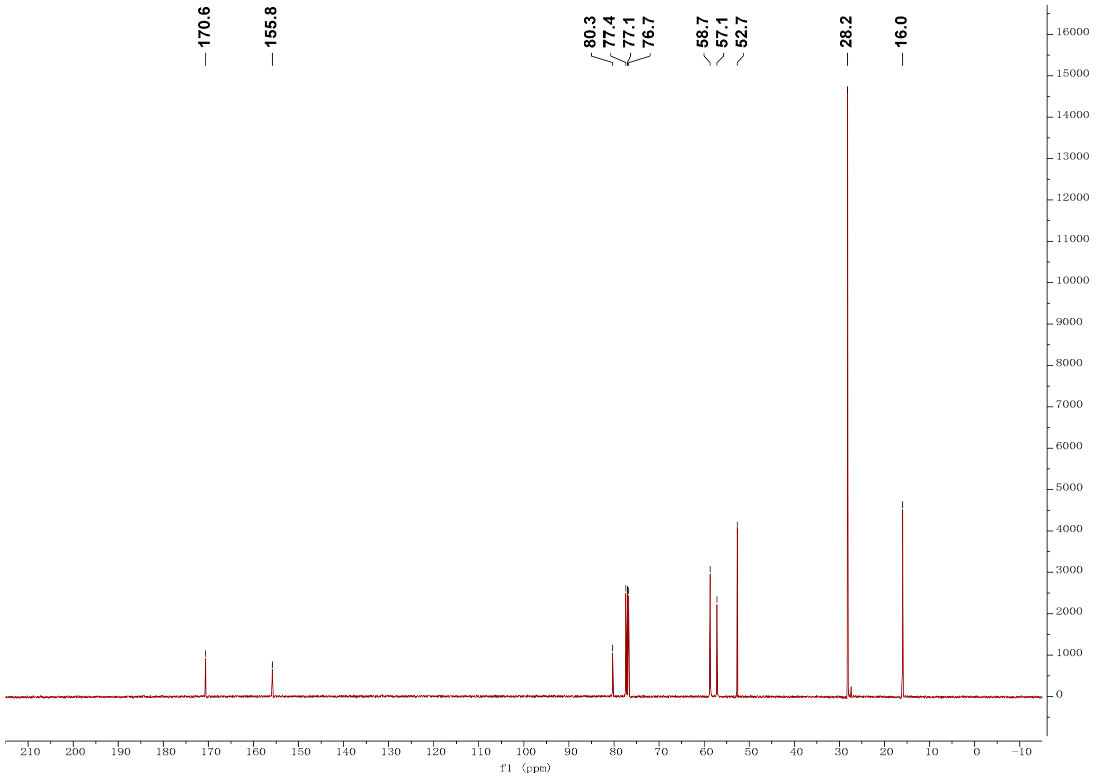

Figure S41. ^{13}C NMR spectrum of compound **20** in $CDCl_3$ (100 MHz)

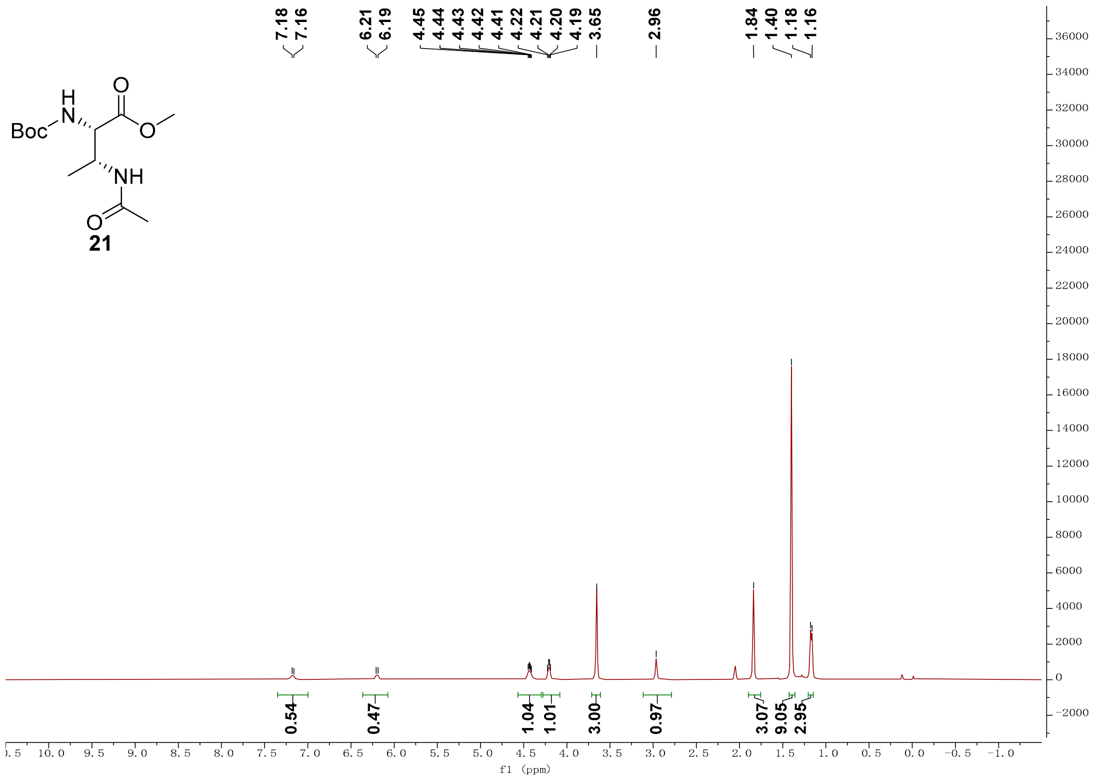

Figure S42. ^{1}H NMR spectrum of compound **21** in Acetone-*d*6 (400 MHz)

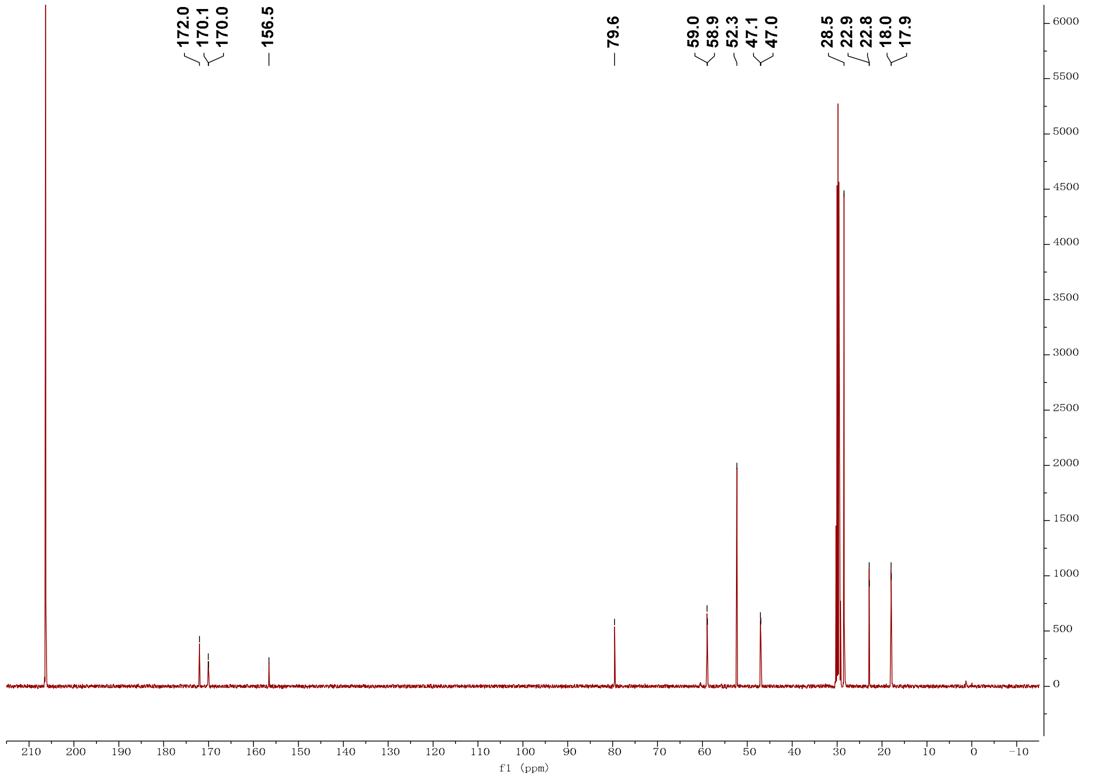

Figure S43. ^{13}C NMR spectrum of compound **21** in Acetone-*d*6 (100 MHz)

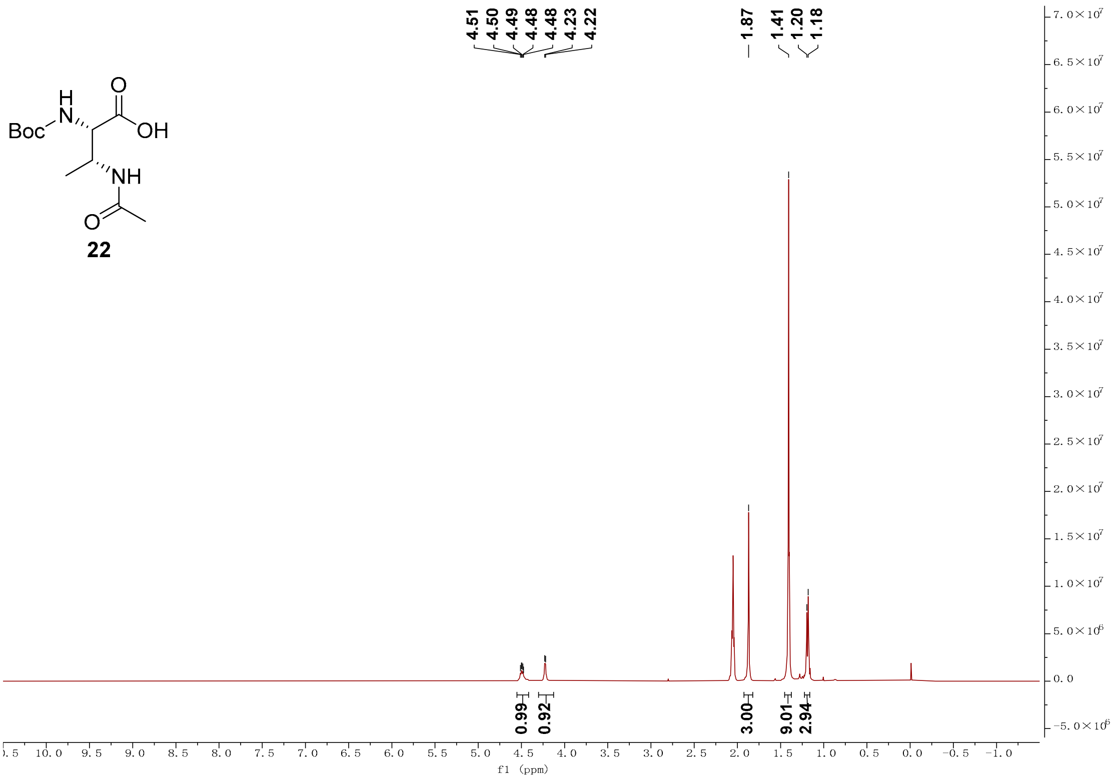

Figure S44. ^{1}H NMR spectrum of compound **22** in Acetone-*d*6 (400 MHz)

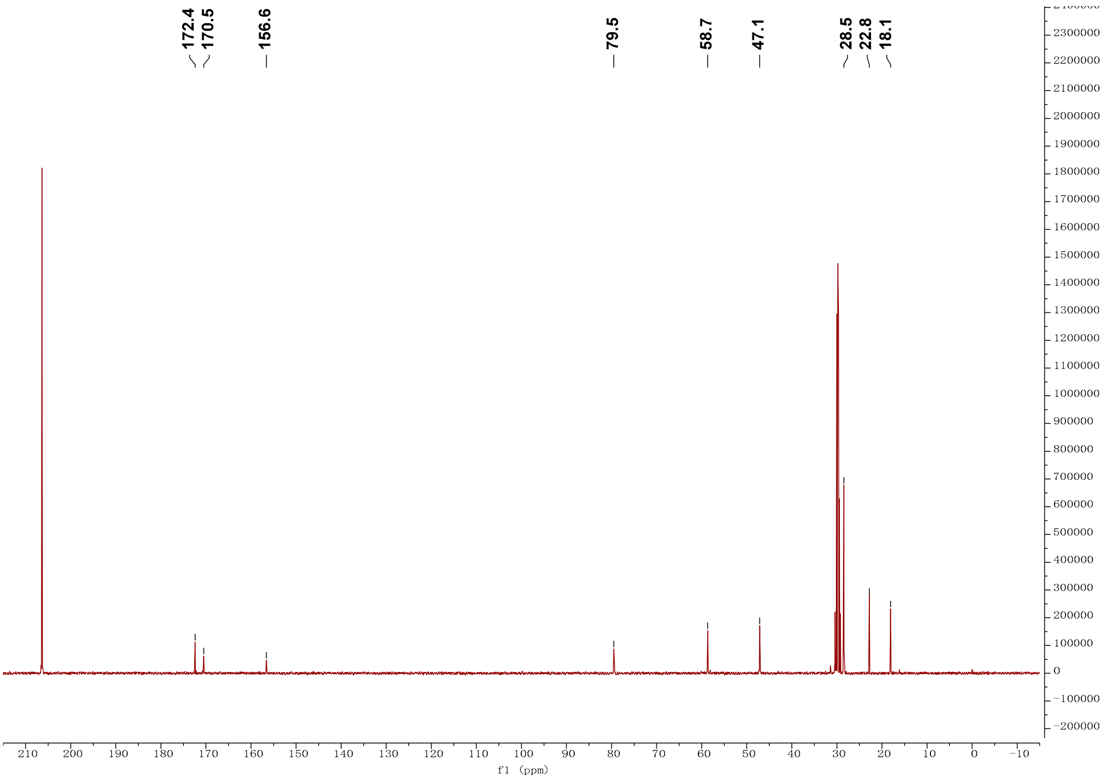

Figure S45. ^{13}C NMR spectrum of compound **22** in Acetone-*d*6 (100 MHz)

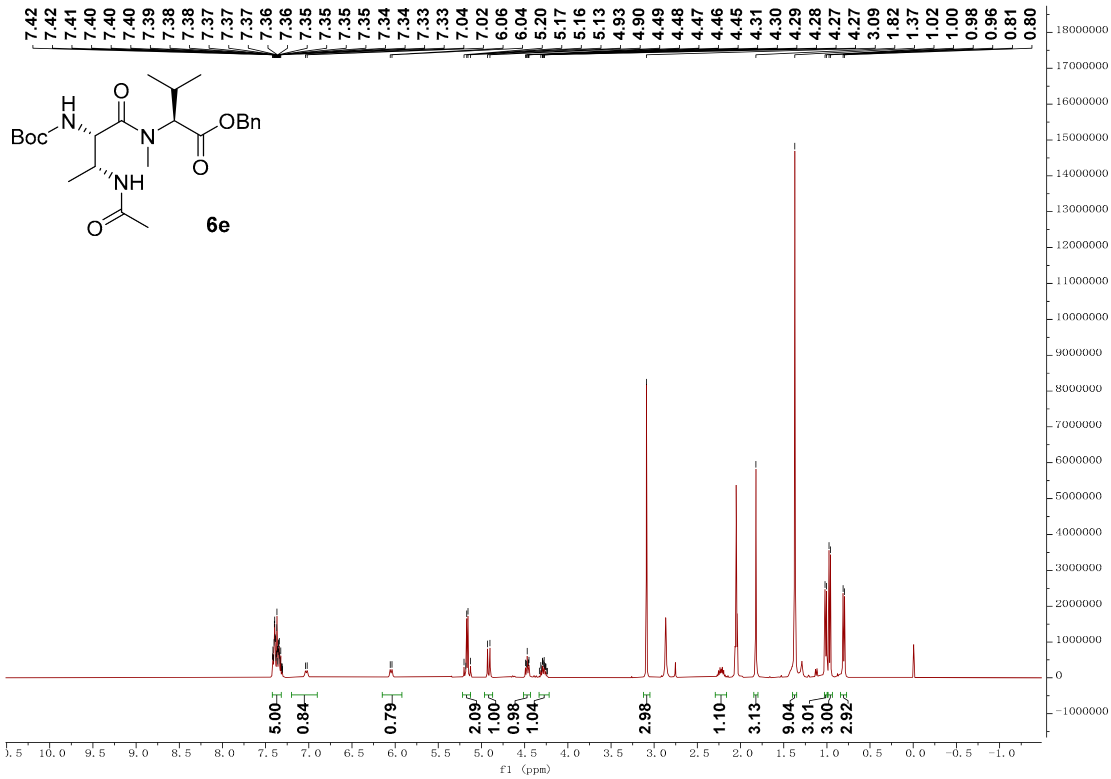

Figure S46. ^{1}H NMR spectrum of compound **6e** in Acetone-*d*6 (400 MHz)

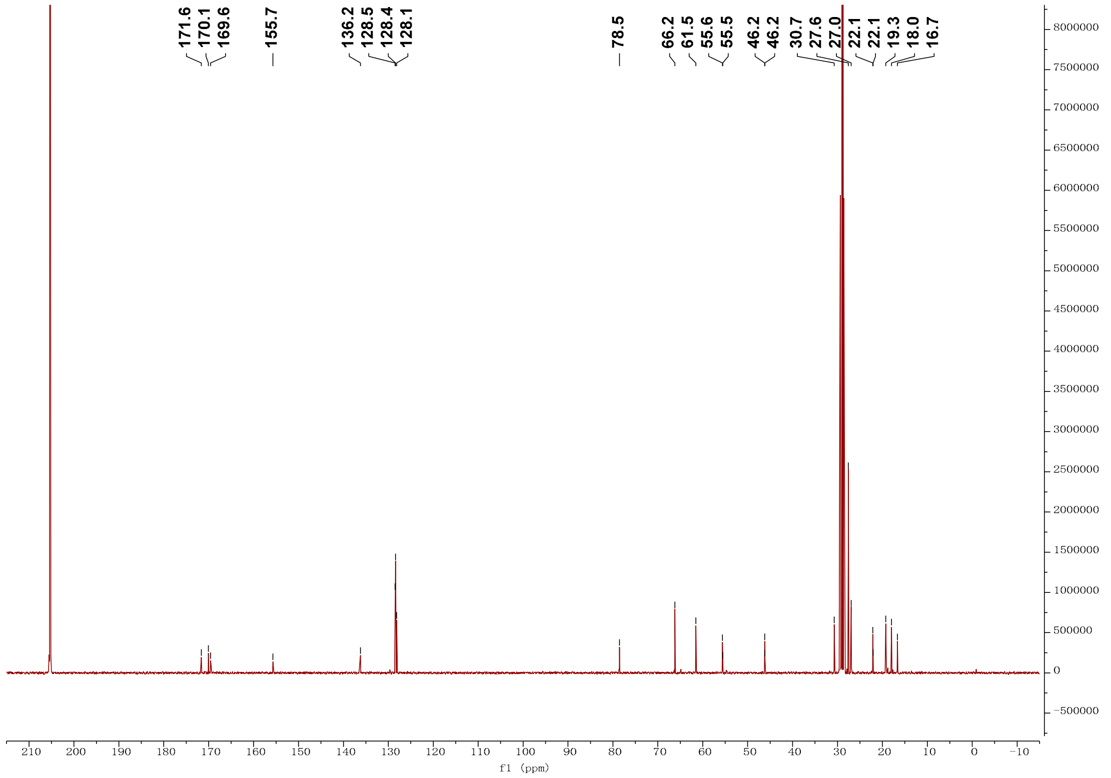

Figure S47. ^{13}C NMR spectrum of compound **6e** in Acetone-*d*6 (100 MHz)

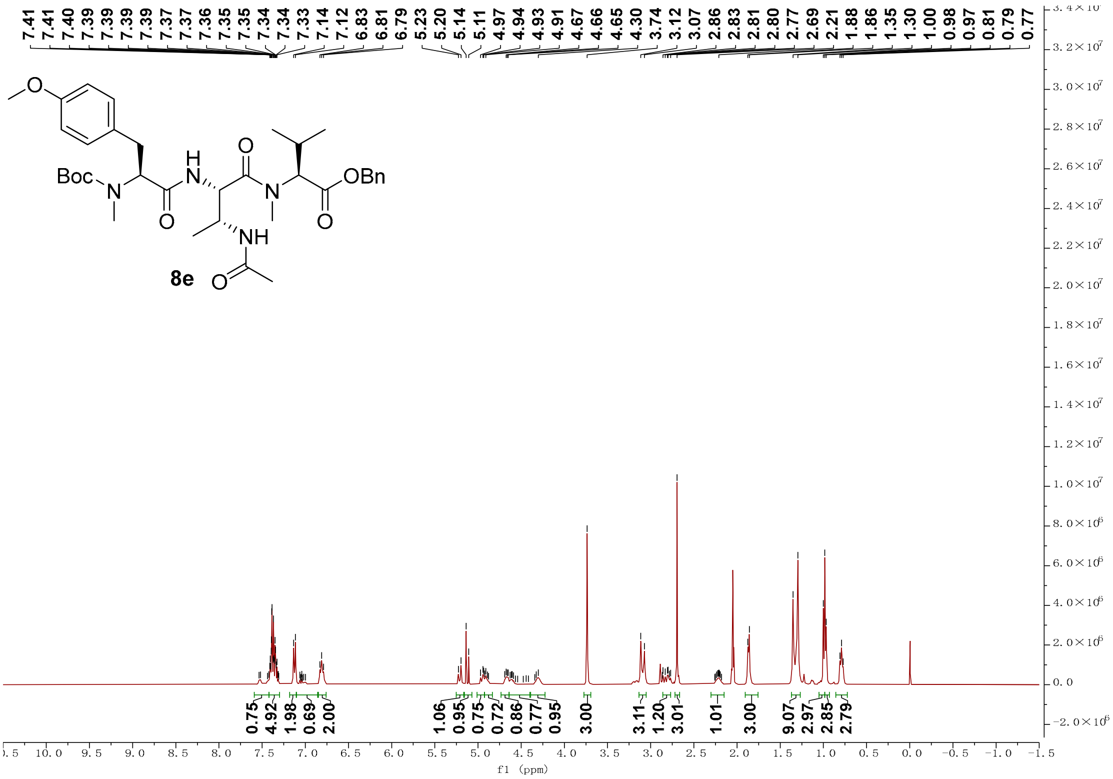

Figure S48. ^{1}H NMR spectrum of compound **8e** in Acetone-*d*6 (400 MHz)

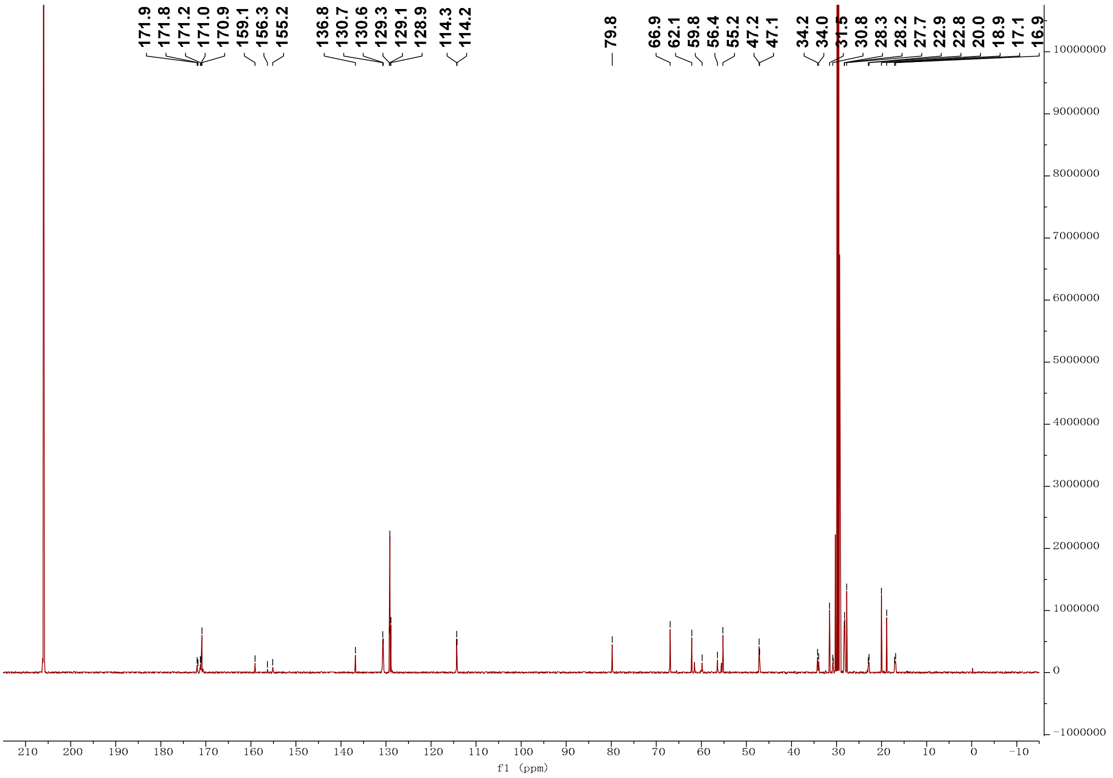

Figure S49. ^{13}C NMR spectrum of compound **8e** in Acetone-*d*6 (100 MHz)

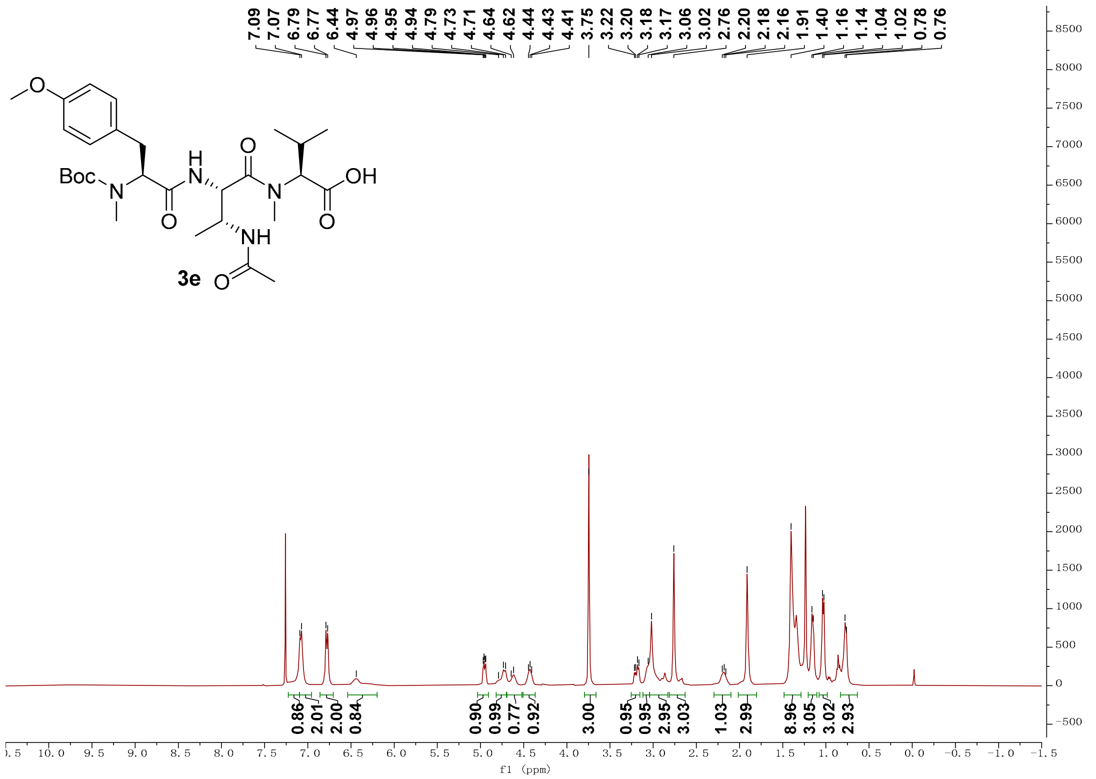

Figure S50. ^{1}H NMR spectrum of compound **3e** in $CDCl_3$ (400 MHz)

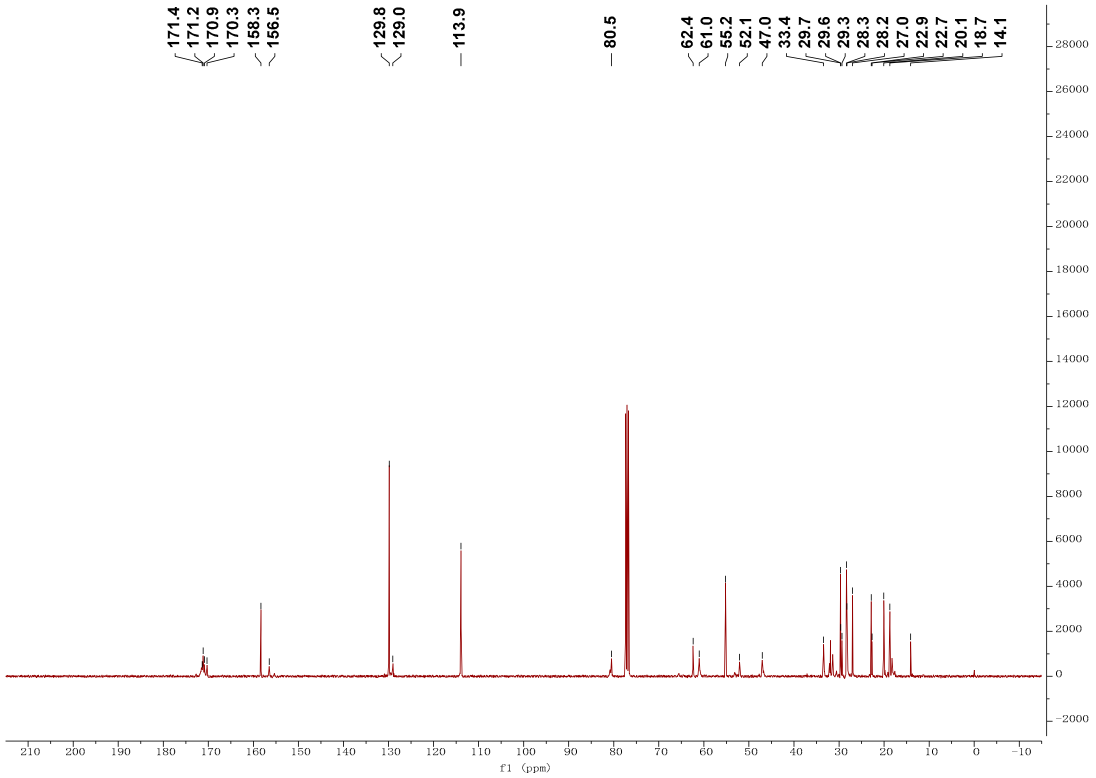

Figure S51. ^{13}C NMR spectrum of compound **3e** in $CDCl_3$ (100 MHz)

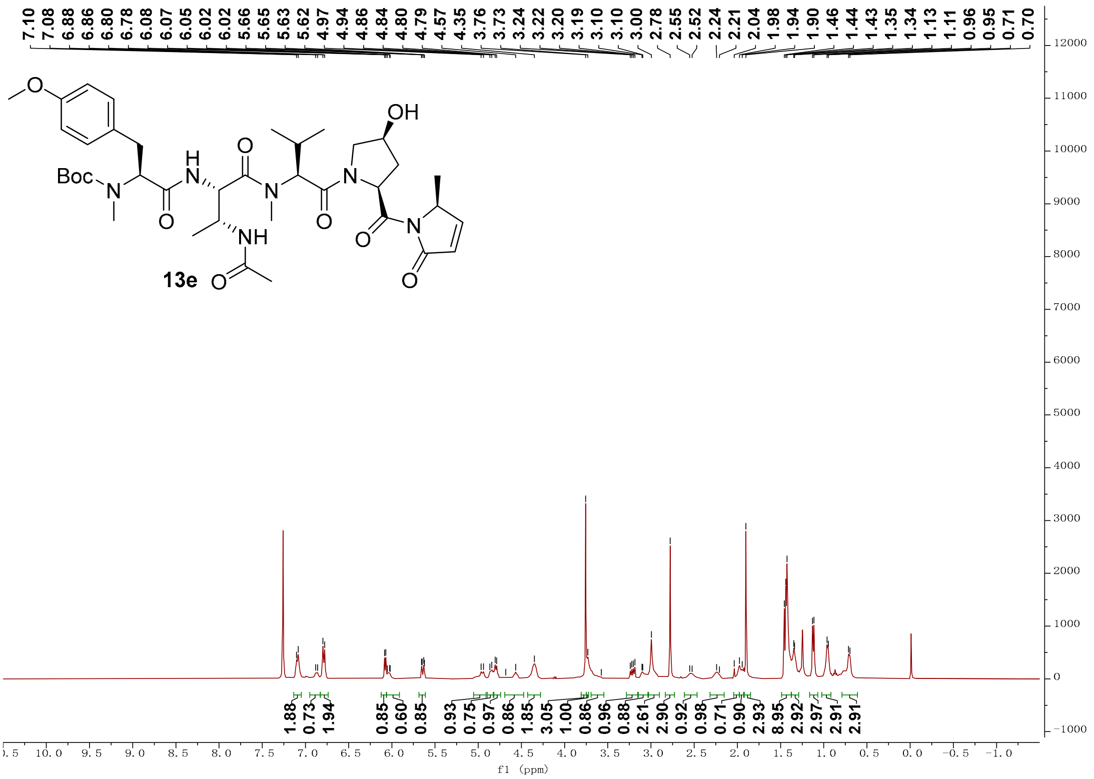

Figure S52. ^{1}H NMR spectrum of compound **13e** in $CDCl_3$ (400 MHz)

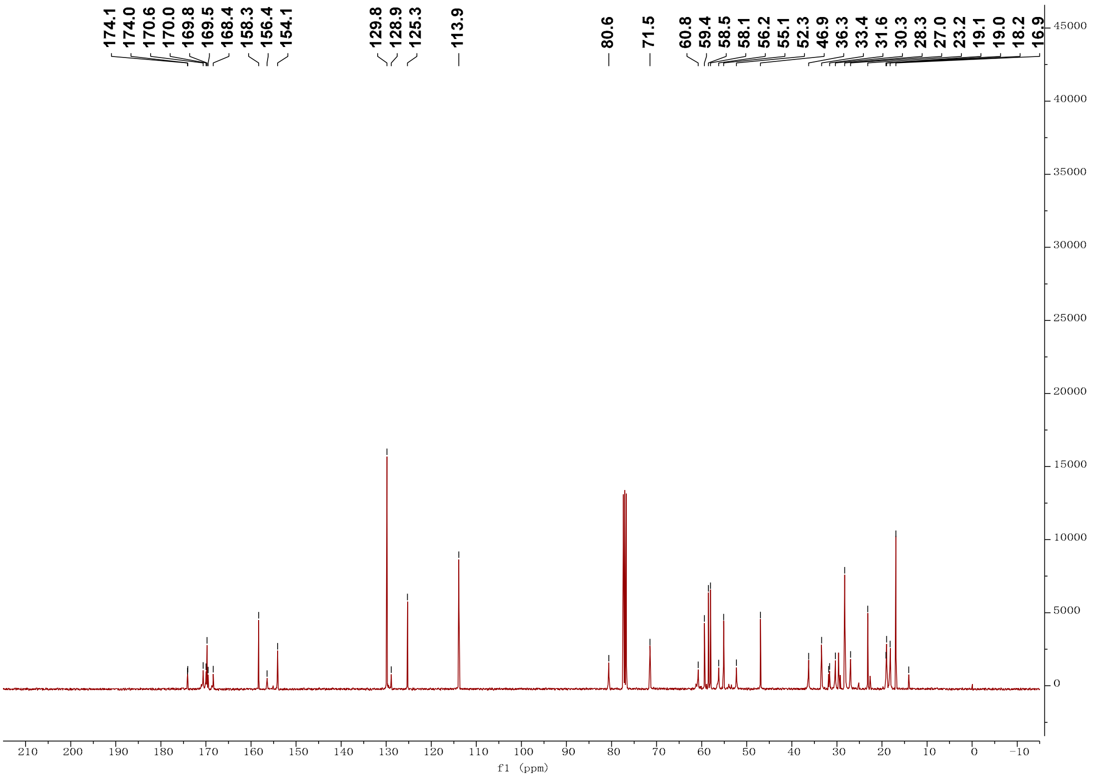

Figure S53. ^{13}C NMR spectrum of compound **13e** in $CDCl_3$ (100 MHz)

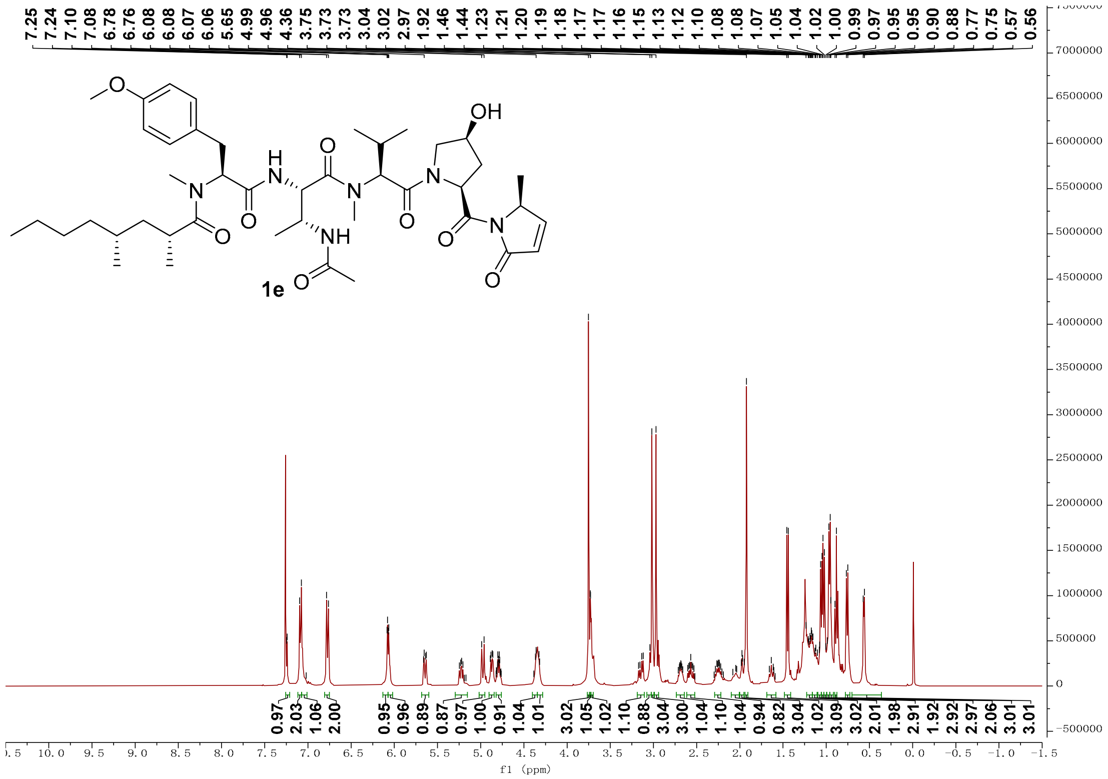

Figure S54. ^{1}H NMR spectrum of compound **1e** in $CDCl_3$ (400 MHz)

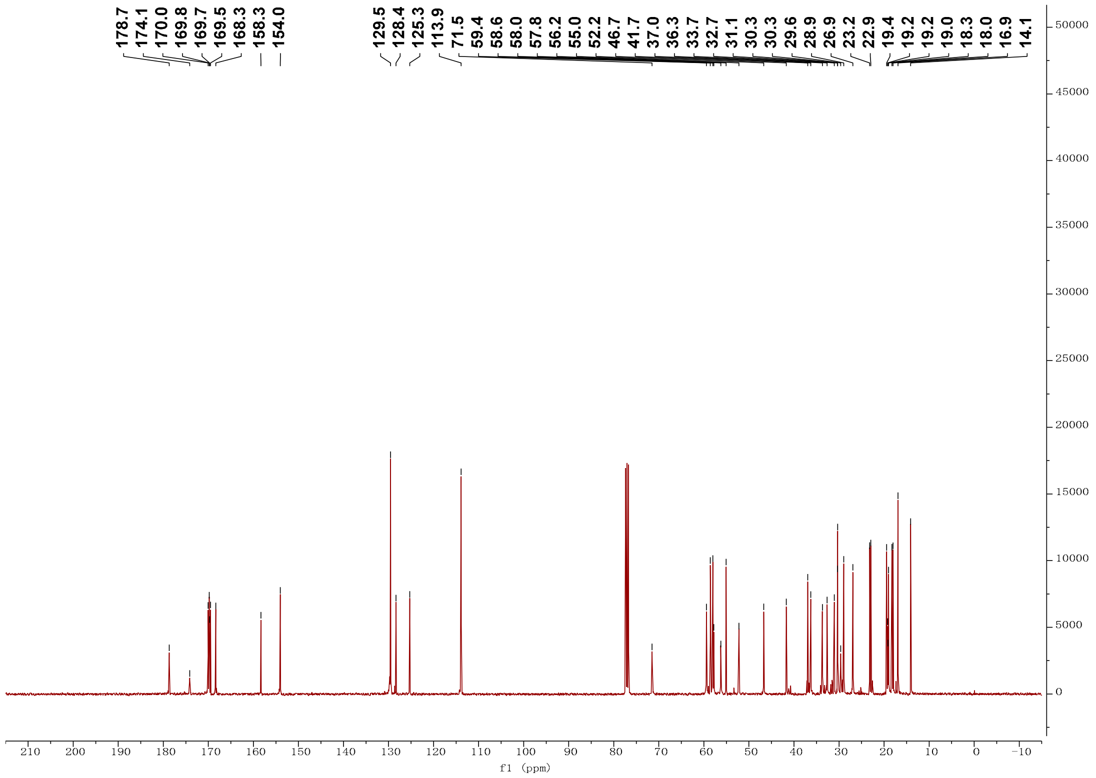

Figure S55. ^{13}C NMR spectrum of compound **1e** in $CDCl_3$ (100 MHz)

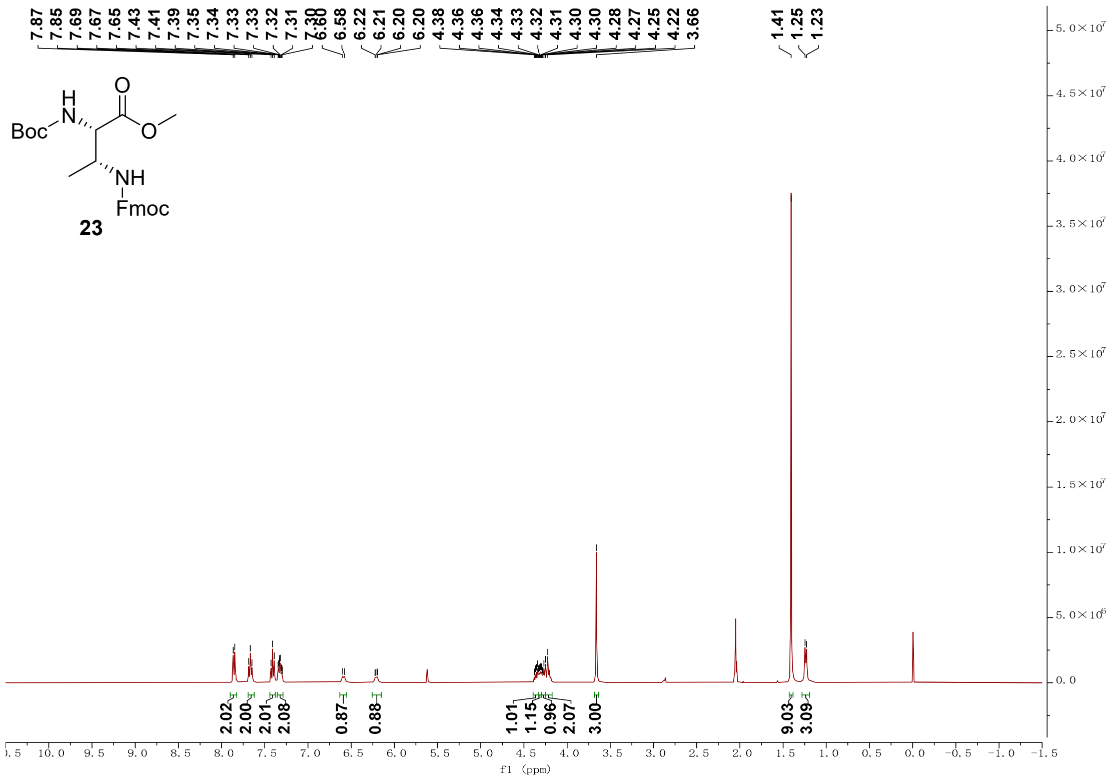

Figure S56. ^{1}H NMR spectrum of compound **23** in Acetone-*d*6 (400 MHz)

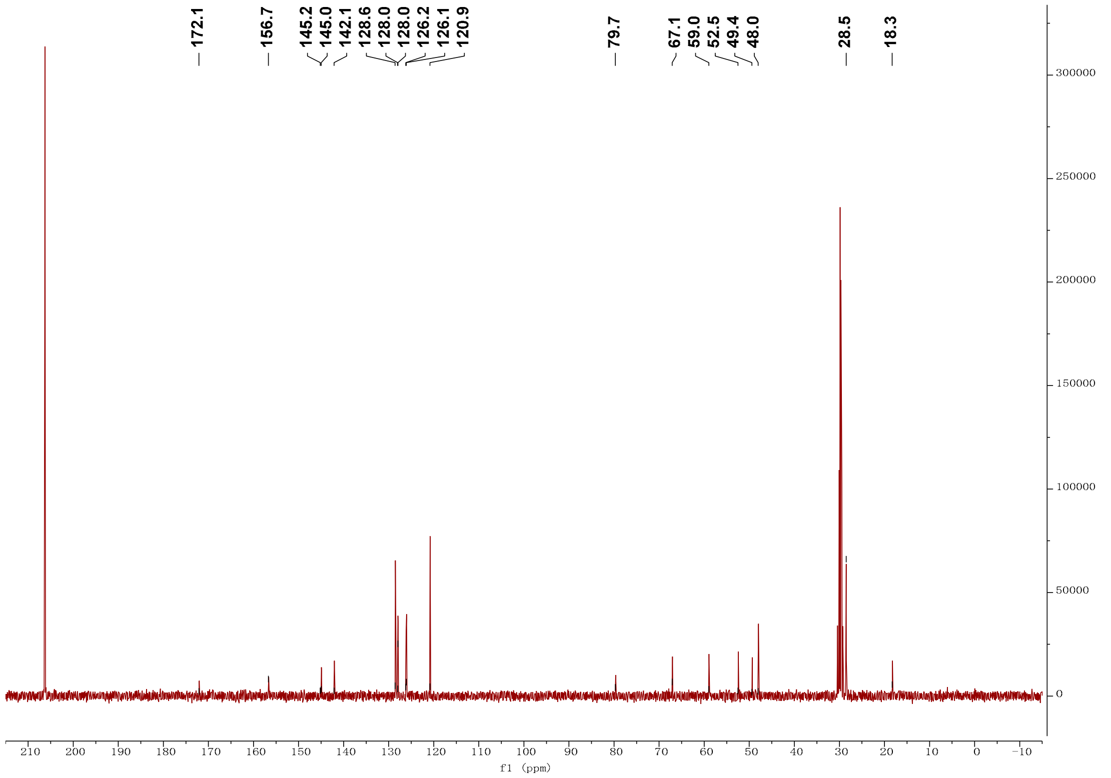

Figure S57. ^{13}C NMR spectrum of compound **23** in Acetone-*d*6 (100 MHz)

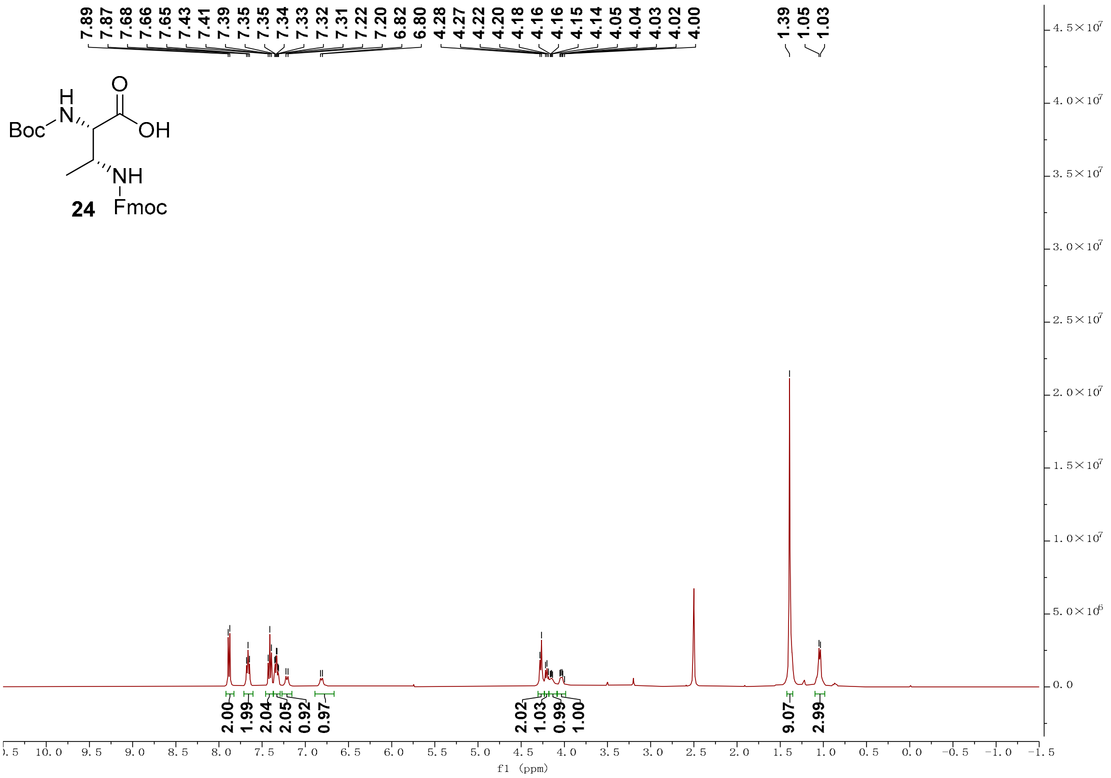

Figure S58. ^{1}H NMR spectrum of compound **24** in DMSO-*d*6 (400 MHz)

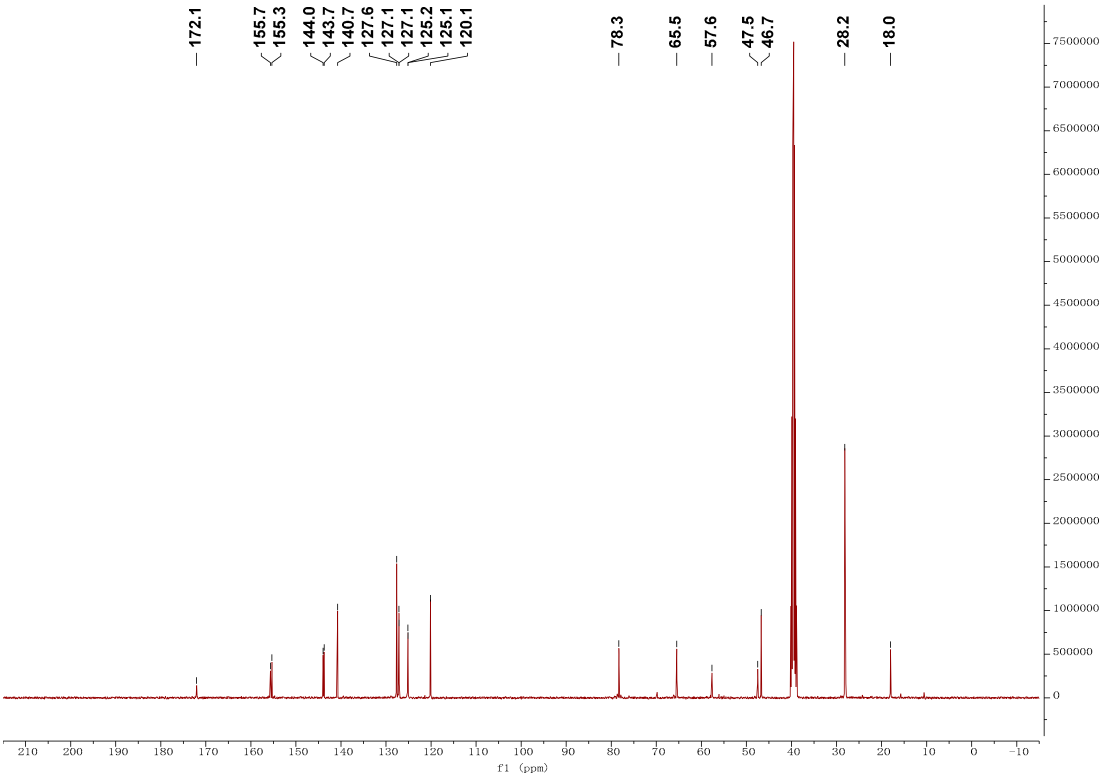

Figure S59. ^{13}C NMR spectrum of compound **24** in DMSO-*d*6 (100 MHz)

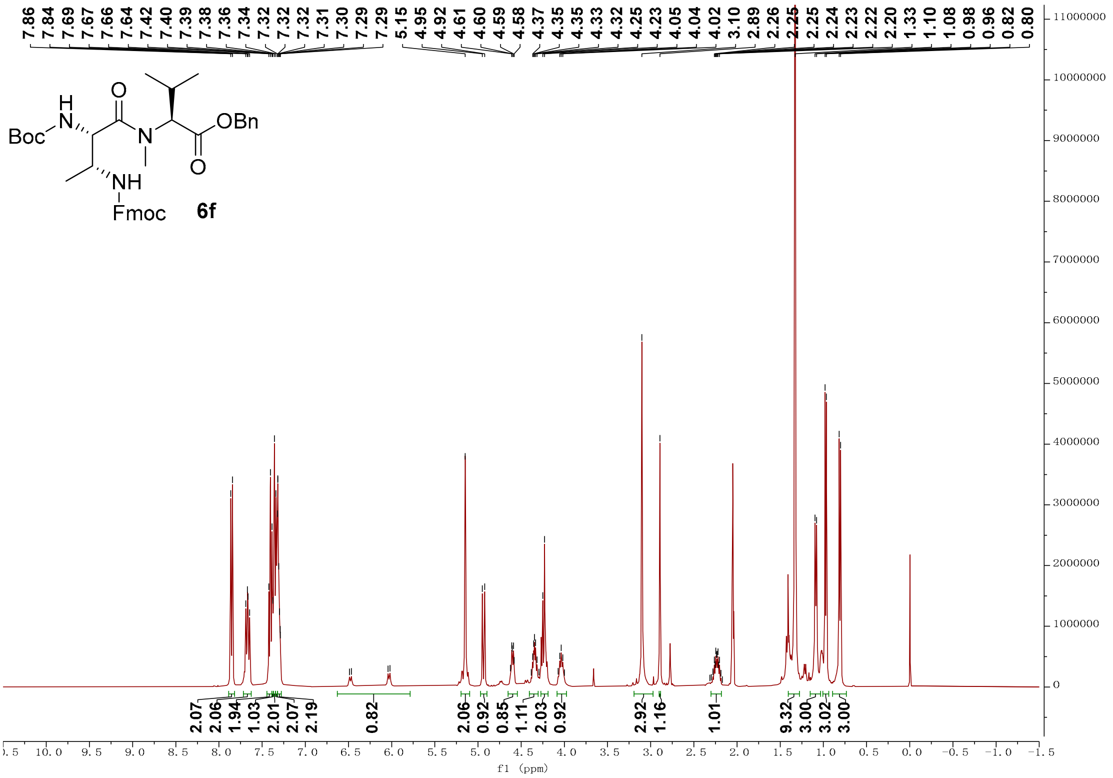

Figure S60. ^{1}H NMR spectrum of compound **6f** in Acetone-*d*6 (400 MHz)

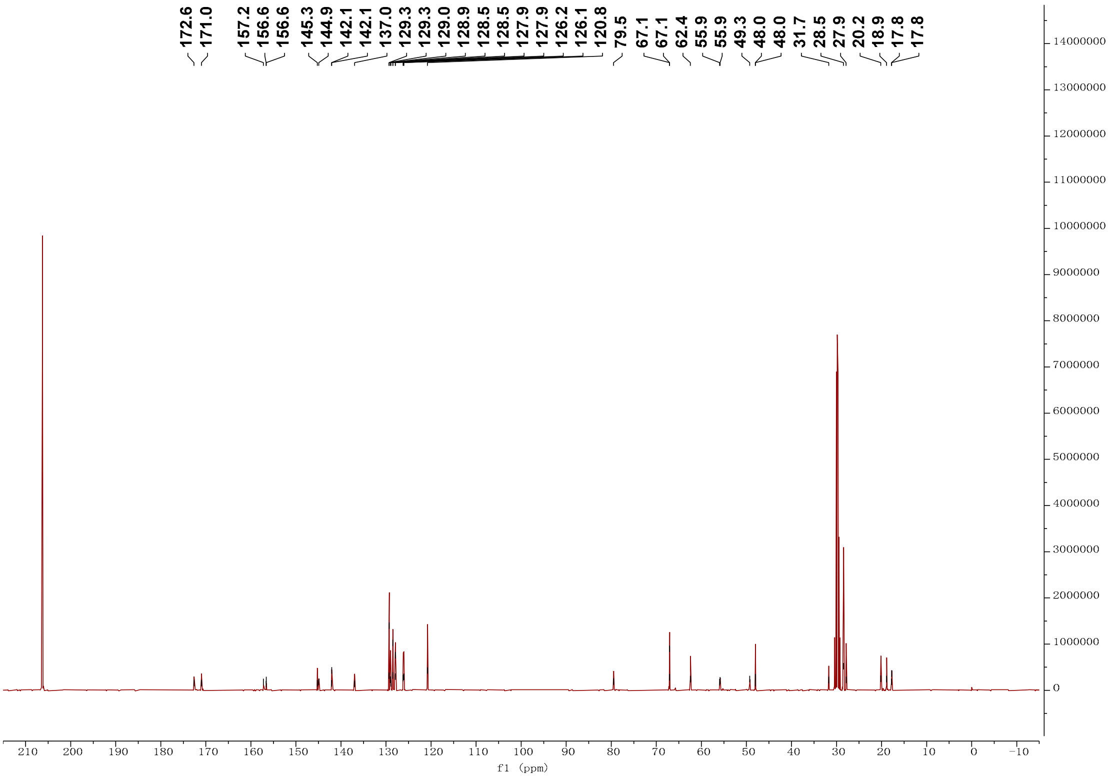

Figure S61. ^{13}C NMR spectrum of compound **6f** in Acetone-*d*6 (100 MHz)

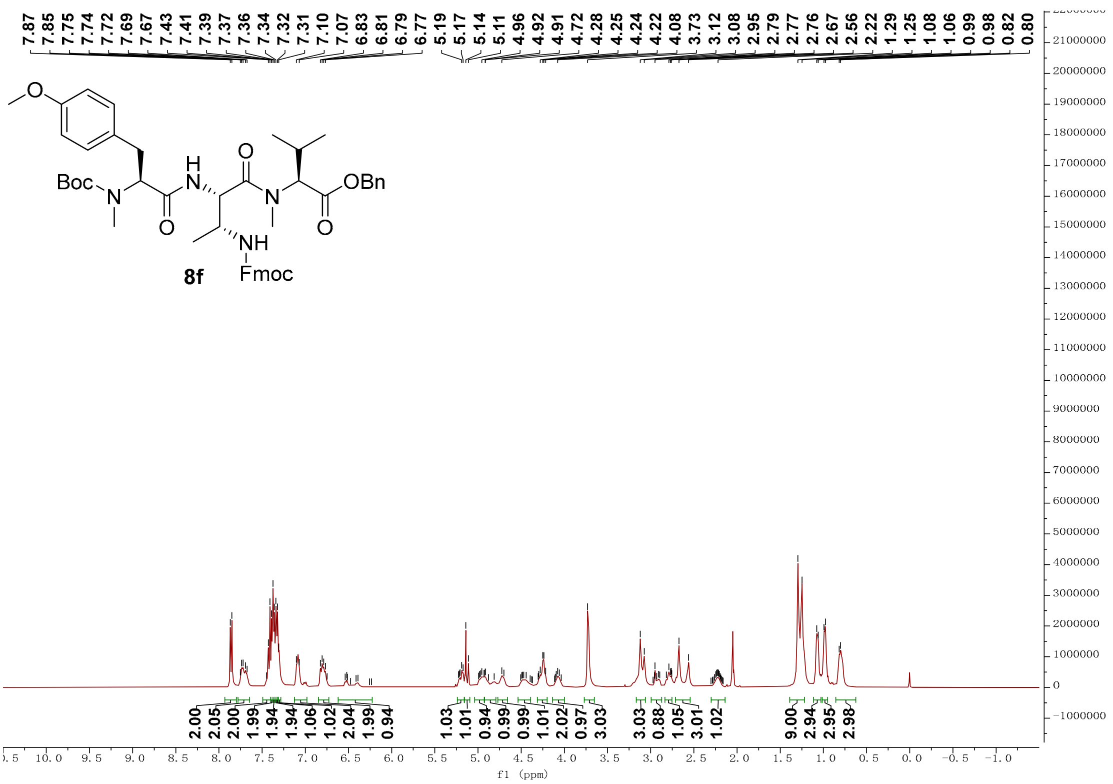

Figure S62. ^{1}H NMR spectrum of compound **8f** in Acetone-*d*6 (400 MHz)

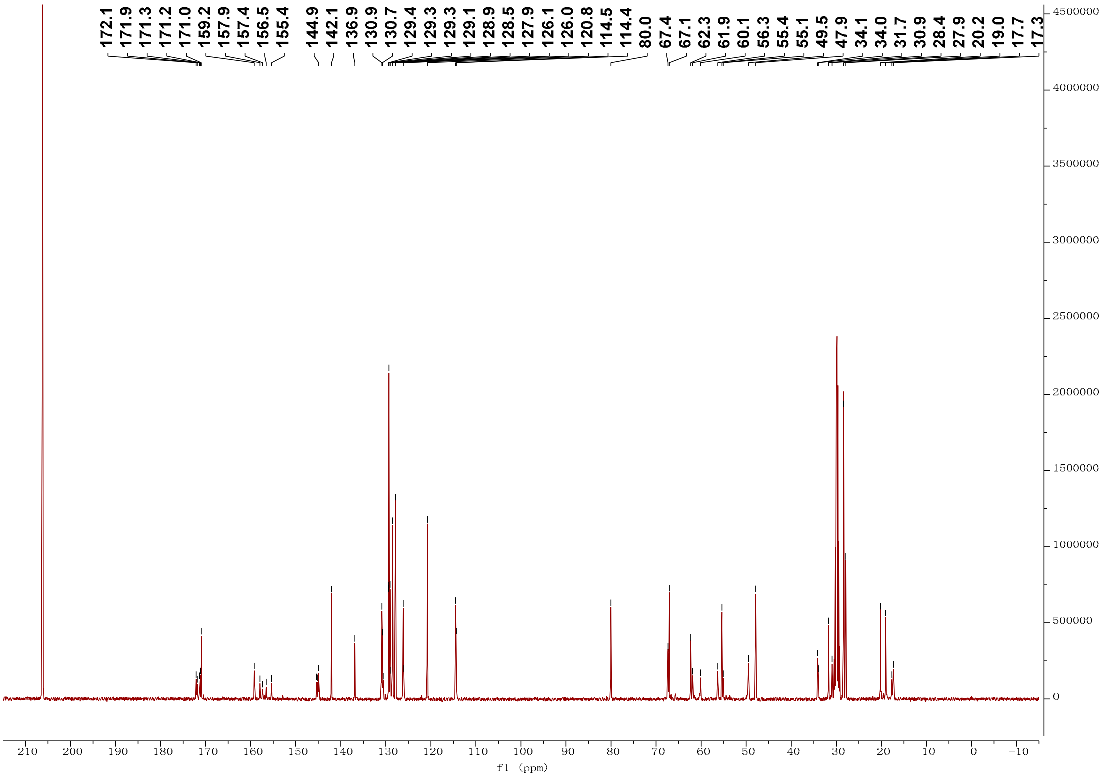

Figure S63. ^{13}C NMR spectrum of compound **8f** in Acetone-*d*6 (100 MHz)

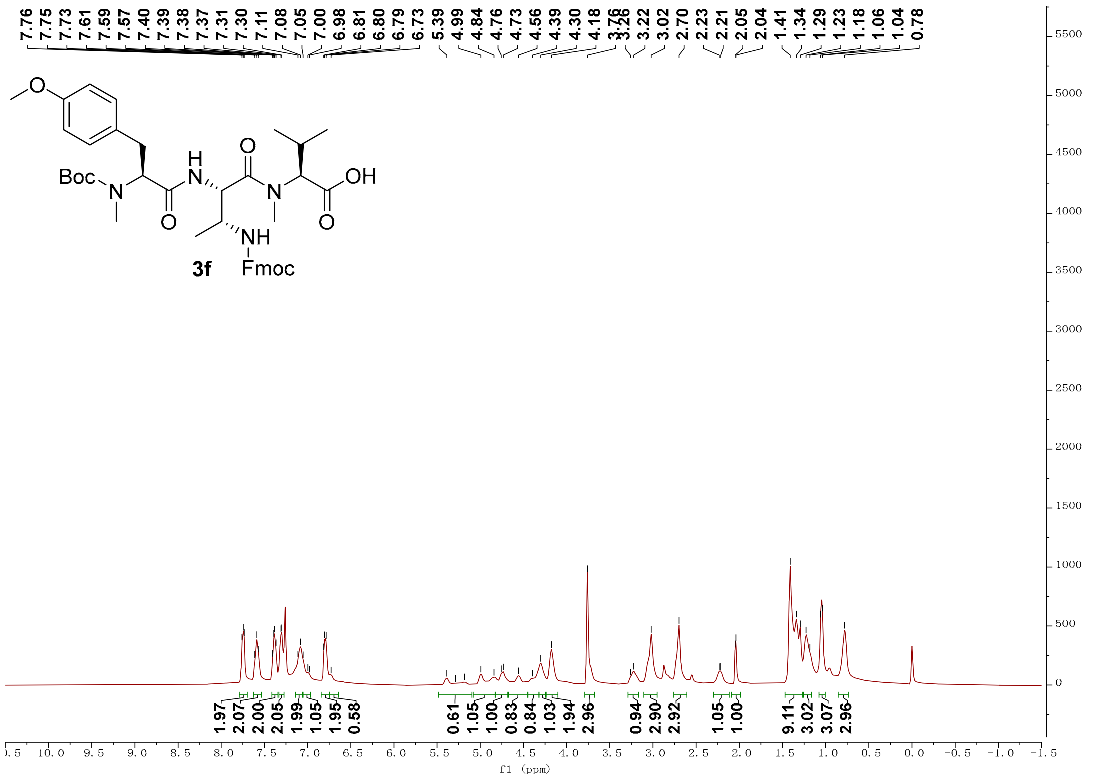

Figure S64. ^{1}H NMR spectrum of compound **3f** in $CDCl_3$ (400 MHz)

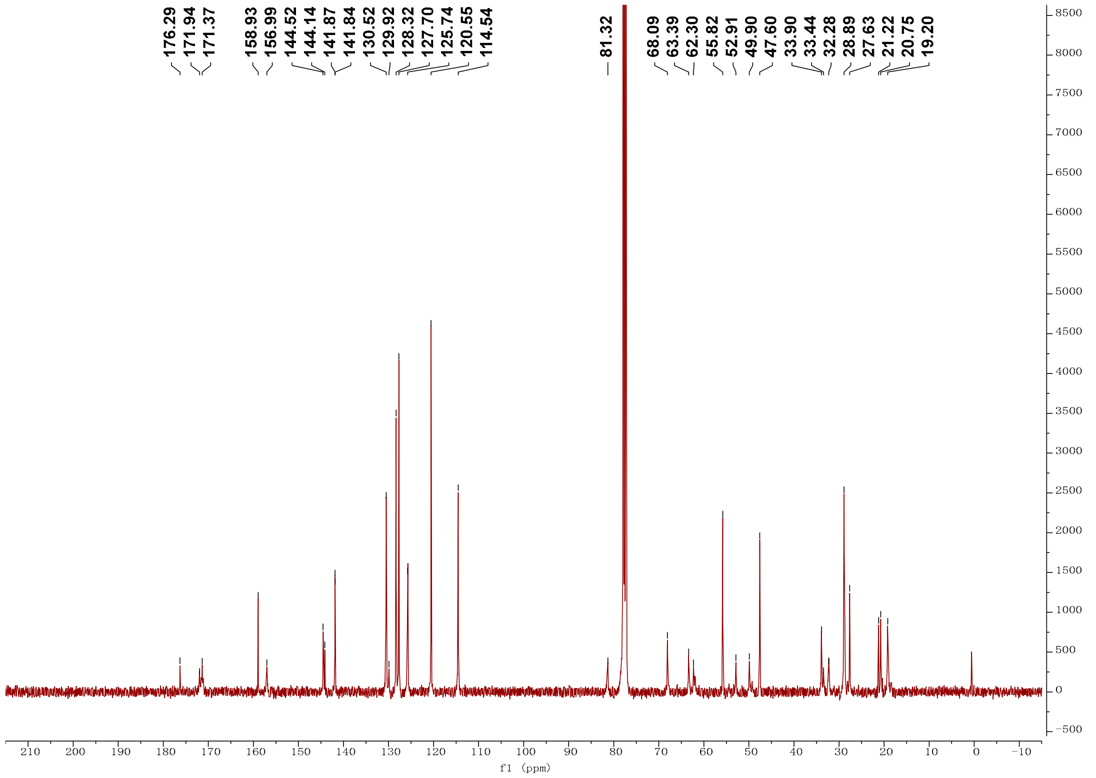

Figure S65. ^{13}C NMR spectrum of compound **3f** in $CDCl_3$ (100 MHz)

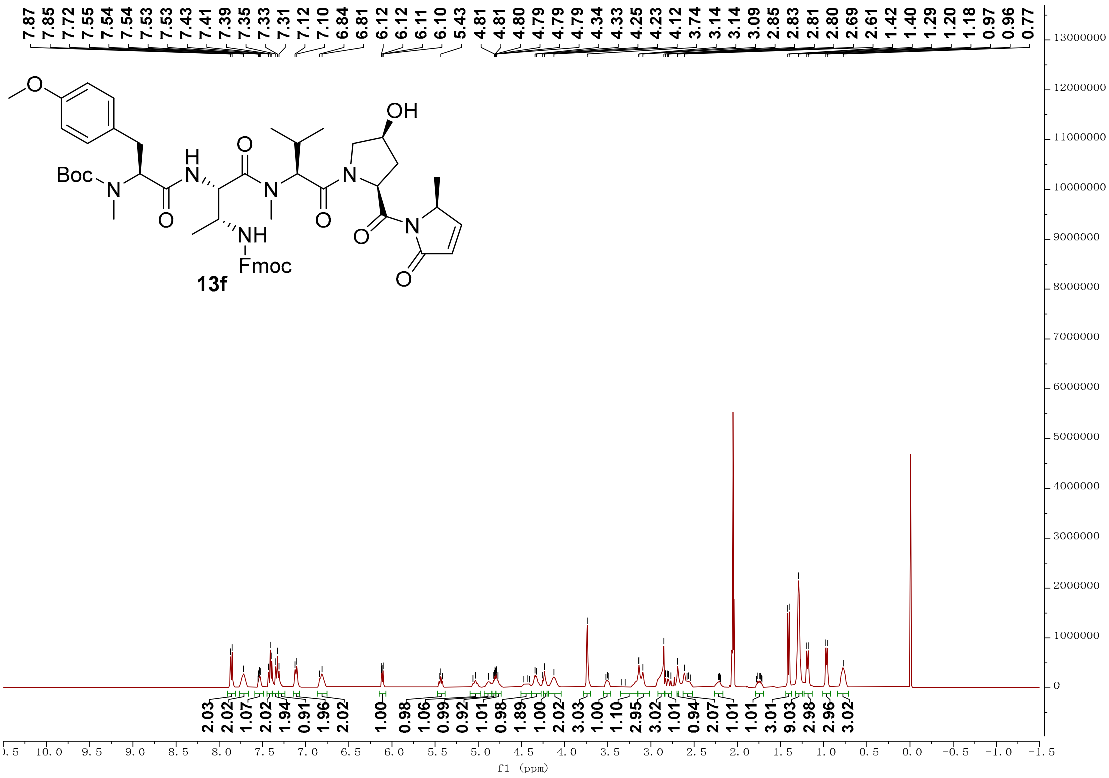

Figure S66. ^{1}H NMR spectrum of compound **13f** in Acetone-*d*6 (400 MHz)

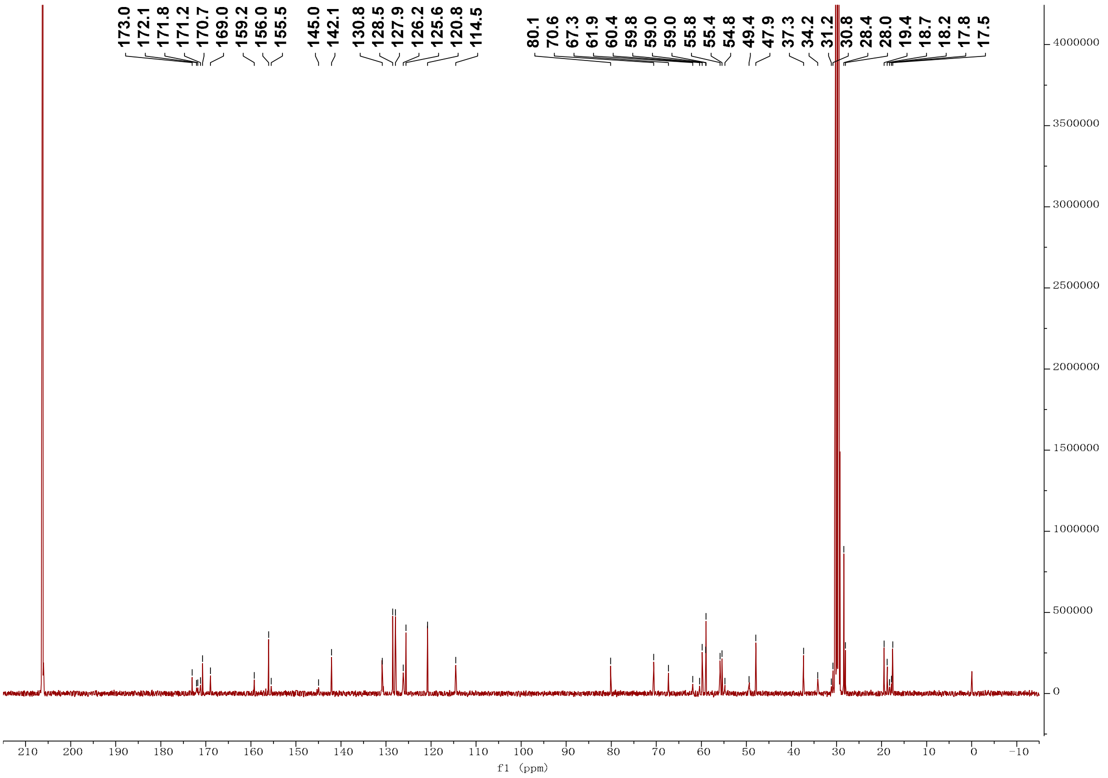

Figure S67. ^{13}C NMR spectrum of compound **13f** in Acetone-*d*6 (100 MHz)

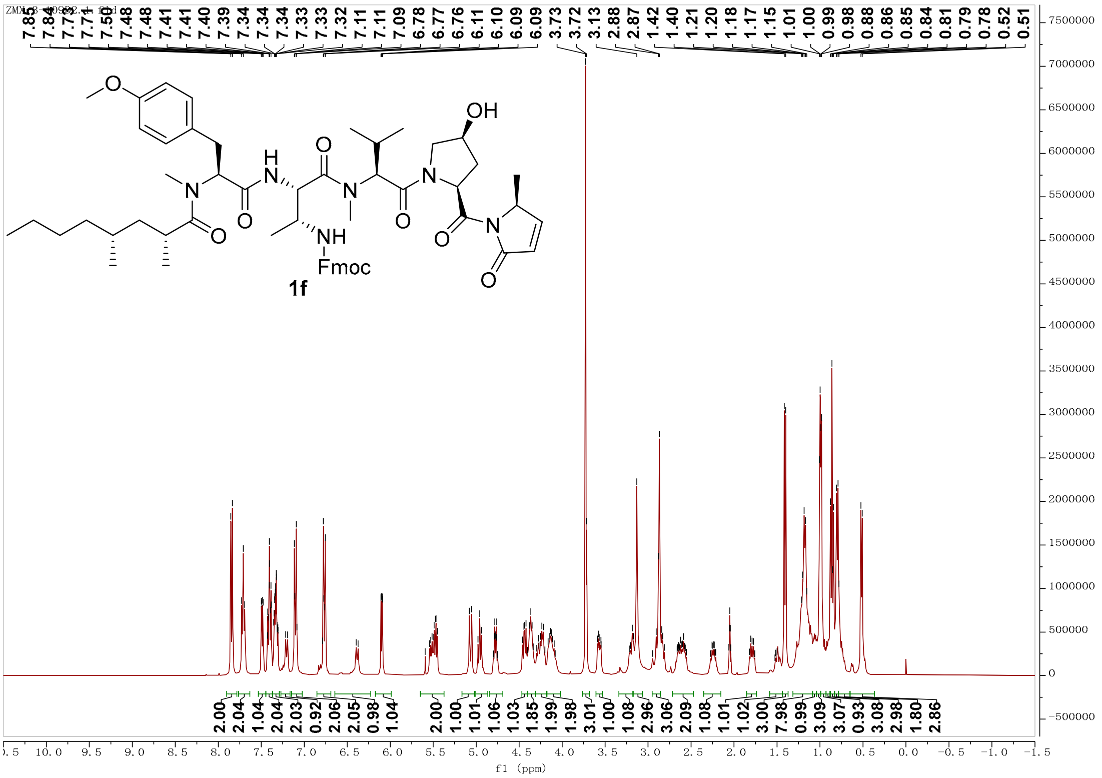

Figure S68. ^{1}H NMR spectrum of compound **1f** in Acetone-*d*6 (400 MHz)

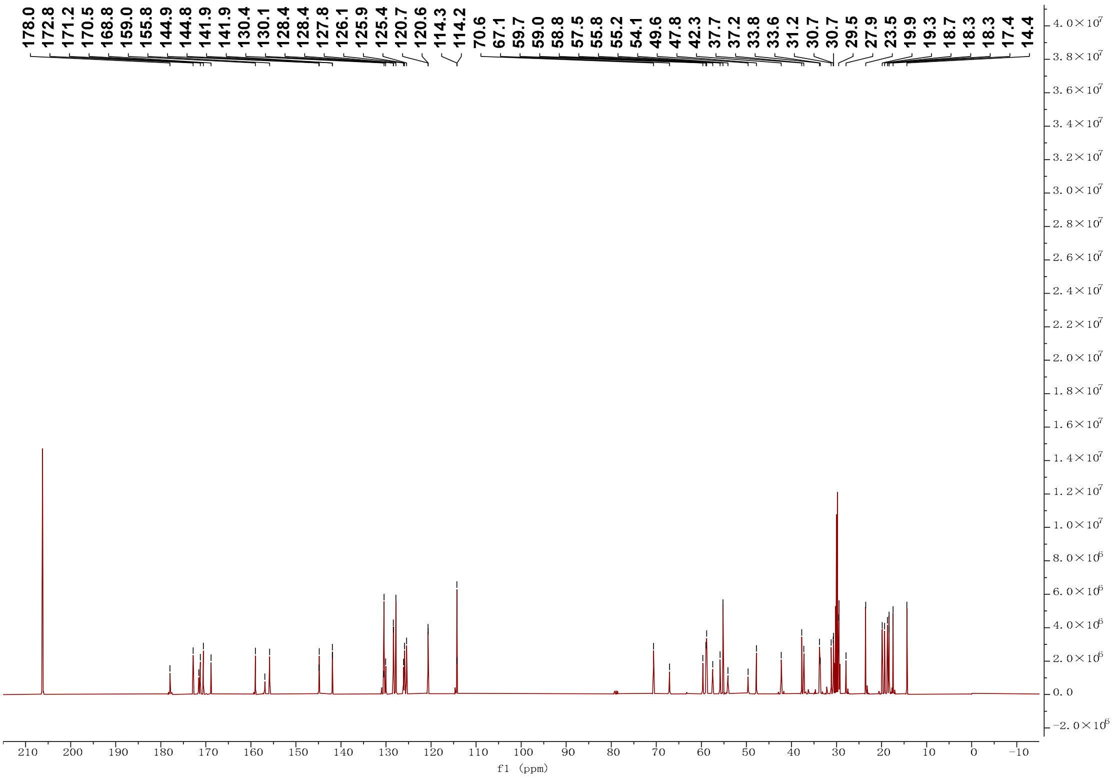

Figure S69. ^{13}C NMR spectrum of compound **1f** in Acetone-*d*6 (100 MHz)

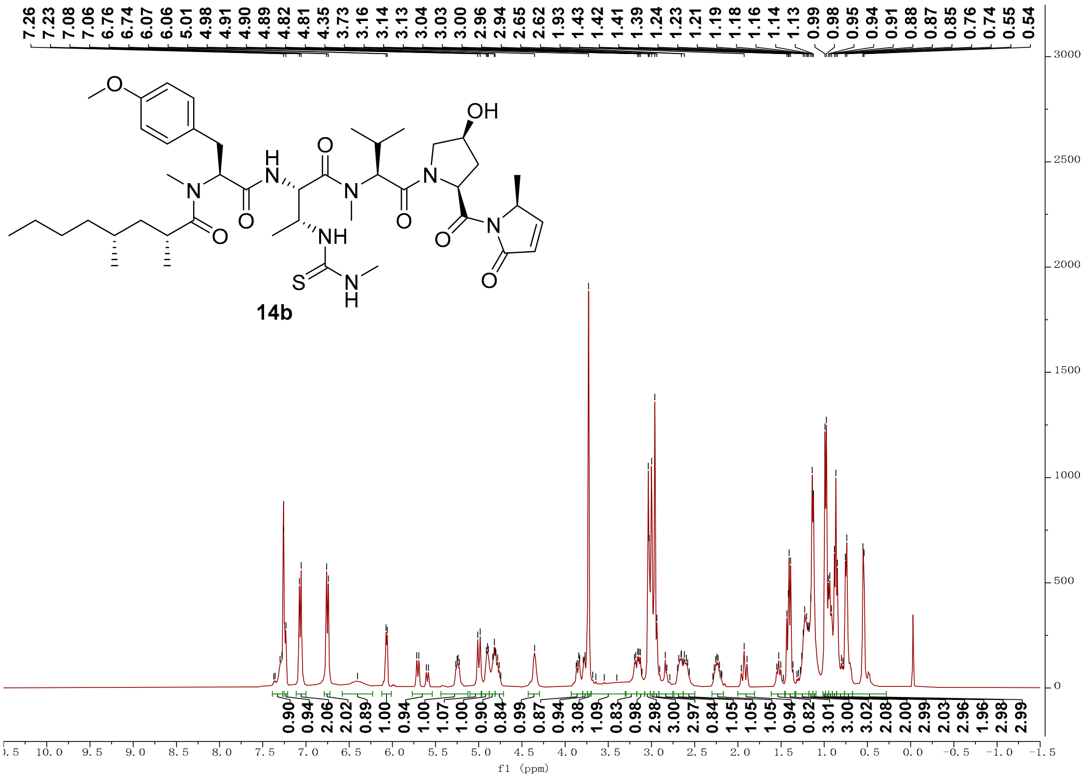

Figure S70. ^{1}H NMR spectrum of compound **14b** in $CDCl_3$ (400 MHz)

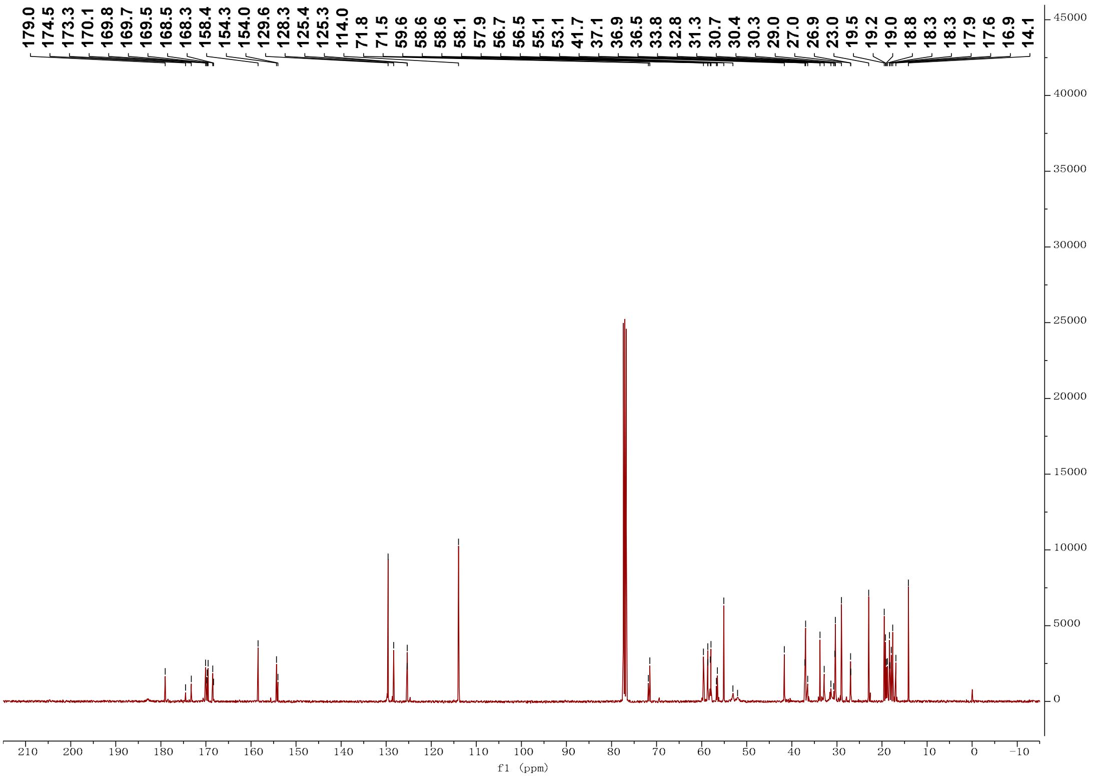

Figure S71. ^{13}C NMR spectrum of compound **14b** in $CDCl_3$ (100 MHz)

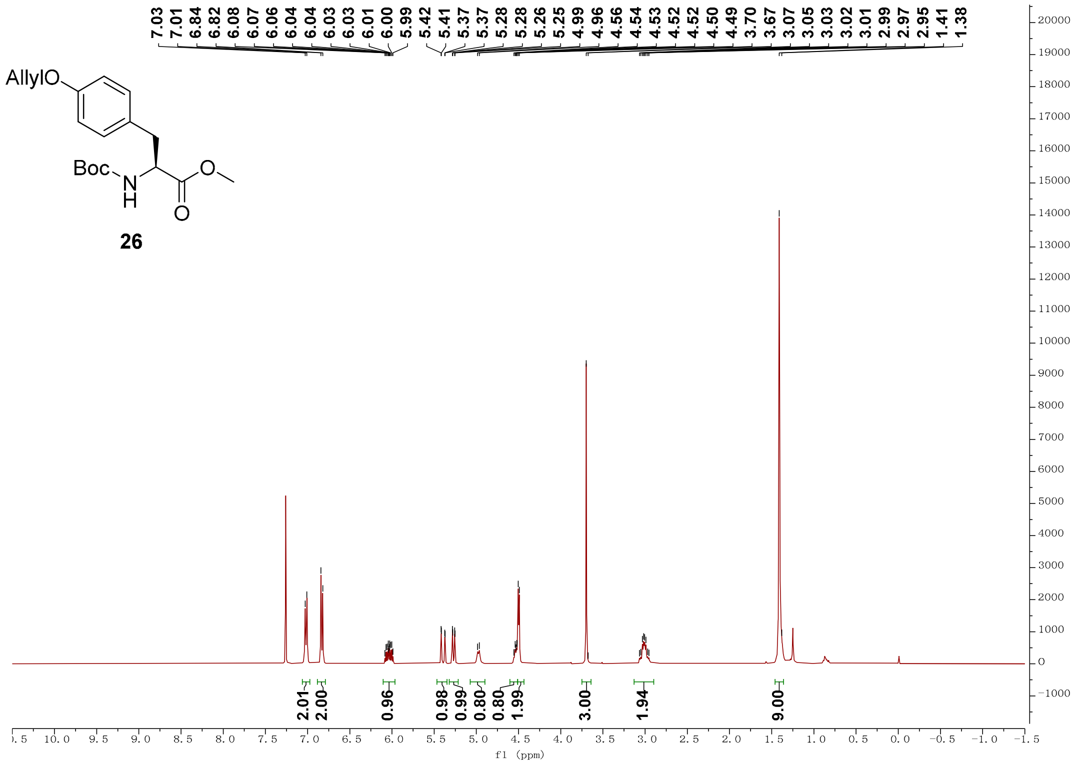

Figure S72. ^{1}H NMR spectrum of compound **26** in $CDCl_3$ (400 MHz)

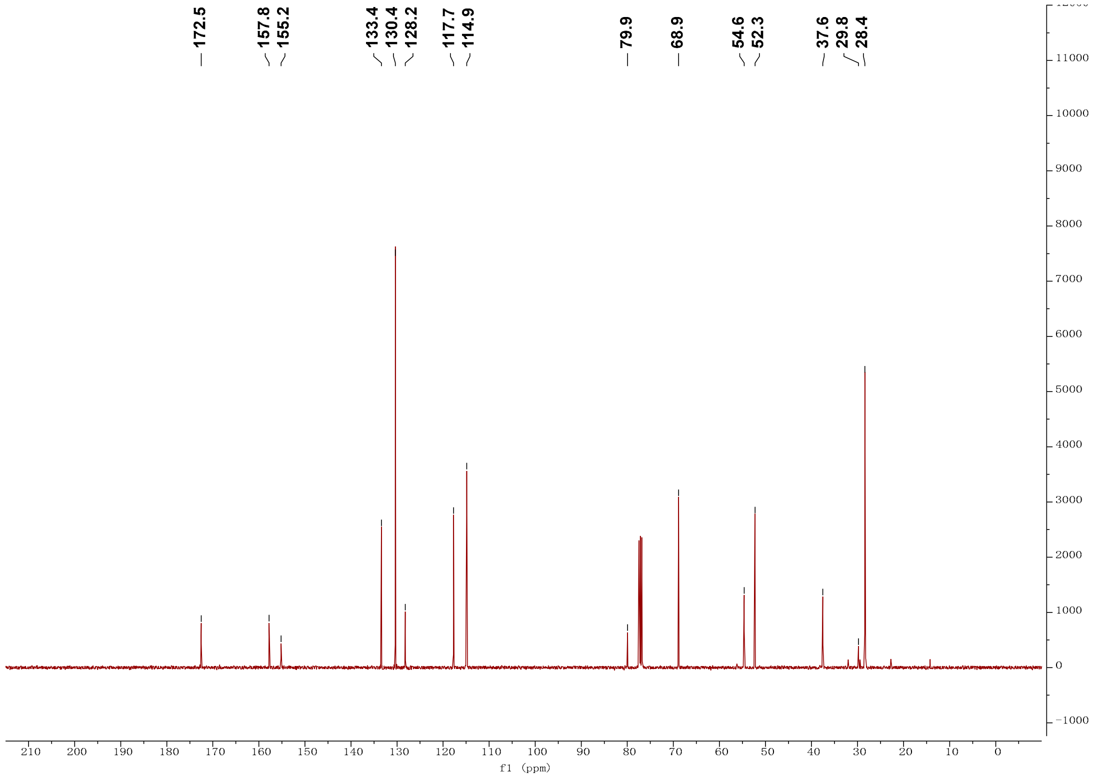

Figure S73. ^{13}C NMR spectrum of compound **26** in $CDCl_3$ (100 MHz)

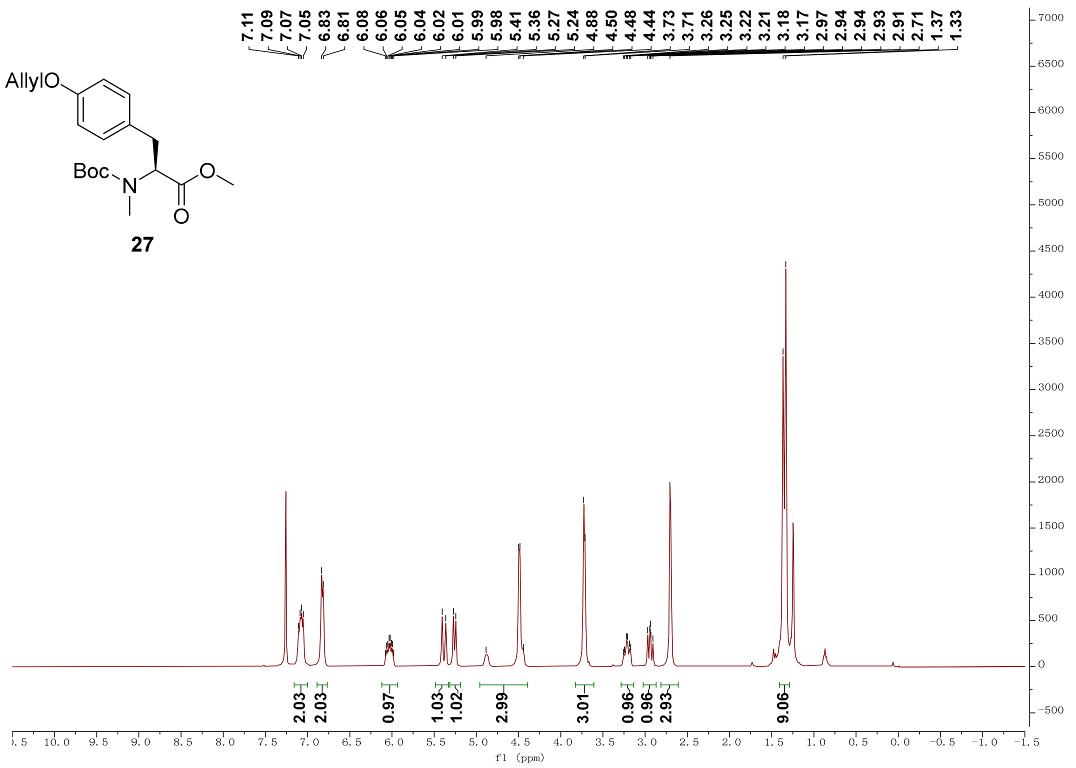

Figure S74. ^{1}H NMR spectrum of compound **27** in $CDCl_3$ (400 MHz)

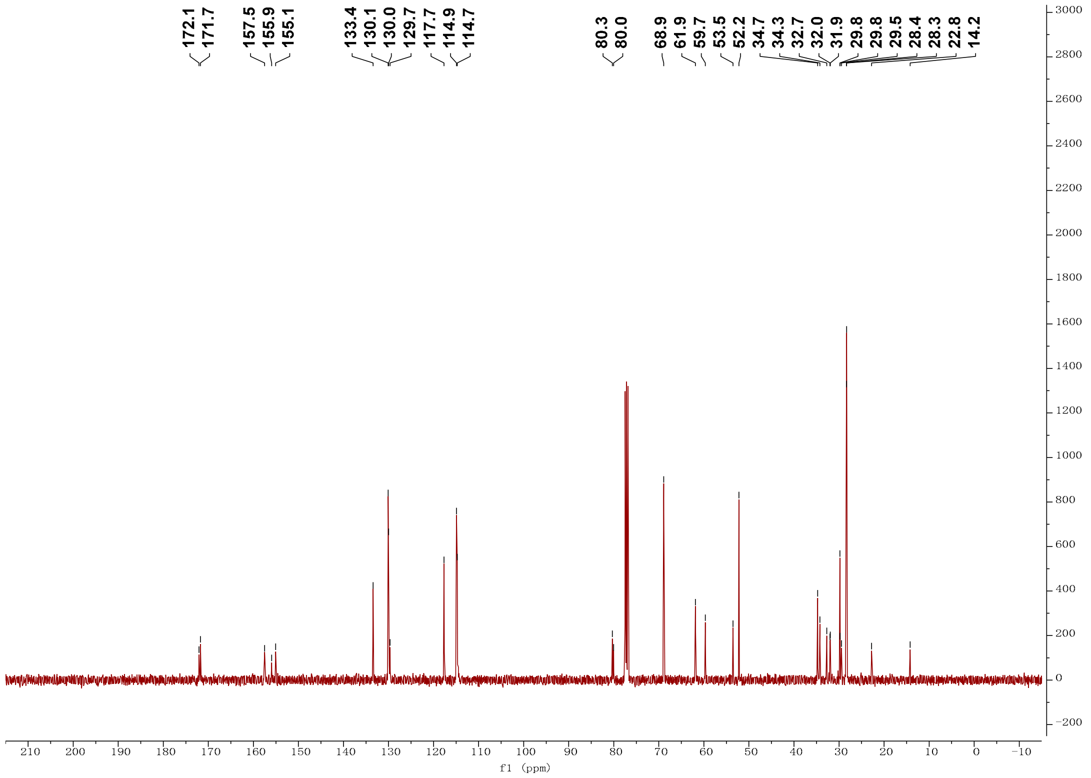

Figure S75. ^{13}C NMR spectrum of compound **27** in $CDCl_3$ (100 MHz)

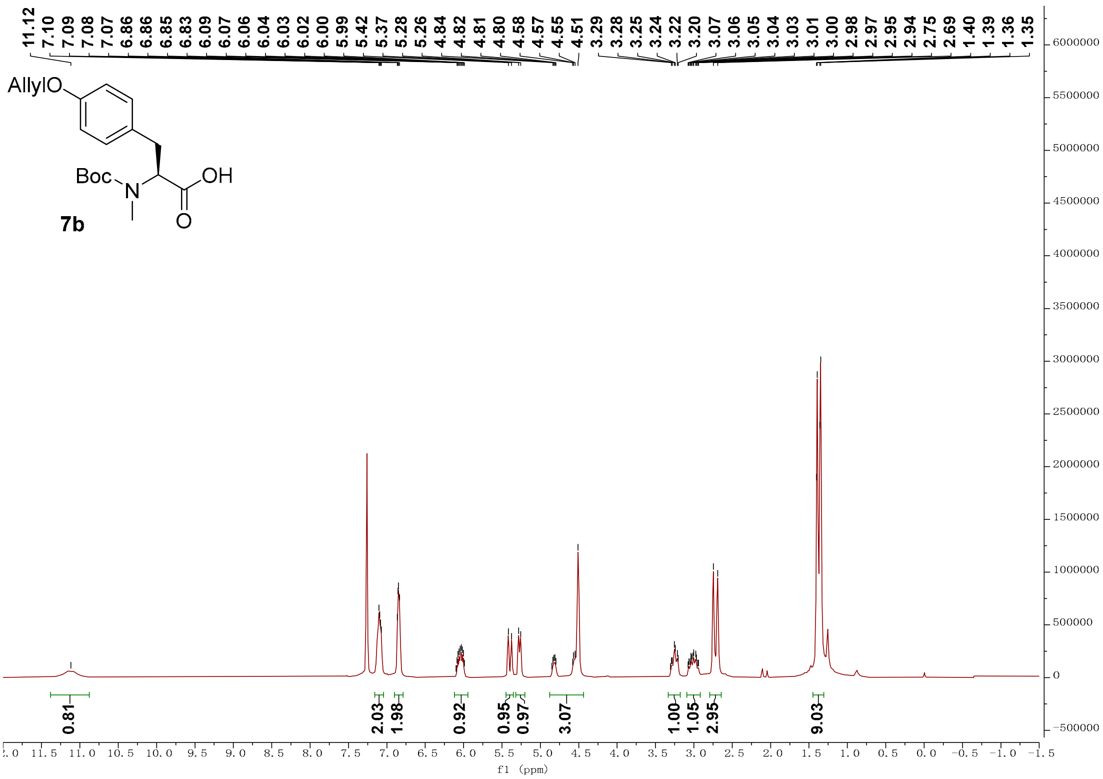

Figure S76. ^{1}H NMR spectrum of compound **7b** in $CDCl_3$ (400 MHz)

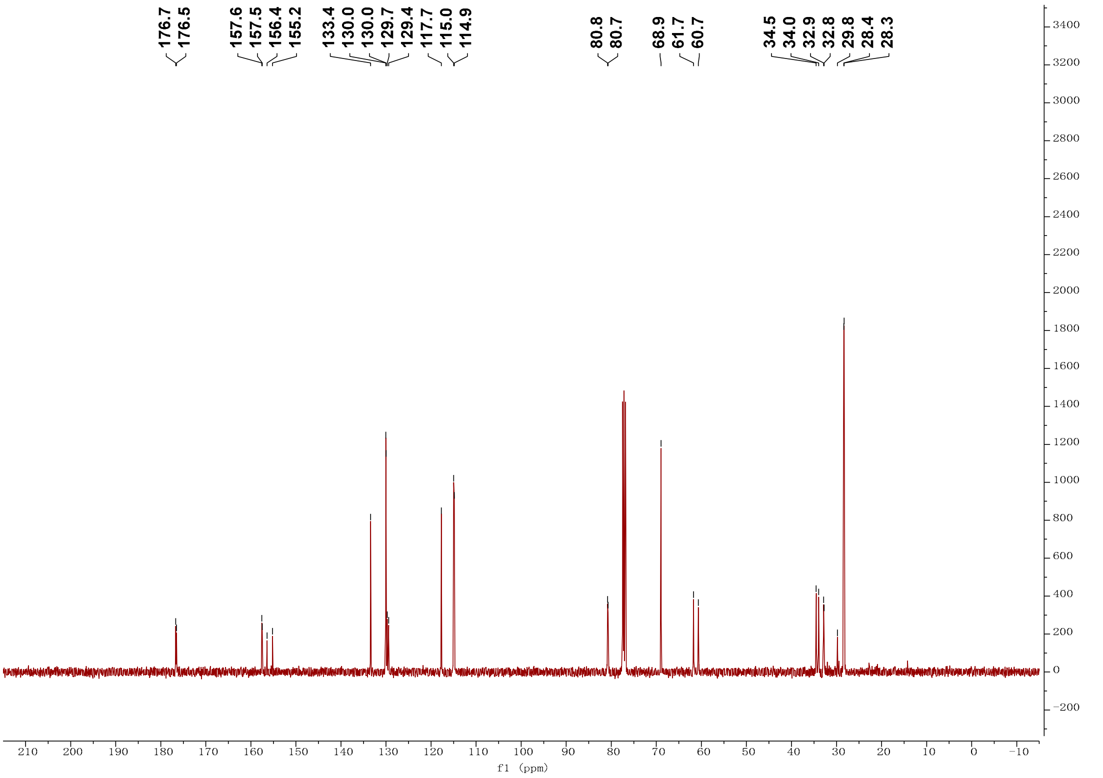

Figure S77. ^{13}C NMR spectrum of compound **7b** in $CDCl_3$ (100 MHz)

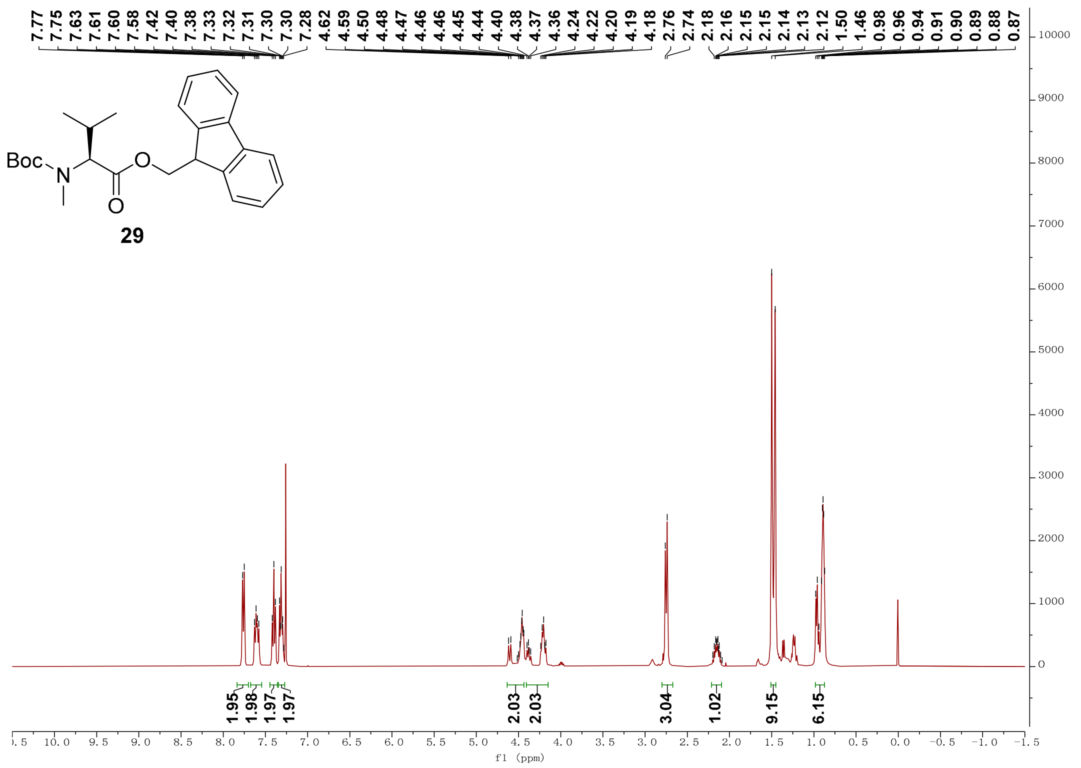

Figure S78. ^{1}H NMR spectrum of compound **29** in $CDCl_3$ (400 MHz)

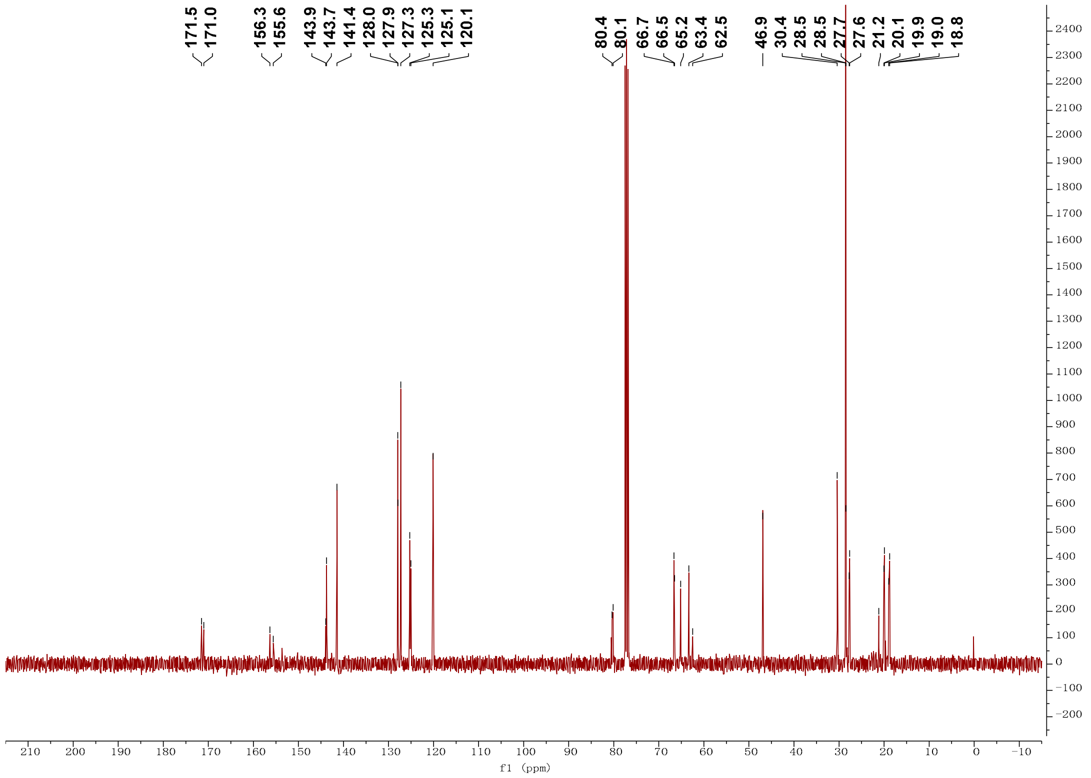

Figure S79. ^{13}C NMR spectrum of compound **29** in $CDCl_3$ (100 MHz)

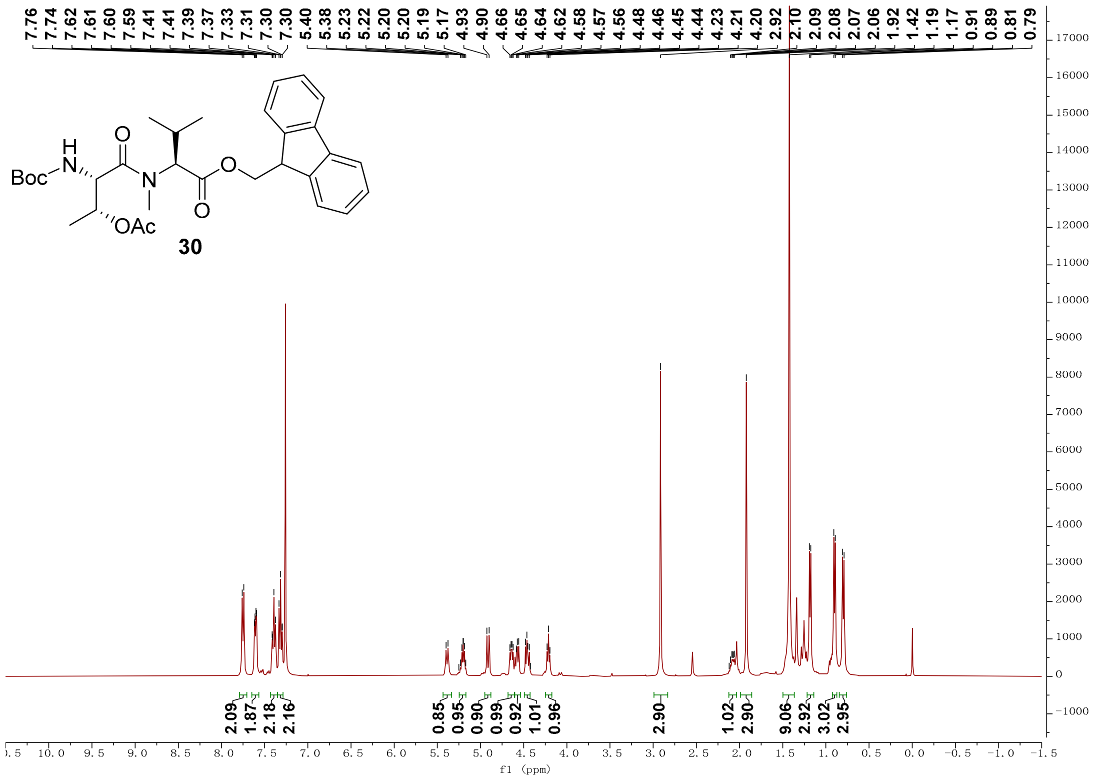

Figure S80. ^{1}H NMR spectrum of compound **30** in $CDCl_3$ (400 MHz)

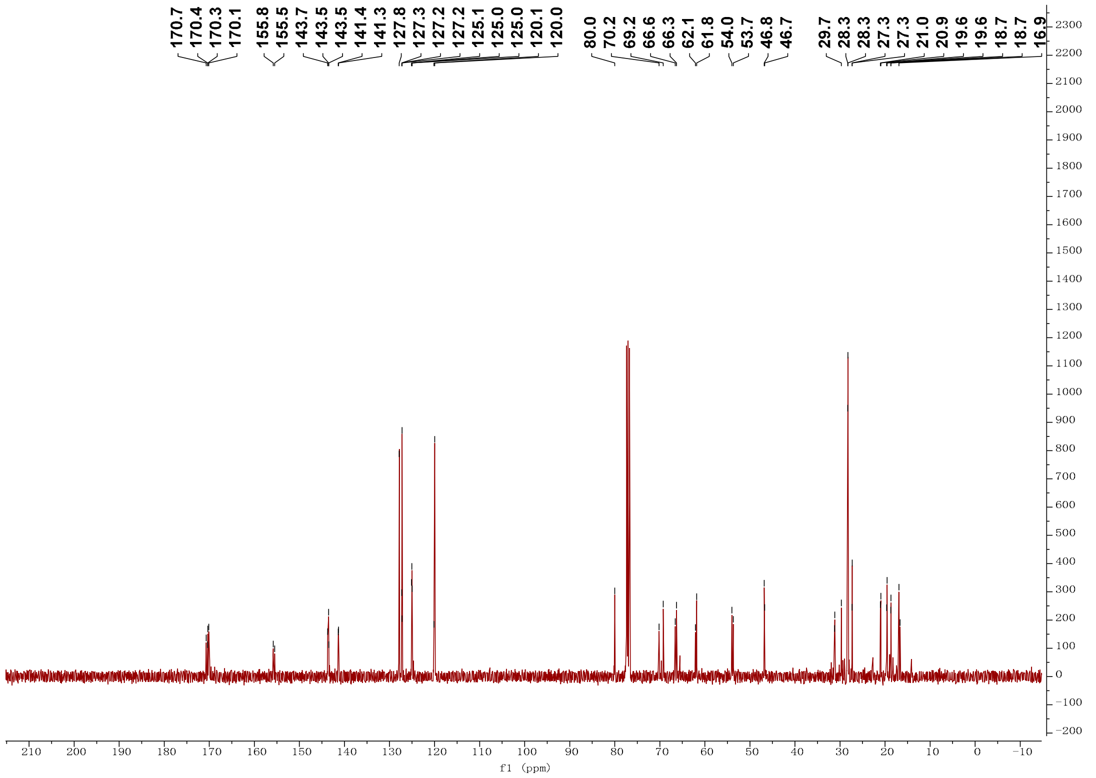

Figure S81. ^{13}C NMR spectrum of compound **30** in $CDCl_3$ (100 MHz)

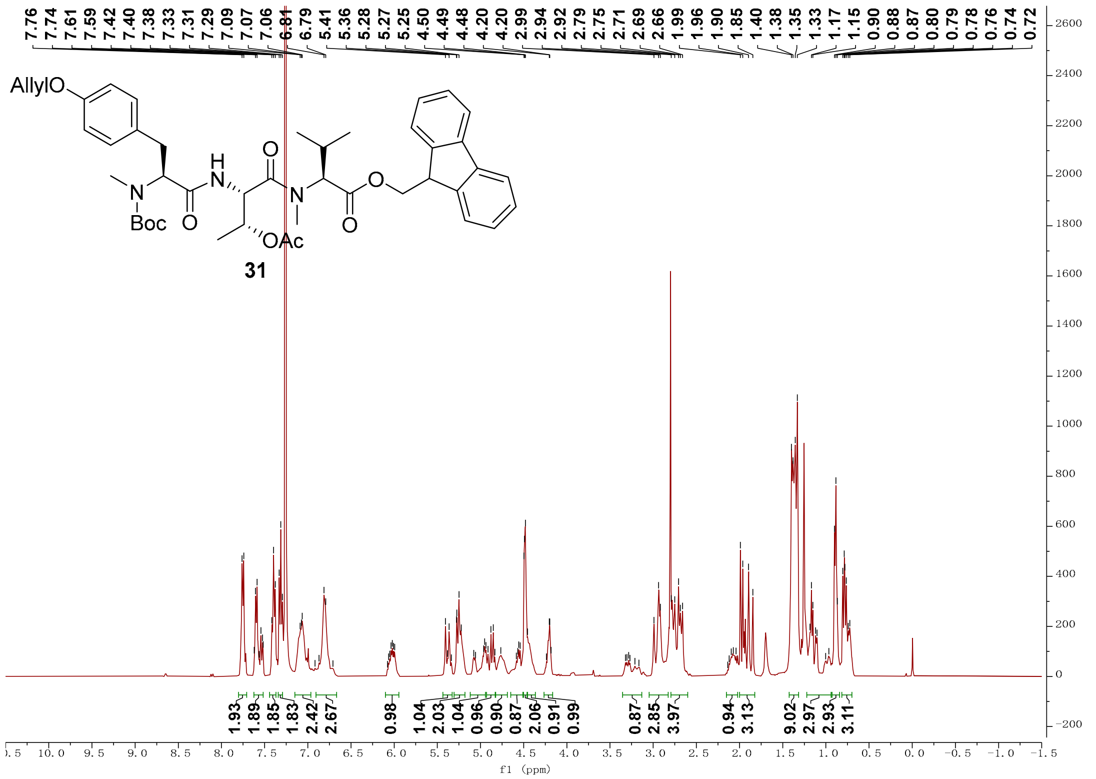

Figure S82. ^{1}H NMR spectrum of compound **31** in $CDCl_3$ (400 MHz)

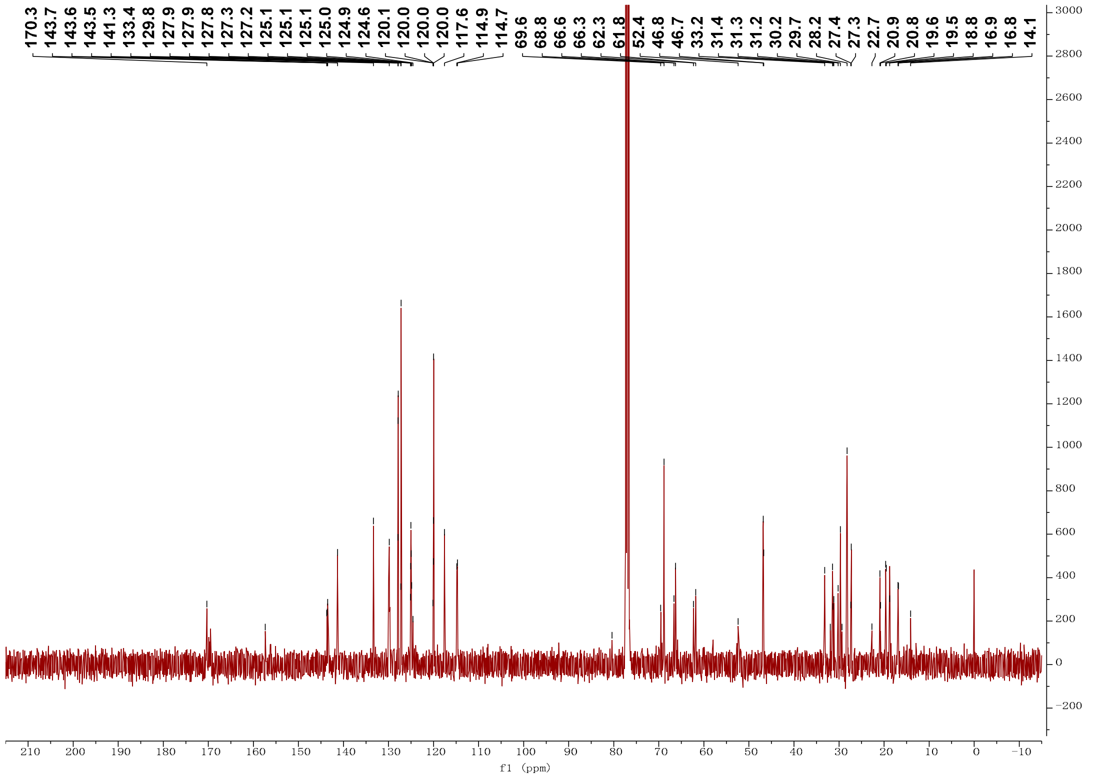

Figure S83. ^{13}C NMR spectrum of compound **31** in $CDCl_3$ (100 MHz)

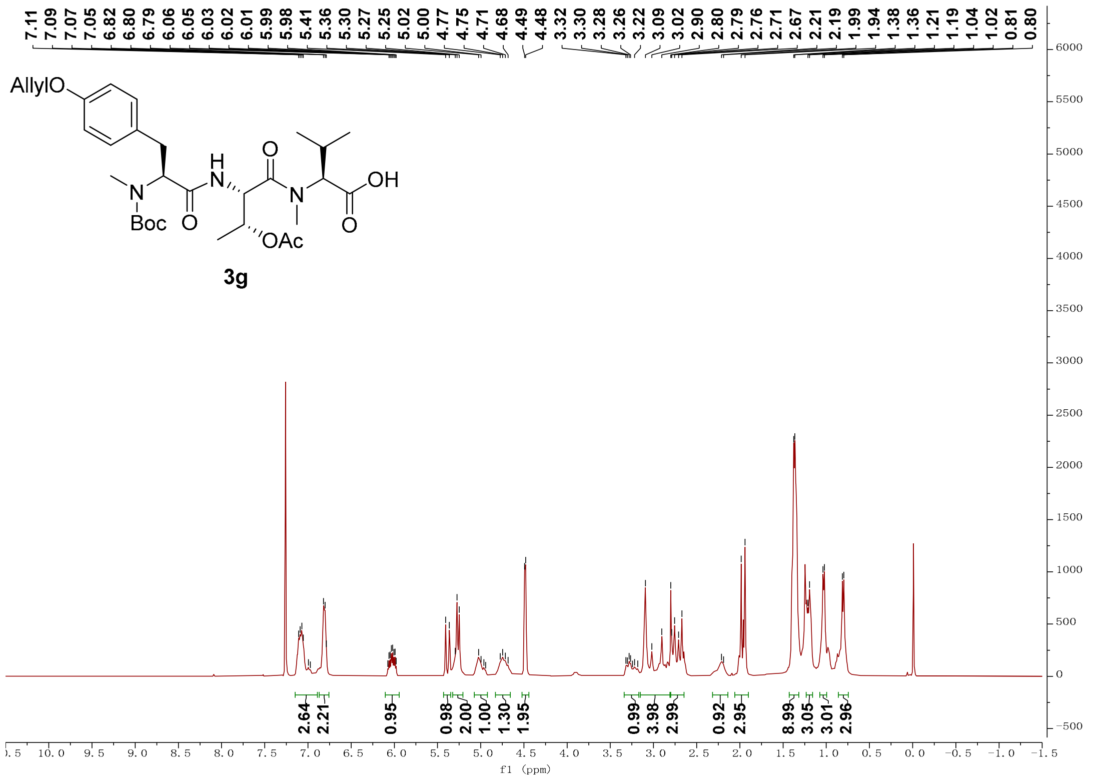

Figure S84. ^{1}H NMR spectrum of compound **3g** in $CDCl_3$ (400 MHz)

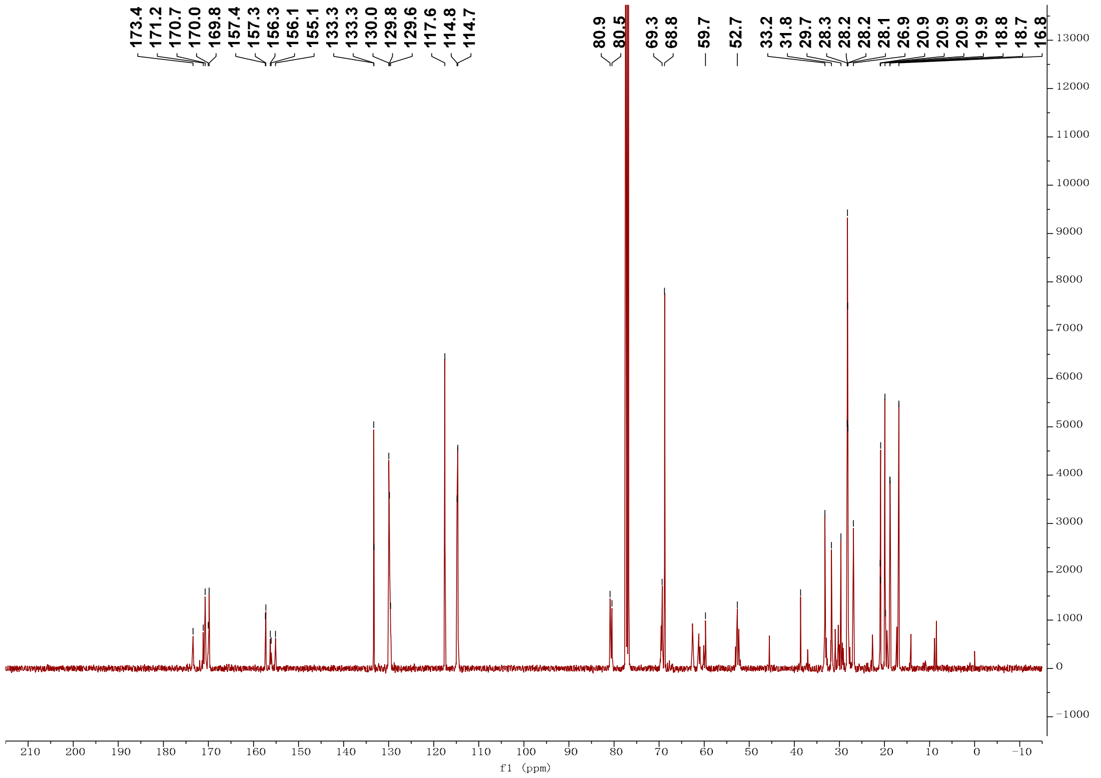

Figure S85. ^{13}C NMR spectrum of compound **3g** in $CDCl_3$ (100 MHz)

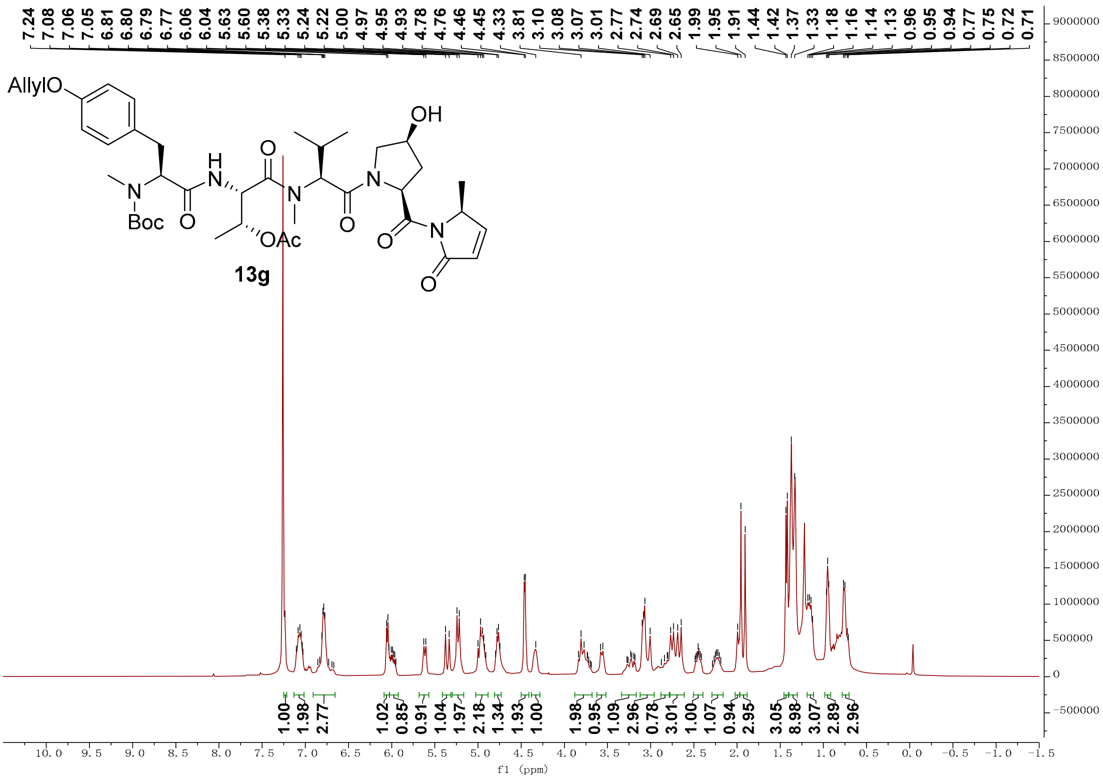

Figure S86. ^{1}H NMR spectrum of compound **13g** in $CDCl_3$ (400 MHz)

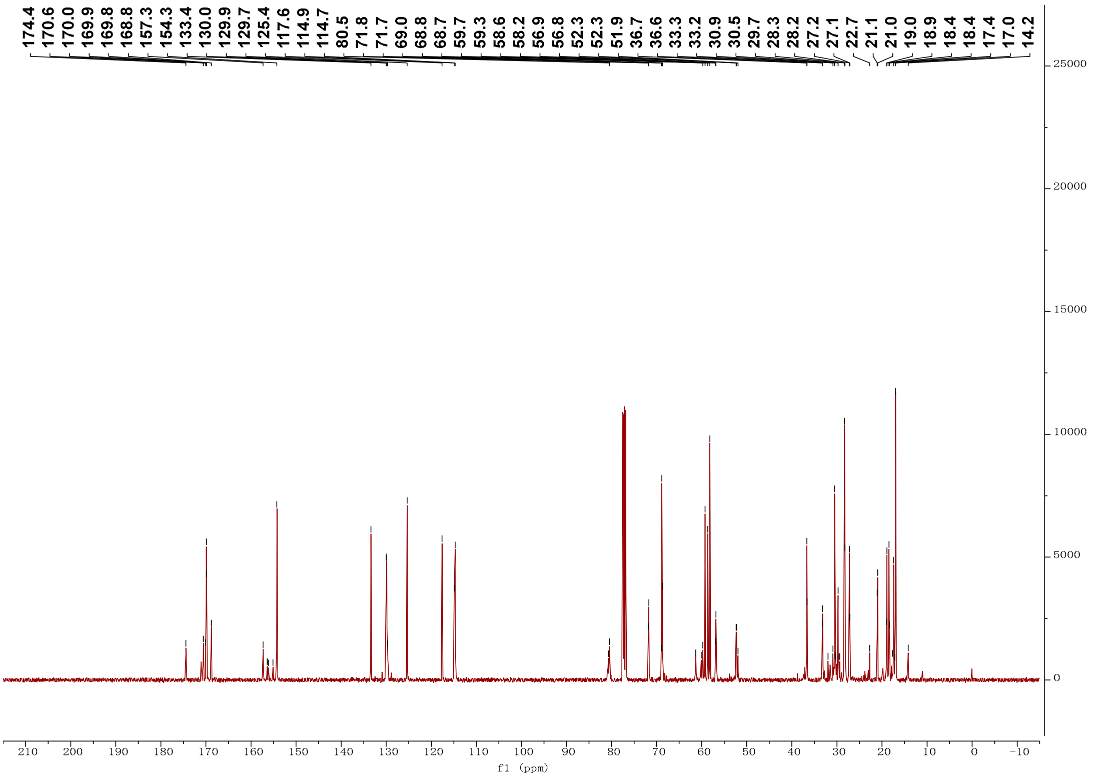

Figure S87. ^{13}C NMR spectrum of compound **13g** in $CDCl_3$ (100 MHz)

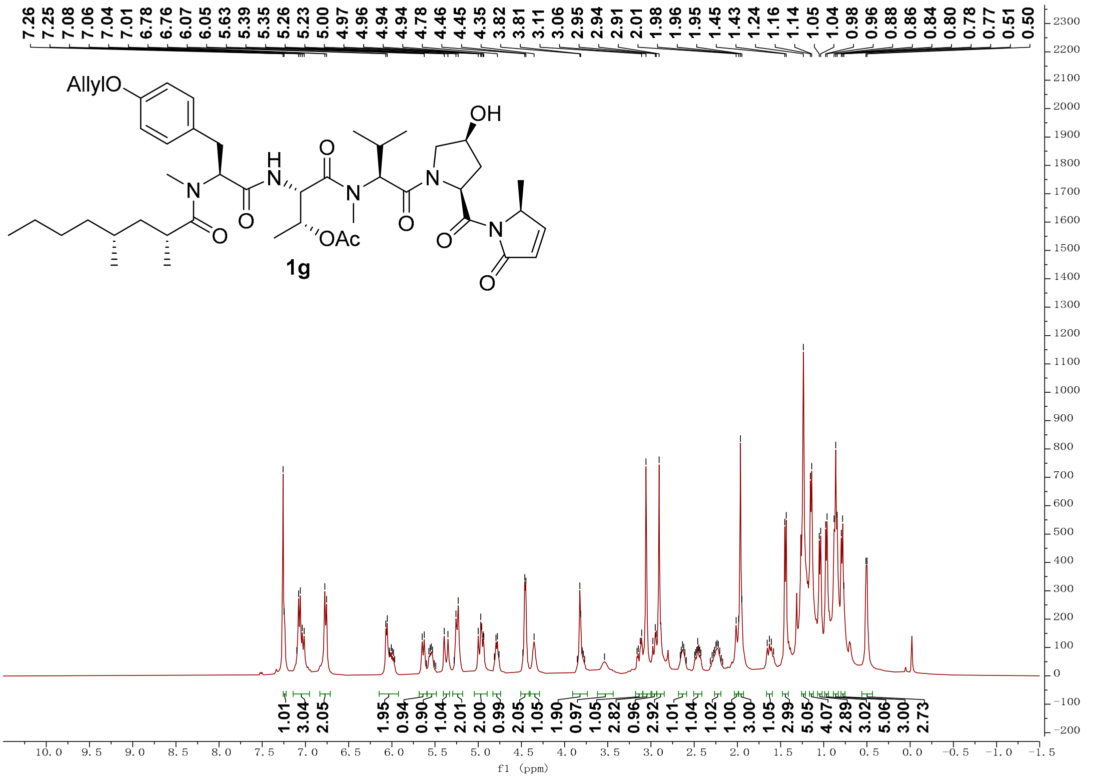

Figure S88. ^{1}H NMR spectrum of compound **1g** in $CDCl_3$ (400 MHz)

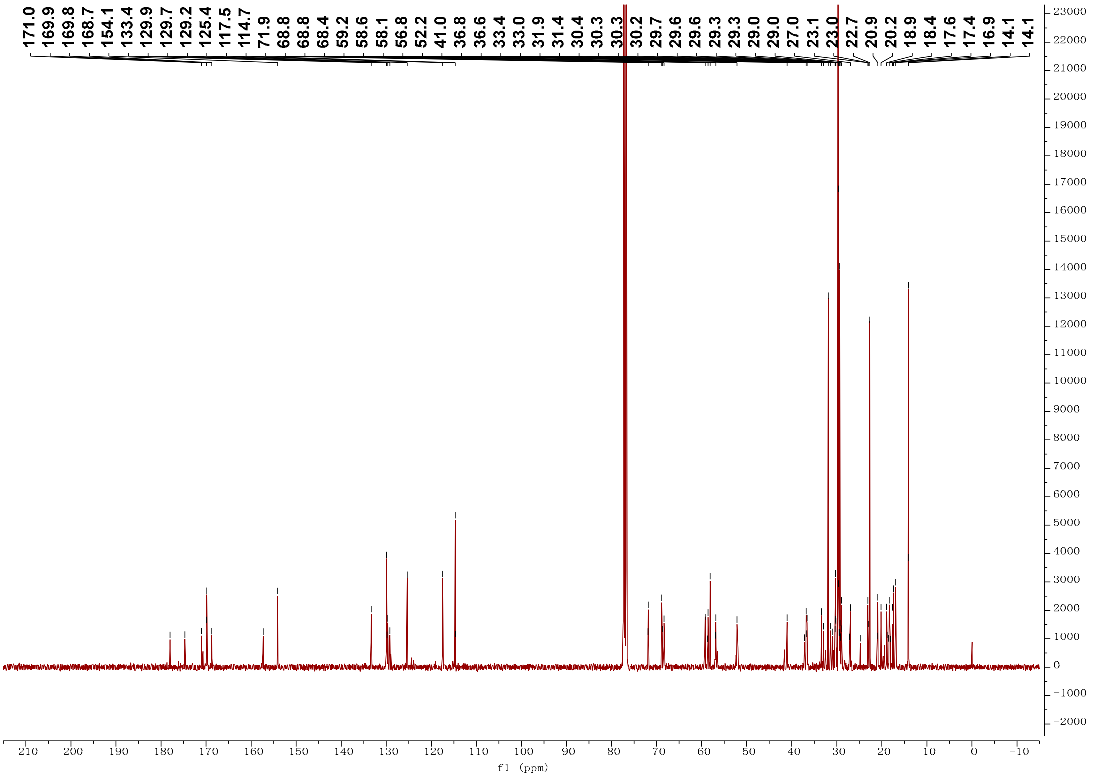

Figure S89. ^{13}C NMR spectrum of compound **1g** in $CDCl_3$ (100 MHz)

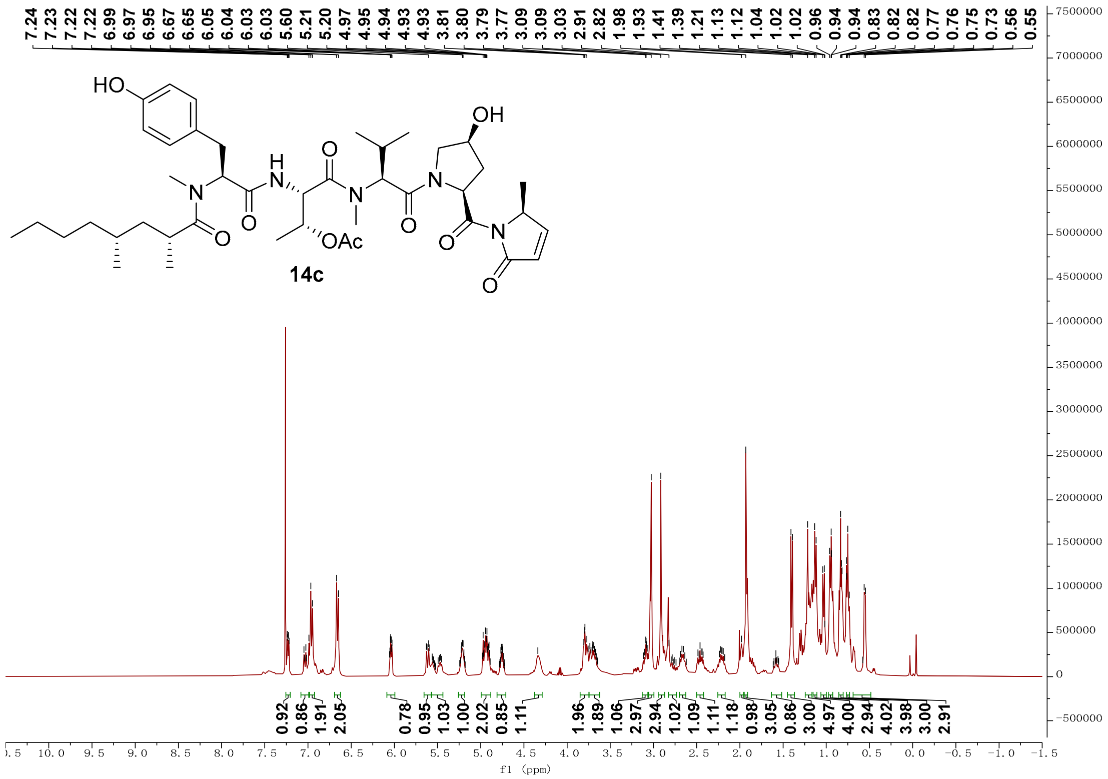

Figure S90. ^{1}H NMR spectrum of compound **14c** in $CDCl_3$ (400 MHz)

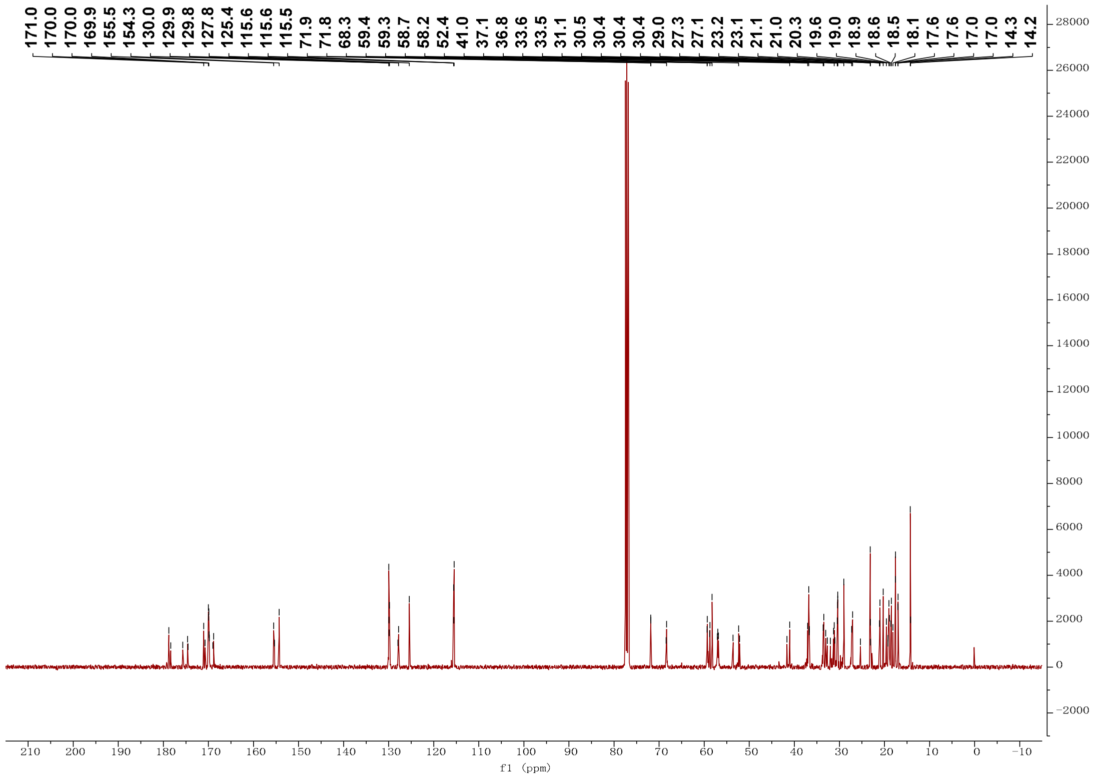

Figure S91. ^{13}C NMR spectrum of compound **14c** in $CDCl_3$ (100 MHz)

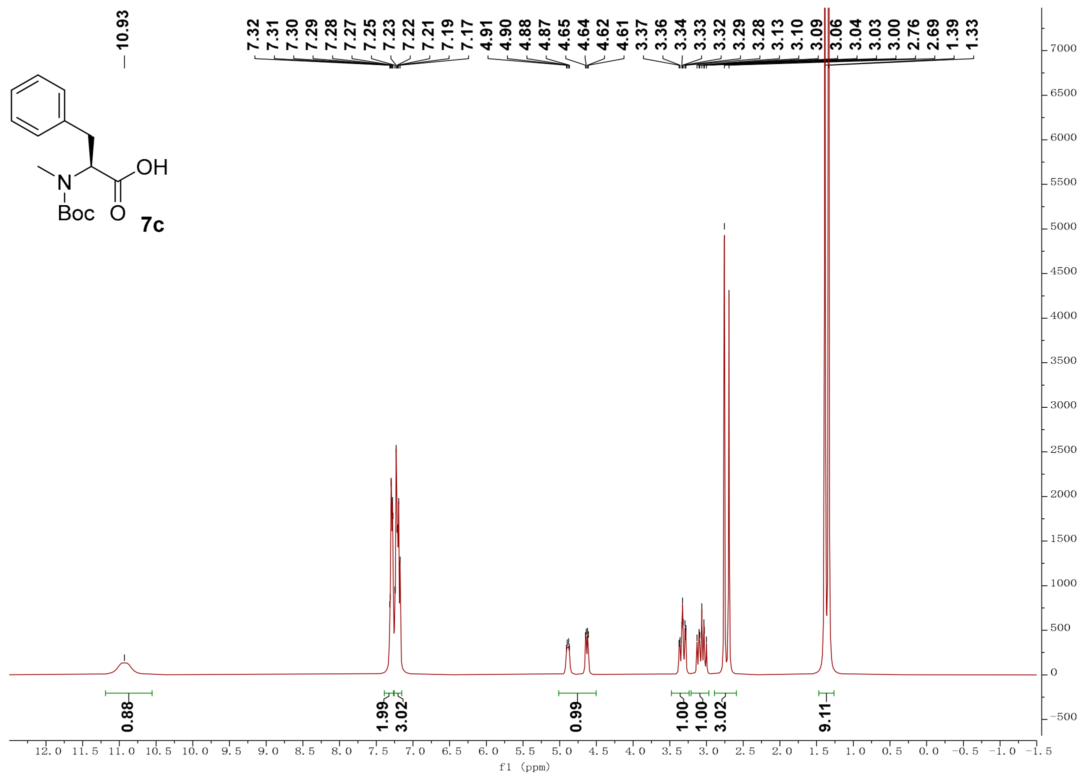

Figure S92. ^{1}H NMR spectrum of compound **7c** in $CDCl_3$ (400 MHz)

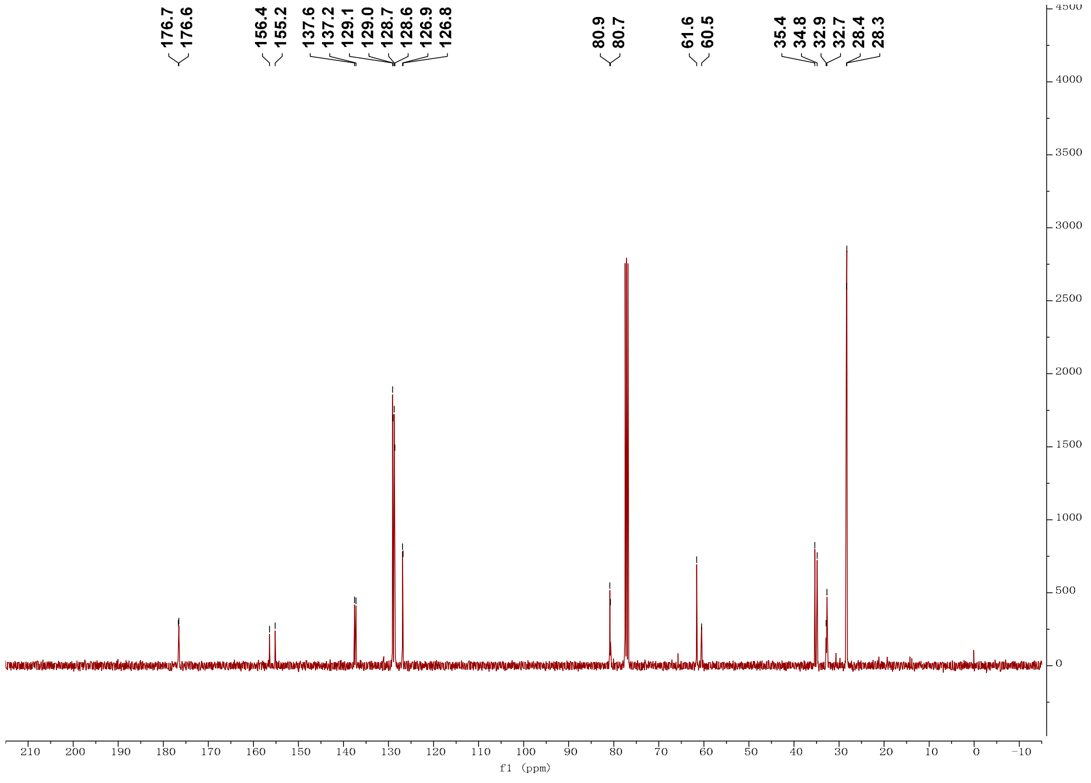

Figure S93. ^{13}C NMR spectrum of compound **7c** in $CDCl_3$ (100 MHz)

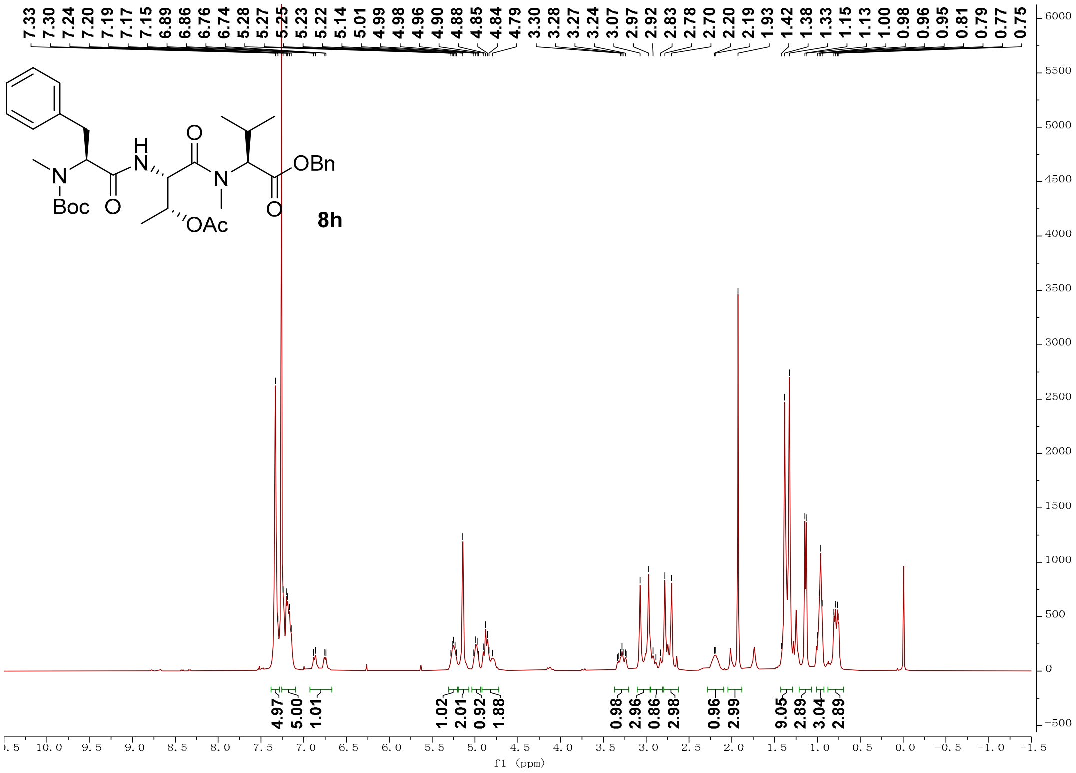

Figure S94. ^{1}H NMR spectrum of compound **8h** in $CDCl_3$ (400 MHz)

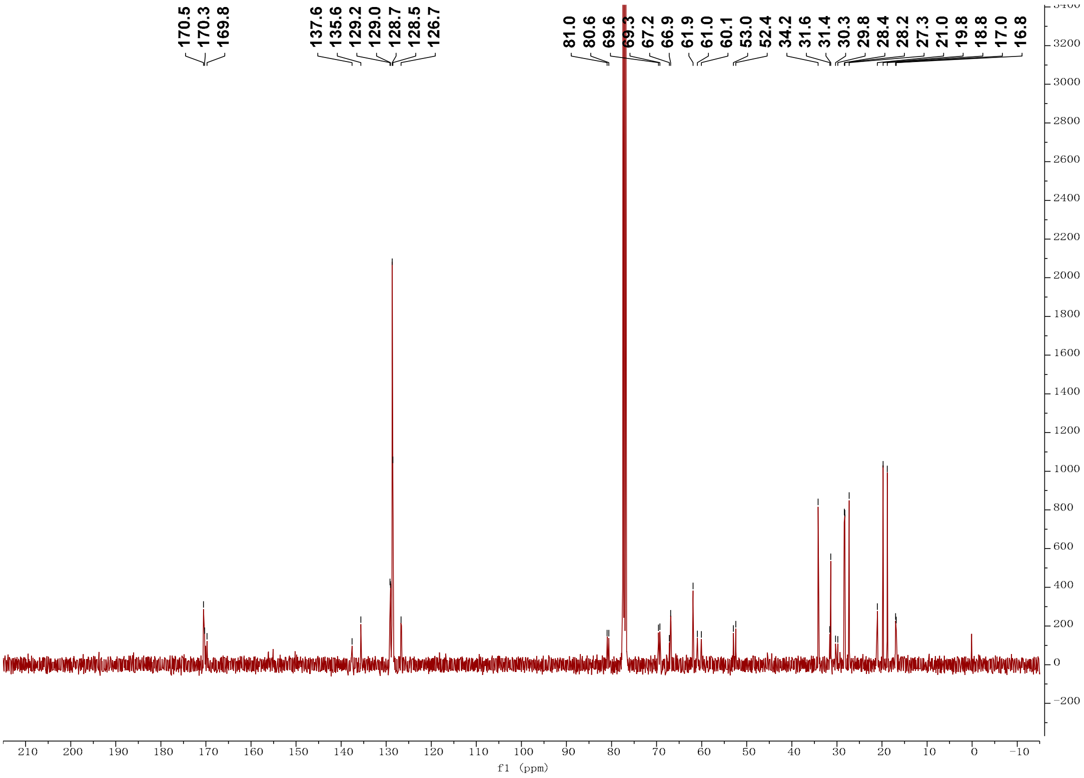

Figure S95. ^{13}C NMR spectrum of compound **8h** in $CDCl_3$ (100 MHz)

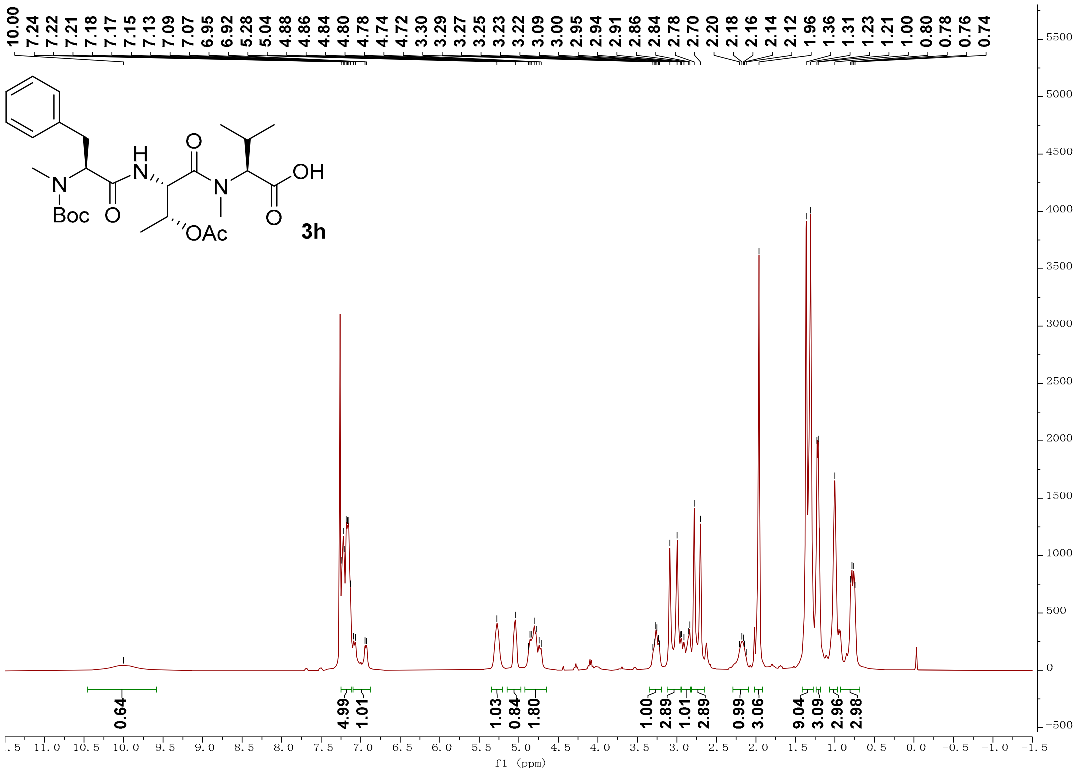

Figure S96. ^{1}H NMR spectrum of compound **3h** in $CDCl_3$ (400 MHz)

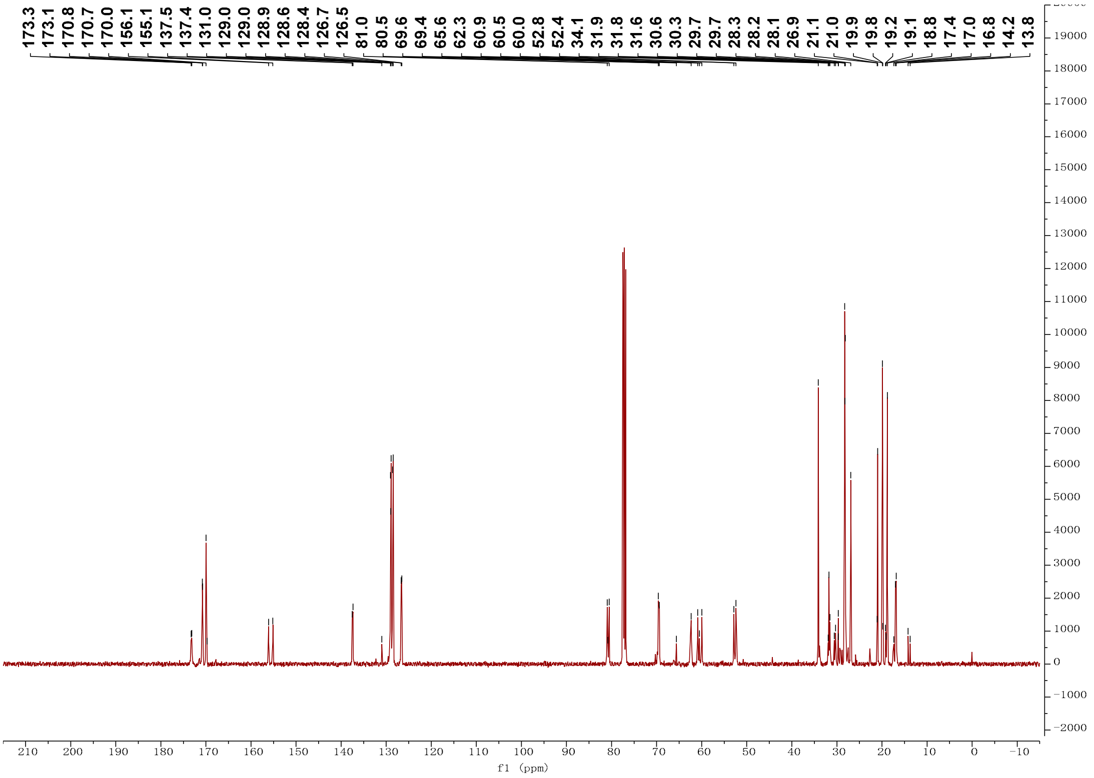

Figure S97. ^{13}C NMR spectrum of compound **3h** in $CDCl_3$ (100 MHz)

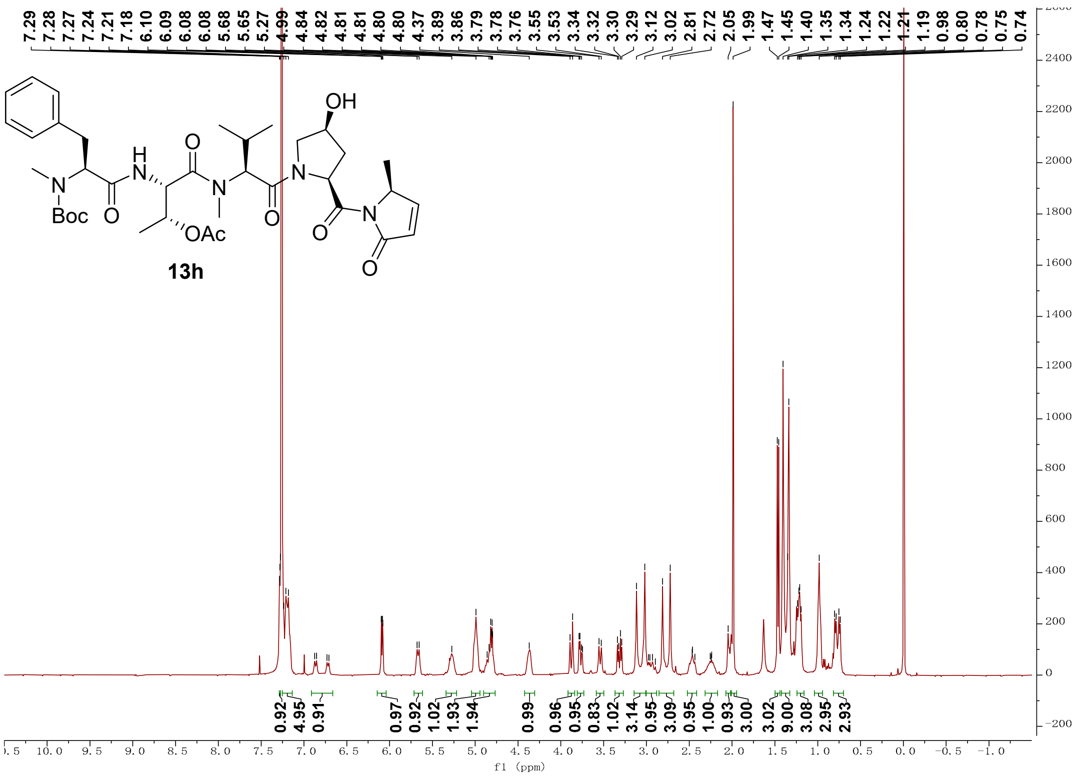

Figure S98. ^{1}H NMR spectrum of compound **13h** in $CDCl_3$ (400 MHz)

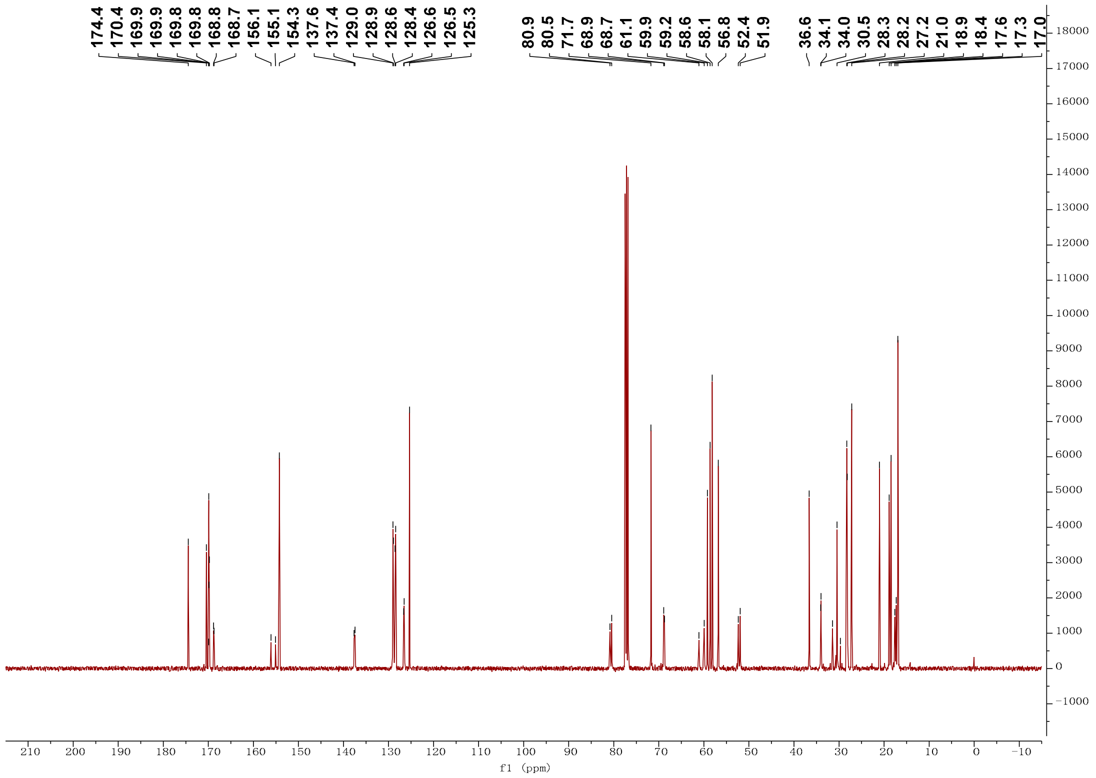

Figure S99. ^{13}C NMR spectrum of compound **13h** in $CDCl_3$ (100 MHz)

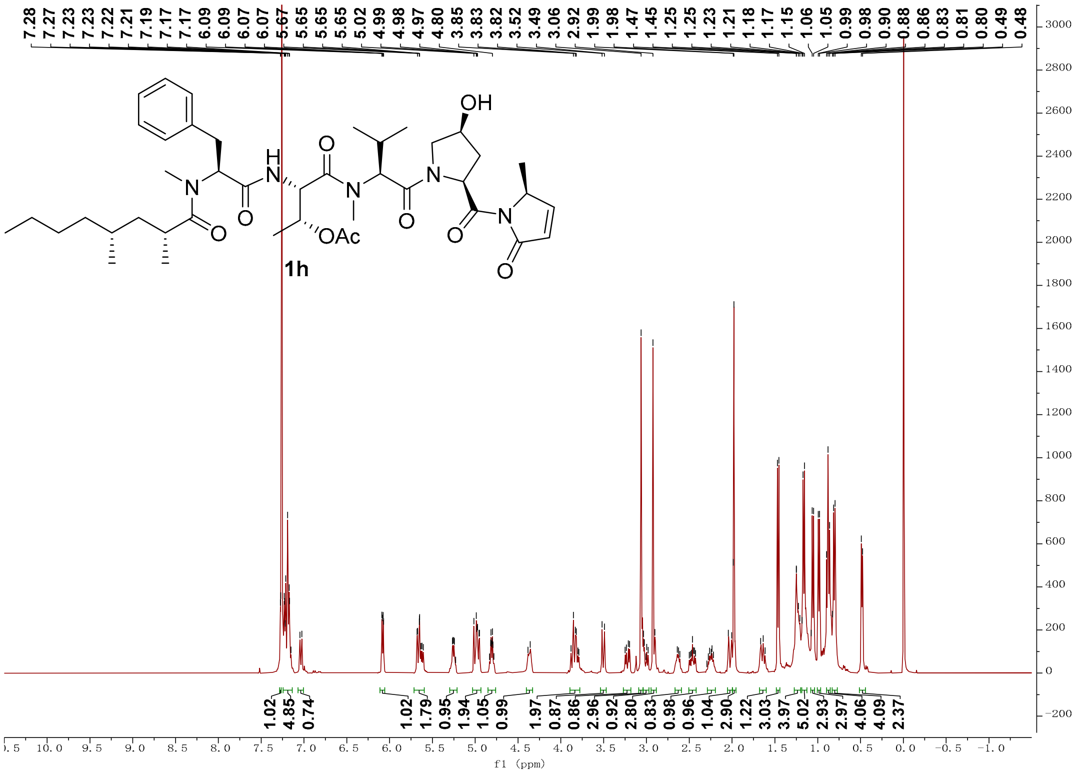

Figure S100. ^{1}H NMR spectrum of compound **1h** in $CDCl_3$ (400 MHz)

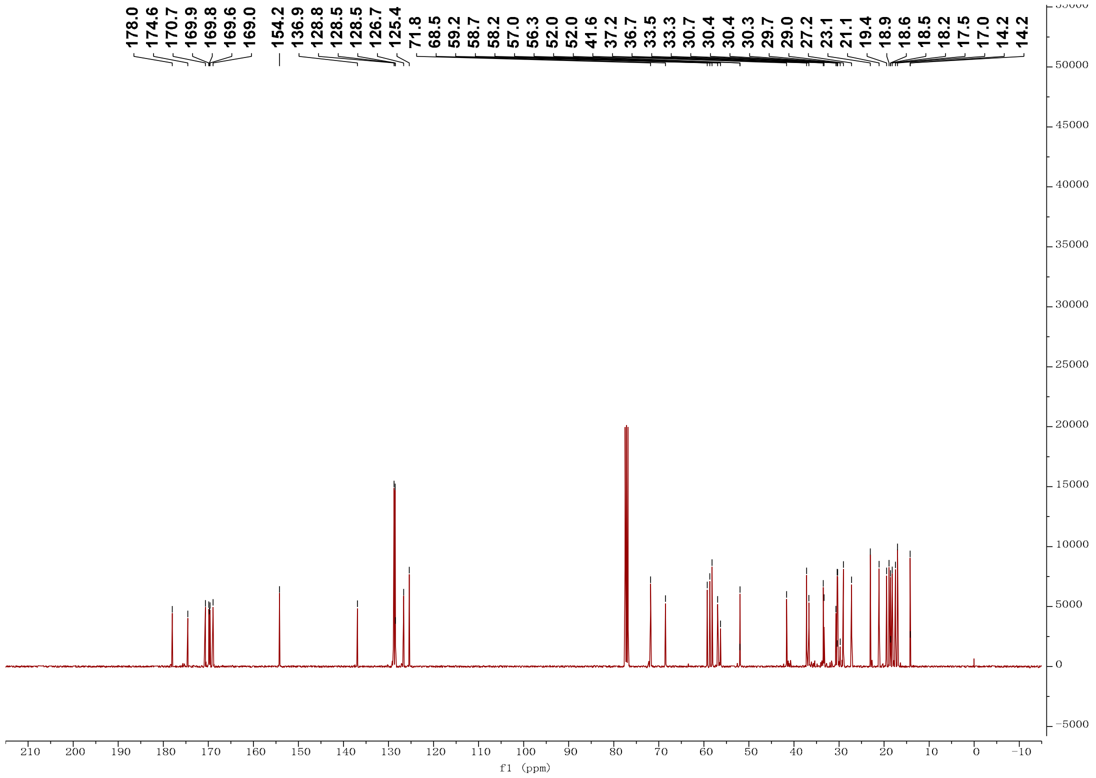

Figure S101. ^{13}C NMR spectrum of compound **1h** in $CDCl_3$ (100 MHz)

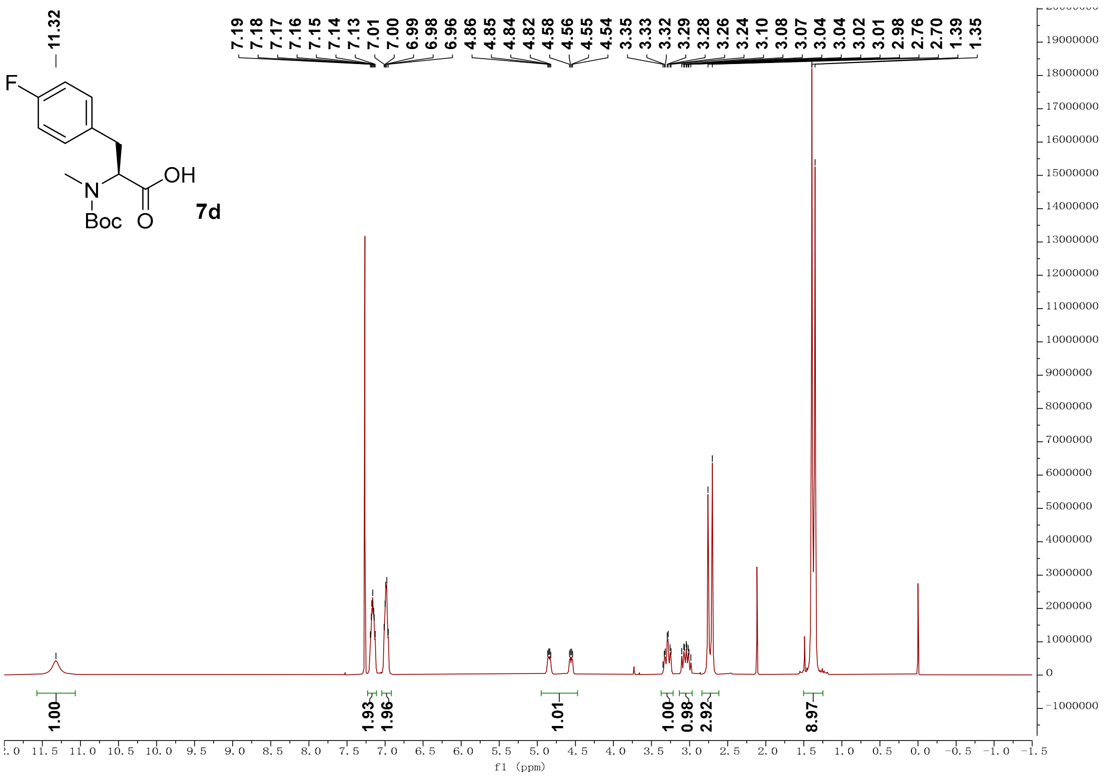

Figure S102. ^{1}H NMR spectrum of compound **7d** in $CDCl_3$ (400 MHz)

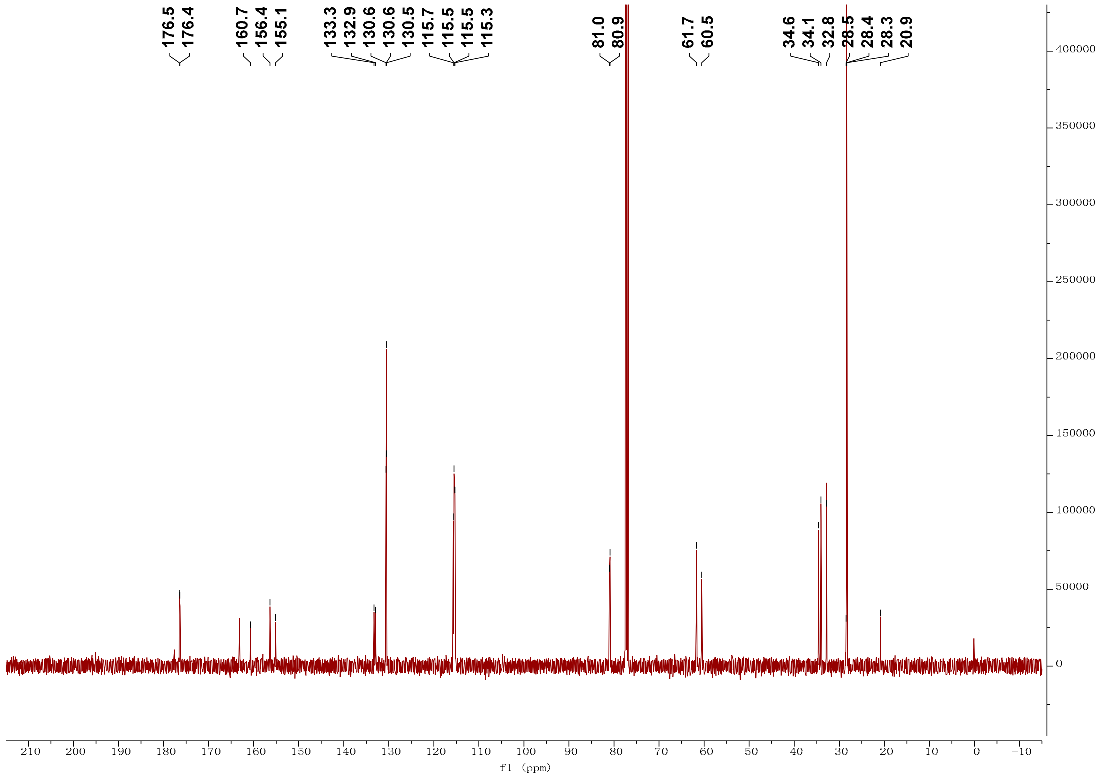

Figure S103. ^{13}C NMR spectrum of compound **7d** in $CDCl_3$ (100 MHz)

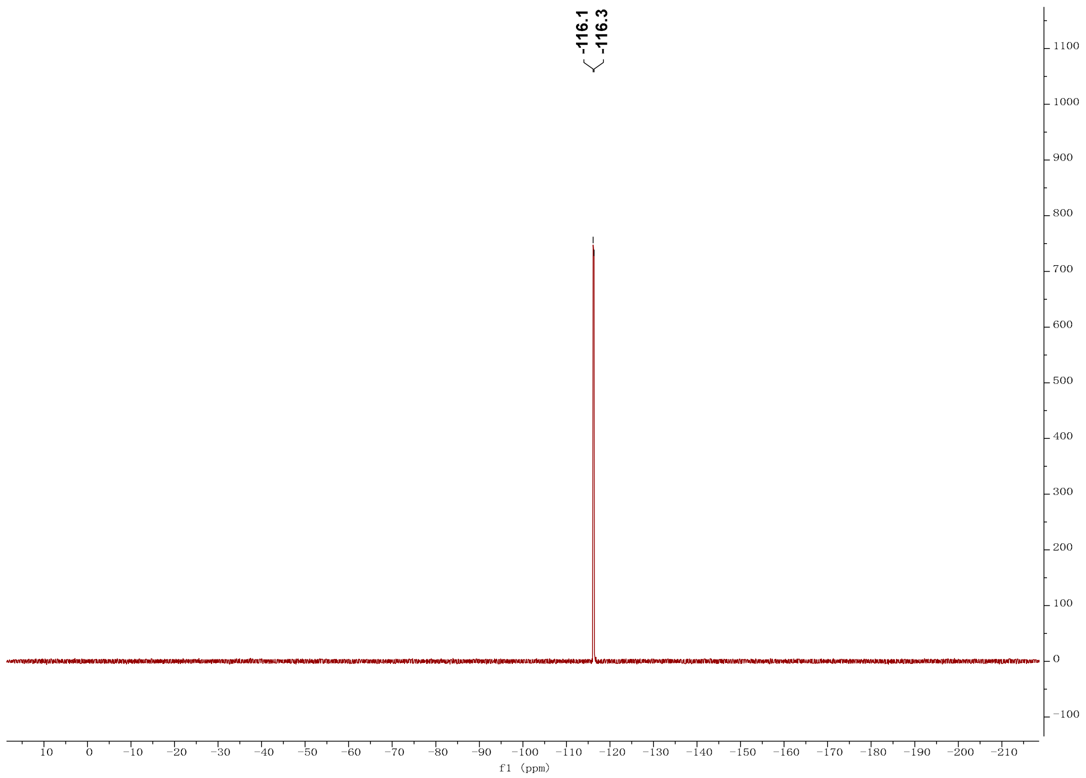

Figure S104. ^{19}F NMR spectrum of compound **7d** in $CDCl_3$ (376 MHz)

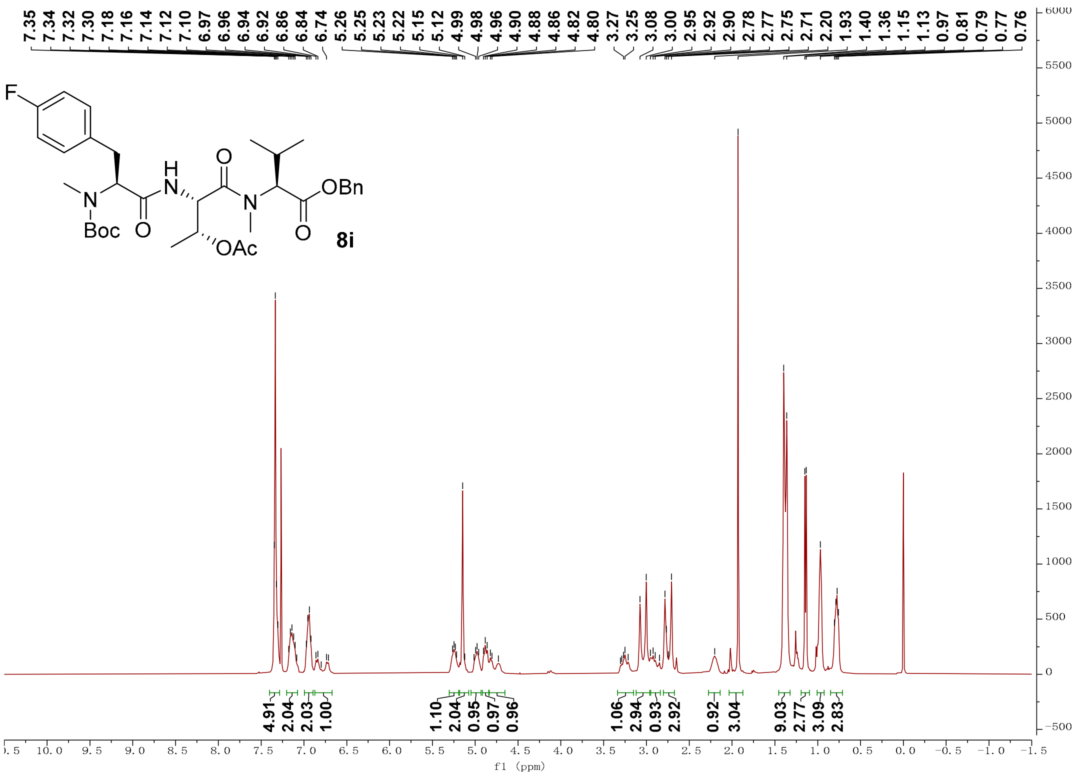

Figure S105. ^{1}H NMR spectrum of compound **8i** in $CDCl_3$ (400 MHz)

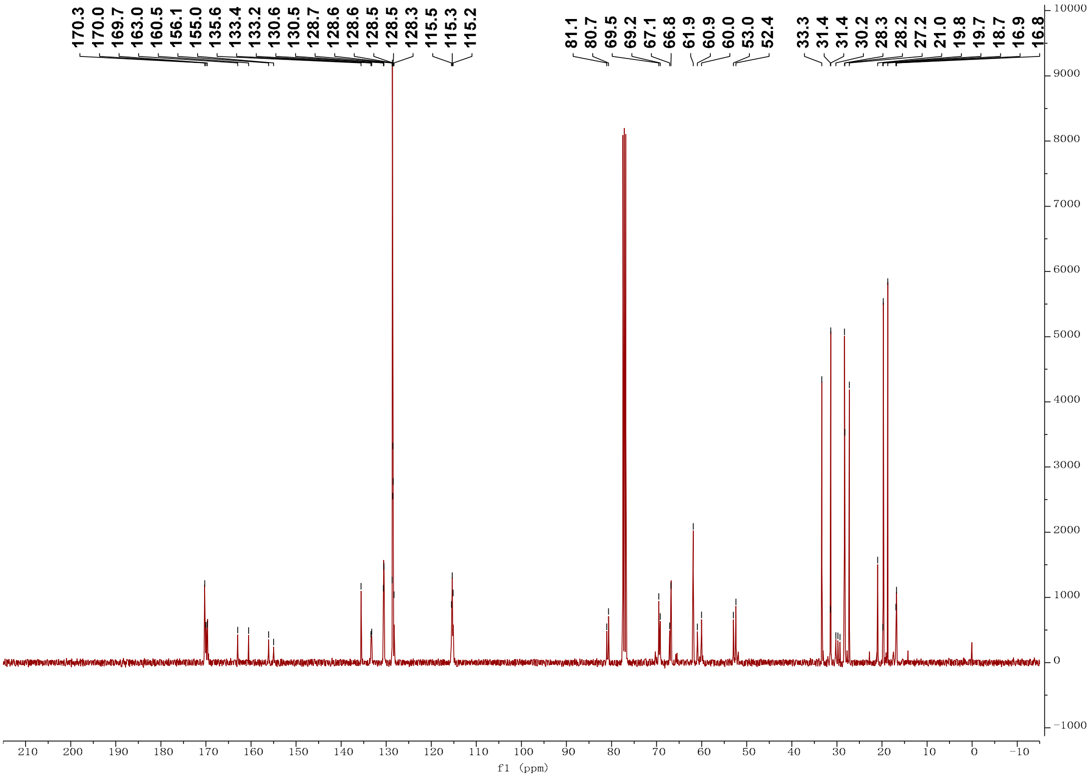

Figure S106. ^{13}C NMR spectrum of compound **8i** in $CDCl_3$ (100 MHz)

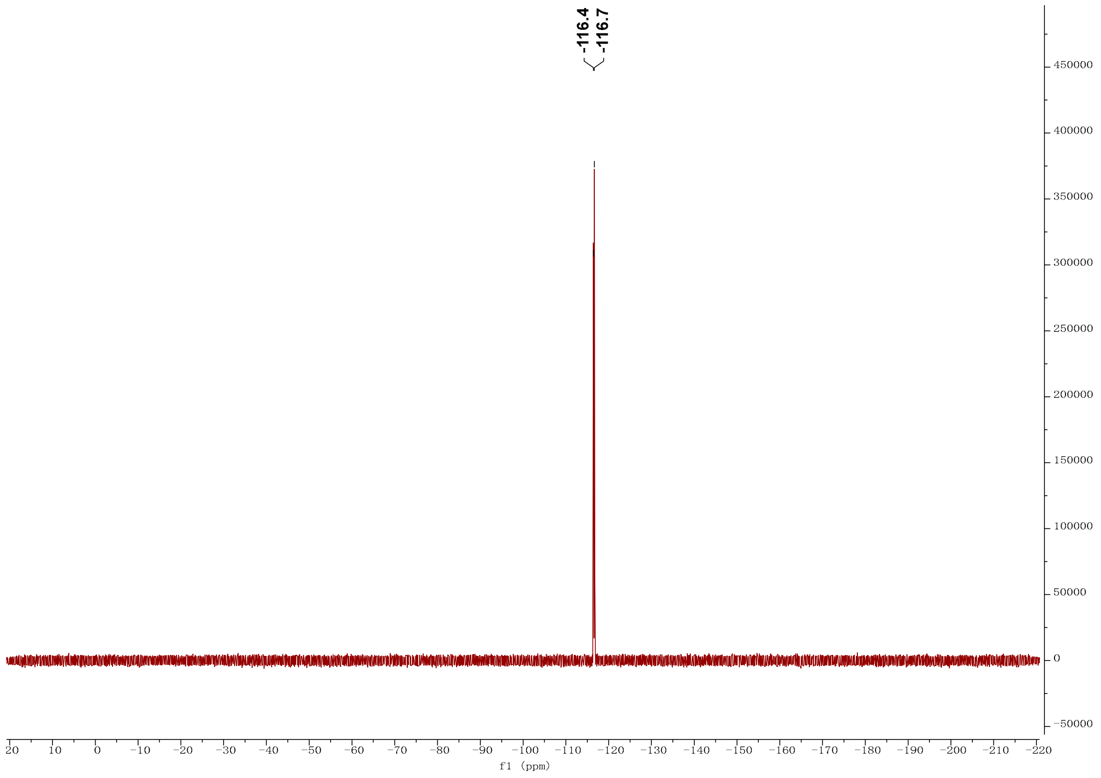

Figure S107. ^{19}F NMR spectrum of compound **8i** in $CDCl_3$ (376 MHz)

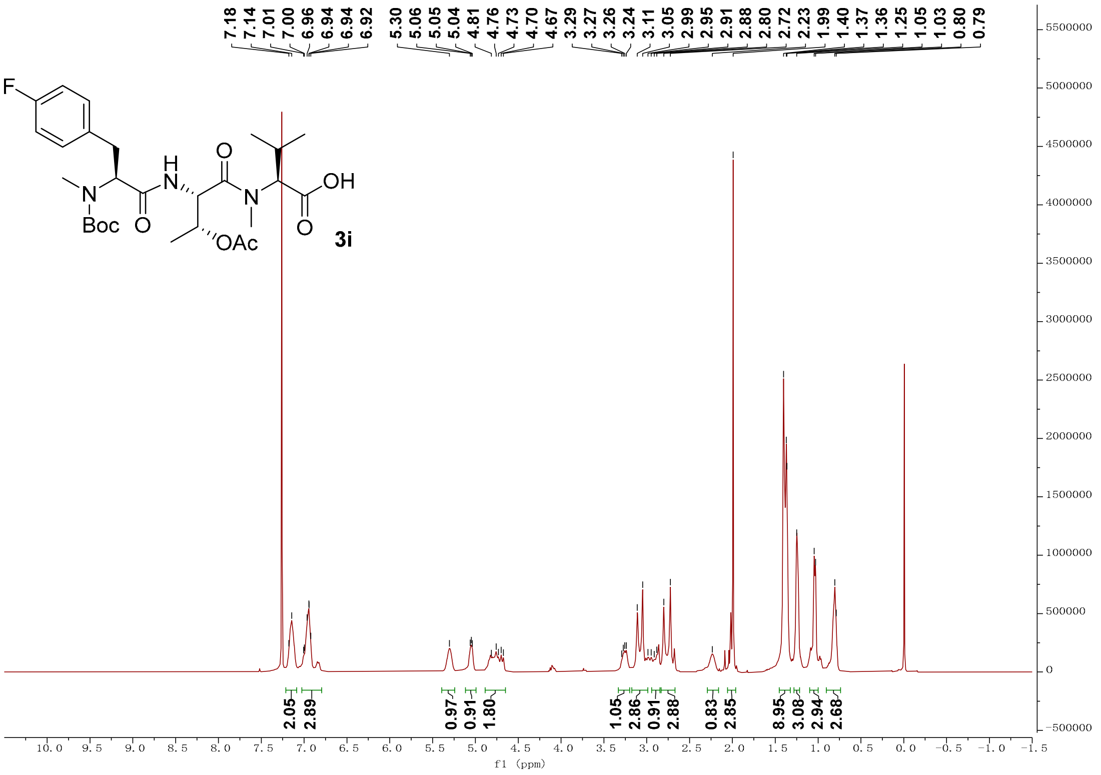

Figure S108. ^{1}H NMR spectrum of compound **3i** in $CDCl_3$ (400 MHz)

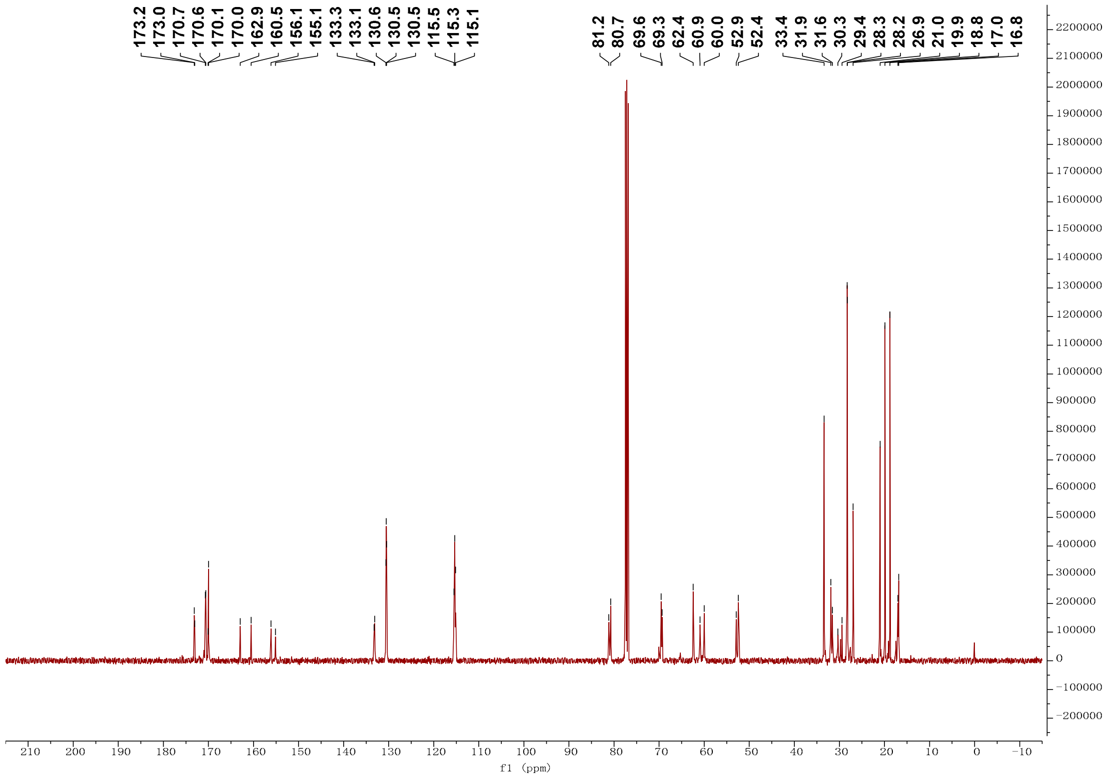

Figure S109. ^{13}C NMR spectrum of compound **3i** in $CDCl_3$ (100 MHz)

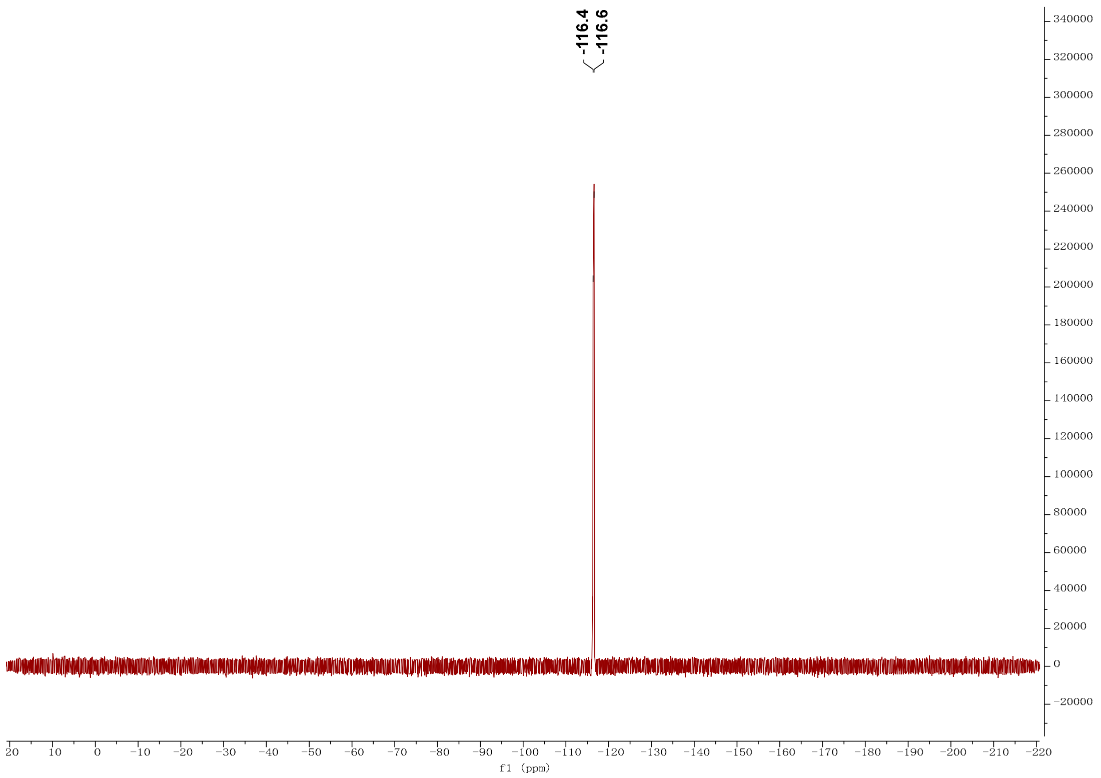

Figure S110. ^{19}F NMR spectrum of compound **3i** in $CDCl_3$ (376 MHz)

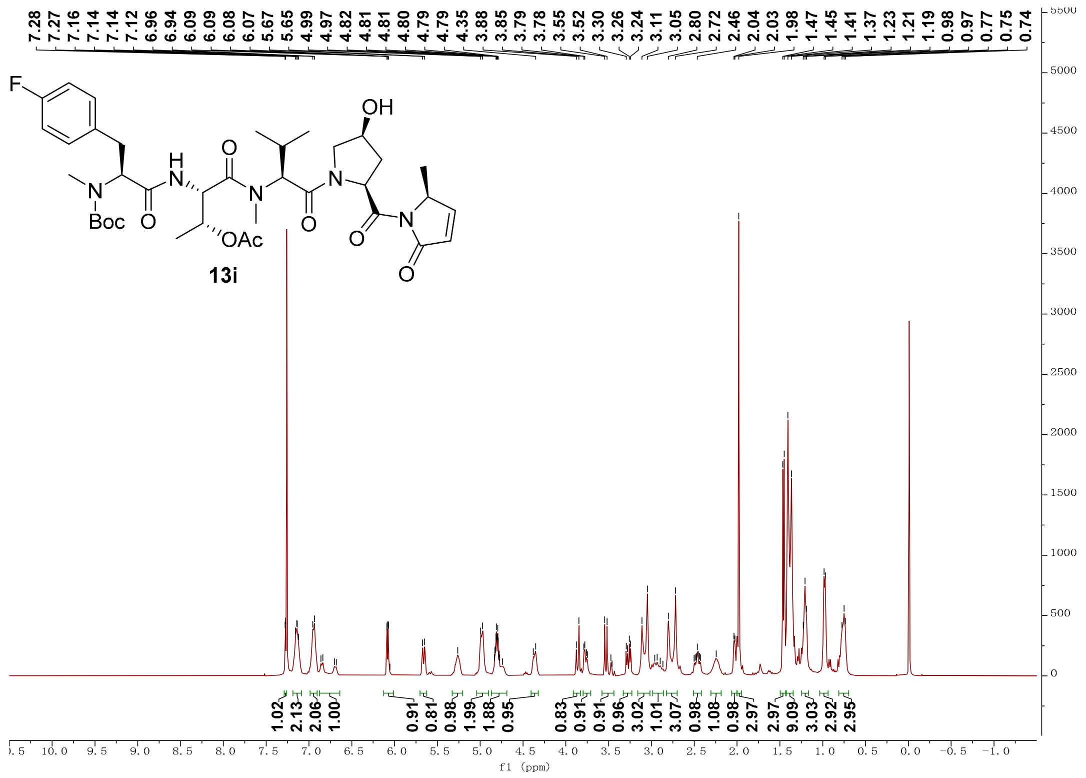

Figure S111. ^{1}H NMR spectrum of compound **13i** in $CDCl_3$ (400 MHz)

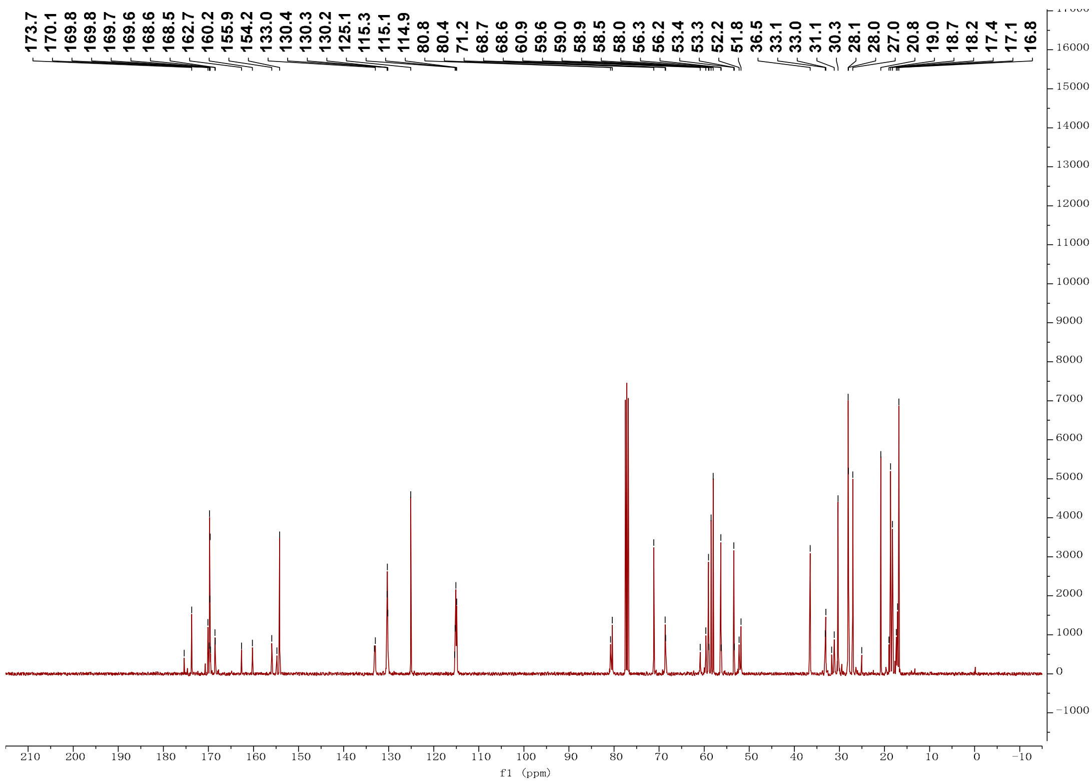

Figure S112. ^{13}C NMR spectrum of compound **13i** in $CDCl_3$ (100 MHz)

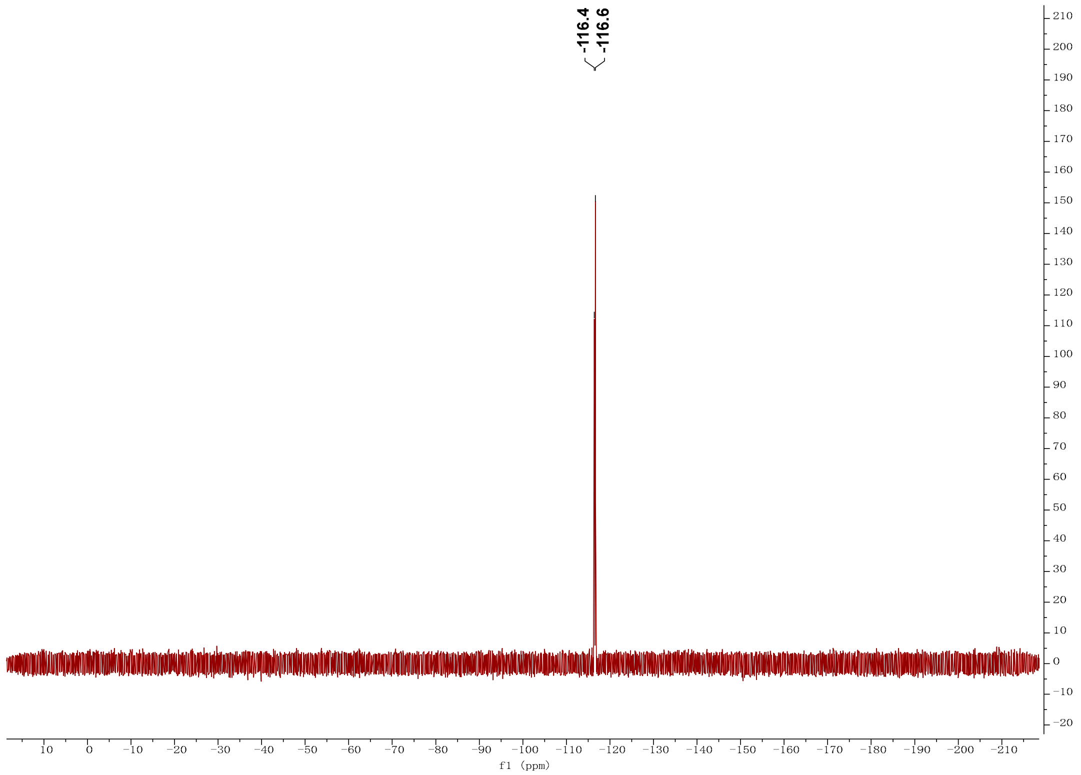

Figure S113. ^{19}F NMR spectrum of compound **13i** in $CDCl_3$ (376 MHz)

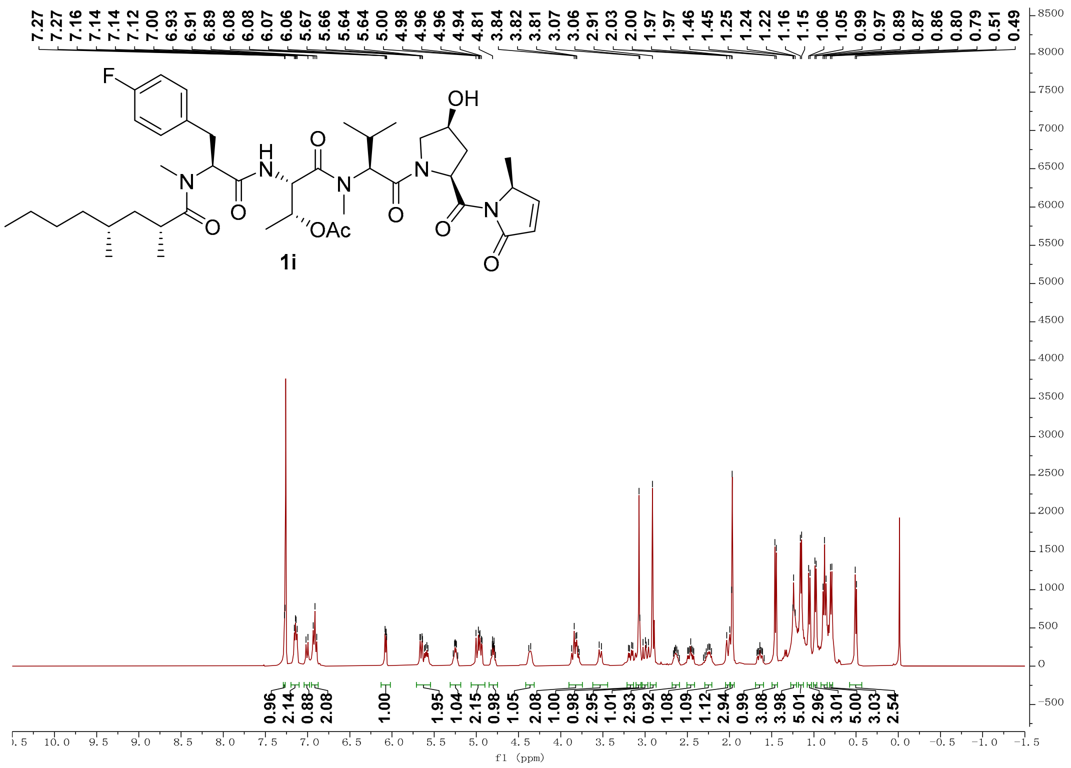

Figure S114. ^{1}H NMR spectrum of compound **1i** in $CDCl_3$ (400 MHz)

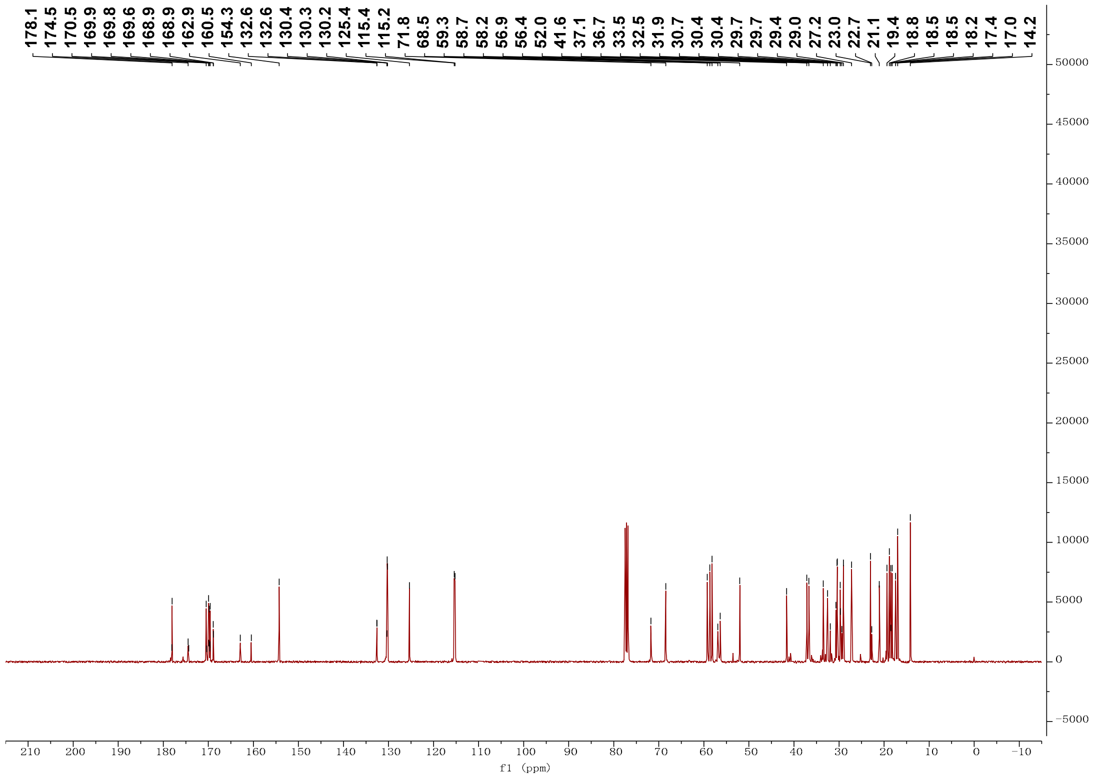

Figure S115. ^{13}C NMR spectrum of compound **1i** in $CDCl_3$ (100 MHz)

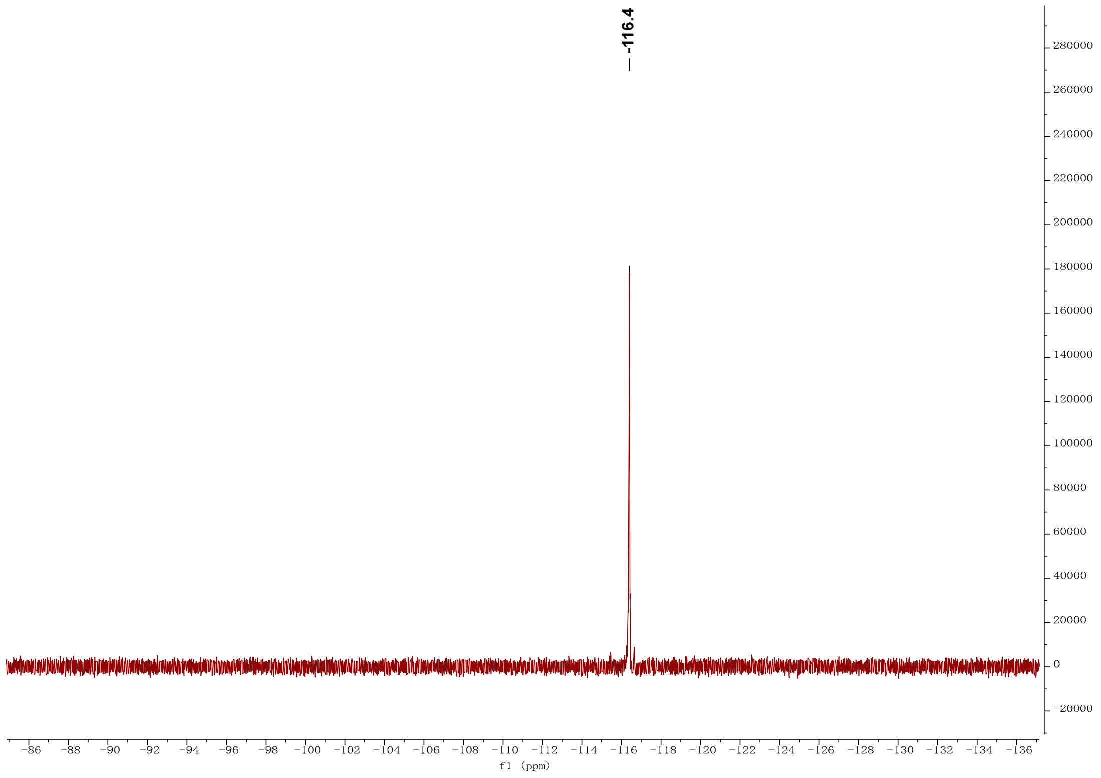

Figure S116. ^{19}F NMR spectrum of compound **1i** in $CDCl_3$ (376 MHz)

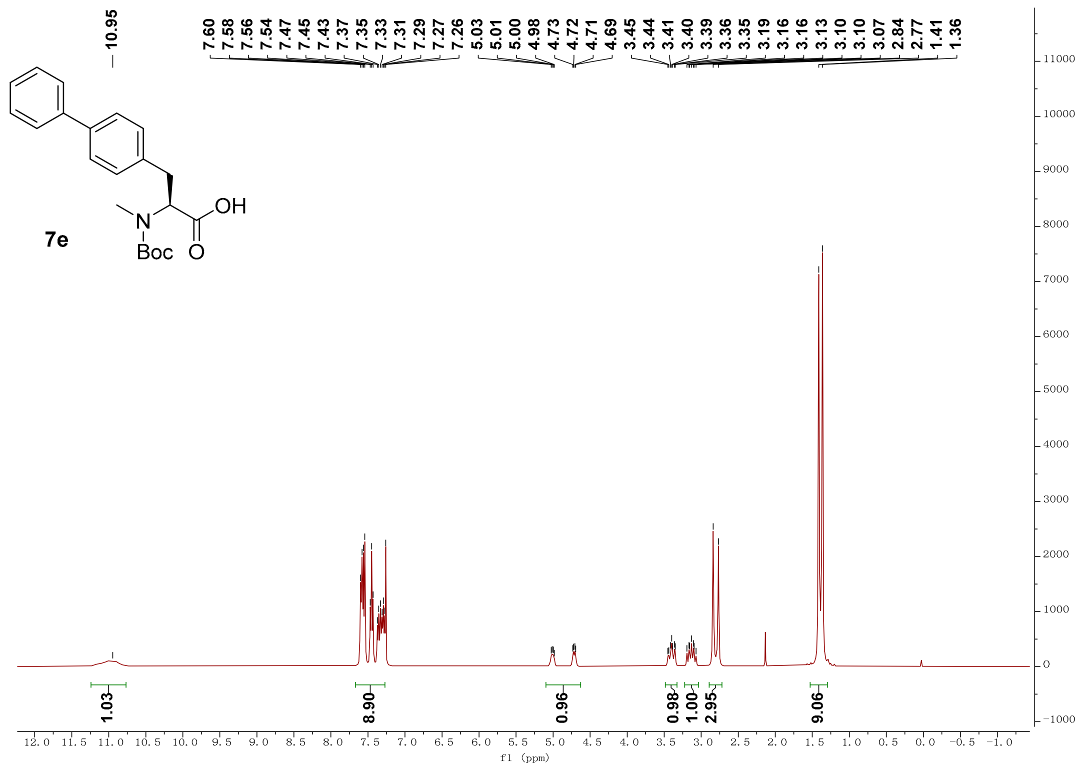

Figure S117. ^{1}H NMR spectrum of compound **7e** in $CDCl_3$ (400 MHz)

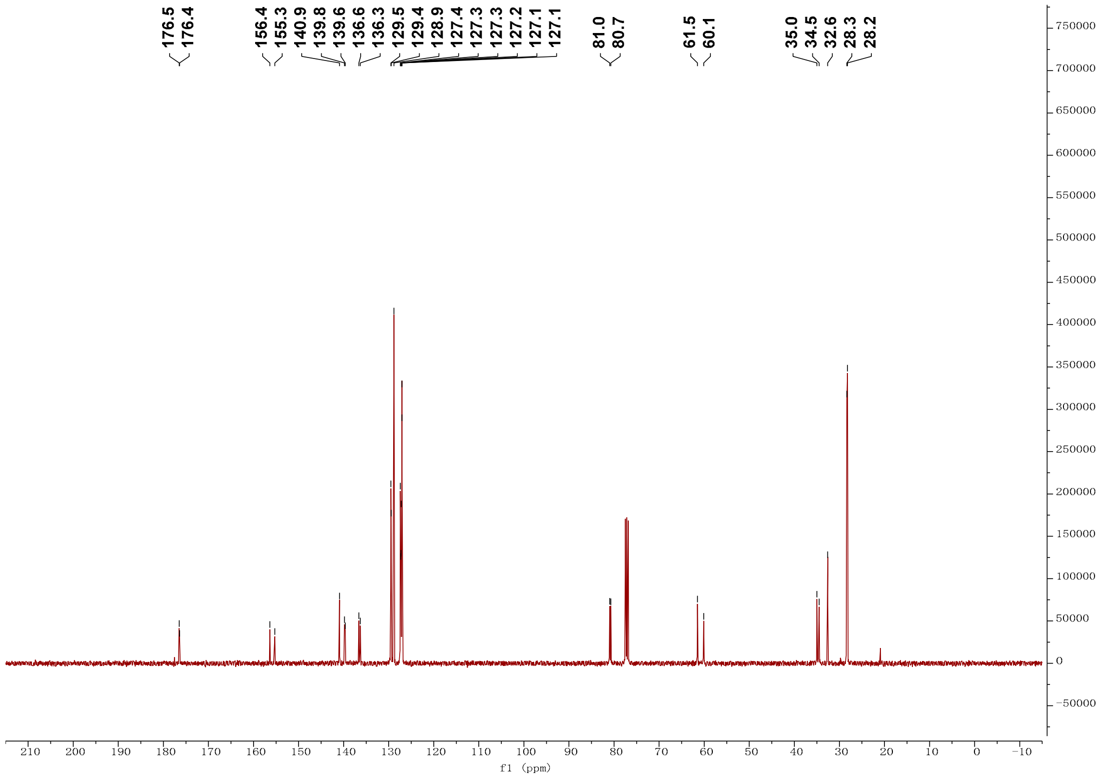

Figure S118. ^{13}C NMR spectrum of compound **7e** in $CDCl_3$ (100 MHz)

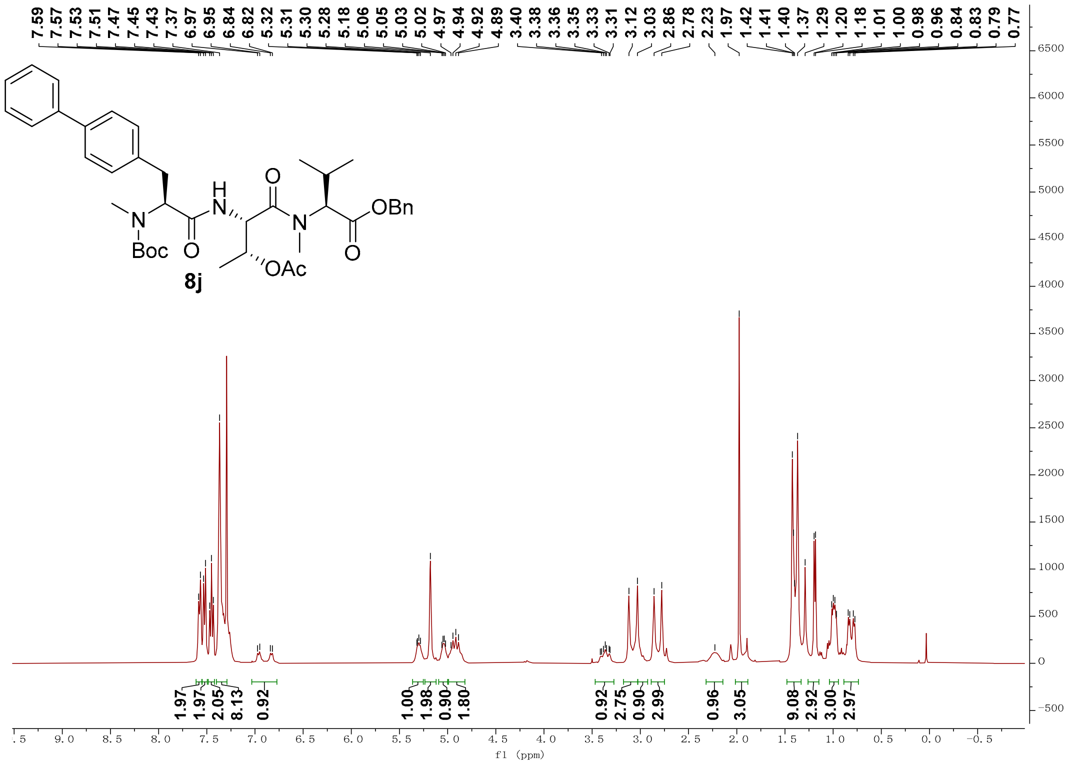

Figure S119. ^{1}H NMR spectrum of compound **8j** in $CDCl_3$ (400 MHz)

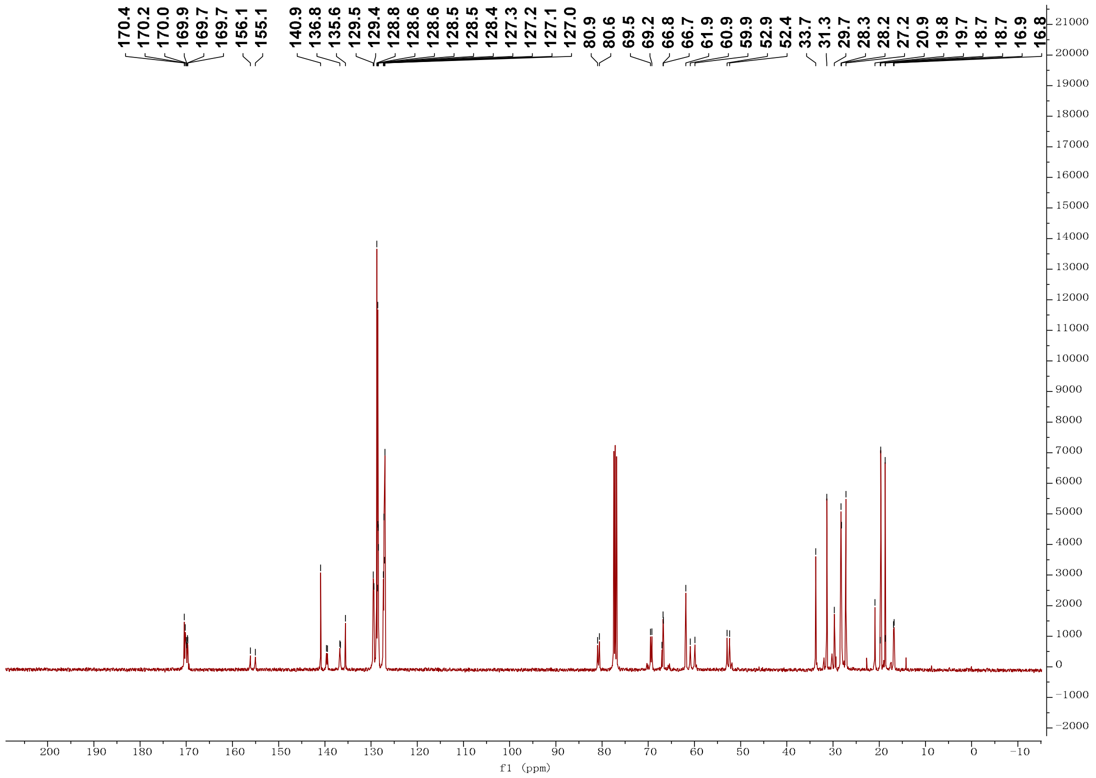

Figure S120. ^{13}C NMR spectrum of compound **8j** in $CDCl_3$ (100 MHz)

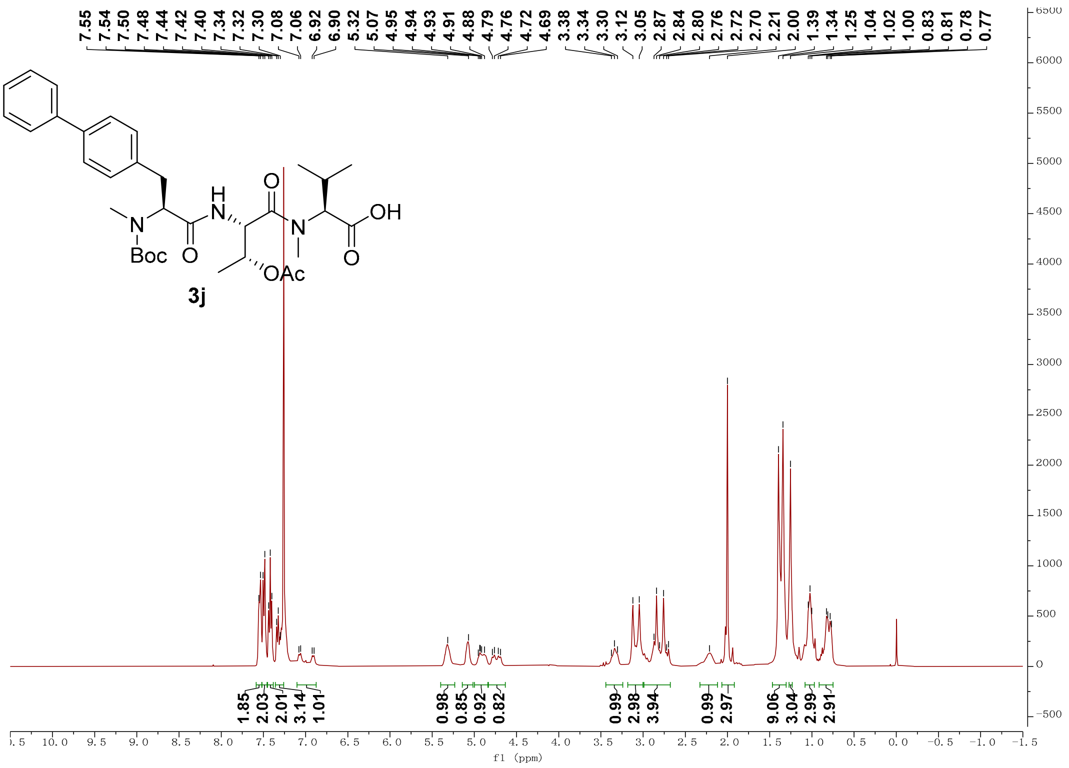

Figure S121. ^{1}H NMR spectrum of compound **3j** in $CDCl_3$ (400 MHz)

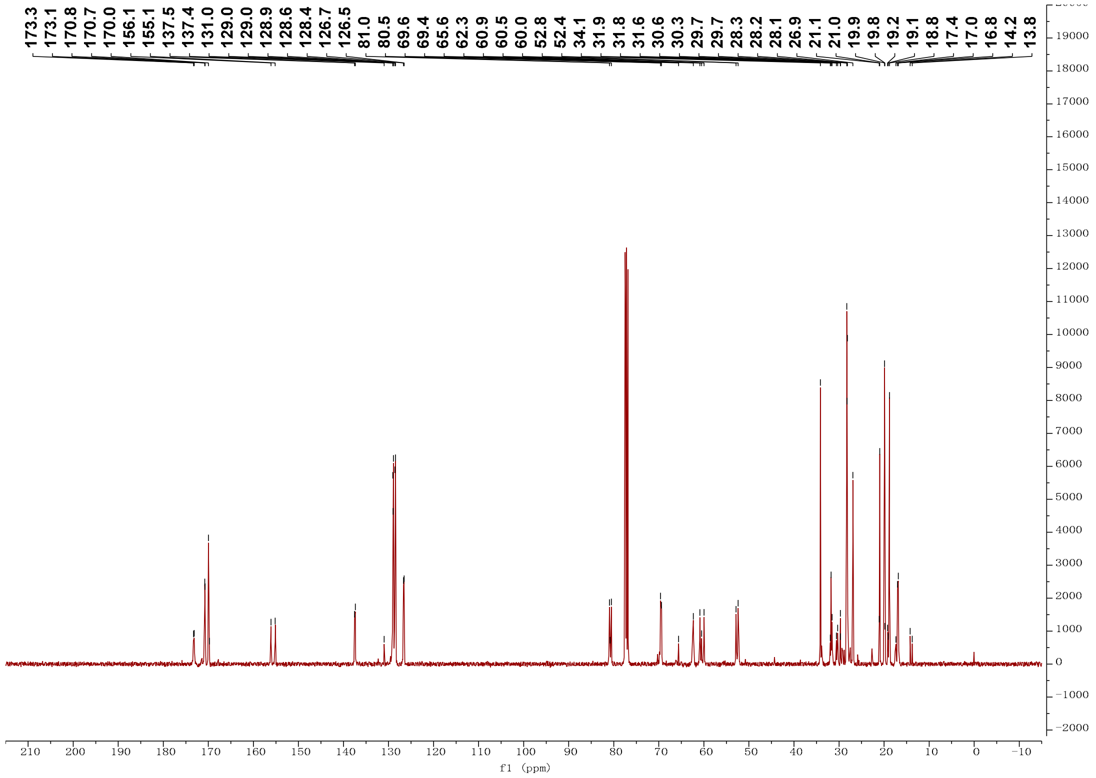

Figure S122. ^{13}C NMR spectrum of compound **3j** in $CDCl_3$ (100 MHz)

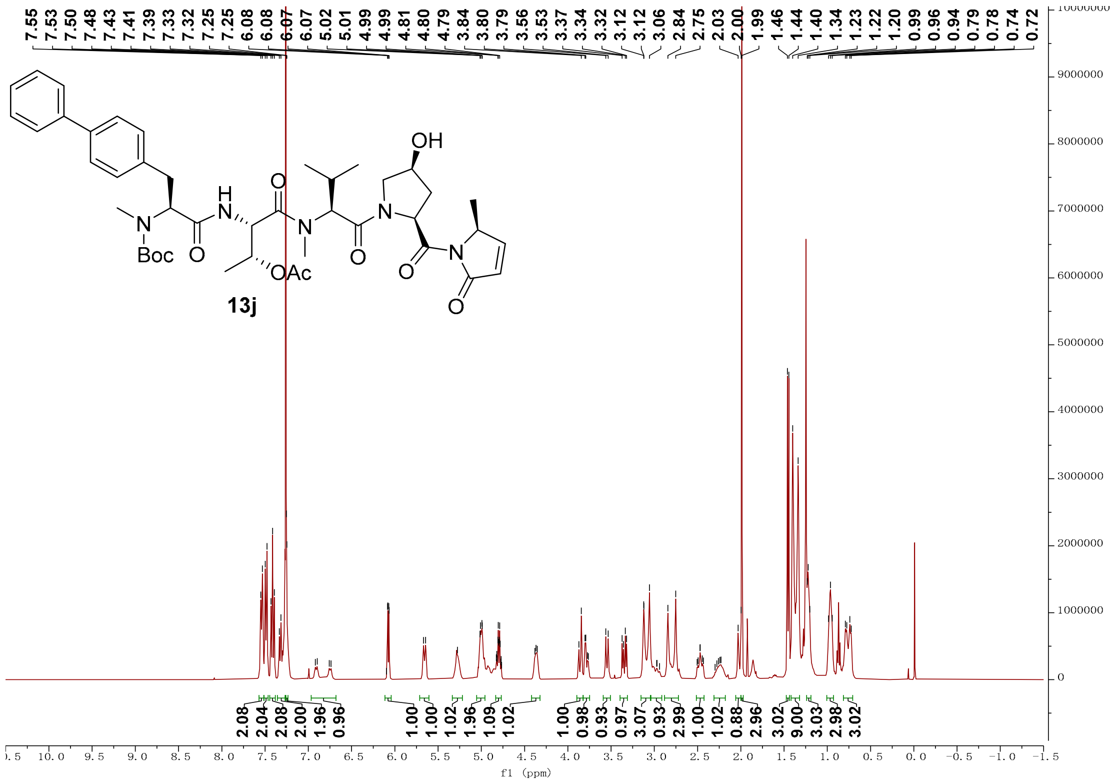

Figure S123. ¹H NMR spectrum of compound **13j** in $CDCl_3$ (400 MHz)

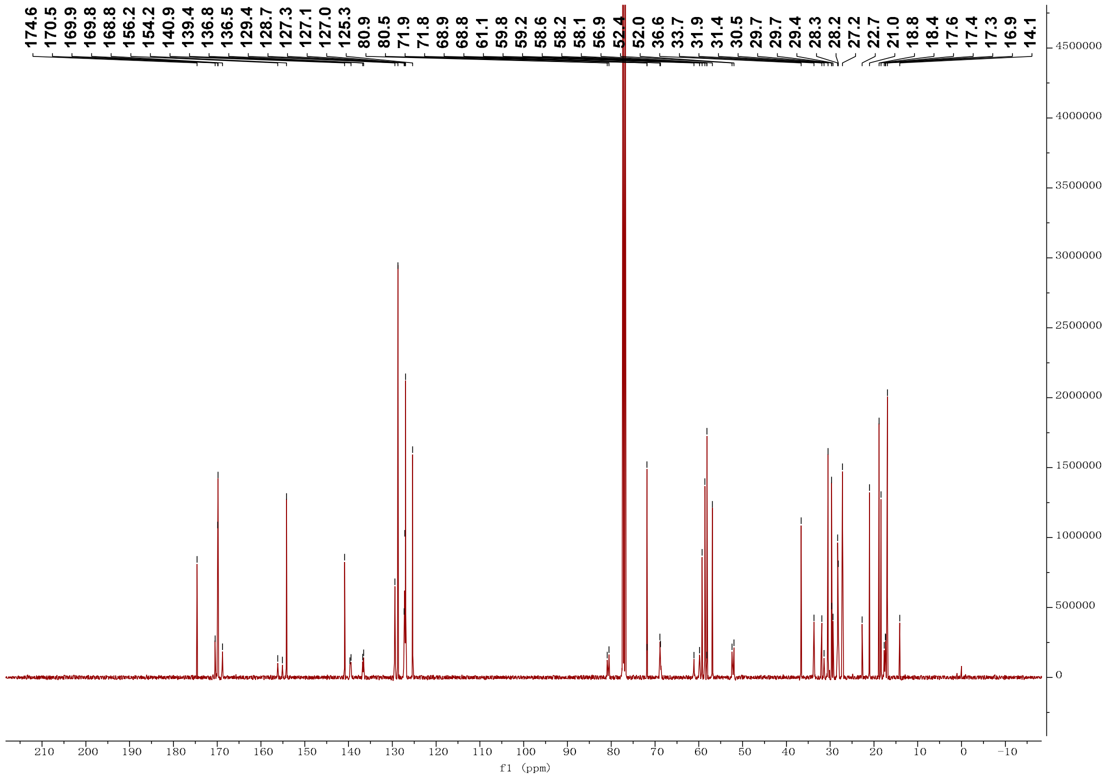

Figure S124. ¹³C NMR spectrum of compound **13j** in $CDCl_3$ (100 MHz)

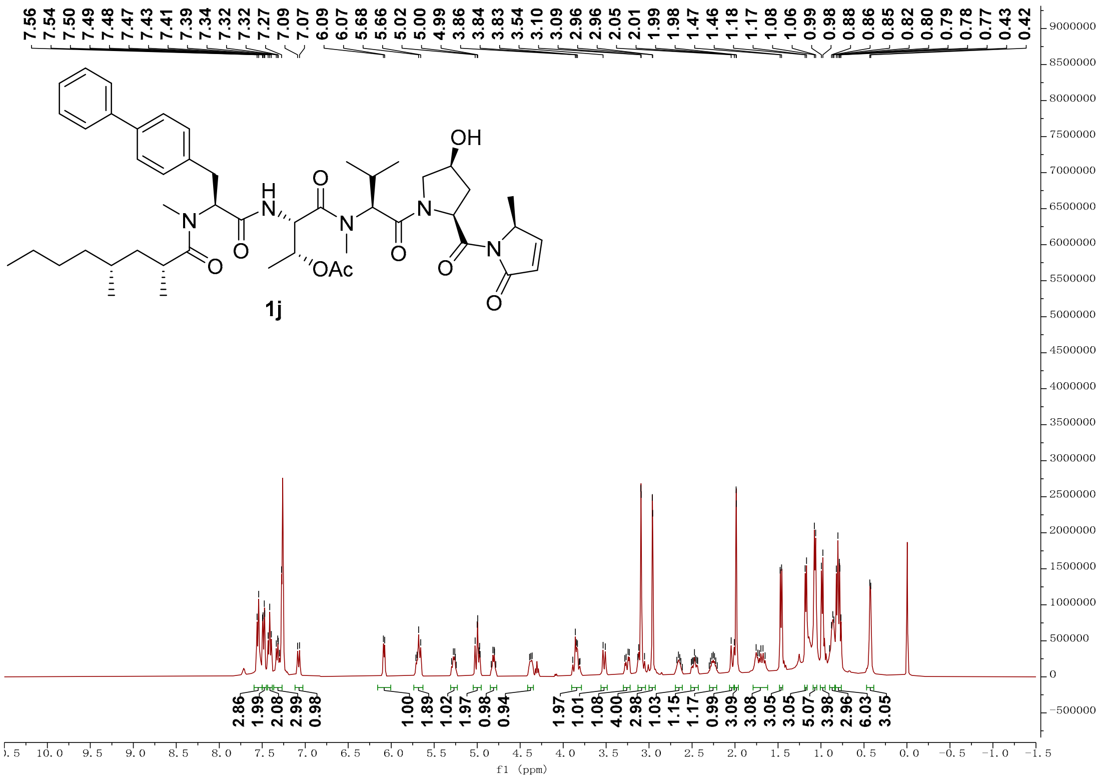

Figure S125. ^{1}H NMR spectrum of compound **1j** in $CDCl_3$ (400 MHz)

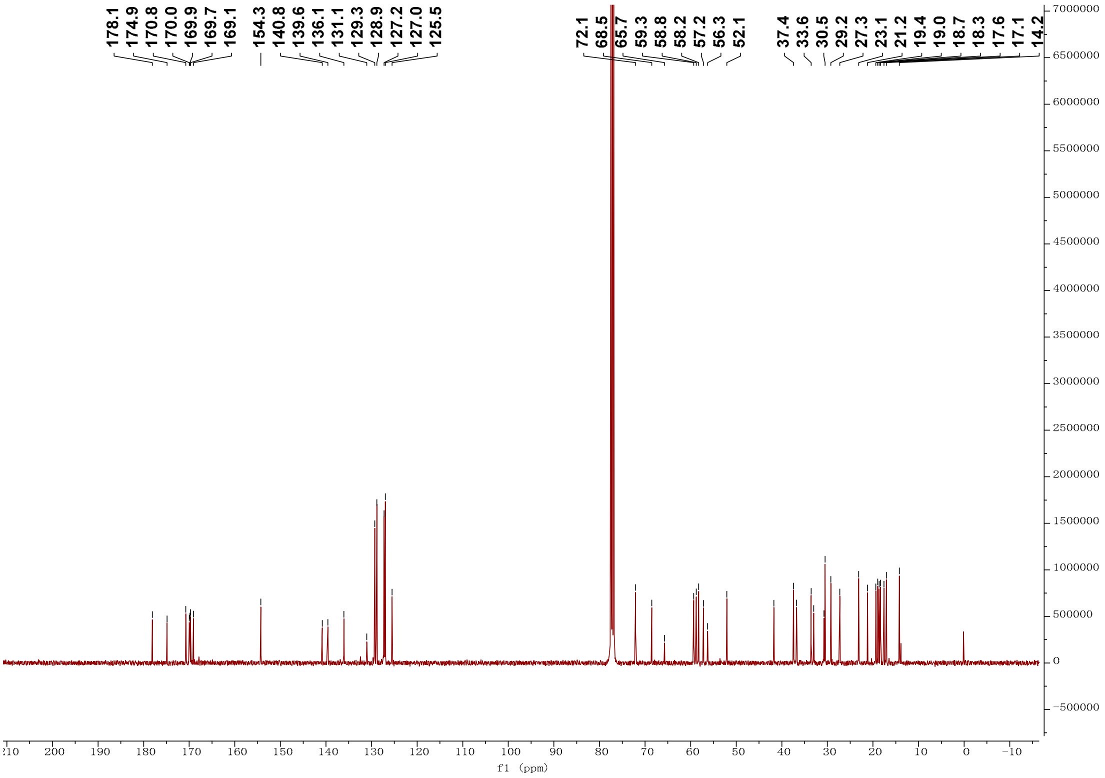

Figure S126. ^{13}C NMR spectrum of compound **1j** in $CDCl_3$ (100 MHz)

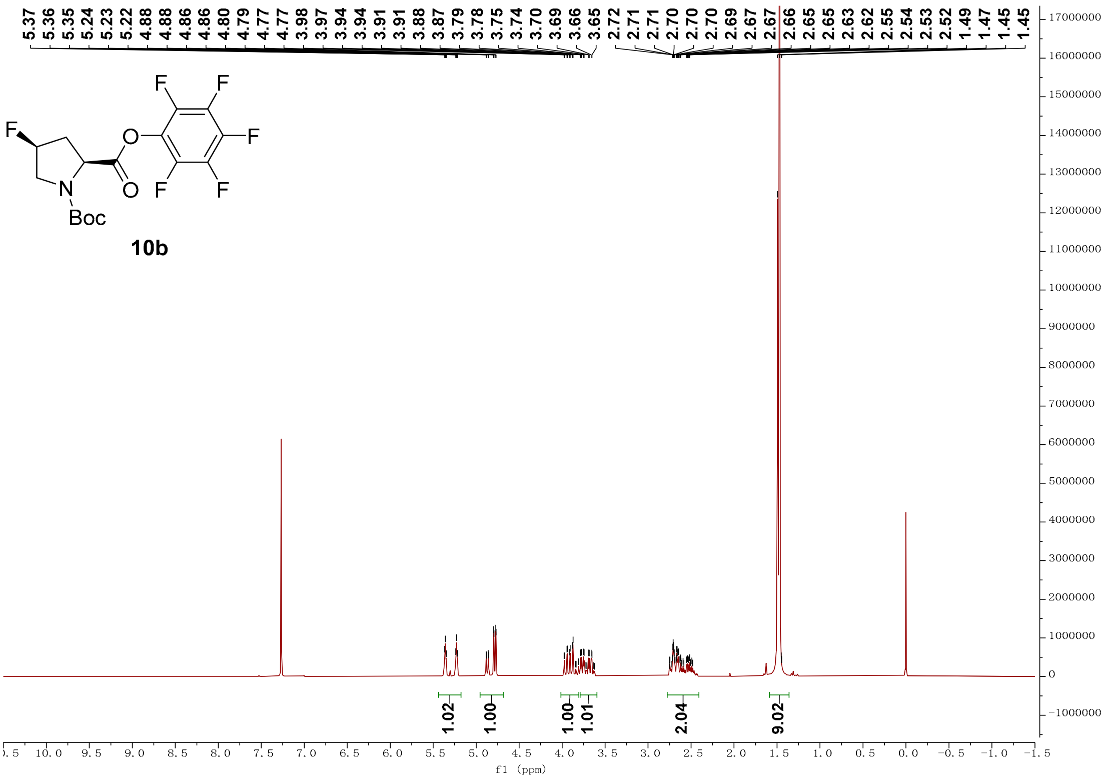

Figure S127. ^{1}H NMR spectrum of compound **10b** in $CDCl_3$ (400 MHz)

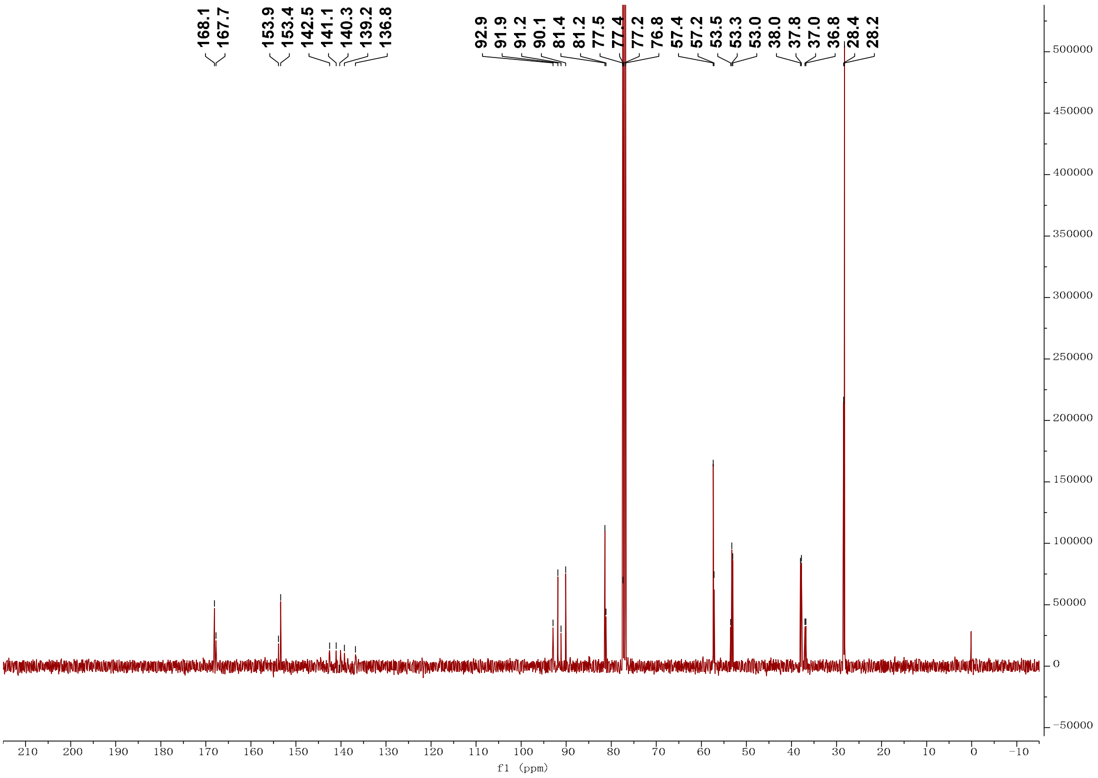

Figure S128. ^{13}C NMR spectrum of compound **10b** in $CDCl_3$ (100 MHz)

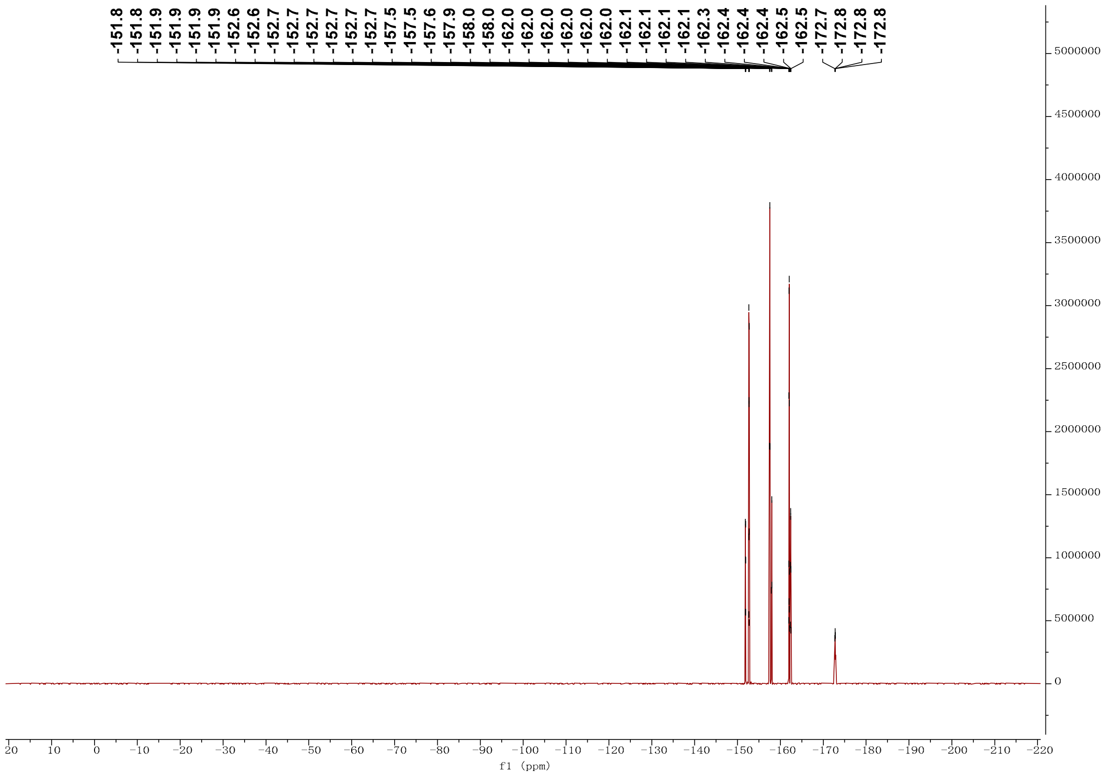

Figure S129. ^{19}F NMR spectrum of compound **10b** in $CDCl_3$ (376 MHz)

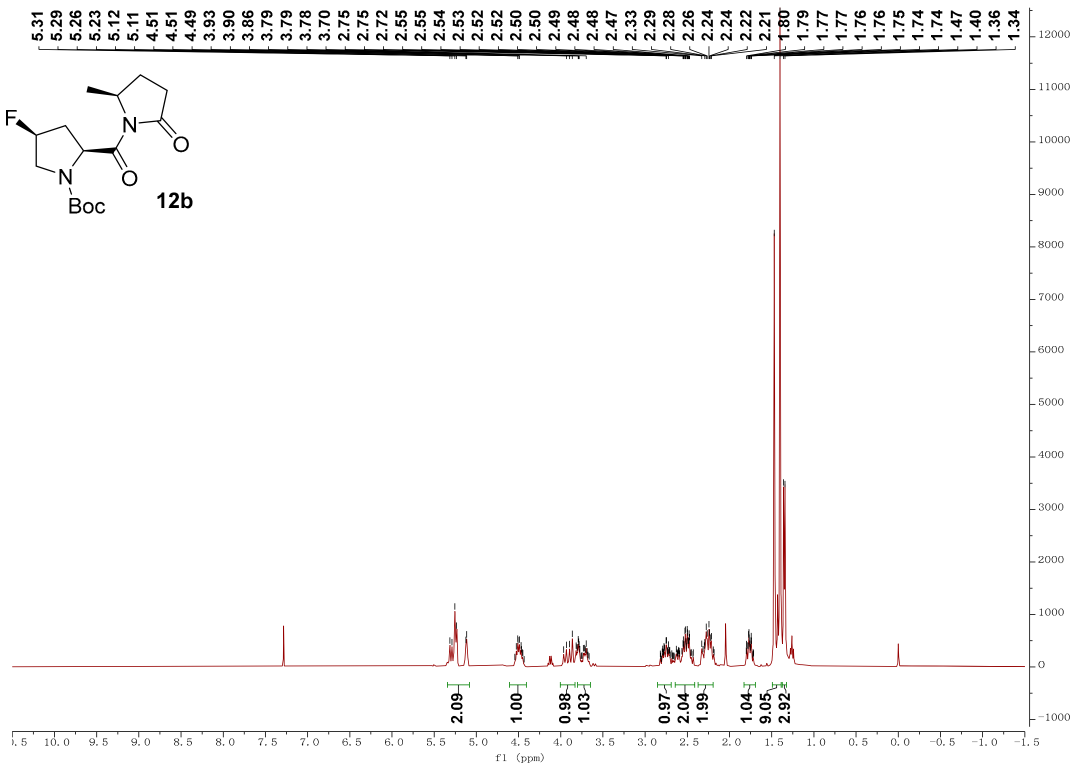

Figure S130. ^{1}H NMR spectrum of compound **12b** in $CDCl_3$ (400 MHz)

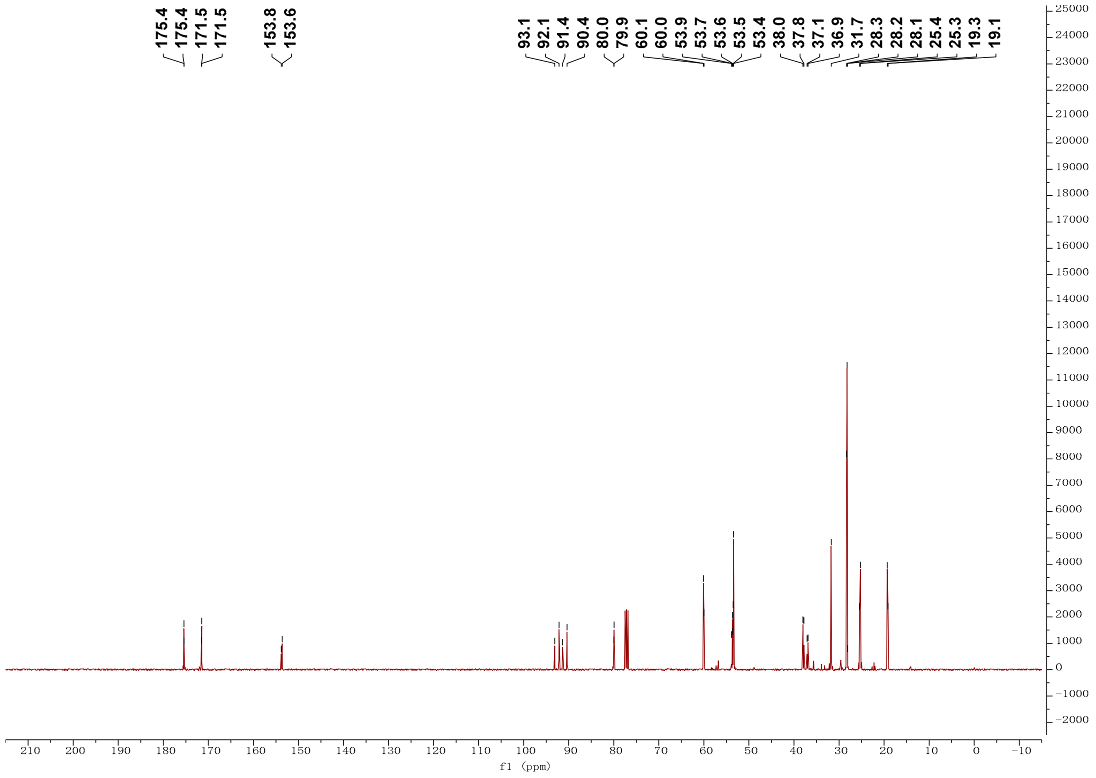

Figure S131. ^{13}C NMR spectrum of compound **12b** in $CDCl_3$ (100 MHz)

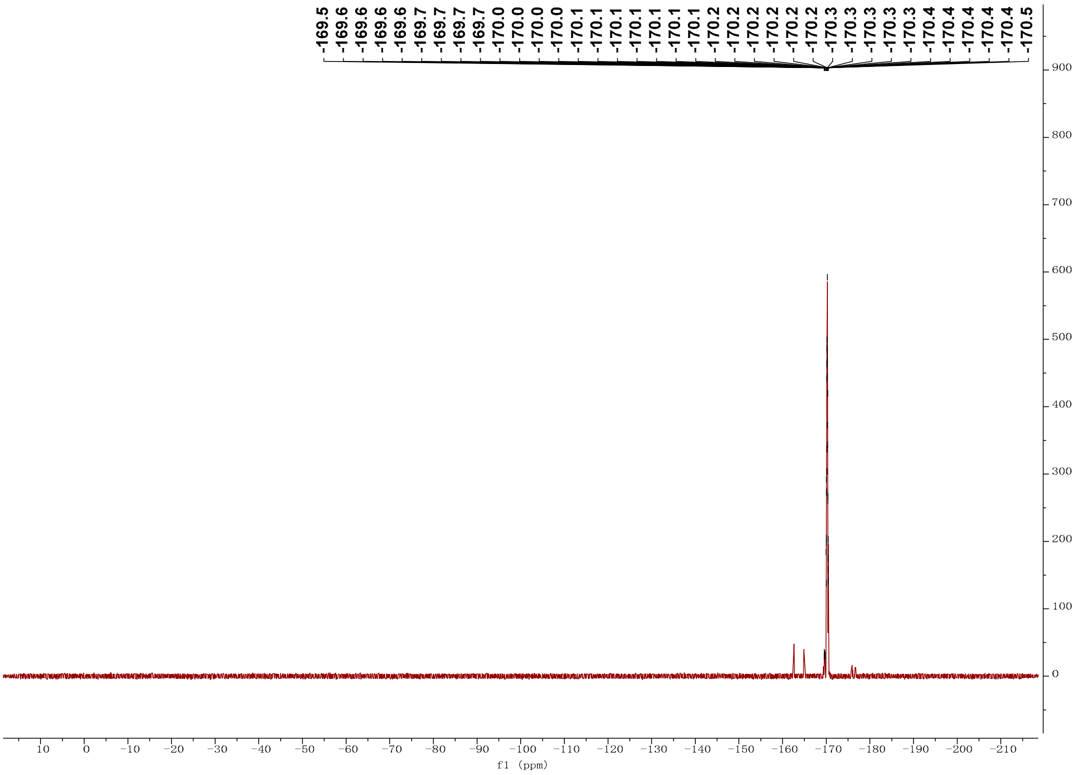

Figure S132. ^{19}F NMR spectrum of compound **12b** in $CDCl_3$ (376 MHz)

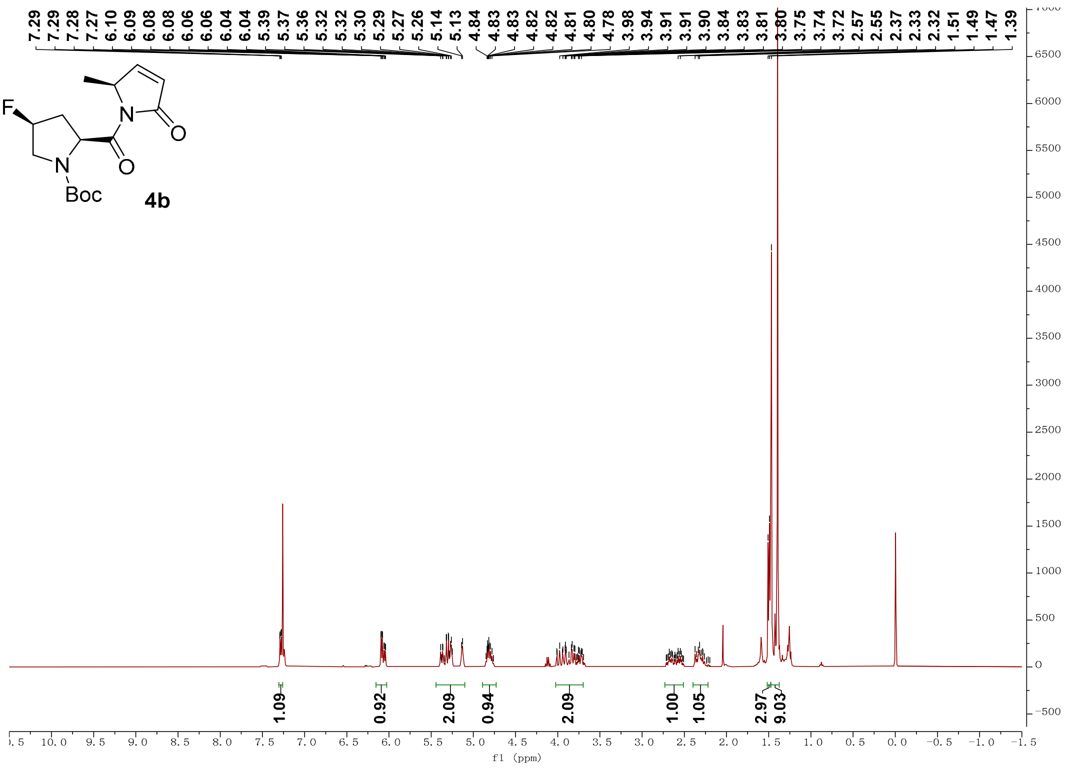

Figure S133. ^{1}H NMR spectrum of compound **4b** in $CDCl_3$ (400 MHz)

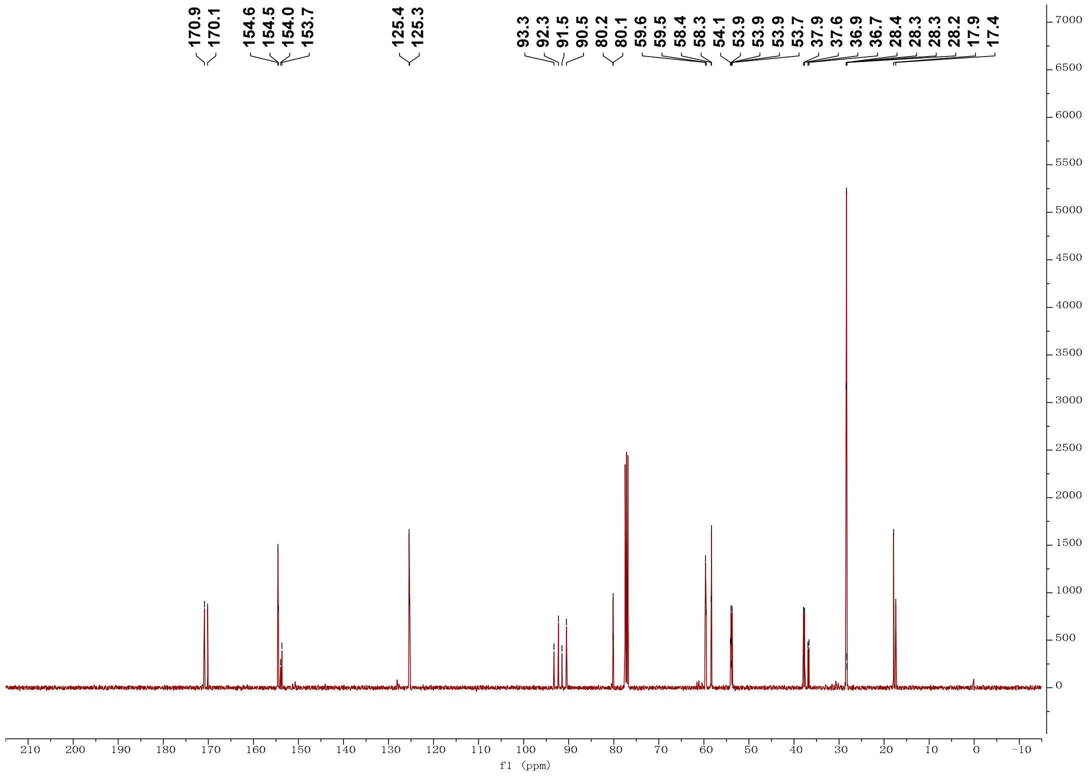

Figure S134. ^{13}C NMR spectrum of compound **4b** in $CDCl_3$ (100 MHz)

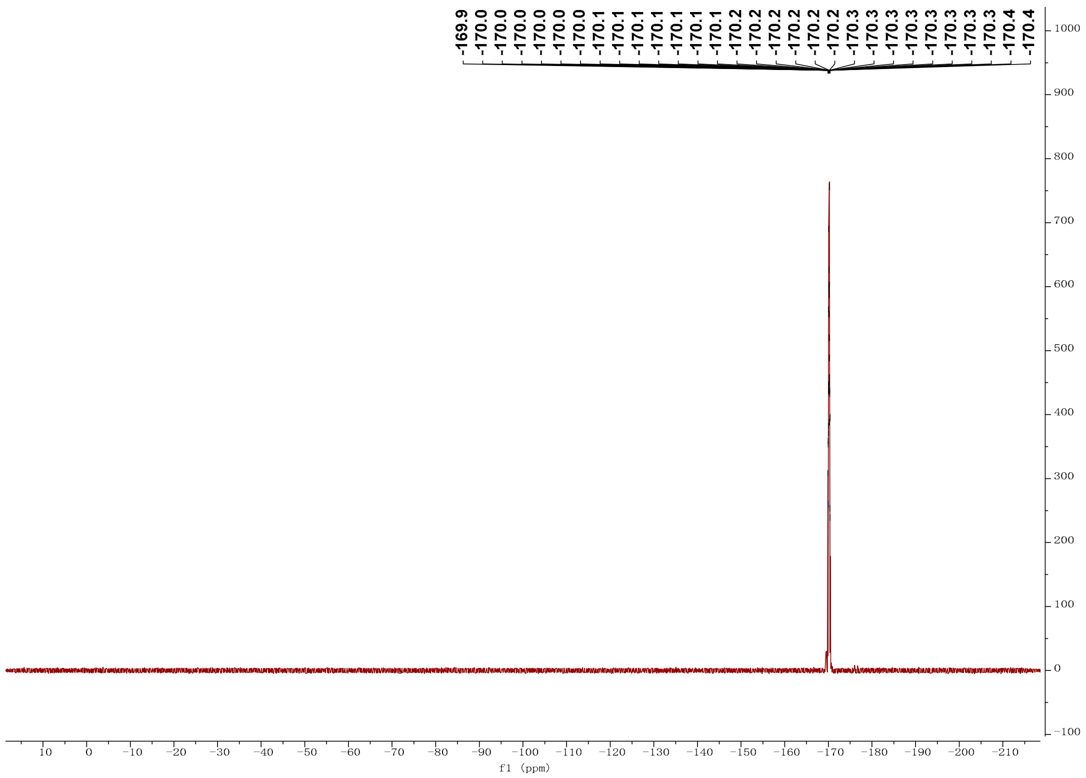

Figure S135. ^{19}F NMR spectrum of compound **4b** in $CDCl_3$ (376 MHz)

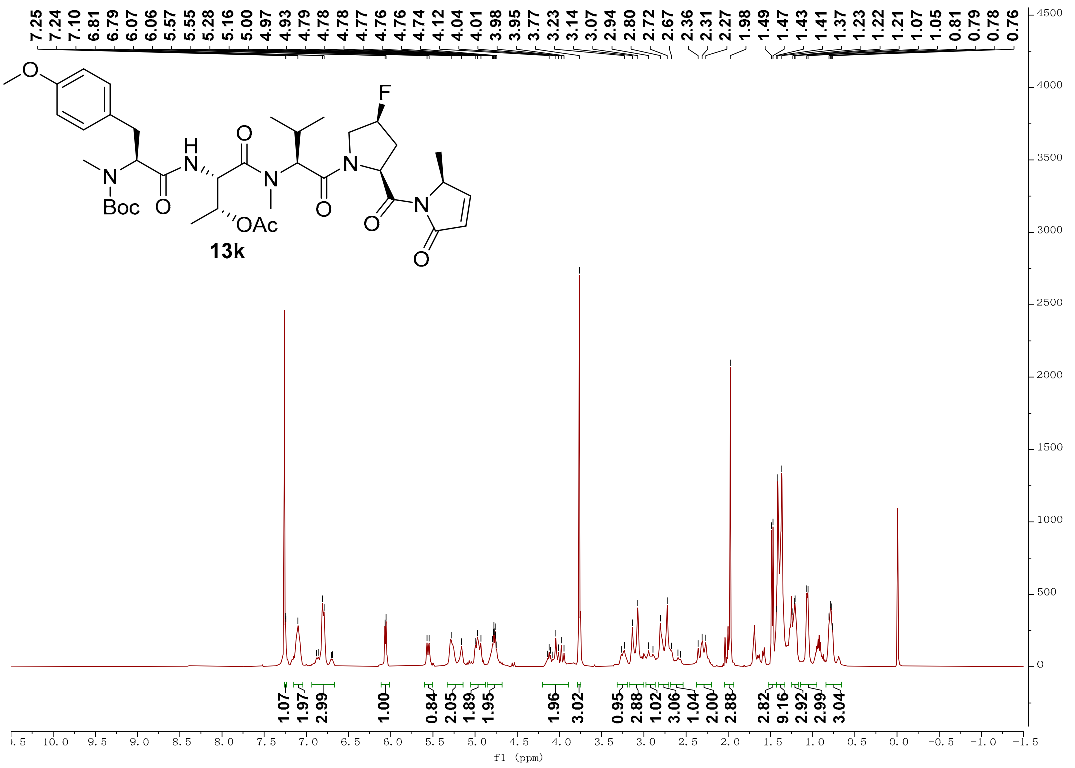

Figure S136. ^{1}H NMR spectrum of compound **13k** in $CDCl_3$ (400 MHz)

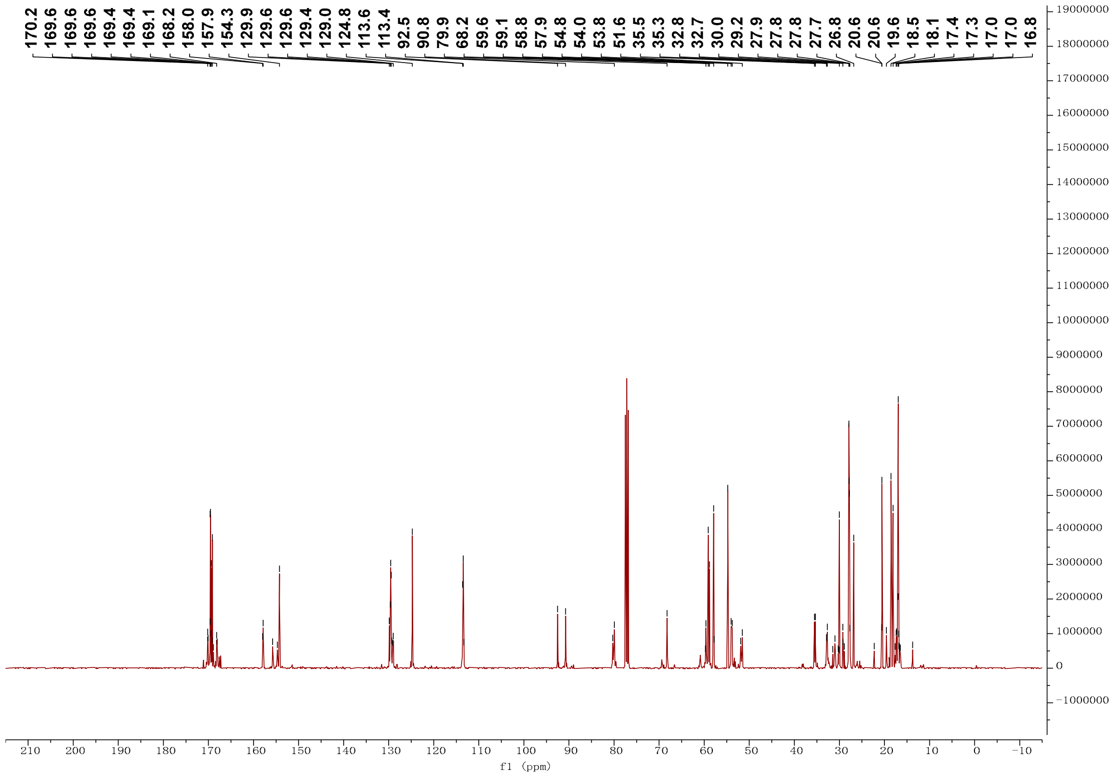

Figure S137. ^{13}C NMR spectrum of compound **13k** in $CDCl_3$ (100 MHz)

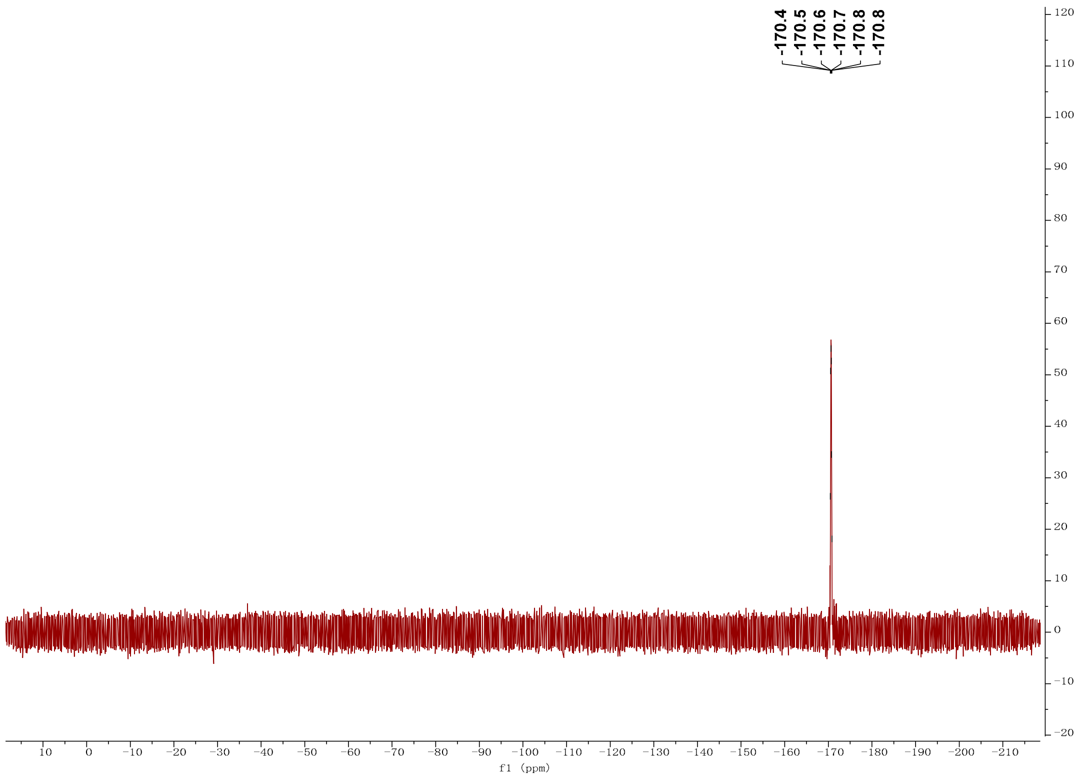

Figure S138. ^{19}F NMR spectrum of compound **13k** in $CDCl_3$ (376 MHz)

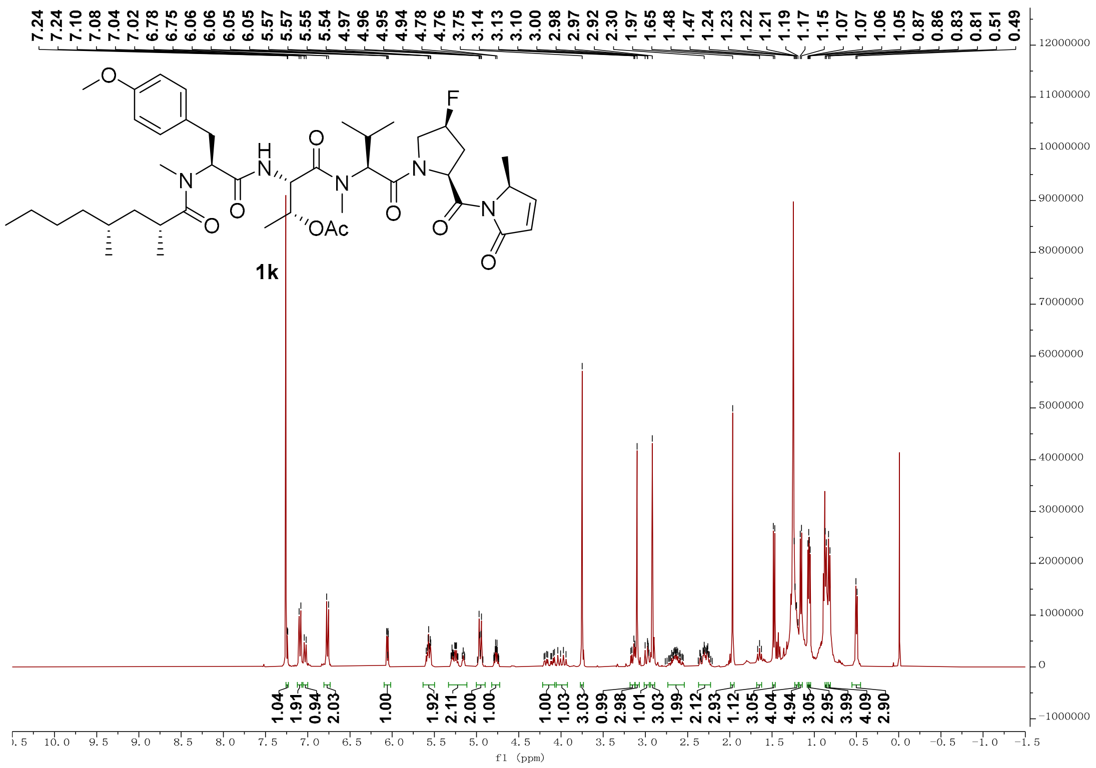

1k

Figure S139. ^{1}H NMR spectrum of compound **1k** in $CDCl_3$ (400 MHz)

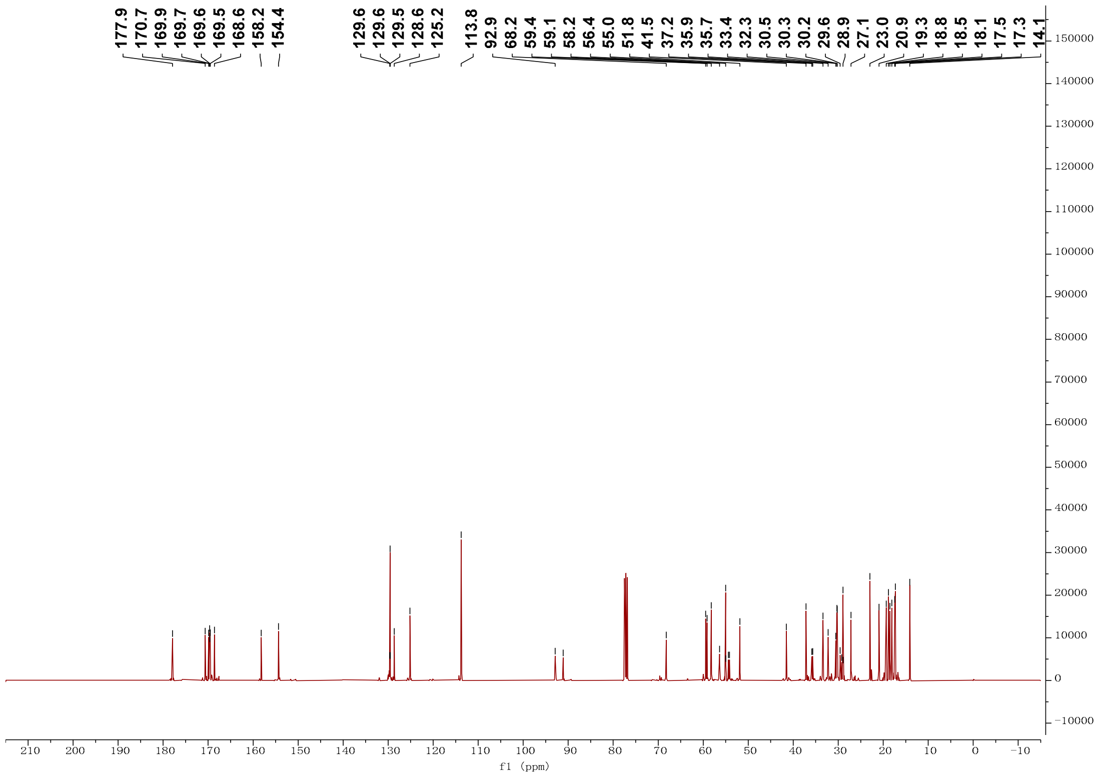

Figure S140. ^{13}C NMR spectrum of compound **1k** in $CDCl_3$ (100 MHz)

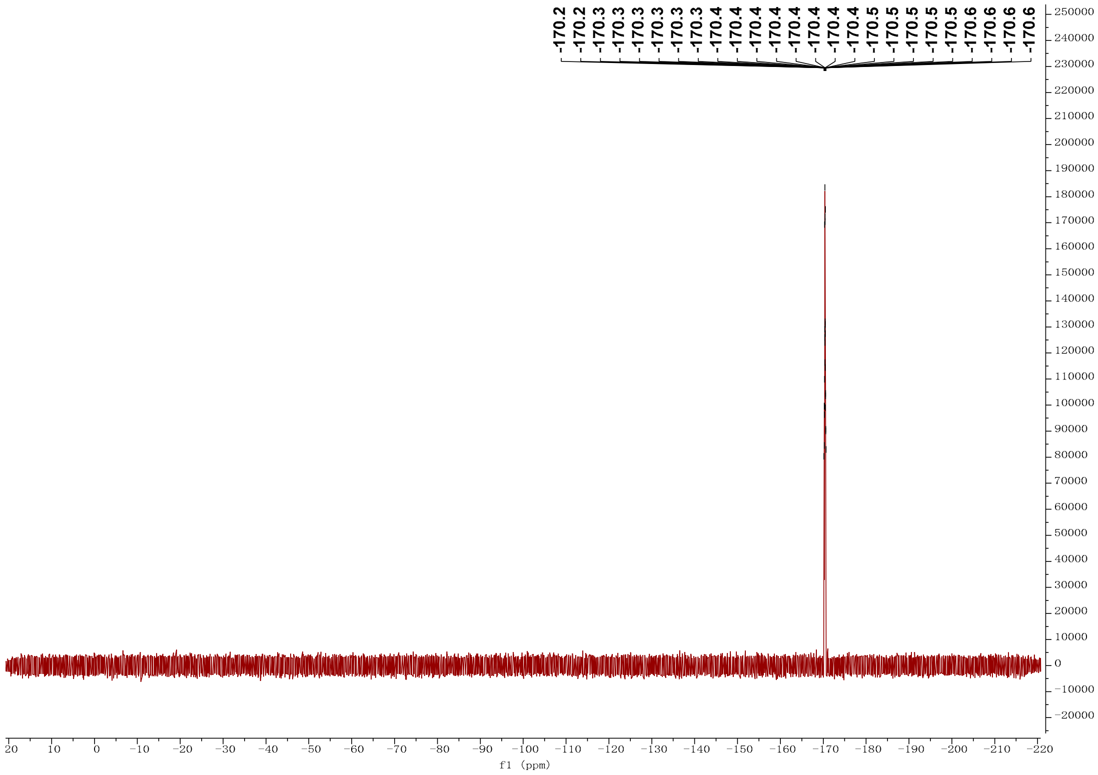

Figure S141. ^{19}F NMR spectrum of compound **1k** in $CDCl_3$ (376 MHz)

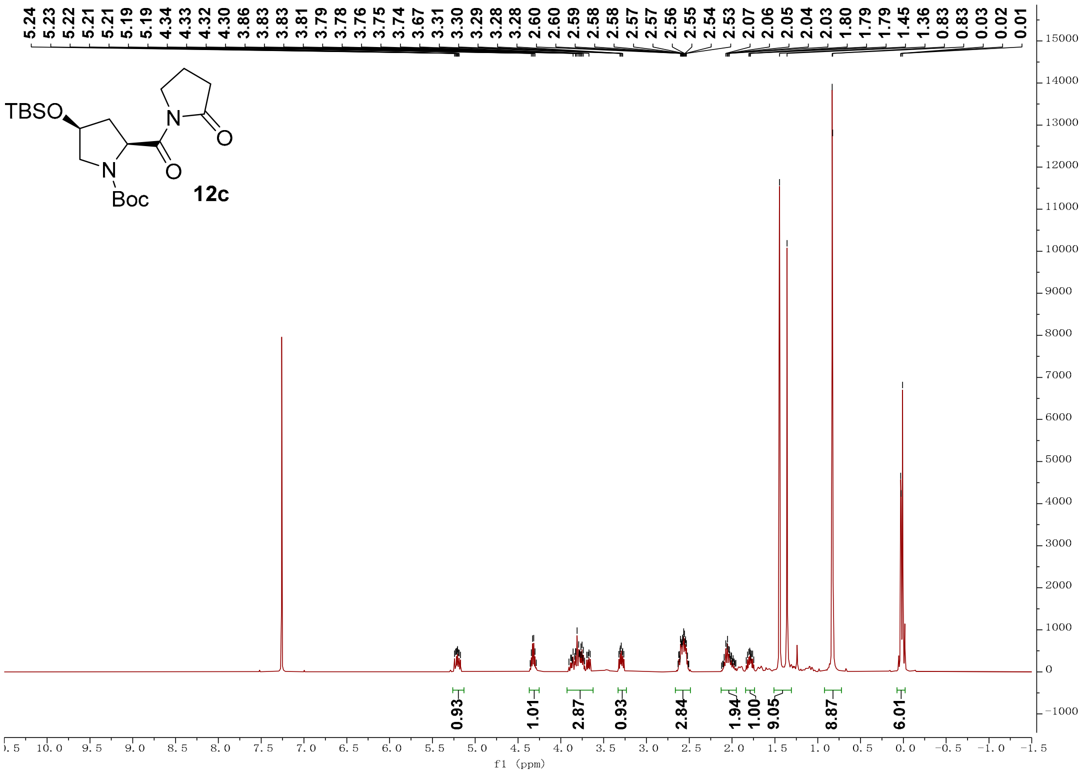

Figure S142. ^{1}H NMR spectrum of compound **12c** in $CDCl_3$ (400 MHz)

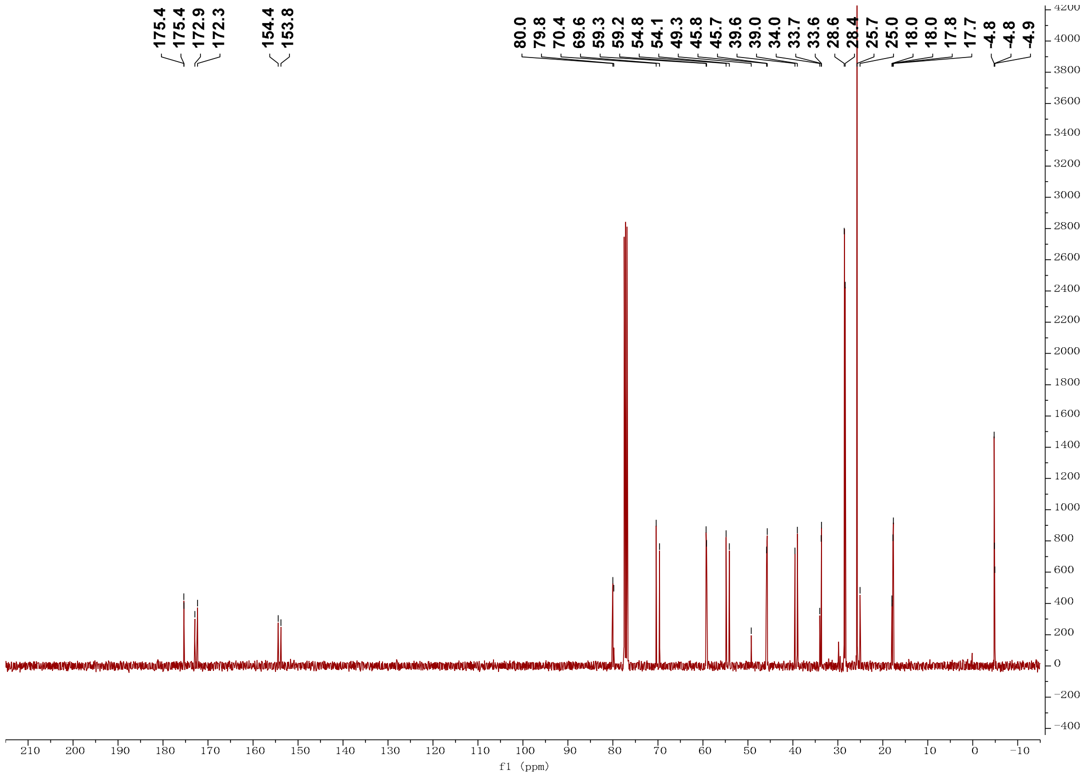

Figure S143. ^{13}C NMR spectrum of compound **12c** in $CDCl_3$ (100 MHz)

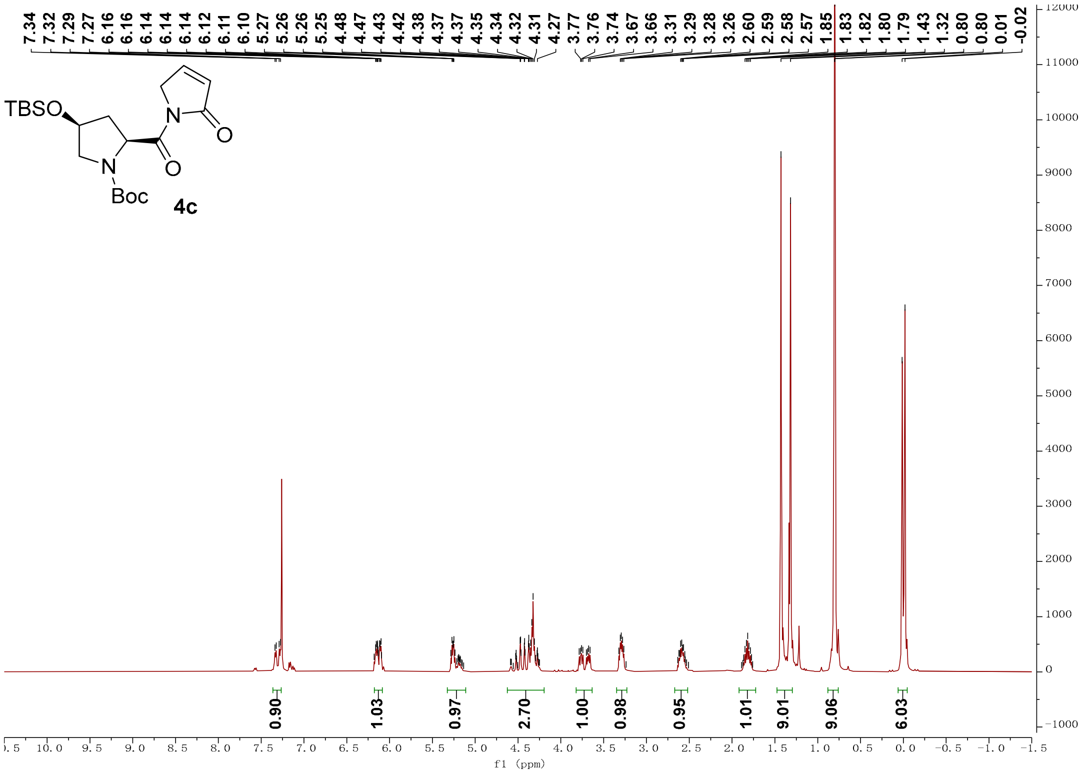

Figure S144. ^{1}H NMR spectrum of compound **4c** in $CDCl_3$ (400 MHz)

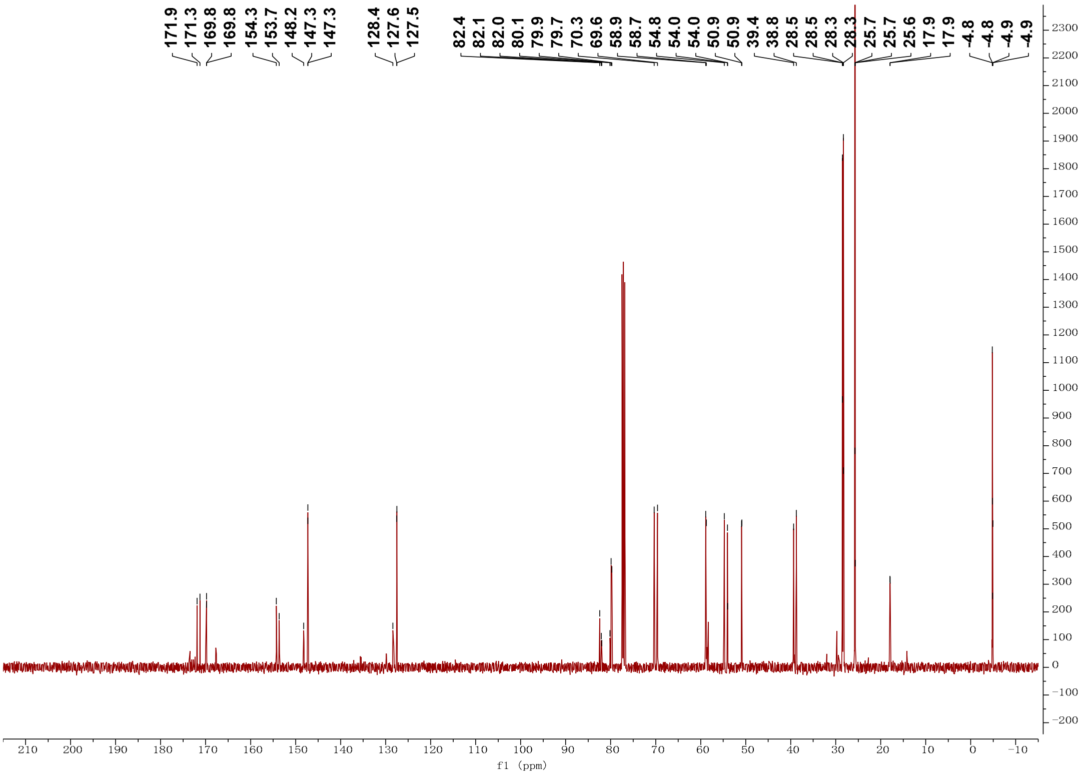

Figure S145. ^{13}C NMR spectrum of compound **4c** in $CDCl_3$ (100 MHz)

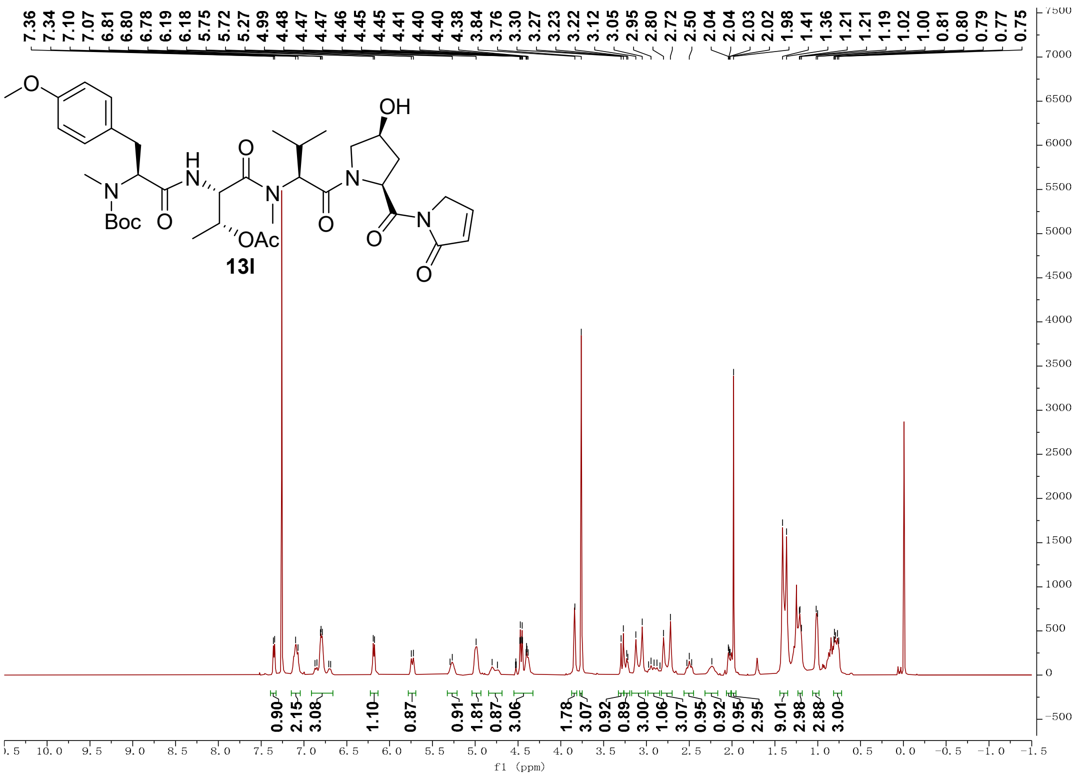

Figure S146. ^{1}H NMR spectrum of compound **13l** in $CDCl_3$ (400 MHz)

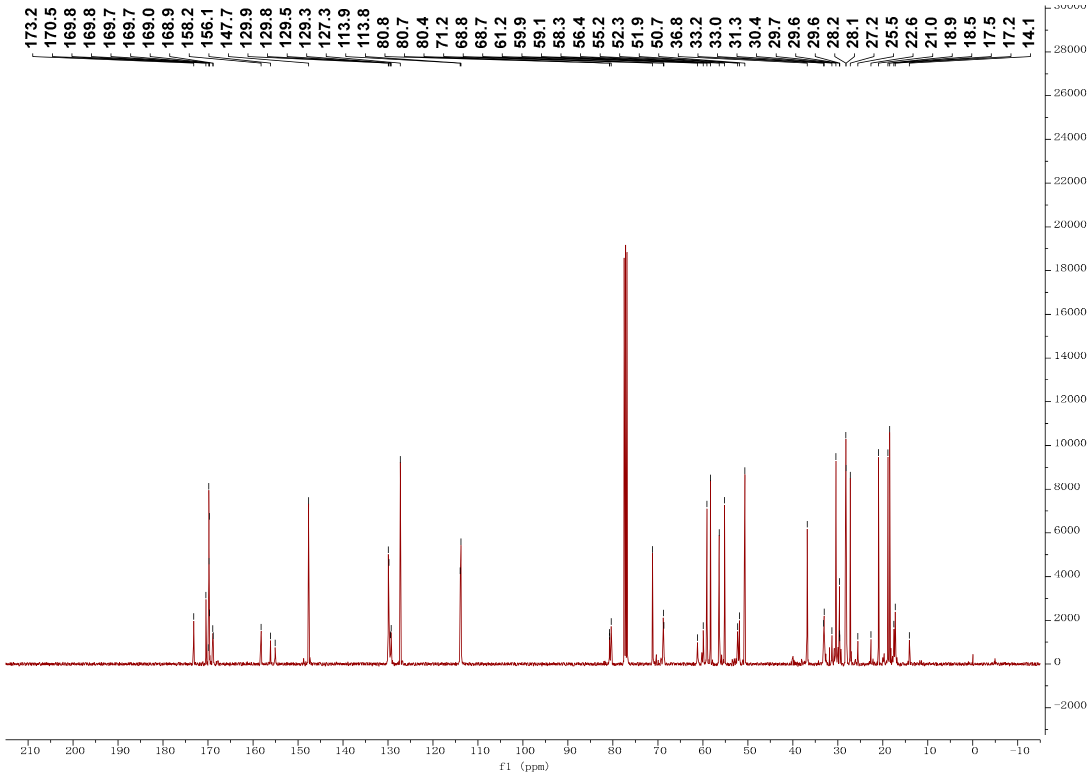

Figure S147. ^{13}C NMR spectrum of compound **13l** in $CDCl_3$ (100 MHz)

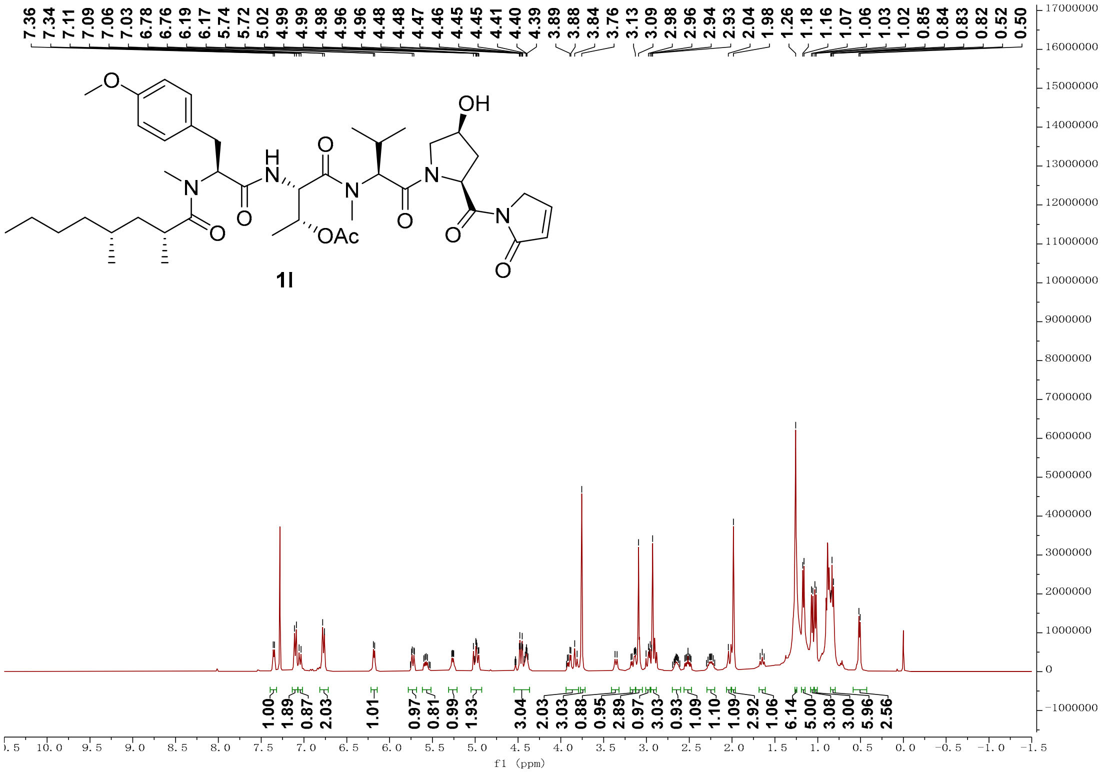

Figure S148. ^{1}H NMR spectrum of compound **1l** in $CDCl_3$ (400 MHz)

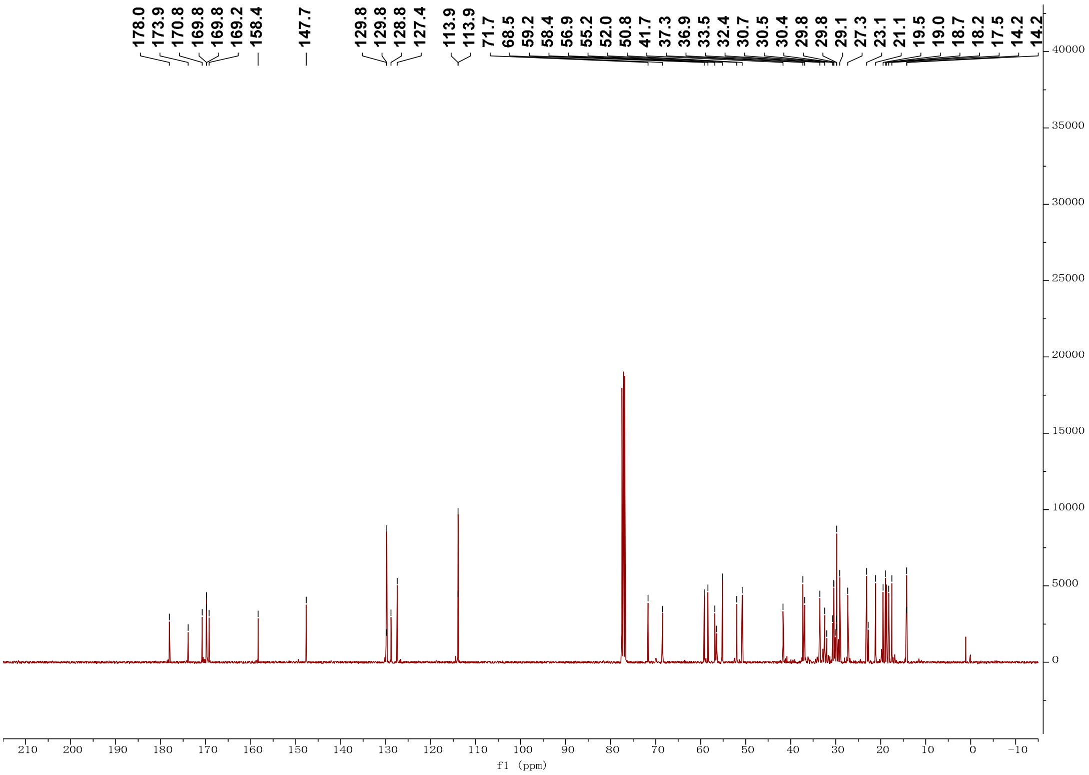

Figure S149. ^{13}C NMR spectrum of compound **1l** in $CDCl_3$ (100 MHz)

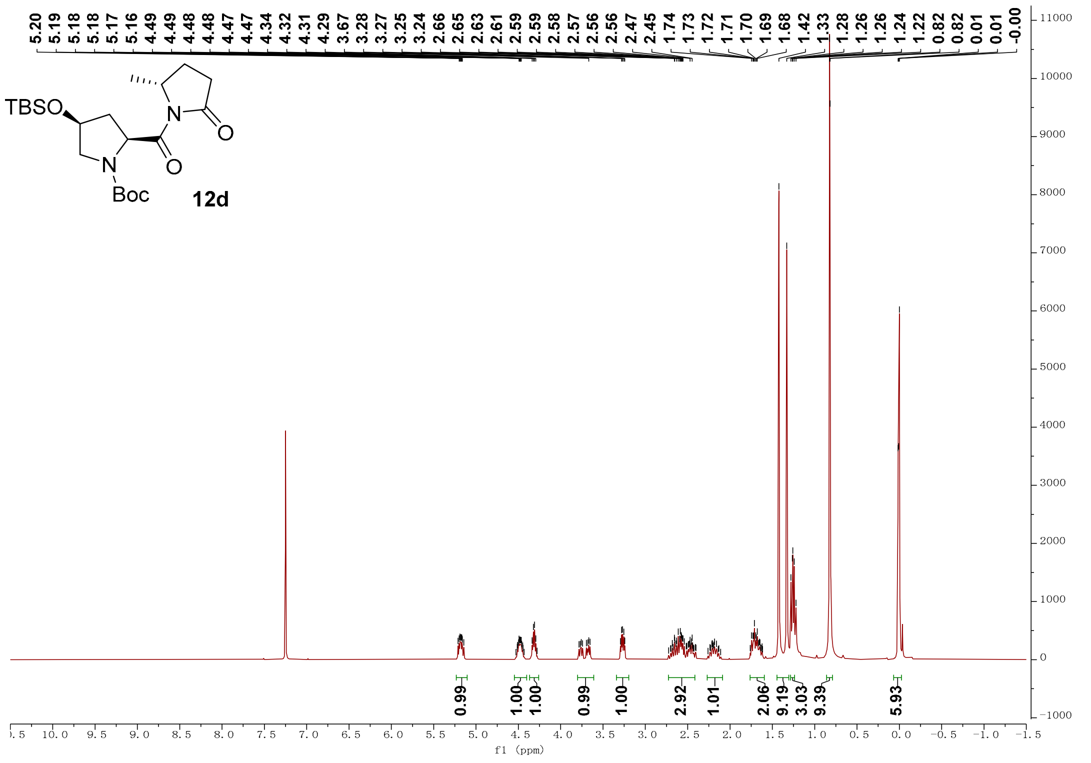

Figure S150. ^{1}H NMR spectrum of compound **12d** in $CDCl_3$ (400 MHz)

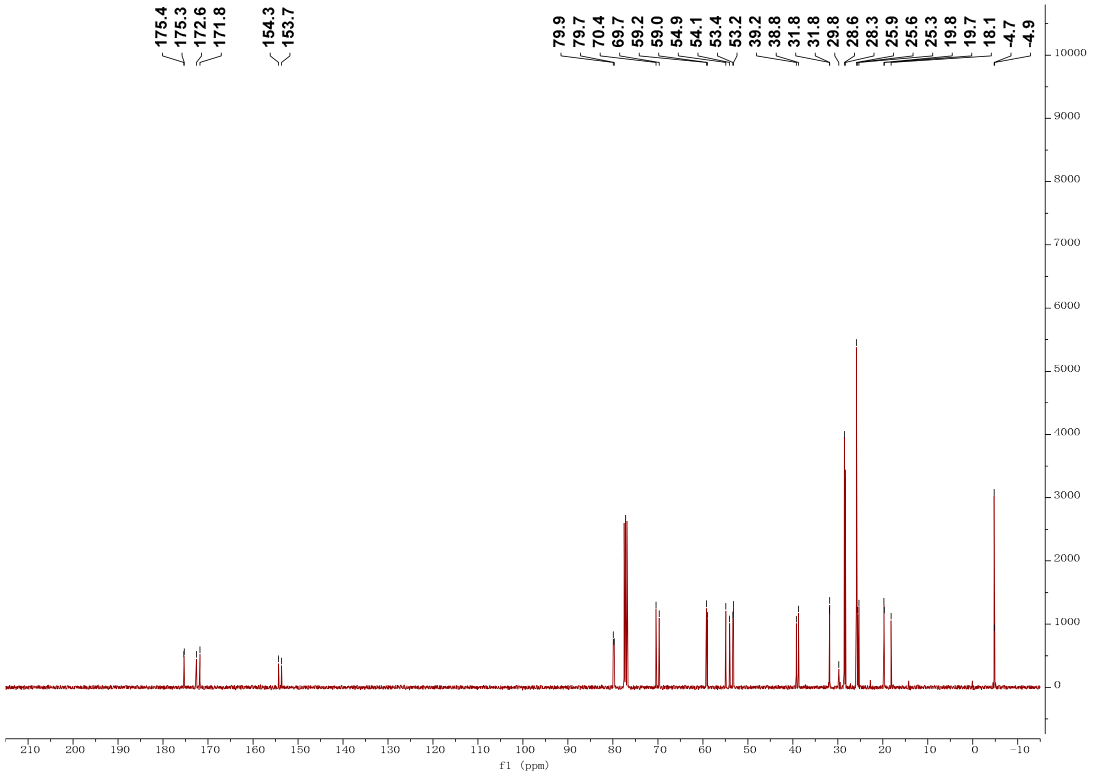

Figure S151. ^{13}C NMR spectrum of compound **12d** in $CDCl_3$ (100 MHz)

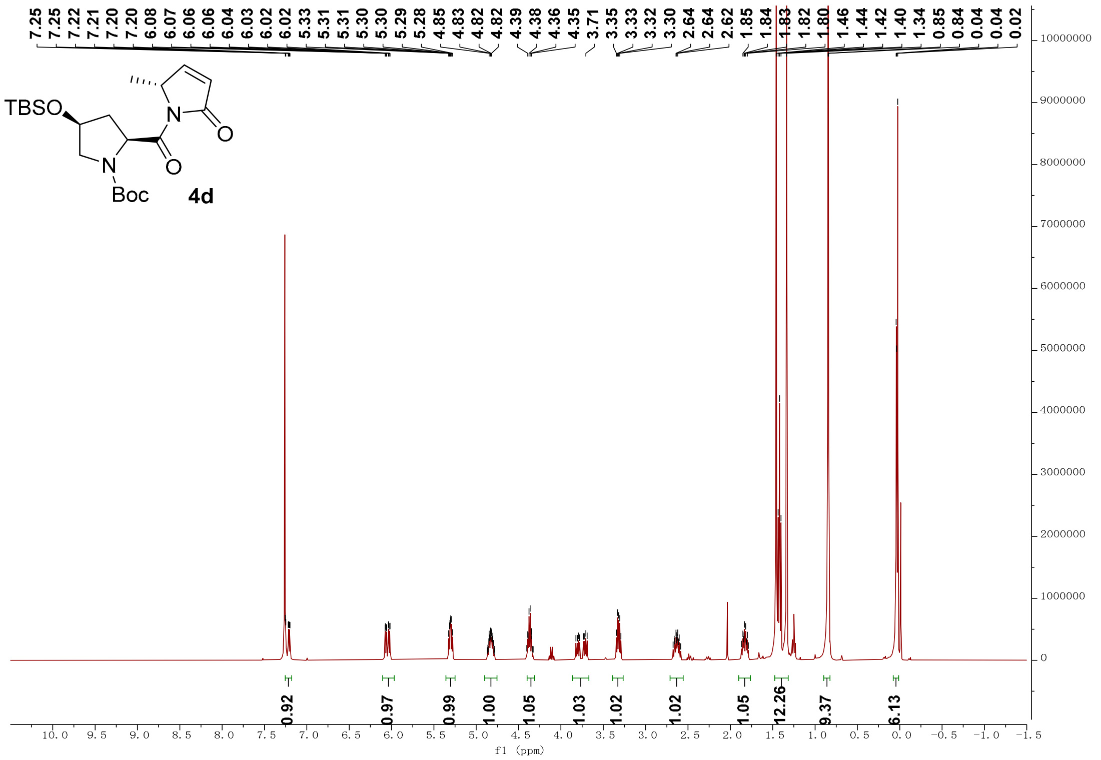

Figure S152. ^{1}H NMR spectrum of compound **4d** in $CDCl_3$ (400 MHz)

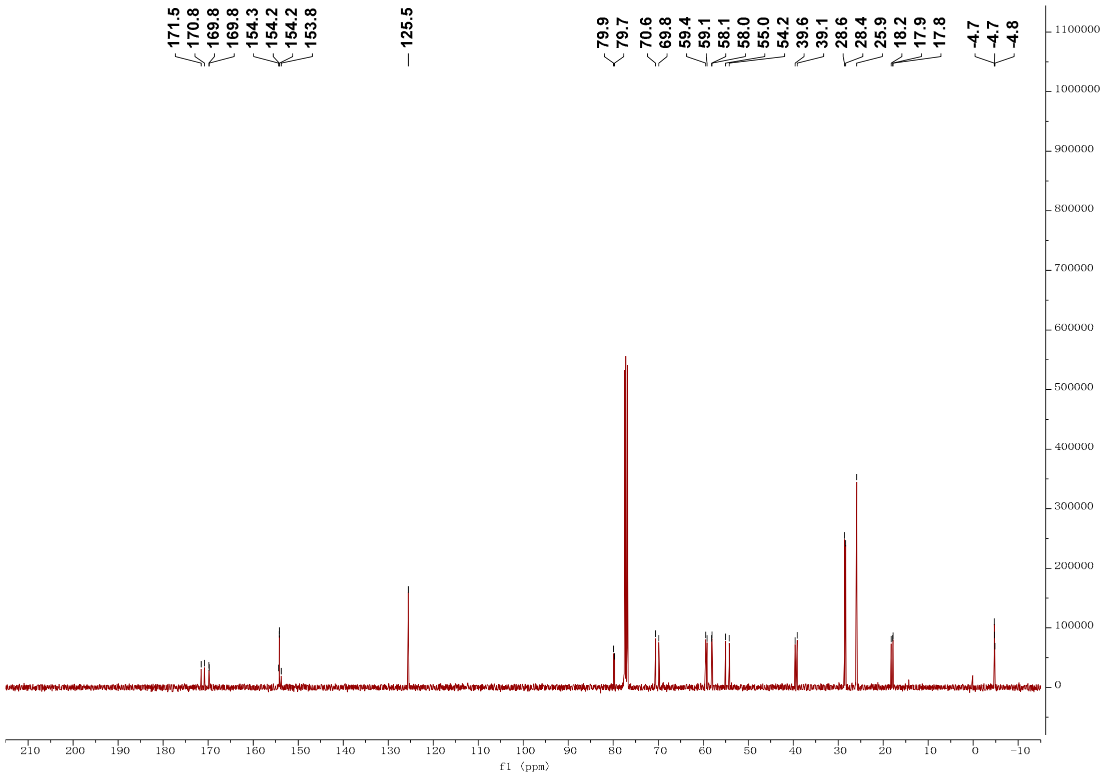

Figure S153. ^{13}C NMR spectrum of compound **4d** in $CDCl_3$ (100 MHz)

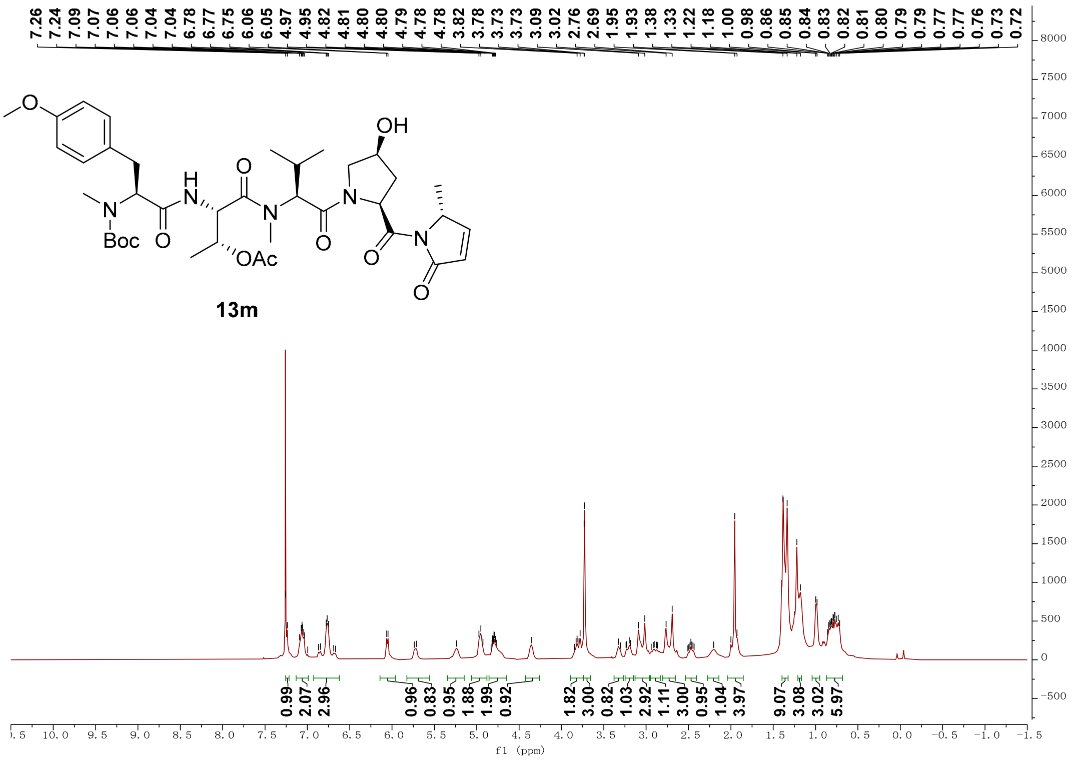

Figure S154. ^{1}H NMR spectrum of compound **13m** in $CDCl_3$ (400 MHz)

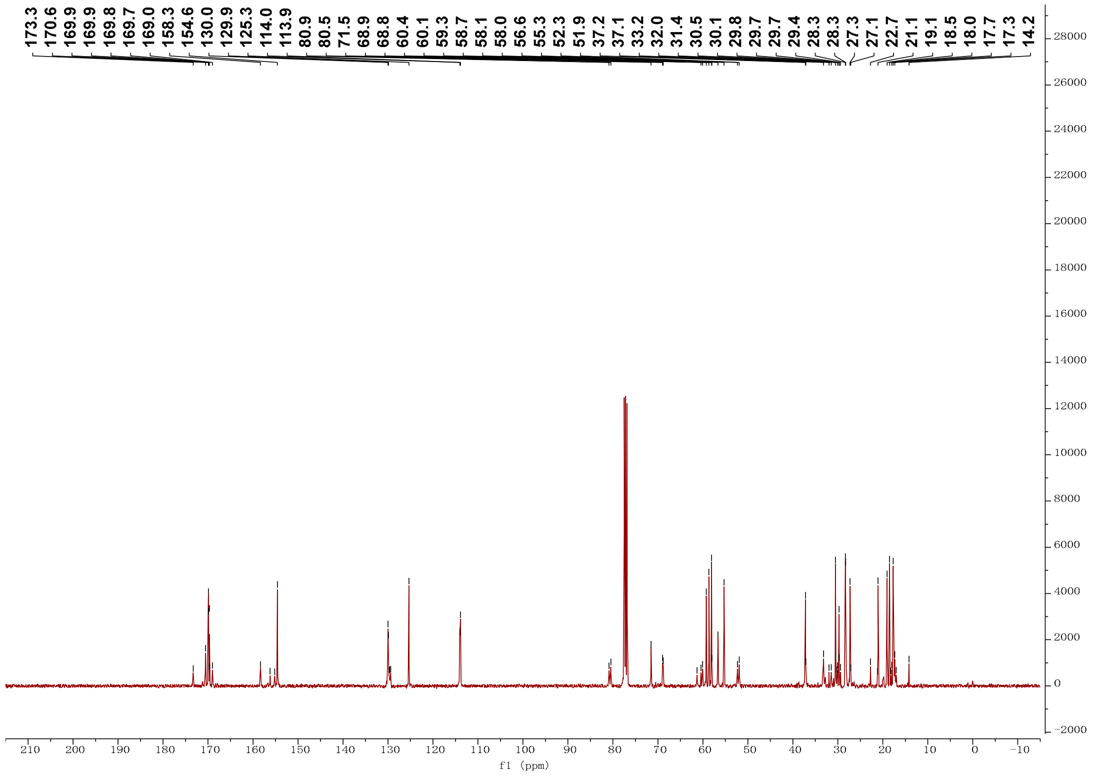

Figure S155. ^{13}C NMR spectrum of compound **13m** in $CDCl_3$ (100 MHz)

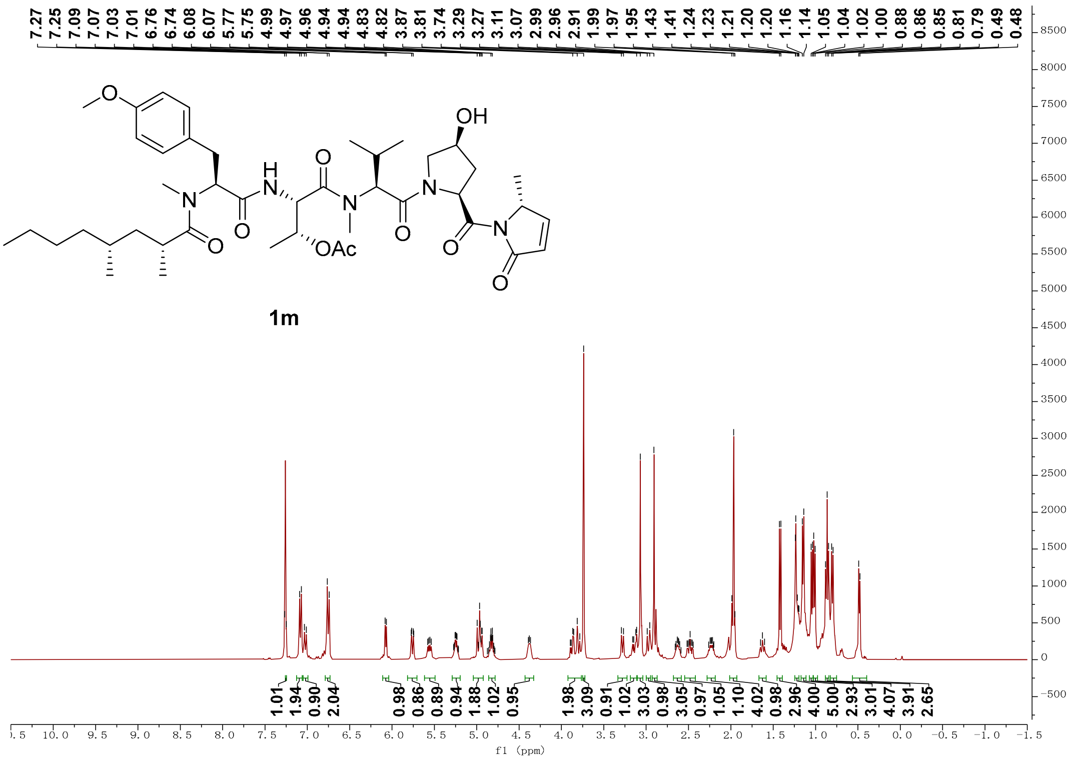

Figure S156. ^{1}H NMR spectrum of compound **1m** in $CDCl_3$ (400 MHz)

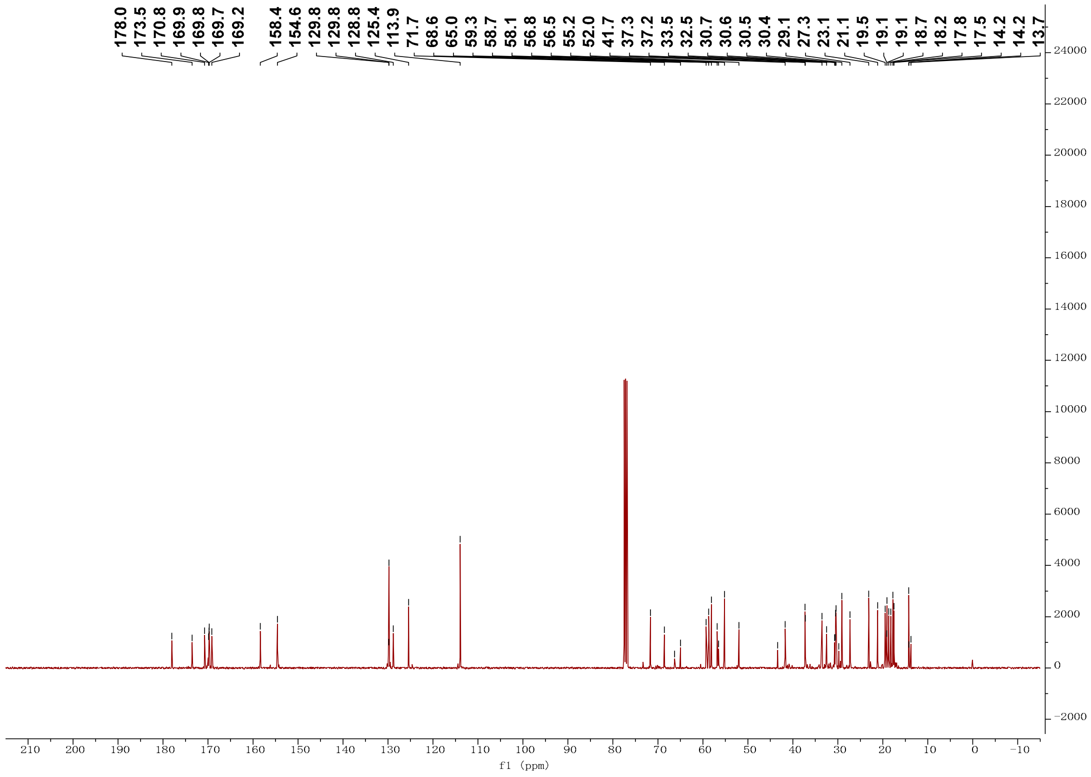

Figure S157. ^{13}C NMR spectrum of compound **1m** in $CDCl_3$ (100 MHz)

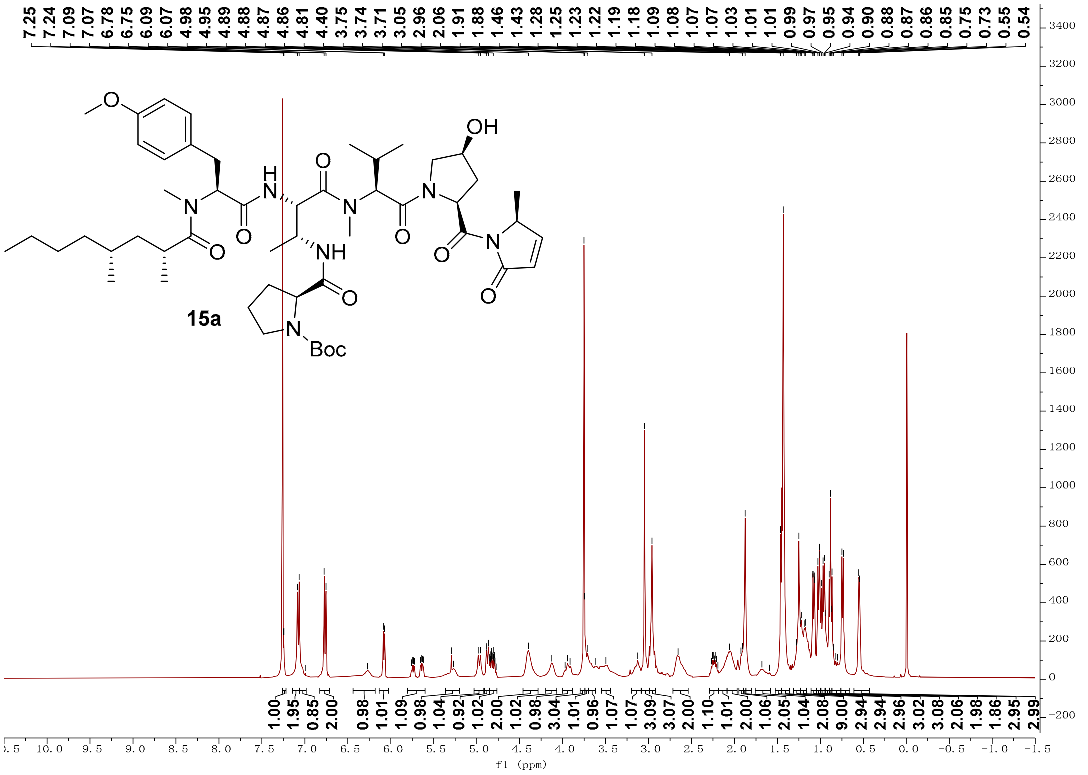

Figure S158. ^{1}H NMR spectrum of compound **15a** in $CDCl_3$ (400 MHz)

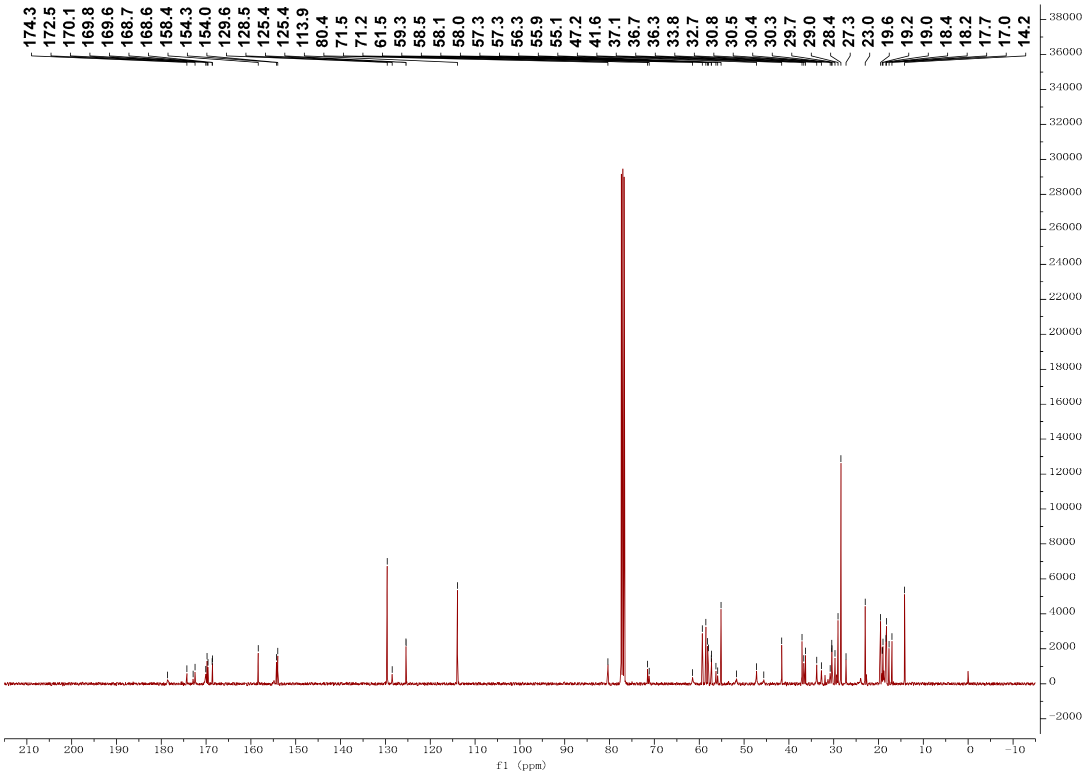

Figure S159. ^{13}C NMR spectrum of compound **15a** in $CDCl_3$ (100 MHz)

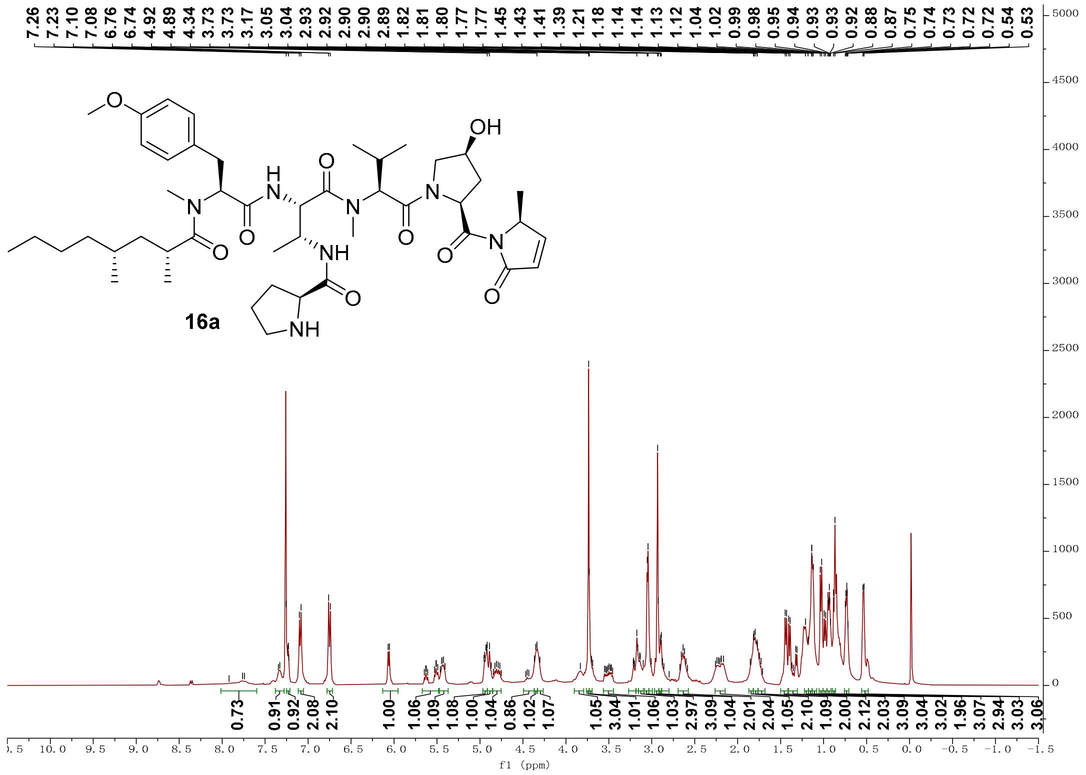

Figure S160. ^{1}H NMR spectrum of compound **16a** in $CDCl_3$ (400 MHz)

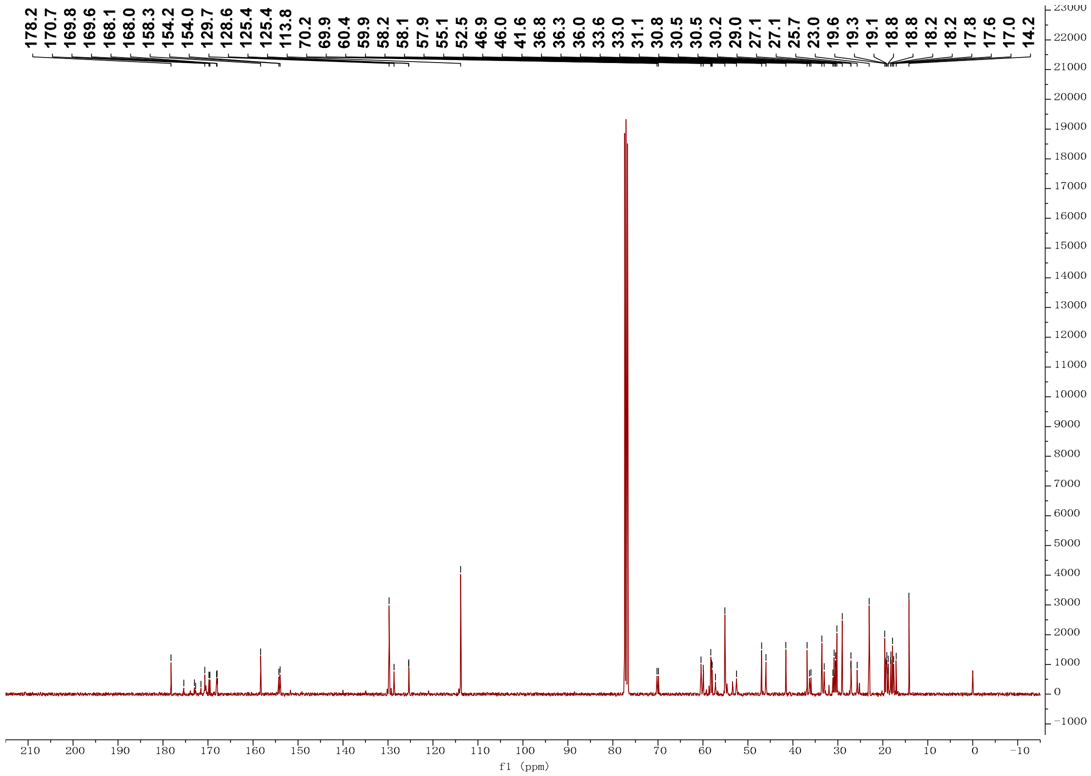

Figure S161. ^{13}C NMR spectrum of compound **16a** in $CDCl_3$ (100 MHz)

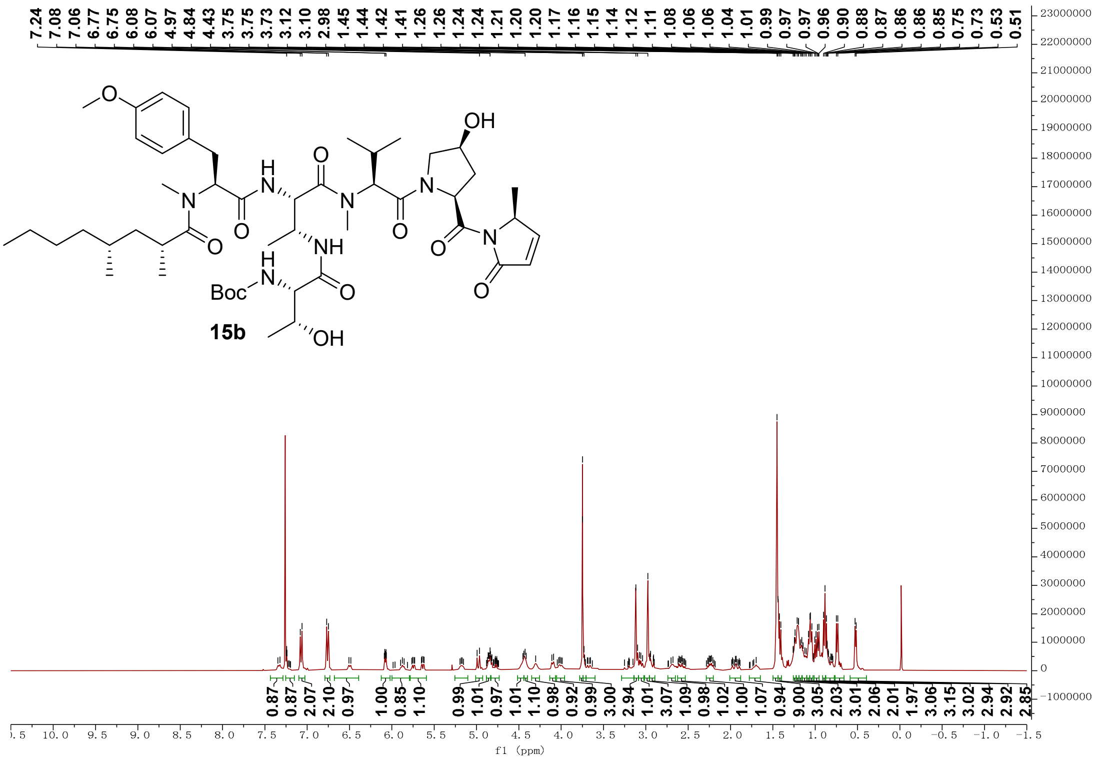

Figure S162. ^{1}H NMR spectrum of compound **15b** in $CDCl_3$ (400 MHz)

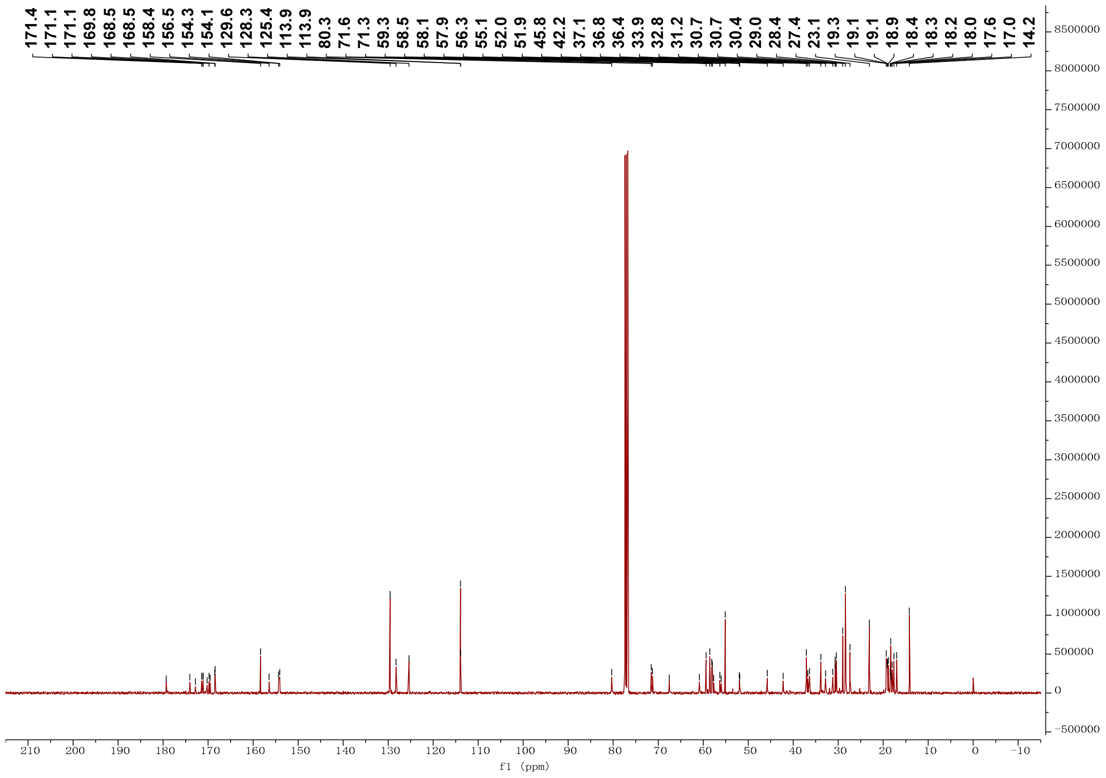

Figure S163. ^{13}C NMR spectrum of compound **15b** in $CDCl_3$ (100 MHz)

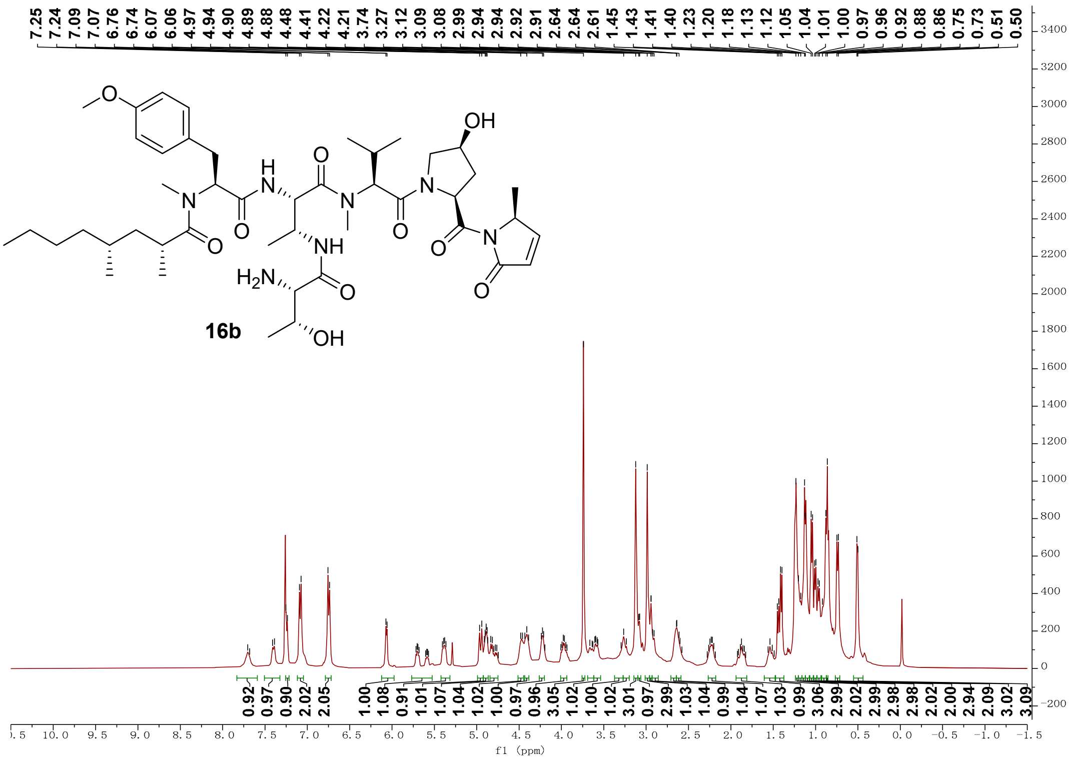

Figure S164. ^{1}H NMR spectrum of compound **16b** in $CDCl_3$ (400 MHz)

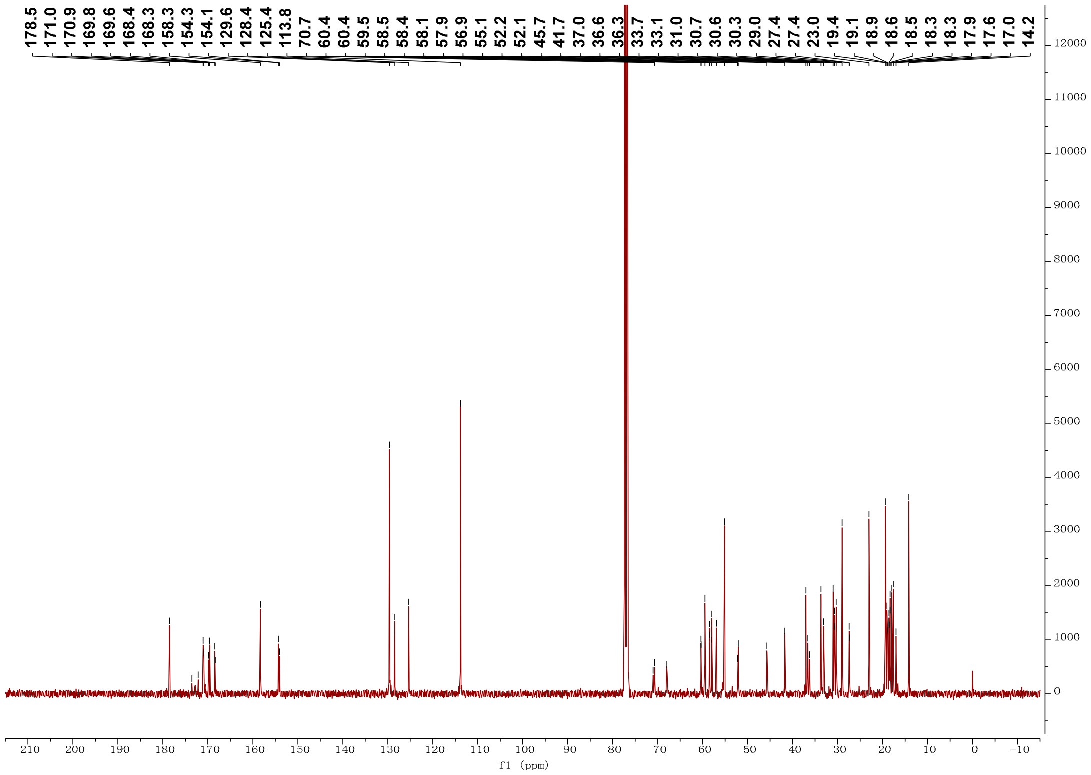

Figure S165. ^{13}C NMR spectrum of compound **16b** in $CDCl_3$ (100 MHz)

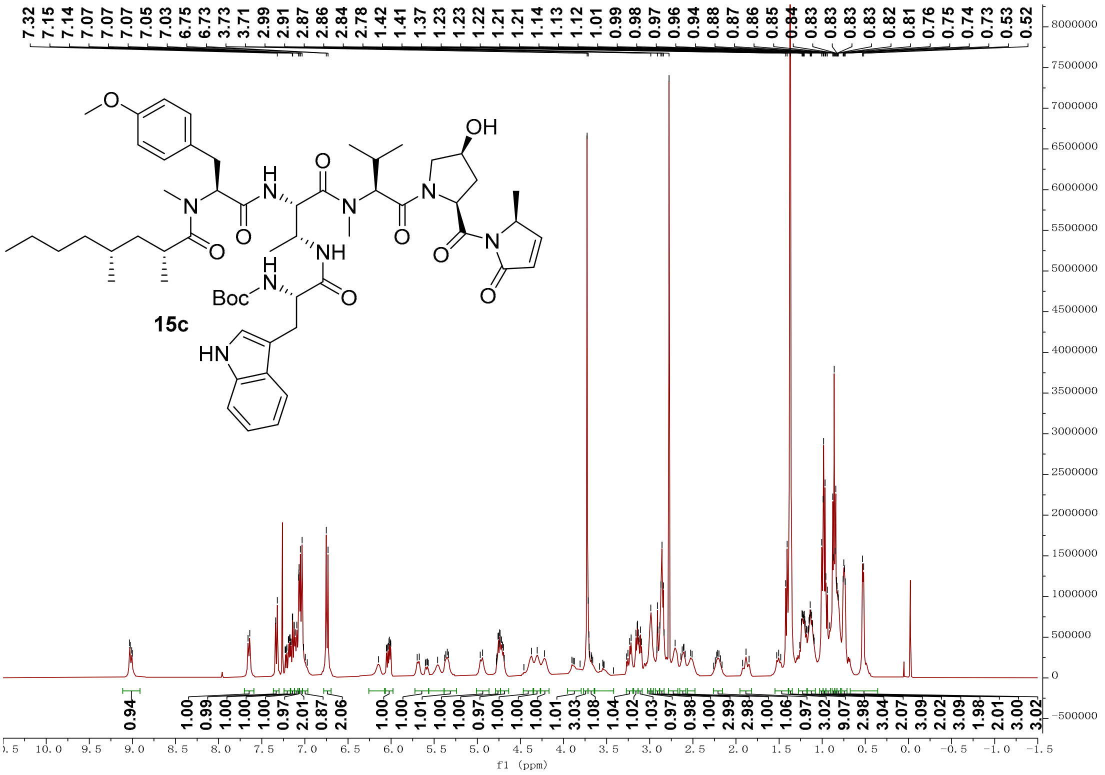

Figure S166. ^{1}H NMR spectrum of compound **15c** in $CDCl_3$ (400 MHz)

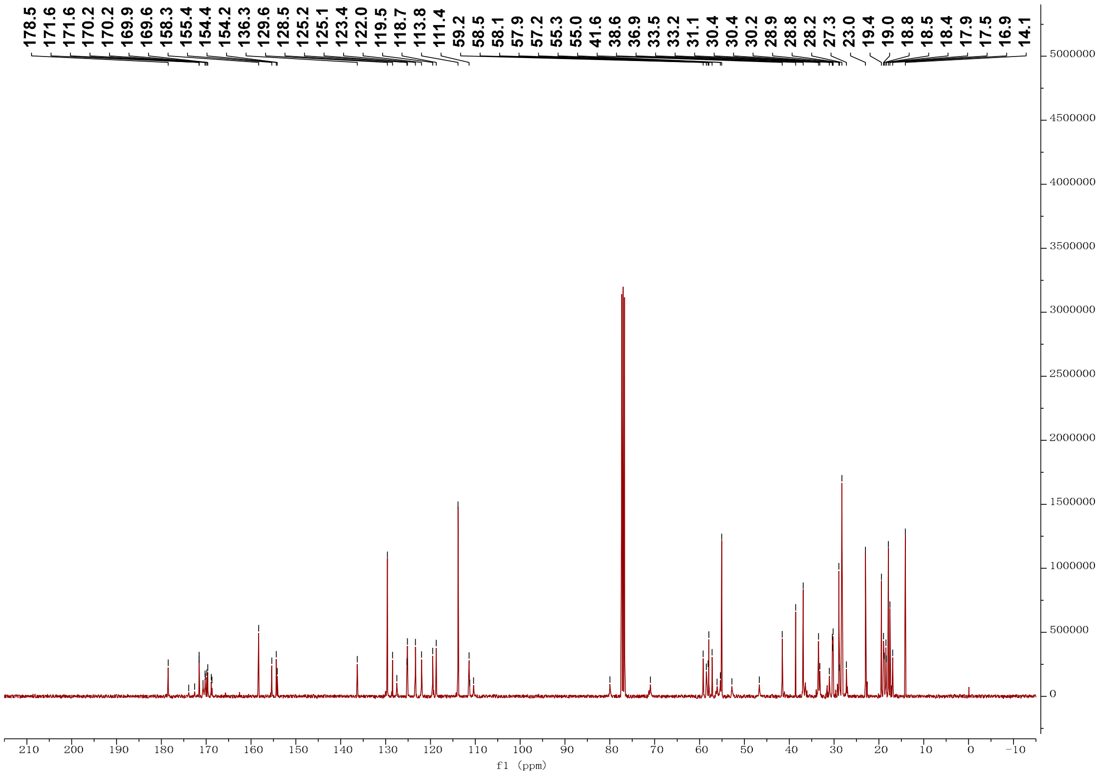

Figure S167. ^{13}C NMR spectrum of compound **15c** in $CDCl_3$ (100 MHz)

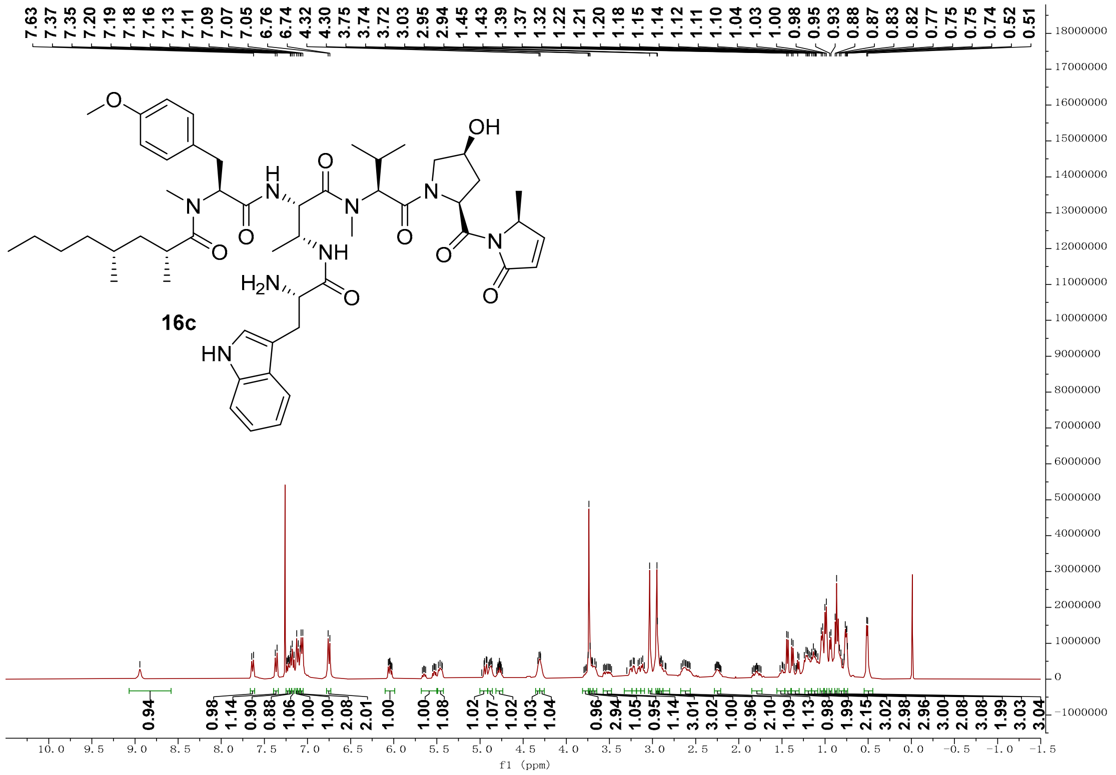

Figure S168. ^{1}H NMR spectrum of compound **16c** in $CDCl_3$ (400 MHz)

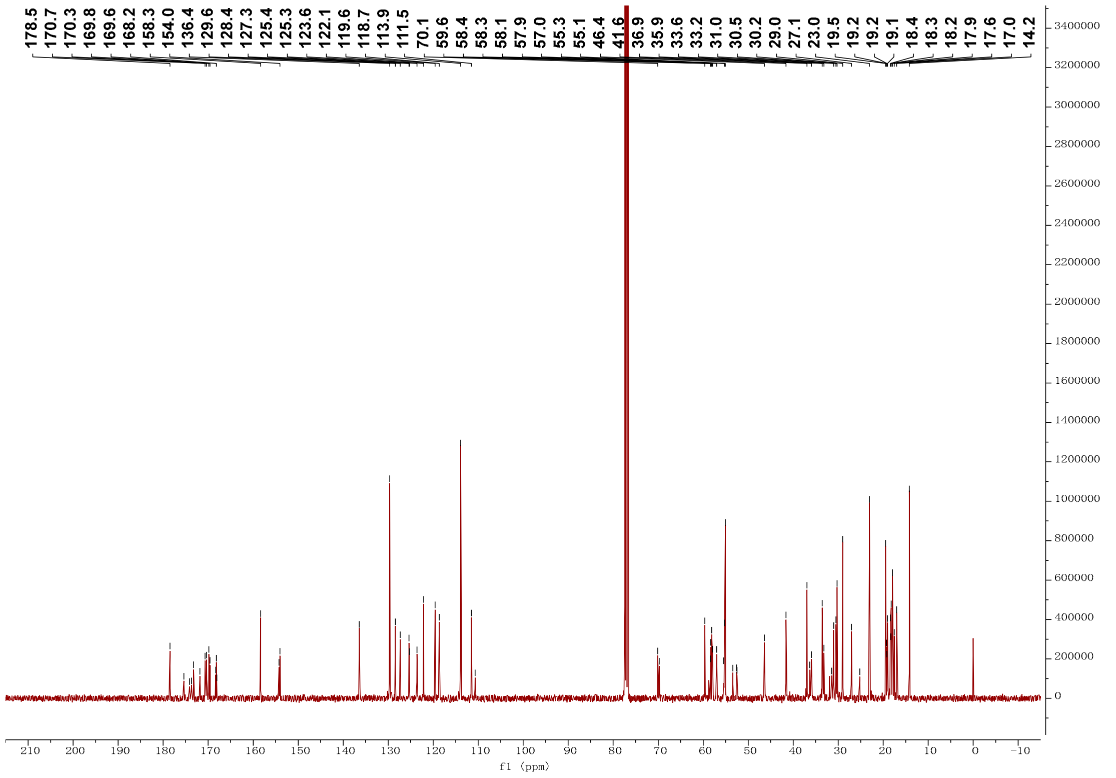

Figure S169. ^{13}C NMR spectrum of compound **16c** in $CDCl_3$ (100 MHz)

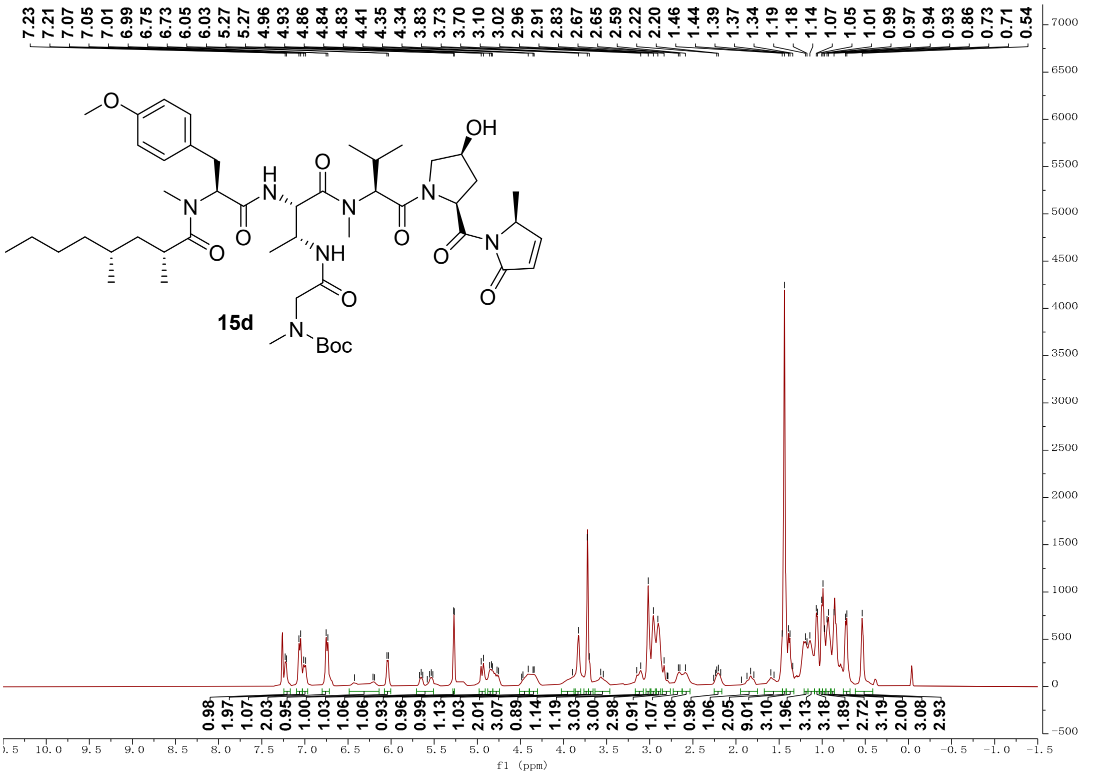

Figure S170. ^{1}H NMR spectrum of compound **15d** in $CDCl_3$ (400 MHz)

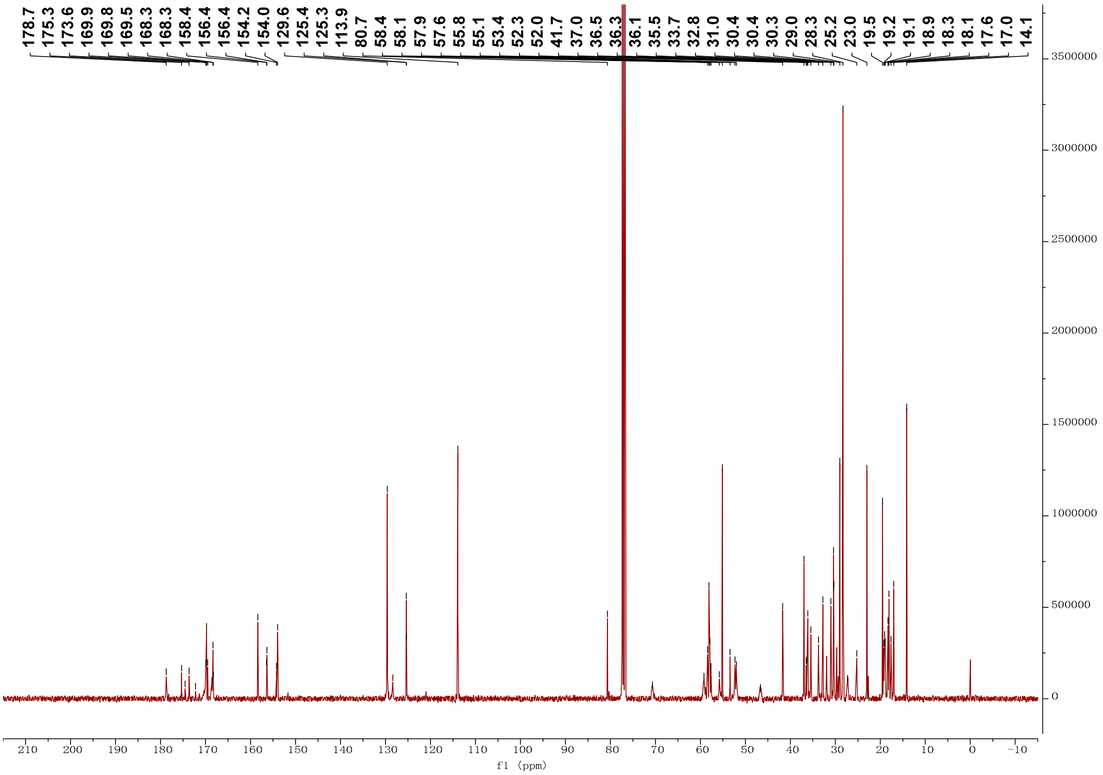

Figure S171. ^{13}C NMR spectrum of compound **15d** in $CDCl_3$ (100 MHz)

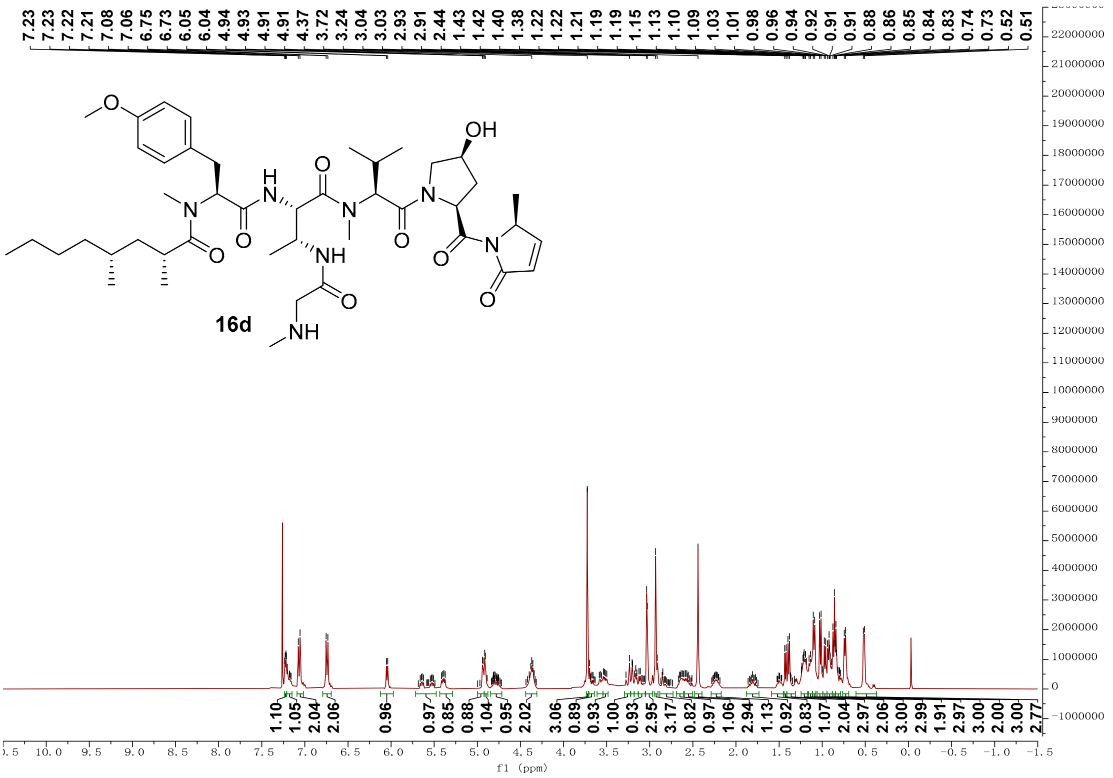

Figure S172. ^{1}H NMR spectrum of compound **16d** in $CDCl_3$ (400 MHz)

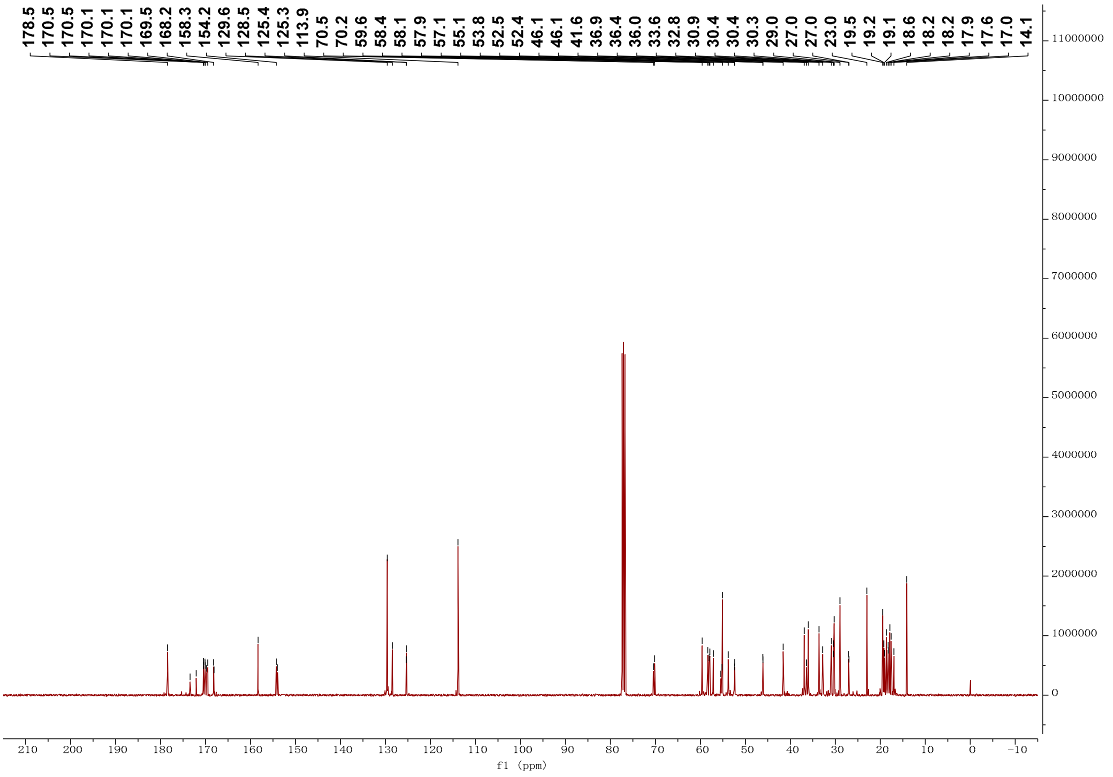

Figure S173. ^{13}C NMR spectrum of compound **16d** in $CDCl_3$ (100 MHz)

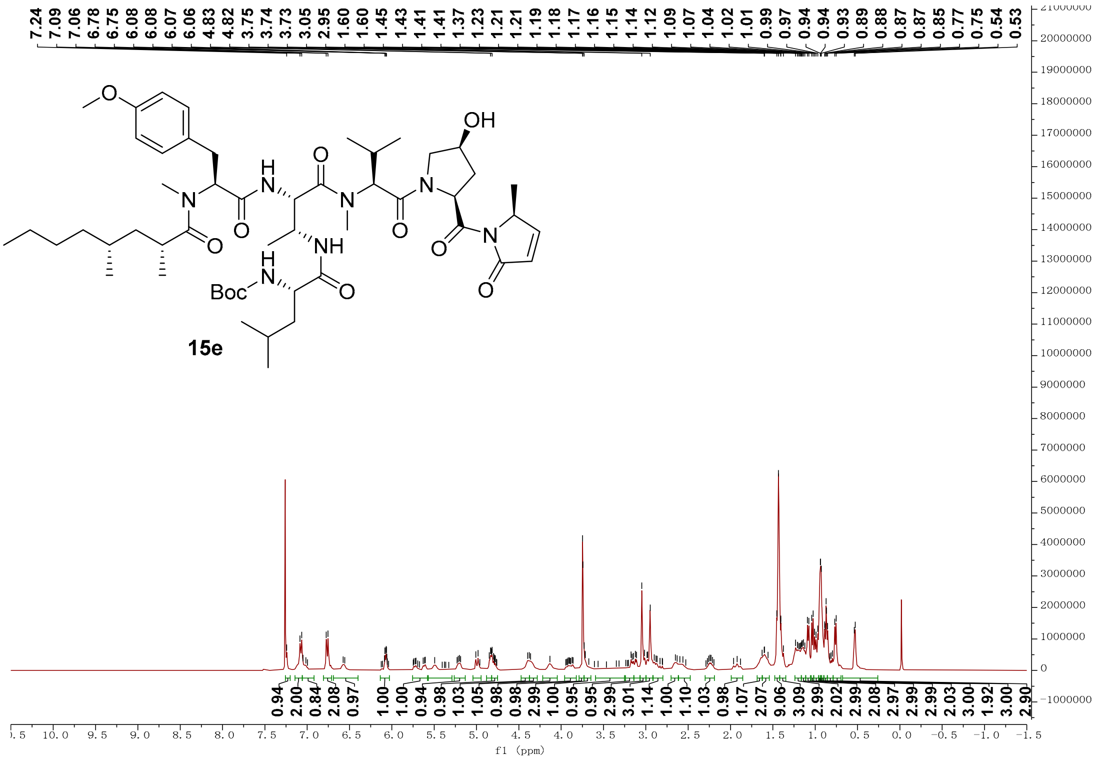

Figure S174. ^{1}H NMR spectrum of compound **15e** in $CDCl_3$ (400 MHz)

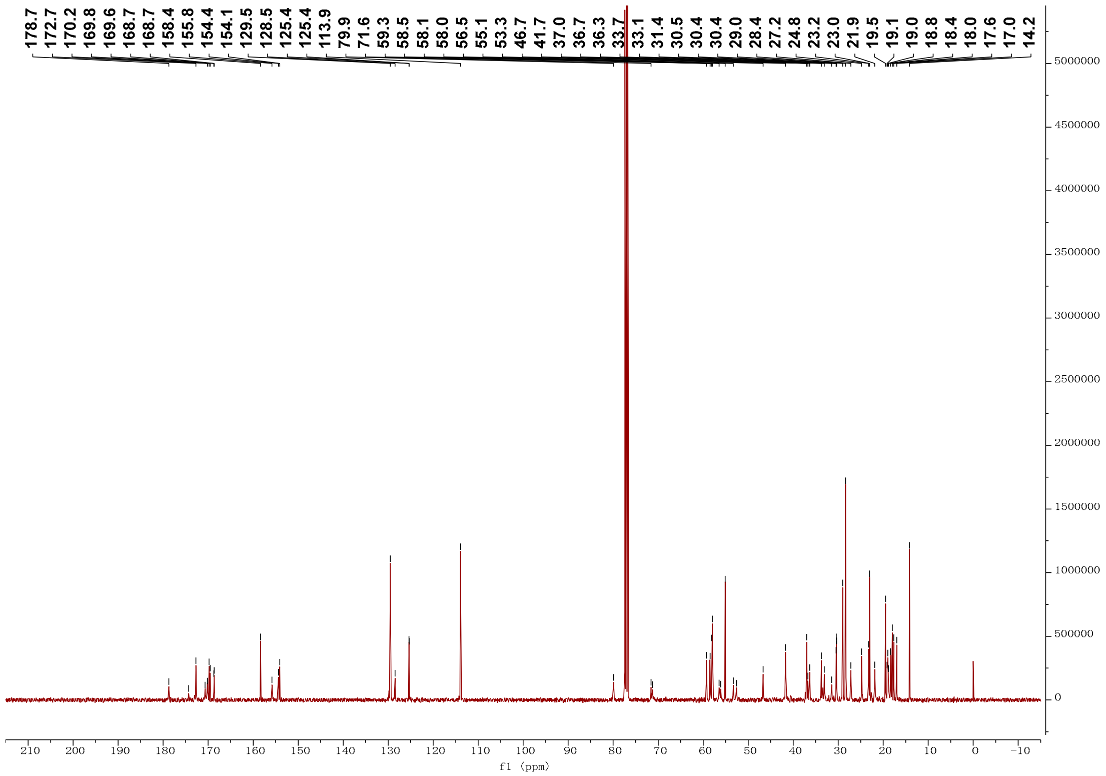

Figure S175. ^{13}C NMR spectrum of compound **15e** in $CDCl_3$ (100 MHz)

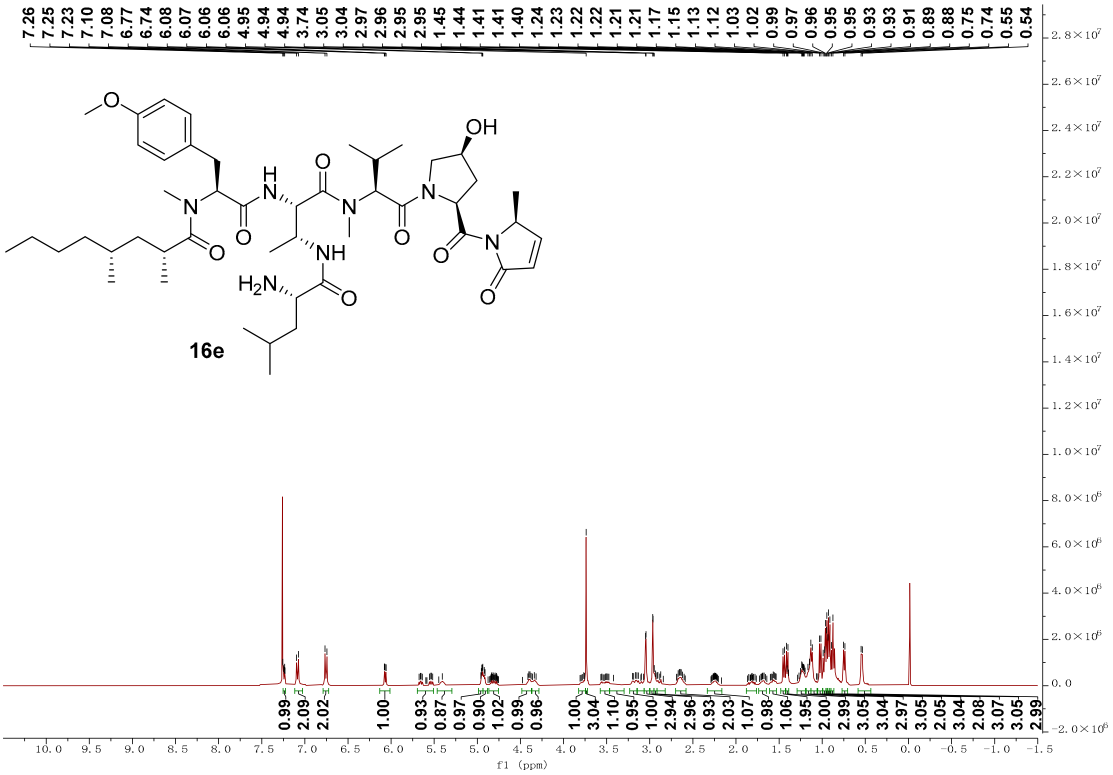

Figure S176. ^{1}H NMR spectrum of compound **16e** in $CDCl_3$ (400 MHz)

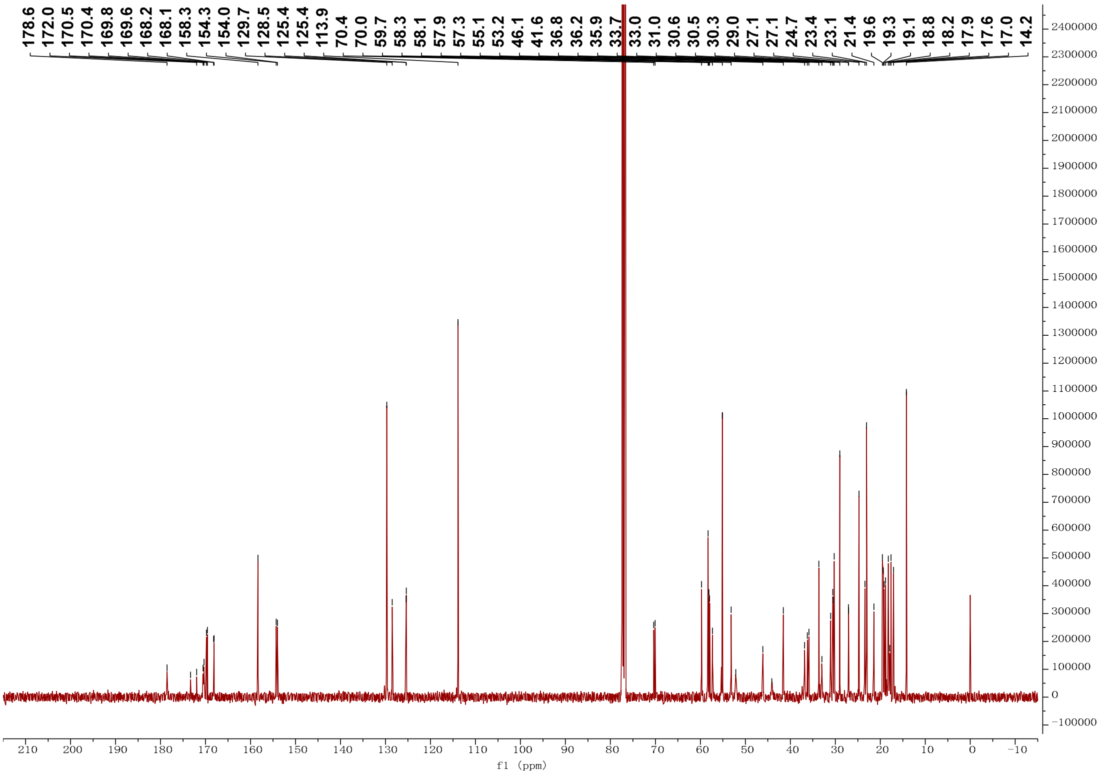

Figure S177. ^{13}C NMR spectrum of compound **16e** in $CDCl_3$ (100 MHz)

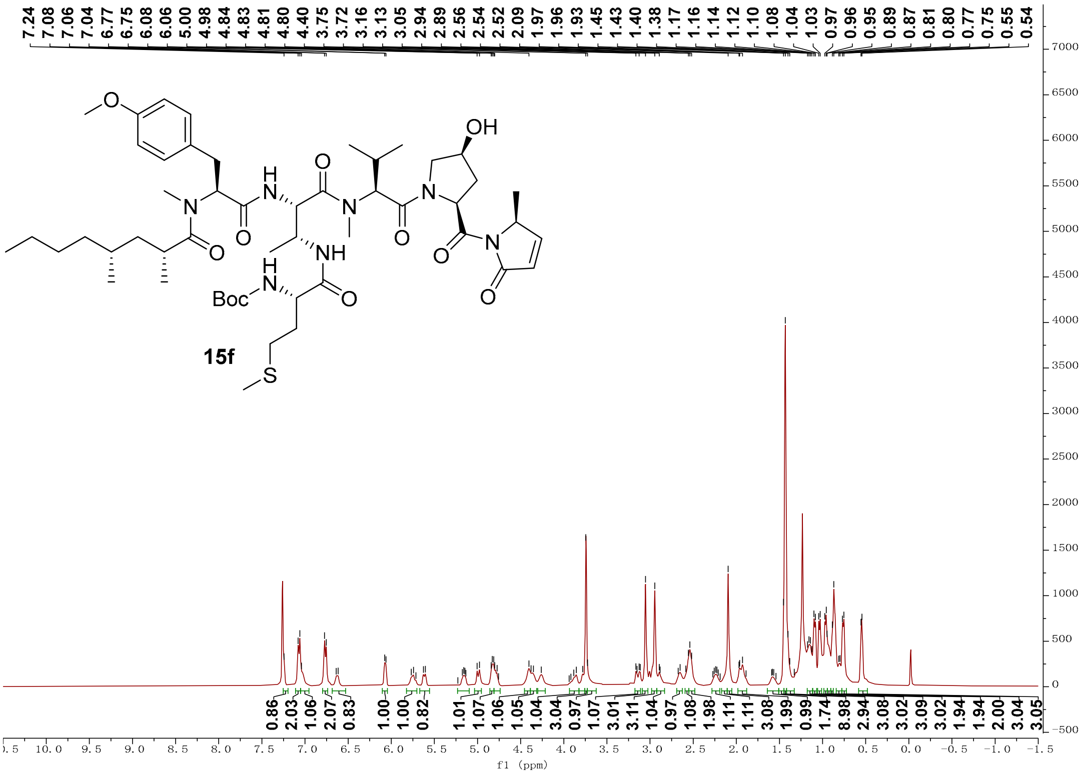

Figure S178. ^{1}H NMR spectrum of compound **15f** in $CDCl_3$ (400 MHz)

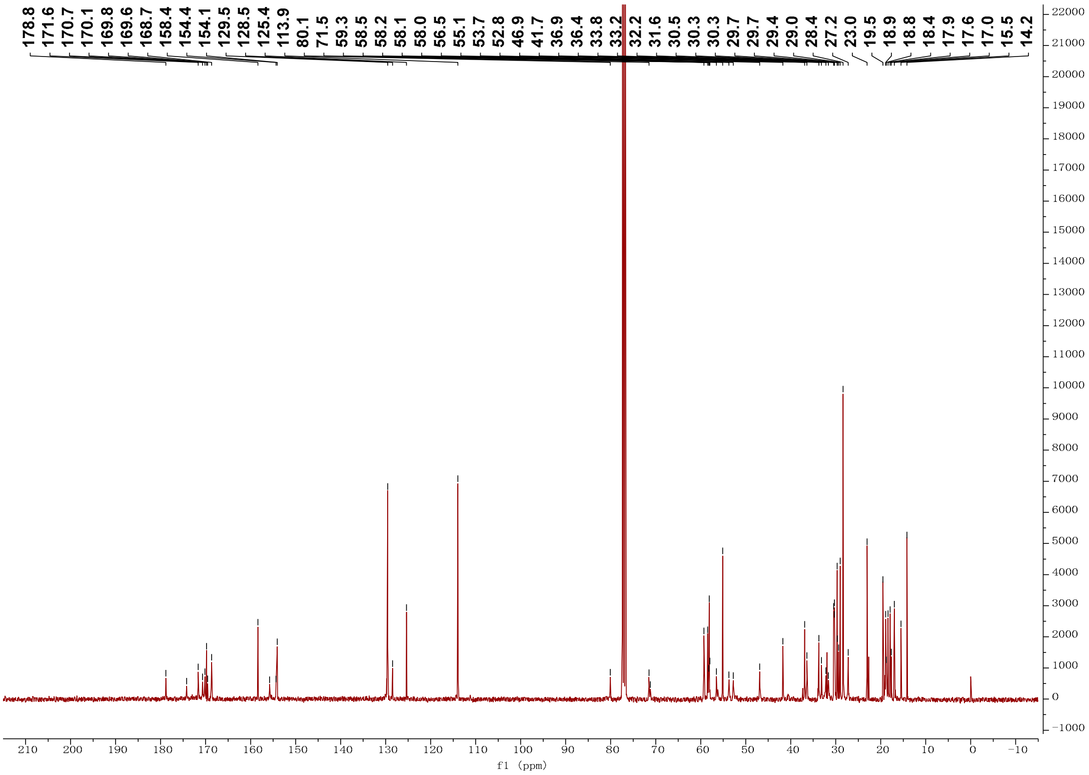

Figure S179. ^{13}C NMR spectrum of compound **15f** in $CDCl_3$ (100 MHz)

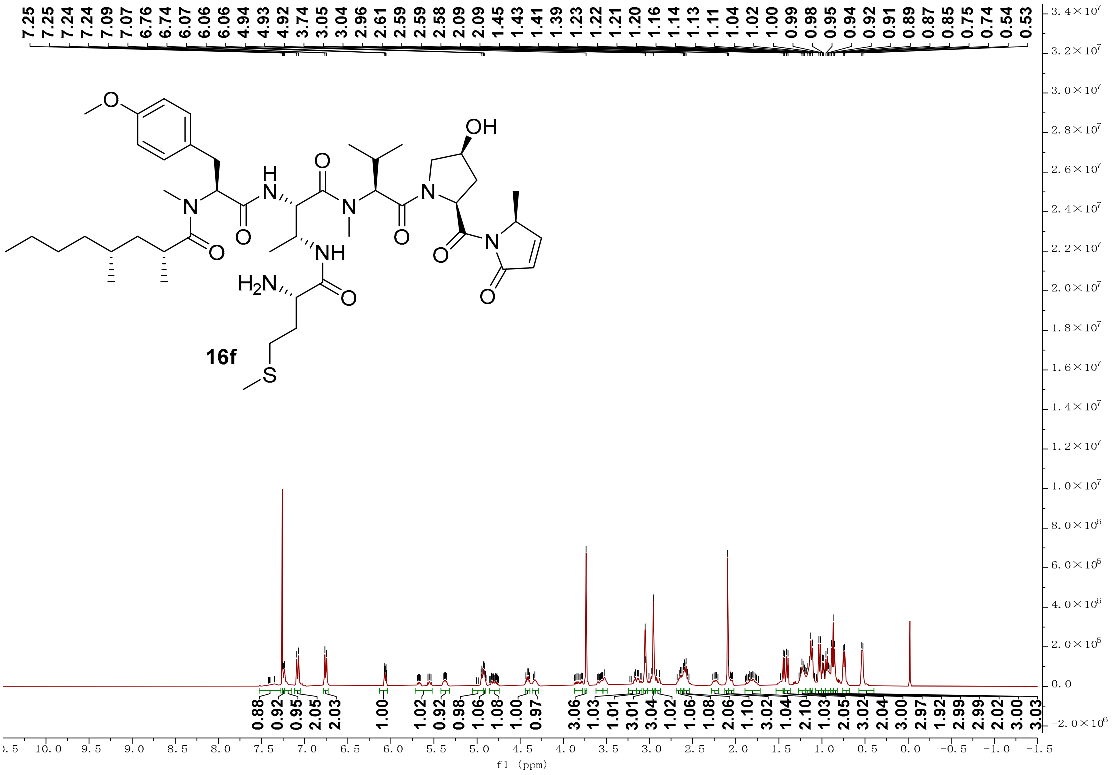

Figure S180. ^{1}H NMR spectrum of compound **16f** in $CDCl_3$ (400 MHz)

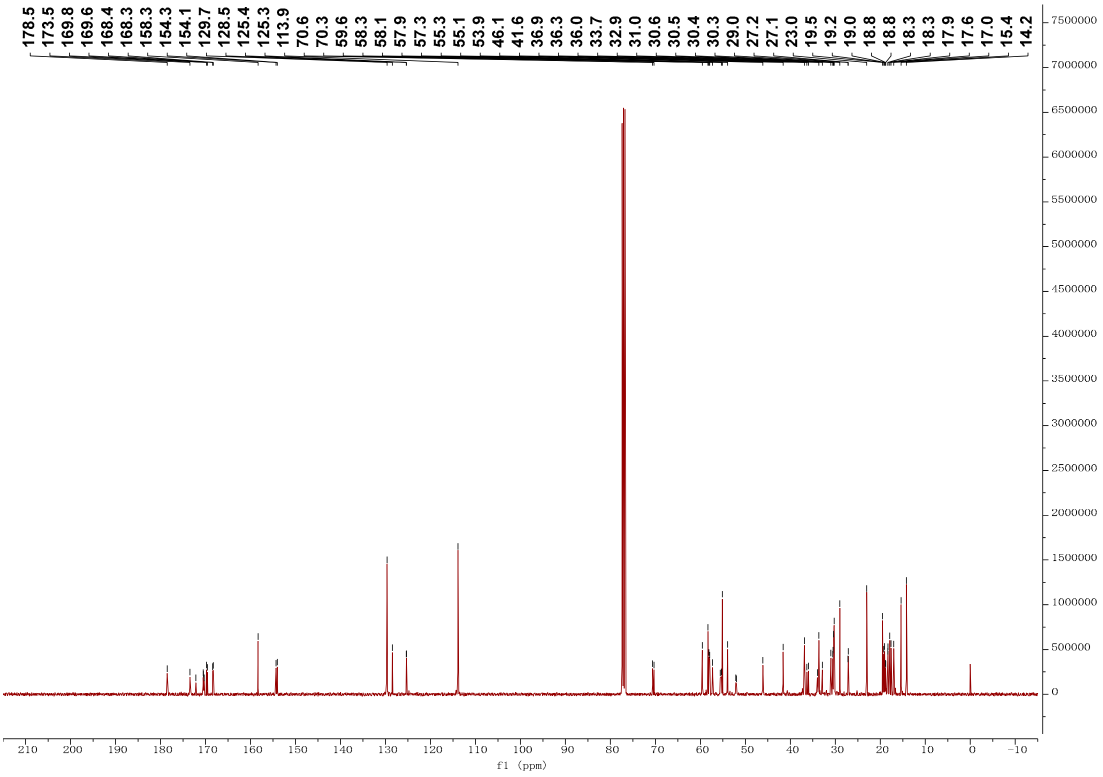

Figure S181. ^{13}C NMR spectrum of compound **16f** in $CDCl_3$ (100 MHz)

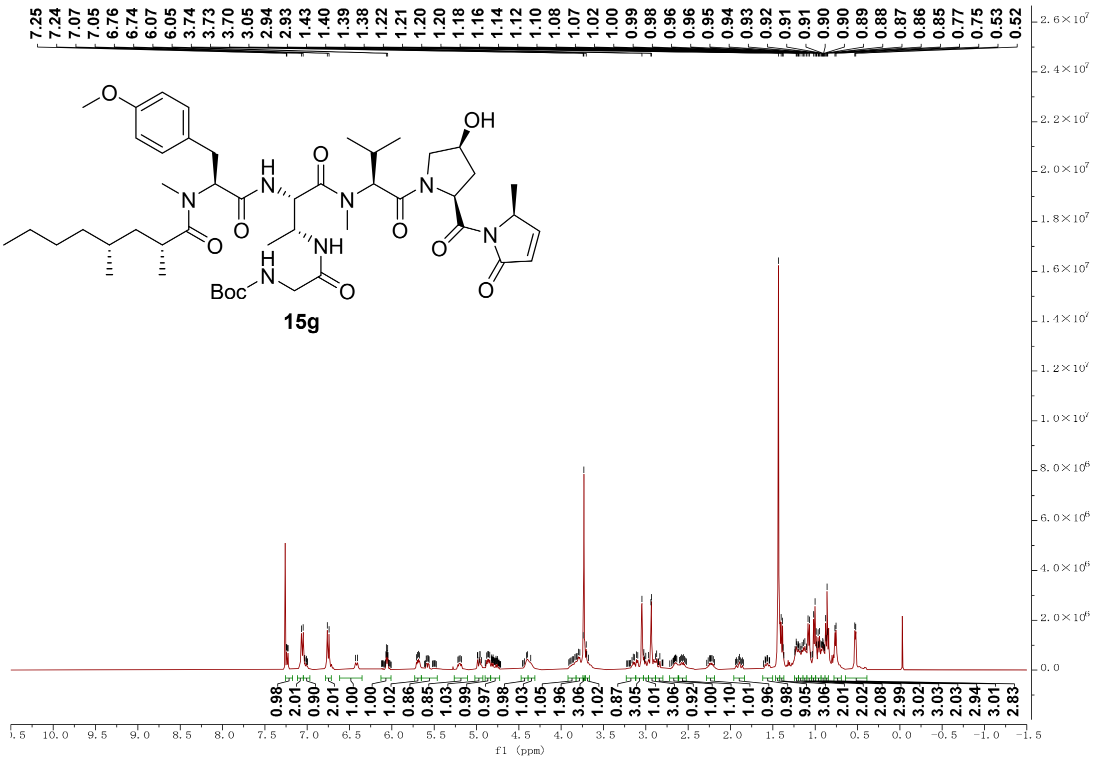

Figure S182. ^{1}H NMR spectrum of compound **15g** in $CDCl_3$ (400 MHz)

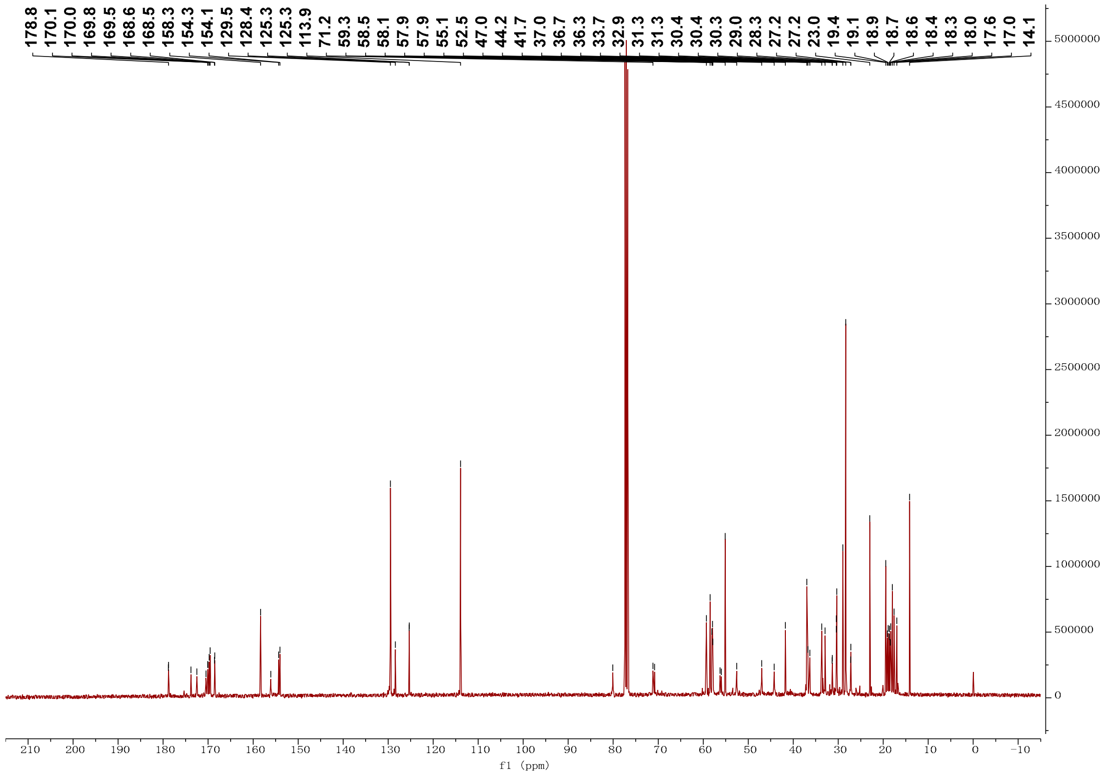

Figure S183. ^{13}C NMR spectrum of compound **15g** in $CDCl_3$ (100 MHz)

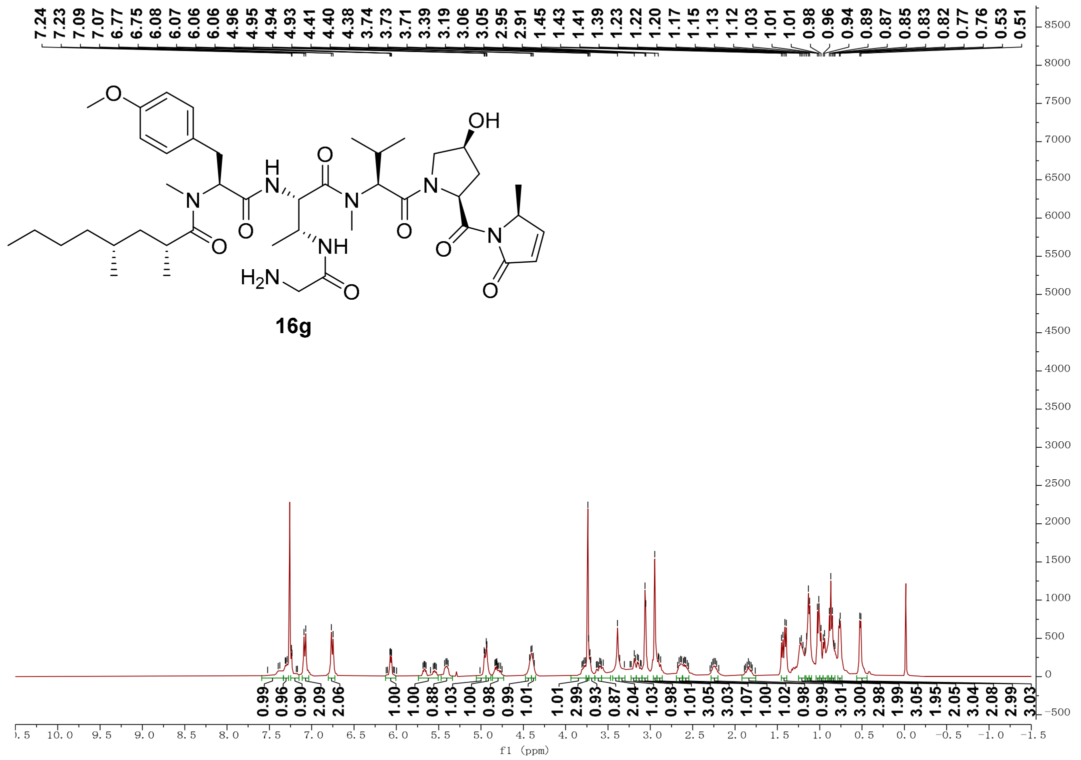

Figure S184. ^{1}H NMR spectrum of compound **16g** in $CDCl_3$ (400 MHz)

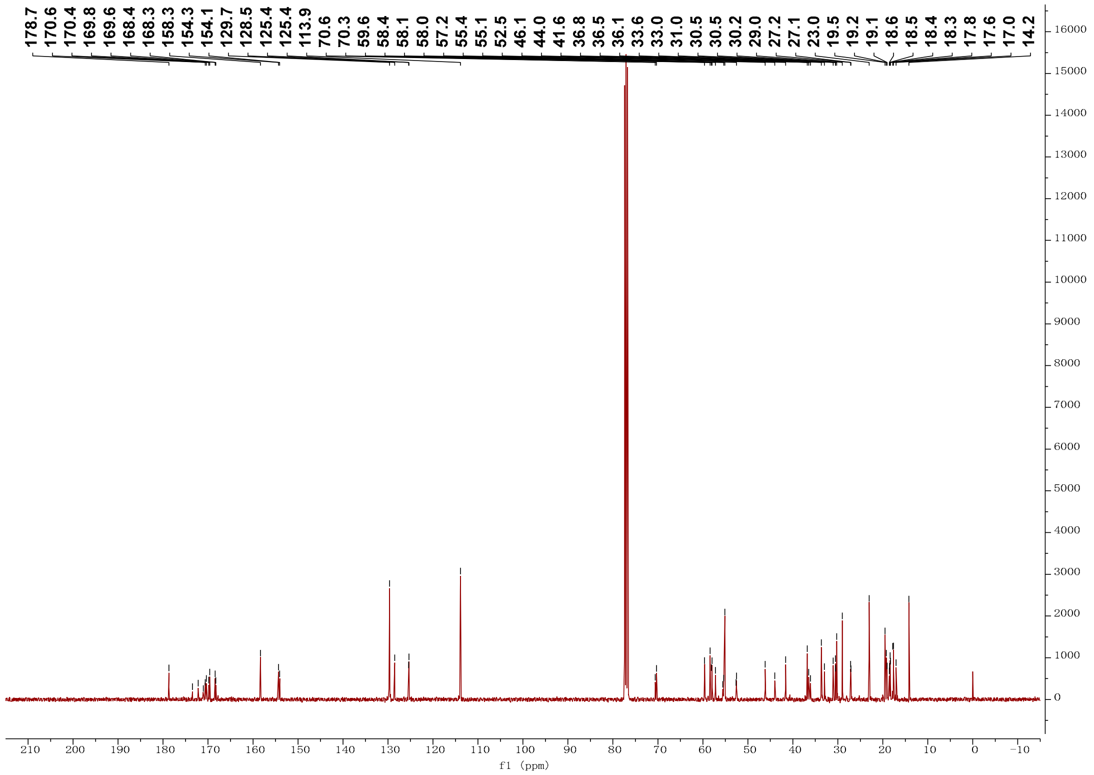

Figure S185. ^{13}C NMR spectrum of compound **16g** in $CDCl_3$ (100 MHz)

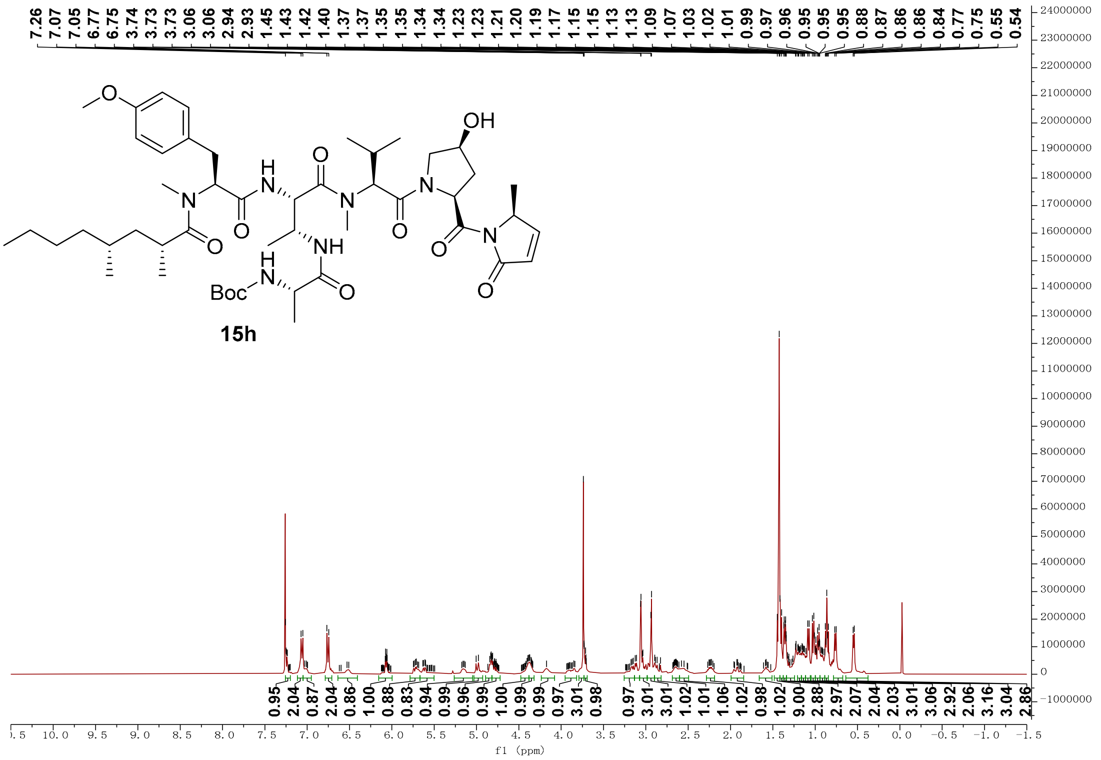

Figure S186. ^{1}H NMR spectrum of compound **15h** in $CDCl_3$ (400 MHz)

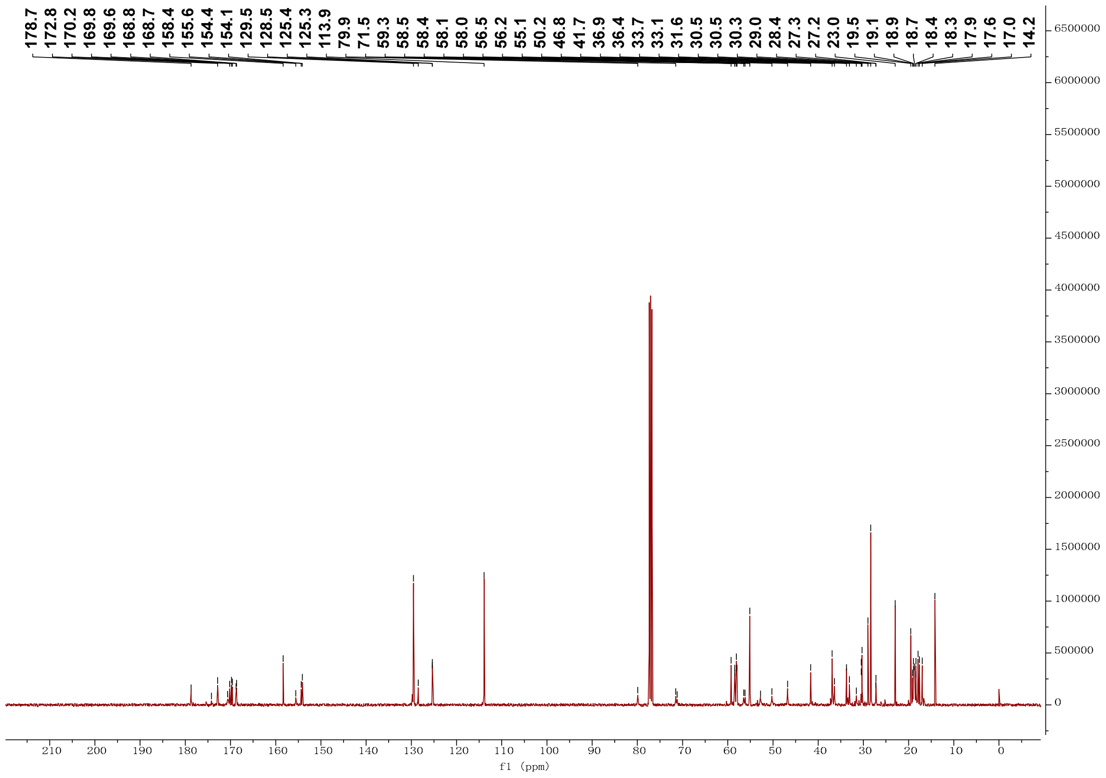

Figure S187. ^{13}C NMR spectrum of compound **15h** in $CDCl_3$ (100 MHz)

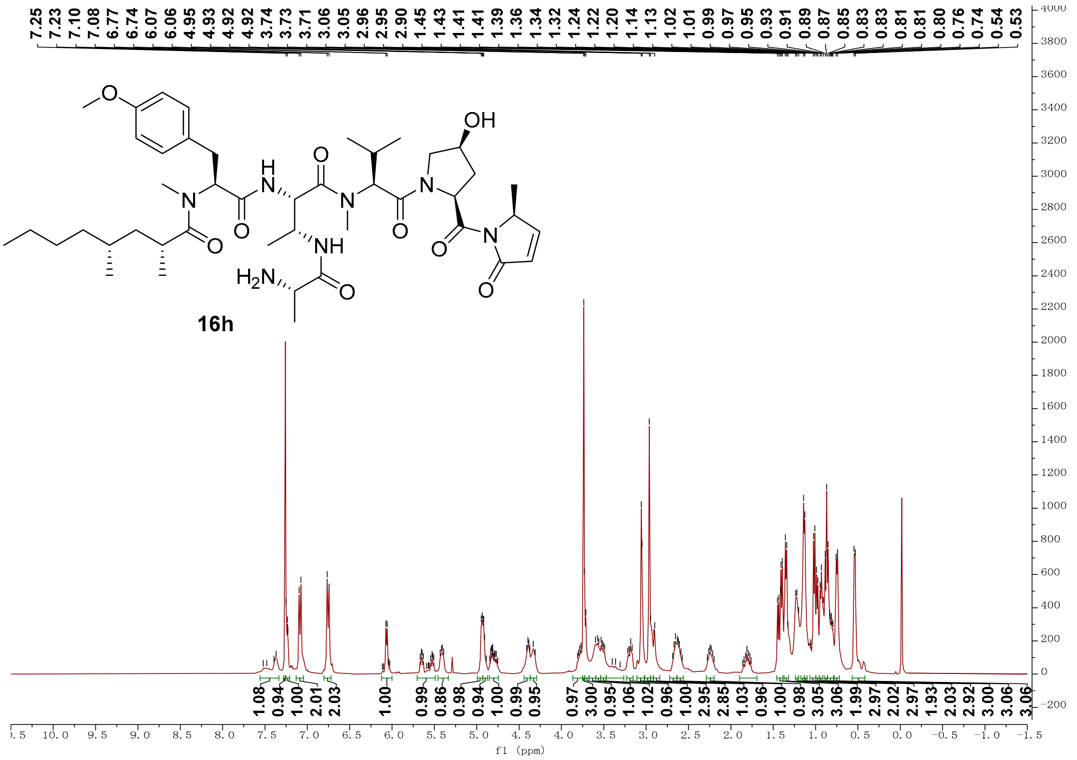

Figure S188. ^{1}H NMR spectrum of compound **16h** in $CDCl_3$ (400 MHz)

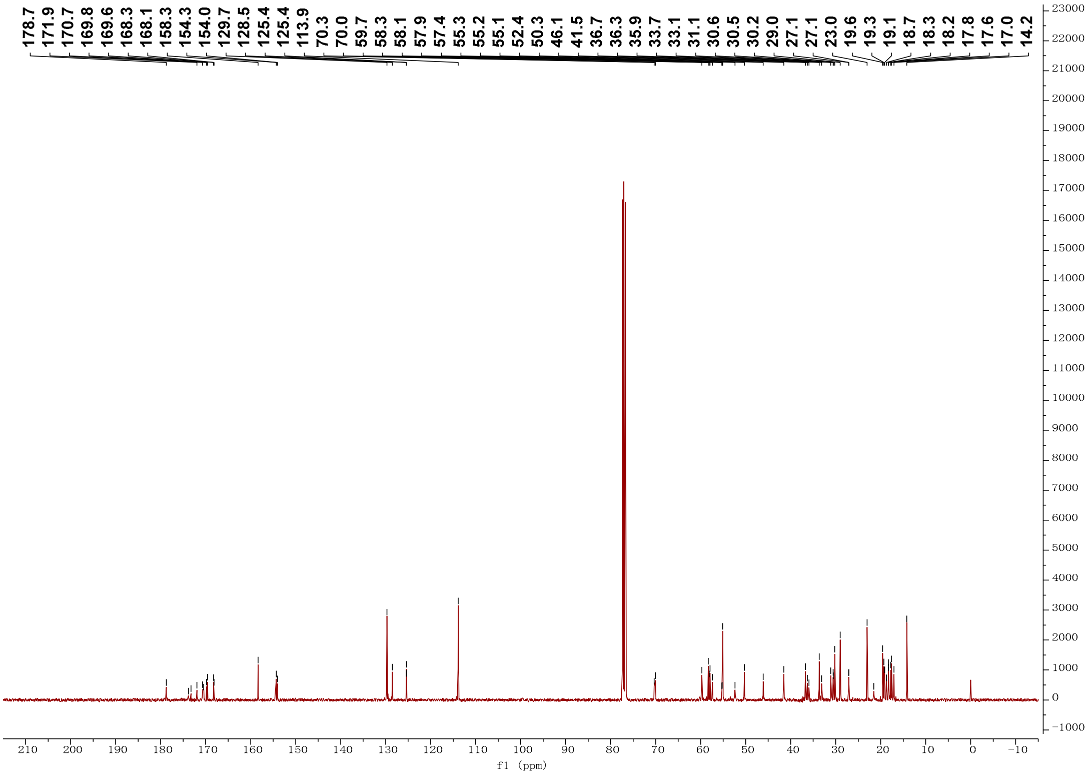

Figure S189. ^{13}C NMR spectrum of compound **16h** in $CDCl_3$ (100 MHz)

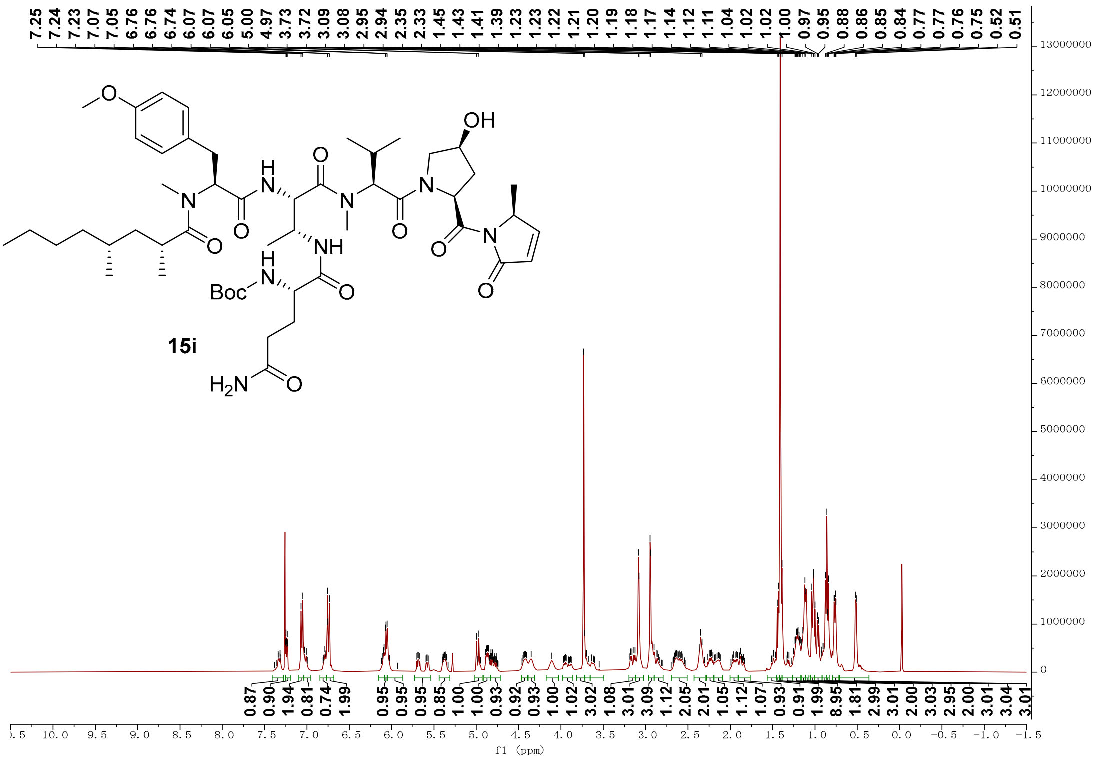

Figure S190. ^{1}H NMR spectrum of compound **15i** in $CDCl_3$ (400 MHz)

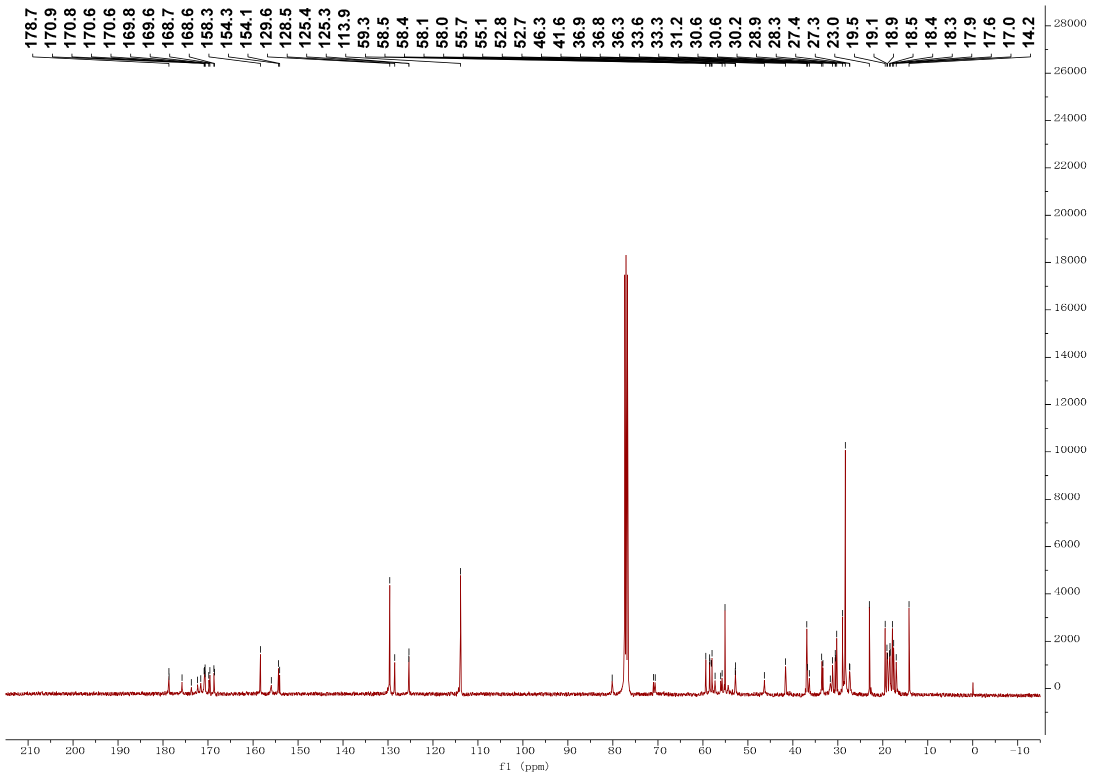

Figure S191. ^{13}C NMR spectrum of compound **15i** in $CDCl_3$ (100 MHz)

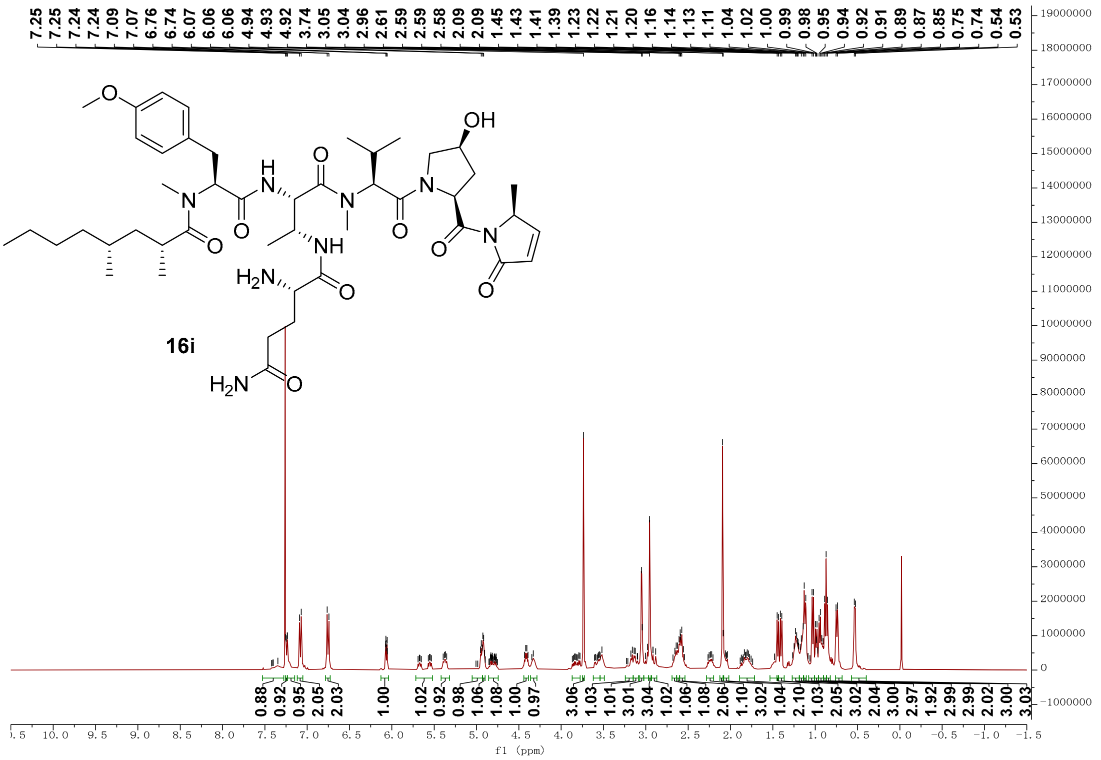

Figure S192. ^{1}H NMR spectrum of compound **16i** in $CDCl_3$ (400 MHz)

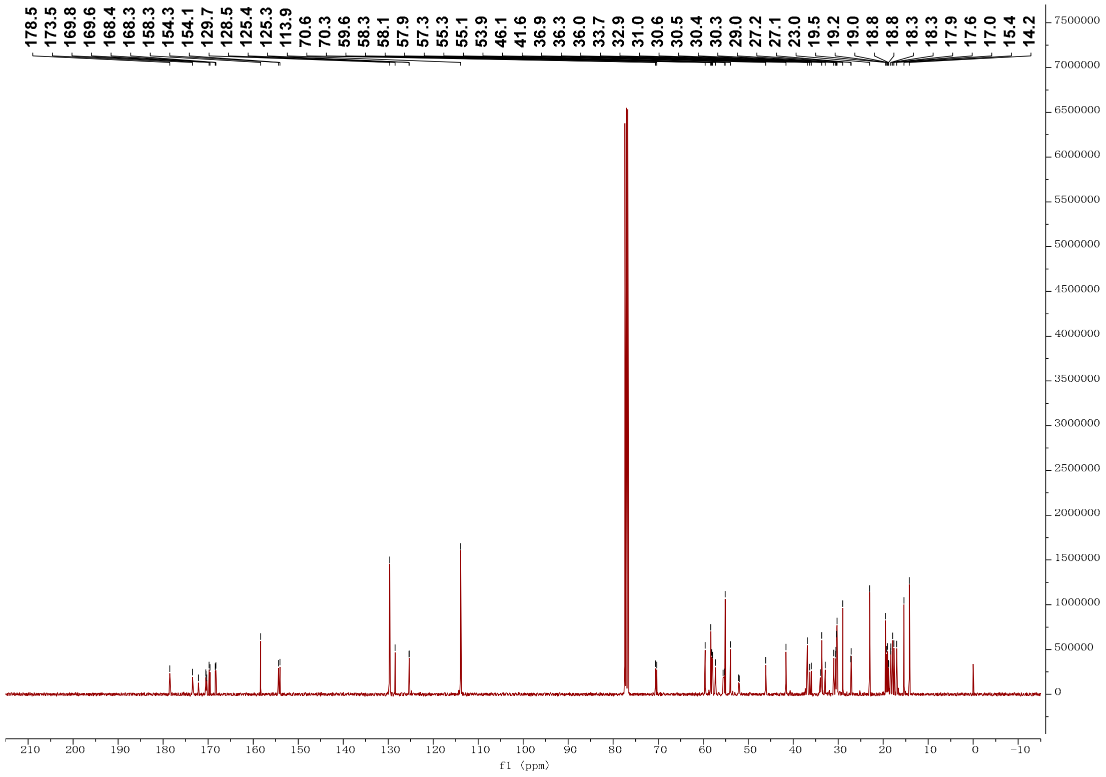

Figure S193. ^{13}C NMR spectrum of compound **16i** in $CDCl_3$ (100 MHz)

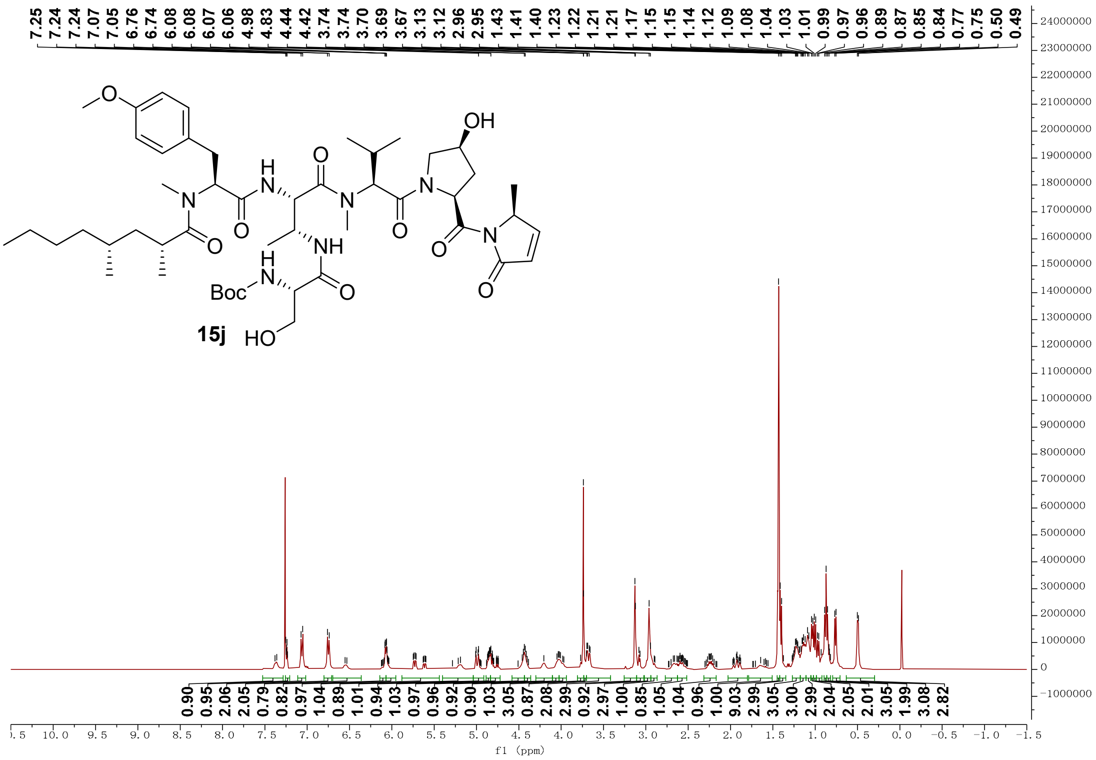

Figure S194. ^{1}H NMR spectrum of compound **15j** in $CDCl_3$ (400 MHz)

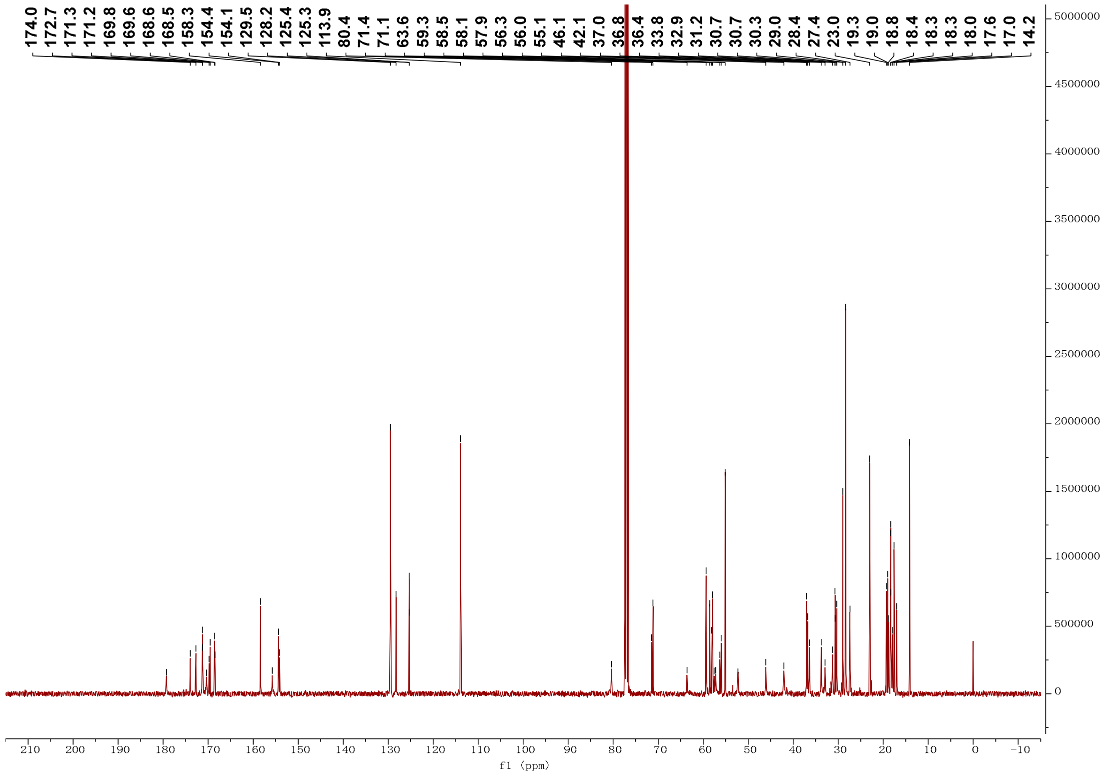

Figure S195. ^{13}C NMR spectrum of compound **15j** in $CDCl_3$ (100 MHz)

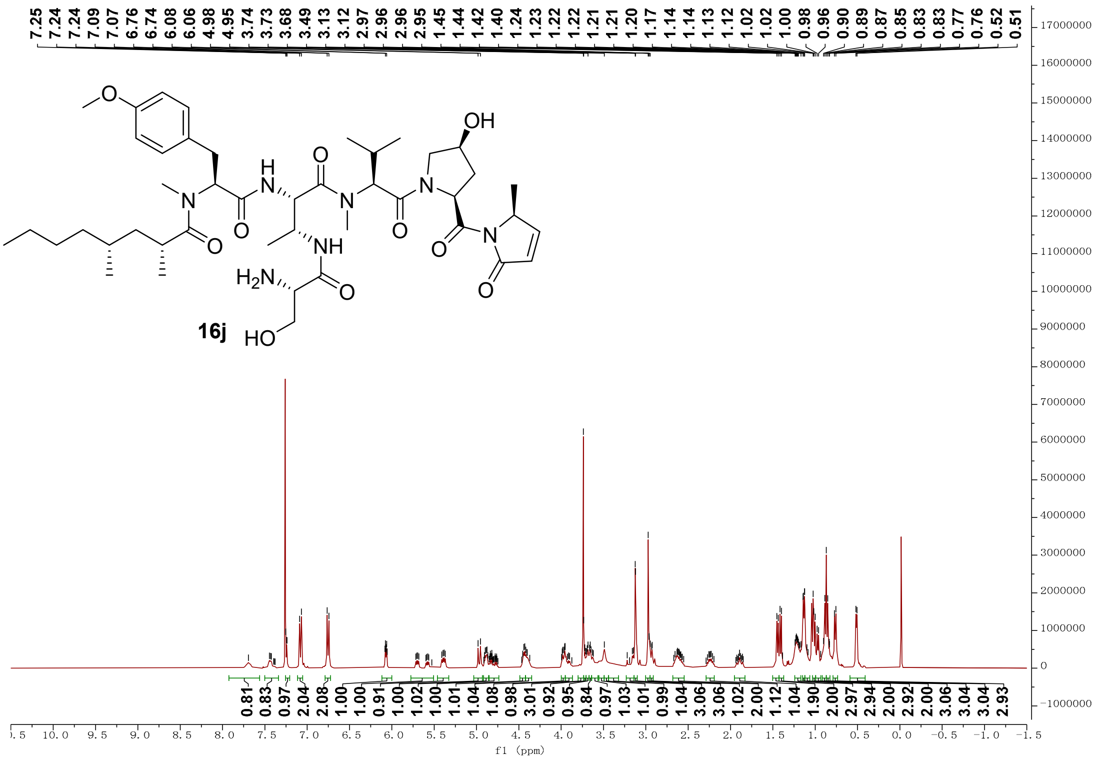

Figure S196. ^{1}H NMR spectrum of compound **16j** in $CDCl_3$ (400 MHz)

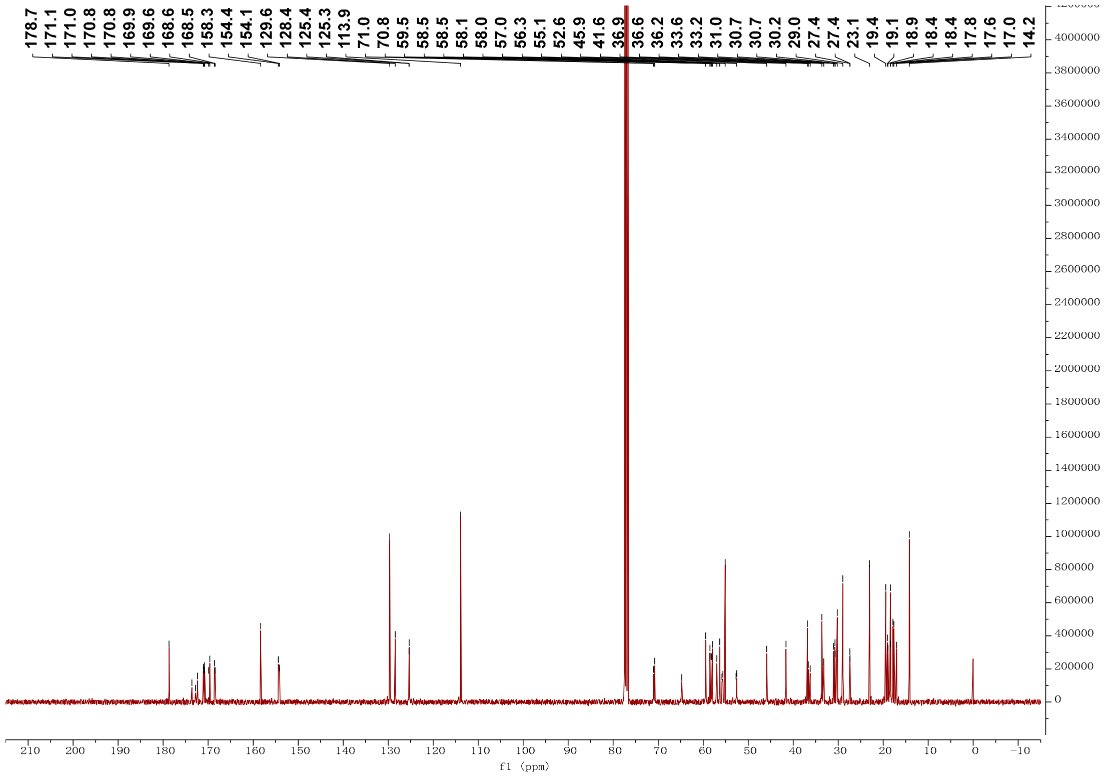

Figure S197. ^{13}C NMR spectrum of compound **16j** in $CDCl_3$ (100 MHz)

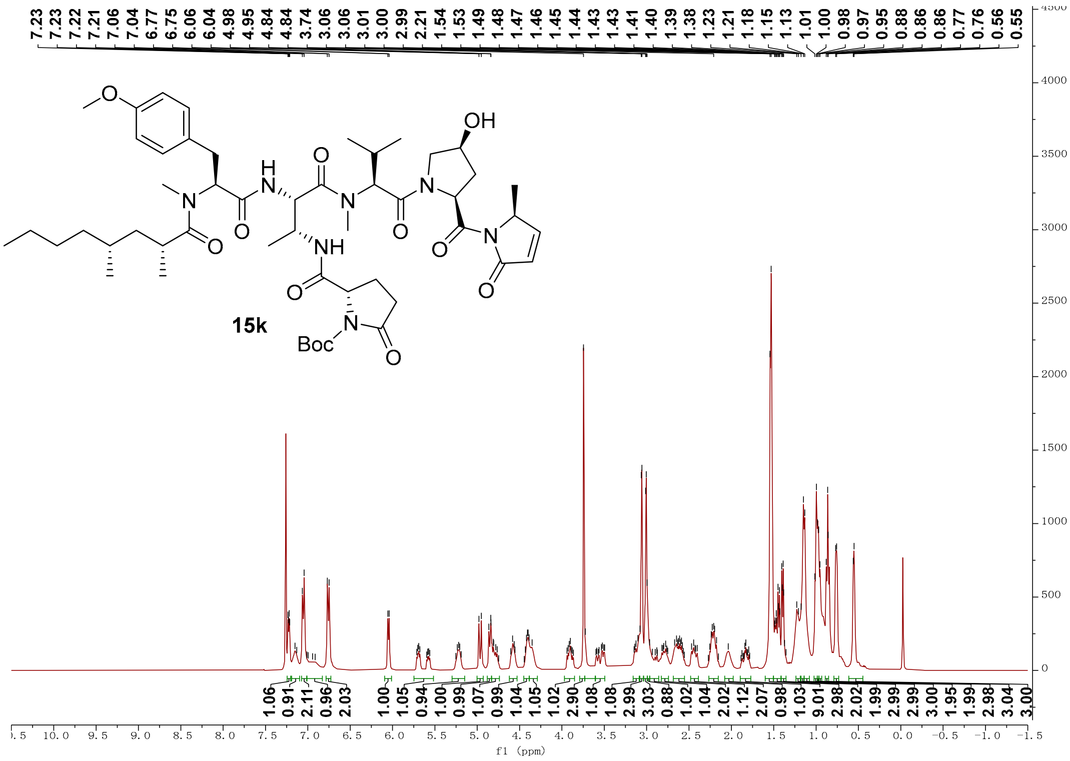

Figure S198. ^{1}H NMR spectrum of compound **15k** in $CDCl_3$ (400 MHz)

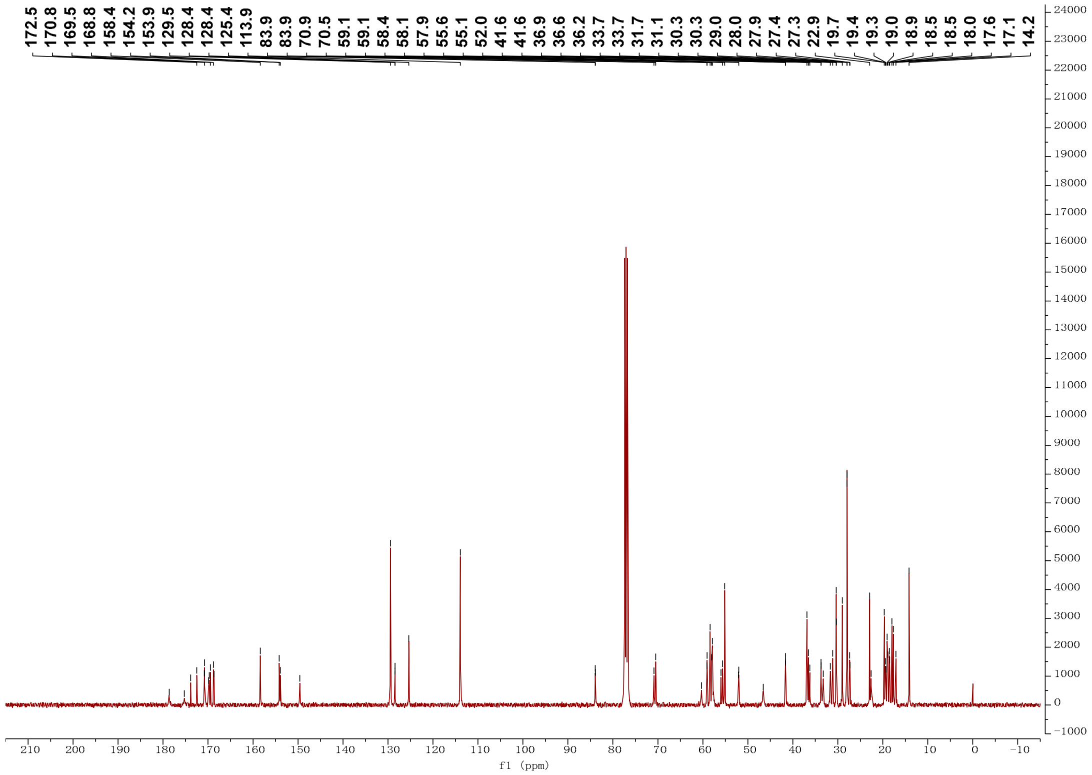

Figure S199. ^{13}C NMR spectrum of compound **15k** in $CDCl_3$ (100 MHz)

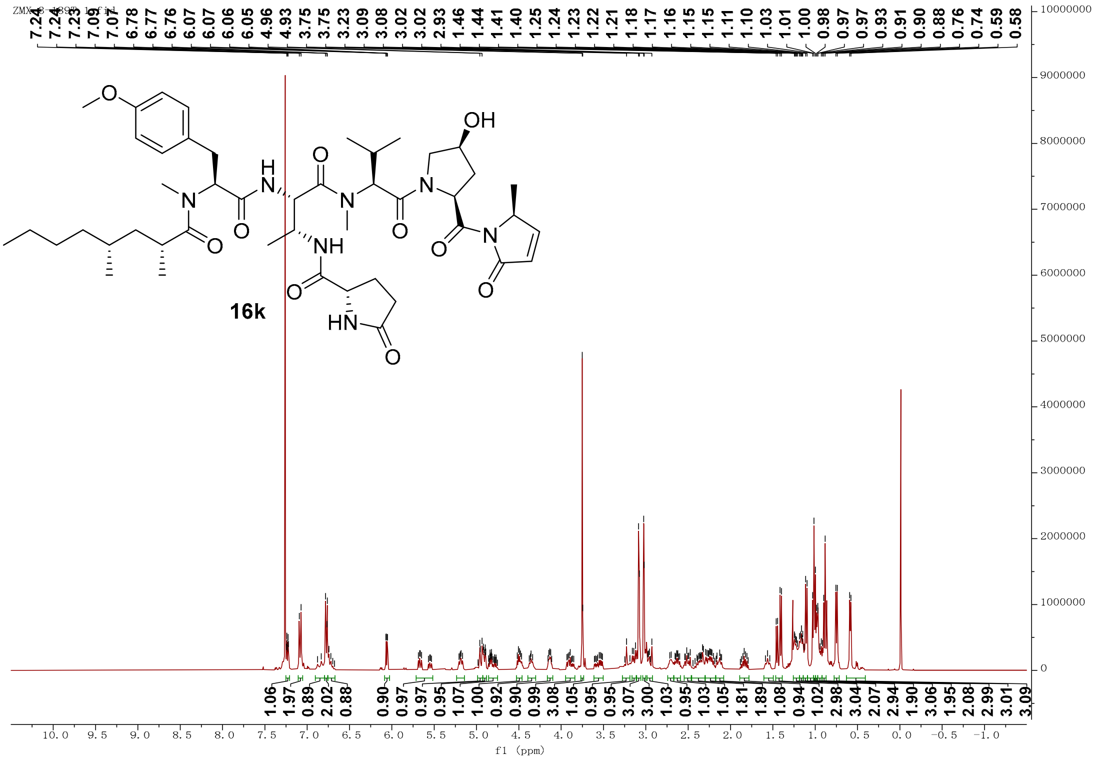

Figure S200. ^{1}H NMR spectrum of compound **16k** in $CDCl_3$ (400 MHz)

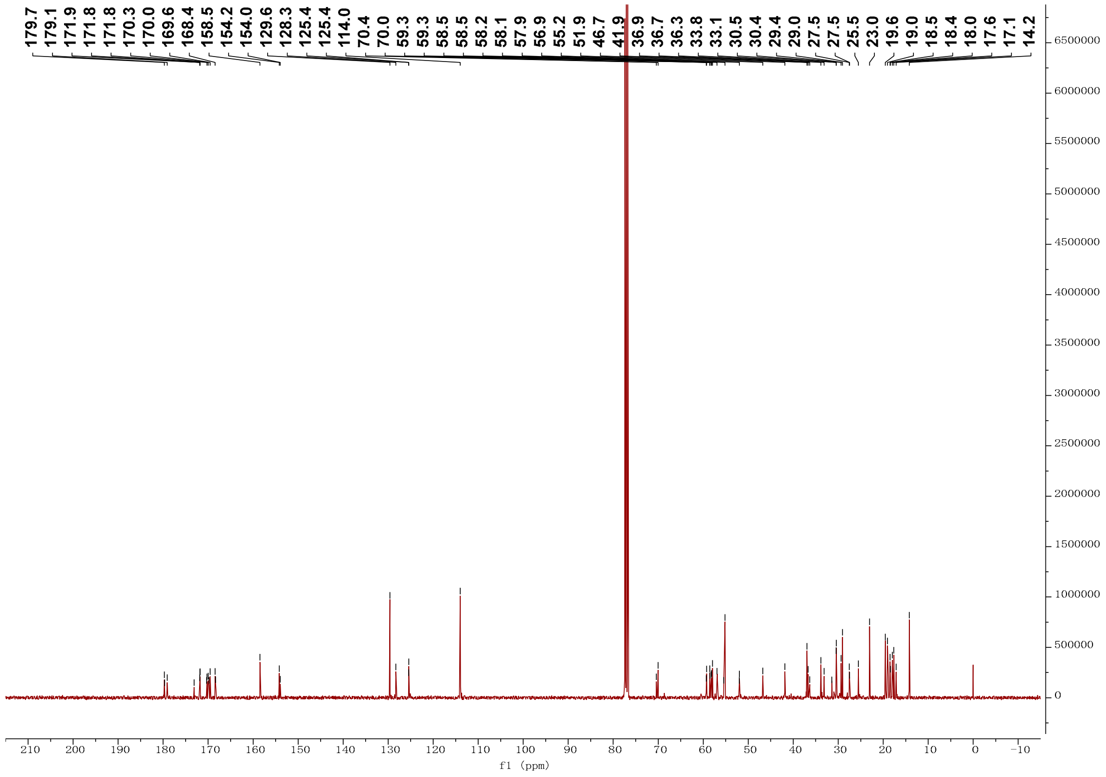

Figure S201. ^{13}C NMR spectrum of compound **16k** in $CDCl_3$ (100 MHz)

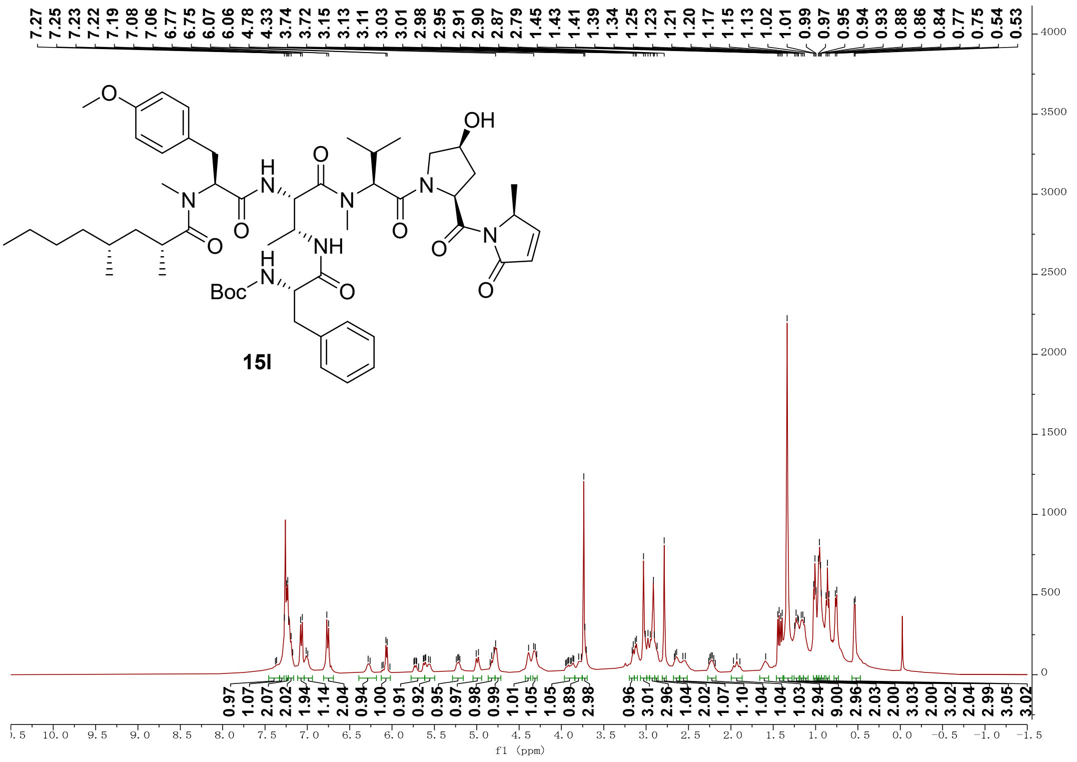

Figure S202. ^{1}H NMR spectrum of compound **15l** in $CDCl_3$ (400 MHz)

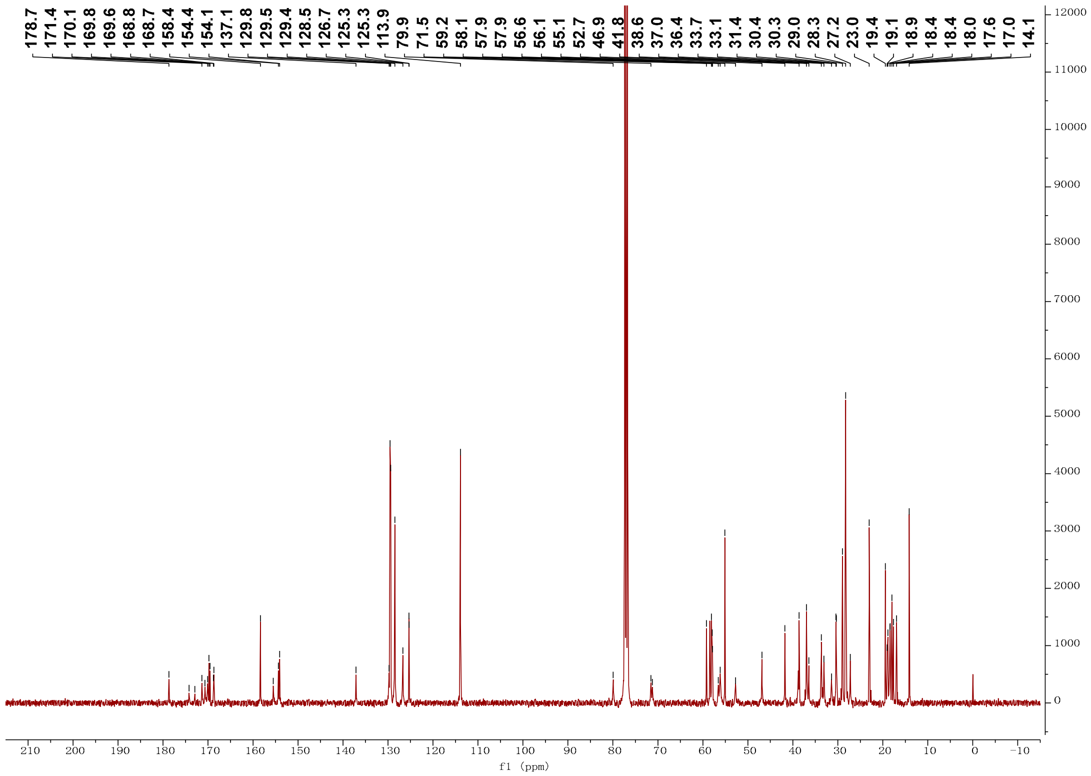

Figure S203. ^{13}C NMR spectrum of compound **15l** in $CDCl_3$ (100 MHz)

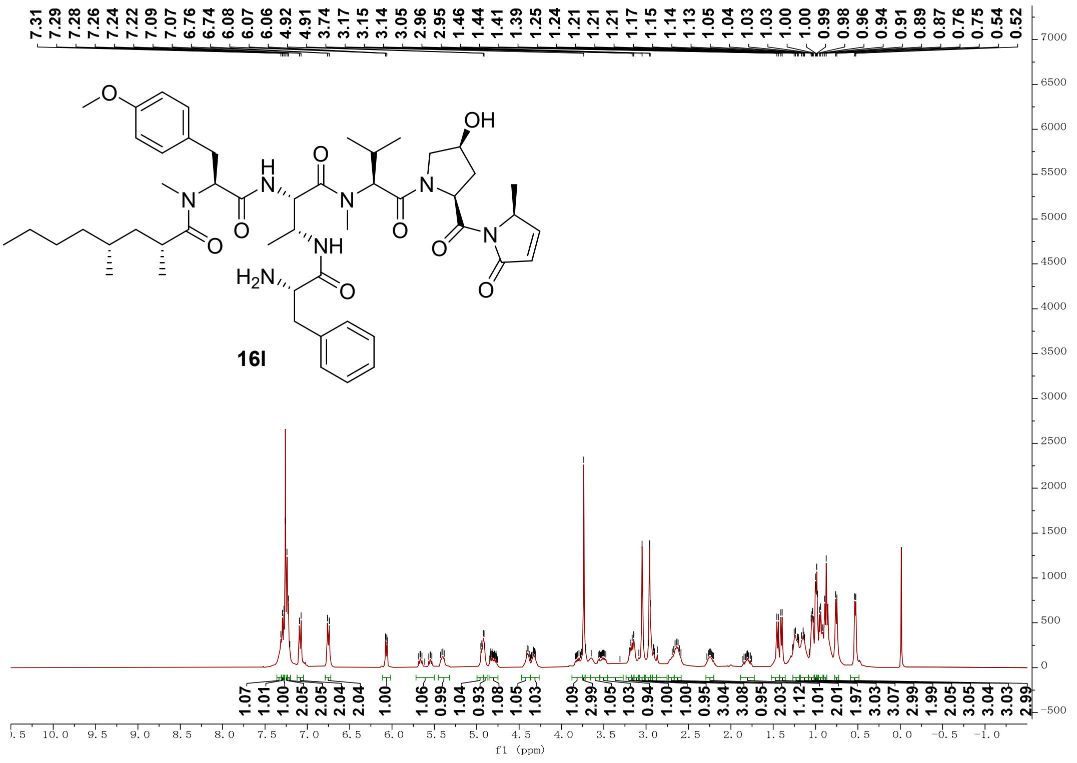

Figure S204. ^{1}H NMR spectrum of compound **16l** in $CDCl_3$ (400 MHz)

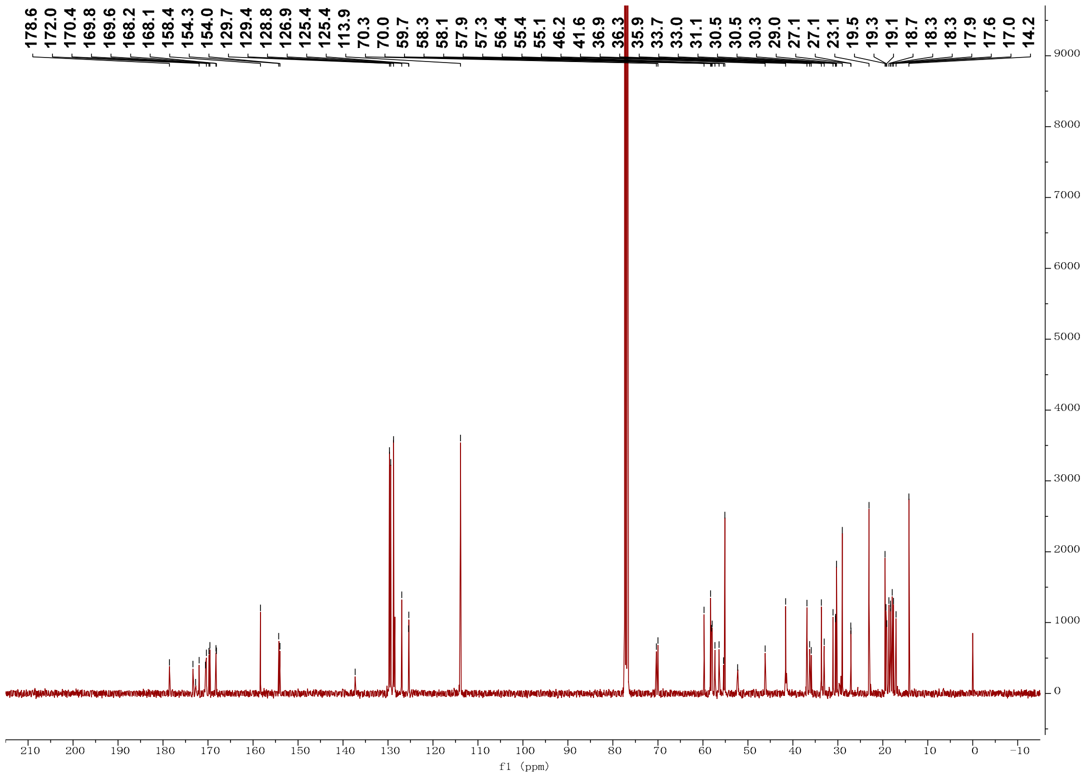

Figure S205. ^{13}C NMR spectrum of compound **16l** in $CDCl_3$ (100 MHz)

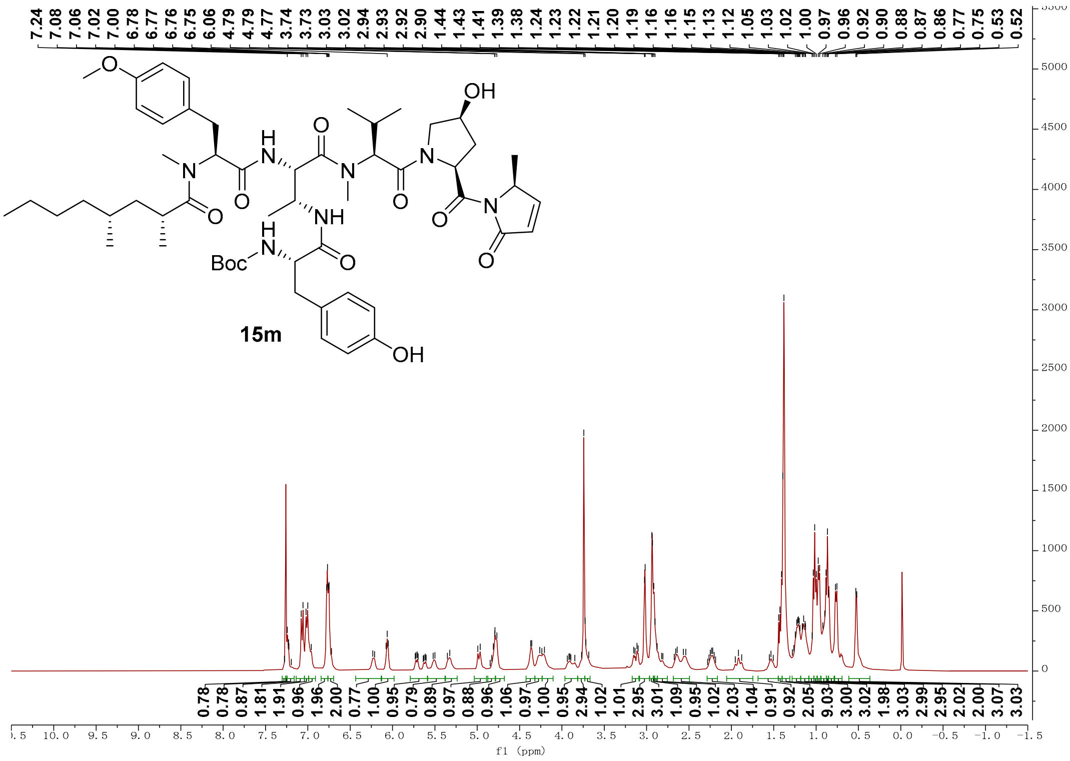

Figure S206. ¹H NMR spectrum of compound **15m** in $CDCl_3$ (400 MHz)

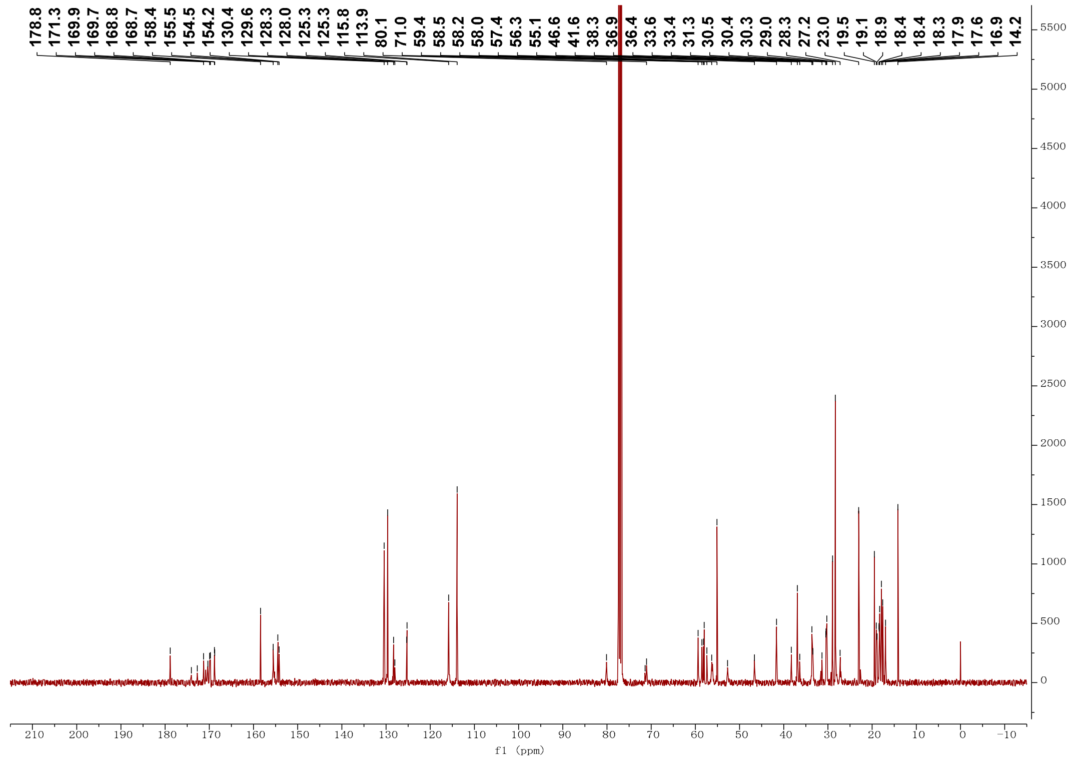

Figure S207. ¹³C NMR spectrum of compound **15m** in $CDCl_3$ (100 MHz)

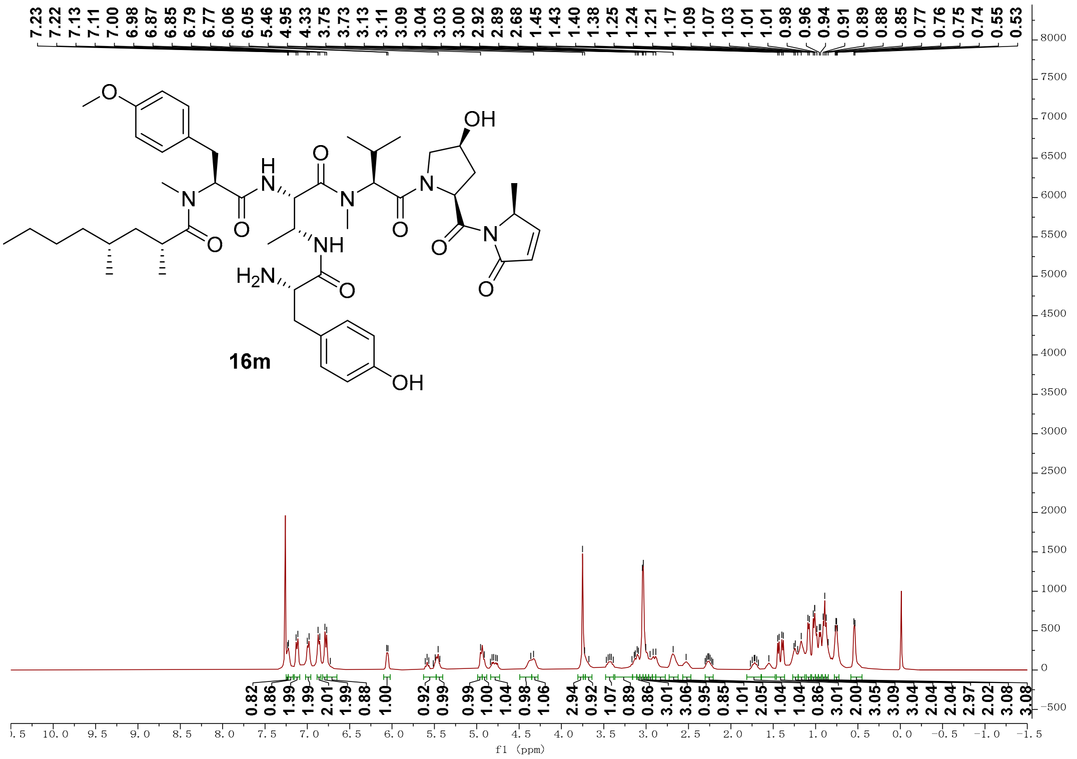

Figure S208. ^{1}H NMR spectrum of compound **16m** in $CDCl_3$ (400 MHz)

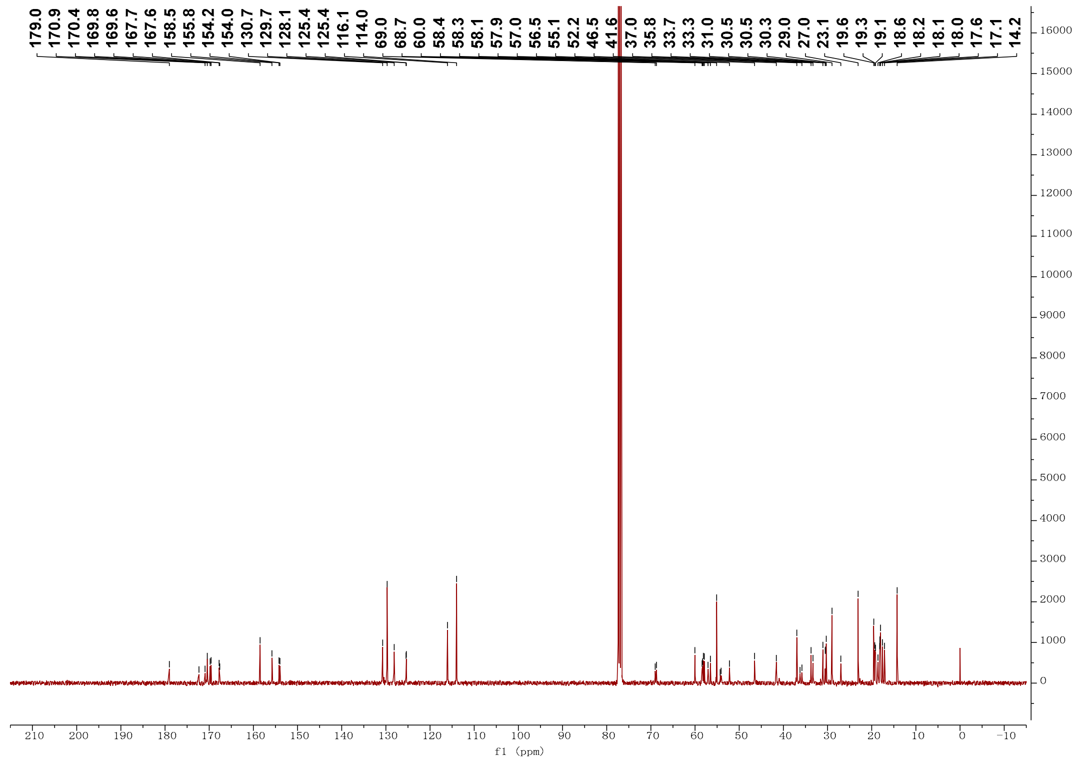

Figure S209. ^{13}C NMR spectrum of compound **16m** in $CDCl_3$ (100 MHz)

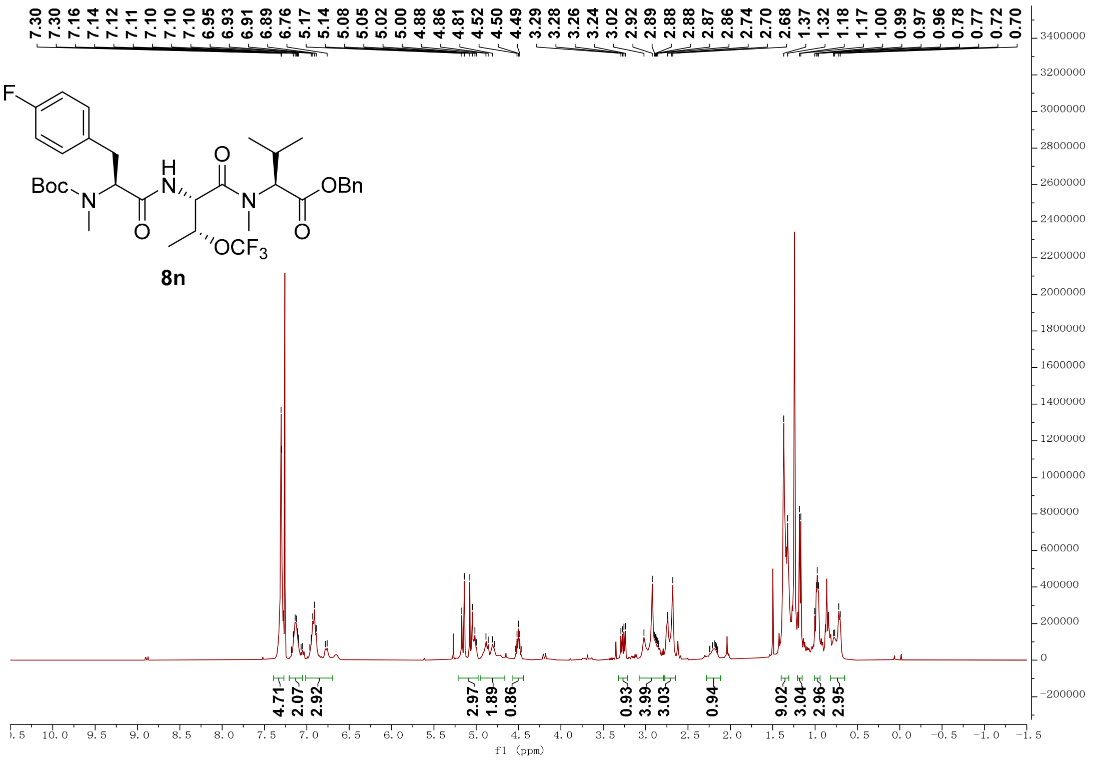

Figure S210. ^{1}H NMR spectrum of compound **8n** in $CDCl_3$ (400 MHz)

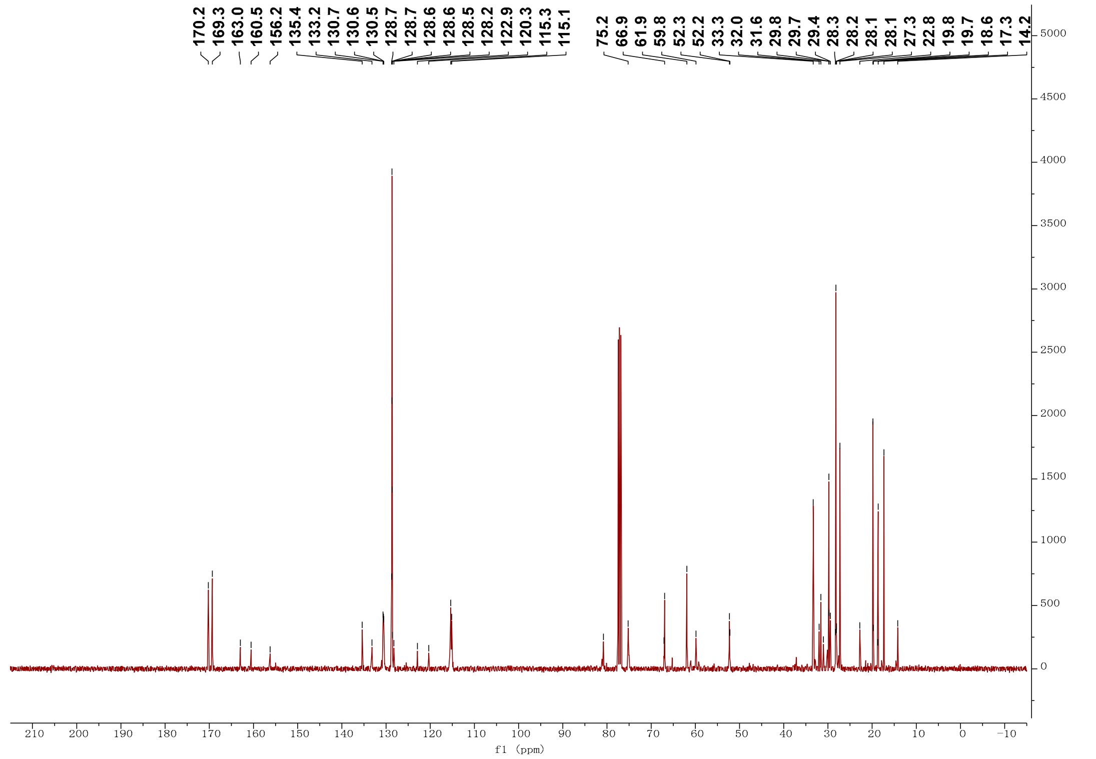

Figure S211. ^{13}C NMR spectrum of compound **8n** in $CDCl_3$ (100 MHz)

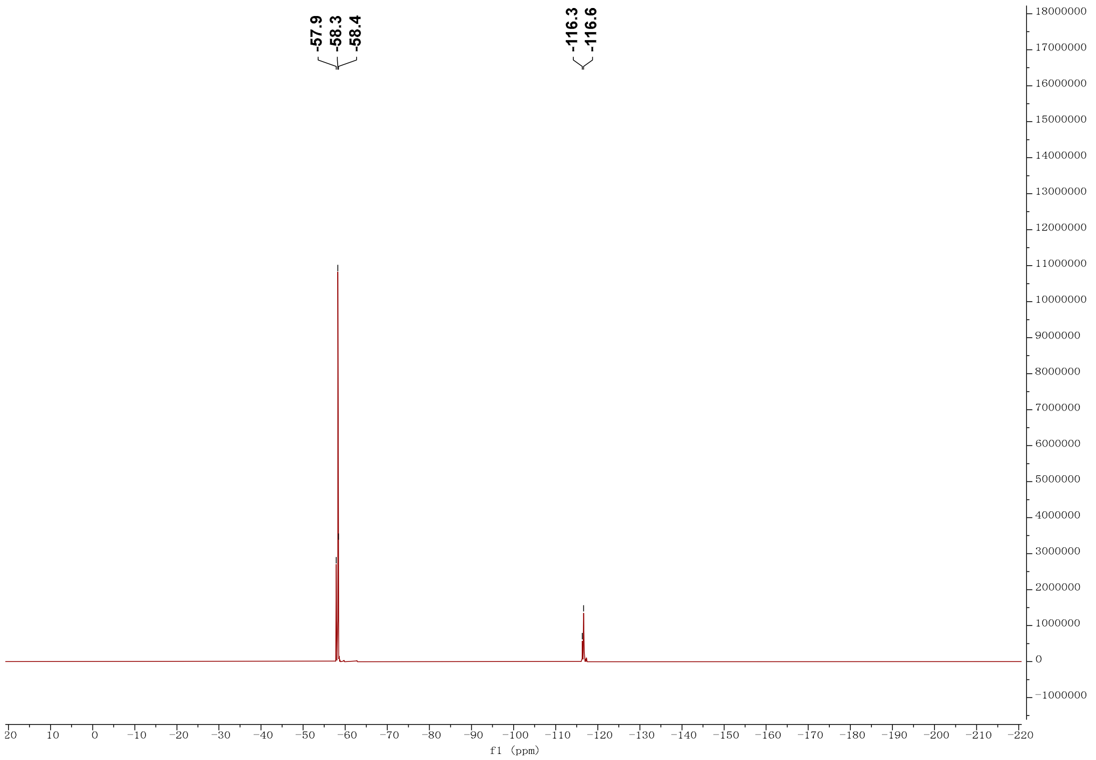

Figure S212. ^{19}F NMR spectrum of compound **8n** in $CDCl_3$ (376 MHz)

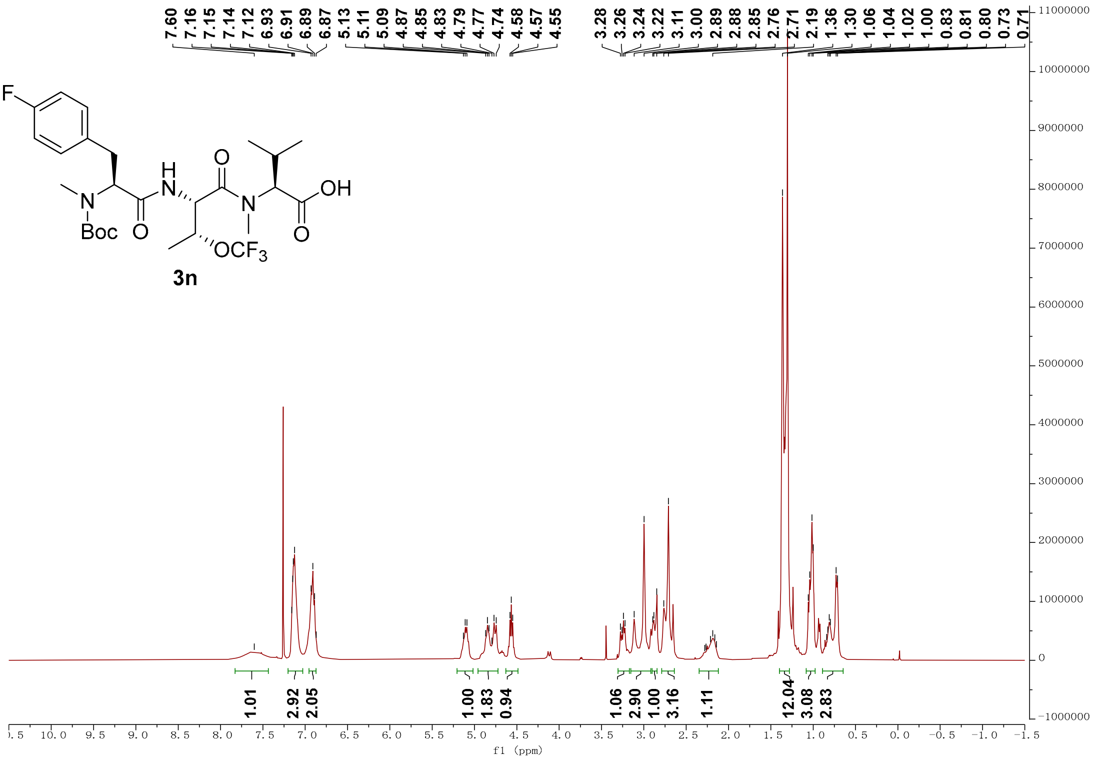

Figure S213. ^{1}H NMR spectrum of compound **3n** in $CDCl_3$ (400 MHz)

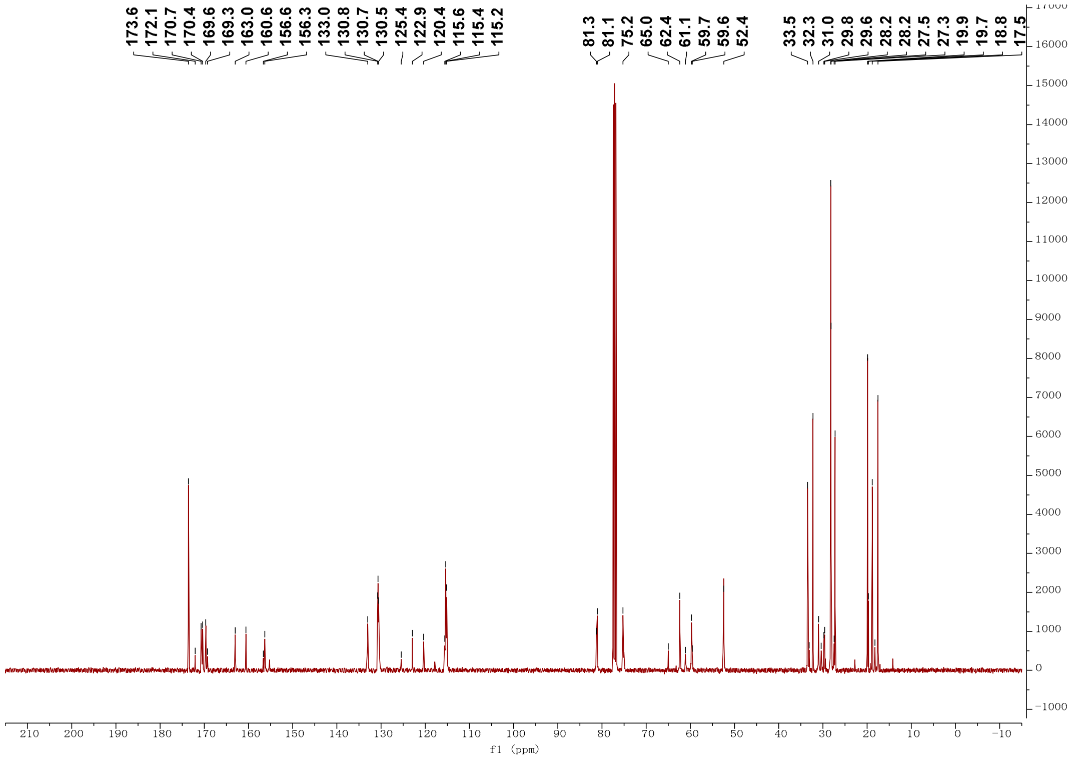

Figure S214. ^{13}C NMR spectrum of compound **3n** in $CDCl_3$ (100 MHz)

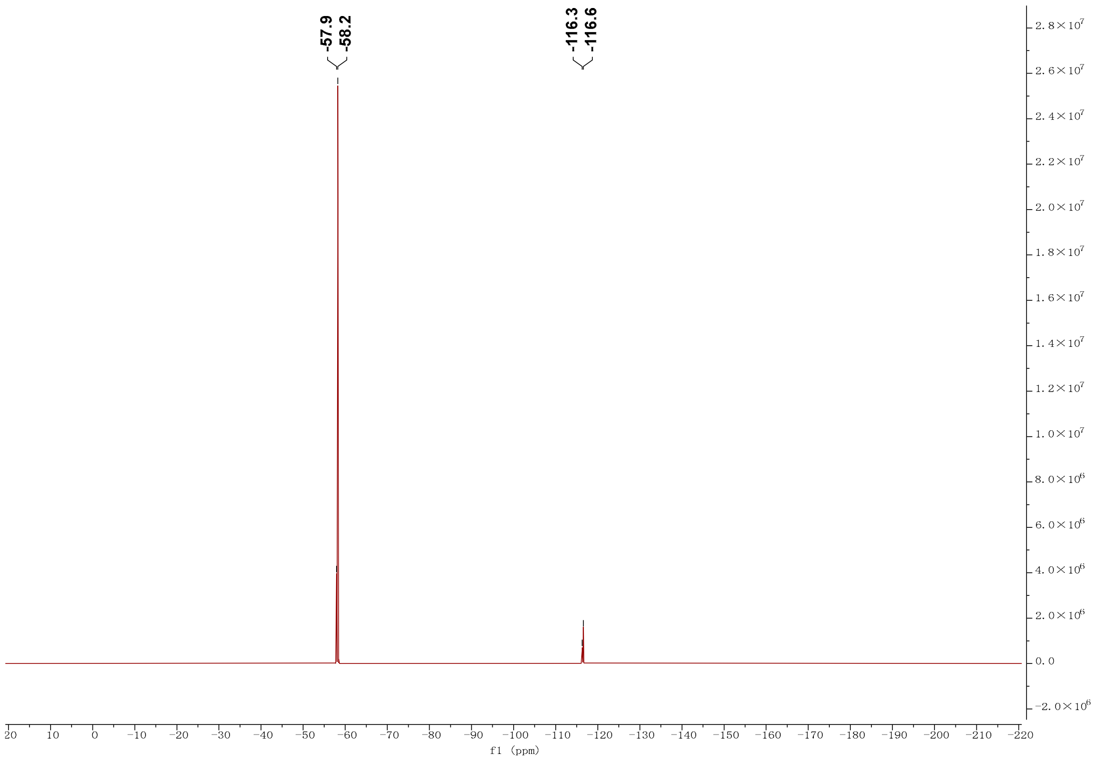

Figure S215. ^{19}F NMR spectrum of compound **3n** in $CDCl_3$ (376 MHz)

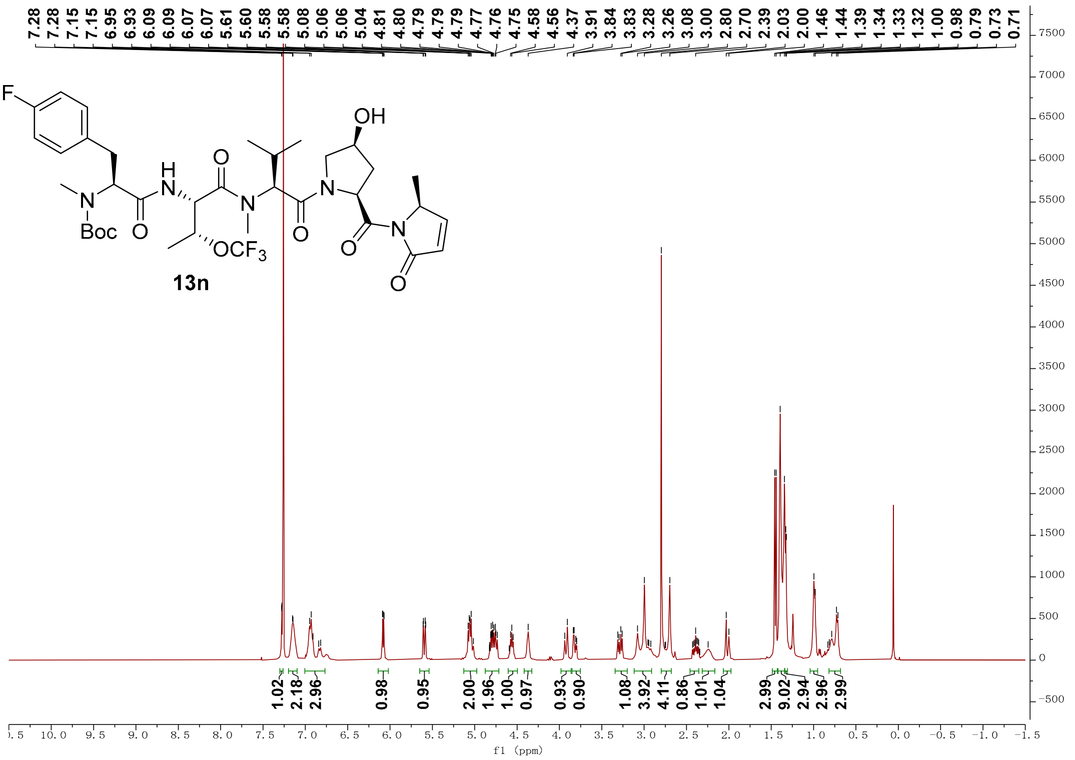

Figure S216. ^{1}H NMR spectrum of compound **13n** in $CDCl_3$ (400 MHz)

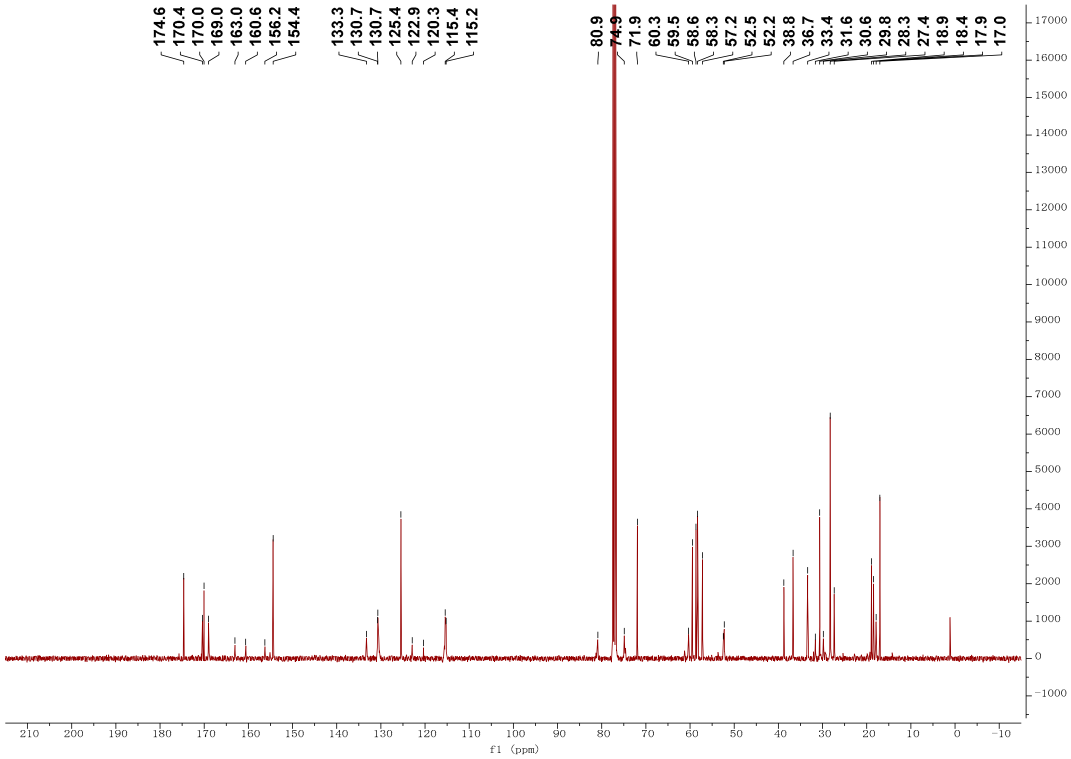

Figure S217. ^{13}C NMR spectrum of compound **13n** in $CDCl_3$ (100 MHz)

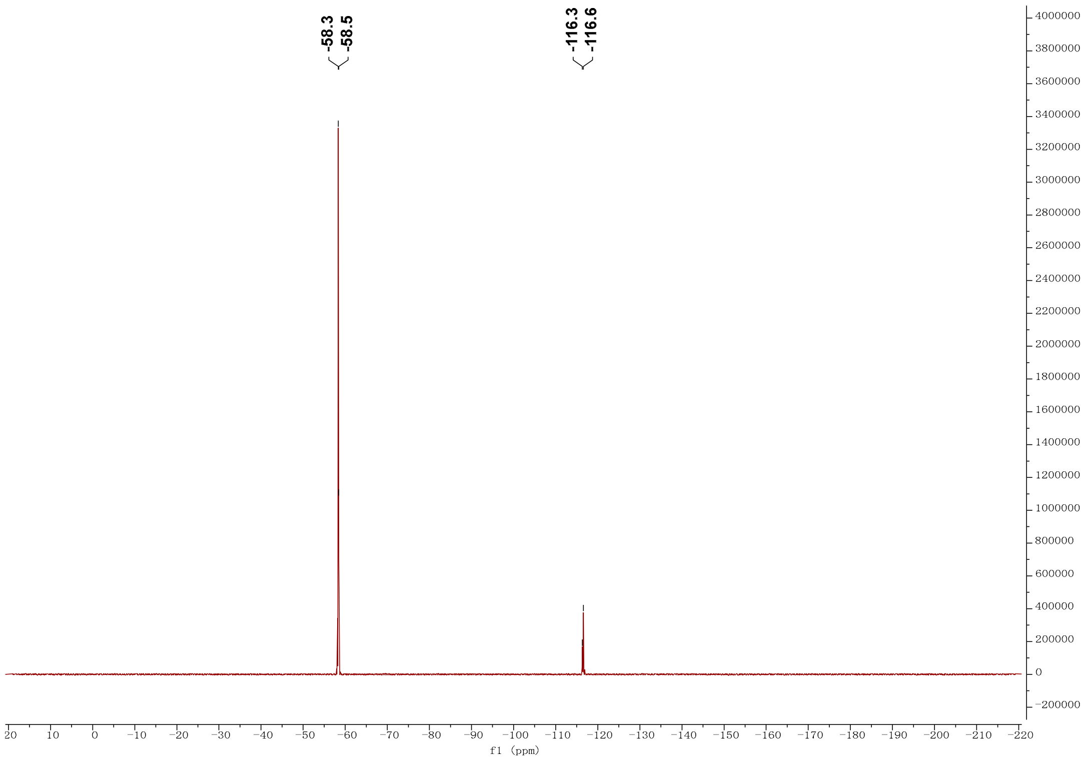

Figure S218. ^{19}F NMR spectrum of compound **13n** in $CDCl_3$ (376 MHz)

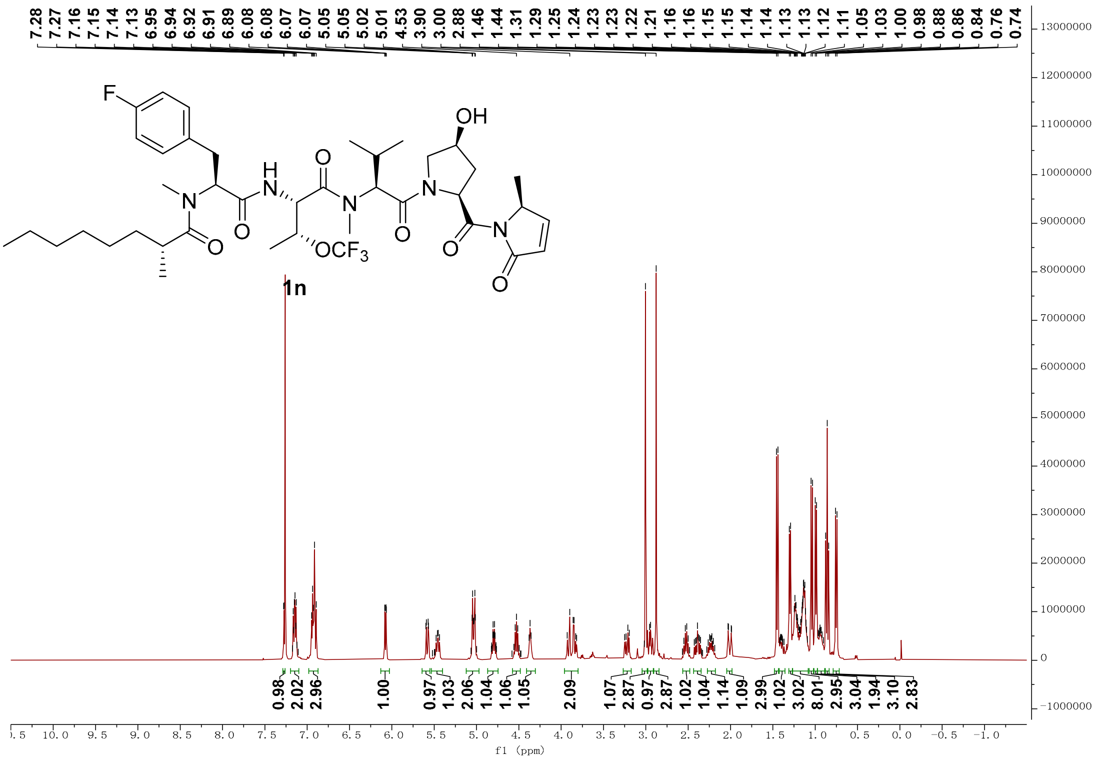

Figure S219. ^{1}H NMR spectrum of compound **1n** in $CDCl_3$ (400 MHz)

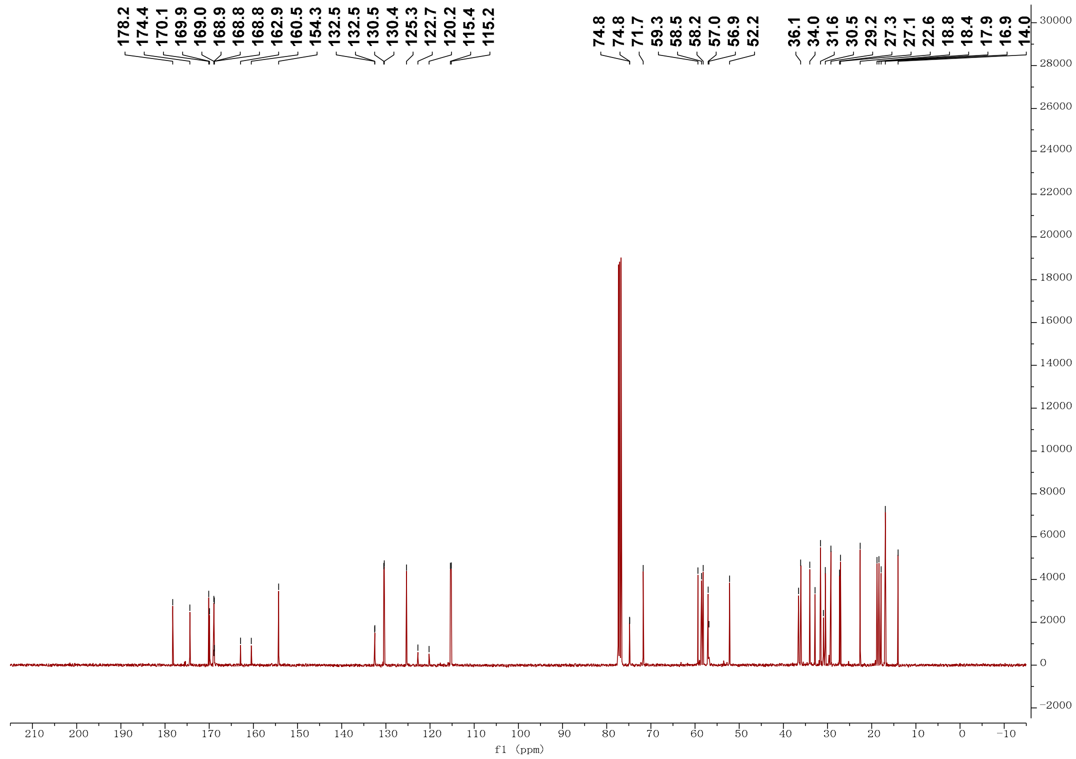

Figure S220. ^{13}C NMR spectrum of compound **1n** in $CDCl_3$ (100 MHz)

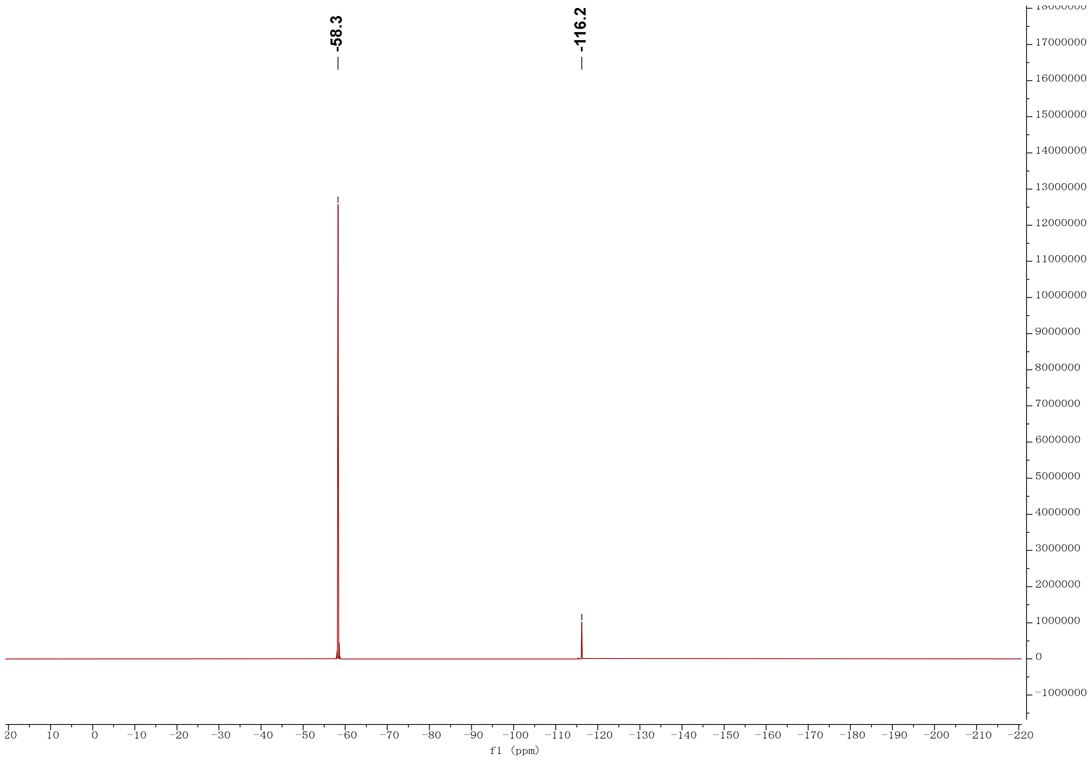

Figure S221. ^{19}F NMR spectrum of compound **1n** in $CDCl_3$ (376 MHz)

3. HPLC Analysis

Solvent A was a 0.1% phosphoric acid in 90% H_2O and 10% acetonitrile, and solvent B was a phosphoric in acid 90% acetonitrile and 10% H_2O.

The linear gradient was from 30 to 100% B over 15 min, and after 15 min was 100% B at a flow rate of 1.0 mL/min.

<Chromatogram>

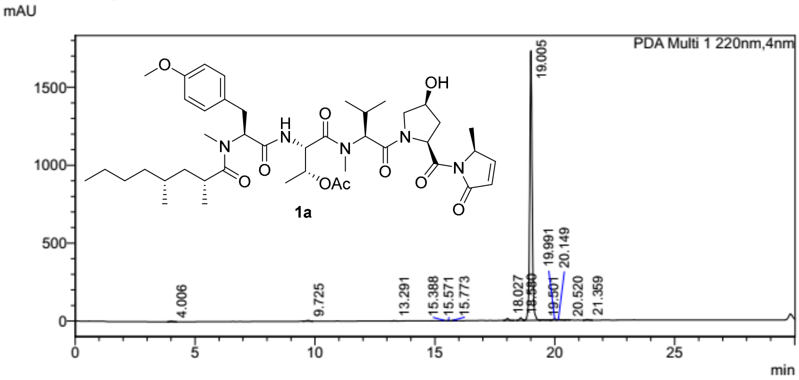

<Peak Table>

PDA Ch1 220nm

Peak#	Ret. Time	Area	Height	Conc.	Unit	Mark	Name
1	4.006	31429	3529	0.252			
2	9.725	49717	4838	0.398			
3	13.291	11465	1717	0.092			
4	15.388	7581	1411	0.061			
5	15.571	7675	1520	0.061			
6	15.773	21280	3402	0.170			
7	18.027	88187	12304	0.706			
8	18.580	127533	16863	1.021			
9	19.005	11949064	1730188	95.671		V	
10	19.501	47351	2747	0.379		V	
11	19.991	65972	9188	0.528		V	
12	20.149	40024	4736	0.320		V	
13	20.520	9799	1555	0.078			
14	21.359	32716	4059	0.262			
Total		12489795	1798055				

Figure S222. HPLC analysis of compound **1a**

<Chromatogram>

mAU

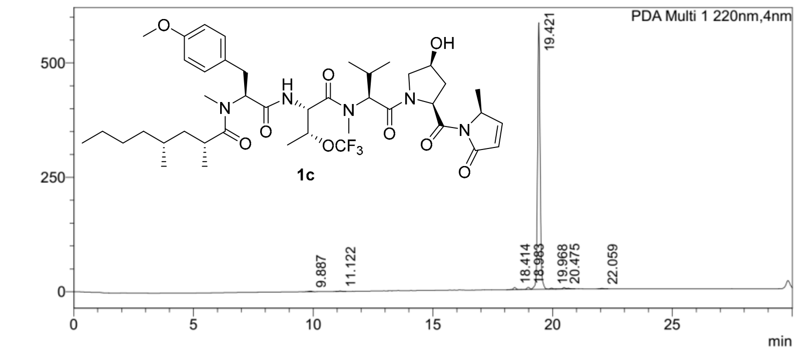

<Peak Table>

PDA Ch1 220nm

Peak#	Ret. Time	Area	Height	Conc.	Unit	Mark	Name
1	9.887	16498	1251	0.372			
2	11.122	8069	1043	0.182			
3	18.414	39211	4389	0.885			
4	18.983	39425	4414	0.890		V	
5	19.421	4252245	565586	96.011		V	
6	19.968	19827	1956	0.448		V	
7	20.475	42779	2819	0.966		V	
8	22.059	10882	1173	0.246			
Total		4428934	582632				

Figure S223. HPLC analysis of compound **1c**

<Chromatogram>

mAU

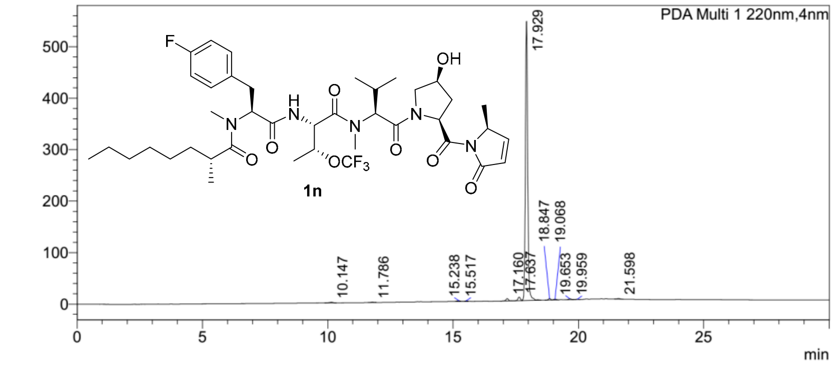

<Peak Table>

PDA Ch1 220nm

Peak#	Ret. Time	Area	Height	Conc.	Unit	Mark	Name
1	10.147	15929	1372	0.436			
2	11.786	5461	682	0.150			
3	15.238	14582	1513	0.399		V	
4	15.517	7597	1061	0.208		V	
5	17.160	31883	4559	0.873			
6	17.637	47664	6998	1.305		V	
7	17.929	3462597	542323	94.822		SV	
8	18.847	21546	3133	0.590			
9	19.068	16651	1743	0.456		V	
10	19.653	12702	1698	0.348			
11	19.959	6997	690	0.192		V	
12	21.598	8055	1079	0.221			
Total		3651664	566850				

Figure S224. HPLC analysis of compound **1n**

4. Biological experiment

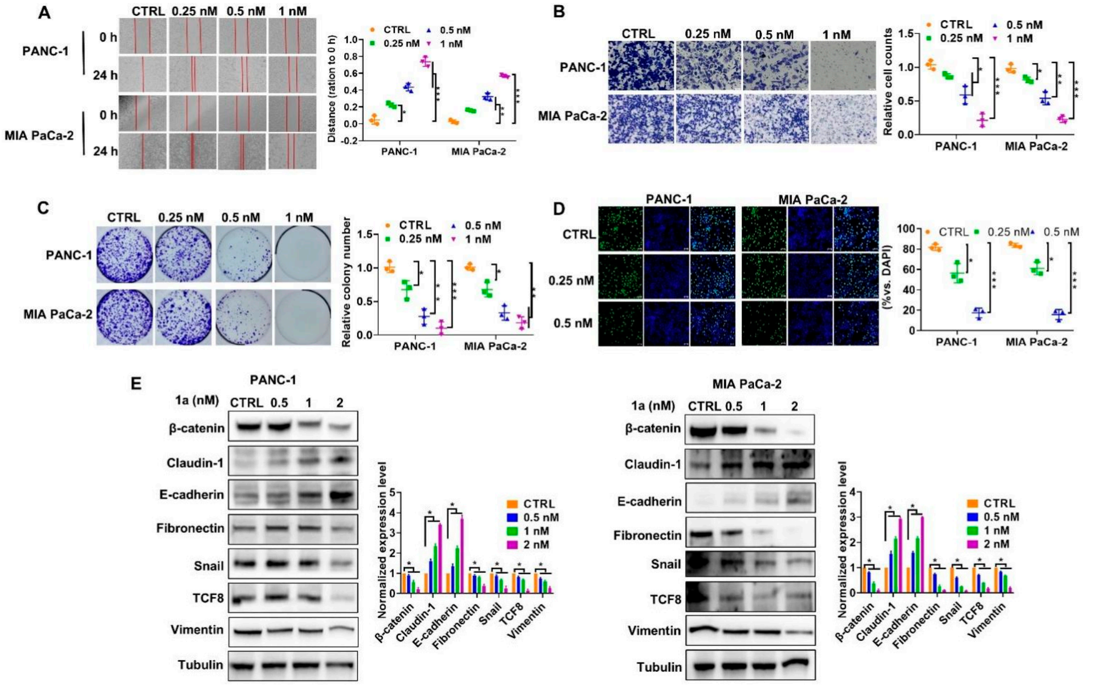

Figure S225. Compound **1a** inhibited the migration, invasion and proliferation of pancreatic cancer cells. (A) The results of wound healing assays to investigate the effects of **1a** on the migration ability against PANC-1 and MIA PaCa-2 pancreatic cancer cells. (B) The results of transwell assays to investigate the effects of **1a** on the invasion ability. (C) Effects of **1a** on the clonogenic ability. (D) EdU assays of **1a**. (E) Western blot assays of **1a** on biomarkers. Data are presented as mean ± SD, n = 3, **p* < 0.05, ***p* < 0.01, ****p* < 0.001.

Approximately 50% of pancreatic cancer patients show distant metastases at the time of diagnosis. More effective chemotherapies are highly needed for patients with metastatic disease. To evaluate the inhibitory effects of majusculamide D (**1a**) on the migratory capabilities of pancreatic cancer cells (PANC-1 and MIA PaCa-2), wound healing assays were performed (Figure S1A). The results showed that treatment with compound **1a** significantly reduced the scratch wound healing rate compared to the control group in a concentration-dependent manner. These findings indicated that compound **1a** effectively inhibited the migration of these pancreatic cancer cells. Further validation with transwell assays confirmed that compound **1a** significantly decreased cell invasion in a concentration-dependent manner compared to the control group (Figure S1B). Since cell migration and invasion are correlated with cancer metastasis, compound **1a** may delay the progression of tumors' local spread and distant metastasis by inhibiting the migration and invasion ability of pancreatic cancer cells.

The results of the colony formation assay showed that treatment with compound **1a** remarkably reduced colony formation compared to the control group in a concentration-dependent manner (Figure S1C). These findings suggested that compound **1a** inhibited the growth and survival of pancreatic cancer cells.

The EdU (5-ethyl-2'-deoxyuridine) assay is a well-established method that assesses cell proliferation activity by detecting DNA synthesis. The results of EdU assays indicated that the cell proliferation ability of the compound **1a-**treated group was significantly decreased in comparison to the control group (Figure S1D), which was also in a concentration-dependent manner. These results suggested that **1a** could effectively reduce the DNA synthesis of pancreatic cancer cells, thereby inhibiting the proliferation of pancreatic cancer cells, which was consistent with the results of clone formation assays.

It is well established that the metastasis of tumors was accompanied by the change of several special proteins. β-catenin is an intracellular signaling molecule primarily involved in the Wnt signaling pathway and regulates cell proliferation and differentiation. The abnormal activation of β-catenin is associated with tumor development and increased invasiveness [1-2]. Claudin-1 belongs to the tight junction protein family, which maintains the barrier function between cells and is correlated with some cancers' metastatic ability [3-4]. E-cadherin is an intercellular adhesion molecule, and its decreased expression is common in the phenotype transition process of cancer cells, such as epithelial-mesenchymal transition (EMT), which is a key step in cancer metastasis [5]. Fibronectin is an extracellular matrix protein involved in cell adhesion and migration, and its expression is often positively associated with cancer's metastatic ability [6]. Snail and TCF8/ZEB1 both are transcription factors that can inhibit the expression of E-cadherin and promote the EMT process [5]. Vimentin is a marker protein of mesenchymal cells, and its expression increases during EMT, further contribute to the process of metastasis by supporting the structural changes needed for cell migration and invasion [7].

We examined the effects of compound **1a** on above key proteins involved in tumor progression pathways employing Western blot analysis in PANC-1 cells and MIA PaCa-2 cells (Figure S1E). The results showed that the expression levels of Claudin-1 and E-cadherin increased with rising concentrations of compound **1a**, suggesting that compound **1a** might inhibit cancer cell invasion and migration by enhancing cell-cell adhesion and barrier function. Conversely, the expression levels of β-catenin, Fibronectin, Snail,

TCF8/ZEB1, and Vimentin decreased with increasing **1a** concentrations. These findings indicate that majusculamide D may inhibit the invasion and migration potential of pancreatic cancer cells by suppressing the Wnt signaling pathway and the EMT process.

All the findings above highlight the value of majusculamide D as a potential lead for treating pancreatic cancer, especially in inhibiting the metastasis and proliferation of pancreatic cancer.

4.1 Wound healing assay

A total of 3×10^5 cells were resuspended in 2 mL of culture medium and added to a 6-well plate. Once the cells had grown to confluence and covered the bottom of the wells, the medium was discarded. A straight line was scratched across the center of each well using a 200 μL pipette tip. Then washed with PBS to remove any floating cells. Fresh serum-free medium containing the compound was added to each well (2 mL per well). Images were taken at 0 hour and 24 hours after treatment to record and analyzed by measuring the area of the wound at each time point using ImageJ.

4.2. Transwell invasion assay

Matrigel (Corning, USA) was thawed at 4°C and diluted on ice by mixing it with the appropriate serum-free medium at a 1:1 ratio. After diluted matrigel (50 μL per insert) was evenly spread across the surface of the inserts and then incubated for 30 minutes. Cells were resuspended in serum-free medium containing the compound at a concentration of 2.5×10^5 cells/mL. Then, 200 μL of the cell suspension was added to the upper chamber of each insert, while 700 μL of complete medium was added to the lower chamber of each well. After incubation for 24 hours, the medium was discard, and the Matrigel was gently wiped off with a cotton swab. The inserts were washed three times with PBS, fixed with pre-cooled methanol (-20°C) for 15 minutes, and then washed three times with PBS. The cells were stained with crystal violet for 30 minutes, followed by PBS washes. The inserts were air-dried in the dark. The membranes at the bottom of the inserts were carefully removed with a scalpel, mounted on slides with neutral resin, and stored for imaging and analysis.

4.3. Clone formation assay

Cells were resuspended at a concentration of 300 cells/mL, and 2 mL of the suspension was added to each well of a 6-well plate. After the cells adhered to the plate, the compound was added, and the cells were incubated for 48 hours, followed by replacement with fresh medium. The cells were then cultured for an additional 10 days. Subsequently, the cells were fixed with methanol and stained with crystal violet. The colonies were imaged under a microscope and quantified using ImageJ.

4.4. EdU assay

The BeyoClick™ EdU-488 Cell Proliferation Assay Kit (Beyotime Biotechnology, China) was used according to the manufacturer's instructions. Cells were seeded in confocal dishes and allowed to adhere before being treated with the compound for 48 hours. EdU working solution was then added to achieve a final concentration of 10 μM, followed by a 2-hour incubation. After EdU incorporation, the medium was removed, and cells were fixed with 1 mL of 4% paraformaldehyde at room temperature for 15 minutes. Cells were washed three times with 1 mL of washing buffer, each wash lasting 5 minutes. Permeabilization was performed using 1 mL of 0.3% Triton X-100 in PBS for 10 minutes. After two additional washes, the Click reaction solution was prepared and added to the cells, followed by incubation in the dark for 30 minutes. The cells were then washed three times, and the nuclei were stained with DAPI in an anti-fade mounting medium. Images were captured using a confocal microscope.

4.5. Western blot assay

Cells were lysed in RIPA lysis buffer (Solarbio, China). The protein concentration was determined using the BCA assay. Cell lysates were mixed with loading buffer and boiled for 8 minutes to denature the proteins. Equal amounts of protein samples were separated by SDS-PAGE and then transferred onto a PVDF membrane. Following protein transfer, the membrane was blocked with 5% non-fat dry milk in TBST (Tris-buffered saline with 0.1% Tween 20) for 2 hours at room temperature to prevent non-specific binding. The membrane was then incubated overnight at 4°C with the appropriate primary antibodies, including anti-β-catenin (51067-2-AP, Proteintech), Claudin-1 (13050-1-AP, Proteintech), E-cadherin (20874-1-AP, Proteintech), Fibronectin (15613-1-AP, Proteintech), Snail (13099-1-AP, Proteintech), TCF8/ZEB1 (21544-1-AP, Proteintech), Vimentin (60330-1-Ig, Proteintech), α-Tubulin (11224-1-AP, Proteintech), GAPDH (60004-1-Ig, Proteintech),

Hexokinase-2 (22029-1-AP, Proteintech), MCT4 (22787-1-AP, Proteintech), PFKFB3 (13763-1-AP, Proteintech), PKM (10078-2-AP, Proteintech), MPC-1 (42898, Signalway), and LCMT1 (sc-365221, Santa Cruz Biotechnology). After primary antibody incubation, the membranes were washed three times with TBST for 5 minutes each. The membranes were then incubated with HRP-conjugated goat anti-rabbit or goat anti-mouse secondary antibodies for 1 hour at room temperature. Following secondary antibody incubation, the membranes were washed again three times with TBST. Protein bands were detected using an enhanced chemiluminescence (ECL) detection system and visualized using an imaging system.

5. References

[1] Wang, Z.; Li, Z.; Ji, H. Direct targeting of *β*-catenin in the Wnt signaling pathway: current progress and perspectives. *Med Res Rev.* **2021**, *41*, 2109–2129.

[2] Valenta, T.; Hausmann, G.; Basler, K. The many faces and functions of β-catenin. *EMBO J.* **2012**, *31*, 2714–2736.

[3] Liu, M.; Yang, J.; Zhang, Y.; Zhou, Z.; Cui, X.; Zhang, L.; Fung, K.-M.; Zheng, W.; Allard, F. D.; Yee, E. U.; Ding, K.; Wu, H.; Liang, Z.; Zheng, L., Fernandez-Zapico, M. E.; Li, Y.-P.; Bronze, M. S.; Morris, K. T.; Postier, R. G.; Houchen, C.W.; Yang, J.; Li, M. ZIP4 promotes pancreatic cancer progression by repressing ZO-1 and Claudin-1 through a ZEB1-dependent transcriptional mechanism, Clin Cancer Res. 24 (2018) 3186–96.

[4] Singh, A.B.; Sharma, A.; Smith, J.J.; Krishnan, M.; Chen, X.; Escheich, S.; Washington, M.K.; Yeatman, T.J.; Beauchamp, R.D.; Dhawan, P. Claudin-1 up-regulates the repressor ZEB-1 to inhibit E-Cadherin expression in colon cancer cells. *Gastroenterology.* **2011**, *141* 2140–2153.

[5] Tan, E.J.; Kahata, K.; Idås, O.; Thuault, S.; Heldin, C.H.; Moustakas, A. The high mobility group A2 protein epigenetically silences the *Cdh1* gene during epithelial-to-mesenchymal transition. *Nucleic Acids Res.* **2015**, *43*, 162–178.

[6] Zhou, Z.; Qutaish, M.; Han, Z.; Schur, R.M.; Liu, Y.; Wilson, D.L.; Lu, Z.-R. LMRI detection of breast cancer micrometastases with a fibronectin-targeting contrast agent. *Nat Commun.* **2015**, *6*, 7984.

[7] Sara, M.R.; Vera, M.G.; Catarina, G.T.; Cláudia, M.L.; João, L.; Diana, M.; Paula, C.D.; Helene, N.K.; Isabelle, B.P.; Rui, H.; Carmen, J. Vimentin epigenetic deregulation in bladder cancer associates with acquisition of invasive and metastatic phenotype through epithelial-to-mesenchymal transition. *Int J. Biol. Sci.* **2023**, *19*, 1-12.
